# Supplementary material for: Pericyte signaling via soluble guanylate cyclase shapes the vascular niche and microenvironment of tumors
Source: EMBO J. 2024 Mar 25;43(8):7. doi: 10.1038/s44318-024-00078-5 (PMC11021551; doi:10.1038/s44318-024-00078-5)
Supplement: Supplementary file 1 — Appendix [file 44318_2024_78_MOESM1_ESM.pdf]

Appendix

Pericyte sGC signaling shapes tumor vascular niche and tumor microenvironment

Jing Zhu, Wu Yang, Jianyun Ma, Hao He, Zhen Liu, Xiaolan Zhu, Xueyang He, Jing He, Zhan Chen, Xiaoliang Jin, Xiaohong Wang, Kaiwen He, Wu Wei, Junhao Hu

TABLE OF CONTENTS **page**

Appendix Figure

Appendix Figure S1-Pericyte-specific sGC inactivation does not affect mature blood vessel stability 2

Appendix Figure S2-Pericyte-specific sGC inactivation inhibits B16F10 and E0771 tumor growth 3

Appendix Figure S3-Pericyte-specific sGC inactivation impairs blood vessel stability in B16F10 and E0771 tumors 4

Appendix Figure S4-Cell proportion of pericytes and ECs in LLC tumors 5

Appendix Figure S5-Pericyte-specific sGC inactivation reprograms gene expression in both ECs and pericytes 6

Appendix Figure S6-The large increase in CAFs after sGC deletion 7

Appendix Figure S7-The large increase in CAFs is not derived from pericytes 8

Appendix Figure S8-CD146 is specifically expressed in tumor pericytes and ECs 9

Appendix Figure S9-ODQ treatment does not affect mature blood vessel stability 10

Appendix Figure S10-The combination of ODQ and Fruquintinib inhibits the growth of breast cancer 11

Appendix Figure S11-Expression levels of *GUCY1B1* and overall survival 12

Appendix Table

Appendix Table S1-Genotyping primers 13

Appendix Table S2-qPCR primers 14

Appendix Figure S1

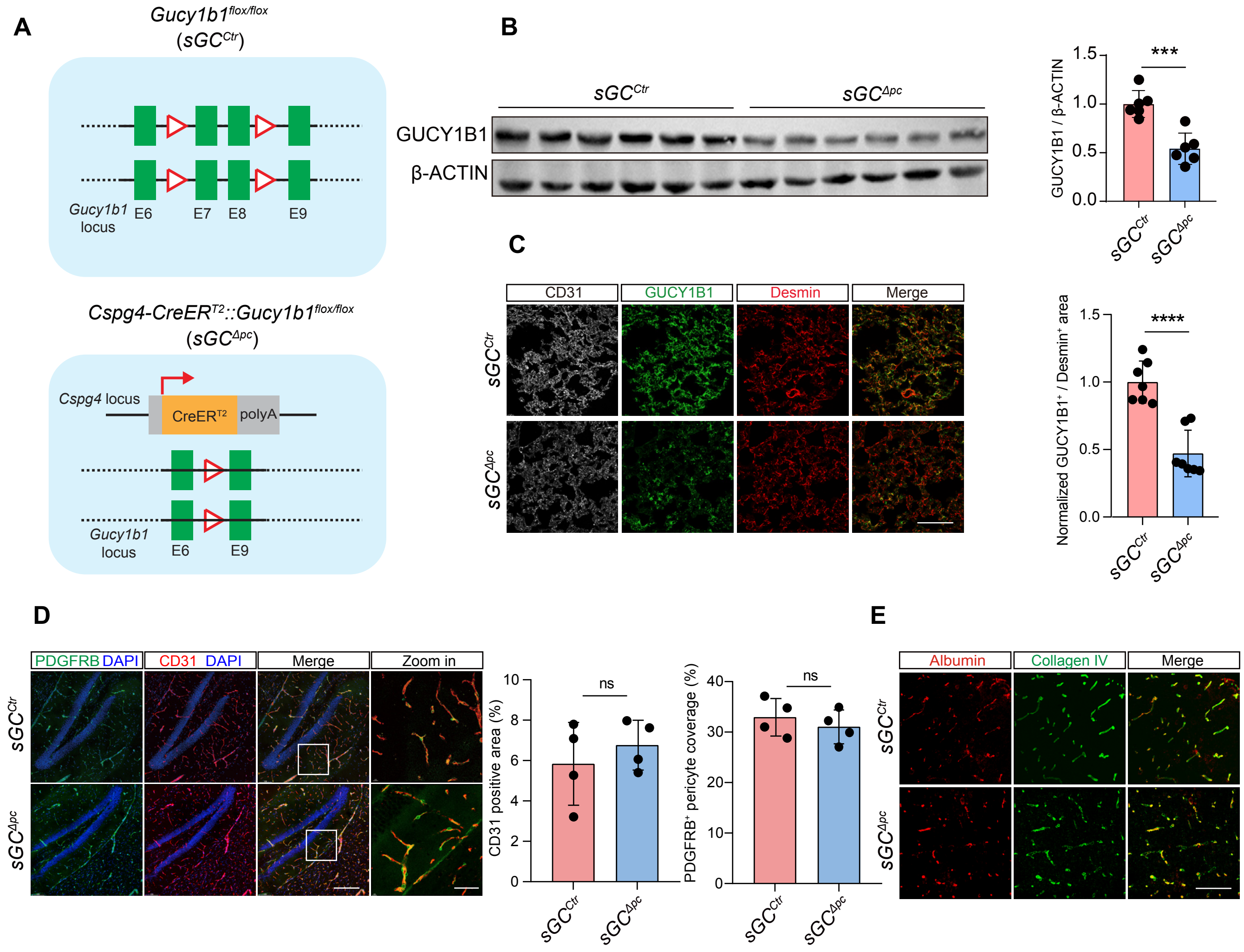

Appendix Figure S1: Pericyte-specific sGC inactivation does not affect mature blood vessel stability. (A) Schematic depiction of the construction strategy of *sGC*^{ctr} and *sGC*^{ΔPC} mice. **(B)** Western blot of GUCY1B1 protein levels in the lungs of *sGC*^{ctr} and *sGC*^{ΔPC} mice. Plots depicting the quantification of western blot results. Data presented as mean ± SD, with n = 6 mice per group. **(C)** Representative fluorescent images revealing GUCY1B1 and CD31-stained lung sections from *sGC*^{ctr} and *sGC*^{ΔPC} mice at 8 weeks of age. Plots depicting GUCY1B1 expression level. Data presented as mean ± SD, with n = 7 mice per group. Scale bar, 100 µm. **(D)** Representative fluorescence images showing CD31, DAPI, and PDGFRB-stained brain sections from *sGC*^{ctr} and *sGC*^{ΔPC} mice at 17 weeks of age. Plots depicting CD31-positive vessel area and the percentage of vessels covered by PDGFRB-positive pericytes. Data presented as mean ± SD, with n = 4 mice per group. Scale bars, 200 µm (Merge); 50 µm (Zoom in). **(E)** Representative fluorescence images revealing Albumin and Collagen IV-stained brain sections from *sGC*^{ctr} and *sGC*^{ΔPC} mice at 17 weeks of age. The absence of albumin leakage indicates vascular integrity was not impaired in both *sGC*^{ctr} and *sGC*^{ΔPC} mice. Scale bar, 100 µm. Statistical significance assessed using two-tailed Student's t test **(B-D)**. ***P < 0.001; ****P < 0.0001.

Appendix Figure S2

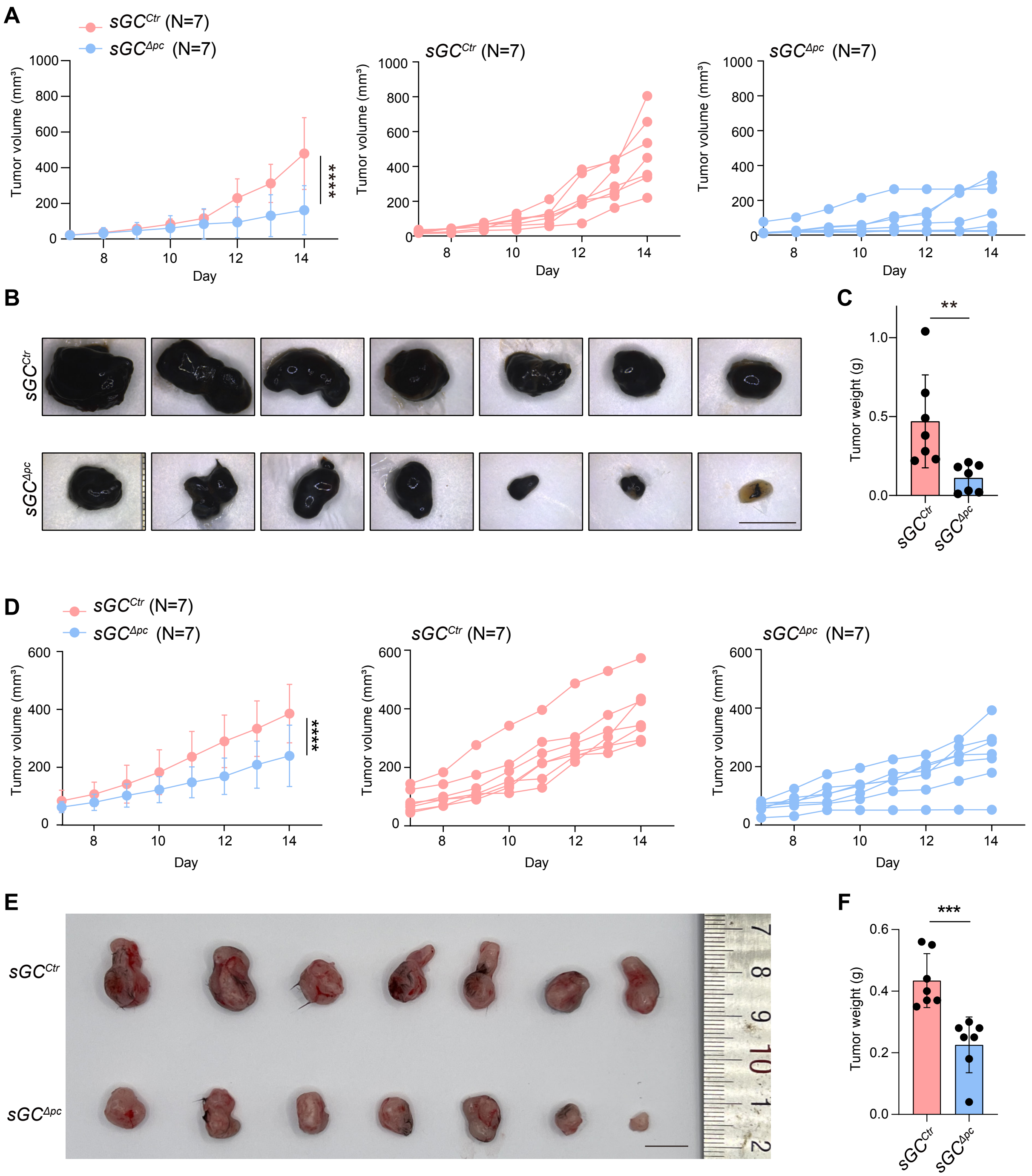

Appendix Figure S2: Pericyte-specific sGC inactivation inhibits B16F10 and E0771 tumor growth. (A) Growth curves of B16F10 tumors in sGC^{ctr} and $sGC^{\Delta PC}$ mice (left). Individual tumor growth curves for sGC^{ctr} (middle) and $sGC^{\Delta PC}$ mice (right). B16F10 tumor cells were subcutaneously injected into the right flank of male sGC^{ctr} and $sGC^{\Delta PC}$ mice.Data presented as mean ± SD, with n = 7 mice per group. **(B)** Macroscope images of B16F10 tumors isolated from sGC^{ctr} and $sGC^{\Delta PC}$ mice. Scale bar, 10 mm. **(C)** Tumor weights of B16F10 tumors isolated from sGC^{ctr} and $sGC^{\Delta PC}$ mice. Data presented as mean ± SD, with n = 7 mice per group. **(D)** Growth curves of EO771 tumors in sGC^{ctr} and $sGC^{\Delta PC}$ mice (left). Individual tumor growth curves for sGC^{ctr} (middle) and $sGC^{\Delta PC}$ mice (right). EO771 tumor cells were orthotopically injected into the mammary fat pad of the fourth gland in female sGC^{ctr} and $sGC^{\Delta PC}$ mice. Data presented as mean ± SD, with n = 7 mice per group. **(E)** Macroscope images of EO771 tumors isolated from sGC^{ctr} and $sGC^{\Delta PC}$ mice. Scale bar, 10 mm. **(F)** Tumor weights of EO771 tumors isolated from sGC^{ctr} and $sGC^{\Delta PC}$ mice. Data presented as mean ± SD, with n = 7 mice per group. Statistical significance assessed using two-way ANOVA test with Tukey test **(A, D)** or Two-tailed Student's t test **(C, F)**. **P < 0.01; ***P < 0.001; ****P < 0.0001.

Appendix Figure S3

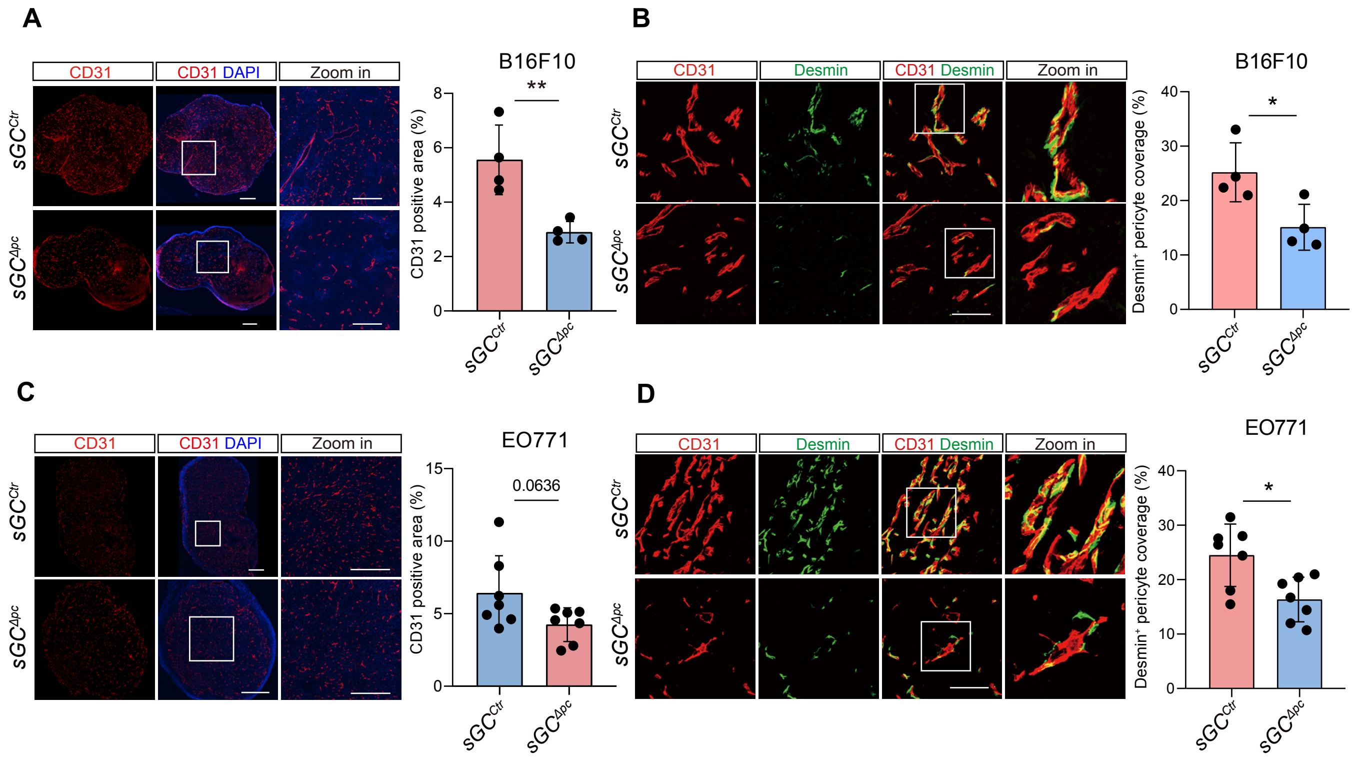

Appendix Figure S3: Pericyte-specific sGC inactivation impairs blood vessel stability in B16F10 and E0771 tumors. (A) Representative fluorescence images showing CD31 and DAPI-stained B16F10 tumor sections from *sGC*^{ctr} and *sGC*$^{\Delta PC}$ mice. Plot showing the percentage of CD31-positive vessel area. Data presented as mean ± SD, with n = 4 mice per group. Scale bars, 1 mm (CD31 DAPI); 500 µm (Zoom in). **(B)** Representative fluorescence images displaying CD31 and Desmin-stained B16F10 tumor sections from *sGC*^{ctr} and *sGC*$^{\Delta PC}$ mice. Plot showing the percentage of vessels covered by Desmin-positive pericytes. Data presented as mean ± SD, with n = 4 mice per group. Scale bar, 100 µm. **(C)** Representative fluorescence images showing CD31 and DAPI-stained EO771 tumor sections from *sGC*^{ctr} and *sGC*$^{\Delta PC}$ mice. Plot showing the percentage of CD31-positive vessel area. Data presented as mean ± SD, with n = 7 mice per group. Scale bars, 1 mm (CD31 DAPI); 500 µm (Zoom in). **(D)** Representative fluorescence images showing CD31 and Desmin-stained EO771 tumor sections from *sGC*^{ctr} and *sGC*$^{\Delta PC}$ mice. Plot showing the percentage of vessels covered by Desmin-positive pericytes. Data presented as mean ± SD, with n = 7 mice per group. Scale bar, 100 µm. Statistical significance assessed using two-tailed Student's t test **(A-D)**. *P < 0.05; **P < 0.01.

Appendix Figure S4

A

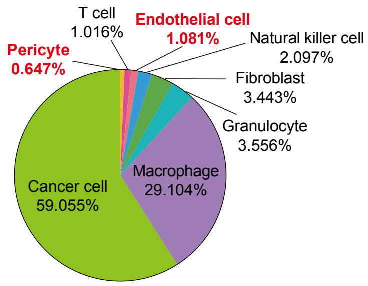

B

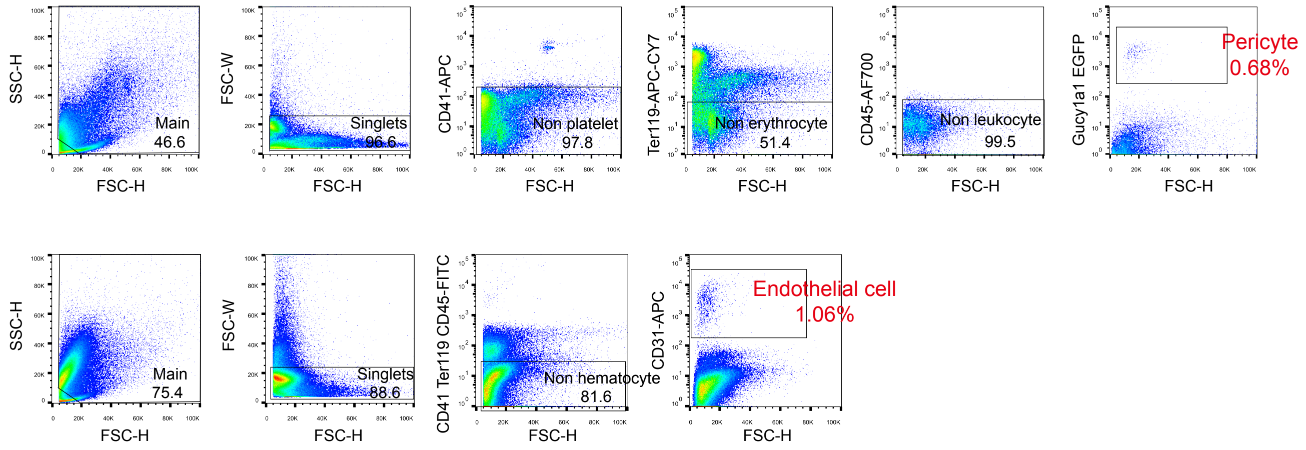

Appendix Figure S4: Cell proportion of pericytes and ECs in LLC tumors. (A) Proportion of tumor cells revealed by single-cell RNA sequencing. **(B)** FACS verification of the proportion of pericytes and ECs in LLC tumors.

Appendix Figure S5

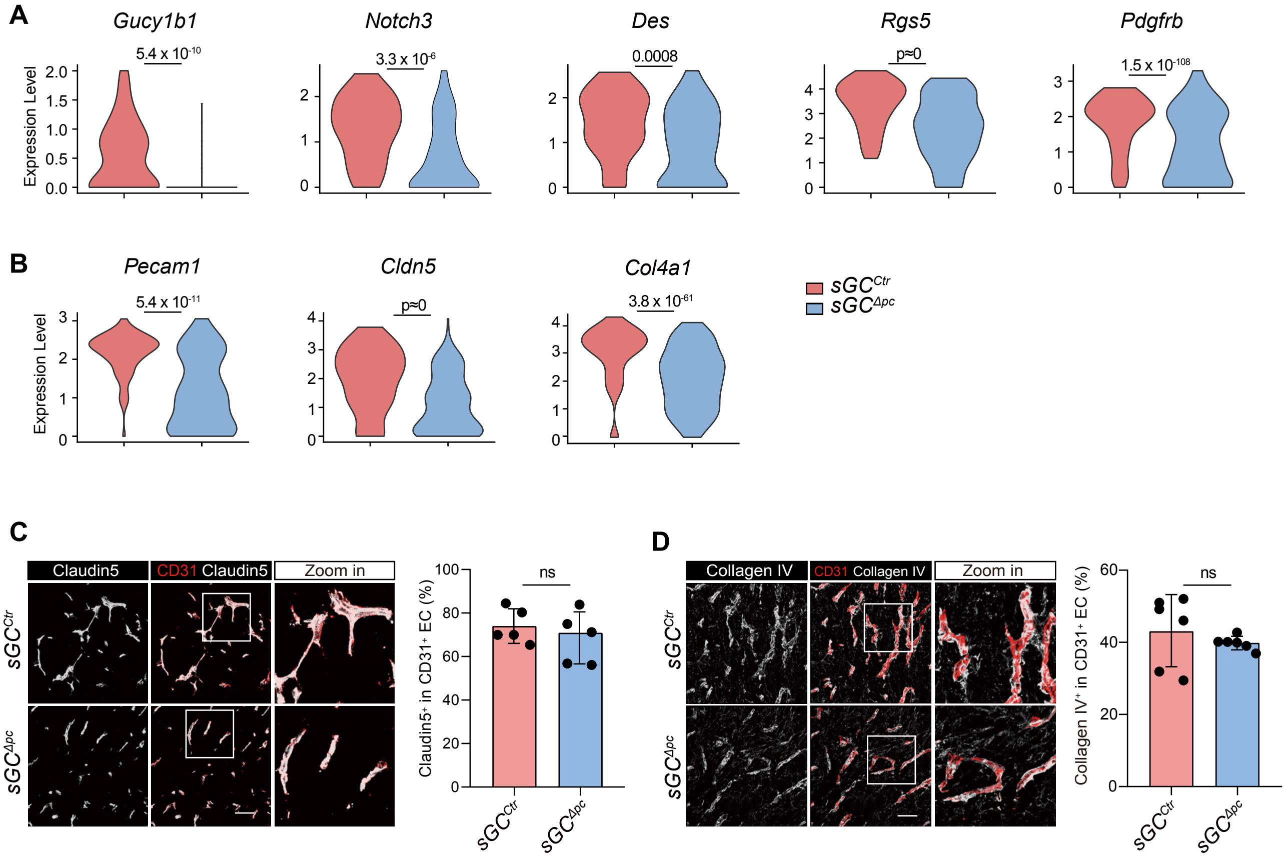

Appendix Figure S5: Pericyte-specific sGC inactivation reprograms gene expression in both ECs and pericytes. (A) Violin plots showing expression levels of *Gucy1b1*, *Notch3*, *Des*, *Rgs5*, and *Pdgfrb* in pericytes. **(B)** Violin plots displaying expression levels of *Pecam1*, *Cldn5*, and *Col4a1* in ECs. **(C)** Representative fluorescence images showing CD31 and Claudin5-stained tumor sections from sGC^{ctr} and $sGC^{\Delta PC}$ mice. Plots showing the proportion of Claudin5⁺ in CD31⁺ ECs, Data presented as mean ± SD, with n = 5 mice per group. Scale bar, 100 μm. **(D)** Representative fluorescence images showing CD31 and Collagen IV-stained tumor sections from sGC^{ctr} and $sGC^{\Delta PC}$ mice. Plots showing the proportion of Collagen IV^+ in $CD31^+$ ECs, Data presented as mean ± SD, with n = 6 mice per group. Scale bar, 100 μm. Statistical significance assessed using unpaired two-samples Wilcoxon test **(A, B)**, two-tailed Student's t test **(C, D)**.

Appendix Figure S6

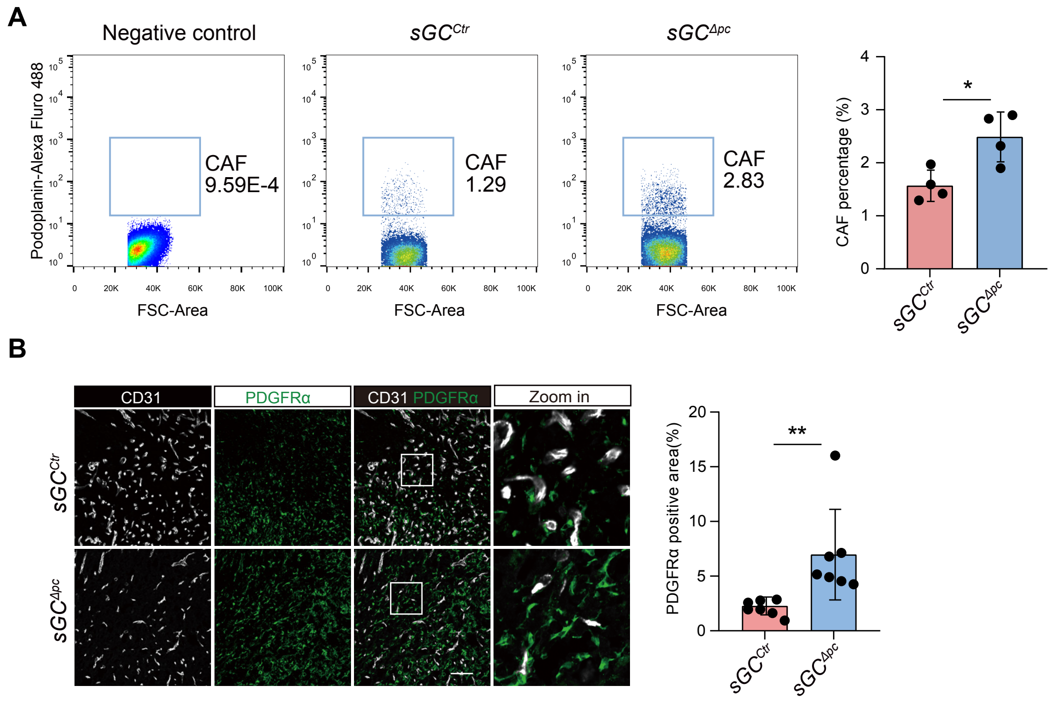

Appendix Figure S6: The large increase in CAFs after sGC deletion. (A) Representative FACS gates for analyzing PDPN-positive CAFs. Tumor cells were stained with antibodies against PDPN, Ter119, CD45, CD41, and the cell-impermeable DNA dye 7-AAD. Dead cells were excluded by gating on 7-AAD-positive cells. Subsequently, erythrocytes, leukocytes, and platelets were excluded through negative gating on Ter119-, CD45-, and CD41-positive cells. Finally, PDPN-positive cells were gated as CAFs. The plot depicts the percentage of PDPN-positive CAFs in tumors of sGC^{ctr} and $sGC^{\Delta PC}$ mice. Data are presented as mean ± SD, with n = 4 mice per group. **(B)** Representative fluorescence images showing CD31, PDGFRα-stained tumor sections. Plots depicting PDGFRα-positive area. Data presented as mean ± SD, with n = 7 mice per group. Scale bar, 100 µm. Statistical significance assessed using two-tailed Student's t test **(A, B)**. *P < 0.05; **P < 0.01.

Appendix Figure S7

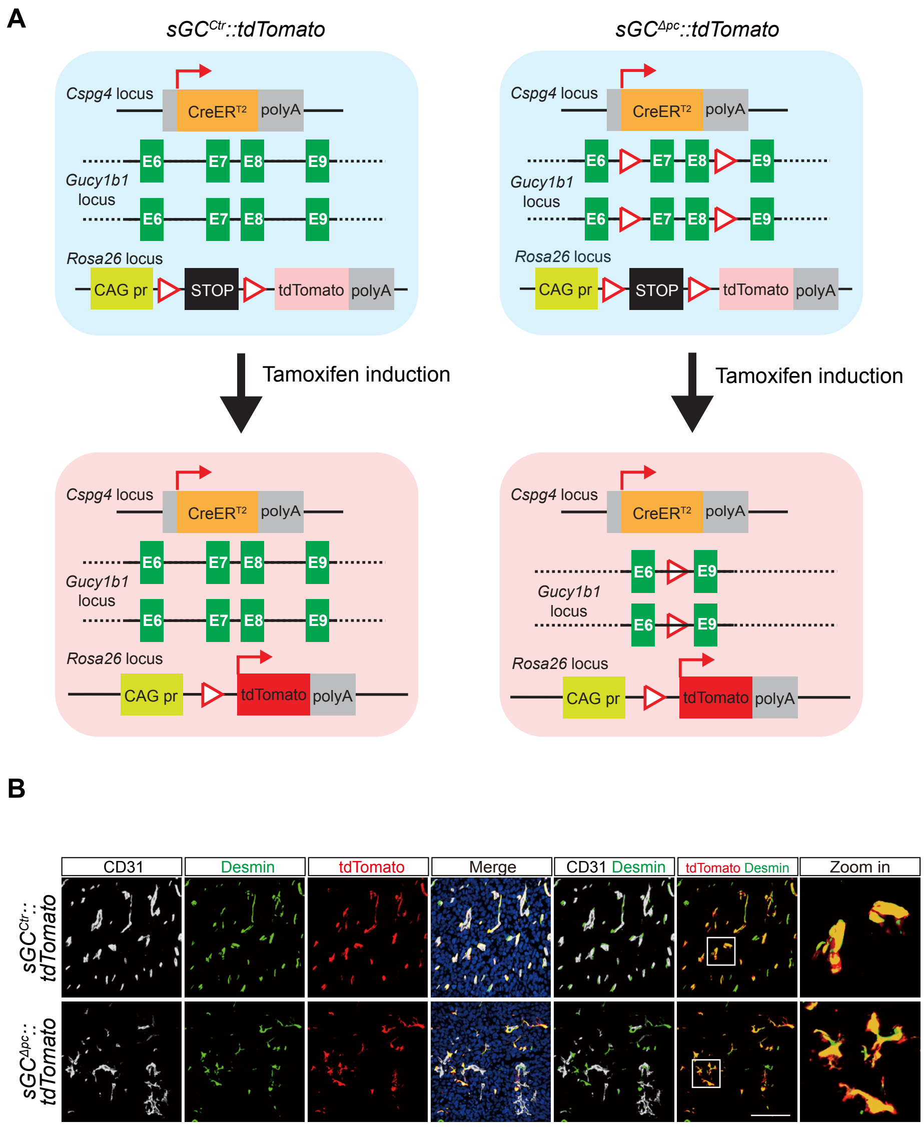

Appendix Figure S7: The large increase in CAFs is not derived from pericytes. (A) Schematic depiction of the construction strategy of *sGC*^{ctr}*::tdTomato* and *sGC*$^{\Delta PC}$*::tdTomato* mice. **(B)** Representative fluorescence images showing CD31, tdTomato, and Desmin-stained LLC tumor sections from *sGC*^{ctr}*::tdTomato* and *sGC*$^{\Delta PC}$*::tdTomato* mice. Scale bar, 100 μm.

Appendix Figure S8

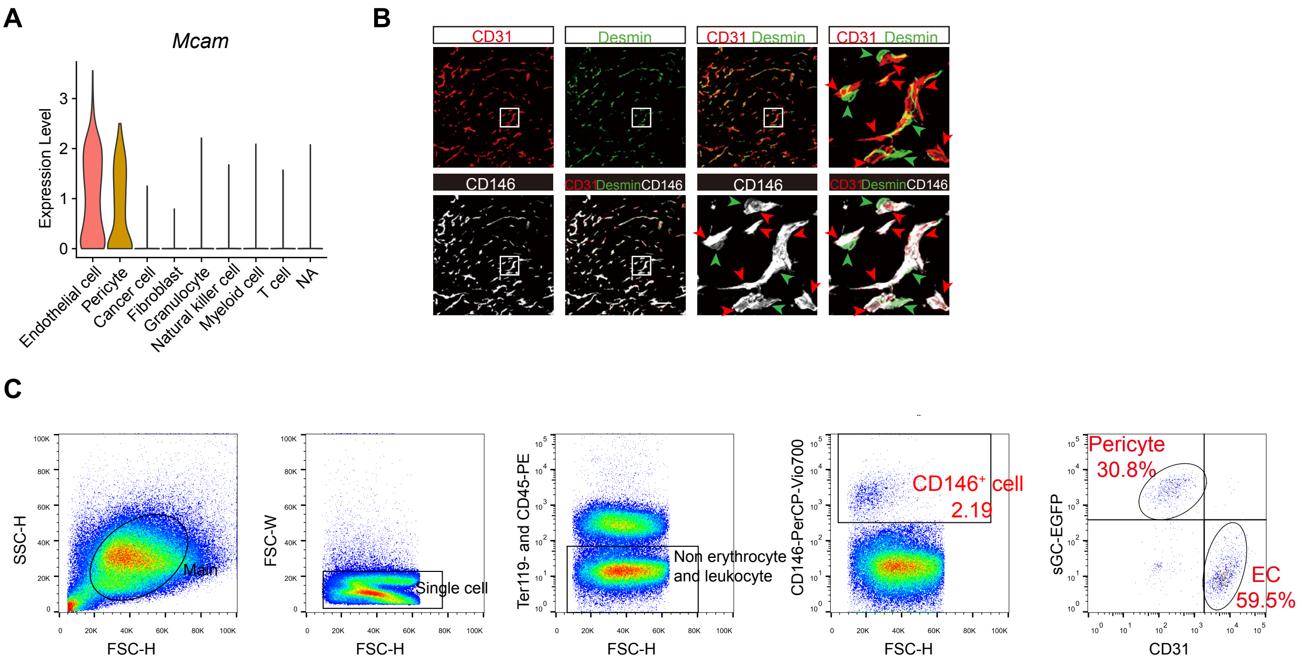

Appendix Figure S8: CD146 is specifically expressed in tumor pericytes and ECs. (A) Violin plots showing the expression levels of *Mcam*, which encodes CD146. **(B)** Representative fluorescence images showing CD31, CD146, and Desmin-stained LLC tumor sections from wild-type mice. Scale bar, 100 μm. **(C)** FACS analysis confirming that CD146-positive cells within LLC tumors corresponded to pericytes and ECs. LLC tumors were isolated from *sGC-EGFP* mice.

Appendix Figure S9

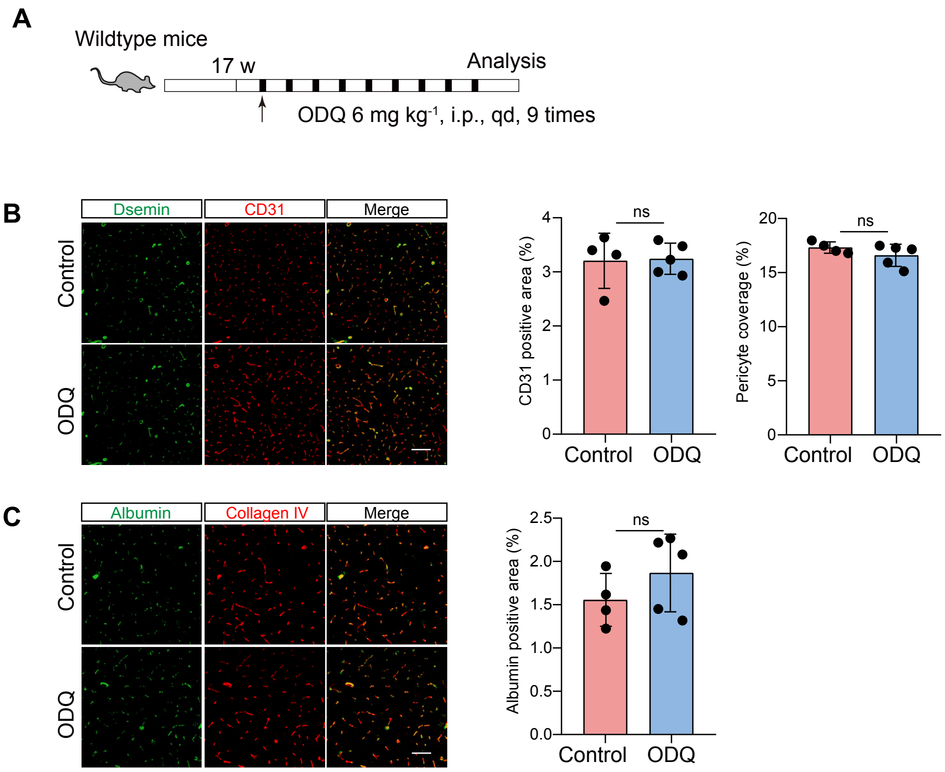

Appendix Figure S9: ODQ treatment does not affect mature blood vessel stability. (A) Schematic depiction of the experimental design. Wild-type mice were received intraperitoneal injection of 6 mg kg-1 ODQ or corresponding vehicle daily at 17 weeks of age, with brain analysis after nine times treatment. **(B)** Representative fluorescence images showing CD31, Desmin-stained brain sections in wild-type mice with ODQ treatment. Plots depicting CD31-positive vessel area and the percentage of vessels covered by Desmin-positive pericytes. Data presented as mean ± SD, with n = 4-5 mice per group. Scale bar, 100 μm. **(C)** Representative fluorescence images revealing albumin and Collagen IV-stained brain sections in wild-type mice with ODQ treatment. Plots depicting the percentage of albumin-positive vessel area. Data presented as mean ± SD, with n = 4-5 mice per group. Scale bar, 100 μm. Statistical significance assessed using Two-tailed Student's t test **(B, C)**.

Appendix Figure S10

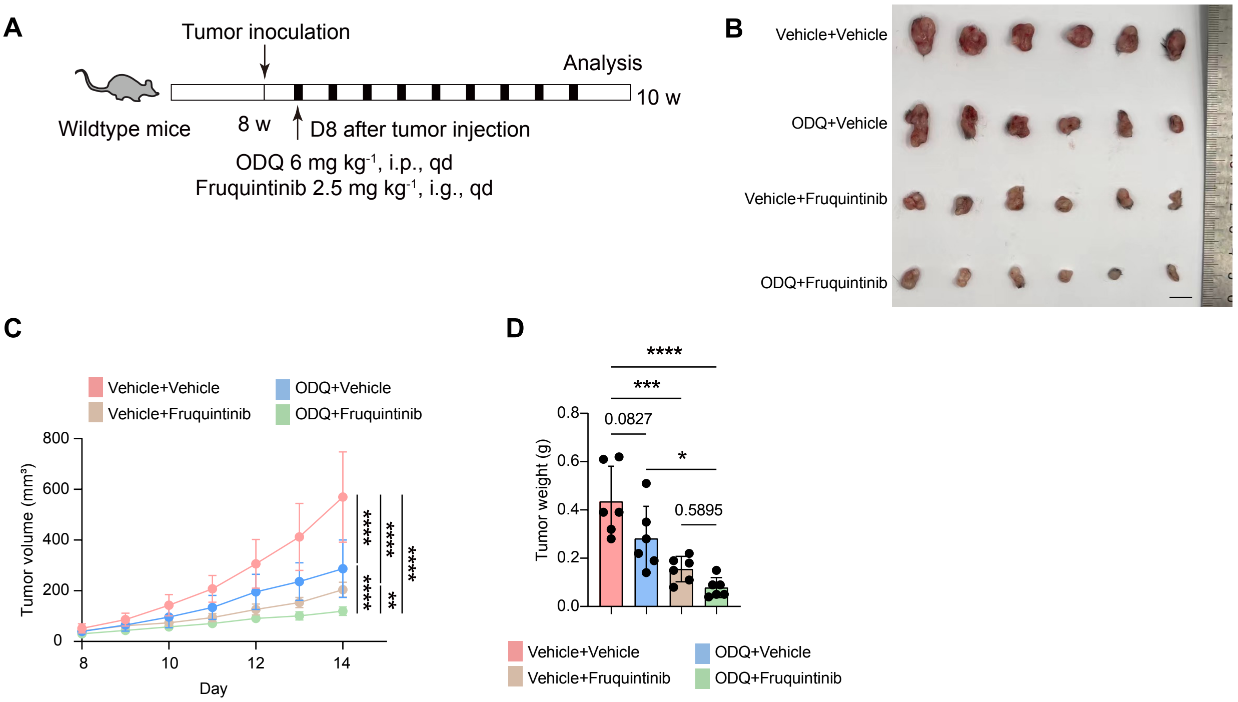

Appendix Figure S10: The combination of ODQ and Fruquintinib inhibits the growth of breast cancer. (A) Schematic depiction of the experimental design. EO771 tumor cells were orthotopically injected into the mammary fat pad of the fourth gland in female mice at 8 weeks of age. The mice then received oral administration of 2.5 mg kg^{-1} fruquintinib or the corresponding vehicle, along with intraperitoneal injection of 6 mg kg^{-1} ODQ or the corresponding vehicle, both starting from day 8 post-tumor inoculation, and analysis was performed at day 14 post-inoculation. **(B)** Macroscope images of EO771 tumors isolated from fruquintinib- or ODQ- treated mice. Scale bar, 10 mm. **(C)** Plot depicting the growth curves of EO771 tumors in wild-type mice with fruquintinib or ODQ treatment. Data presented as mean ± SD, with n = 6 mice per group. **(D)** Plot showing EO771 tumor weights following fruquintinib or ODQ treatment. Data presented as mean ± SD, with n = 6 mice per group. Statistical significance assessed using two-way ANOVA test **(C)** or One-way ANOVA test with Tukey test **(D)**. *P < 0.05; **P < 0.01; ***P < 0.001; ****P < 0.0001.

Appendix Figure S11

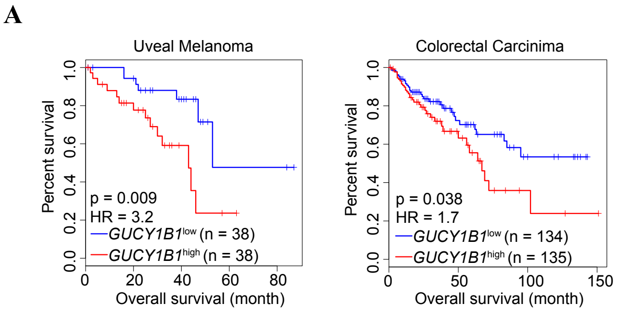

Appendix Figure S11: Expression levels of *GUCY1B1* and overall survival. (A) Overall survival of uveal melanoma, colorectal carcinoma, and mesothelioma patients selected from TCGA dataset and stratified by the expression levels of *GUCY1B1*.

Appendix Table S1: Genotyping primers

Primer name	Primer sequence
Gucy1a1-EGFP-F	GTCAGAAGGAAGCCACTGCCAA
Gucy1a1-EGFP-R	TAGCGGCTGAAGCACTGCA
Gucy1b1-flox-F	TAAGAGCAGTCAGCTTTCACT
Gucy1b1-flox-R	TTCTGCTTCCACATCTACAGCT
Cspg4 CreER^{T2}-F1	GGCAAACCCAGAGCCCTGCC
Cspg4 CreER^{T2}-F2	GCTGGAGCTGACAGCGGGTG
Cspg4 CreER^{T2}-R	GCCCGGACCGACGATGAAGC

Appendix Table S2: qPCR primers

Primer name	Primer sequence	Species
GUCY1A1-F	TCAGCCCTACTTGTTGTACTCC	Human
GUCY1A1-R	CAGAATAGCGATGTGGGAATCAC	Human
GUCY1B1-F	GCTGCTTACGTCTCAAGGGTC	Human
GUCY1B1-R	GAACAAGATCGCGCGTGGCATC	Human
GAPDH-F	ATGACATCAAGAAGGTGGTG	Human
GAPDH-R	CATACCAGGAAATGAGCTTG	Human
PLVAP-F	CTCTTCATGGTCTATGGCAACG	Human
PLVAP-R	GCGAGCATTCAGCCACATC	Human
ITGA1-F	CTGGACATAGTCATAGTGCTGGA	Human
ITGA1-R	ACCTGTGTCTGTTTAGGACCA	Human
TSPAN15-F	AGGACTACCGAGATTGGAGCA	Human
TSPAN15-R	CAACTTCTGTCGTGTTCCTGAT	Human
CSPG4-F	CTTTGACCCTGACTATGTTGGC	Human
CSPG4-R	TGCAGGCGTCCAGAGTAGA	Human
ABCC9-F	CCAGCCGTGATGGGATTCG	Human
ABCC9-R	GGCCATTACCCAATACAGGAAC	Human
PDE3A-F	GAATCCCGTCACTTCGCTCAG	Human
PDE3A-R	GAAACTCGTCTCAACAAGCCA	Human
TGFB1-F	CAATTCCTGGCGATACCTCAG	Human
TGFB1-R	GCACAACTCCGGTGACATCAA	Human
VASP-F	ATCGTCCGGGGTGTCAAGTAT	Human
VASP-R	TCCTTCCAACGCCTCTAGGG	Human
EDNRB-F	GTCCCAATATCTTGATCGCCAG	Human
EDNRB-R	AAGGCACCAGCTTACACATCT	Human
ABCA1-F	ACCCACCCTATGAACAACATGA	Human
ABCA1-R	GAGTCGGGTAACGGAAACAGG	Human
FLT1-F	TTTGCCTGAAATGGTGAGTAAGG	Human
FLT1-R	TGGTTTGCTTGAGCTGTGTTC	Human
ADAMTS1-F	CAGAGCACTATGACACAGCAA	Human
ADAMTS1-R	AGCCATCCCAAGAGTATCACA	Human
JAM2-F	AACTGGGTCGGAGTGTCTC	Human
JAM2-R	GGGGCACTAACTTCACAACGA	Human

CDH13-F	AGCGATGGCGGCTTAGTTG	Human
CDH13-R	ATGGACGAACAGAGTTTTGCC	Human
Eng-F	AGGGGTGAGGTGACGTTTAC	Mouse
Eng-R	GTGCCATTTTGCTTGGATGC	Mouse
Cd74-F	GCTGGATGAAGCAGTGGCTCTT	Mouse
Cd74-R	GATGTGGCTGACTTCTTCCTGG	Mouse
Igfbp5-F	AGACAGGAATCCGAACAAGGC	Mouse
Igfbp5-R	GTAGAATCCTTTGCGGTCACA	Mouse
Cxcl14-F	AGTGTAAGTGTTCCCGGAAGG	Mouse
Cxcl14-R	GCAGTGTGGGTACTTTGGCTT	Mouse
Cd14-F	ACTTCTCAGATCCGAAGCCAG	Mouse
Cd14-R	CCGCCGTACAATTCCACAT	Mouse
Cxcl2-F	CCAACCACCAGGCTACAGG	Mouse
Cxcl2-R	GCGTCACACTCAAGCTCTG	Mouse
Tnf-α-F	CAGGCGGTGCCTATGTCTC	Mouse
Tnf-α-R	CGATCACCCCGAAGTTCAGTAG	Mouse
Il-6-F	CTGCAAGAGACTTCCATCCAG	Mouse
Il-6-R	AGTGGTATAGACAGGTCTGTTGG	Mouse
Tgfb1-F	CCACCTGCAAGACCATCGAC	Mouse
Tgfb1-R	CTGGCGAGCCTTAGTTTGGAC	Mouse
Cd163-F	GGTGGACACAGAATGGTTCTTC	Mouse
Cd163-R	CCAGGAGCGTTAGTGACAGC	Mouse
Mrc1-F	CTCTGTTCAGCTATTGGACGC	Mouse
Mrc1-R	CGGAATTTCTGGGATTCAGCTTC	Mouse
Il-10-F	CTTACTGACTGGCATGAGGATCA	Mouse
Il-10-R	GCAGCTCTAGGAGCATGTGG	Mouse
Arg1-F	TGACTGAAGTAGACAAGCTGGGGAT	Mouse
Arg1-R	CGACATCAAAGCTCAGGTGAATCGG	Mouse
Nos2-F	GGAGTGACGGCAAACATGACT	Mouse
Nos2-R	TCGATGCACAACTGGGTGAAC	Mouse
Il-1b-F	GAAATGCCACCTTTTGACAGTG	Mouse
Il-1b-R	TGGATGCTCTCATCAGGACAG	Mouse
Mif-F	GCCAGAGGGGTTTCTGTCG	Mouse
Mif-R	GTTCGTGCCGCTAAAAGTCA	Mouse

18S RNA-F	GCAATTATTCCCCATGAACG	Mouse
18S RNA-R	GGCCTCACTAAACCATCCAA	Mouse
β-Actin-F	GGCTGTATTCCCCTCCATCG	Mouse
β-Actin-R	CCAGTTGGTAACAATGCCATGT	Mouse
Gapdh-F	AGGTCGGTGTGAACGGATTTG	Mouse
Gapdh-R	TGTAGACCATGTAGTTGAGGTCA	Mouse
