# Supplementary figures and images for: Pericyte signaling via soluble guanylate cyclase shapes the vascular niche and microenvironment of tumors (part 1 of 4)
Source: EMBO J. 2024 Mar 25;43(8):7. doi: 10.1038/s44318-024-00078-5 (PMC11021551; doi:10.1038/s44318-024-00078-5)

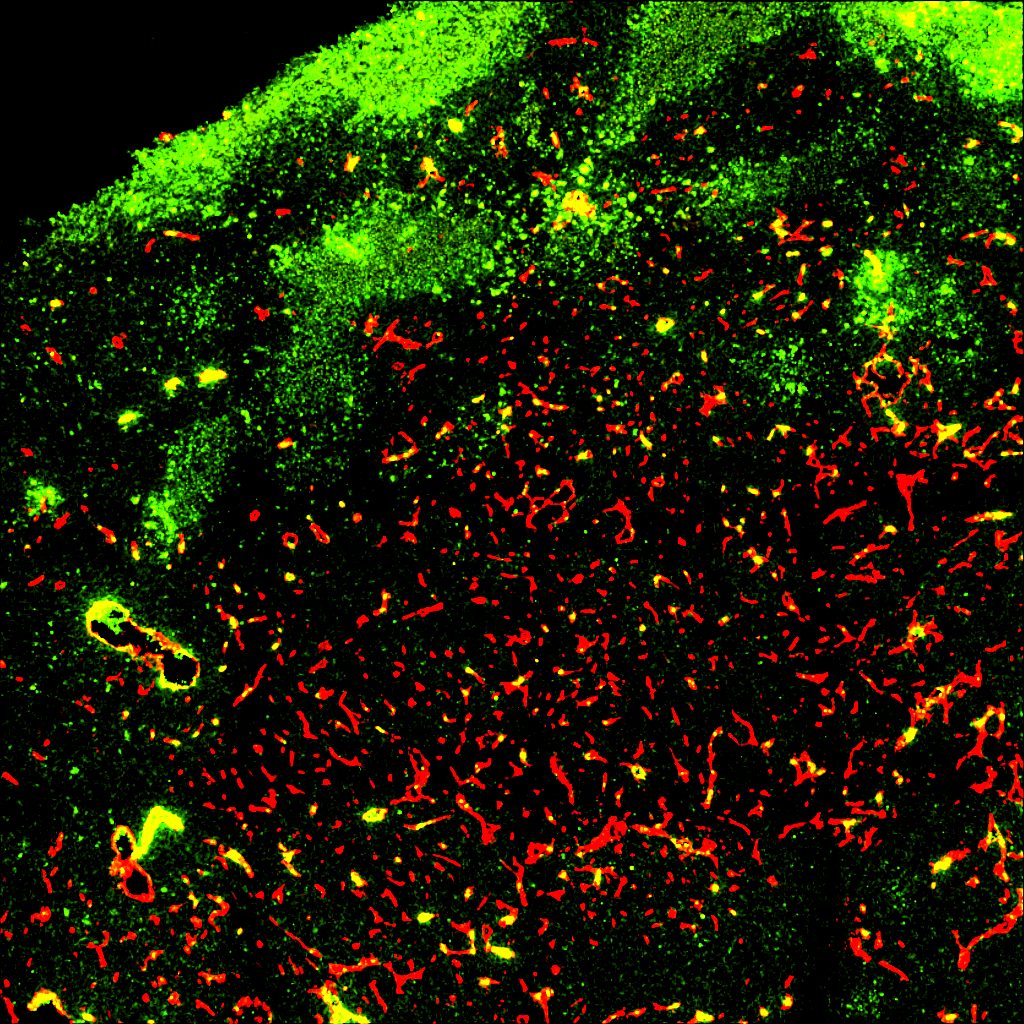

Supplement: Supplementary file 2 — Source data Fig. 1 [file 44318_2024_78_MOESM2_ESM.zip › Figure 1/1H/sGCCtr-4.tif]

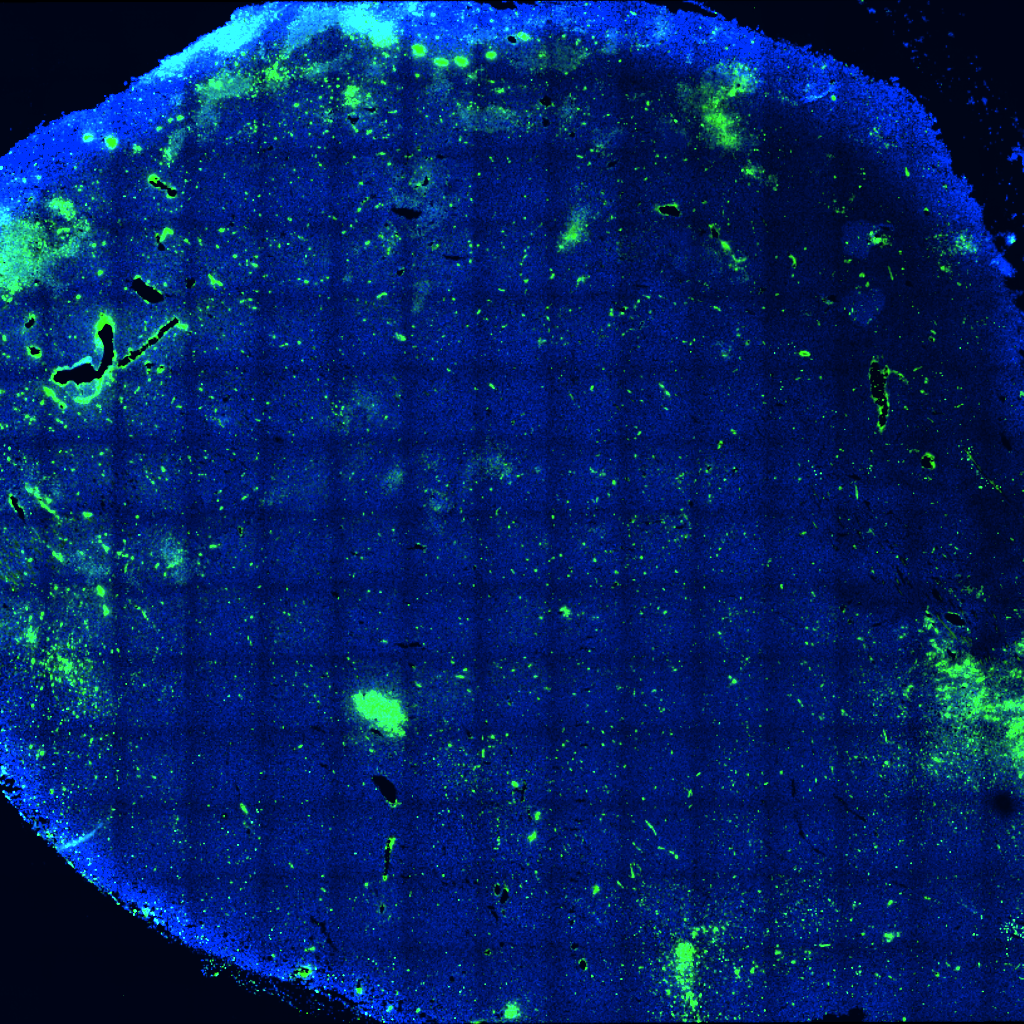

Supplement: Supplementary file 2 — Source data Fig. 1 [file 44318_2024_78_MOESM2_ESM.zip › Figure 1/1H/sGCCtr-3.tif]

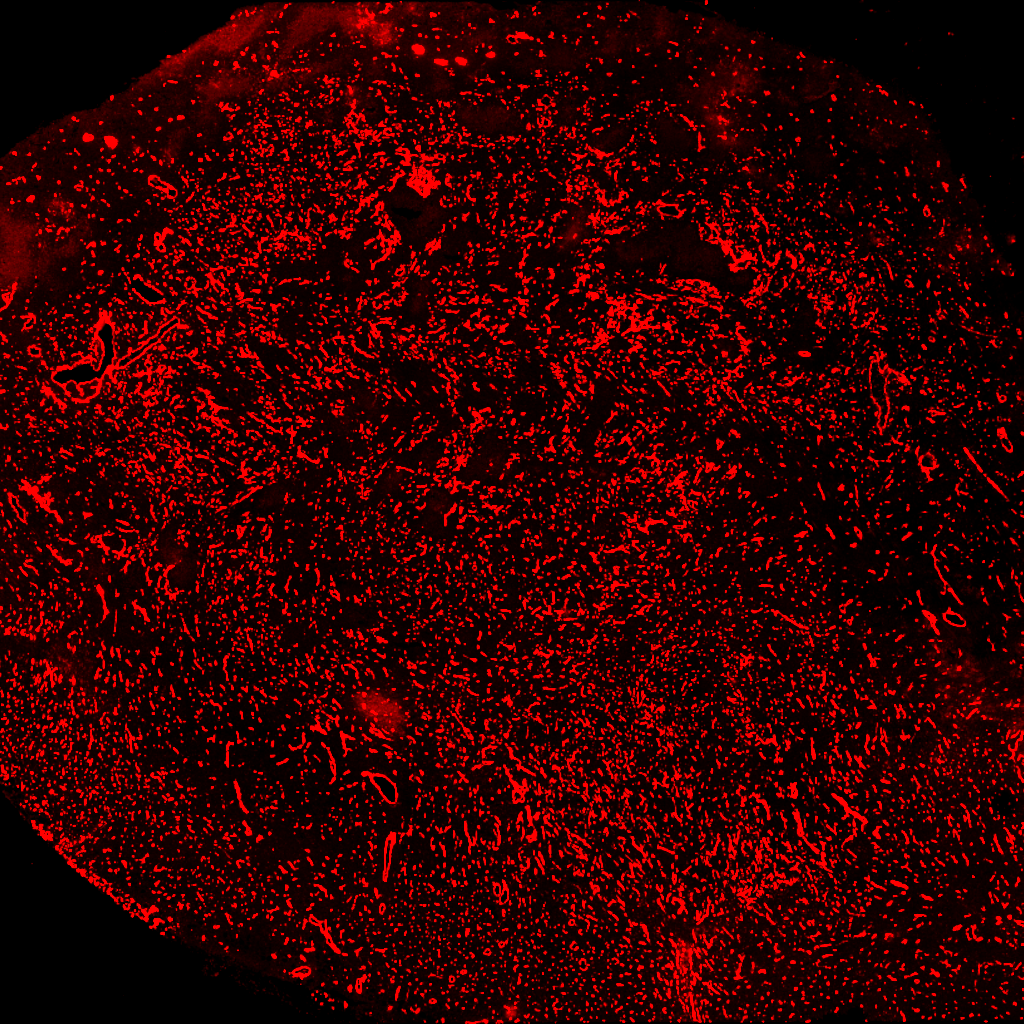

Supplement: Supplementary file 2 — Source data Fig. 1 [file 44318_2024_78_MOESM2_ESM.zip › Figure 1/1H/sGCCtr-2.tif]

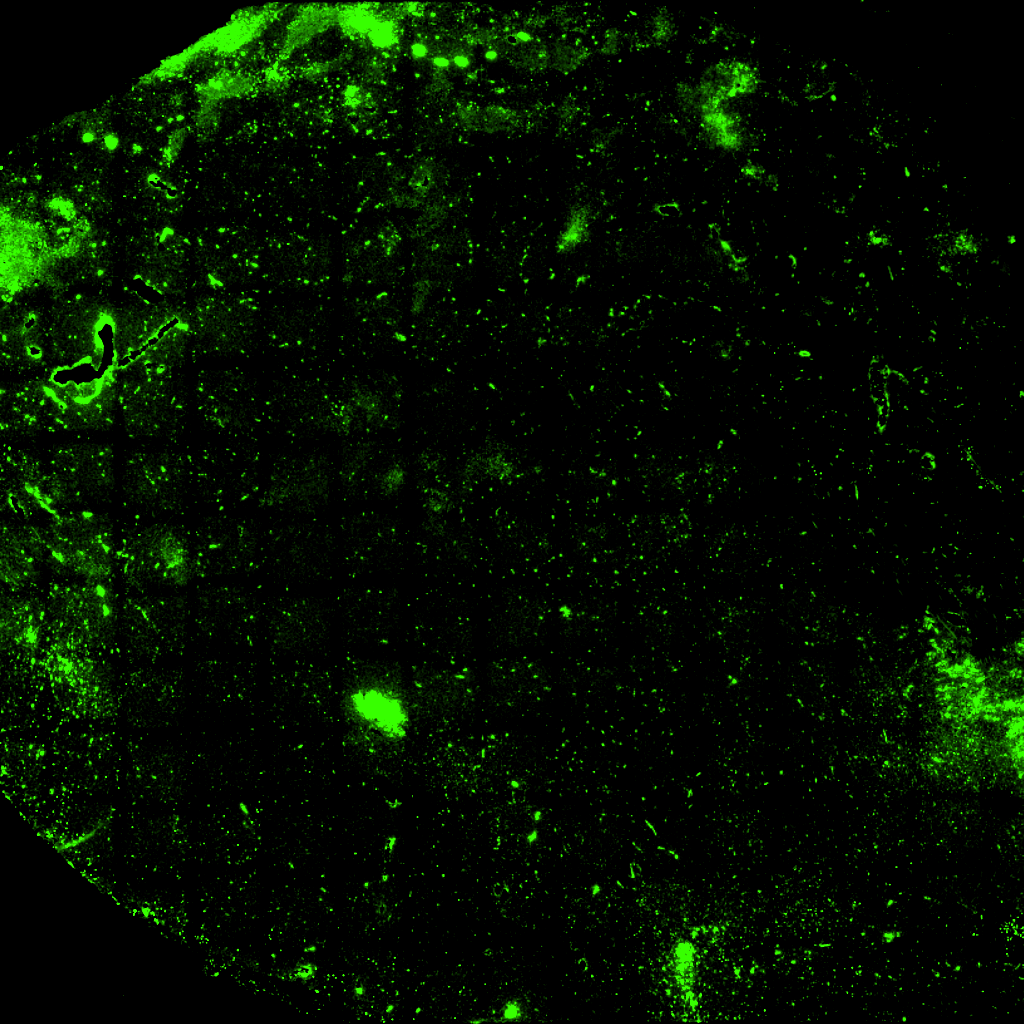

Supplement: Supplementary file 2 — Source data Fig. 1 [file 44318_2024_78_MOESM2_ESM.zip › Figure 1/1H/sGCCtr-1.tif]

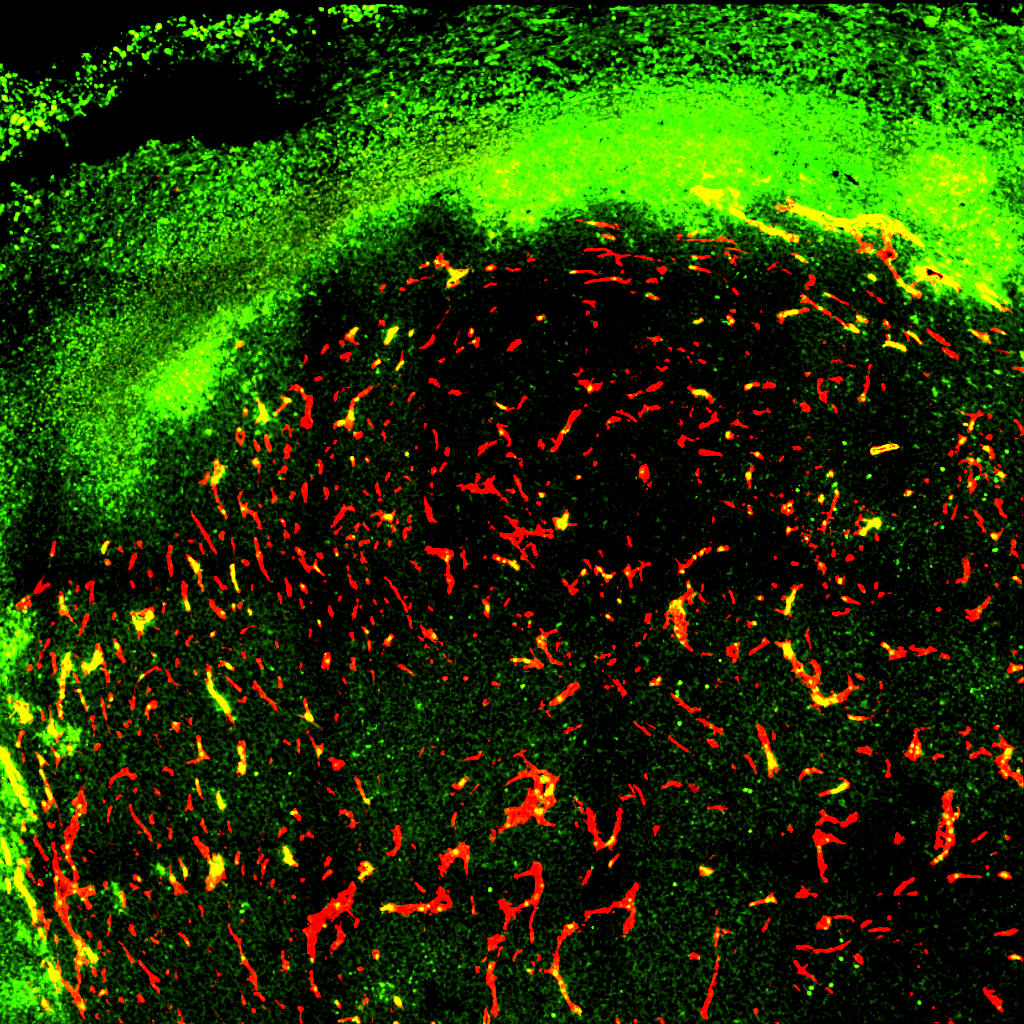

Supplement: Supplementary file 2 — Source data Fig. 1 [file 44318_2024_78_MOESM2_ESM.zip › Figure 1/1H/sGC╬öPC-4.tif]

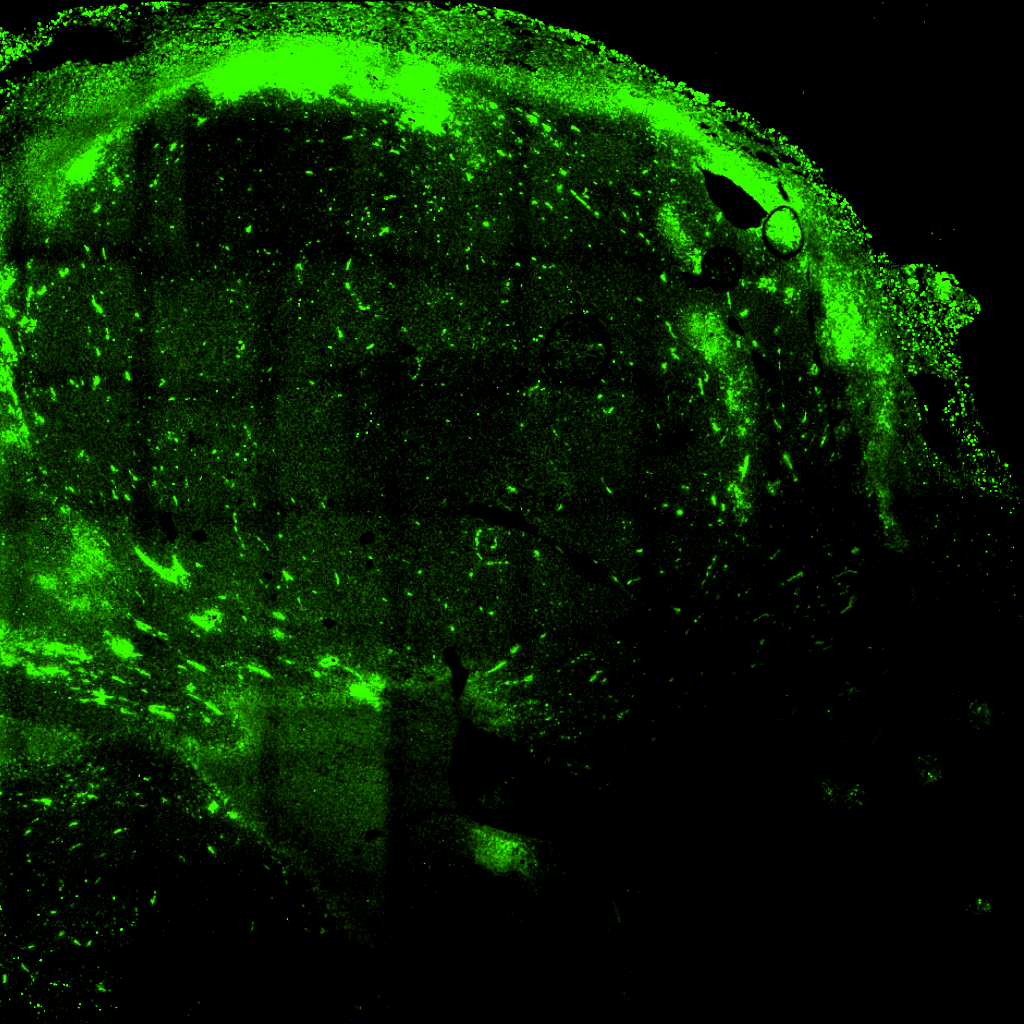

Supplement: Supplementary file 2 — Source data Fig. 1 [file 44318_2024_78_MOESM2_ESM.zip › Figure 1/1H/sGC╬öPC-1.tif]

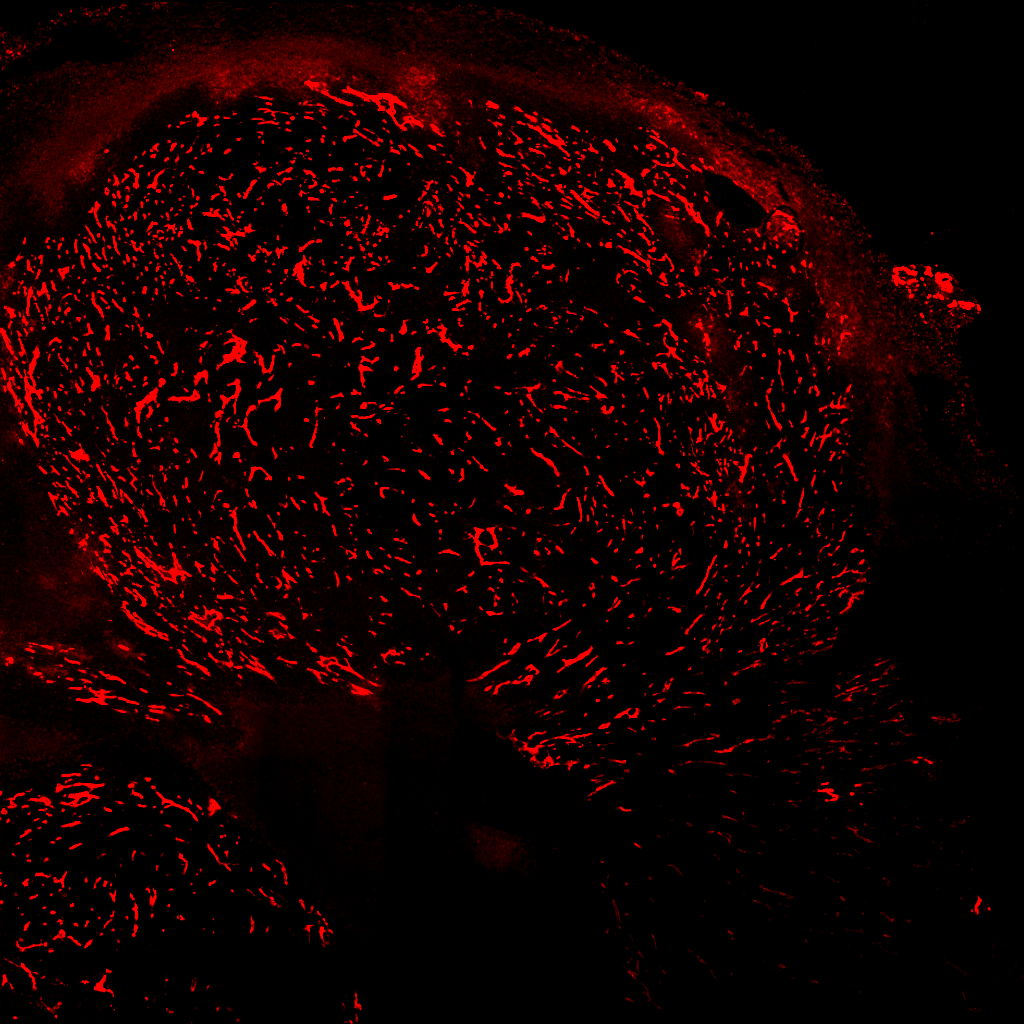

Supplement: Supplementary file 2 — Source data Fig. 1 [file 44318_2024_78_MOESM2_ESM.zip › Figure 1/1H/sGC╬öPC-2.tif]

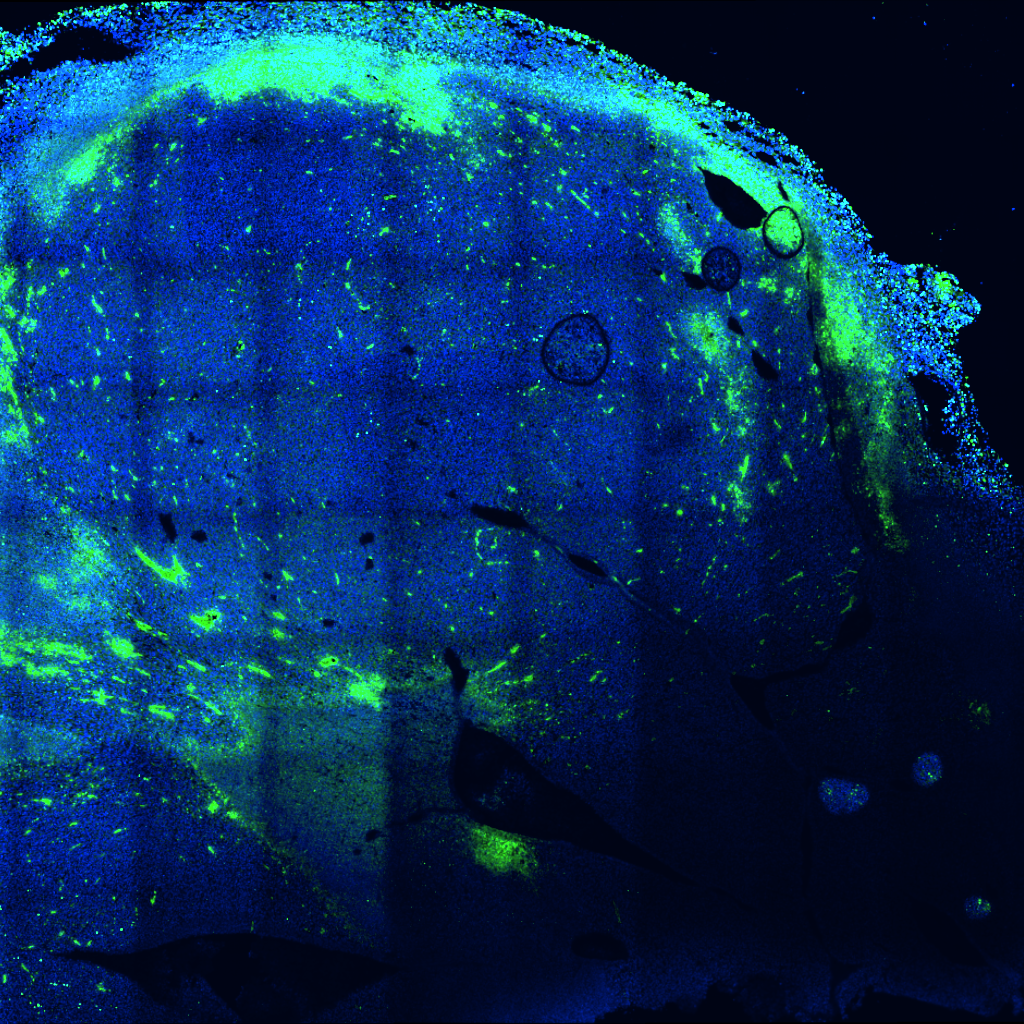

Supplement: Supplementary file 2 — Source data Fig. 1 [file 44318_2024_78_MOESM2_ESM.zip › Figure 1/1H/sGC╬öPC-3.tif]

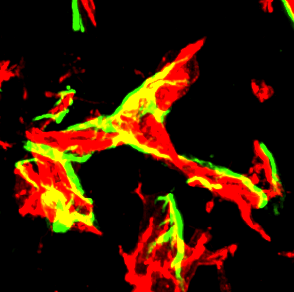

Supplement: Supplementary file 2 — Source data Fig. 1 [file 44318_2024_78_MOESM2_ESM.zip › Figure 1/1F/sGCCtr-4.tif]

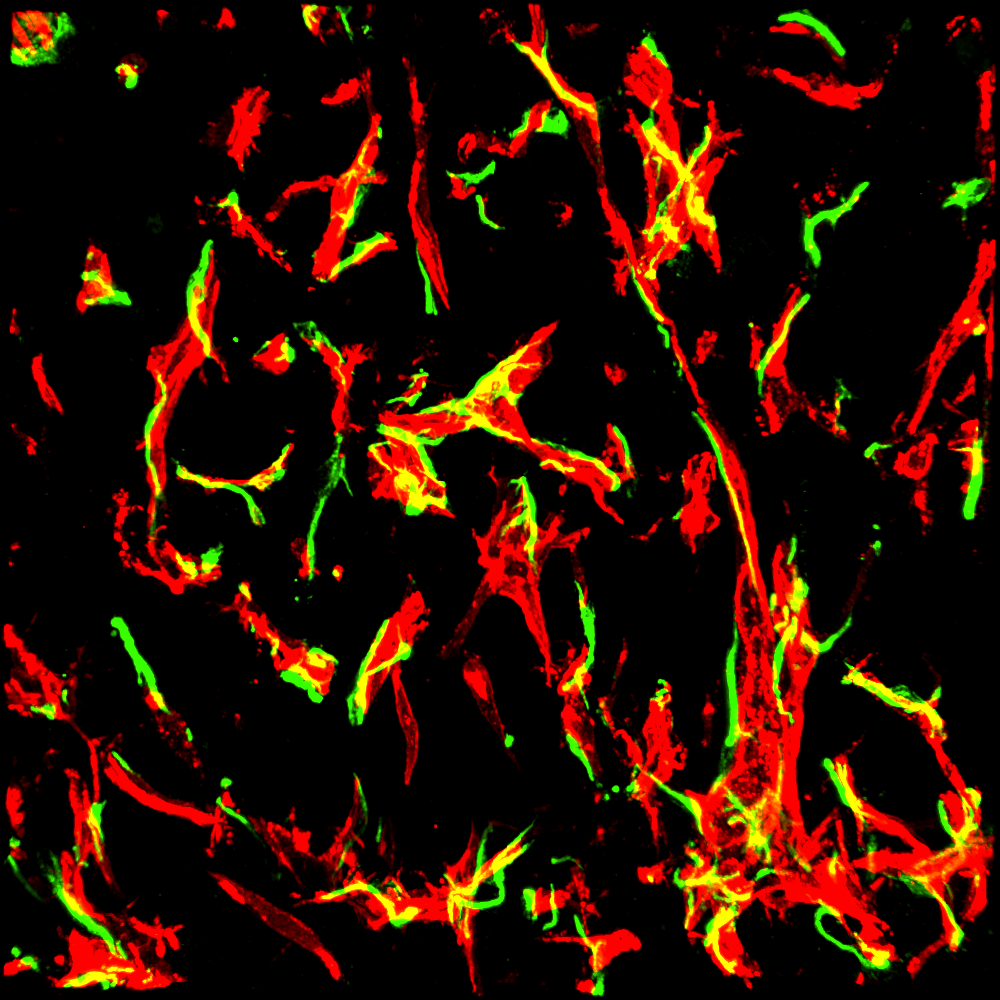

Supplement: Supplementary file 2 — Source data Fig. 1 [file 44318_2024_78_MOESM2_ESM.zip › Figure 1/1F/sGCCtr-3.tif]

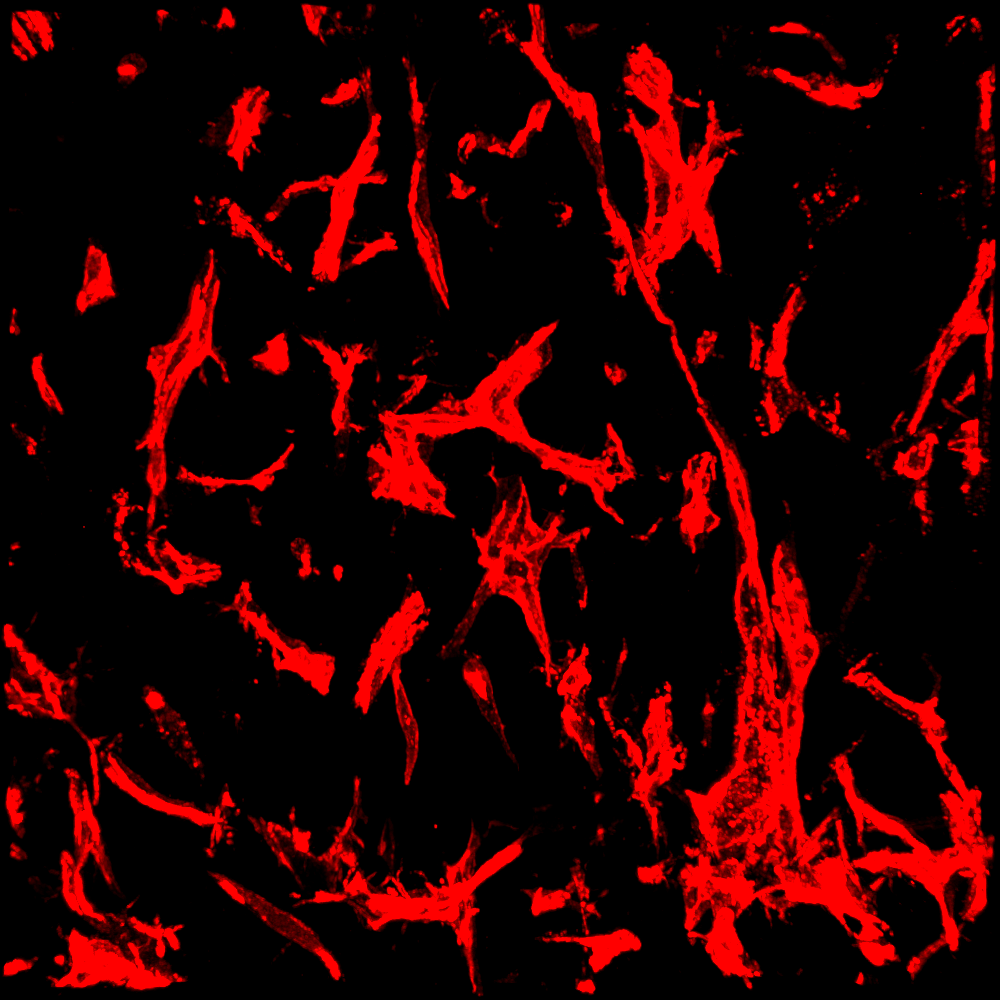

Supplement: Supplementary file 2 — Source data Fig. 1 [file 44318_2024_78_MOESM2_ESM.zip › Figure 1/1F/sGCCtr-2.tif]

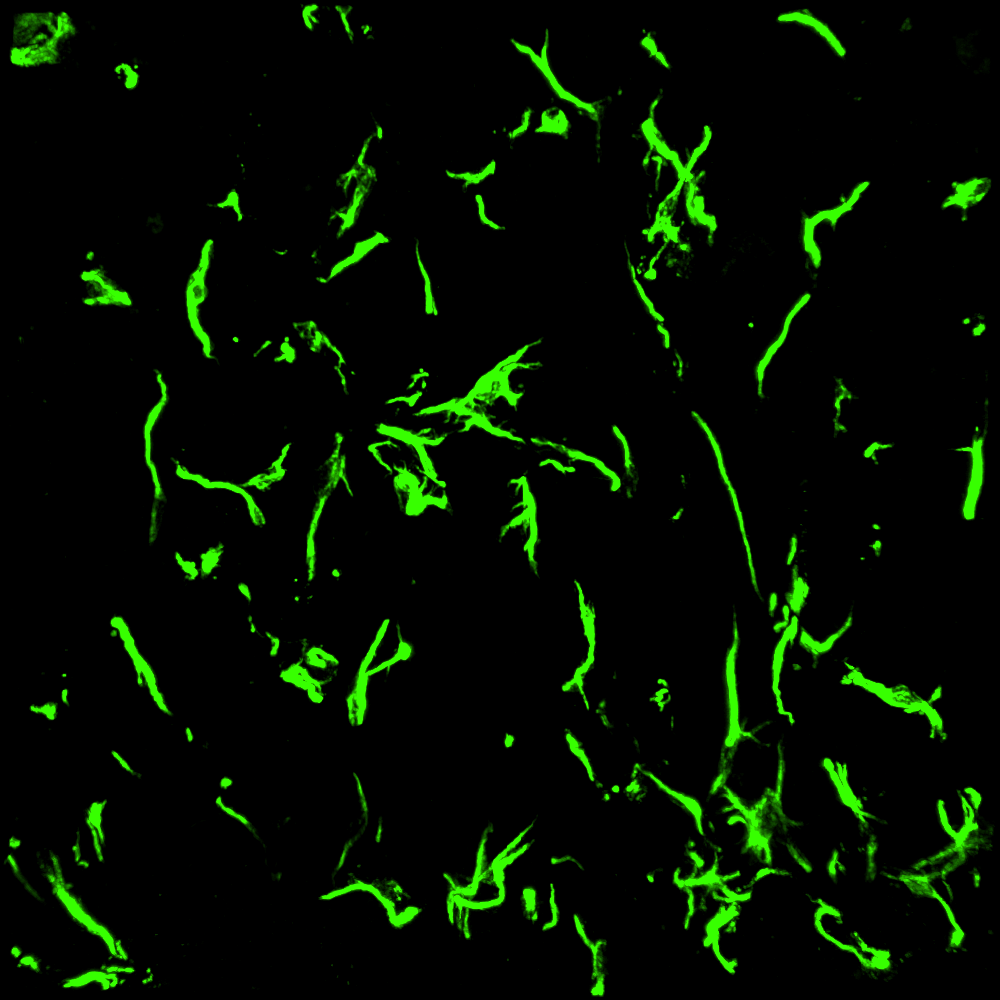

Supplement: Supplementary file 2 — Source data Fig. 1 [file 44318_2024_78_MOESM2_ESM.zip › Figure 1/1F/sGCCtr-1.tif]

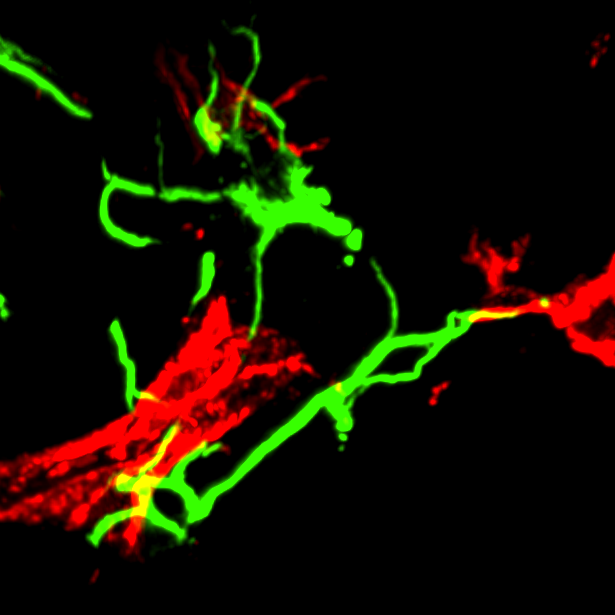

Supplement: Supplementary file 2 — Source data Fig. 1 [file 44318_2024_78_MOESM2_ESM.zip › Figure 1/1F/sGC╬öPC-4.tif]

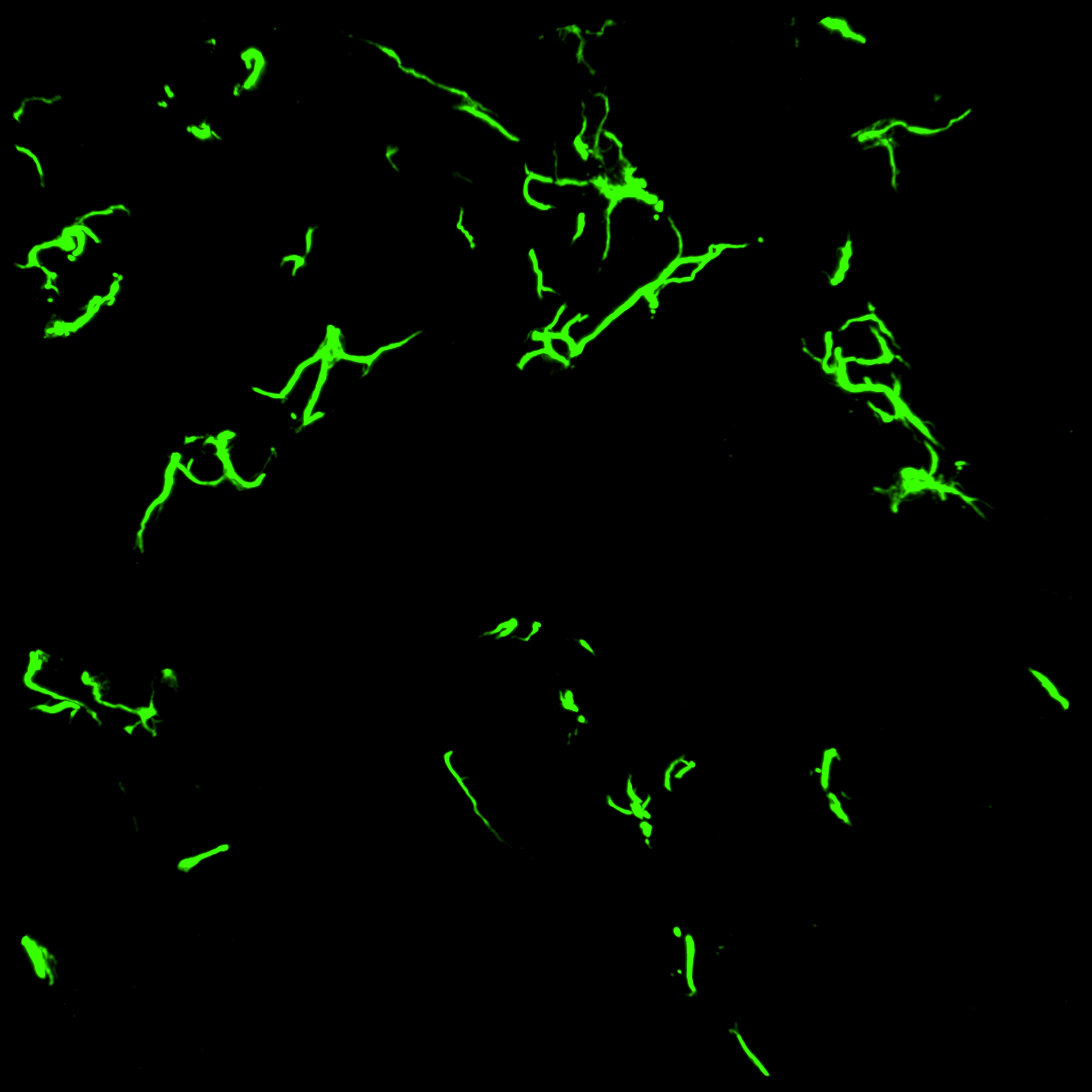

Supplement: Supplementary file 2 — Source data Fig. 1 [file 44318_2024_78_MOESM2_ESM.zip › Figure 1/1F/sGC╬öPC-1.tif]

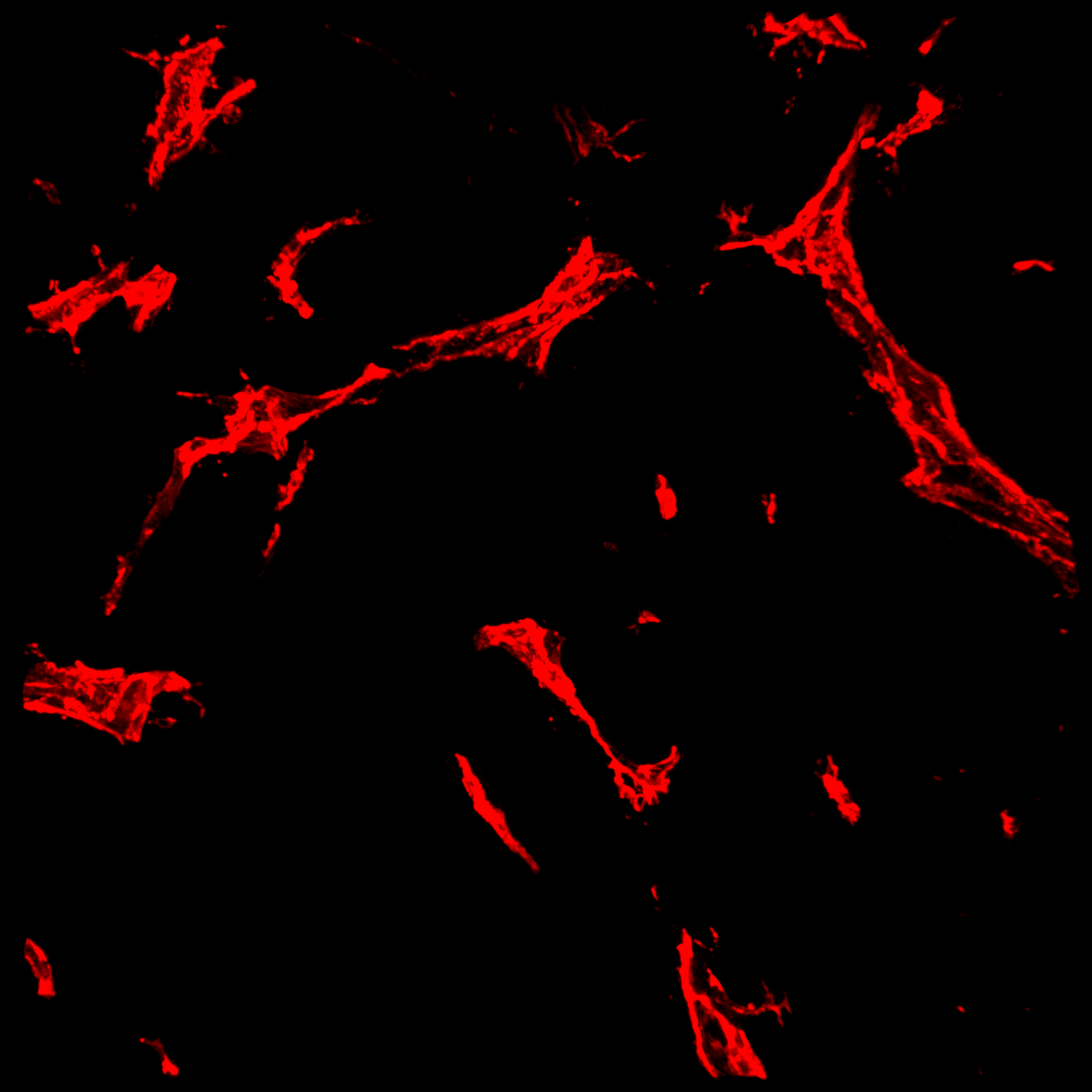

Supplement: Supplementary file 2 — Source data Fig. 1 [file 44318_2024_78_MOESM2_ESM.zip › Figure 1/1F/sGC╬öPC-2.tif]

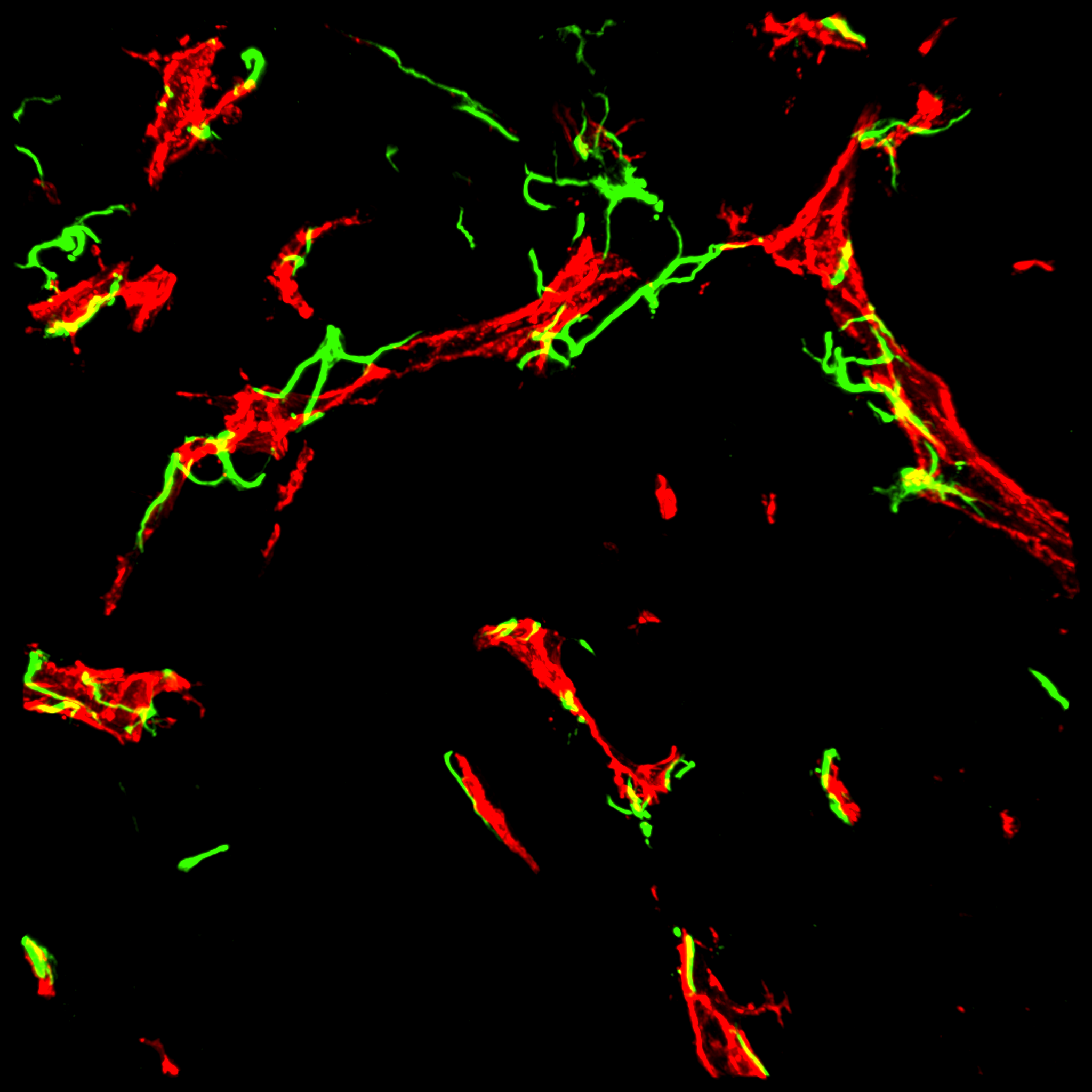

Supplement: Supplementary file 2 — Source data Fig. 1 [file 44318_2024_78_MOESM2_ESM.zip › Figure 1/1F/sGC╬öPC-3.tif]

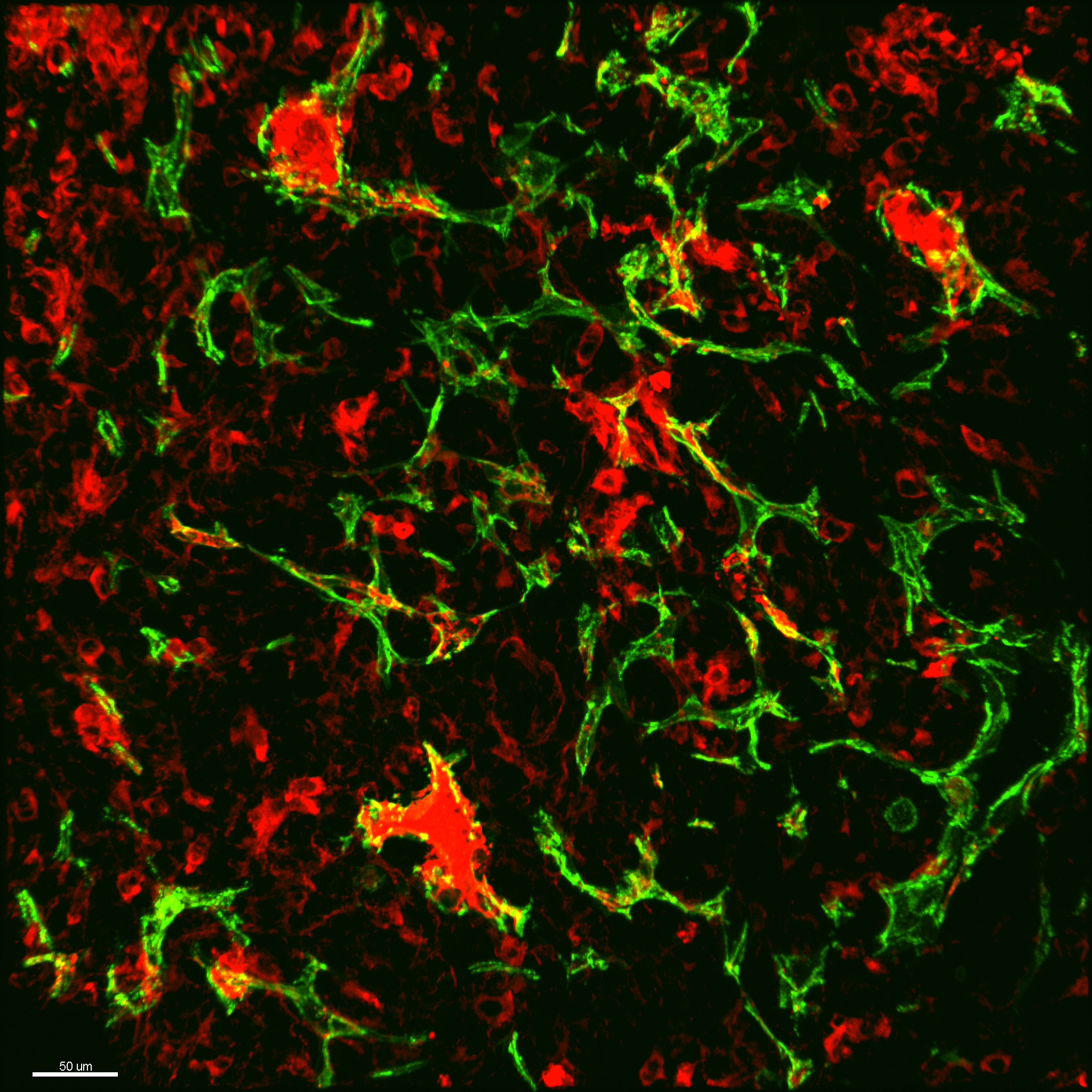

Supplement: Supplementary file 2 — Source data Fig. 1 [file 44318_2024_78_MOESM2_ESM.zip › Figure 1/1G/sGCCtr-3.tif]

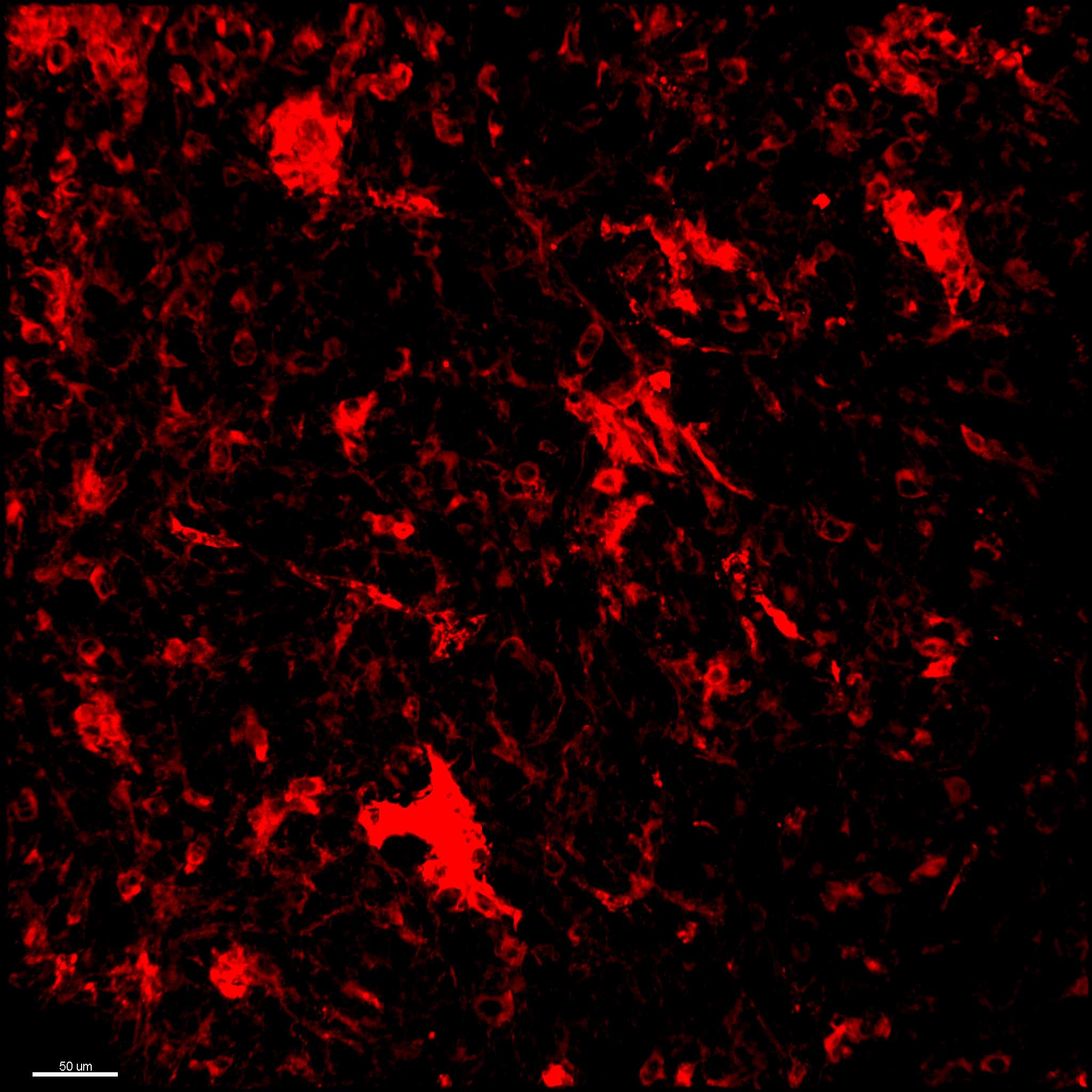

Supplement: Supplementary file 2 — Source data Fig. 1 [file 44318_2024_78_MOESM2_ESM.zip › Figure 1/1G/sGCCtr-2.tif]

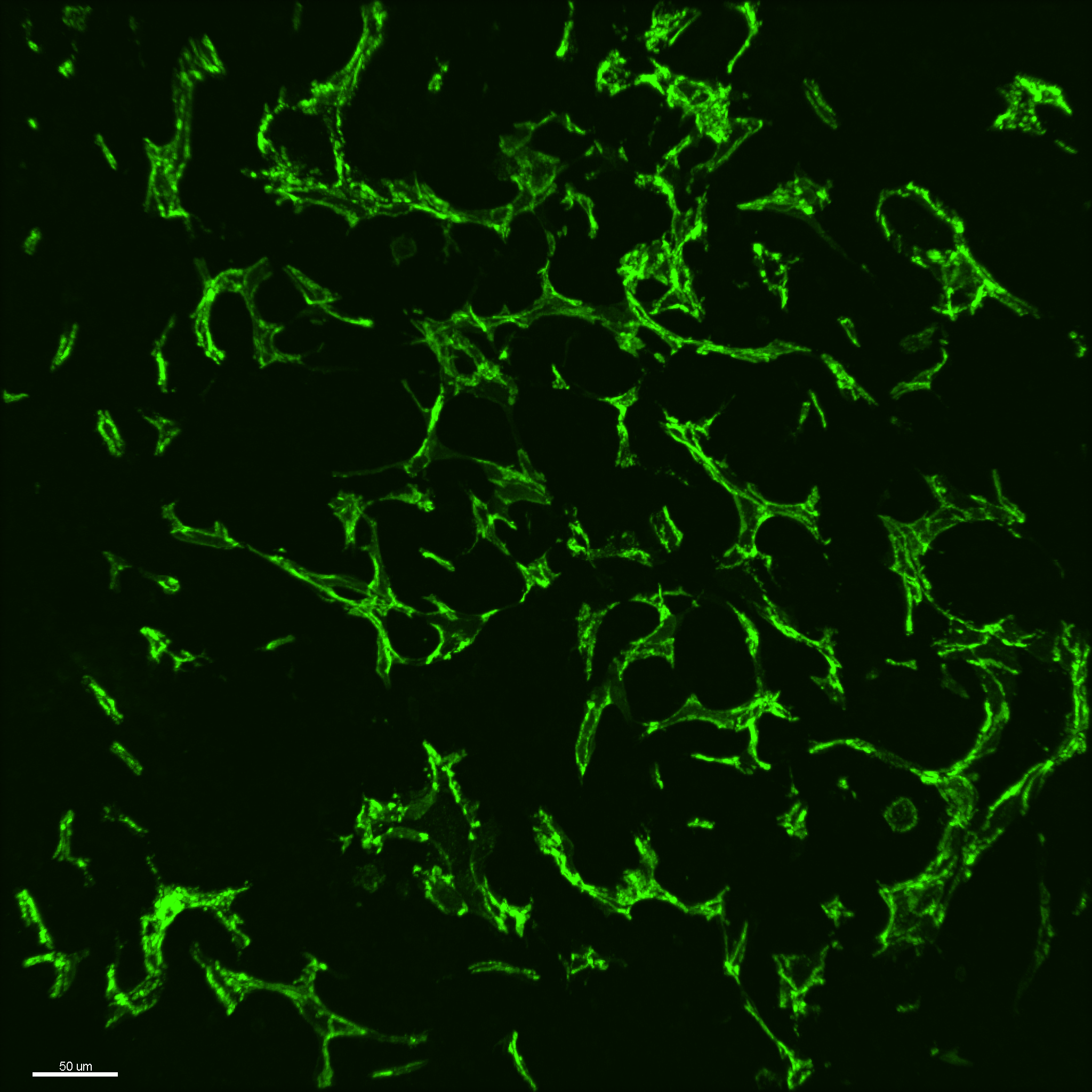

Supplement: Supplementary file 2 — Source data Fig. 1 [file 44318_2024_78_MOESM2_ESM.zip › Figure 1/1G/sGCCtr-1.tif]

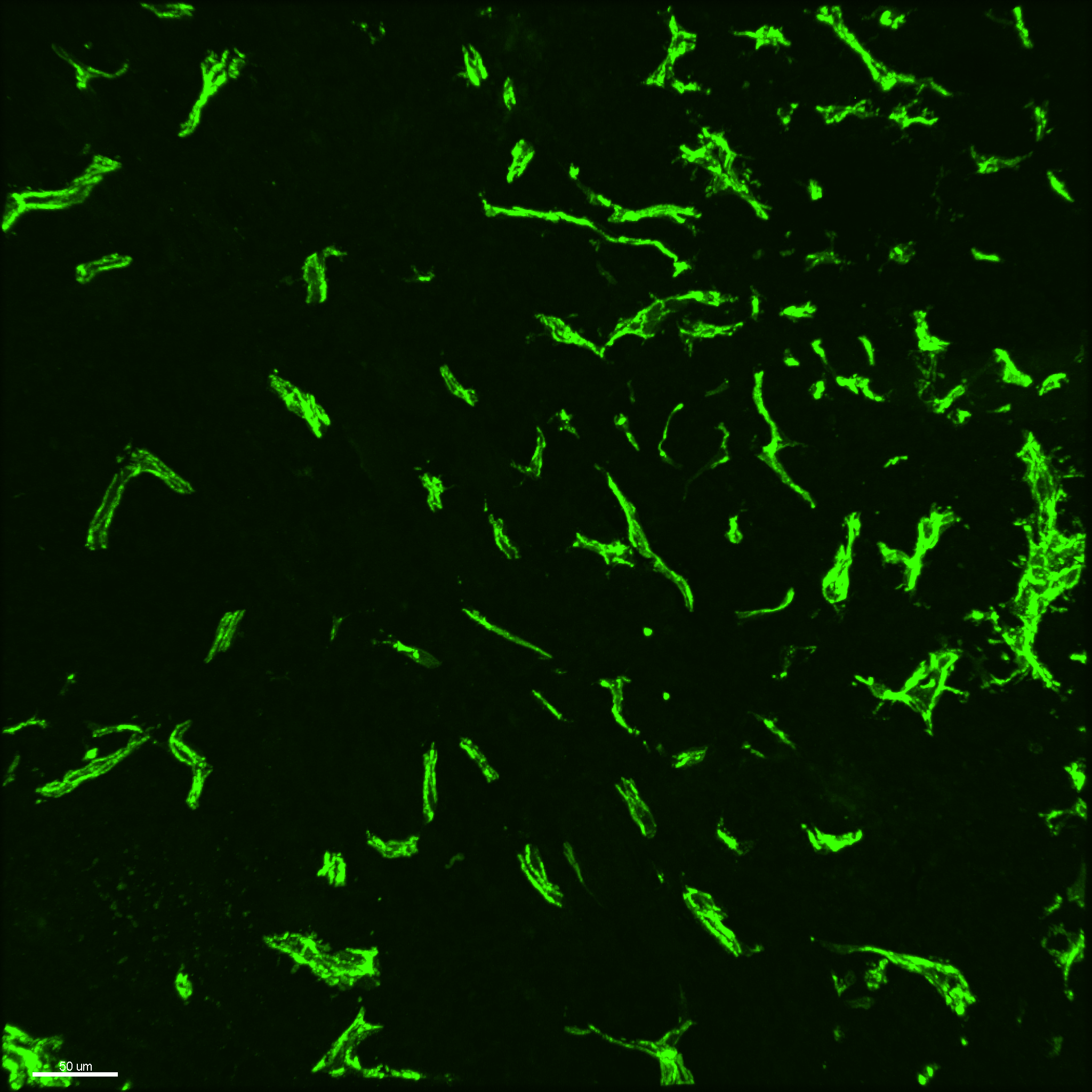

Supplement: Supplementary file 2 — Source data Fig. 1 [file 44318_2024_78_MOESM2_ESM.zip › Figure 1/1G/sGC╬öPC-1.tif]

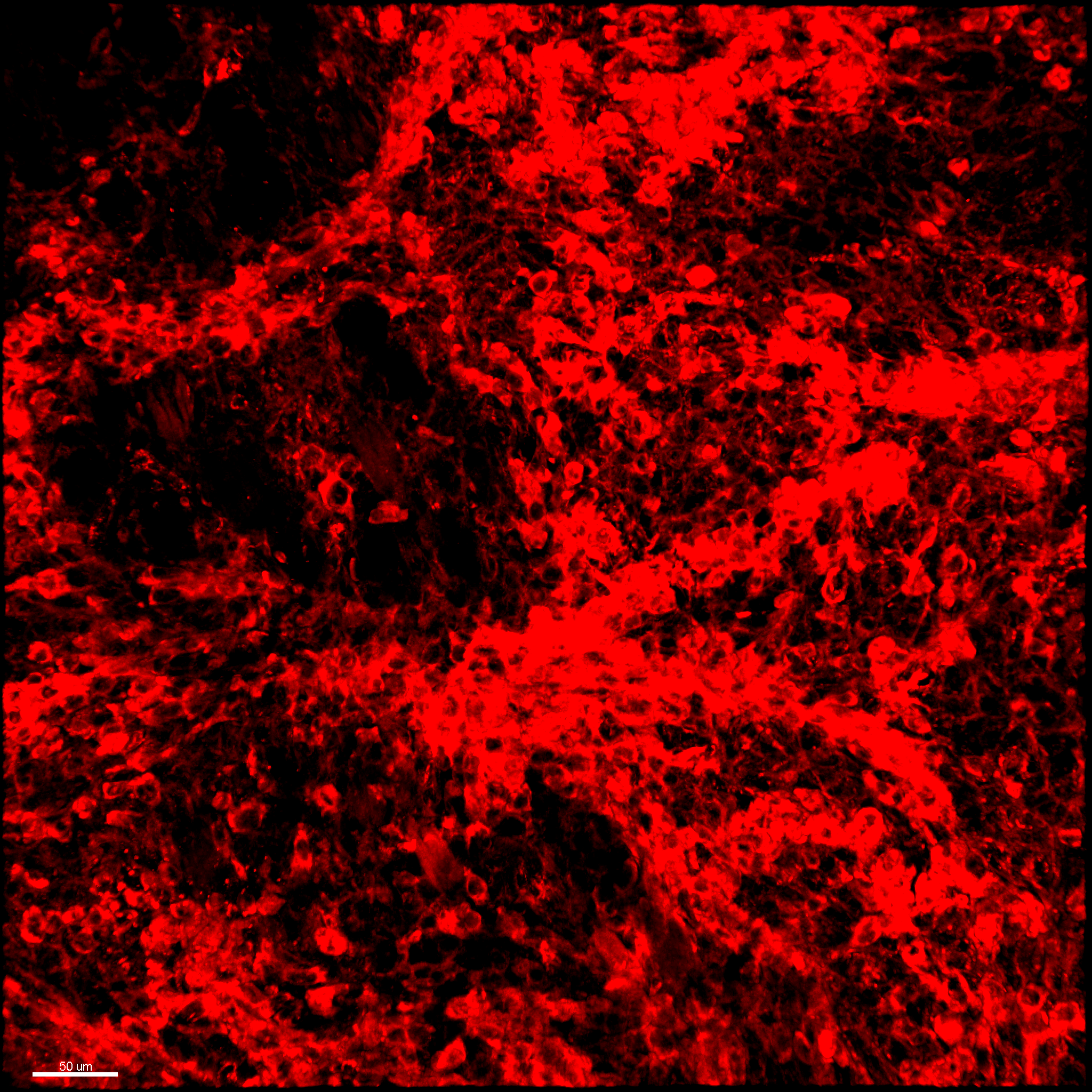

Supplement: Supplementary file 2 — Source data Fig. 1 [file 44318_2024_78_MOESM2_ESM.zip › Figure 1/1G/sGC╬öPC-2.tif]

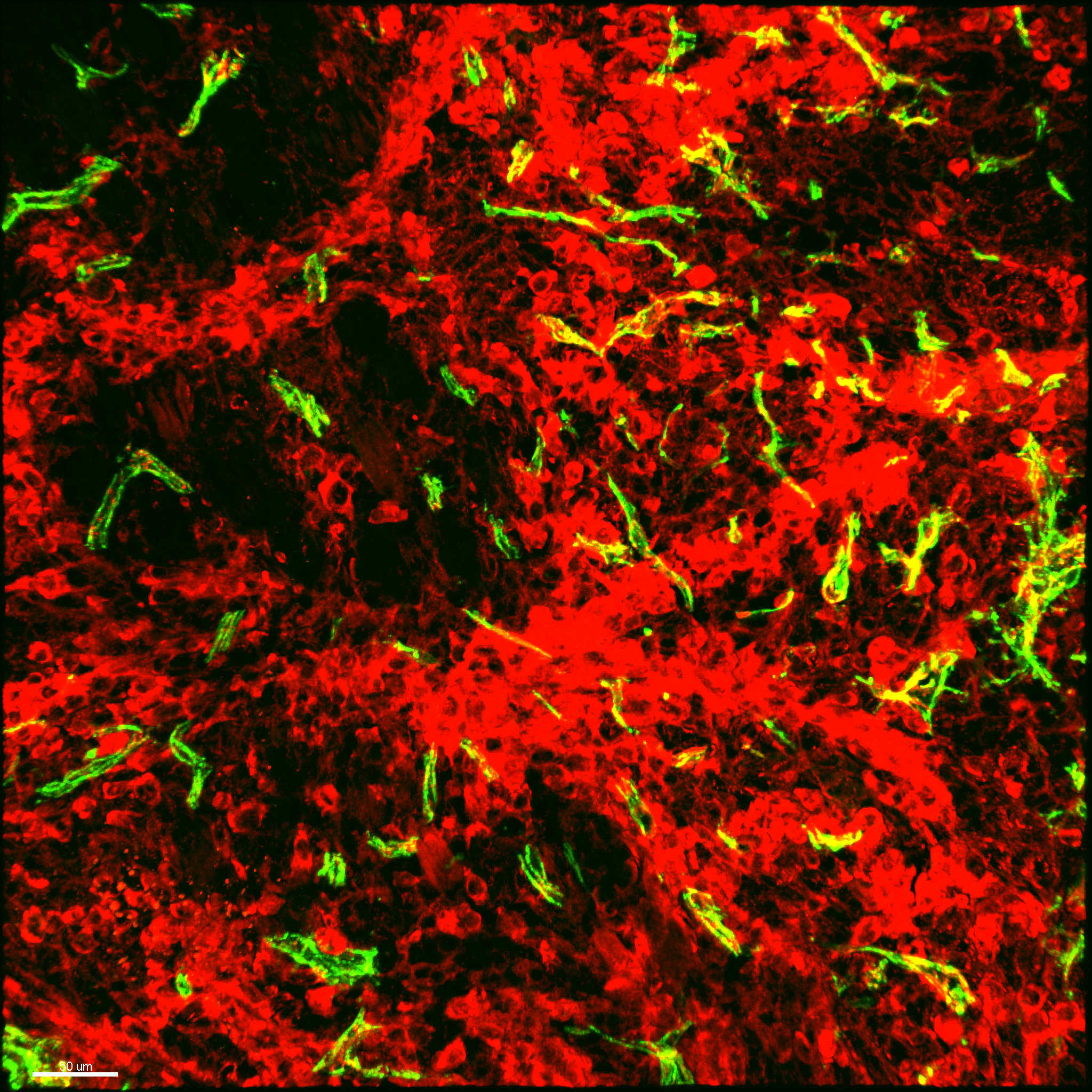

Supplement: Supplementary file 2 — Source data Fig. 1 [file 44318_2024_78_MOESM2_ESM.zip › Figure 1/1G/sGC╬öPC-3.tif]

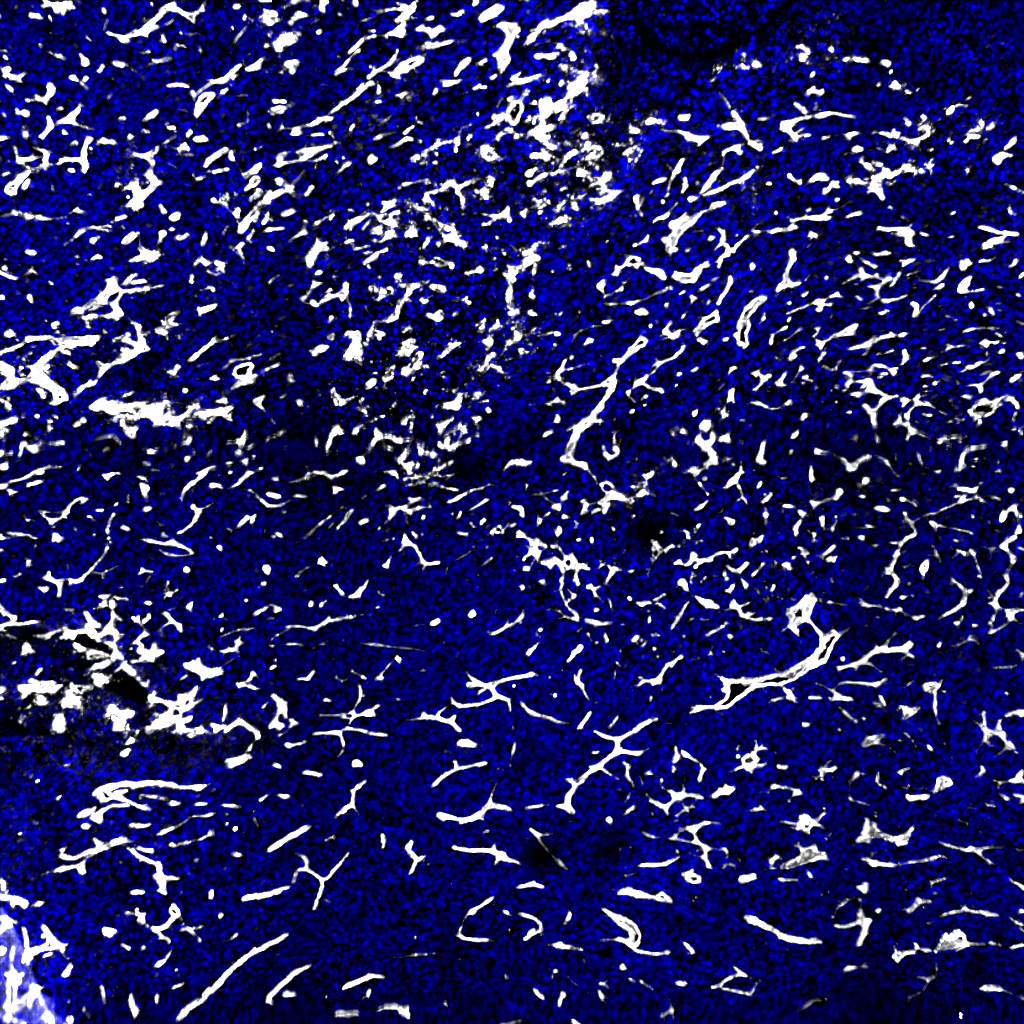

Supplement: Supplementary file 2 — Source data Fig. 1 [file 44318_2024_78_MOESM2_ESM.zip › Figure 1/1E/hypoxyprobe ct1399_405_40um_Zyla_488_561 Zyla 40um_640 Zyla_40um_ZJ Liver 40x water_16.34_005.tif]

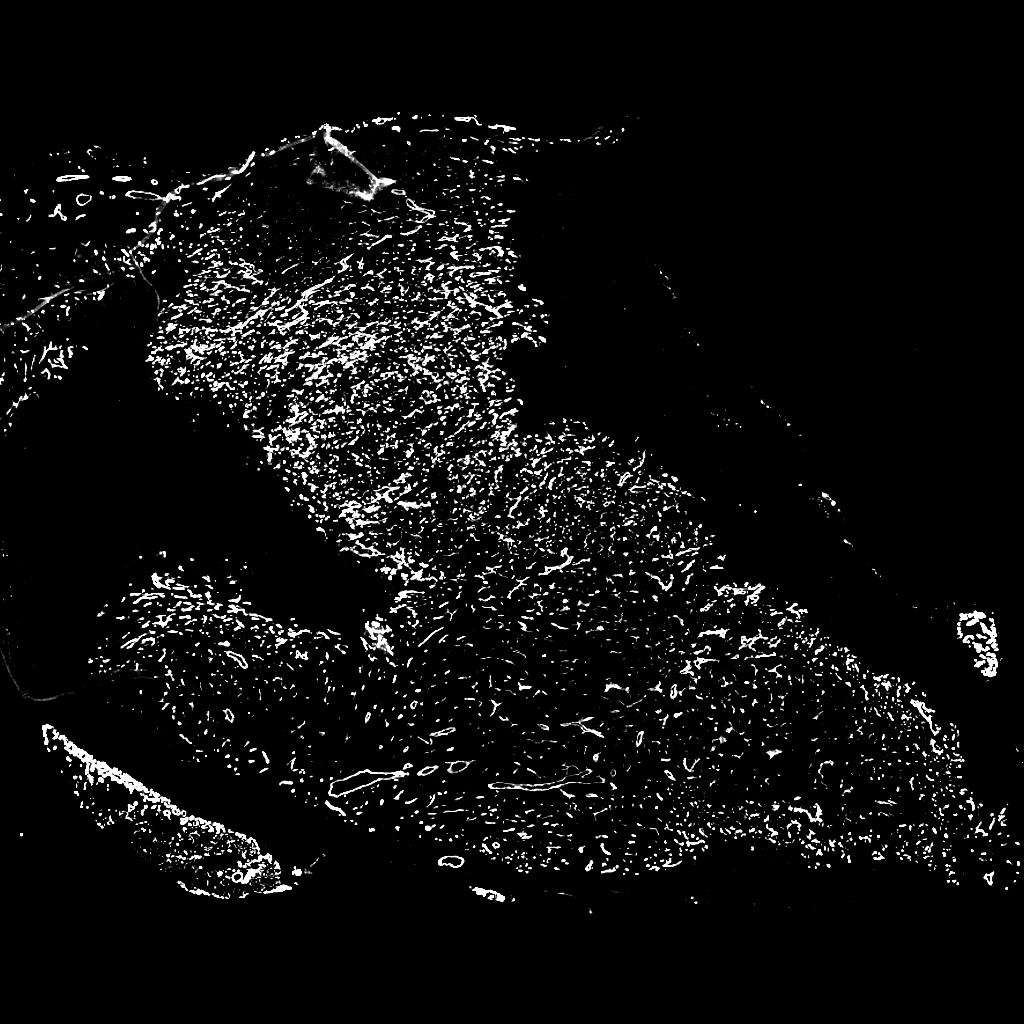

Supplement: Supplementary file 2 — Source data Fig. 1 [file 44318_2024_78_MOESM2_ESM.zip › Figure 1/1E/hypoxyprobe ct1399_405_40um_Zyla_488_561 Zyla 40um_640 Zyla_40um_ZJ Liver 40x water_16.34_002.tif]

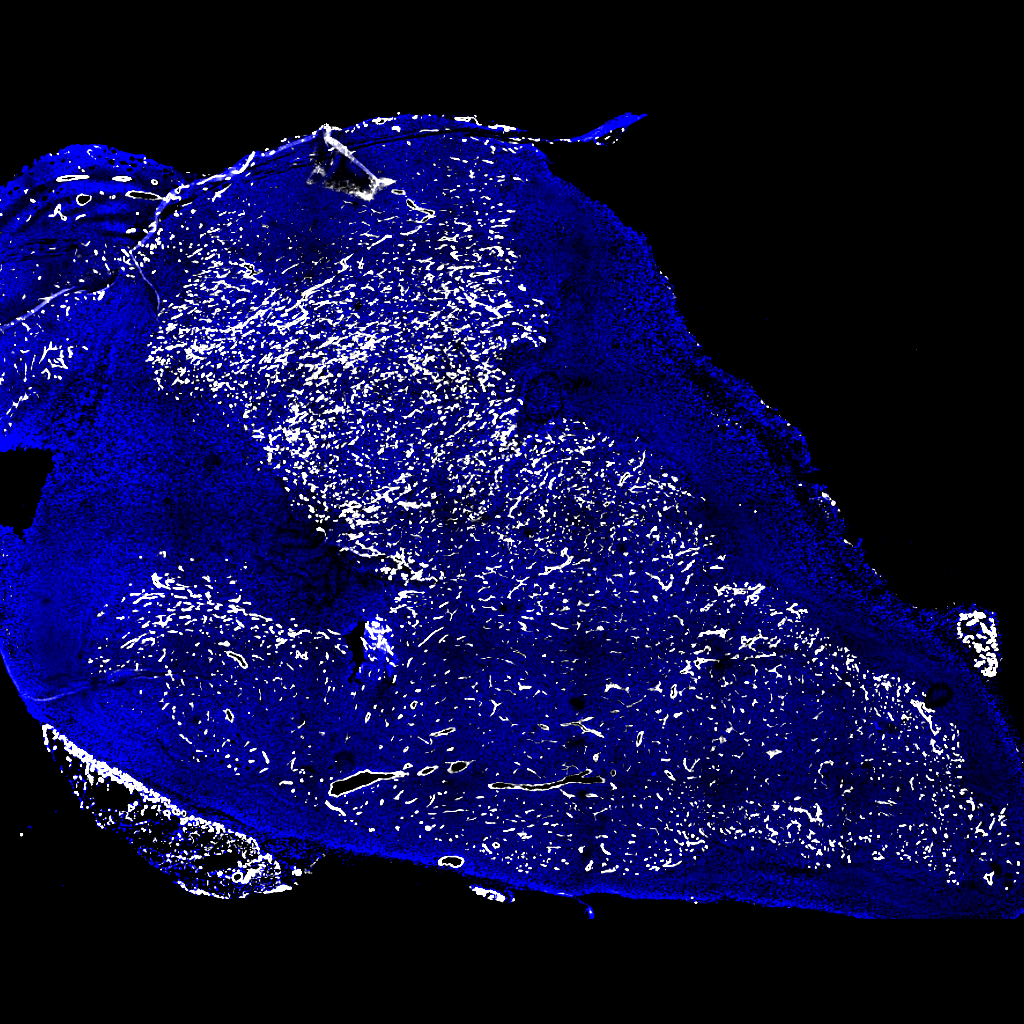

Supplement: Supplementary file 2 — Source data Fig. 1 [file 44318_2024_78_MOESM2_ESM.zip › Figure 1/1E/hypoxyprobe ct1399_405_40um_Zyla_488_561 Zyla 40um_640 Zyla_40um_ZJ Liver 40x water_16.34_003.tif]

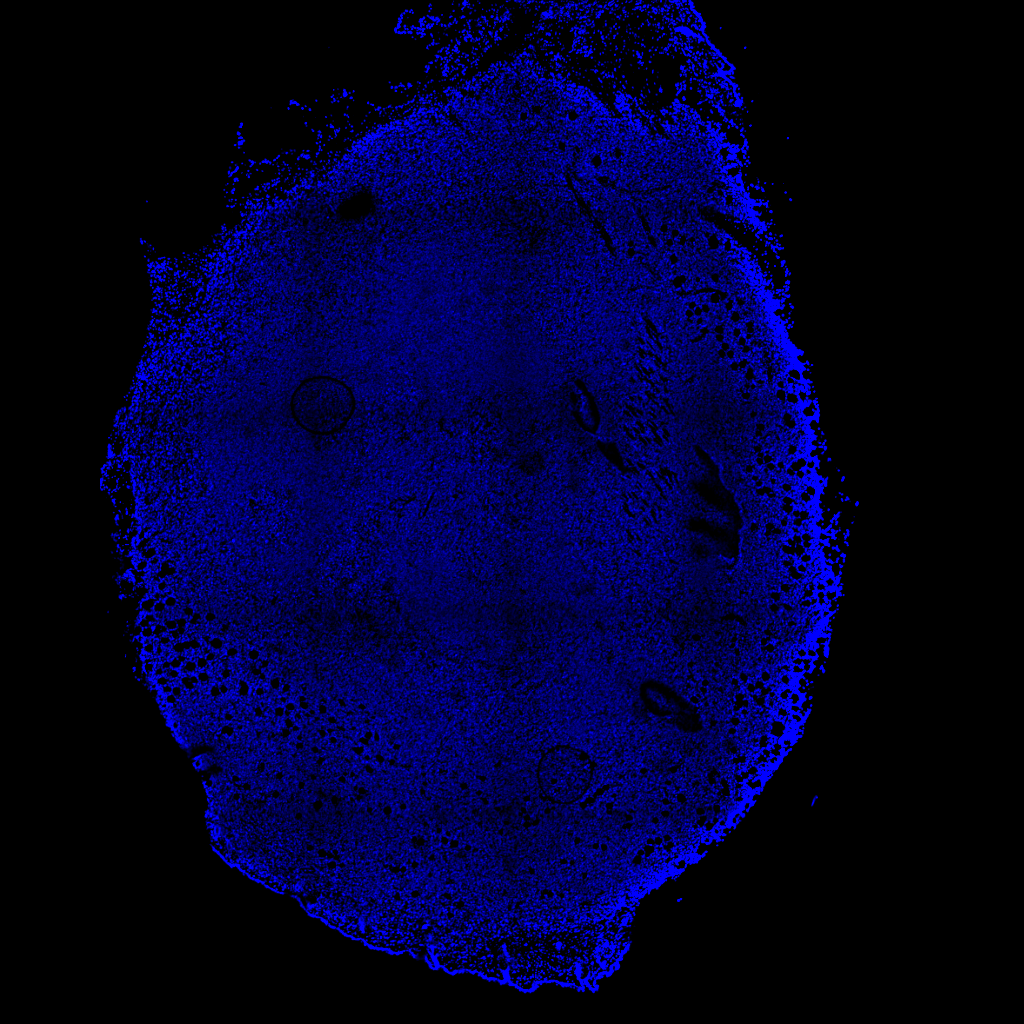

Supplement: Supplementary file 2 — Source data Fig. 1 [file 44318_2024_78_MOESM2_ESM.zip › Figure 1/1E/hypoxyprobe ko1381-1_405_40um_Zyla_488_561 Zyla 40um_640 Zyla_40um_ZJ Liver 40x water_15.45_001.tif]

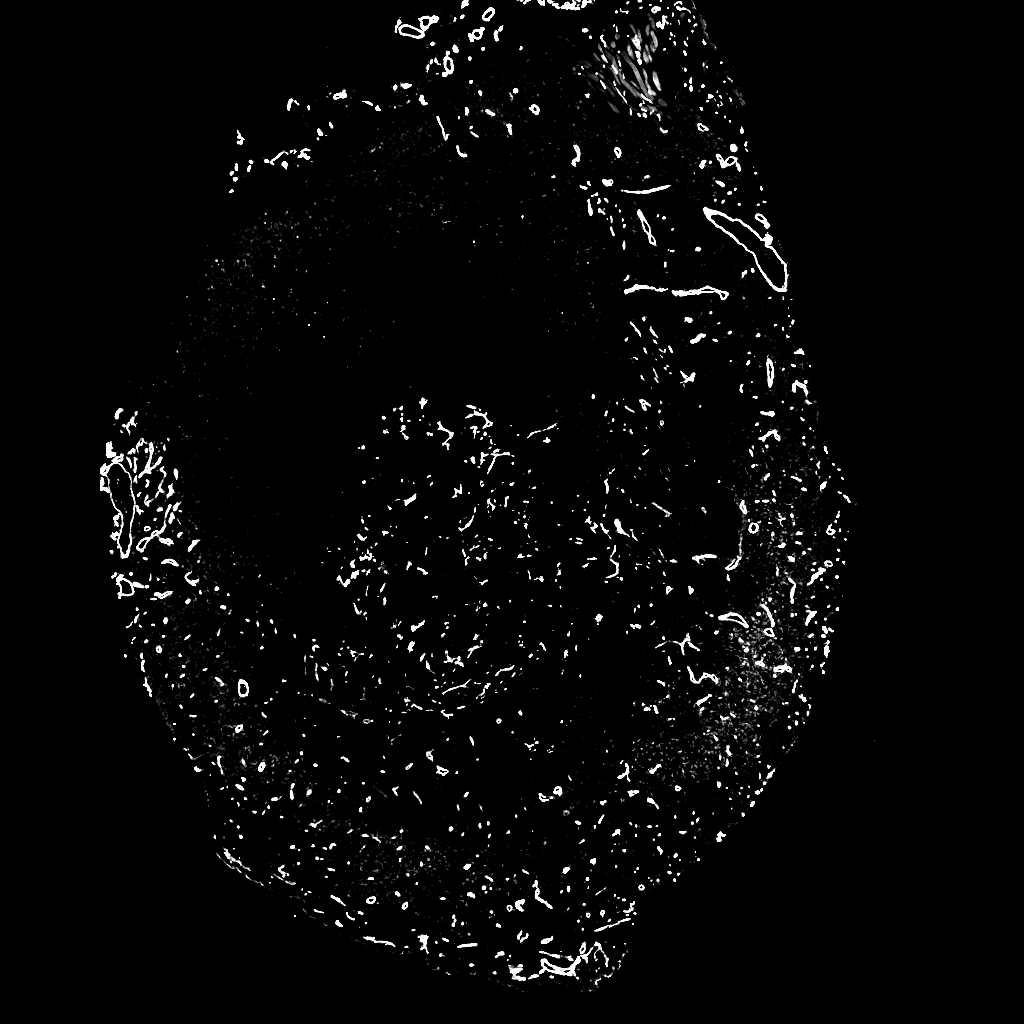

Supplement: Supplementary file 2 — Source data Fig. 1 [file 44318_2024_78_MOESM2_ESM.zip › Figure 1/1E/hypoxyprobe ko1381-1_405_40um_Zyla_488_561 Zyla 40um_640 Zyla_40um_ZJ Liver 40x water_15.45_002.tif]

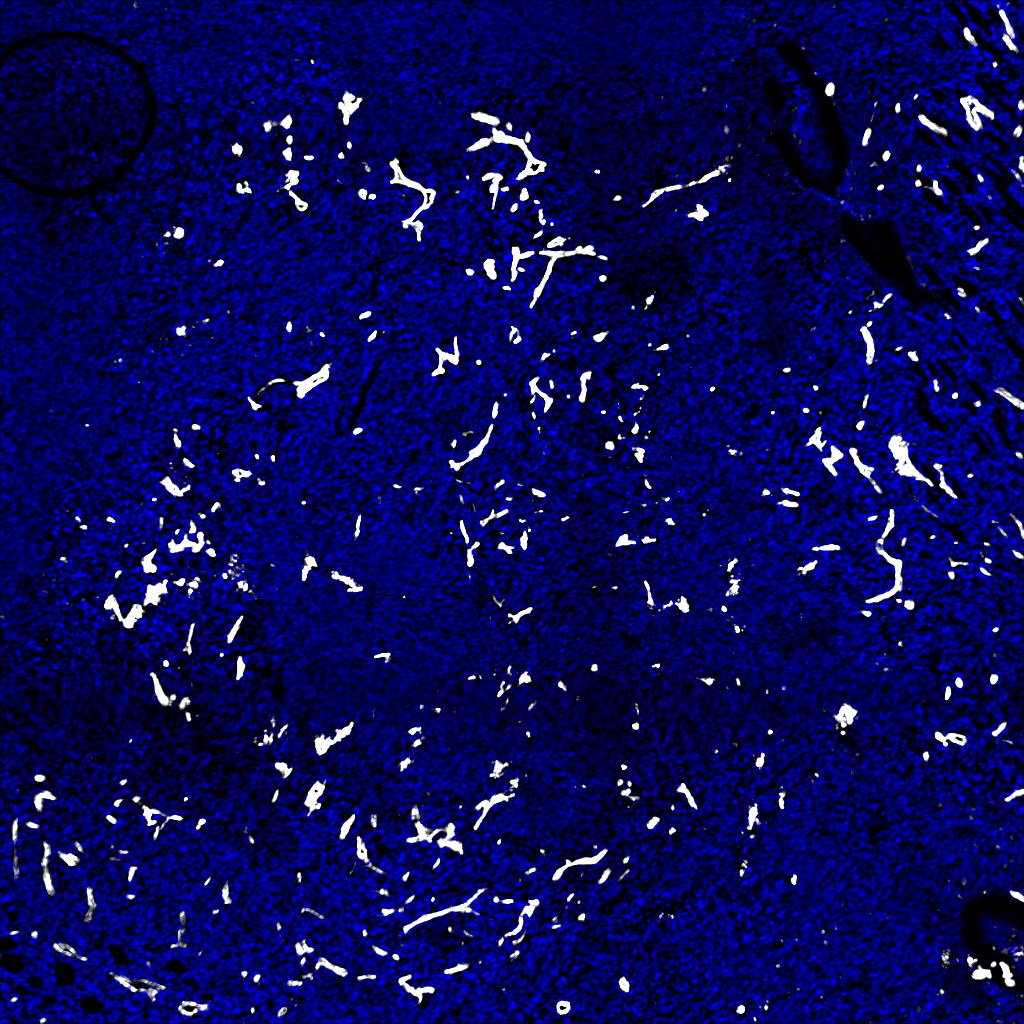

Supplement: Supplementary file 2 — Source data Fig. 1 [file 44318_2024_78_MOESM2_ESM.zip › Figure 1/1E/hypoxyprobe ko1381-1_405_40um_Zyla_488_561 Zyla 40um_640 Zyla_40um_ZJ Liver 40x water_15.45_006.tif]

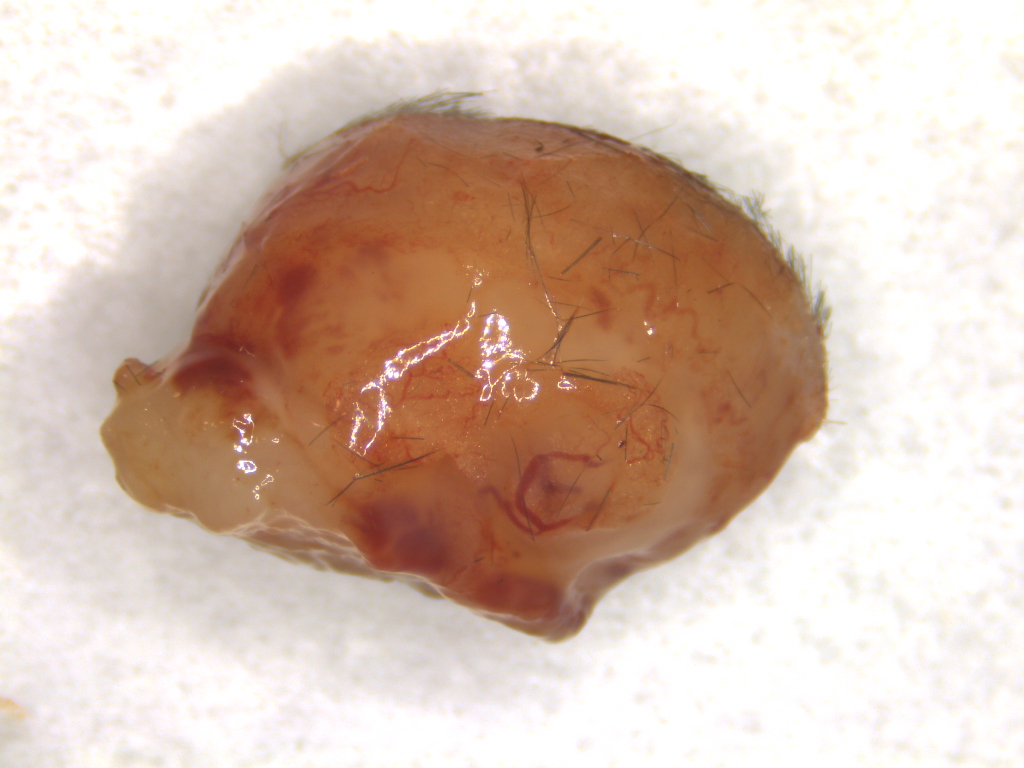

Supplement: Supplementary file 2 — Source data Fig. 1 [file 44318_2024_78_MOESM2_ESM.zip › Figure 1/1C/sGCCtr-5.tif]

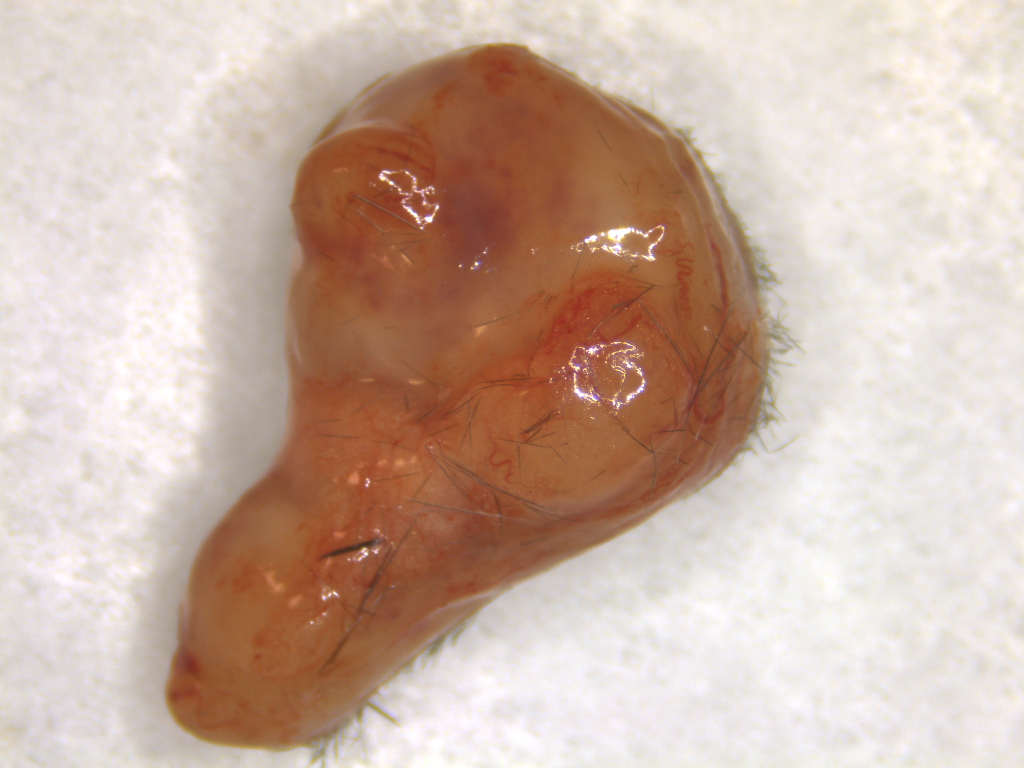

Supplement: Supplementary file 2 — Source data Fig. 1 [file 44318_2024_78_MOESM2_ESM.zip › Figure 1/1C/sGCCtr-4.tif]

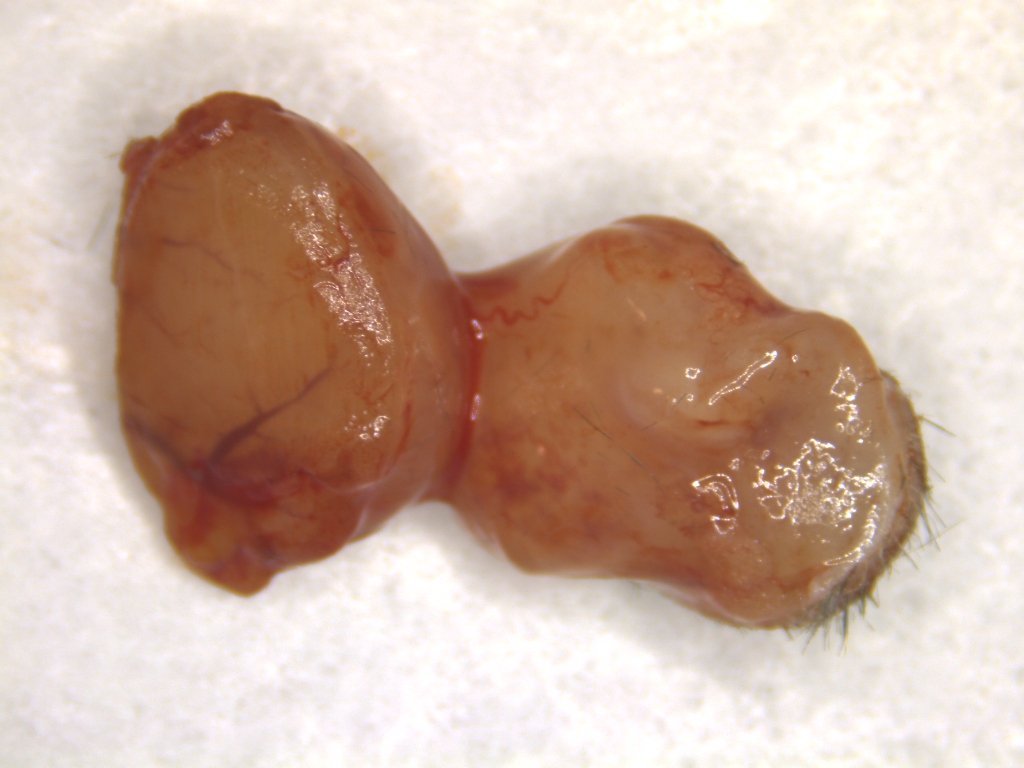

Supplement: Supplementary file 2 — Source data Fig. 1 [file 44318_2024_78_MOESM2_ESM.zip › Figure 1/1C/sGCCtr-6.tif]

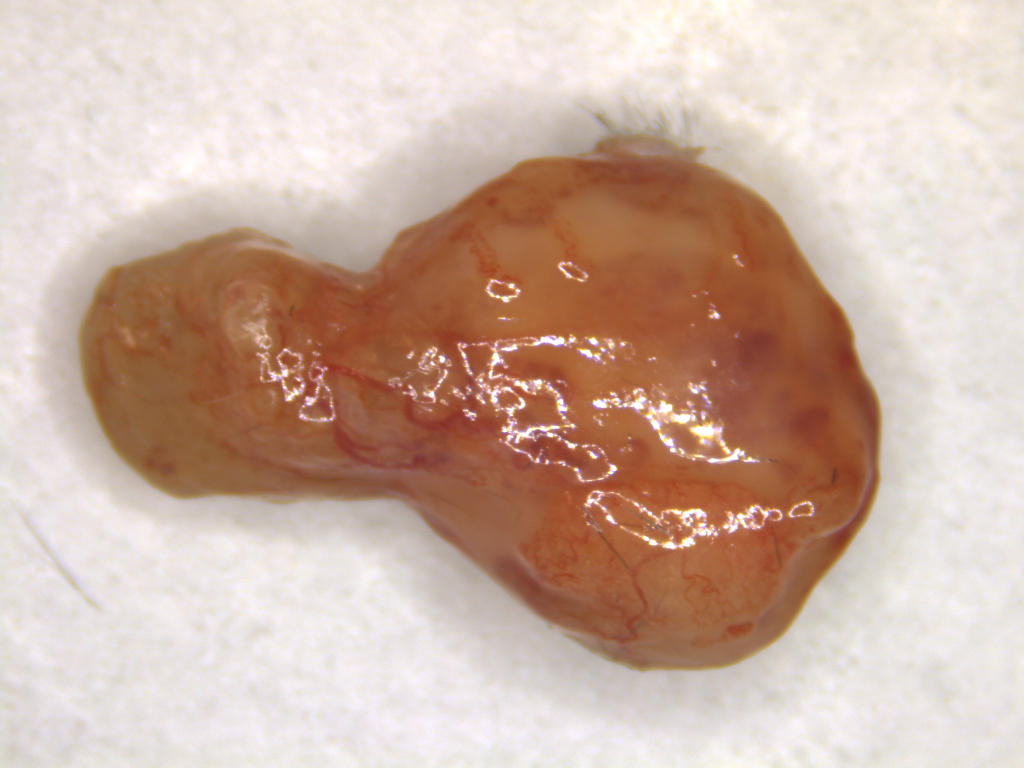

Supplement: Supplementary file 2 — Source data Fig. 1 [file 44318_2024_78_MOESM2_ESM.zip › Figure 1/1C/sGCCtr-7.tif]

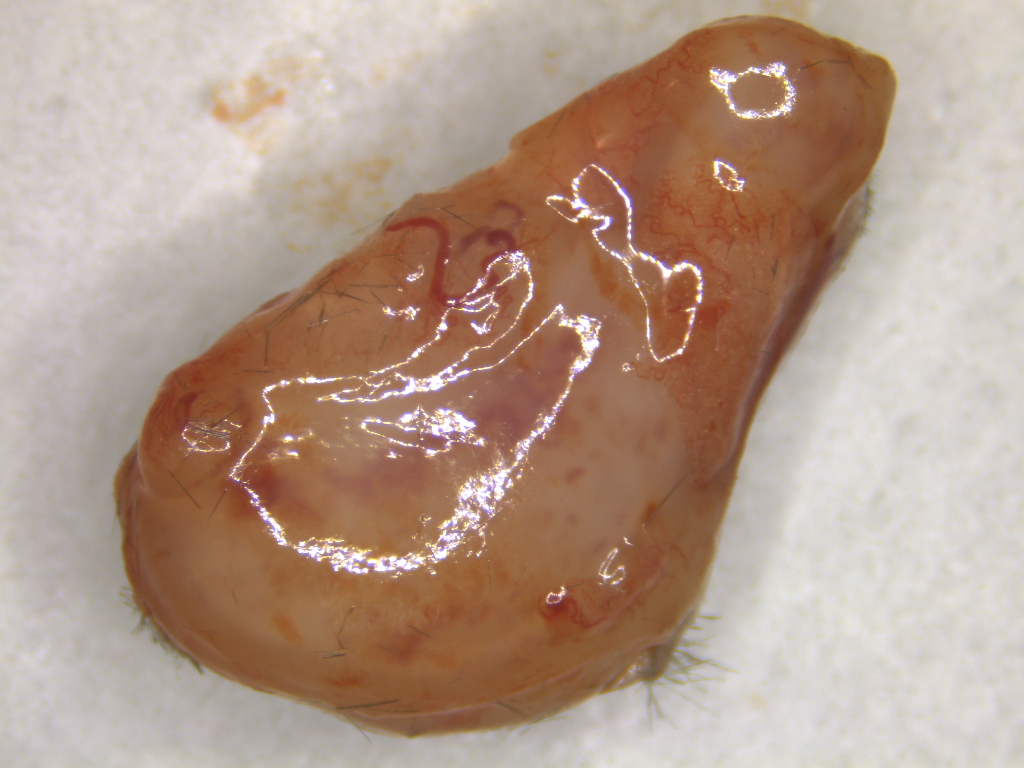

Supplement: Supplementary file 2 — Source data Fig. 1 [file 44318_2024_78_MOESM2_ESM.zip › Figure 1/1C/sGCCtr-3.tif]

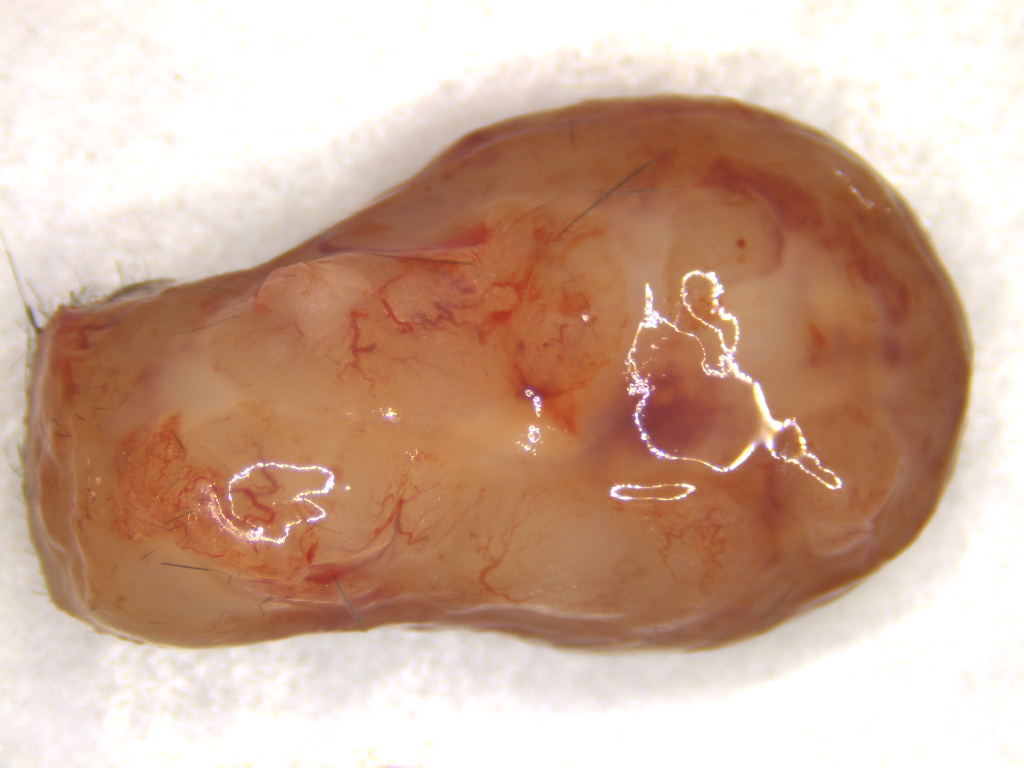

Supplement: Supplementary file 2 — Source data Fig. 1 [file 44318_2024_78_MOESM2_ESM.zip › Figure 1/1C/sGCCtr-2.tif]

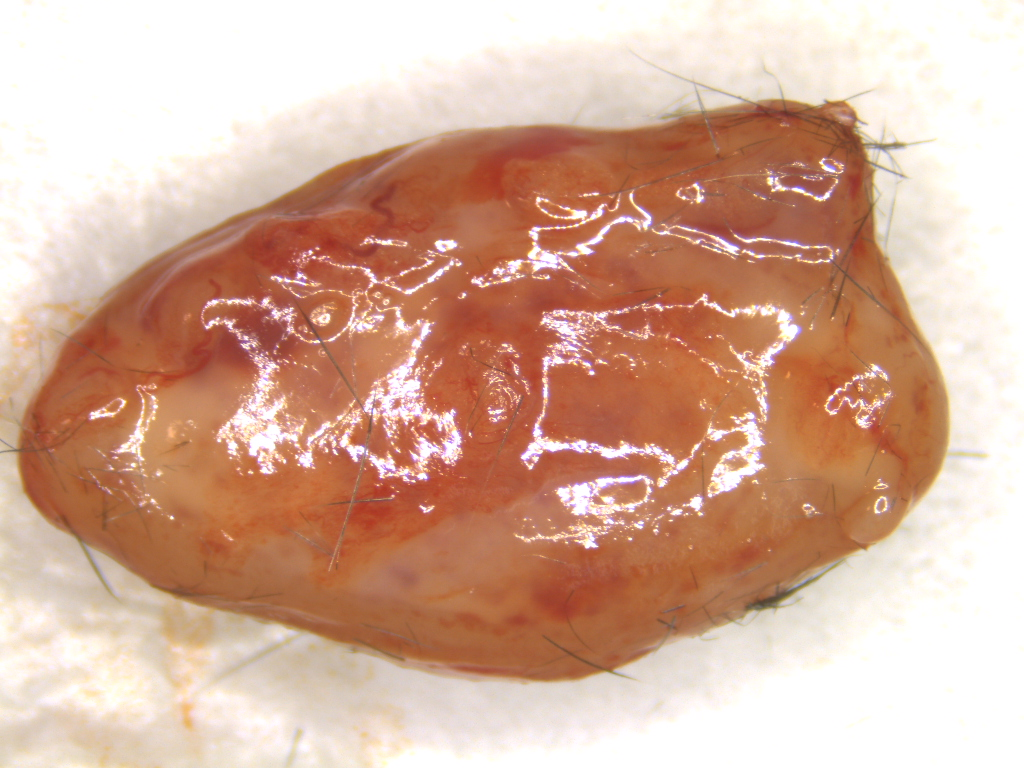

Supplement: Supplementary file 2 — Source data Fig. 1 [file 44318_2024_78_MOESM2_ESM.zip › Figure 1/1C/sGCCtr-1.tif]

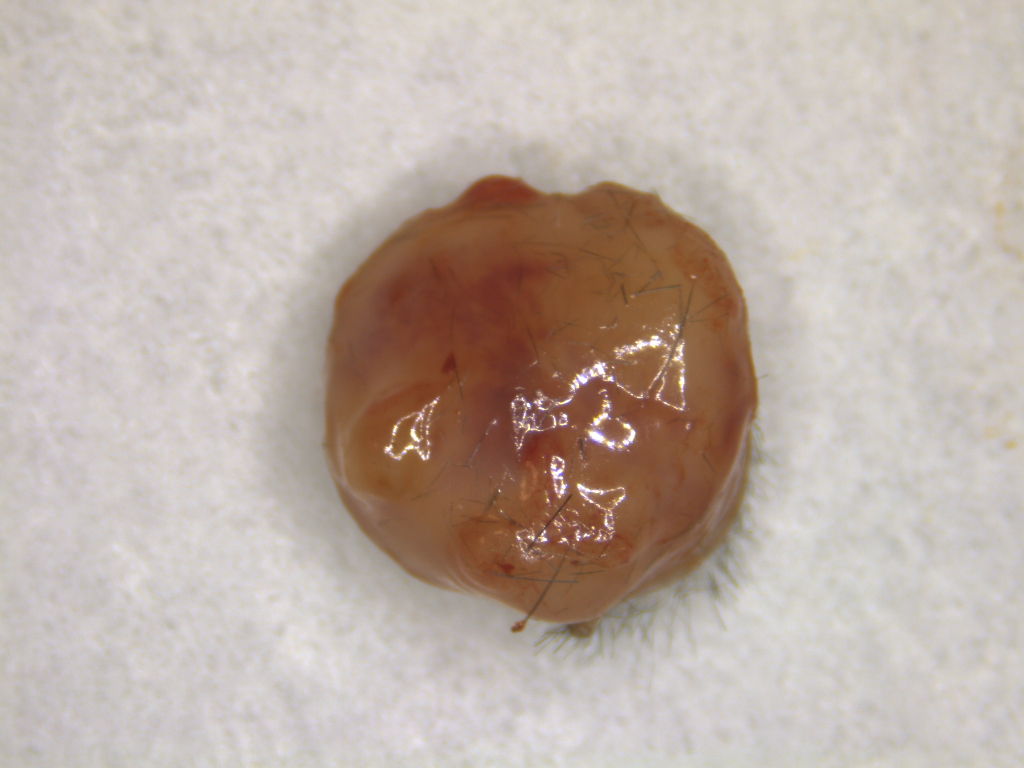

Supplement: Supplementary file 2 — Source data Fig. 1 [file 44318_2024_78_MOESM2_ESM.zip › Figure 1/1C/sGC╬öPC-7.tif]

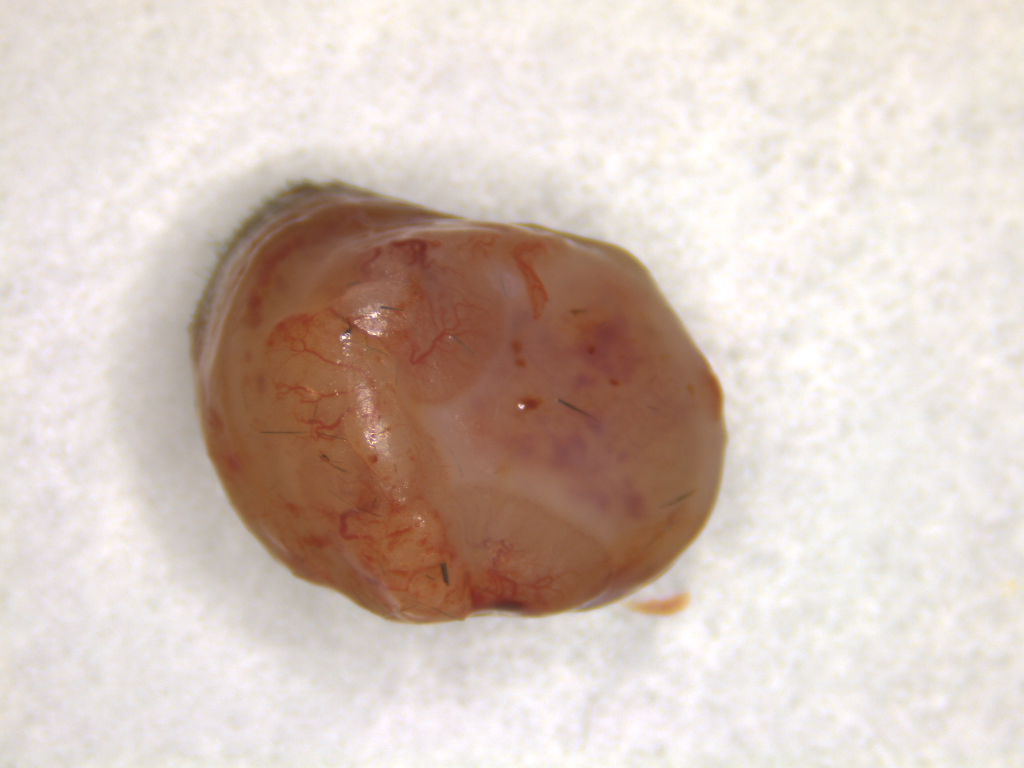

Supplement: Supplementary file 2 — Source data Fig. 1 [file 44318_2024_78_MOESM2_ESM.zip › Figure 1/1C/sGC╬öPC-6.tif]

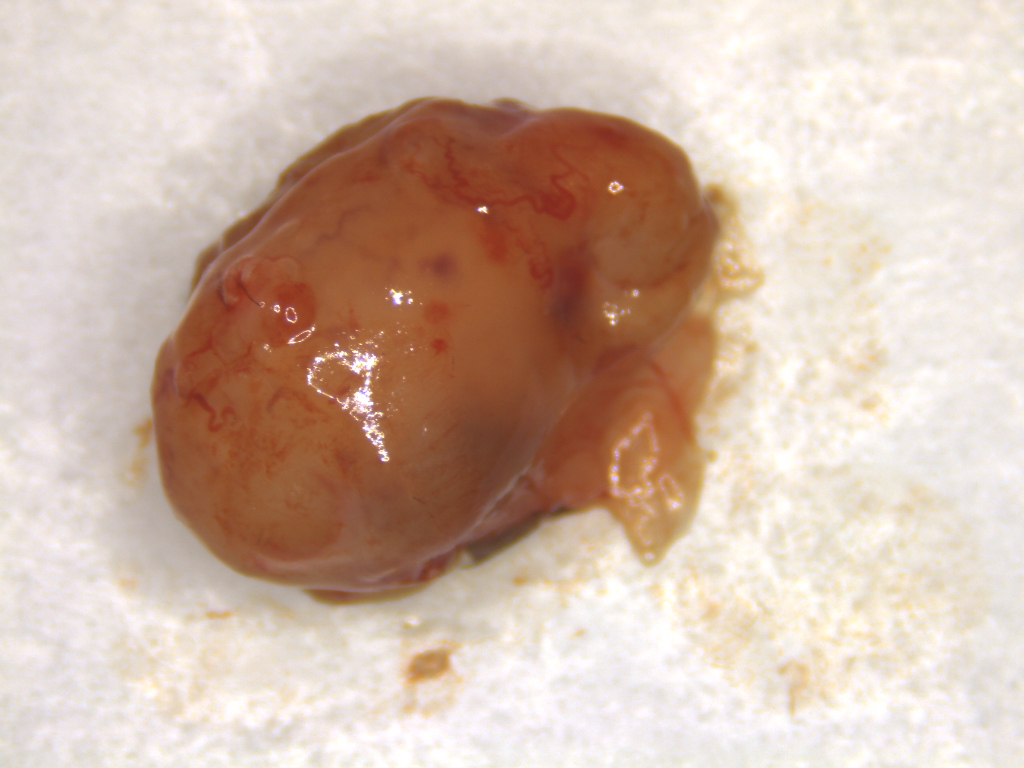

Supplement: Supplementary file 2 — Source data Fig. 1 [file 44318_2024_78_MOESM2_ESM.zip › Figure 1/1C/sGC╬öPC-4.tif]

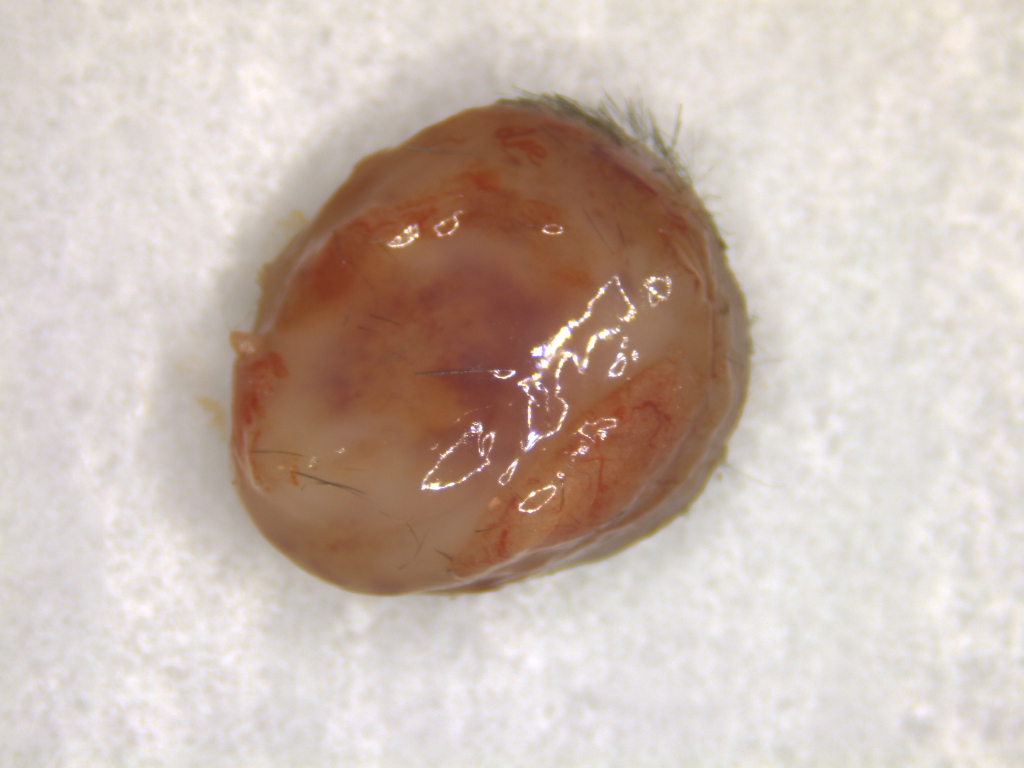

Supplement: Supplementary file 2 — Source data Fig. 1 [file 44318_2024_78_MOESM2_ESM.zip › Figure 1/1C/sGC╬öPC-5.tif]

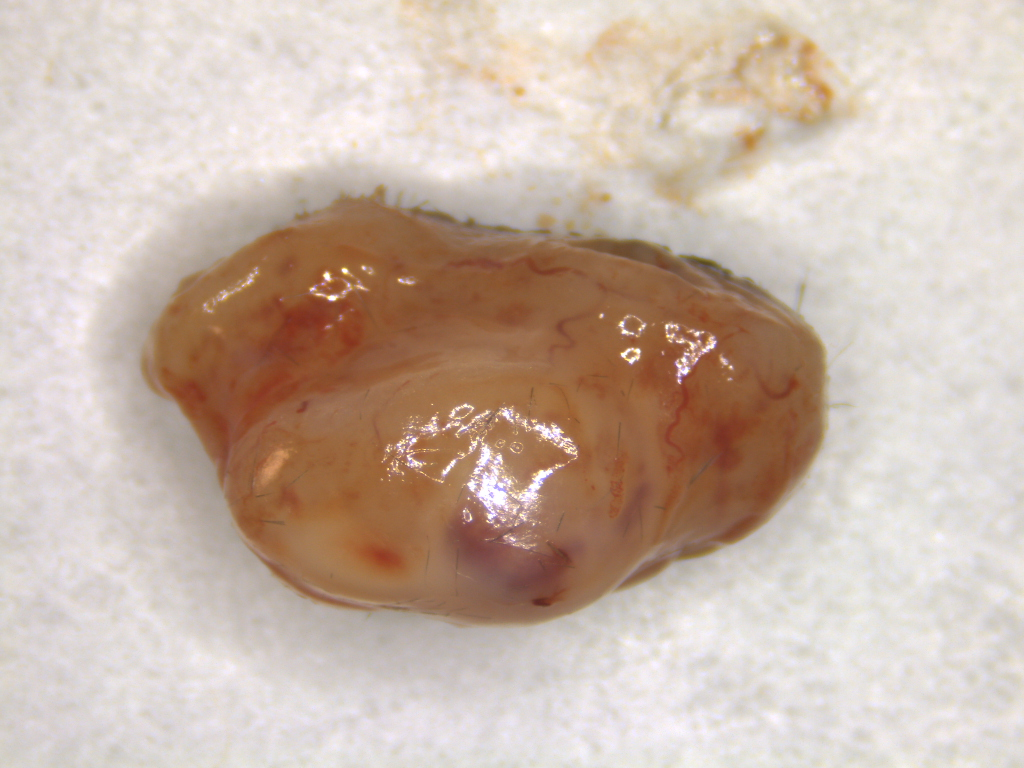

Supplement: Supplementary file 2 — Source data Fig. 1 [file 44318_2024_78_MOESM2_ESM.zip › Figure 1/1C/sGC╬öPC-1.tif]

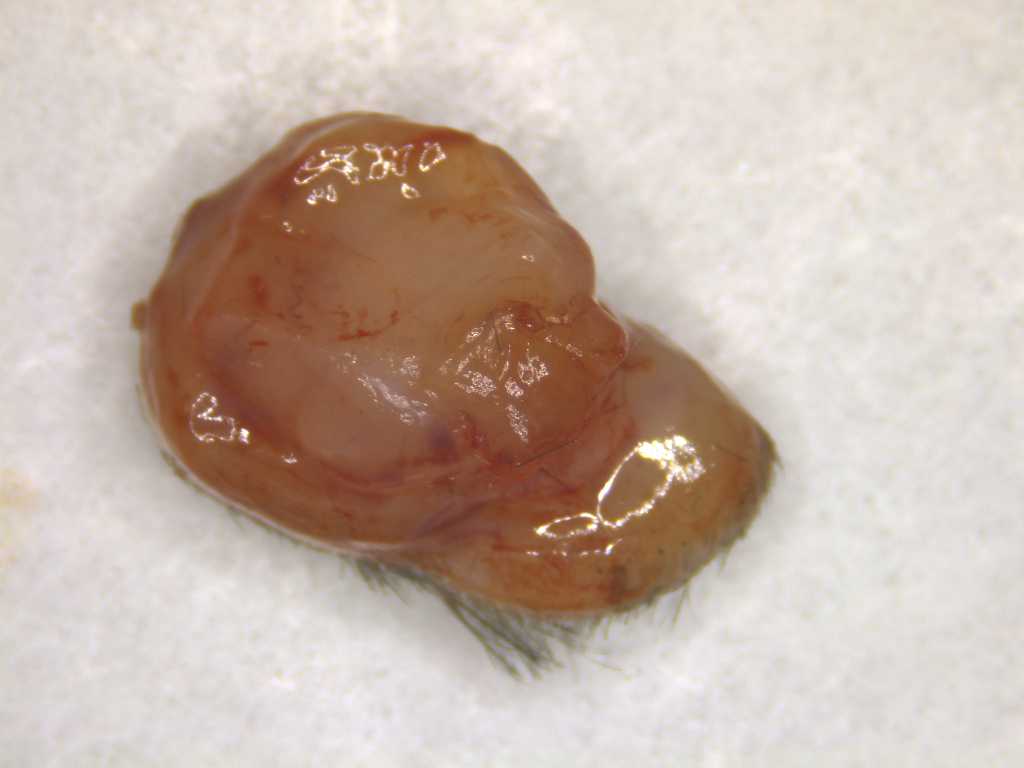

Supplement: Supplementary file 2 — Source data Fig. 1 [file 44318_2024_78_MOESM2_ESM.zip › Figure 1/1C/sGC╬öPC-2.tif]

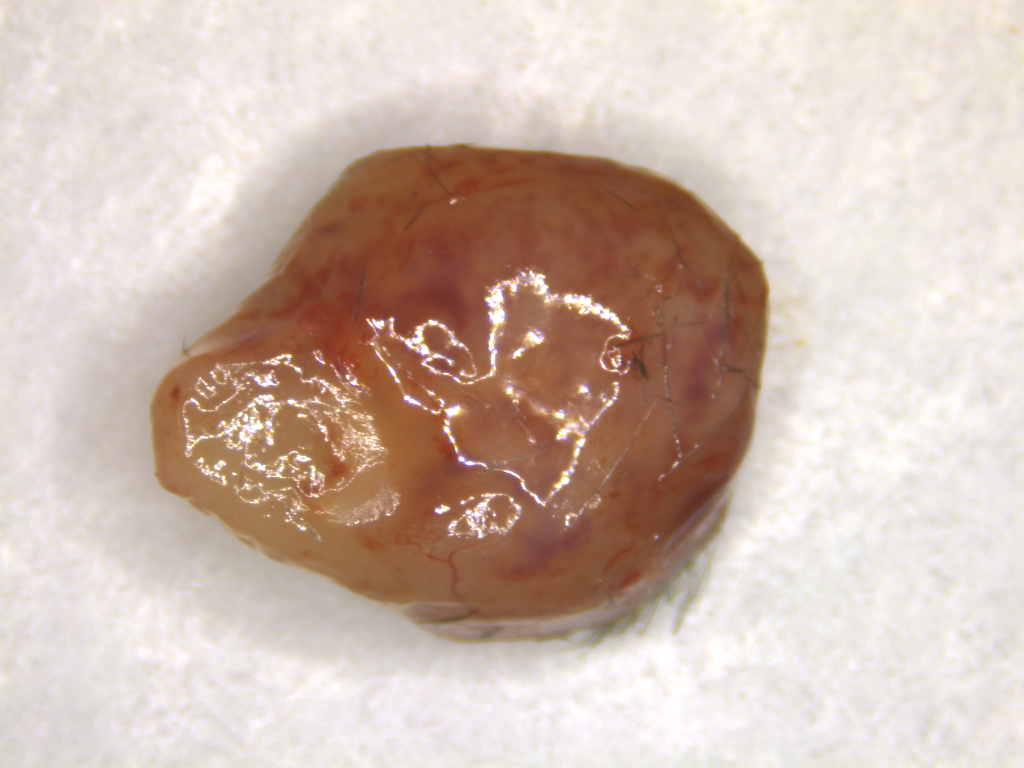

Supplement: Supplementary file 2 — Source data Fig. 1 [file 44318_2024_78_MOESM2_ESM.zip › Figure 1/1C/sGC╬öPC-3.tif]

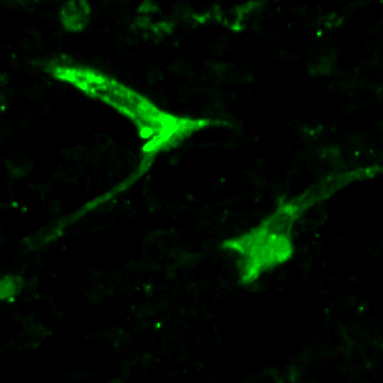

Supplement: Supplementary file 3 — Source data Fig. 3 [file 44318_2024_78_MOESM3_ESM.zip › Figure 3/3K/sGCCtr-3.tif]

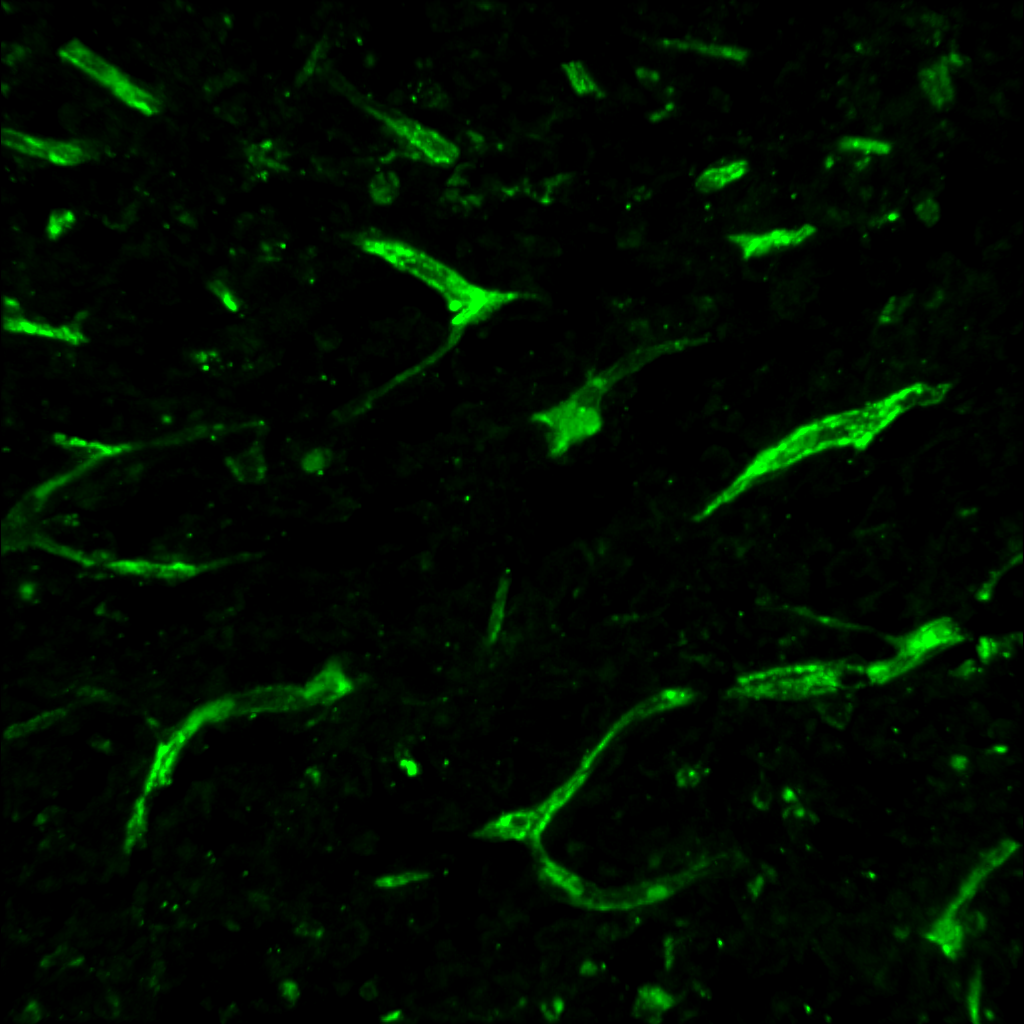

Supplement: Supplementary file 3 — Source data Fig. 3 [file 44318_2024_78_MOESM3_ESM.zip › Figure 3/3K/sGCCtr-2.tif]

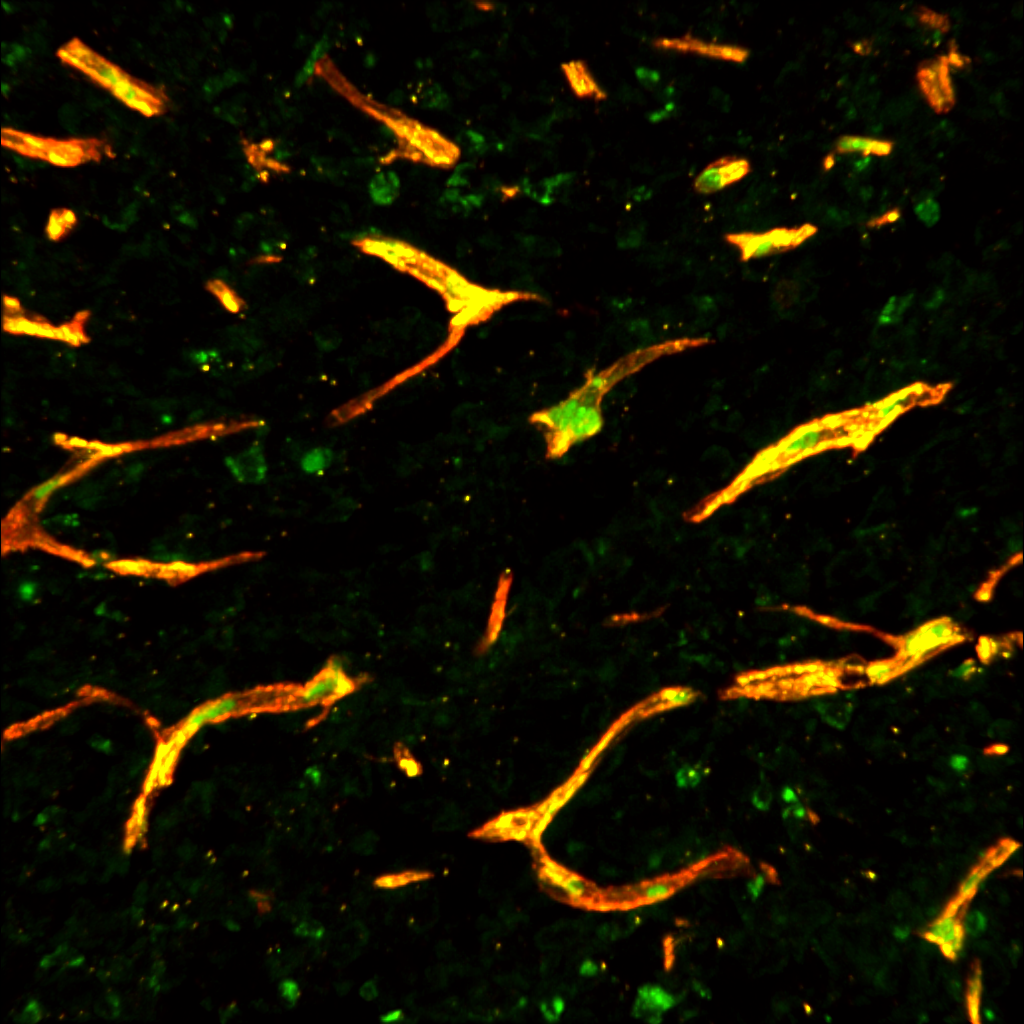

Supplement: Supplementary file 3 — Source data Fig. 3 [file 44318_2024_78_MOESM3_ESM.zip › Figure 3/3K/sGCCtr-1.tif]

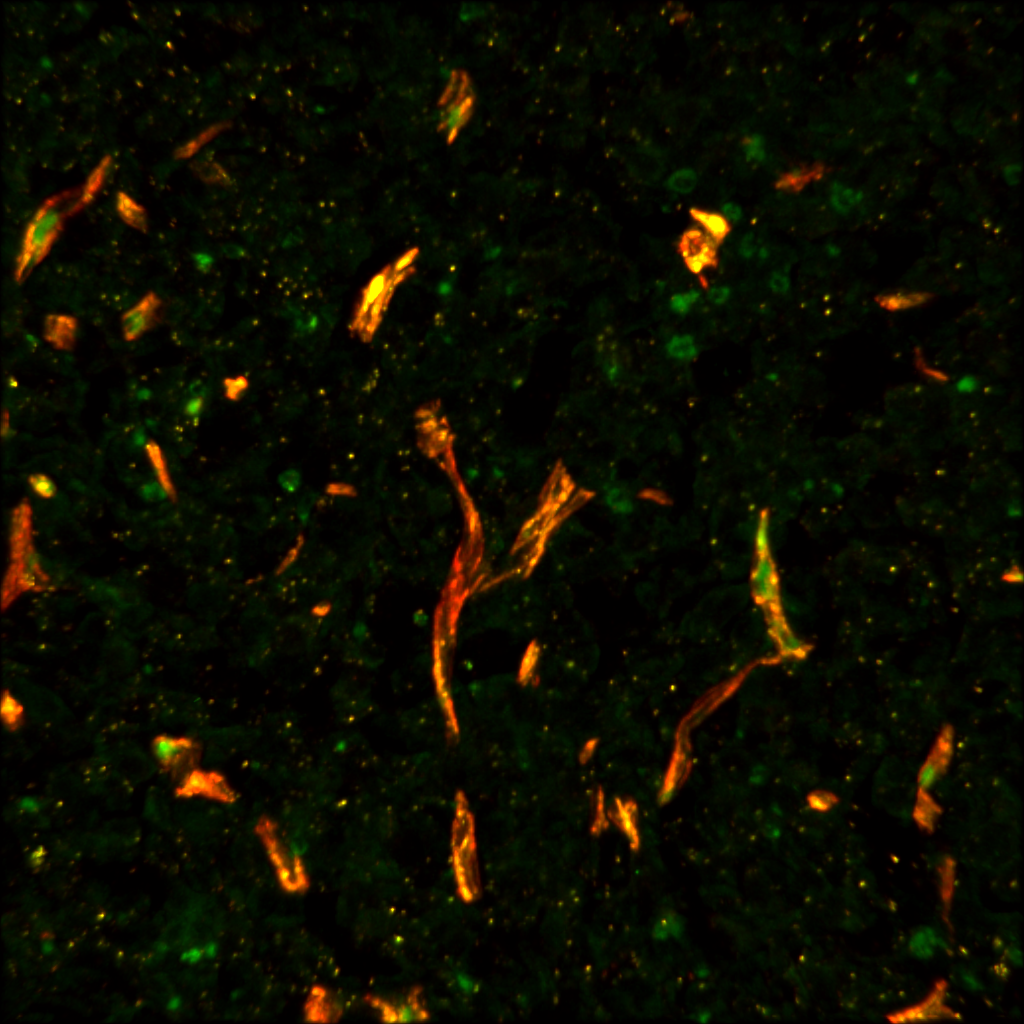

Supplement: Supplementary file 3 — Source data Fig. 3 [file 44318_2024_78_MOESM3_ESM.zip › Figure 3/3K/sGC╬öPC-1.tif]

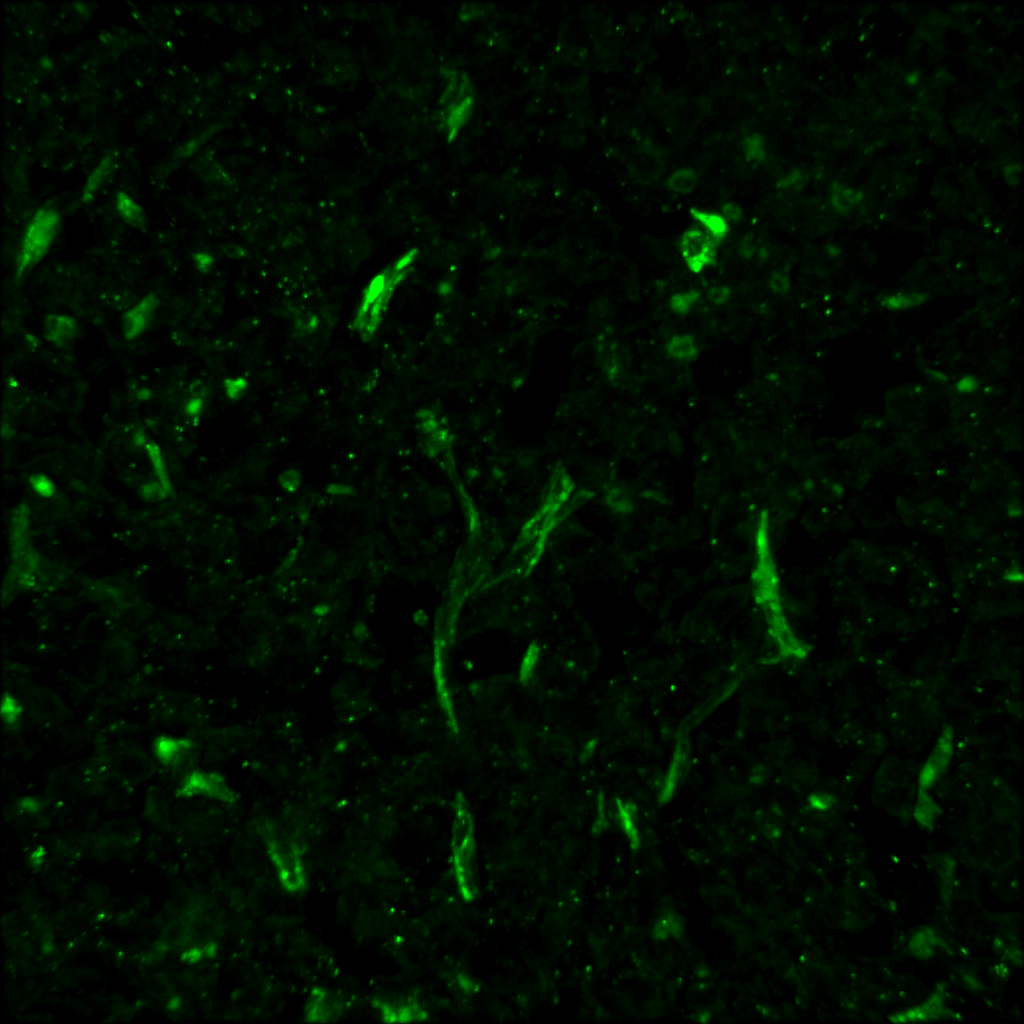

Supplement: Supplementary file 3 — Source data Fig. 3 [file 44318_2024_78_MOESM3_ESM.zip › Figure 3/3K/sGC╬öPC-2.tif]

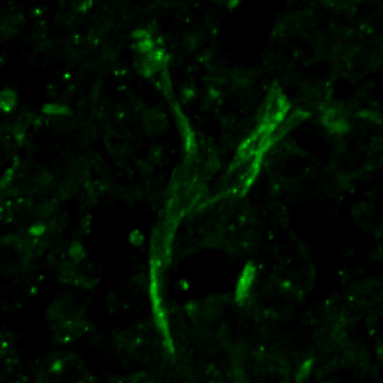

Supplement: Supplementary file 3 — Source data Fig. 3 [file 44318_2024_78_MOESM3_ESM.zip › Figure 3/3K/sGC╬öPC-3.tif]

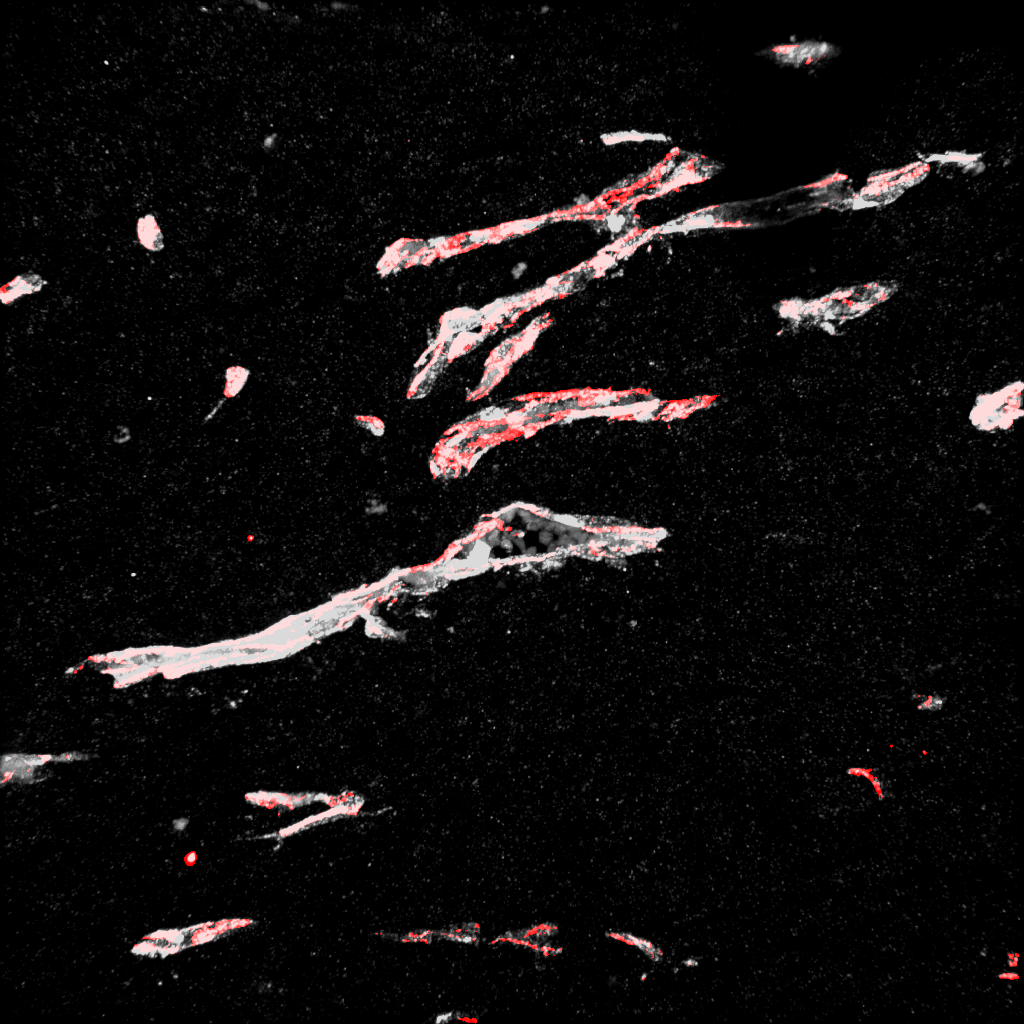

Supplement: Supplementary file 3 — Source data Fig. 3 [file 44318_2024_78_MOESM3_ESM.zip › Figure 3/3E/sGCCtr-1.tif]

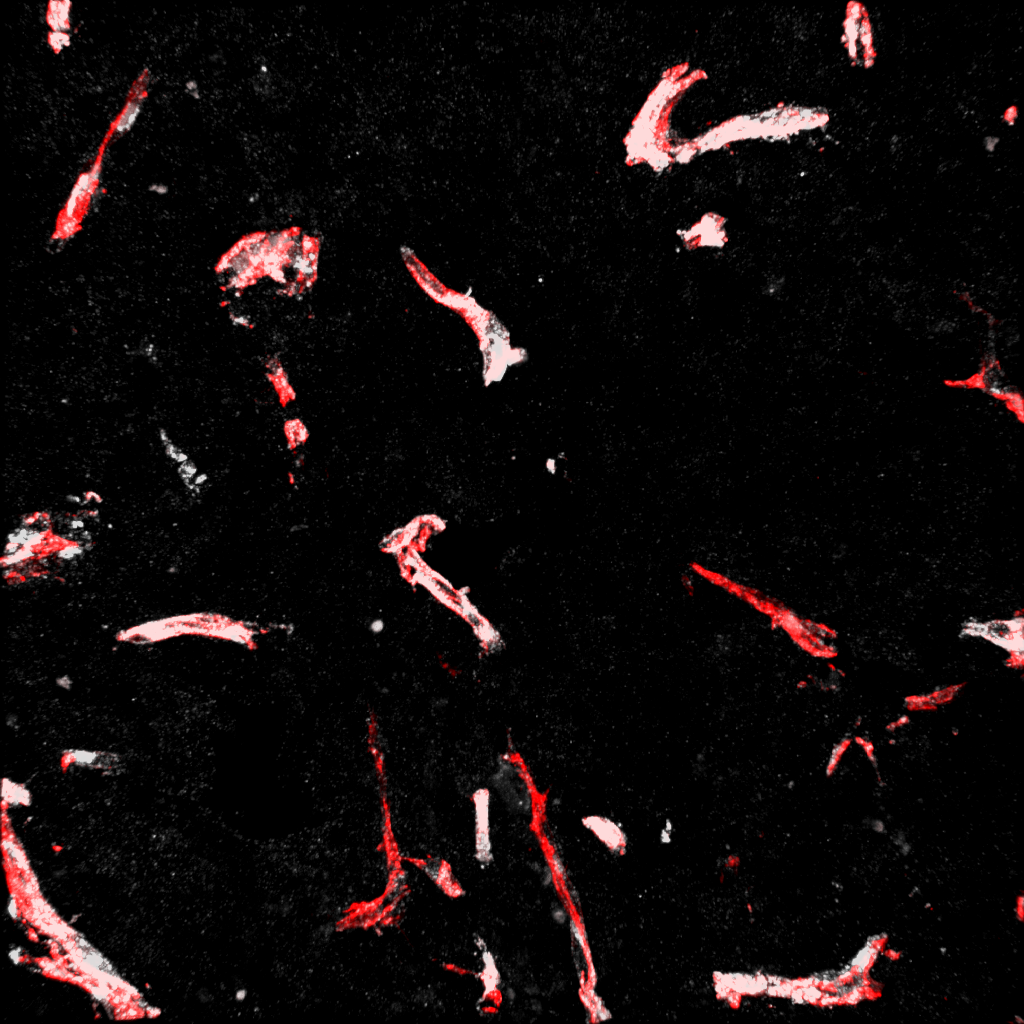

Supplement: Supplementary file 3 — Source data Fig. 3 [file 44318_2024_78_MOESM3_ESM.zip › Figure 3/3E/sGC╬öPC-1.tif]

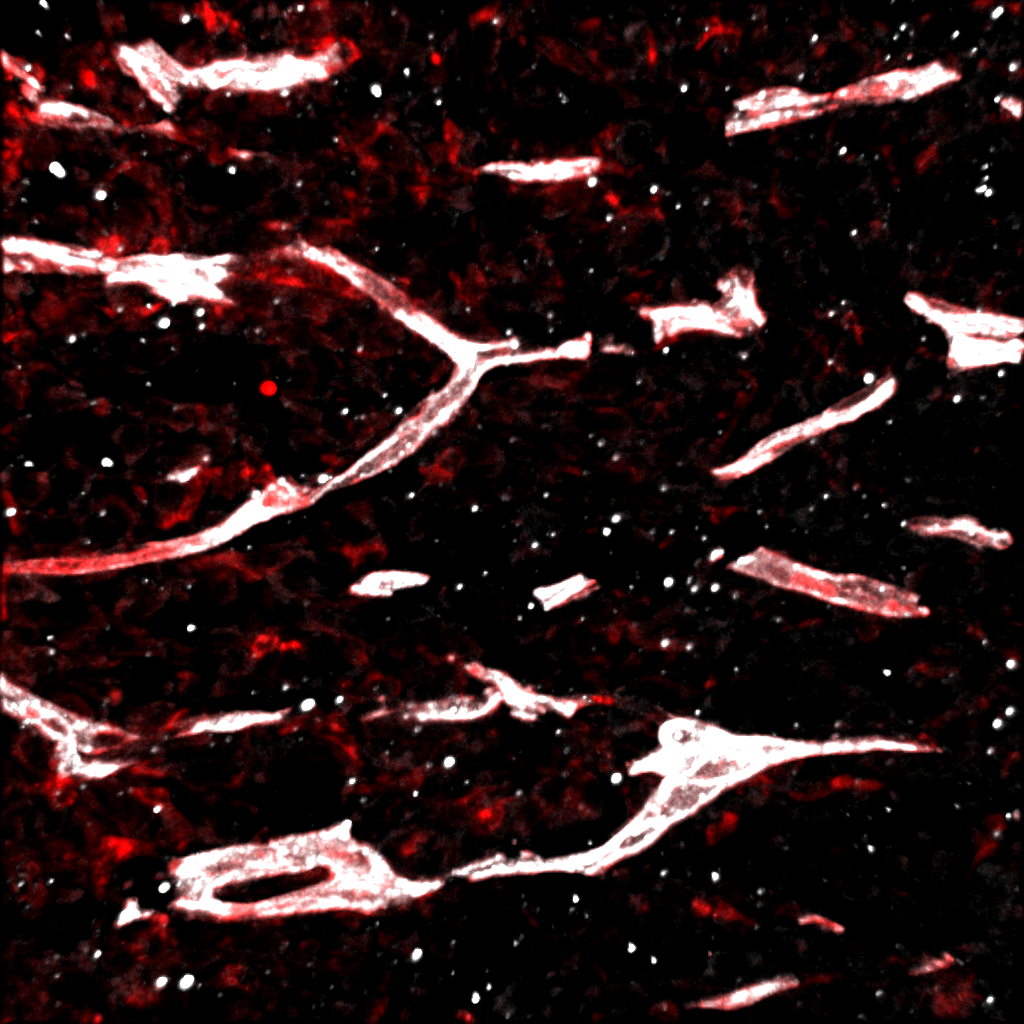

Supplement: Supplementary file 3 — Source data Fig. 3 [file 44318_2024_78_MOESM3_ESM.zip › Figure 3/3D/sGCCtr-1.tif]

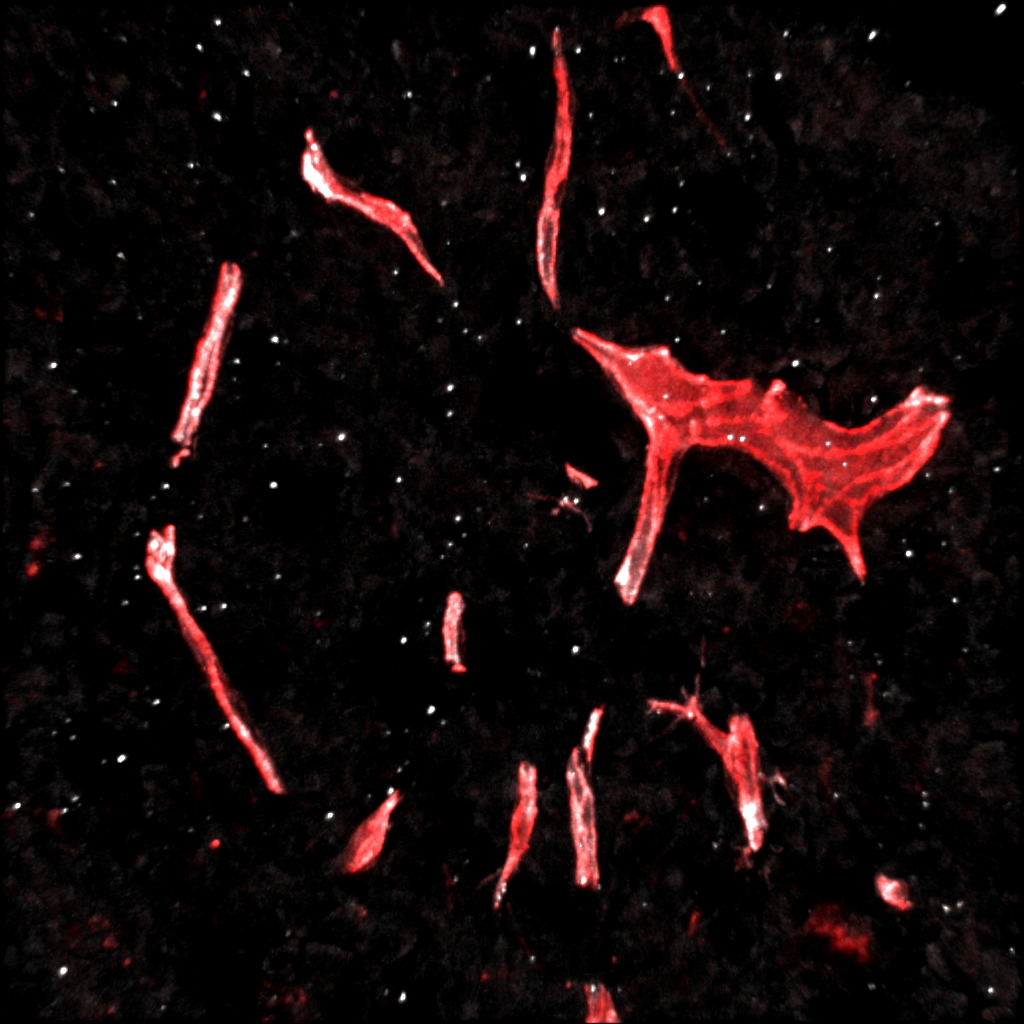

Supplement: Supplementary file 3 — Source data Fig. 3 [file 44318_2024_78_MOESM3_ESM.zip › Figure 3/3D/sGC╬öPC-1.tif]

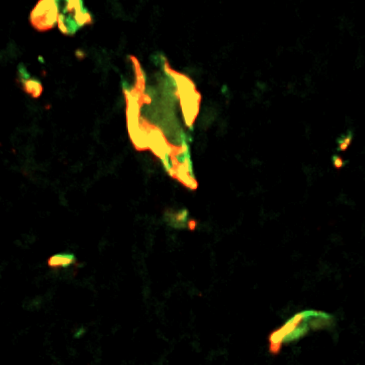

Supplement: Supplementary file 3 — Source data Fig. 3 [file 44318_2024_78_MOESM3_ESM.zip › Figure 3/3J/sGCCtr-3.tif]

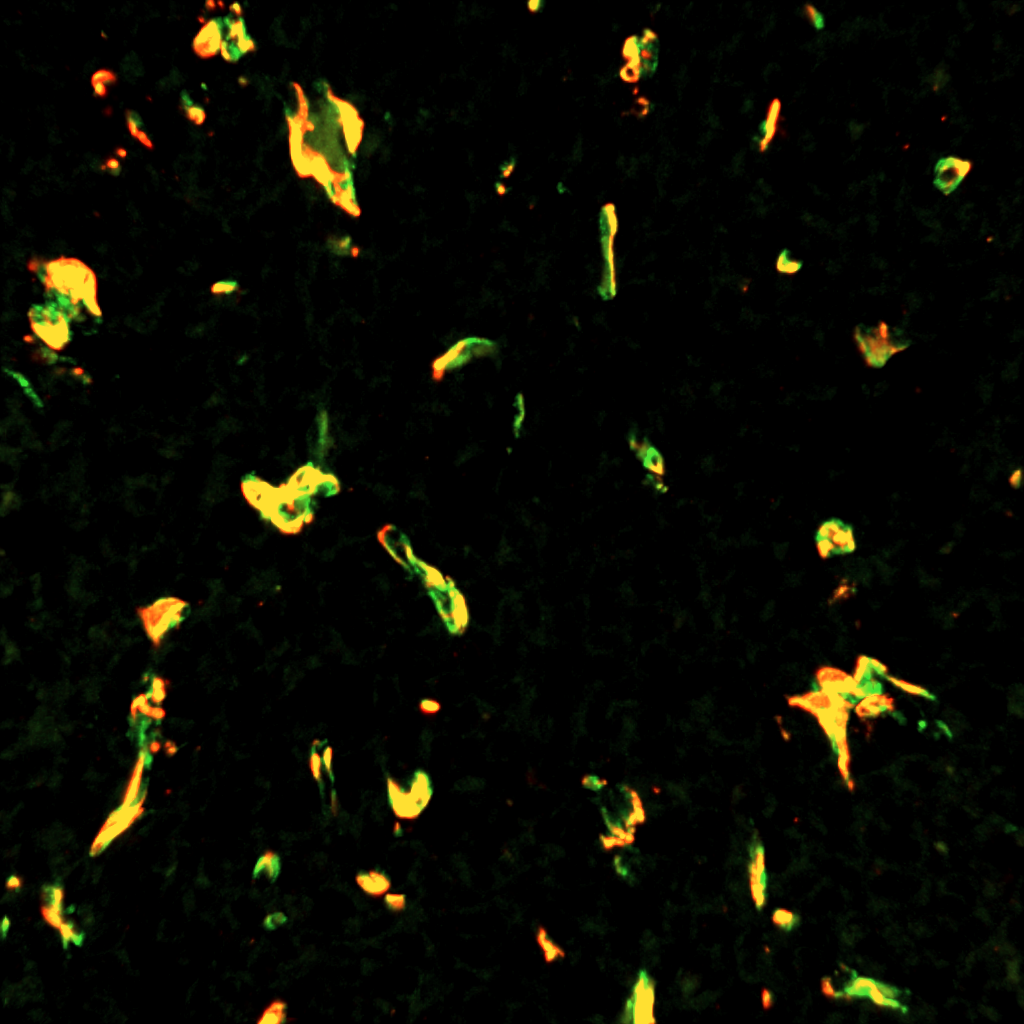

Supplement: Supplementary file 3 — Source data Fig. 3 [file 44318_2024_78_MOESM3_ESM.zip › Figure 3/3J/sGCCtr-2.tif]

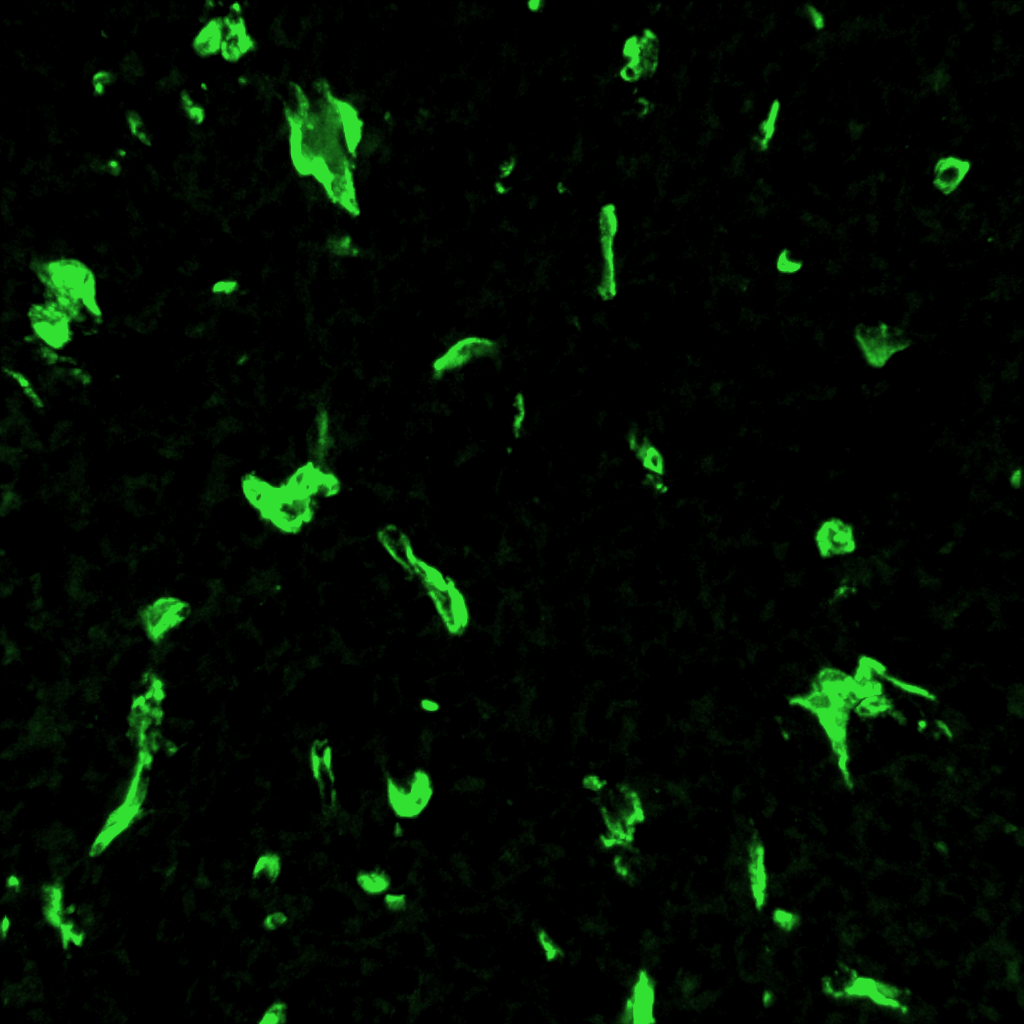

Supplement: Supplementary file 3 — Source data Fig. 3 [file 44318_2024_78_MOESM3_ESM.zip › Figure 3/3J/sGCCtr-1.tif]

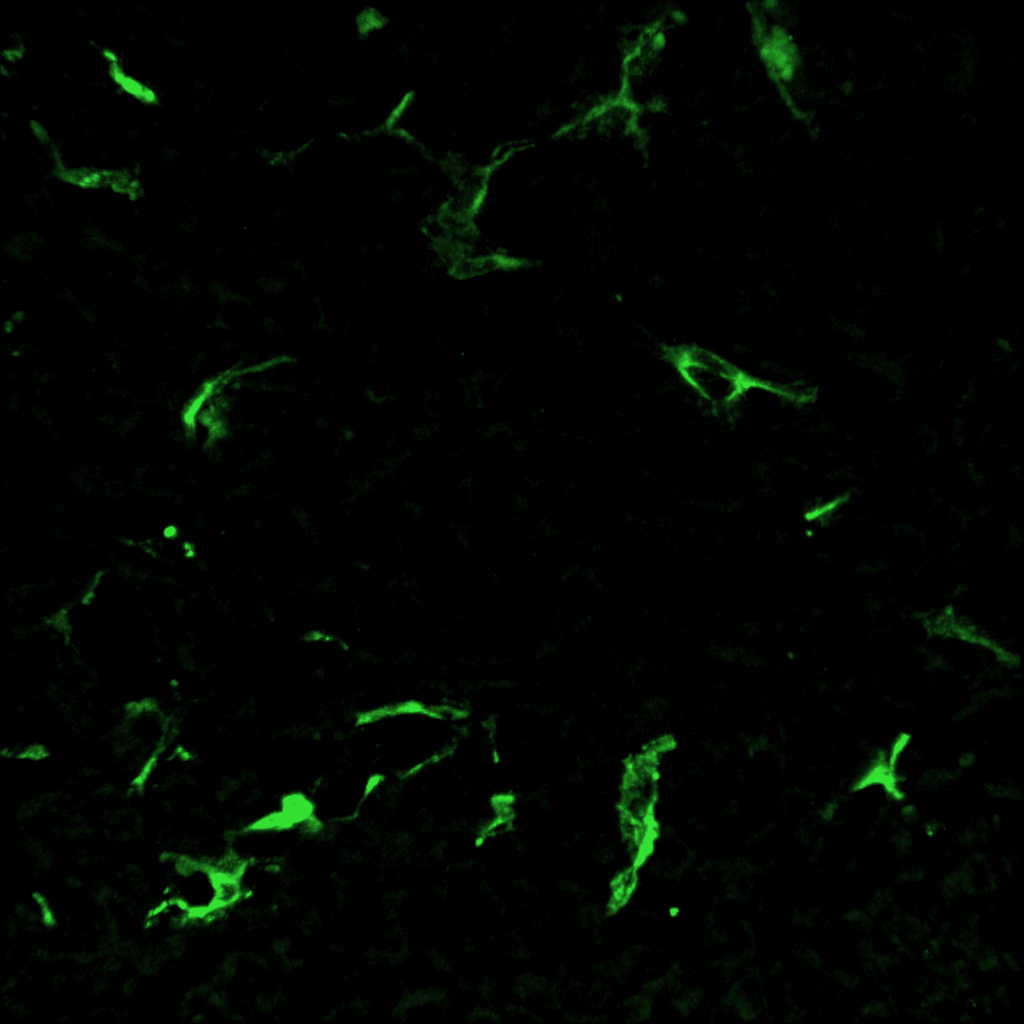

Supplement: Supplementary file 3 — Source data Fig. 3 [file 44318_2024_78_MOESM3_ESM.zip › Figure 3/3J/sGC╬öPC-1.tif]

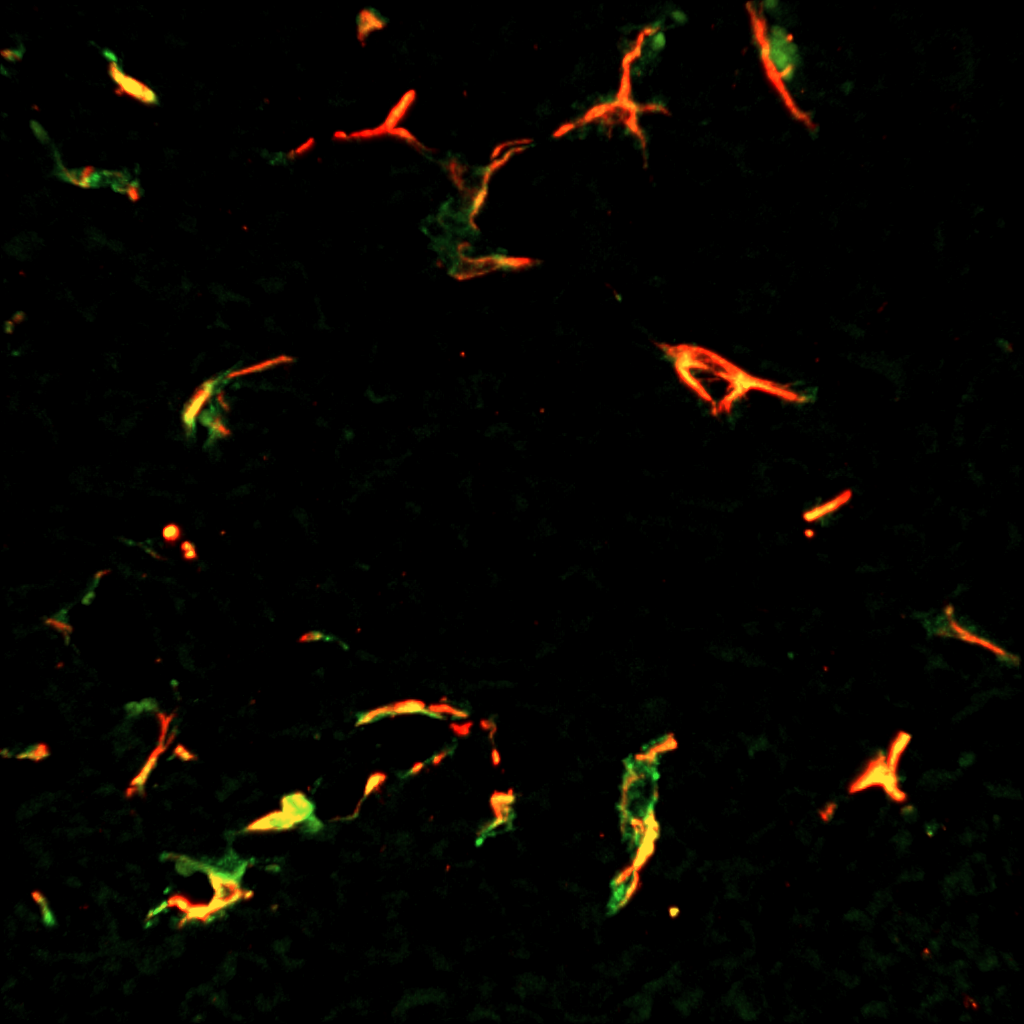

Supplement: Supplementary file 3 — Source data Fig. 3 [file 44318_2024_78_MOESM3_ESM.zip › Figure 3/3J/sGC╬öPC-2.tif]

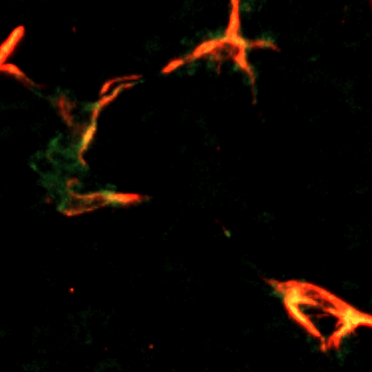

Supplement: Supplementary file 3 — Source data Fig. 3 [file 44318_2024_78_MOESM3_ESM.zip › Figure 3/3J/sGC╬öPC-3.tif]

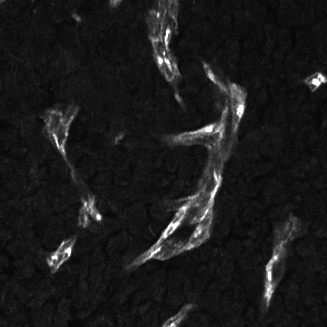

Supplement: Supplementary file 3 — Source data Fig. 3 [file 44318_2024_78_MOESM3_ESM.zip › Figure 3/3F/sGCCtr-3.tif]

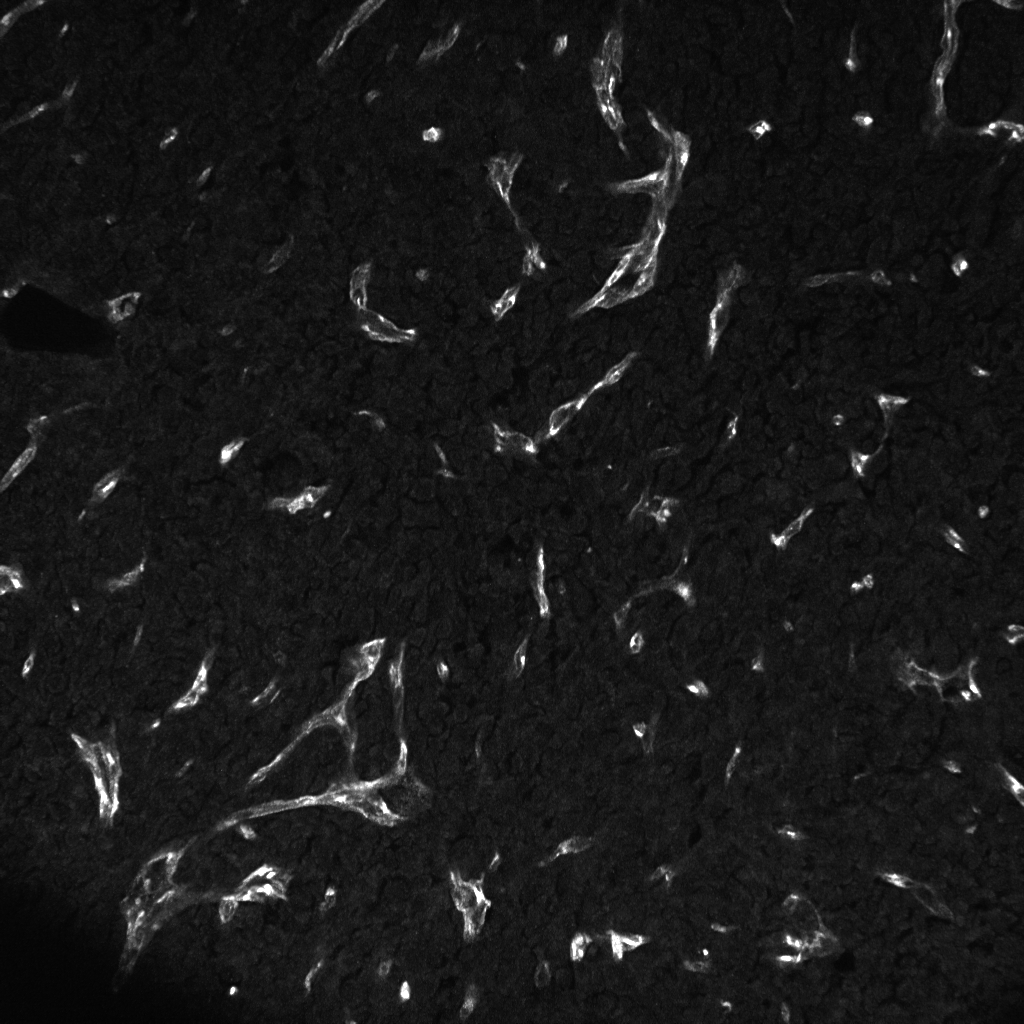

Supplement: Supplementary file 3 — Source data Fig. 3 [file 44318_2024_78_MOESM3_ESM.zip › Figure 3/3F/sGCCtr-2.tif]

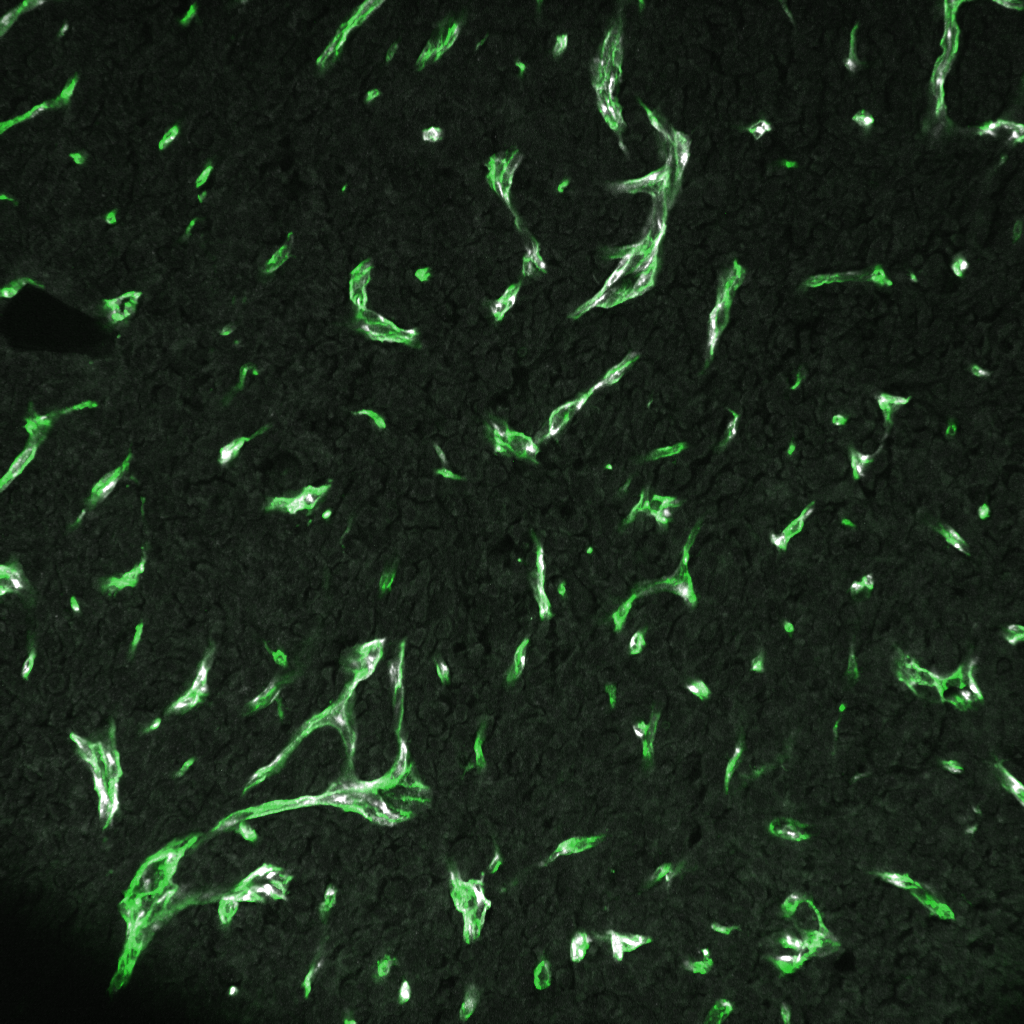

Supplement: Supplementary file 3 — Source data Fig. 3 [file 44318_2024_78_MOESM3_ESM.zip › Figure 3/3F/sGCCtr-1.tif]

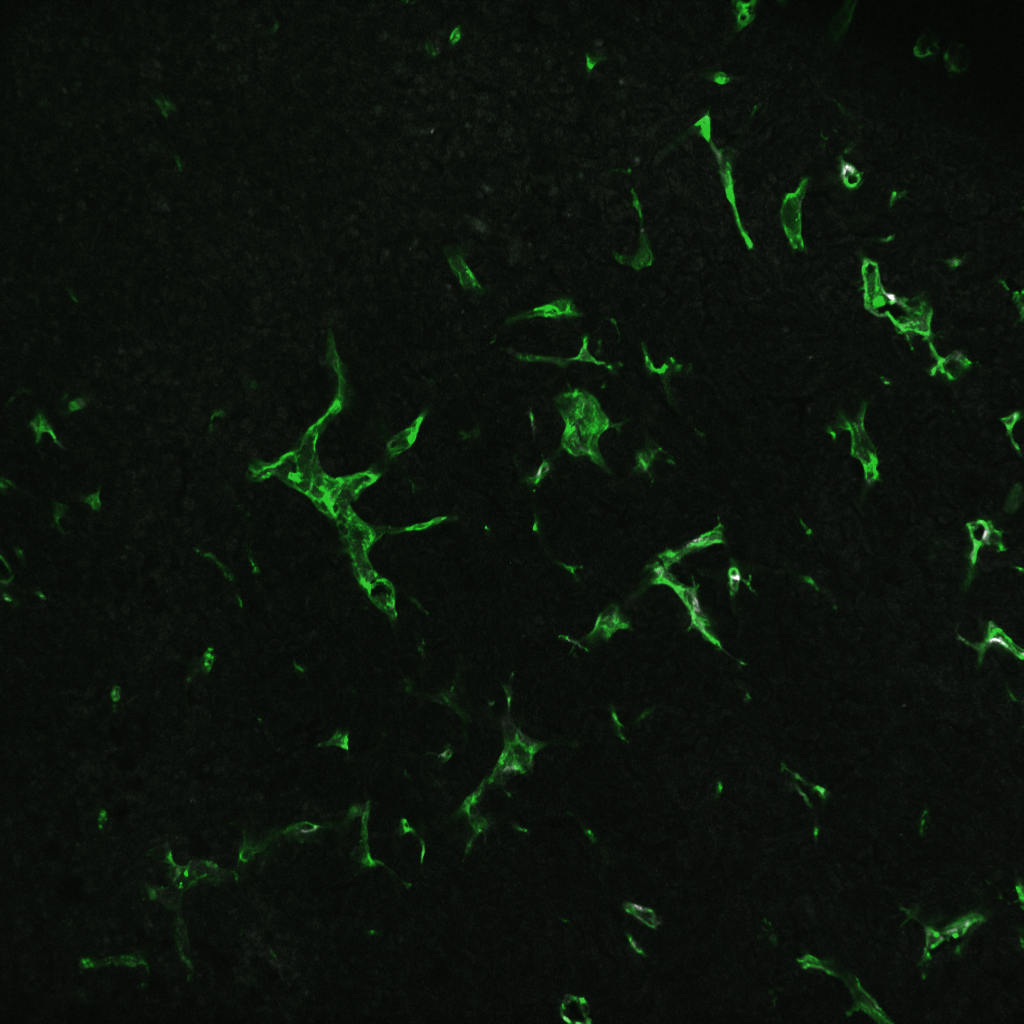

Supplement: Supplementary file 3 — Source data Fig. 3 [file 44318_2024_78_MOESM3_ESM.zip › Figure 3/3F/sGC╬öPC-1.tif]

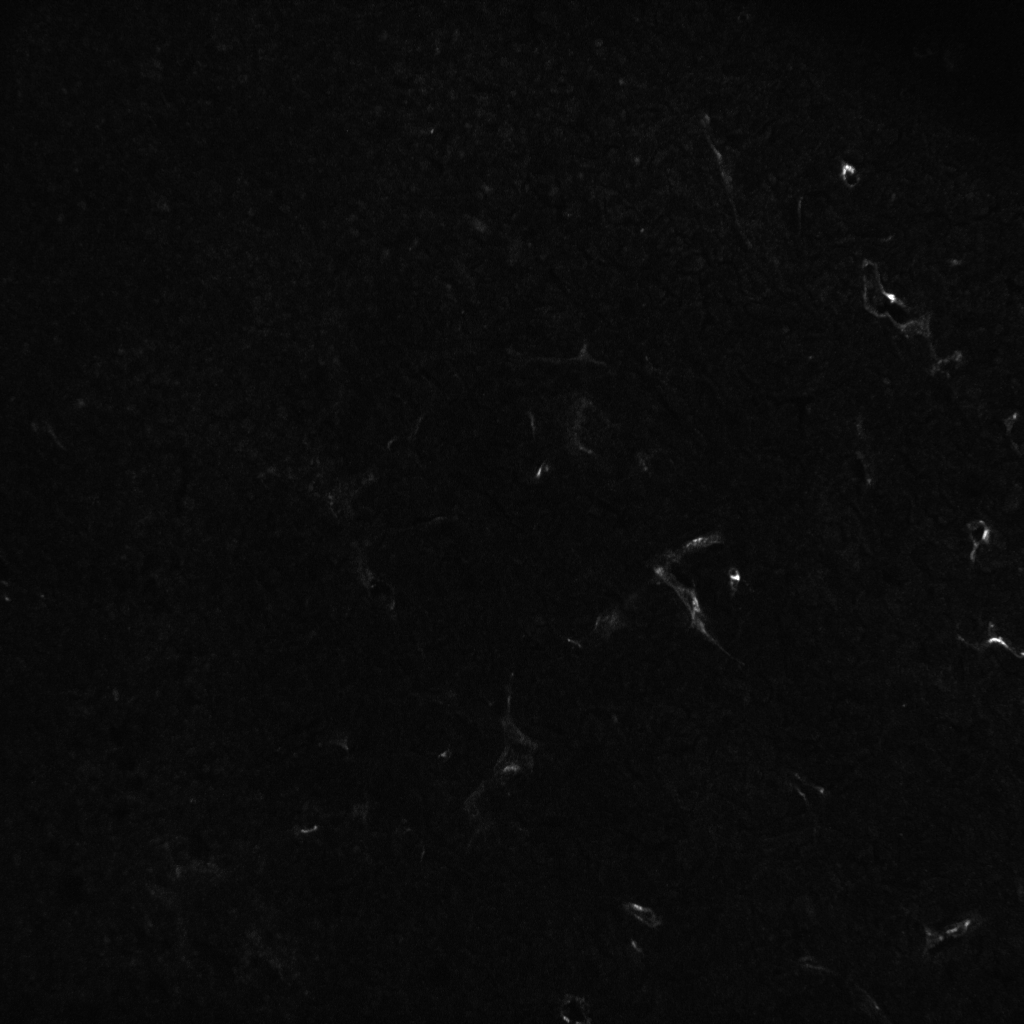

Supplement: Supplementary file 3 — Source data Fig. 3 [file 44318_2024_78_MOESM3_ESM.zip › Figure 3/3F/sGC╬öPC-2.tif]

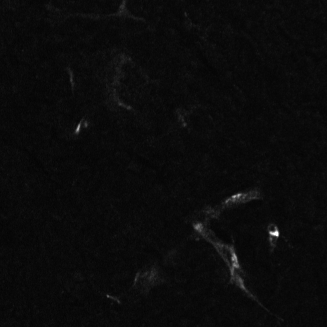

Supplement: Supplementary file 3 — Source data Fig. 3 [file 44318_2024_78_MOESM3_ESM.zip › Figure 3/3F/sGC╬öPC-3.tif]

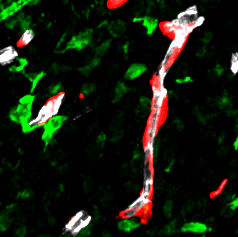

Supplement: Supplementary file 4 — Source data Fig. 4 [file 44318_2024_78_MOESM4_ESM.zip › Figure 4/4J/sGCCtr tdTomato-3.tif]

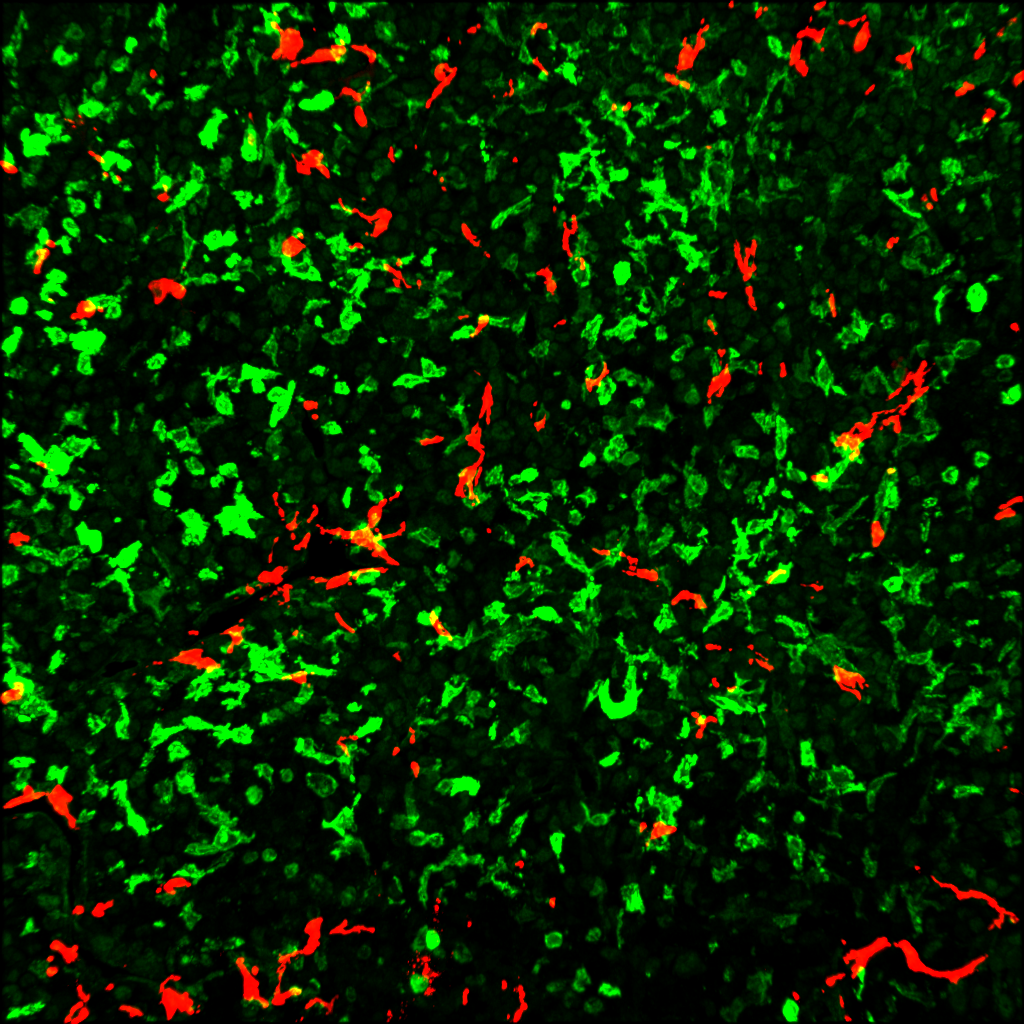

Supplement: Supplementary file 4 — Source data Fig. 4 [file 44318_2024_78_MOESM4_ESM.zip › Figure 4/4J/sGC╬öpc tdTomato-2.tif]

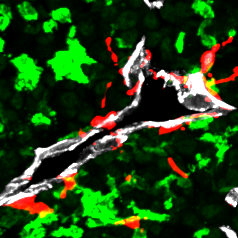

Supplement: Supplementary file 4 — Source data Fig. 4 [file 44318_2024_78_MOESM4_ESM.zip › Figure 4/4J/sGC╬öpc tdTomato-3.tif]

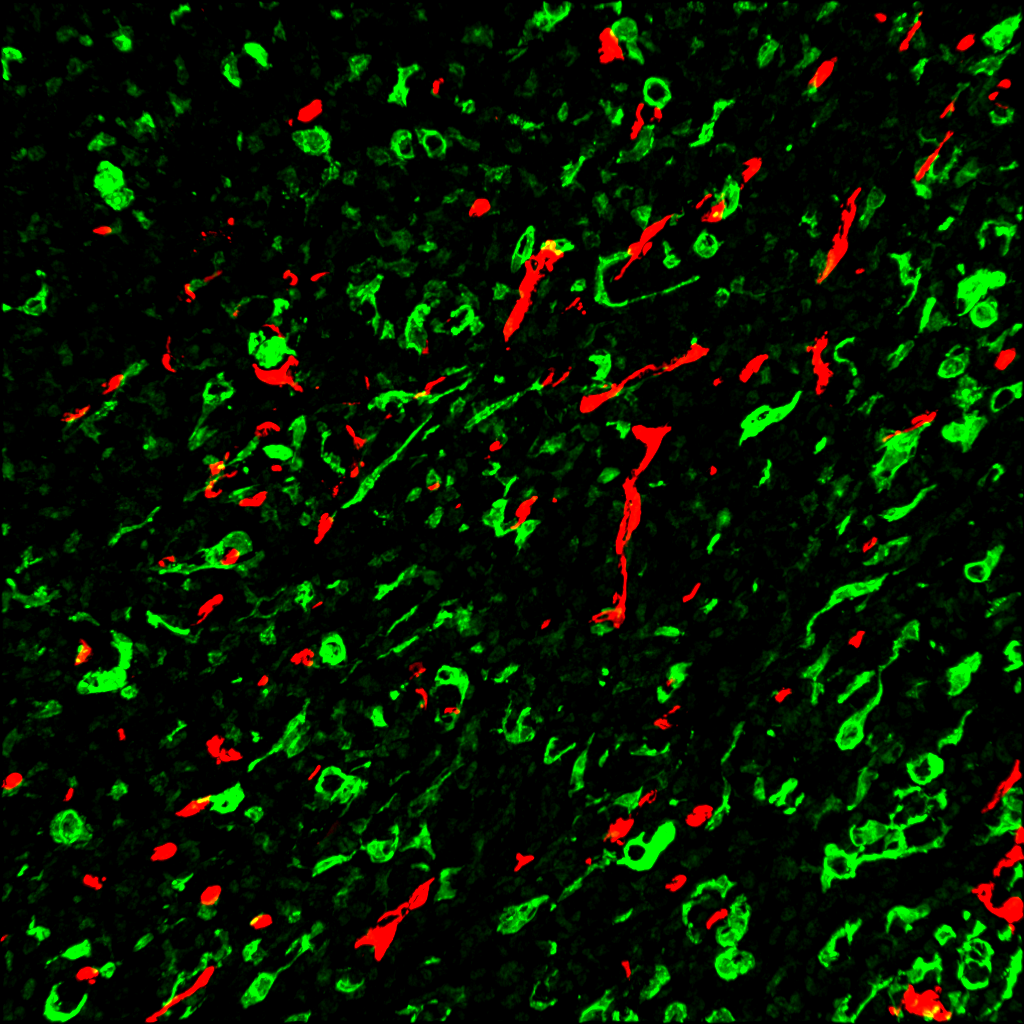

Supplement: Supplementary file 4 — Source data Fig. 4 [file 44318_2024_78_MOESM4_ESM.zip › Figure 4/4J/sGCCtr tdTomato-2.tif]

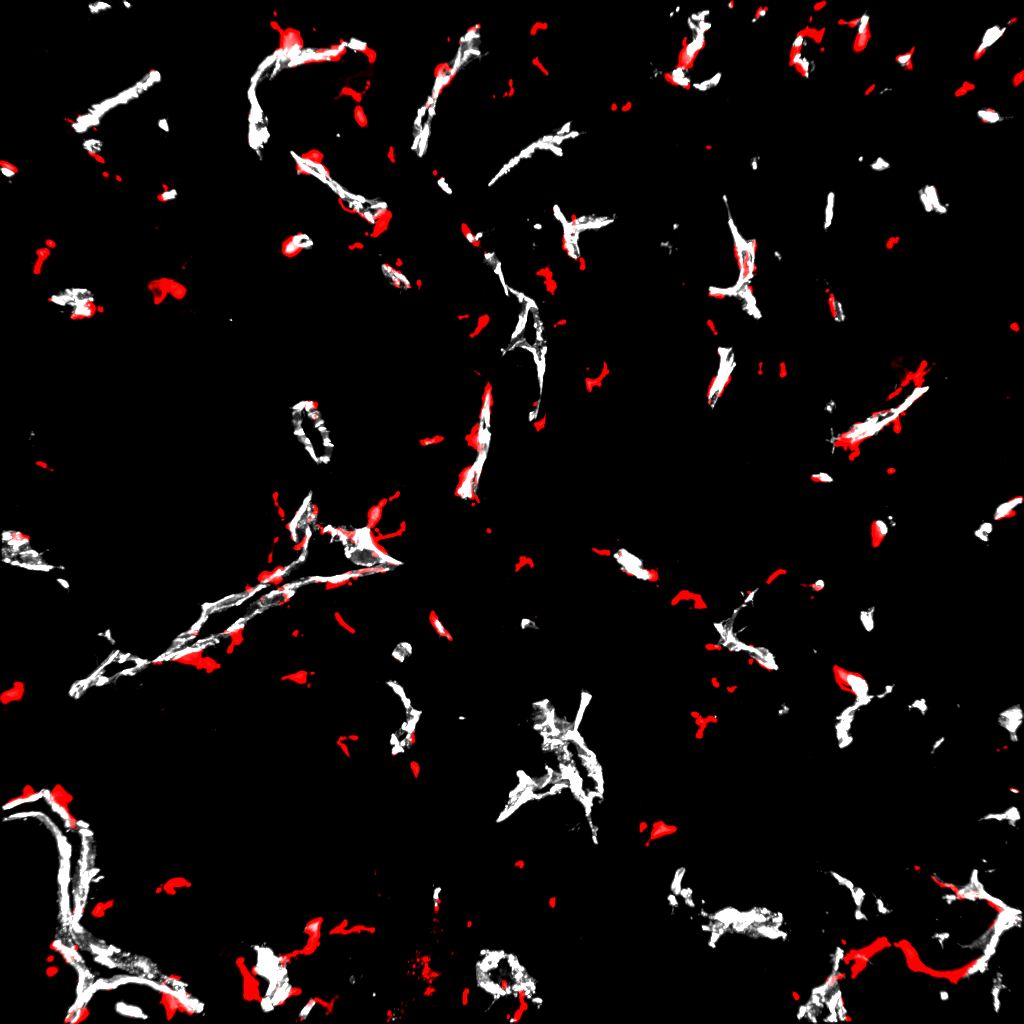

Supplement: Supplementary file 4 — Source data Fig. 4 [file 44318_2024_78_MOESM4_ESM.zip › Figure 4/4J/sGC╬öpc tdTomato-1.tif]

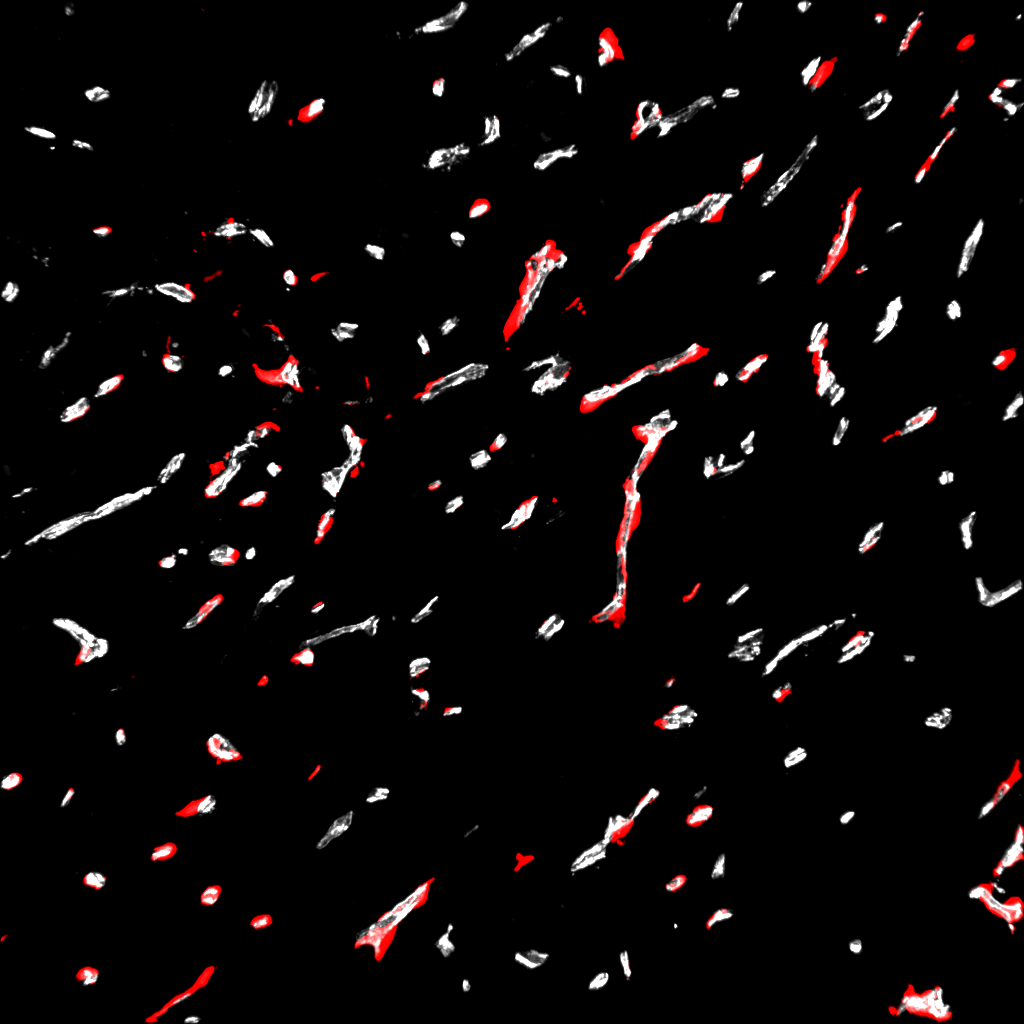

Supplement: Supplementary file 4 — Source data Fig. 4 [file 44318_2024_78_MOESM4_ESM.zip › Figure 4/4J/sGCCtr tdTomato-1.tif]

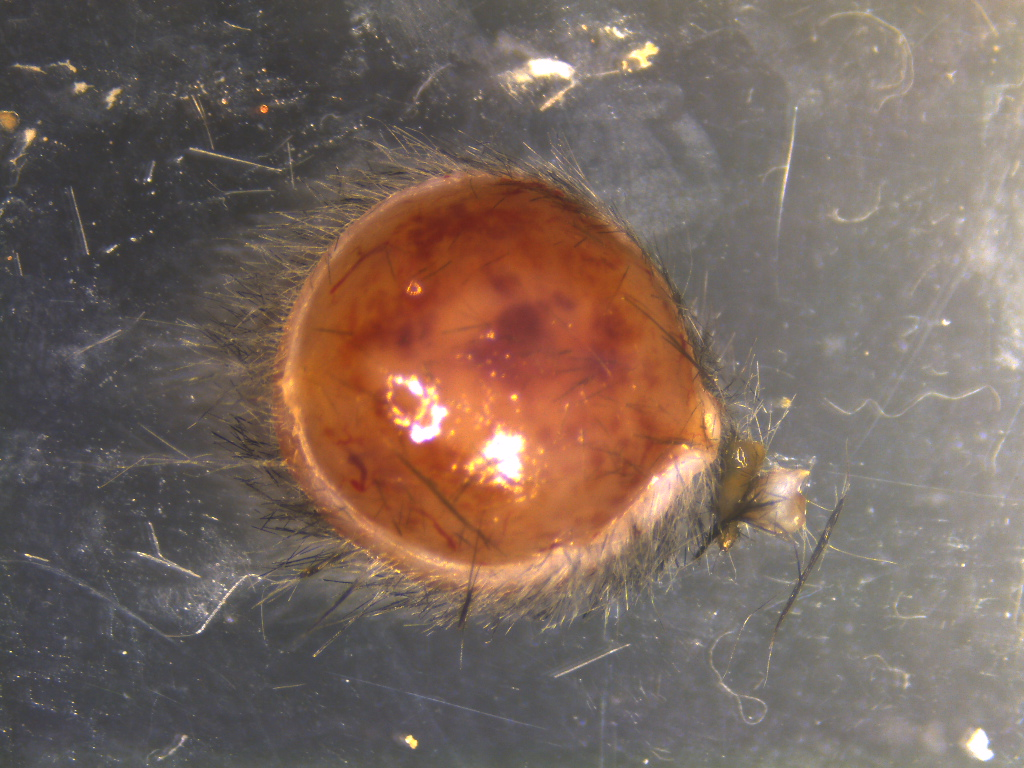

Supplement: Supplementary file 6 — Source data Fig. 6 [file 44318_2024_78_MOESM6_ESM.zip › Figure 6/6I/ODQ+Vehicle6.tif]

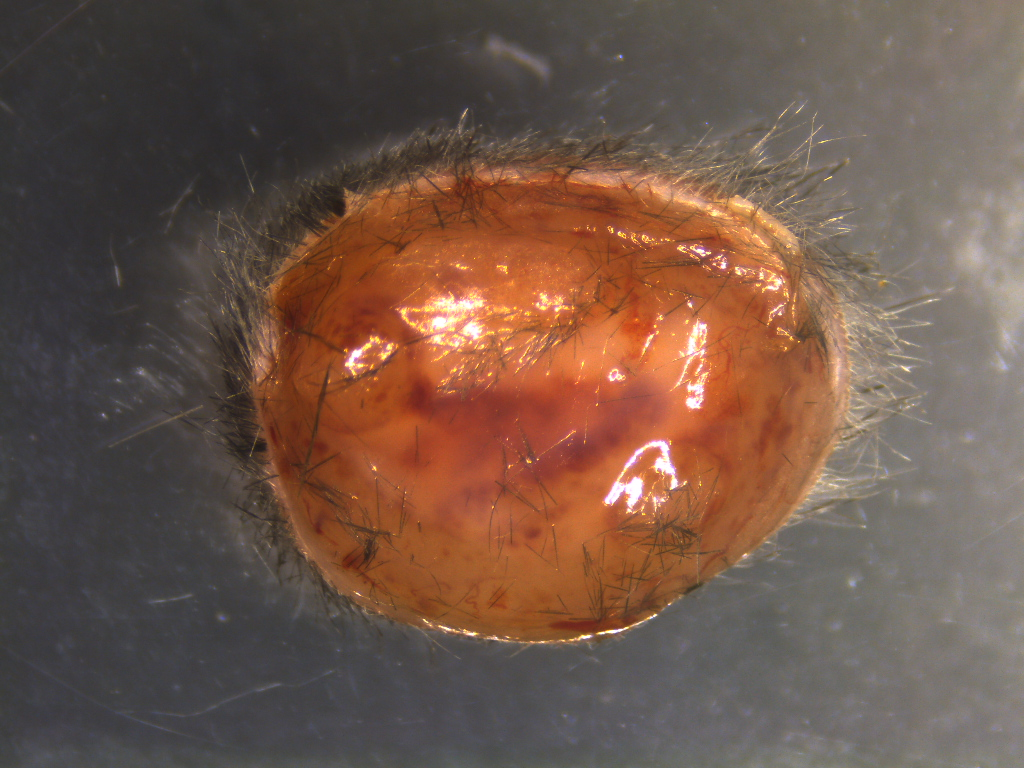

Supplement: Supplementary file 6 — Source data Fig. 6 [file 44318_2024_78_MOESM6_ESM.zip › Figure 6/6I/Vehicle+Vehicle-7.tif]

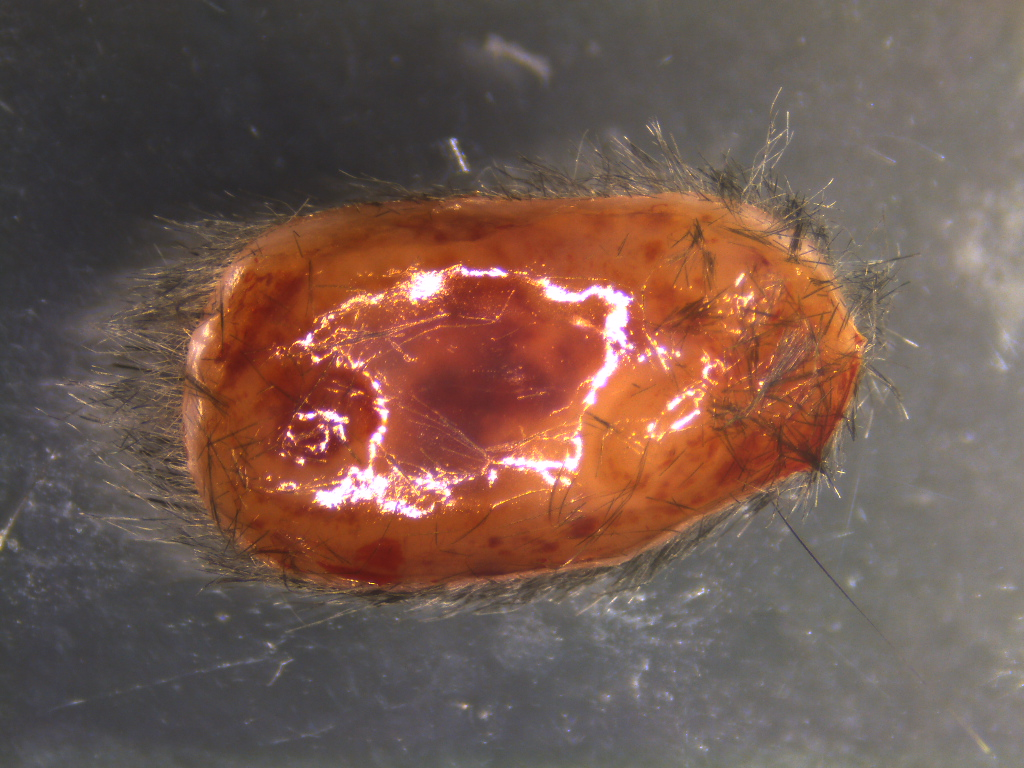

Supplement: Supplementary file 6 — Source data Fig. 6 [file 44318_2024_78_MOESM6_ESM.zip › Figure 6/6I/Vehicle+Vehicle-6.tif]

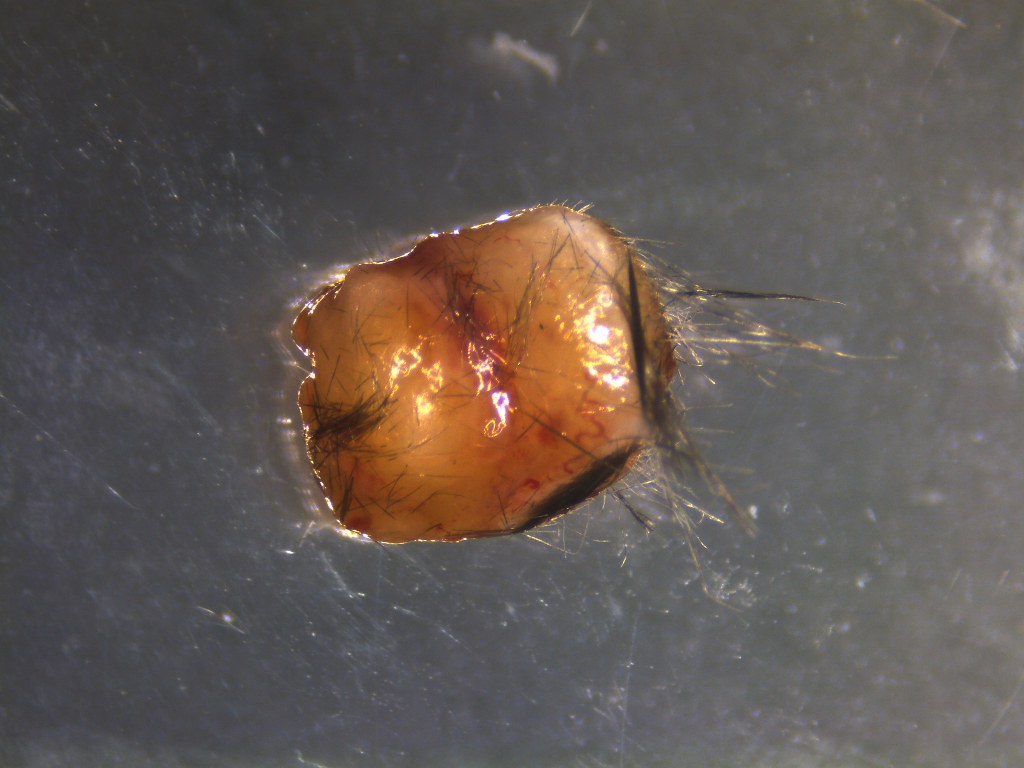

Supplement: Supplementary file 6 — Source data Fig. 6 [file 44318_2024_78_MOESM6_ESM.zip › Figure 6/6I/ODQ+Vehicle7.tif]

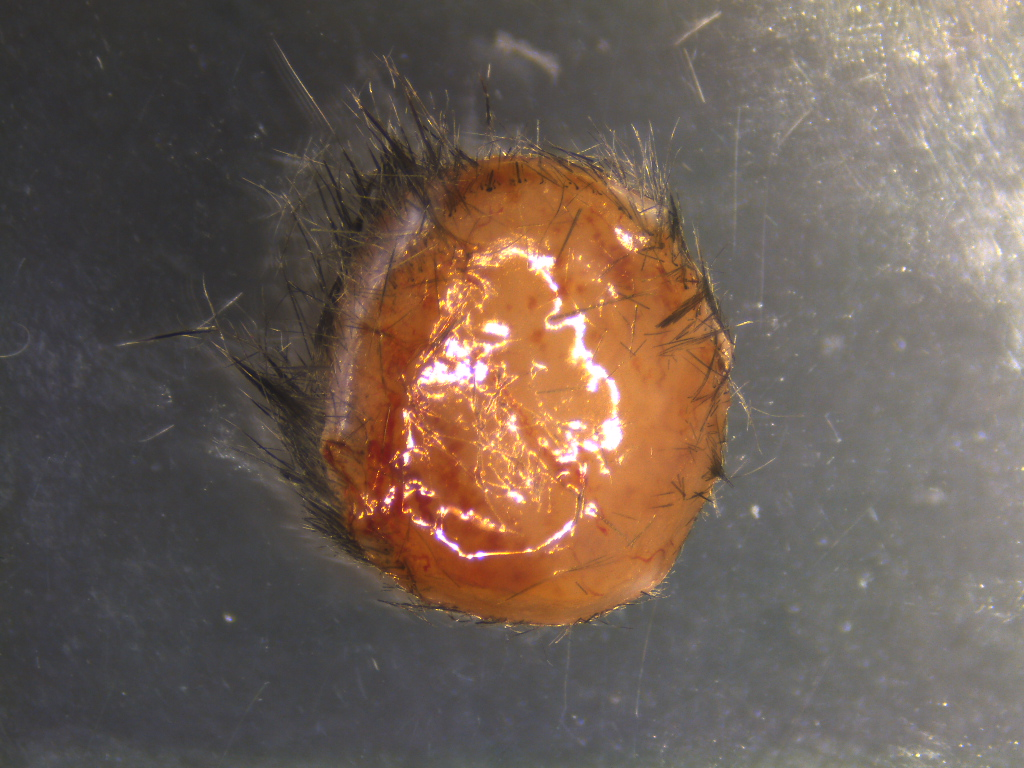

Supplement: Supplementary file 6 — Source data Fig. 6 [file 44318_2024_78_MOESM6_ESM.zip › Figure 6/6I/ODQ+Vehicle5.tif]

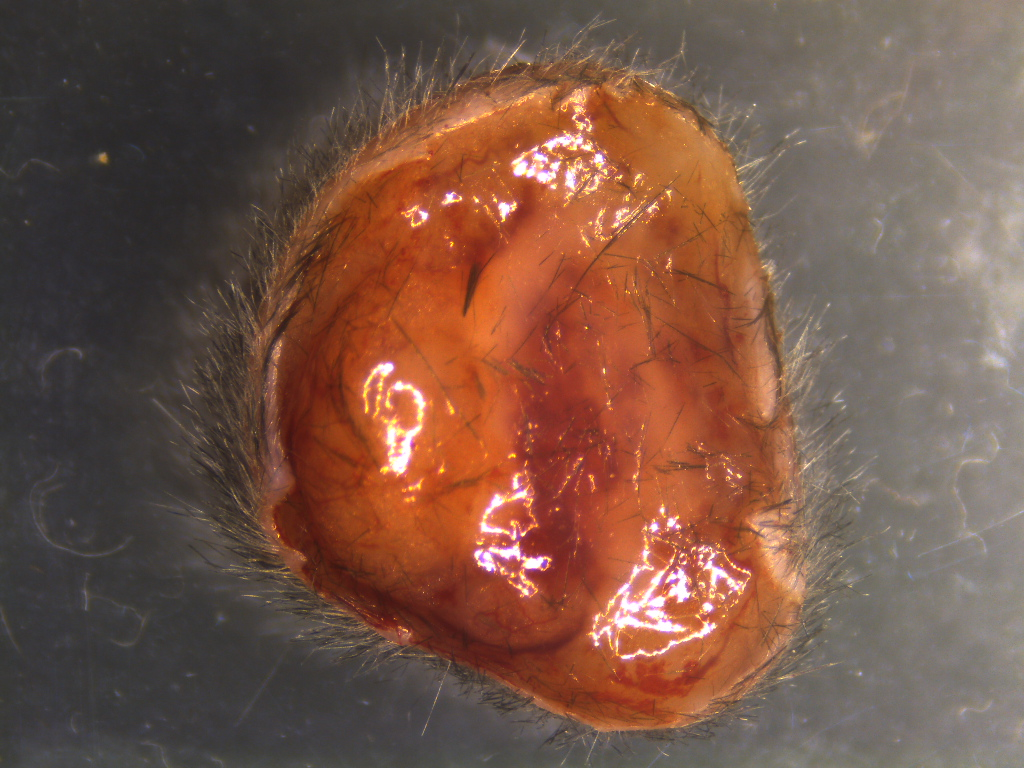

Supplement: Supplementary file 6 — Source data Fig. 6 [file 44318_2024_78_MOESM6_ESM.zip › Figure 6/6I/Vehicle+Vehicle-4.tif]

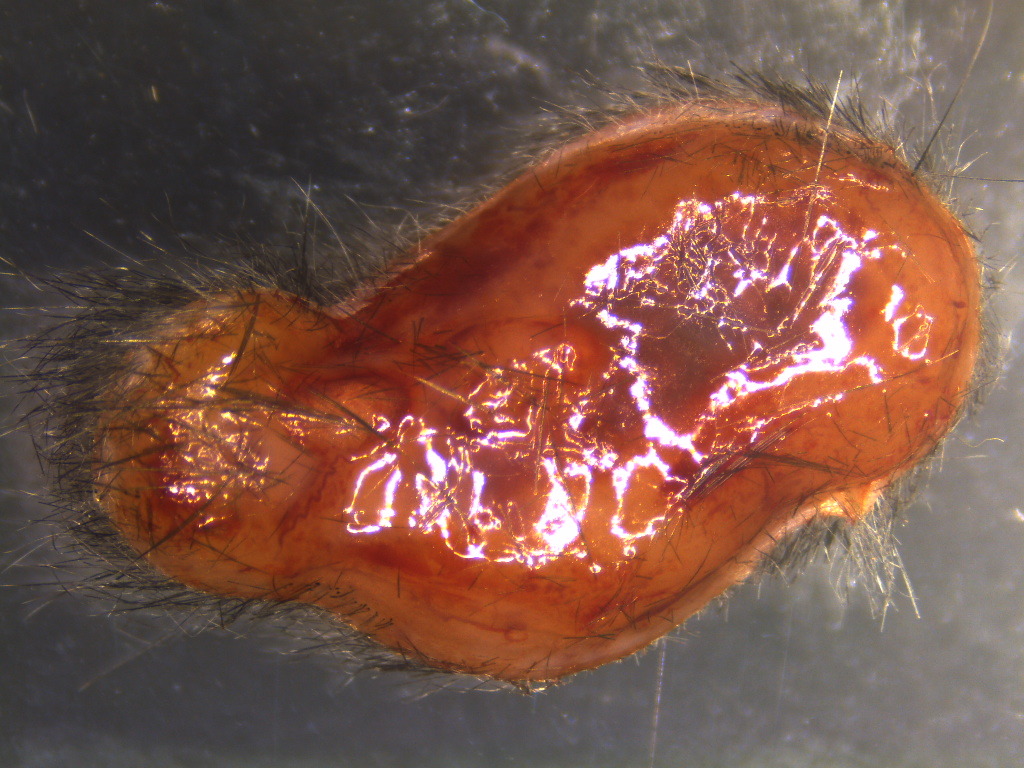

Supplement: Supplementary file 6 — Source data Fig. 6 [file 44318_2024_78_MOESM6_ESM.zip › Figure 6/6I/ODQ+Vehicle-1.tif]

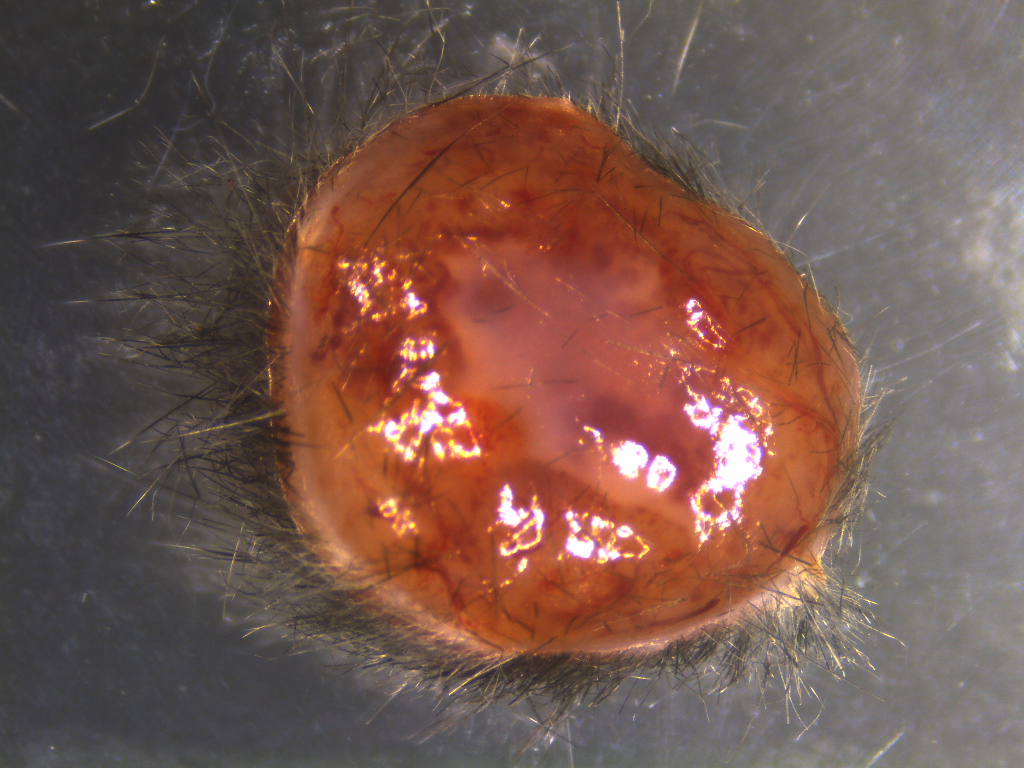

Supplement: Supplementary file 6 — Source data Fig. 6 [file 44318_2024_78_MOESM6_ESM.zip › Figure 6/6I/Vehicle+Vehicle-5.tif]

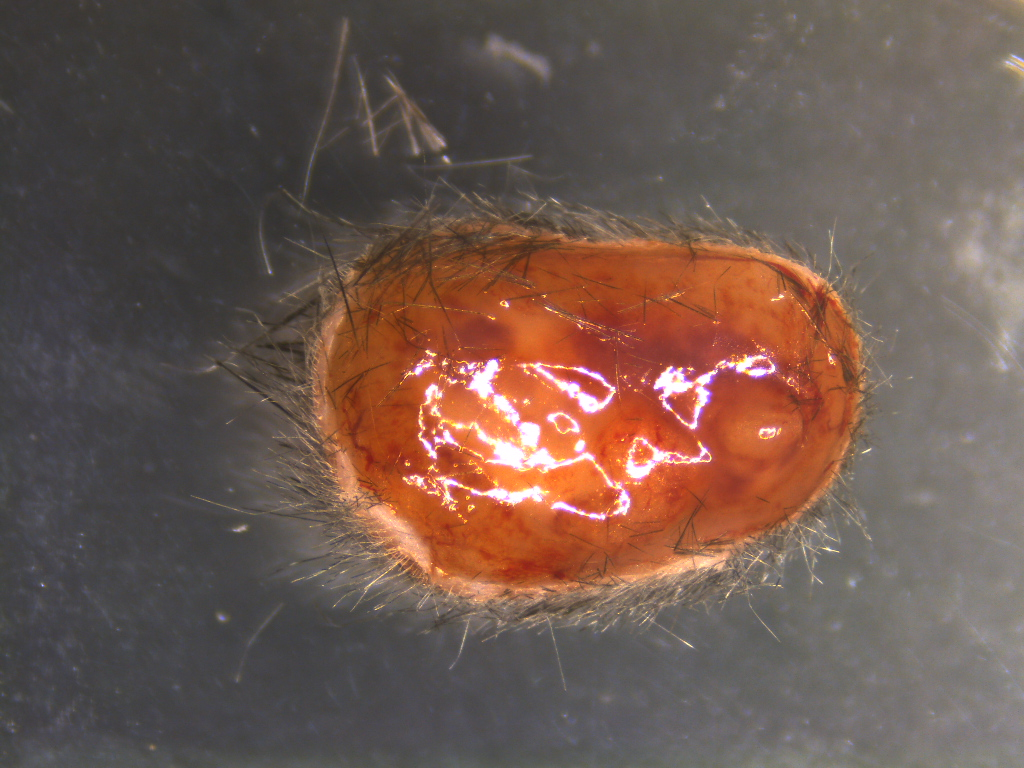

Supplement: Supplementary file 6 — Source data Fig. 6 [file 44318_2024_78_MOESM6_ESM.zip › Figure 6/6I/ODQ+Vehicle4.tif]

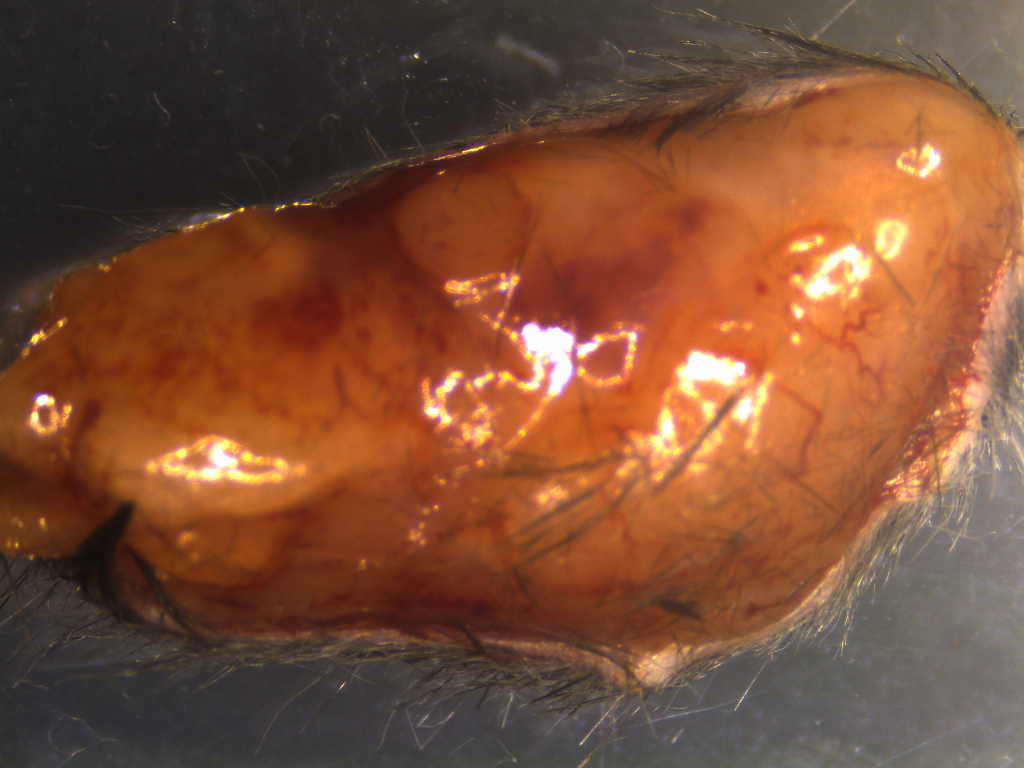

Supplement: Supplementary file 6 — Source data Fig. 6 [file 44318_2024_78_MOESM6_ESM.zip › Figure 6/6I/Vehicle+Vehicle-1.tif]

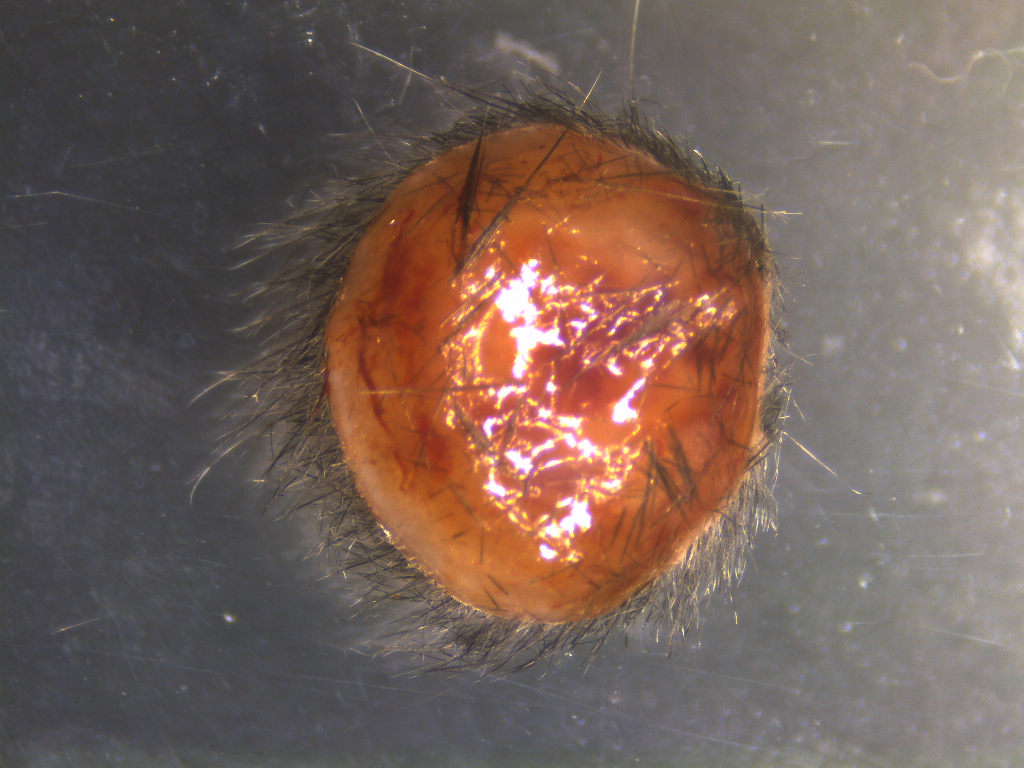

Supplement: Supplementary file 6 — Source data Fig. 6 [file 44318_2024_78_MOESM6_ESM.zip › Figure 6/6I/ODQ+Vehicle3.tif]

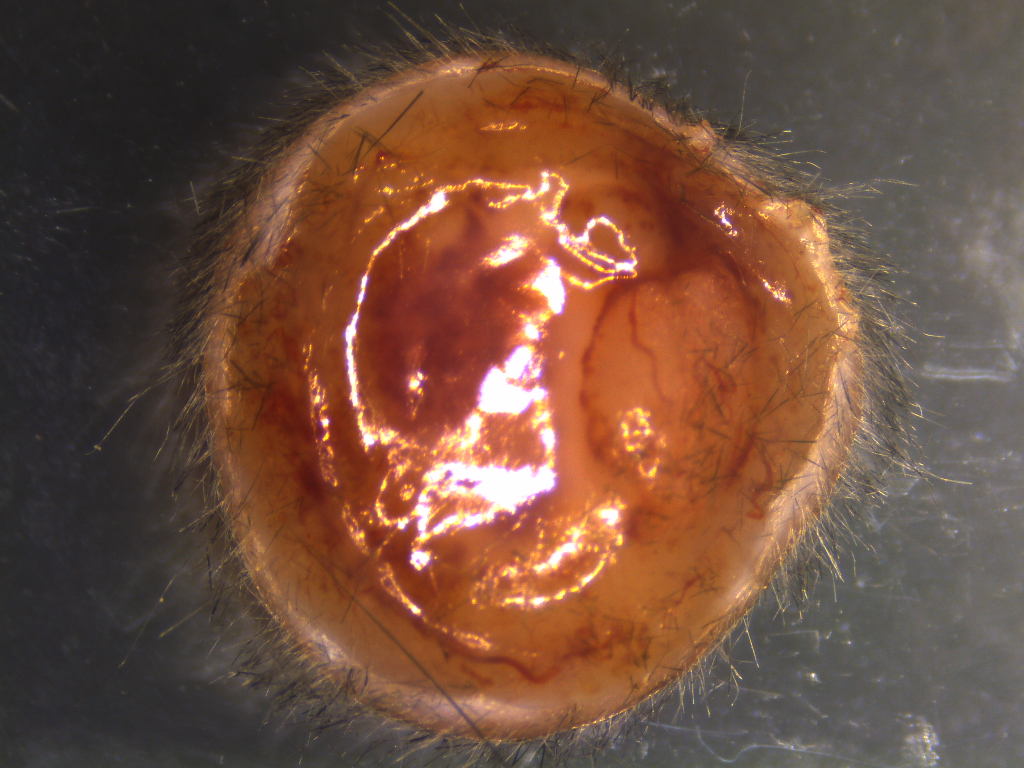

Supplement: Supplementary file 6 — Source data Fig. 6 [file 44318_2024_78_MOESM6_ESM.zip › Figure 6/6I/Vehicle+Vehicle-2.tif]

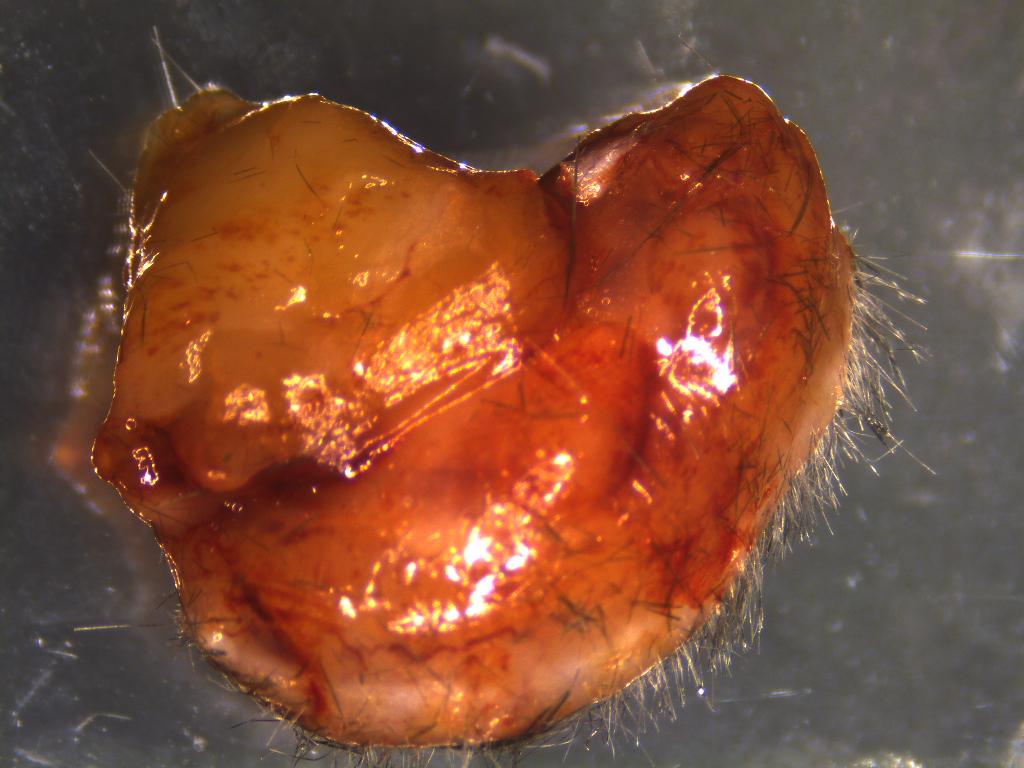

Supplement: Supplementary file 6 — Source data Fig. 6 [file 44318_2024_78_MOESM6_ESM.zip › Figure 6/6I/Vehicle+Vehicle-3.tif]

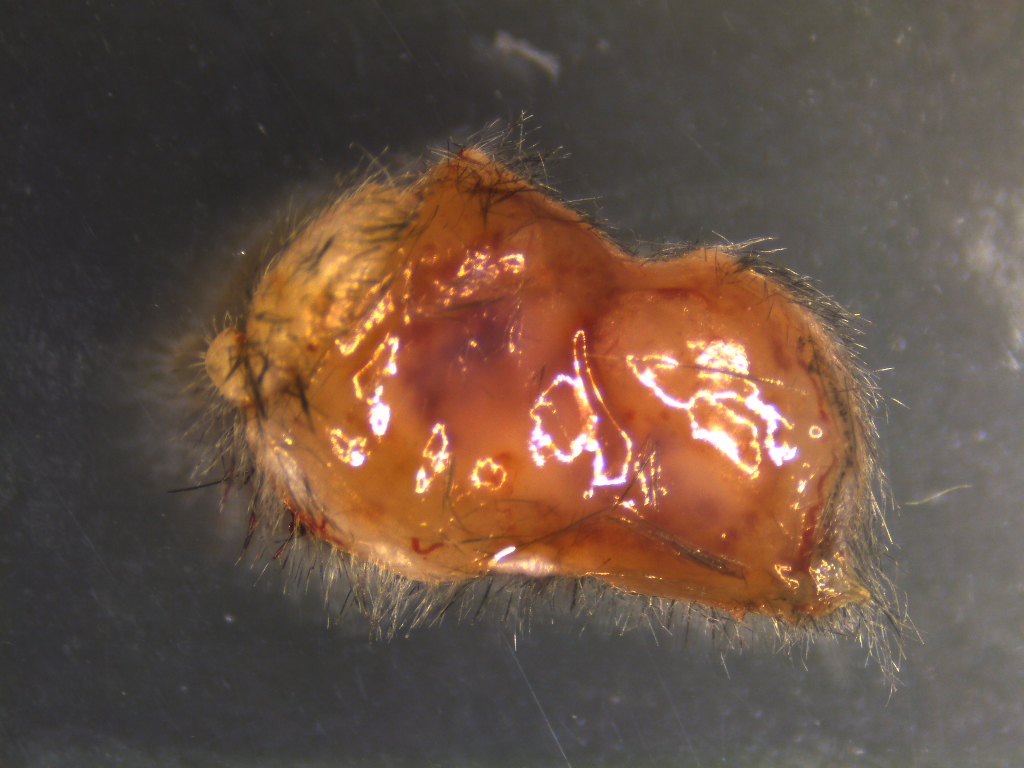

Supplement: Supplementary file 6 — Source data Fig. 6 [file 44318_2024_78_MOESM6_ESM.zip › Figure 6/6I/ODQ+Vehicle2.tif]

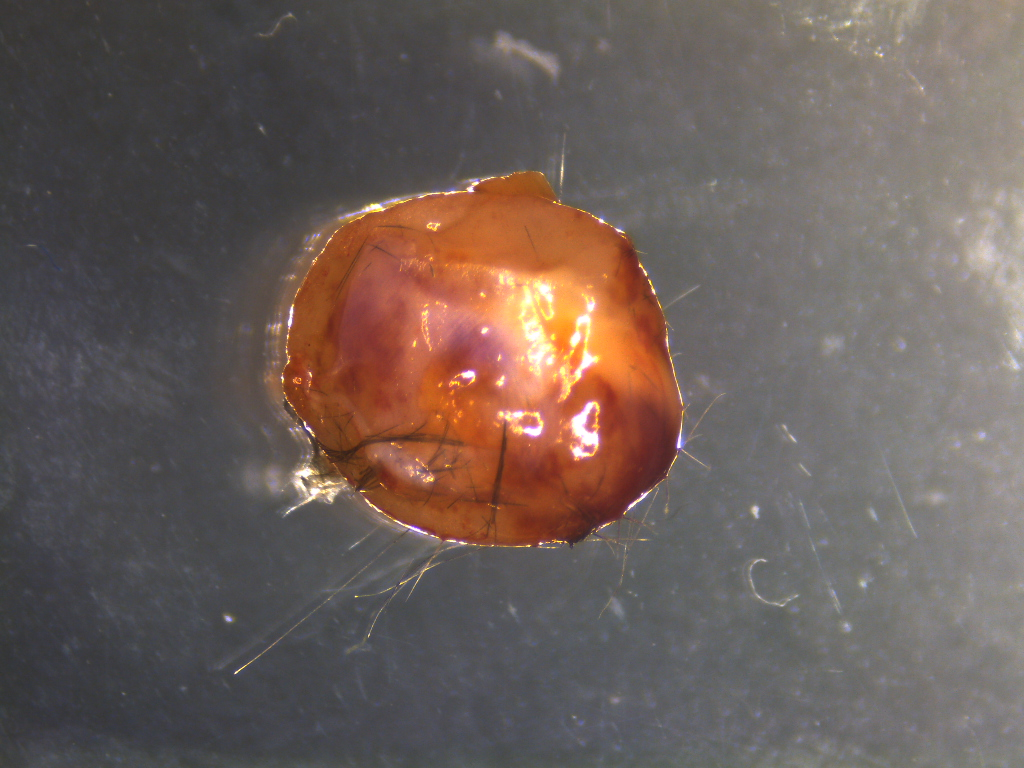

Supplement: Supplementary file 6 — Source data Fig. 6 [file 44318_2024_78_MOESM6_ESM.zip › Figure 6/6I/ODQ+Fruquintinib-3.tif]

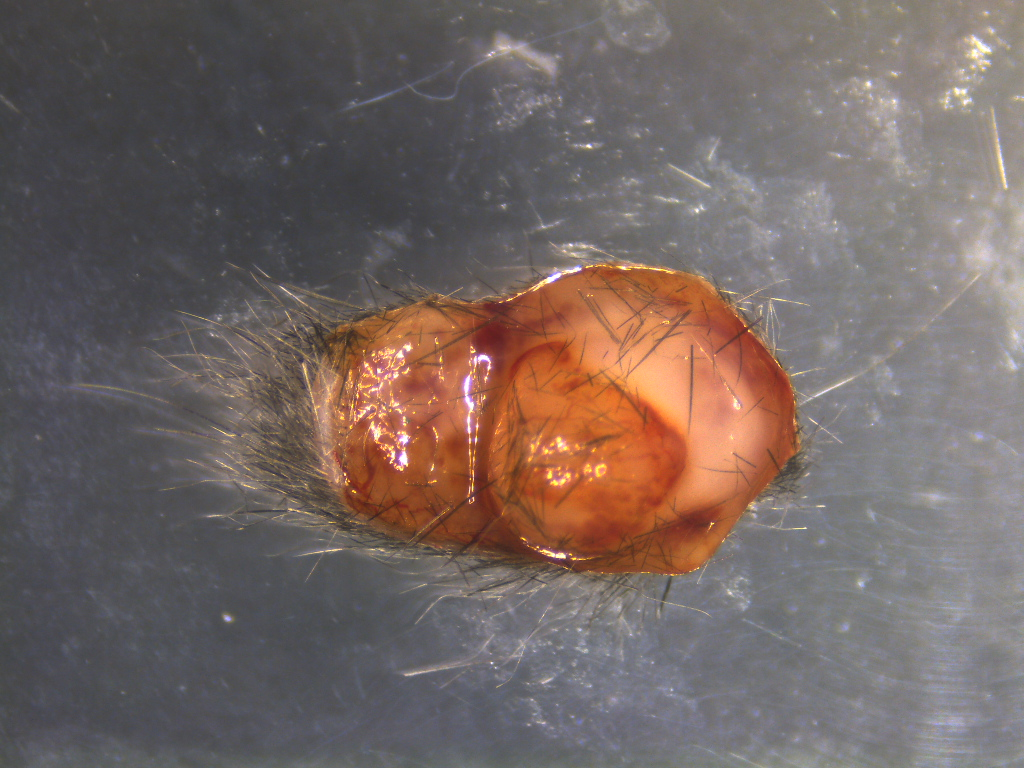

Supplement: Supplementary file 6 — Source data Fig. 6 [file 44318_2024_78_MOESM6_ESM.zip › Figure 6/6I/ODQ+Fruquintinib-2.tif]

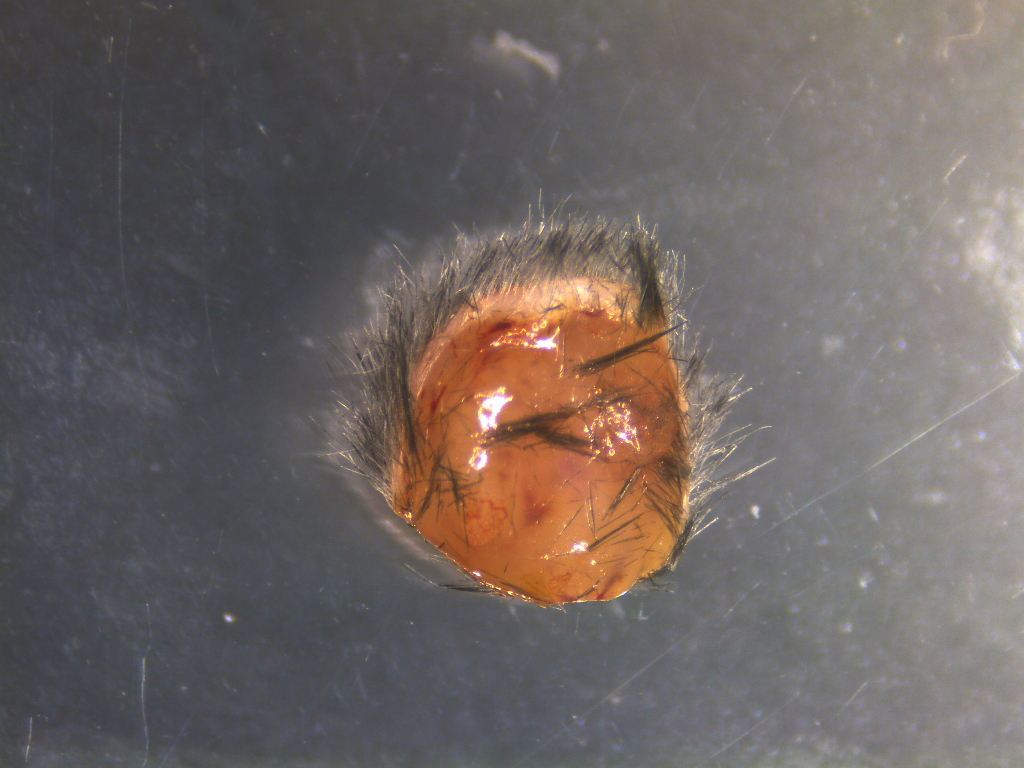

Supplement: Supplementary file 6 — Source data Fig. 6 [file 44318_2024_78_MOESM6_ESM.zip › Figure 6/6I/Vehicle+Fruquintinib-8.tif]

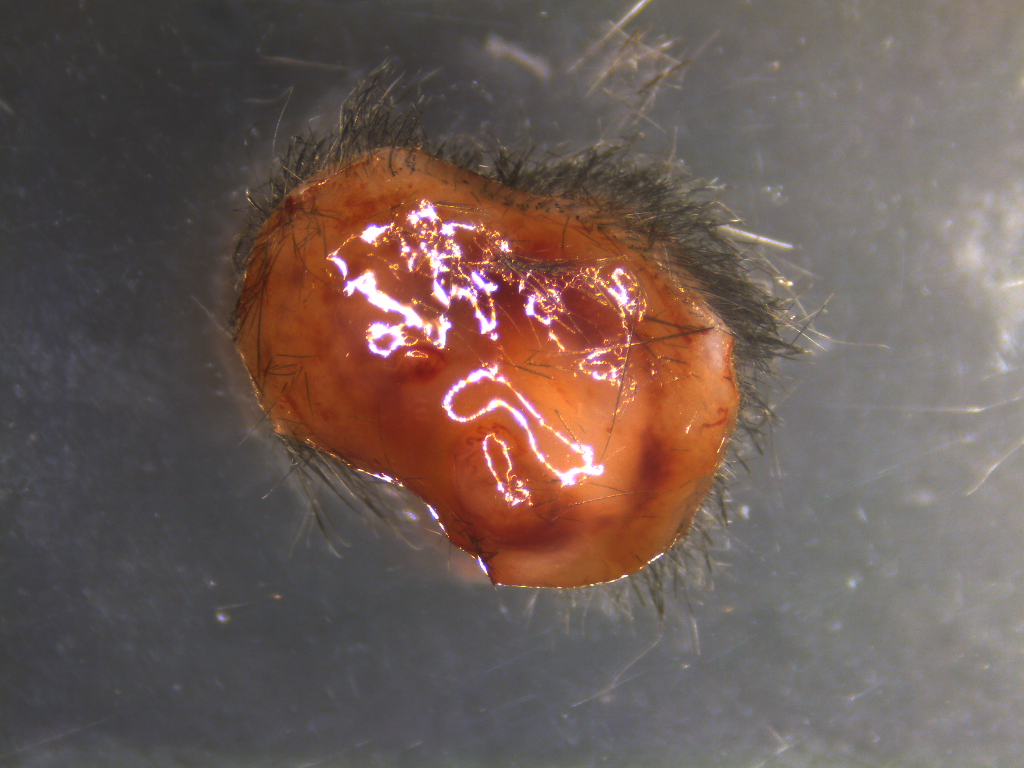

Supplement: Supplementary file 6 — Source data Fig. 6 [file 44318_2024_78_MOESM6_ESM.zip › Figure 6/6I/ODQ+Fruquintinib-1.tif]

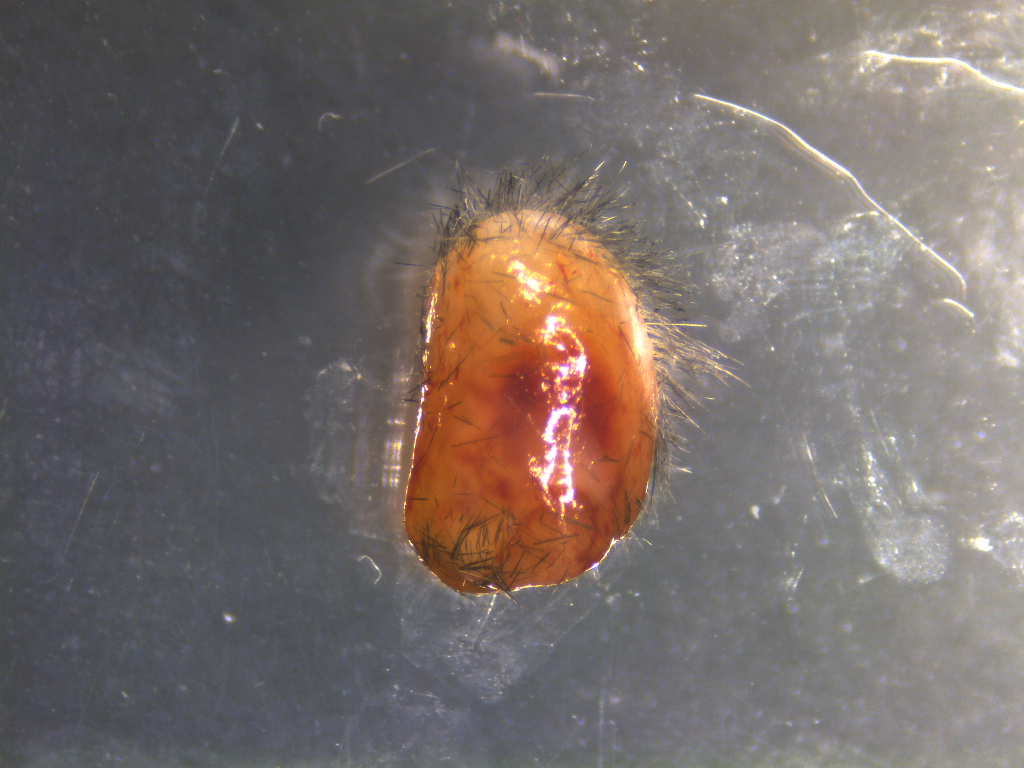

Supplement: Supplementary file 6 — Source data Fig. 6 [file 44318_2024_78_MOESM6_ESM.zip › Figure 6/6I/ODQ+Fruquintinib-5.tif]

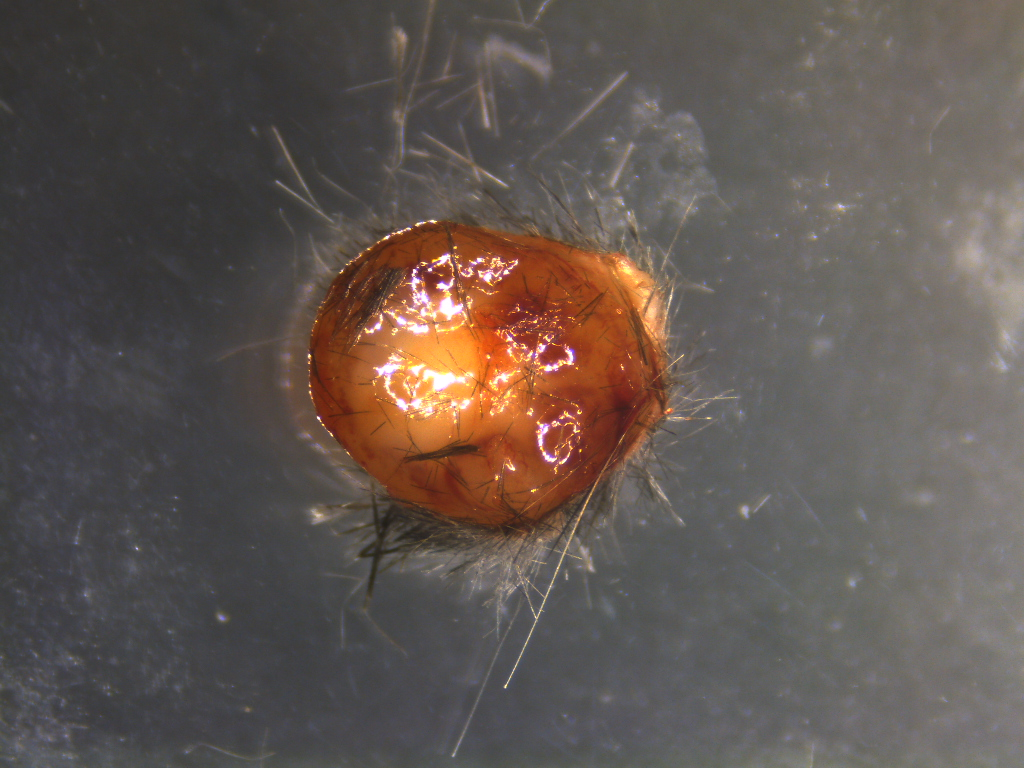

Supplement: Supplementary file 6 — Source data Fig. 6 [file 44318_2024_78_MOESM6_ESM.zip › Figure 6/6I/ODQ+Fruquintinib-4.tif]

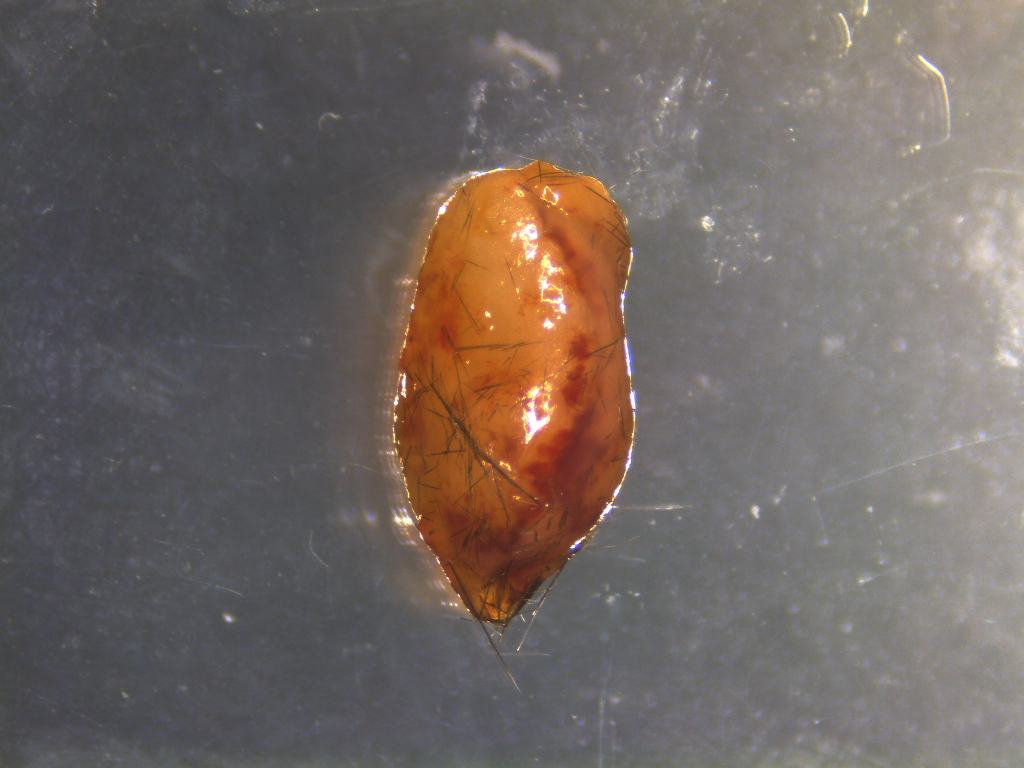

Supplement: Supplementary file 6 — Source data Fig. 6 [file 44318_2024_78_MOESM6_ESM.zip › Figure 6/6I/ODQ+Fruquintinib-6.tif]

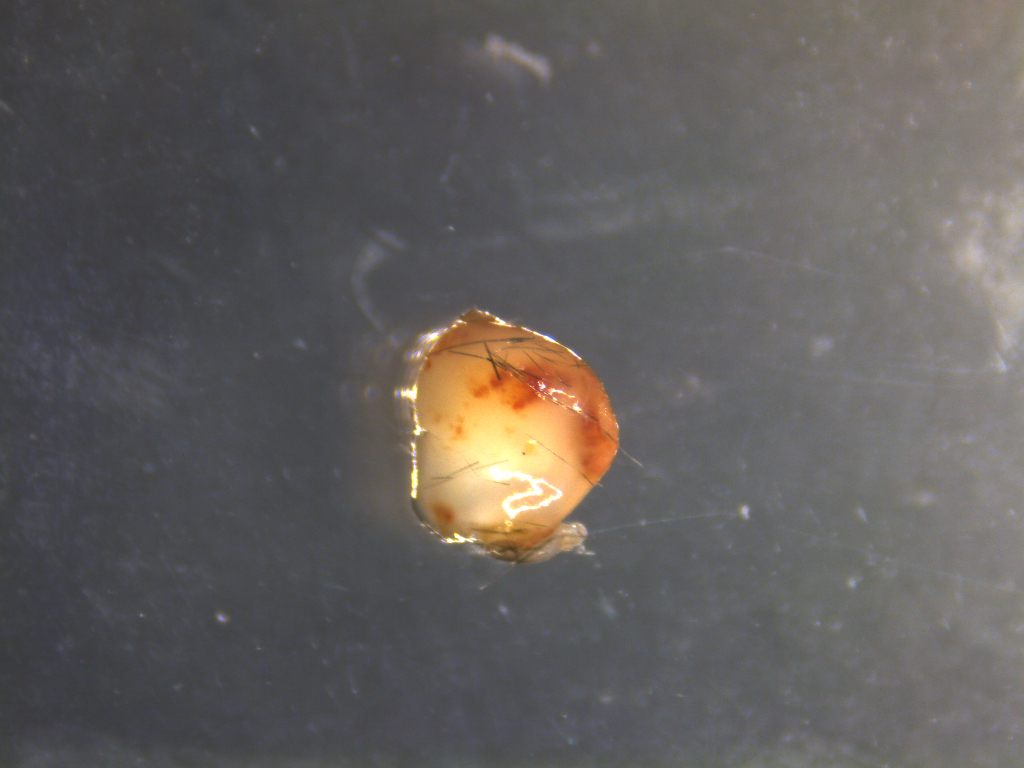

Supplement: Supplementary file 6 — Source data Fig. 6 [file 44318_2024_78_MOESM6_ESM.zip › Figure 6/6I/ODQ+Fruquintinib-7.tif]

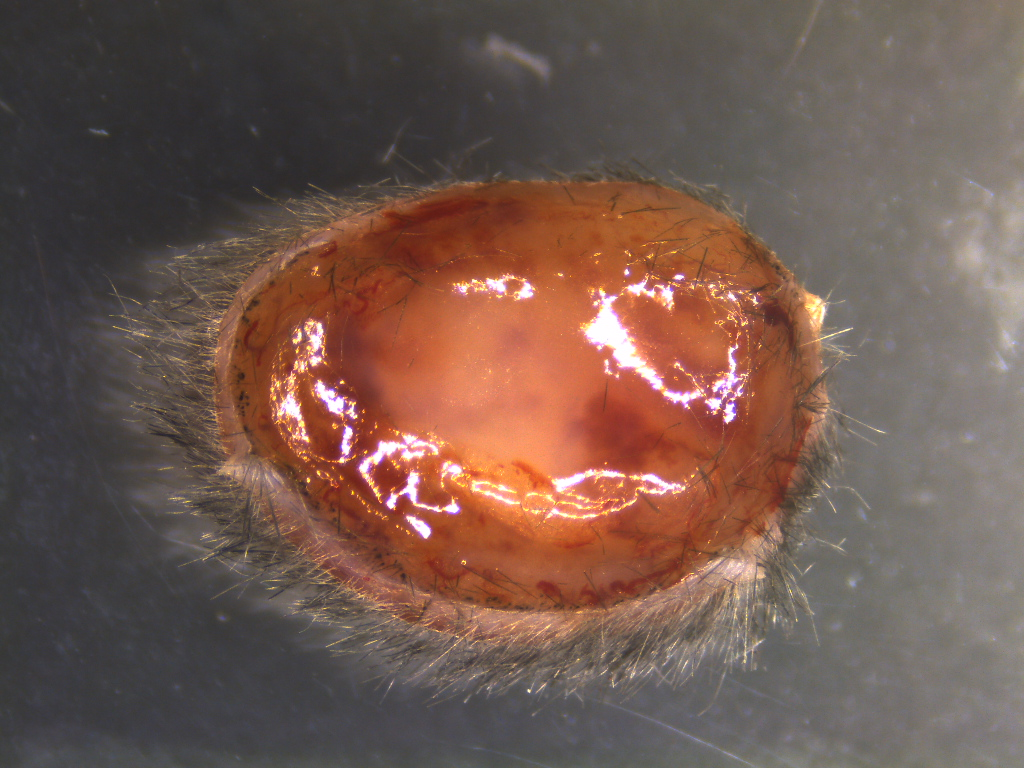

Supplement: Supplementary file 6 — Source data Fig. 6 [file 44318_2024_78_MOESM6_ESM.zip › Figure 6/6I/Vehicle+Fruquintinib-3.tif]

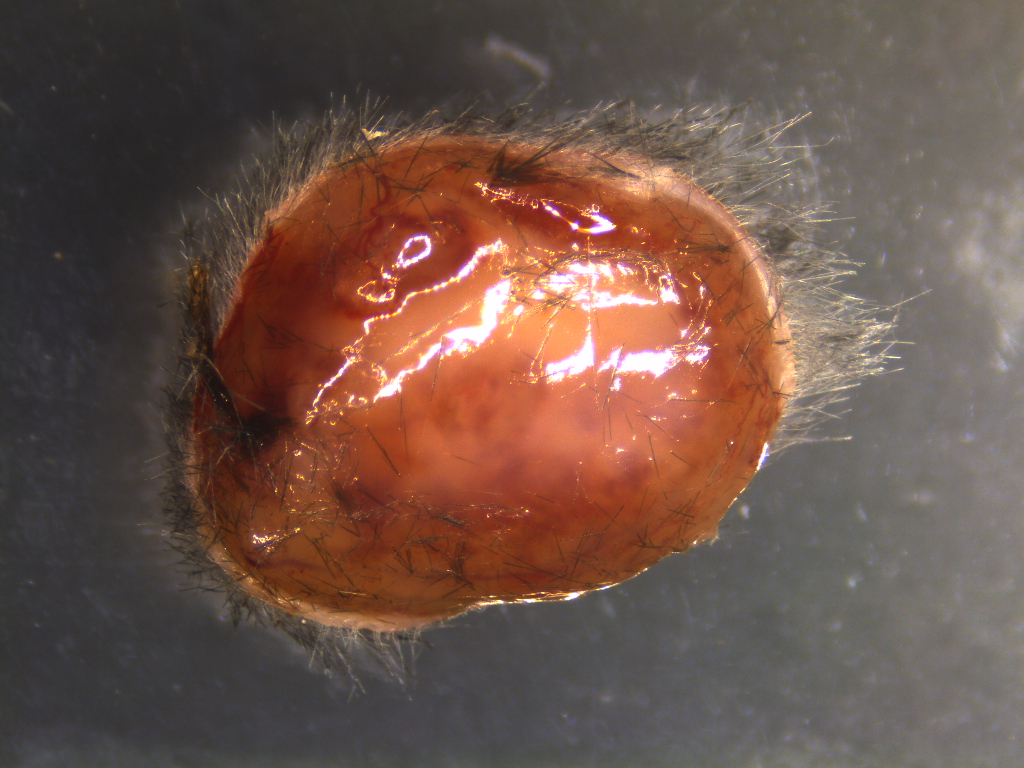

Supplement: Supplementary file 6 — Source data Fig. 6 [file 44318_2024_78_MOESM6_ESM.zip › Figure 6/6I/Vehicle+Fruquintinib-2.tif]

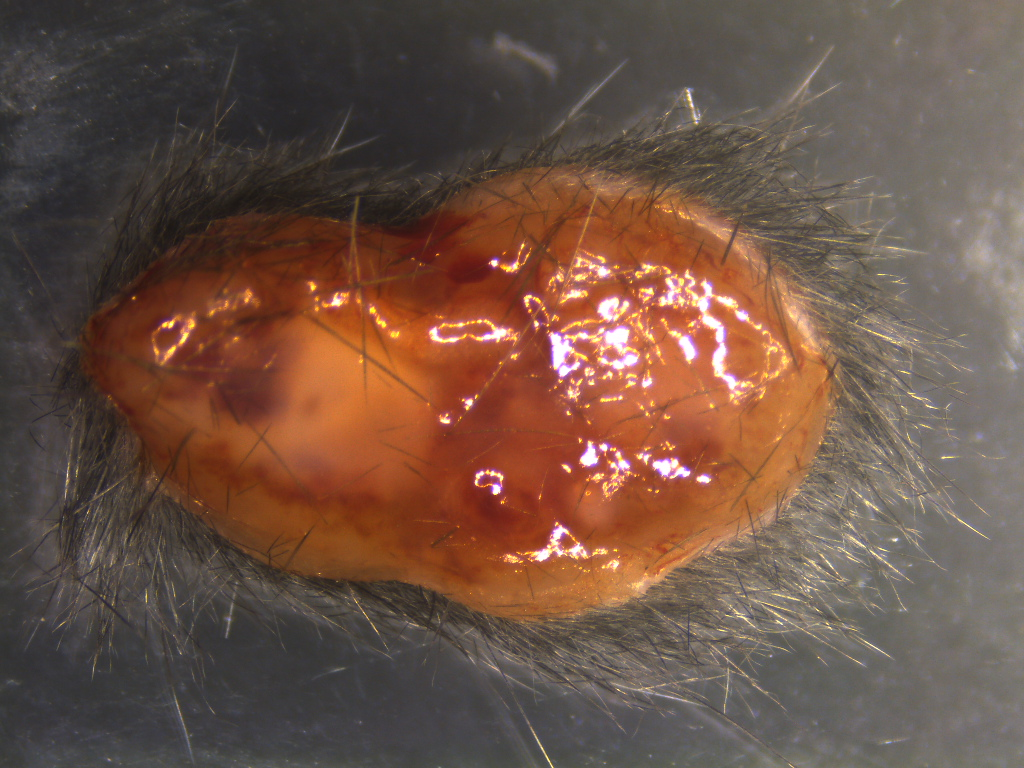

Supplement: Supplementary file 6 — Source data Fig. 6 [file 44318_2024_78_MOESM6_ESM.zip › Figure 6/6I/Vehicle+Fruquintinib-1.tif]

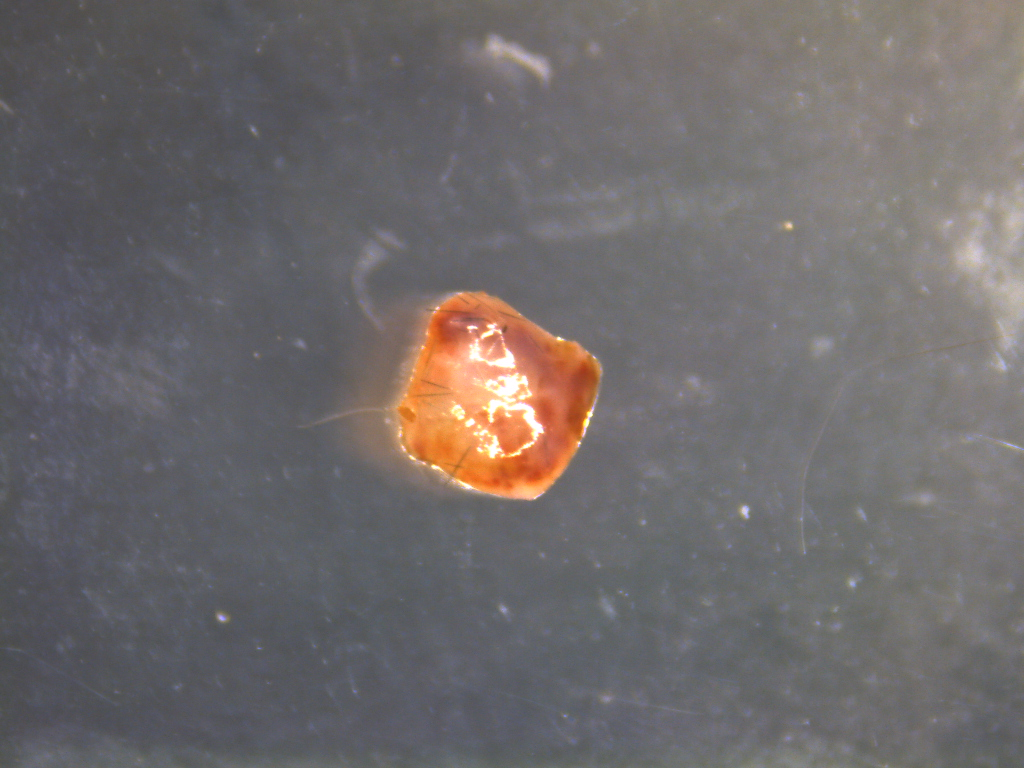

Supplement: Supplementary file 6 — Source data Fig. 6 [file 44318_2024_78_MOESM6_ESM.zip › Figure 6/6I/ODQ+Fruquintinib-8.tif]

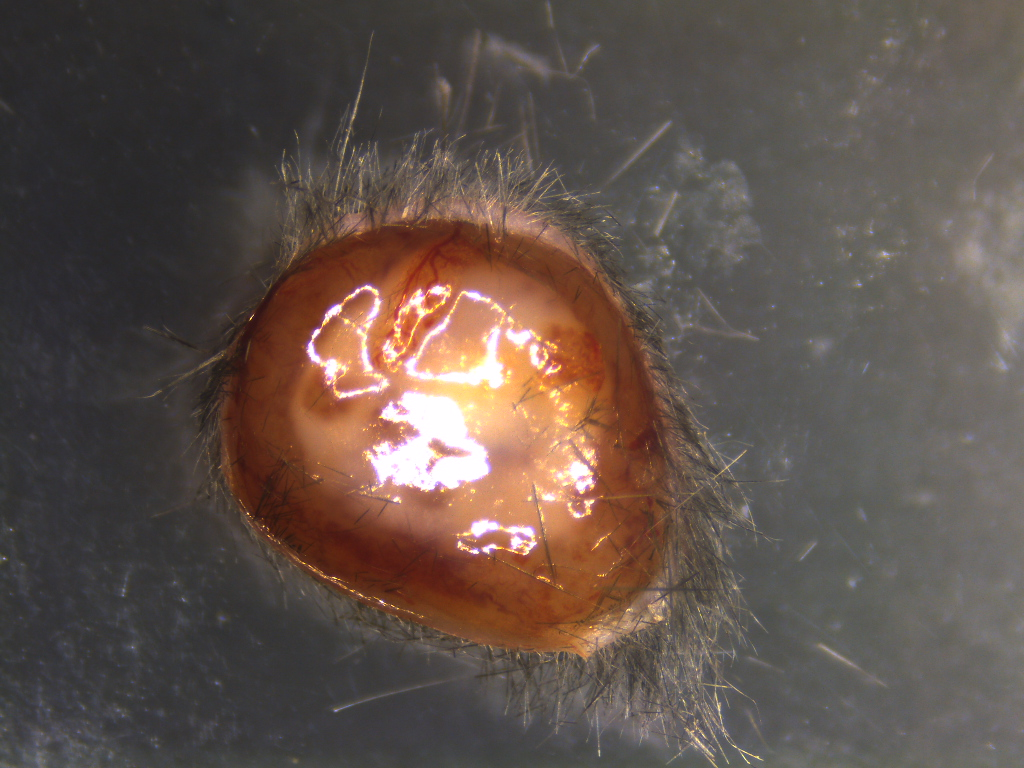

Supplement: Supplementary file 6 — Source data Fig. 6 [file 44318_2024_78_MOESM6_ESM.zip › Figure 6/6I/Vehicle+Fruquintinib-5.tif]

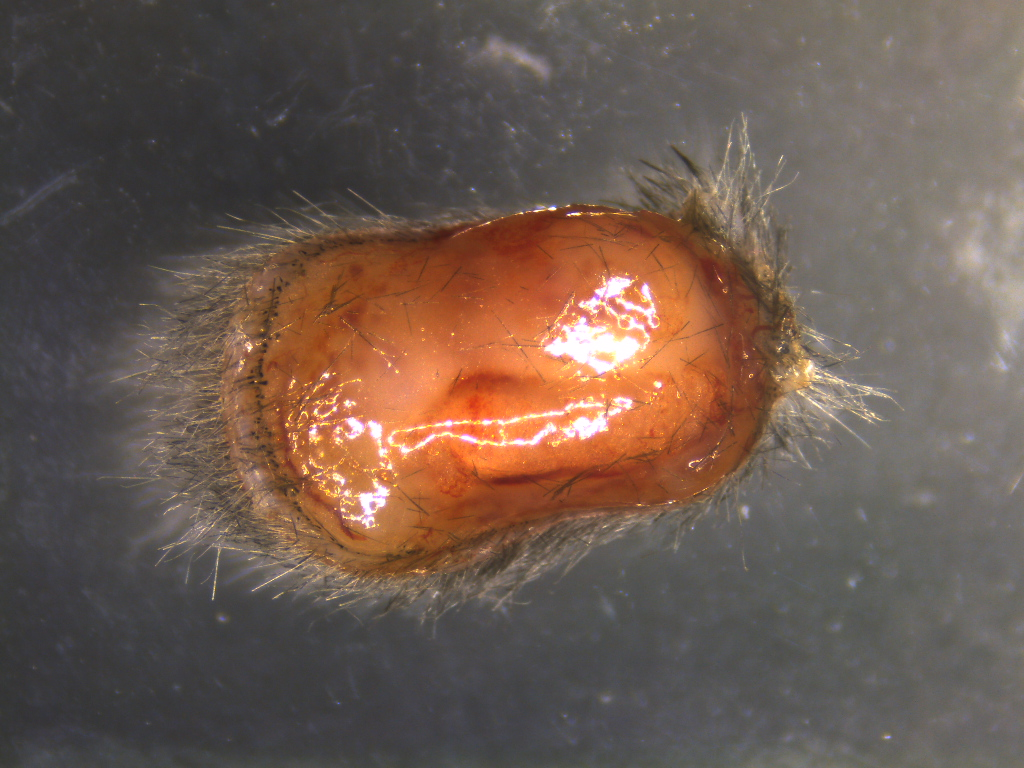

Supplement: Supplementary file 6 — Source data Fig. 6 [file 44318_2024_78_MOESM6_ESM.zip › Figure 6/6I/Vehicle+Fruquintinib-4.tif]

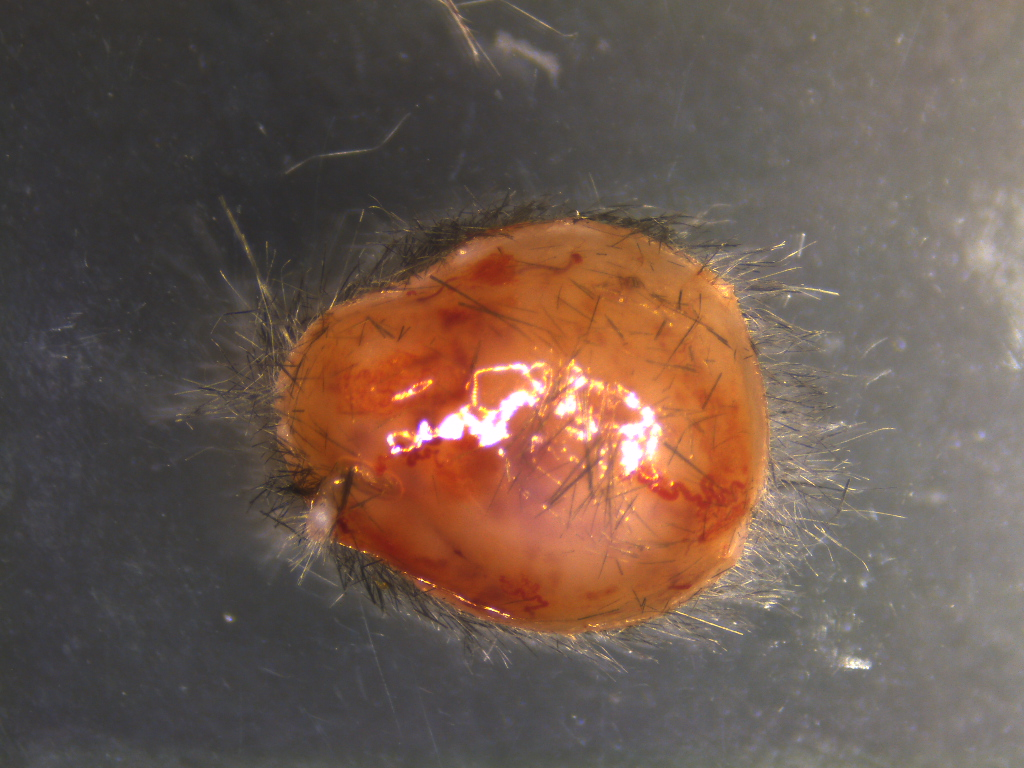

Supplement: Supplementary file 6 — Source data Fig. 6 [file 44318_2024_78_MOESM6_ESM.zip › Figure 6/6I/Vehicle+Fruquintinib-6.tif]

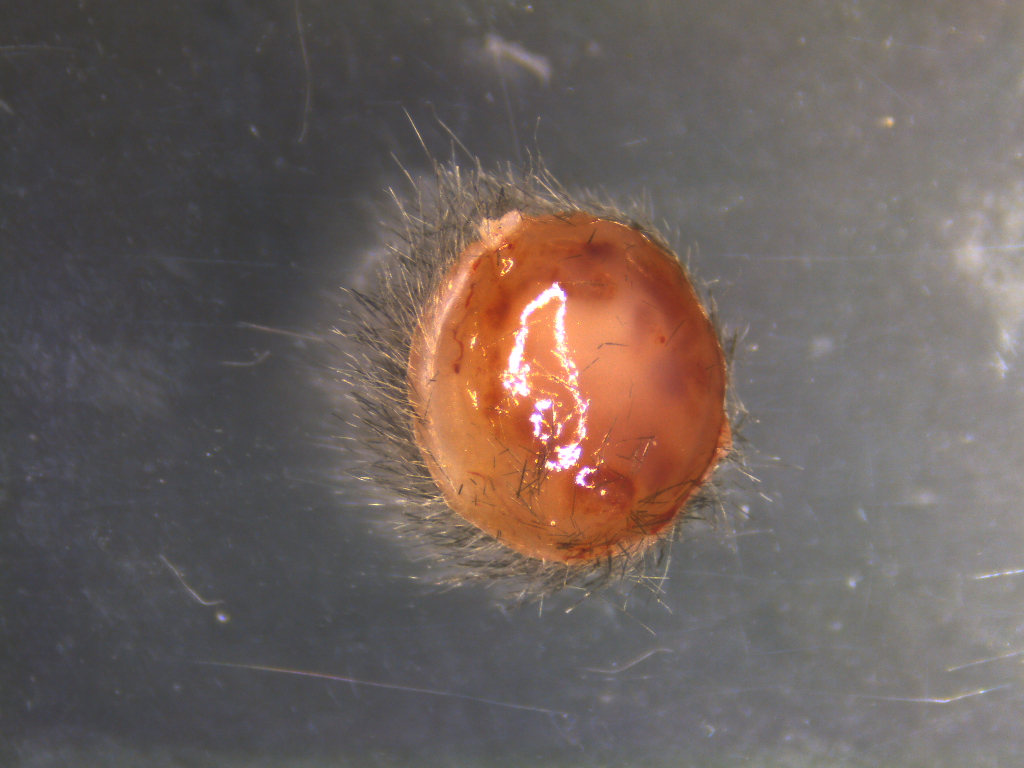

Supplement: Supplementary file 6 — Source data Fig. 6 [file 44318_2024_78_MOESM6_ESM.zip › Figure 6/6I/Vehicle+Fruquintinib-7.tif]
