# Supplementary figures and images for: Pericyte signaling via soluble guanylate cyclase shapes the vascular niche and microenvironment of tumors (part 2 of 4)
Source: EMBO J. 2024 Mar 25;43(8):7. doi: 10.1038/s44318-024-00078-5 (PMC11021551; doi:10.1038/s44318-024-00078-5)

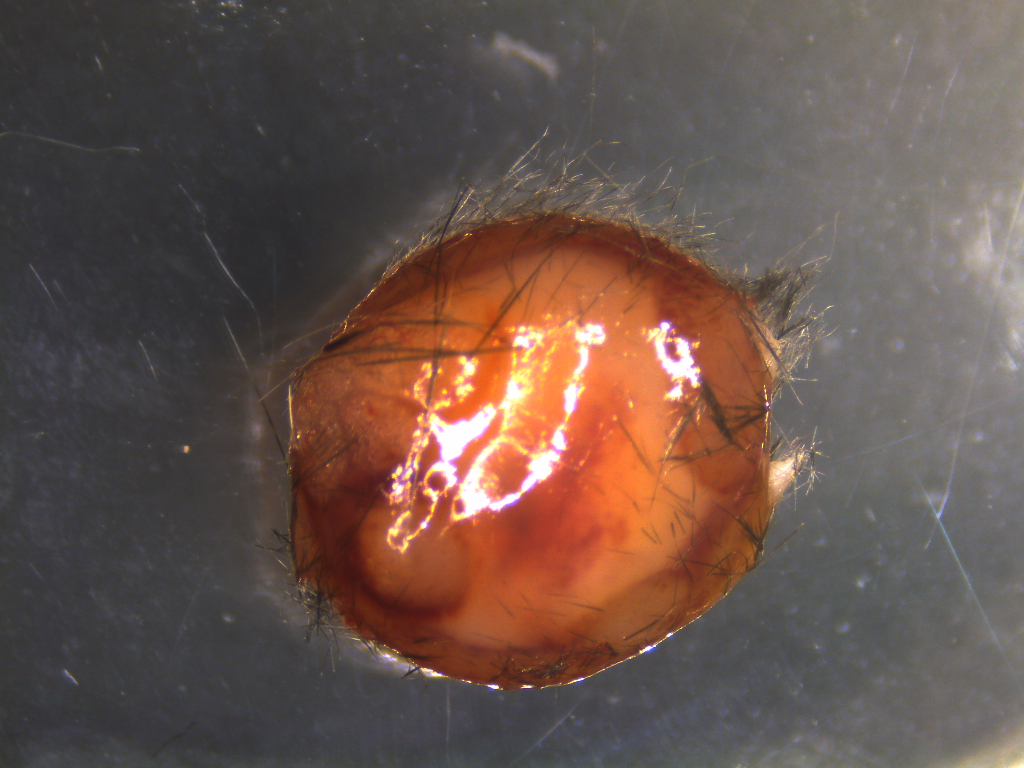

Supplement: Supplementary file 6 — Source data Fig. 6 [file 44318_2024_78_MOESM6_ESM.zip › Figure 6/6I/Vehicle+Vehicle-8.tif]

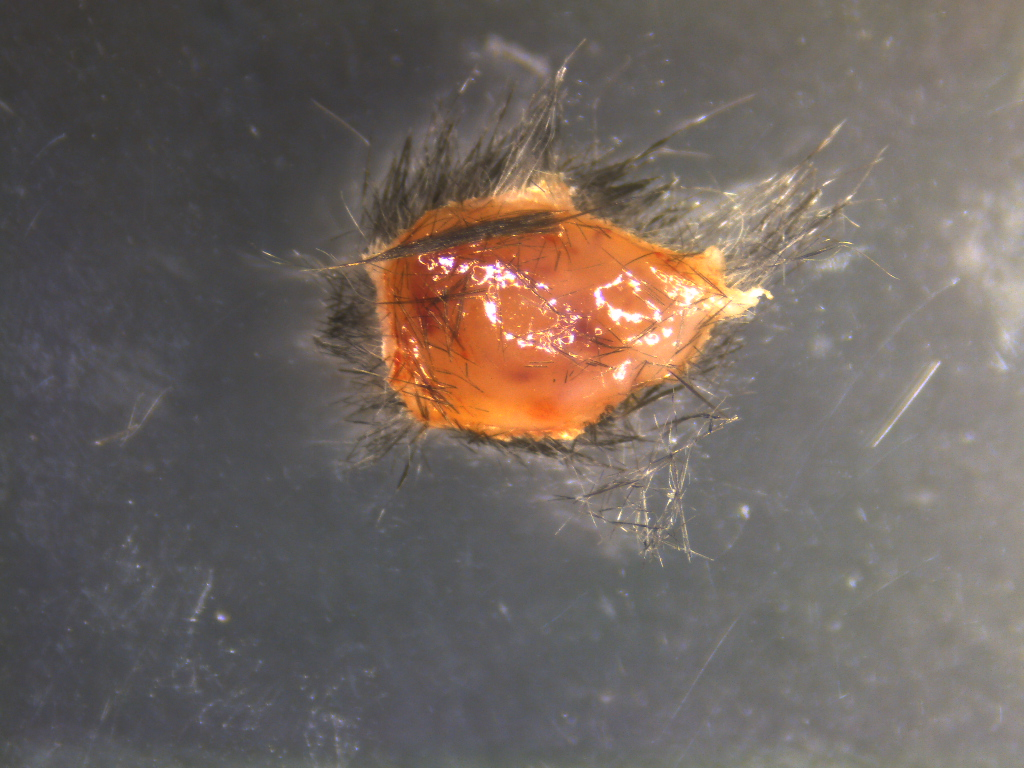

Supplement: Supplementary file 6 — Source data Fig. 6 [file 44318_2024_78_MOESM6_ESM.zip › Figure 6/6I/ODQ+Vehicle8.tif]

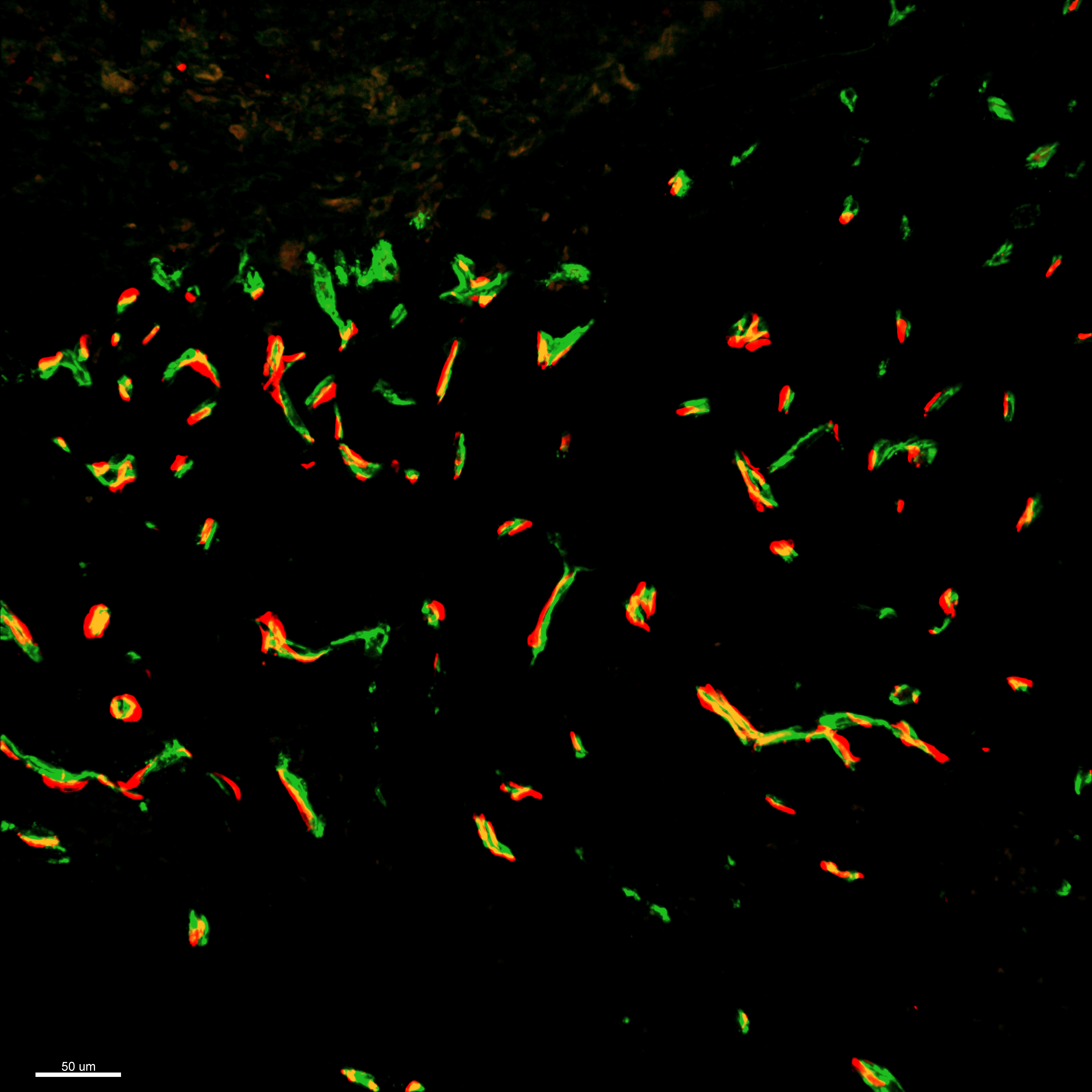

Supplement: Supplementary file 6 — Source data Fig. 6 [file 44318_2024_78_MOESM6_ESM.zip › Figure 6/6E/sGCCtr+Fruquintinib-Desmin.tif]

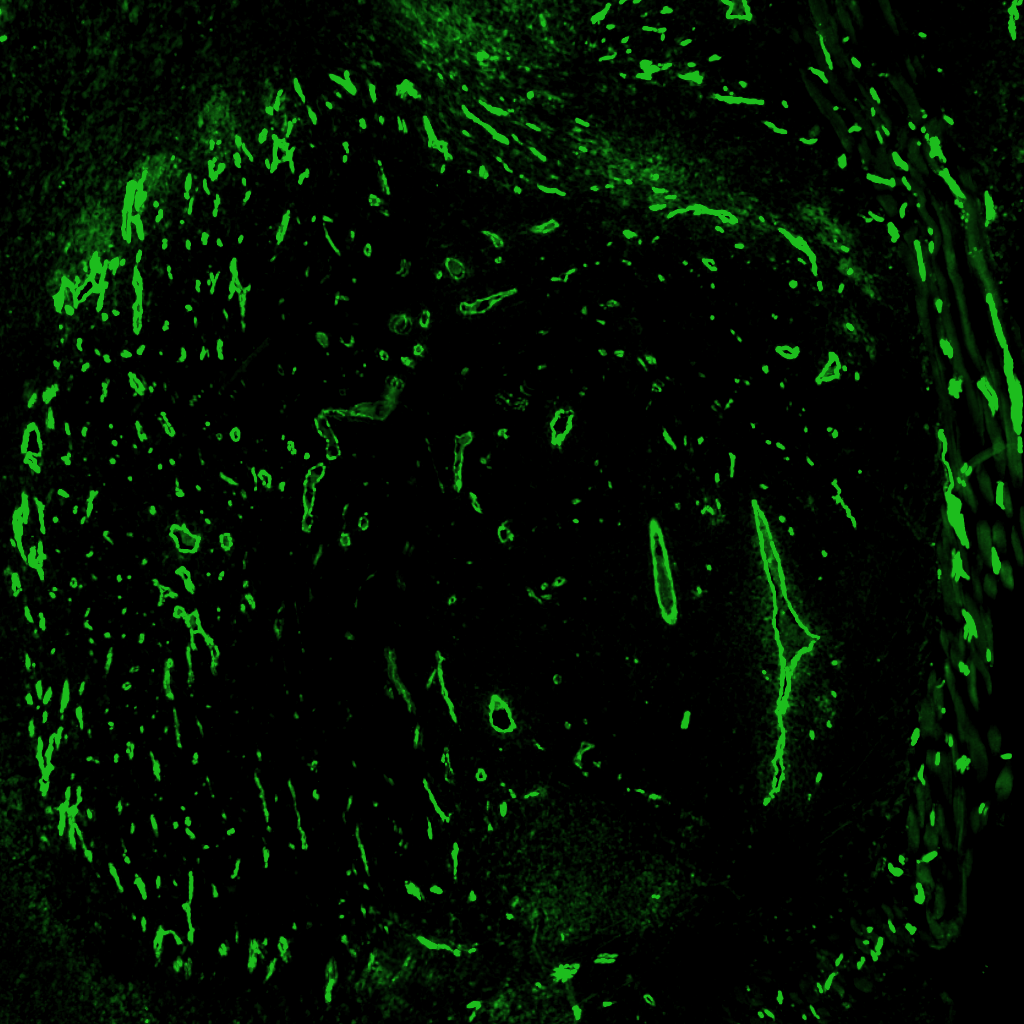

Supplement: Supplementary file 6 — Source data Fig. 6 [file 44318_2024_78_MOESM6_ESM.zip › Figure 6/6E/sGCCtr+Vehicle-CD31-Zoom in.tif]

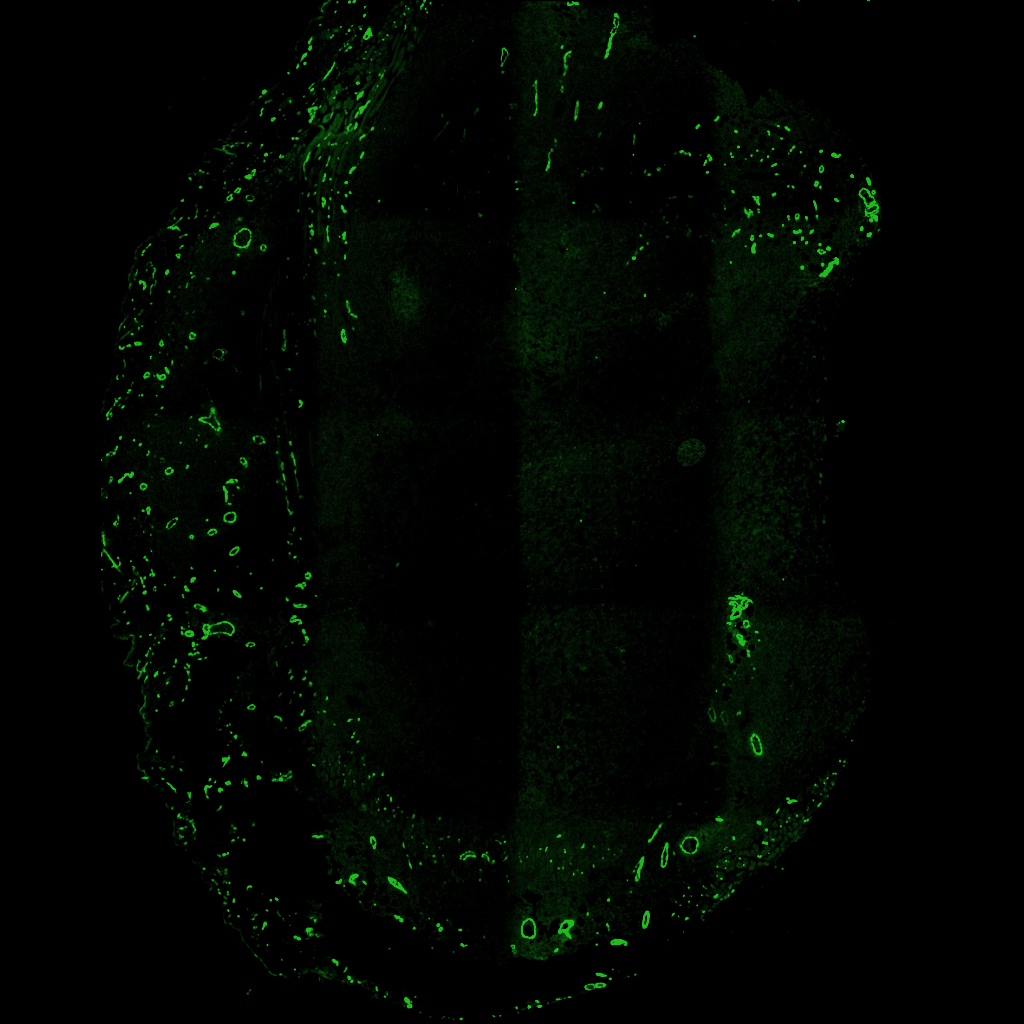

Supplement: Supplementary file 6 — Source data Fig. 6 [file 44318_2024_78_MOESM6_ESM.zip › Figure 6/6E/sGC╬öpc+Fruquintinib-CD31.tif]

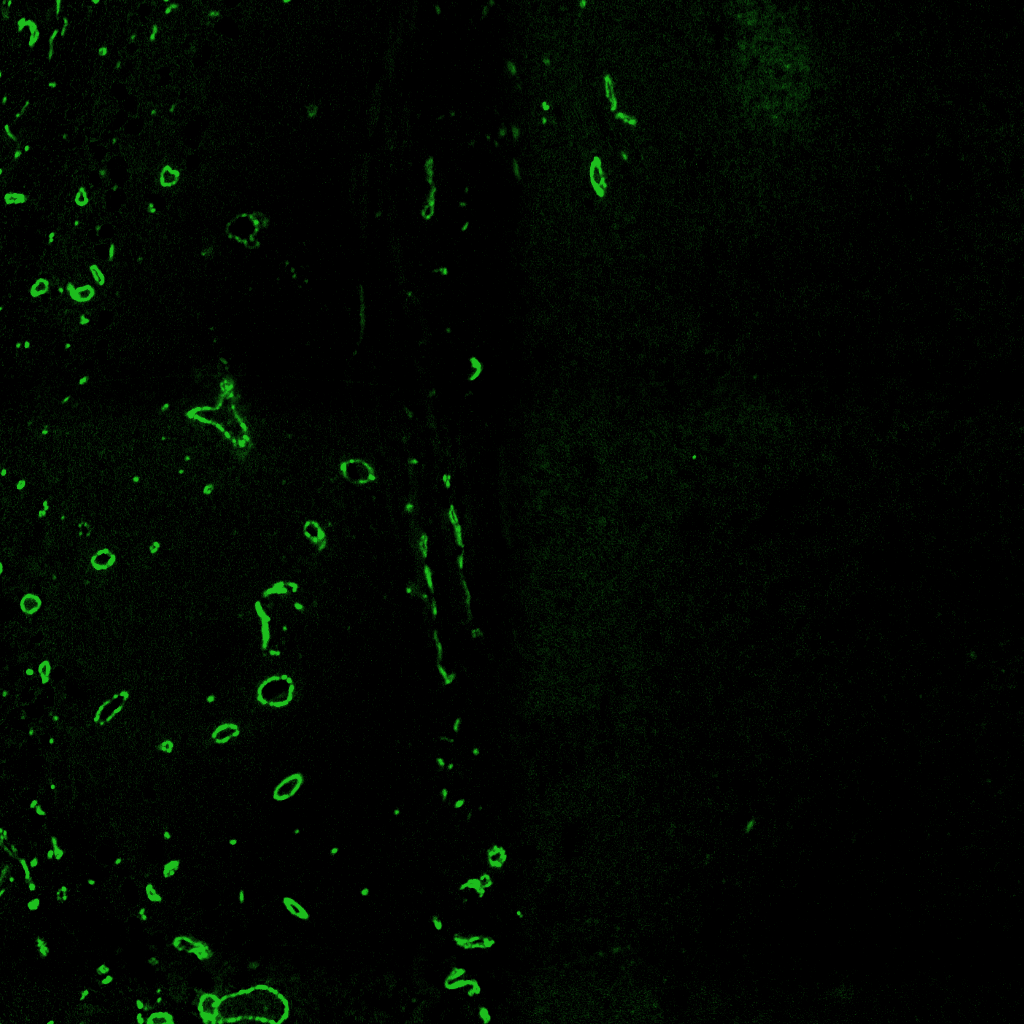

Supplement: Supplementary file 6 — Source data Fig. 6 [file 44318_2024_78_MOESM6_ESM.zip › Figure 6/6E/sGC╬öpc+Fruquintinib-CD31-Zoom in.tif]

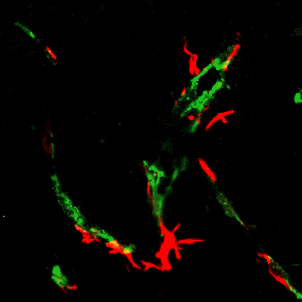

Supplement: Supplementary file 6 — Source data Fig. 6 [file 44318_2024_78_MOESM6_ESM.zip › Figure 6/6E/sGC╬öpc+Vehicle-Desmin-Zoom in.tif]

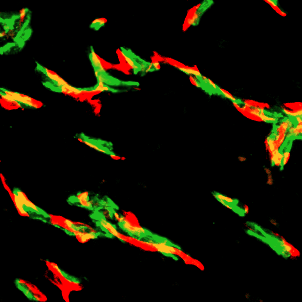

Supplement: Supplementary file 6 — Source data Fig. 6 [file 44318_2024_78_MOESM6_ESM.zip › Figure 6/6E/sGCCtr+Vehicle-Desmin-Zoom in.tif]

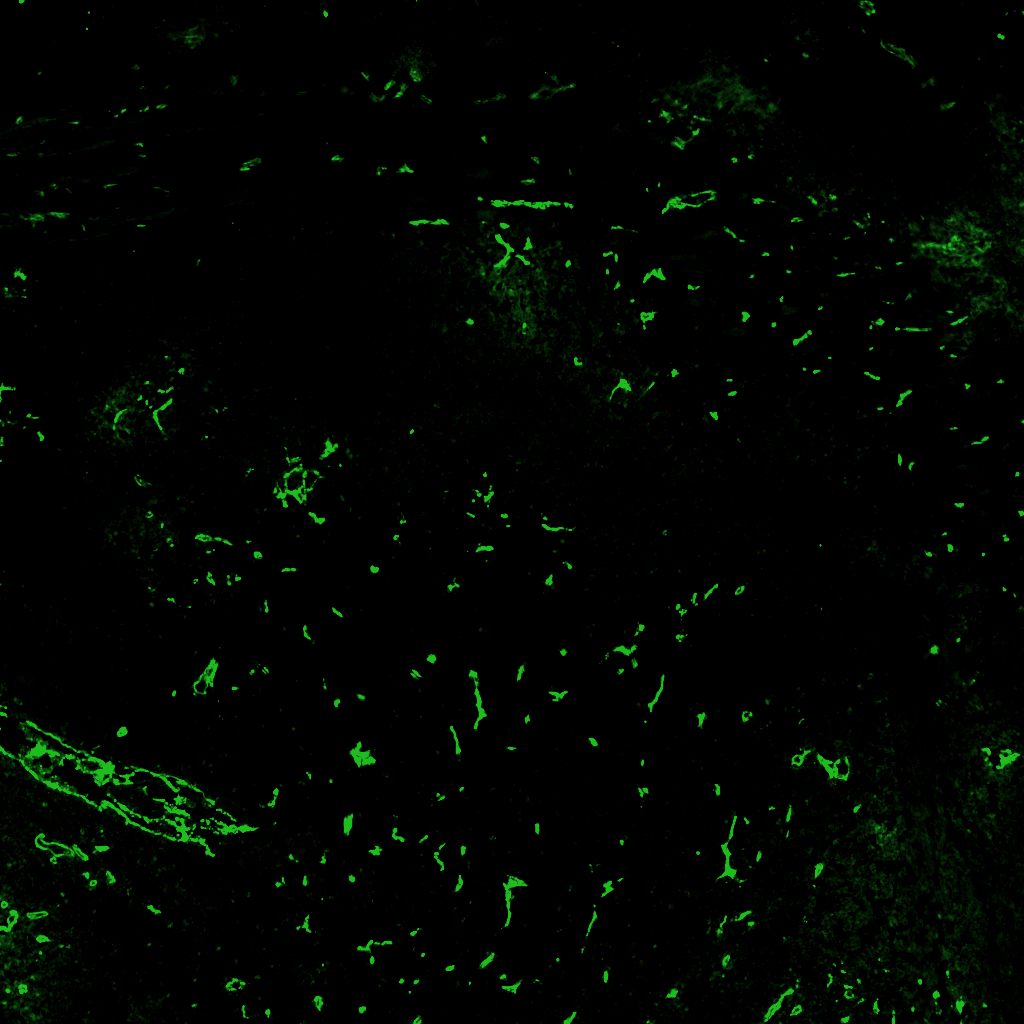

Supplement: Supplementary file 6 — Source data Fig. 6 [file 44318_2024_78_MOESM6_ESM.zip › Figure 6/6E/sGCCtr+Fruquintinib-CD31-Zoom in.tif]

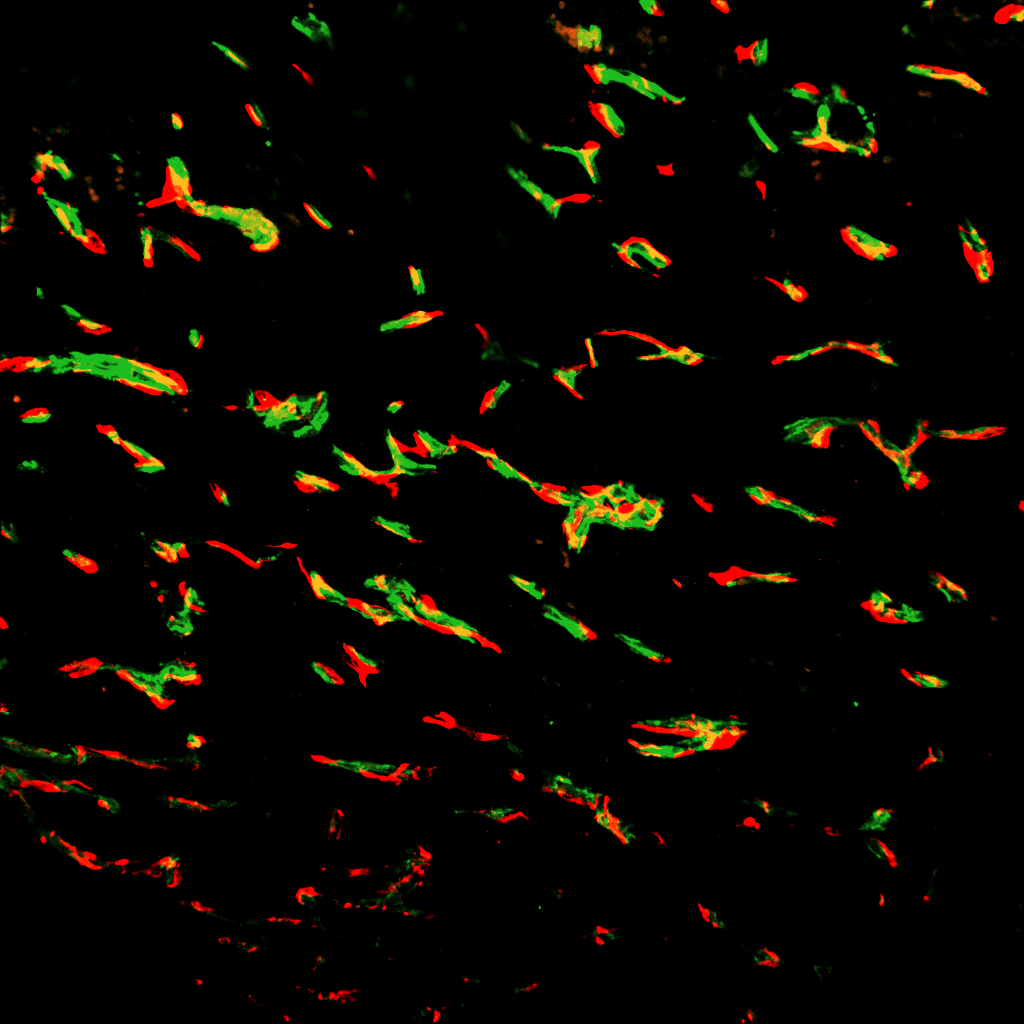

Supplement: Supplementary file 6 — Source data Fig. 6 [file 44318_2024_78_MOESM6_ESM.zip › Figure 6/6E/sGCCtr+Vehicle-Desmin.tif]

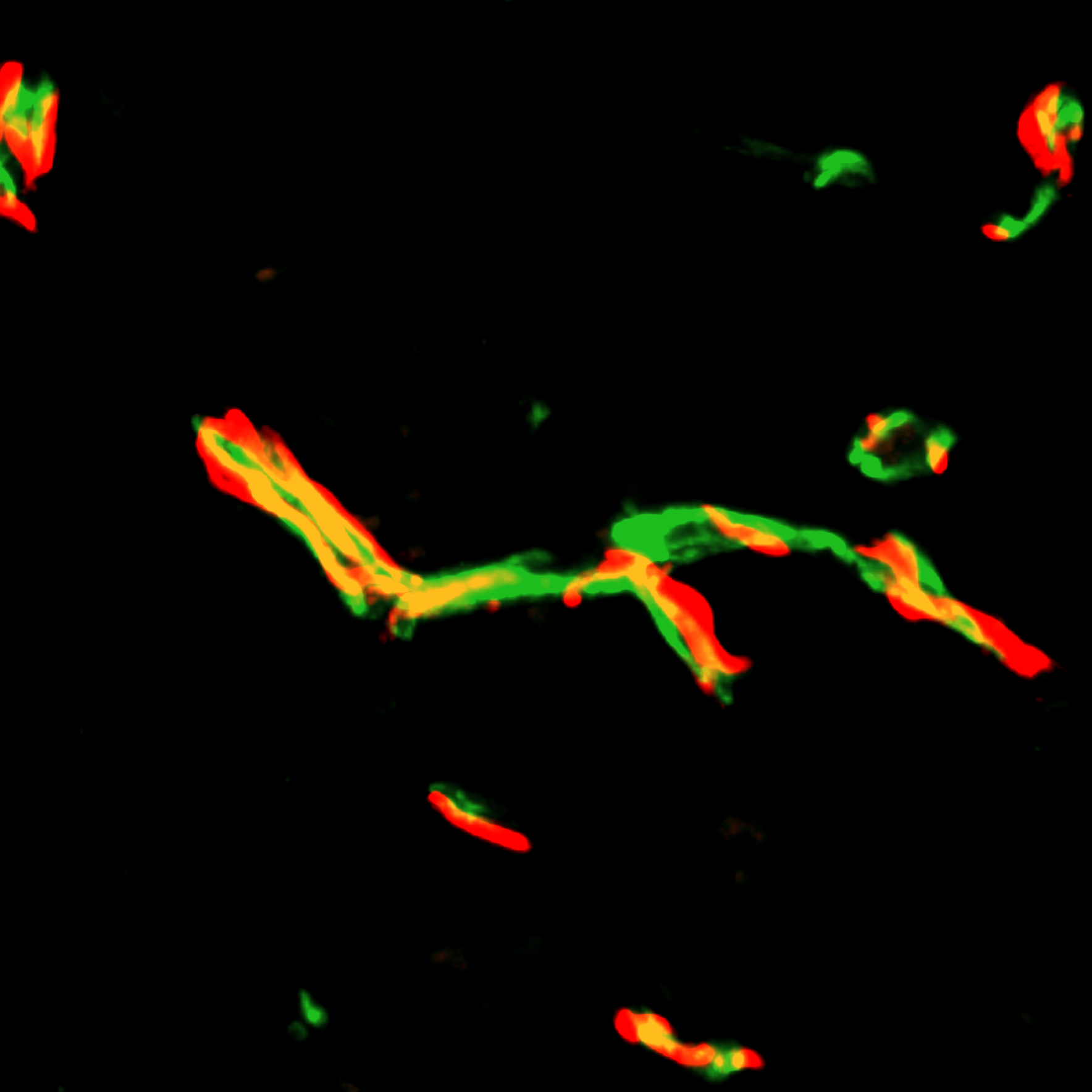

Supplement: Supplementary file 6 — Source data Fig. 6 [file 44318_2024_78_MOESM6_ESM.zip › Figure 6/6E/sGCCtr+Fruquintinib-Desmin-Zoom in.tif]

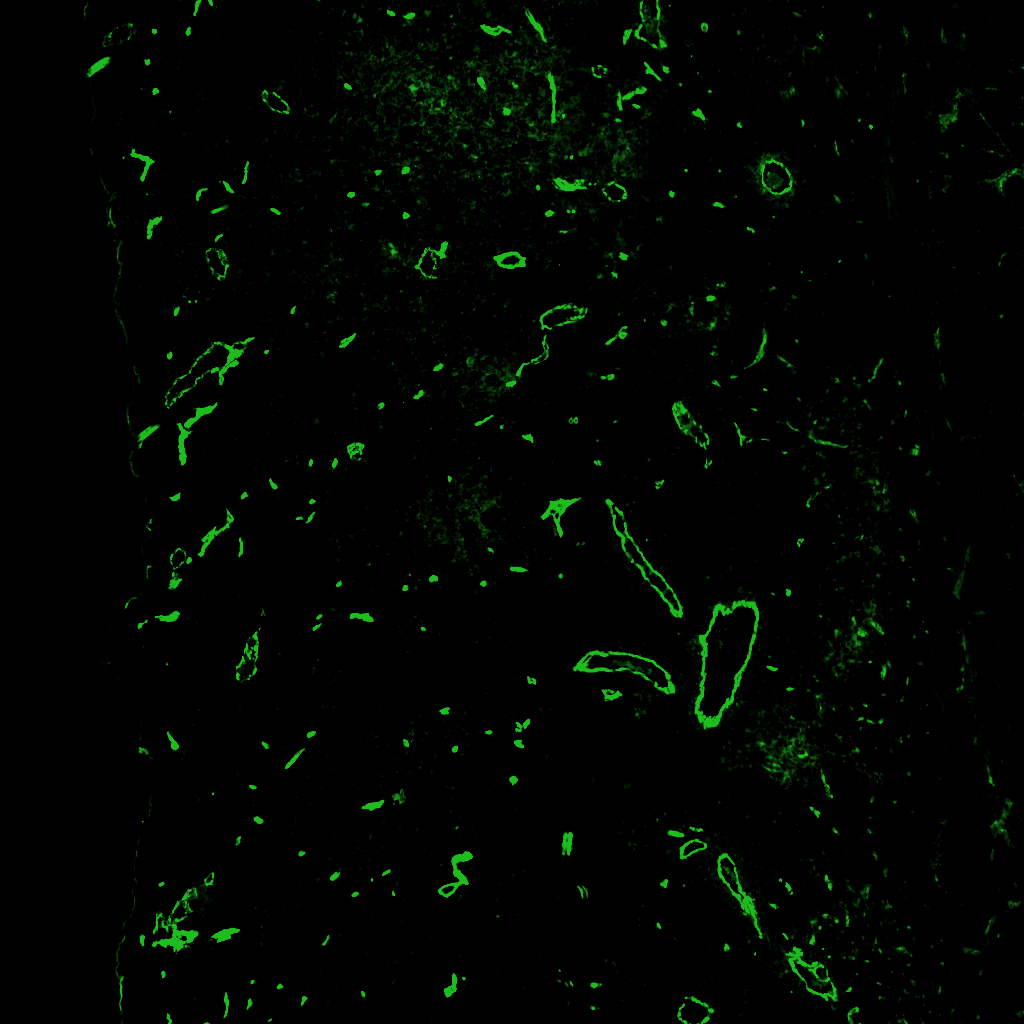

Supplement: Supplementary file 6 — Source data Fig. 6 [file 44318_2024_78_MOESM6_ESM.zip › Figure 6/6E/sGC╬öpc+Vehicle-CD31-Zoom in.tif]

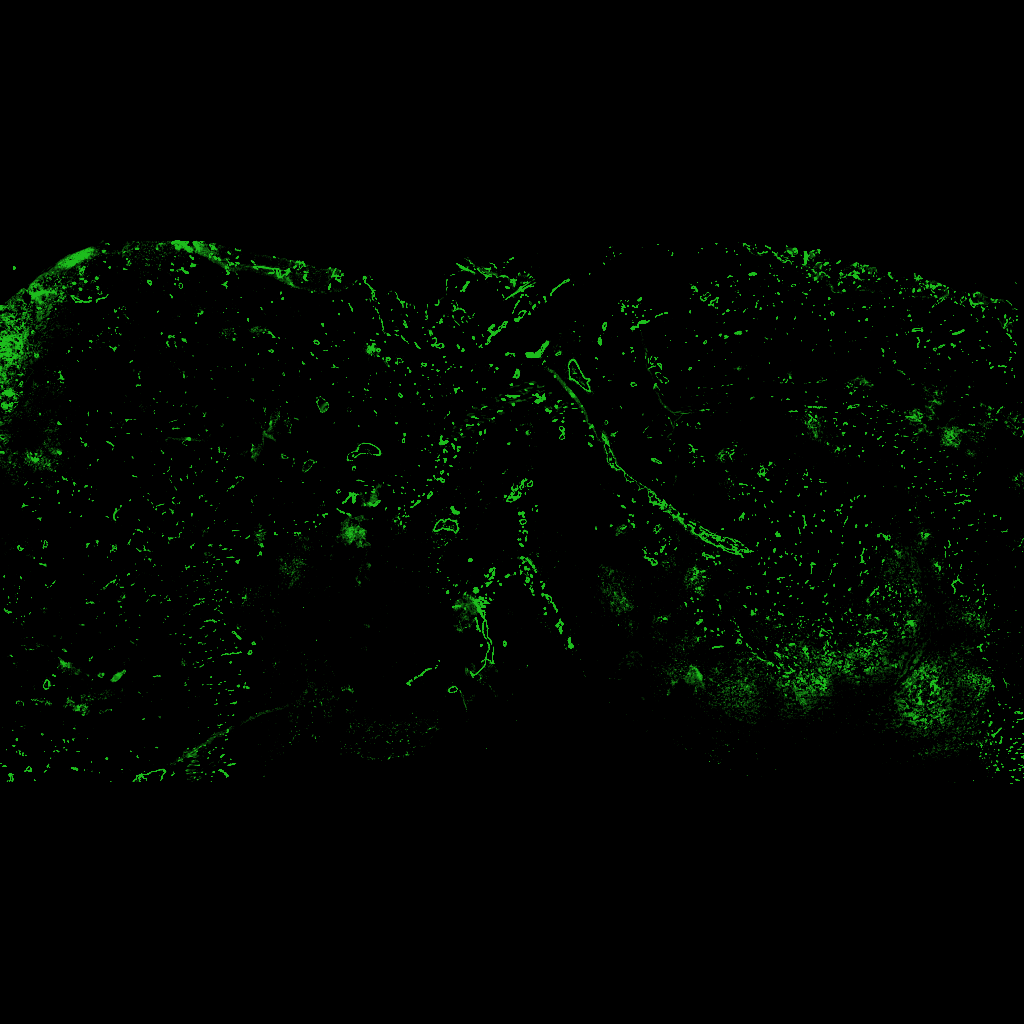

Supplement: Supplementary file 6 — Source data Fig. 6 [file 44318_2024_78_MOESM6_ESM.zip › Figure 6/6E/sGCCtr+Fruquintinib-CD31.tif]

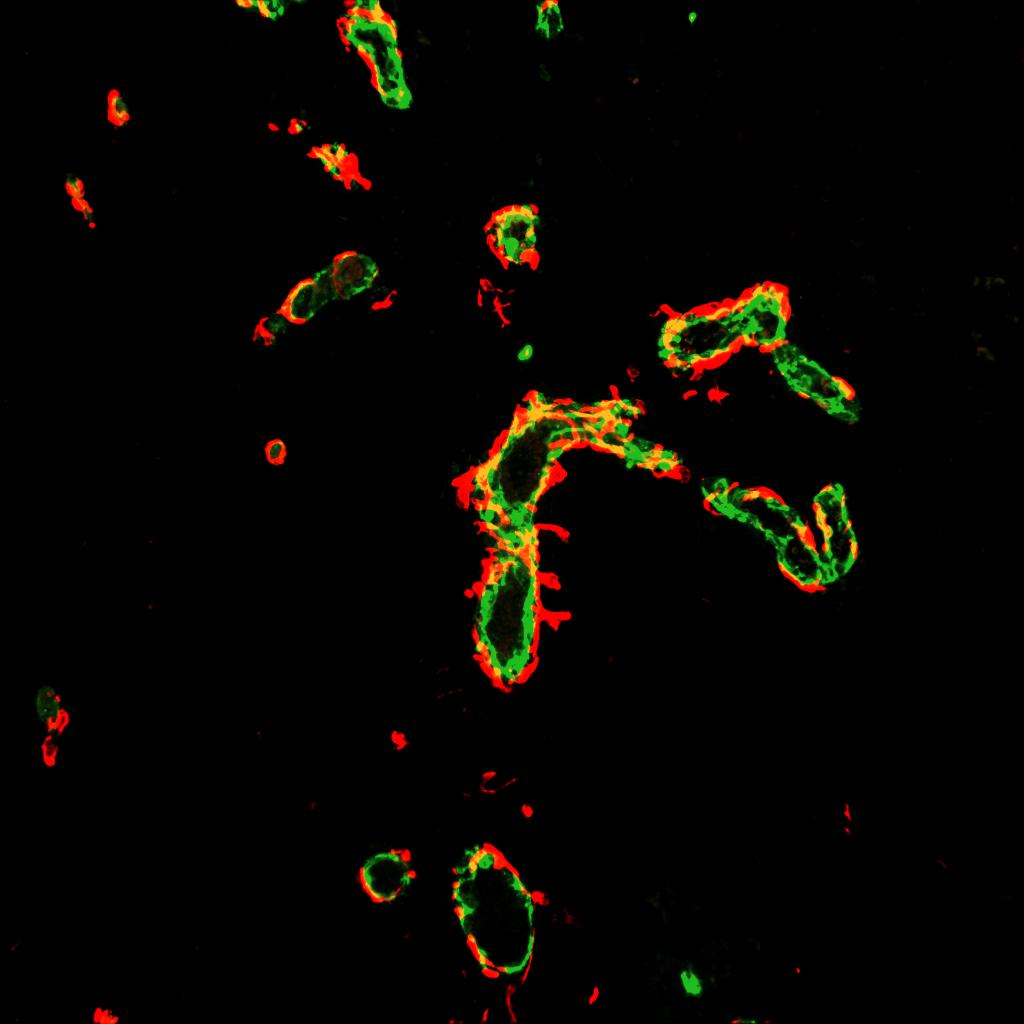

Supplement: Supplementary file 6 — Source data Fig. 6 [file 44318_2024_78_MOESM6_ESM.zip › Figure 6/6E/sGC╬öpc+Fruquintinib-Desmin.tif]

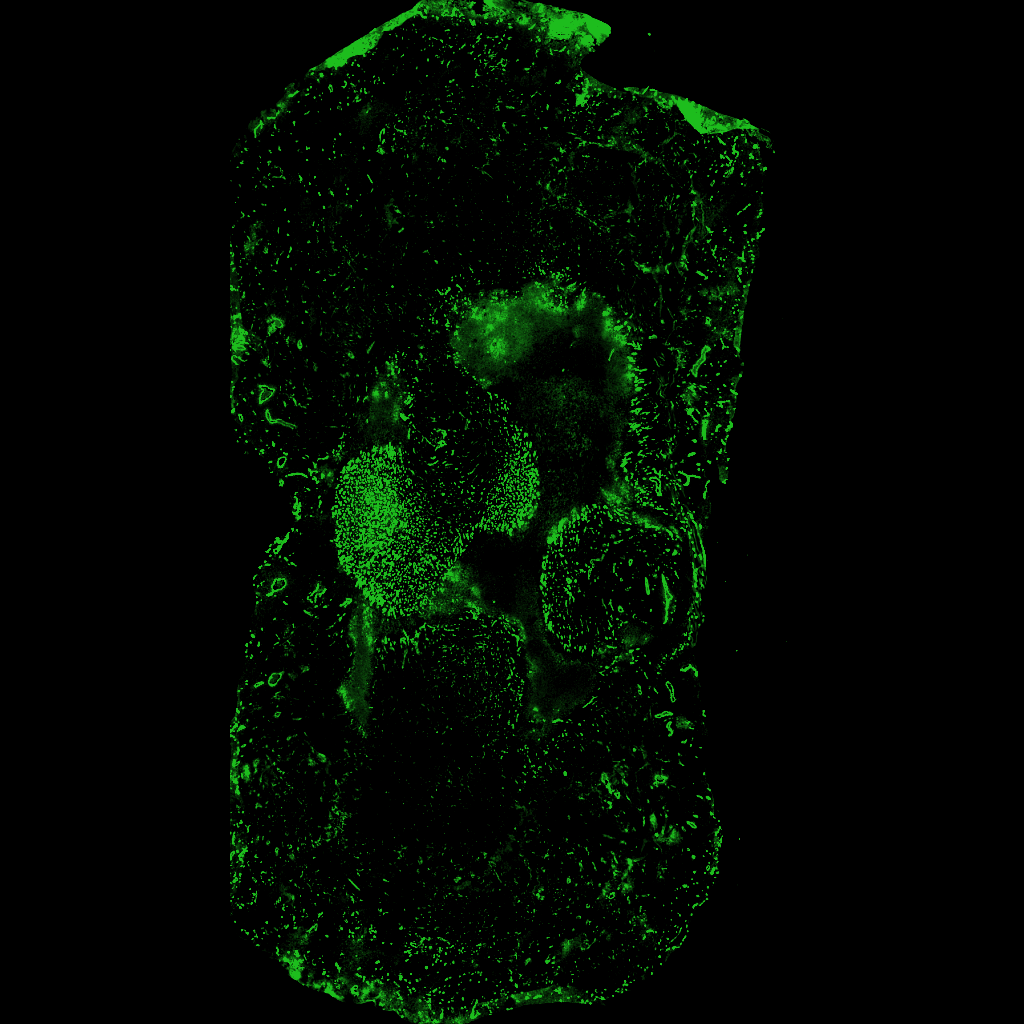

Supplement: Supplementary file 6 — Source data Fig. 6 [file 44318_2024_78_MOESM6_ESM.zip › Figure 6/6E/sGCCtr+Vehicle-CD31.tif]

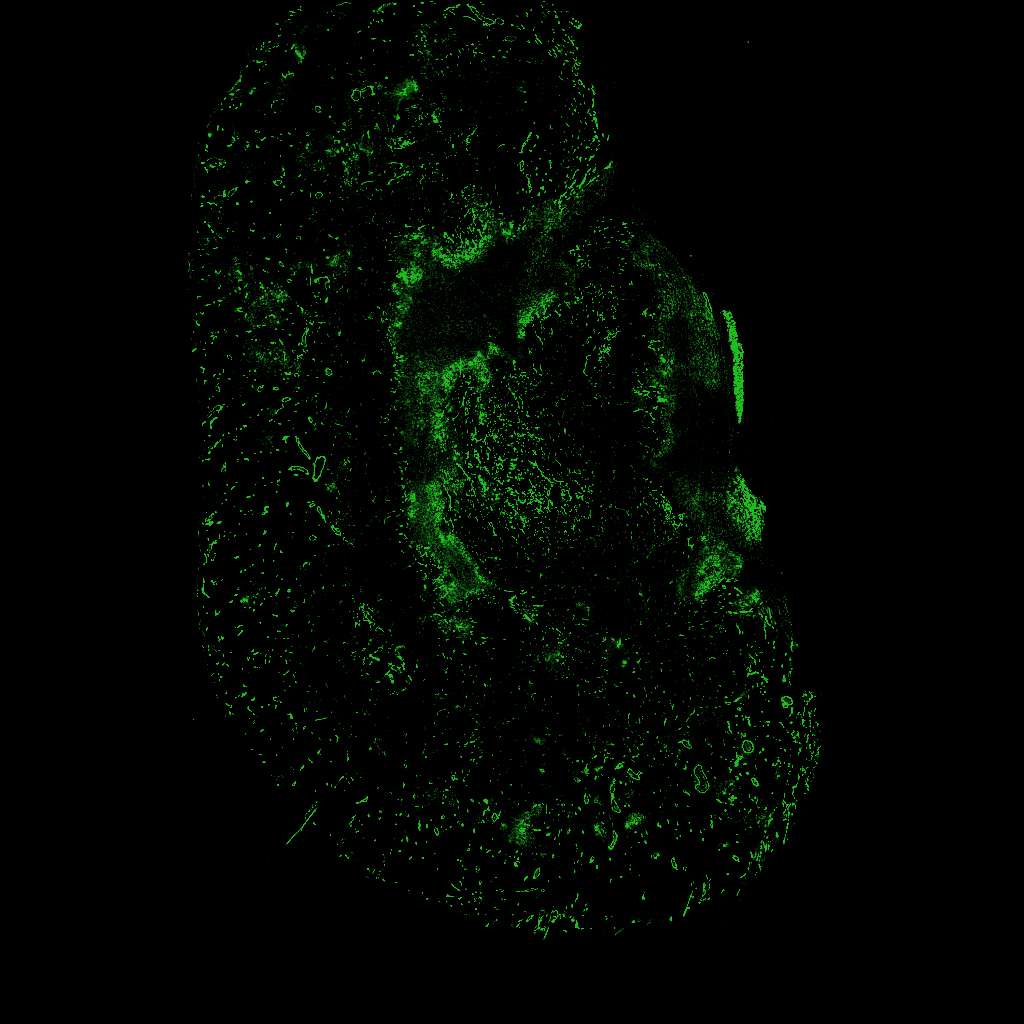

Supplement: Supplementary file 6 — Source data Fig. 6 [file 44318_2024_78_MOESM6_ESM.zip › Figure 6/6E/sGC╬öpc+Vehicle-CD31.tif]

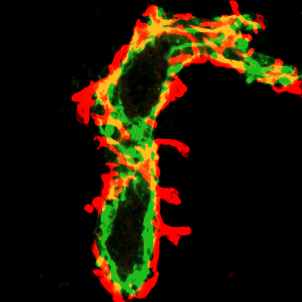

Supplement: Supplementary file 6 — Source data Fig. 6 [file 44318_2024_78_MOESM6_ESM.zip › Figure 6/6E/sGC╬öpc+Fruquintinib-Desmin-Zoom in.tif]

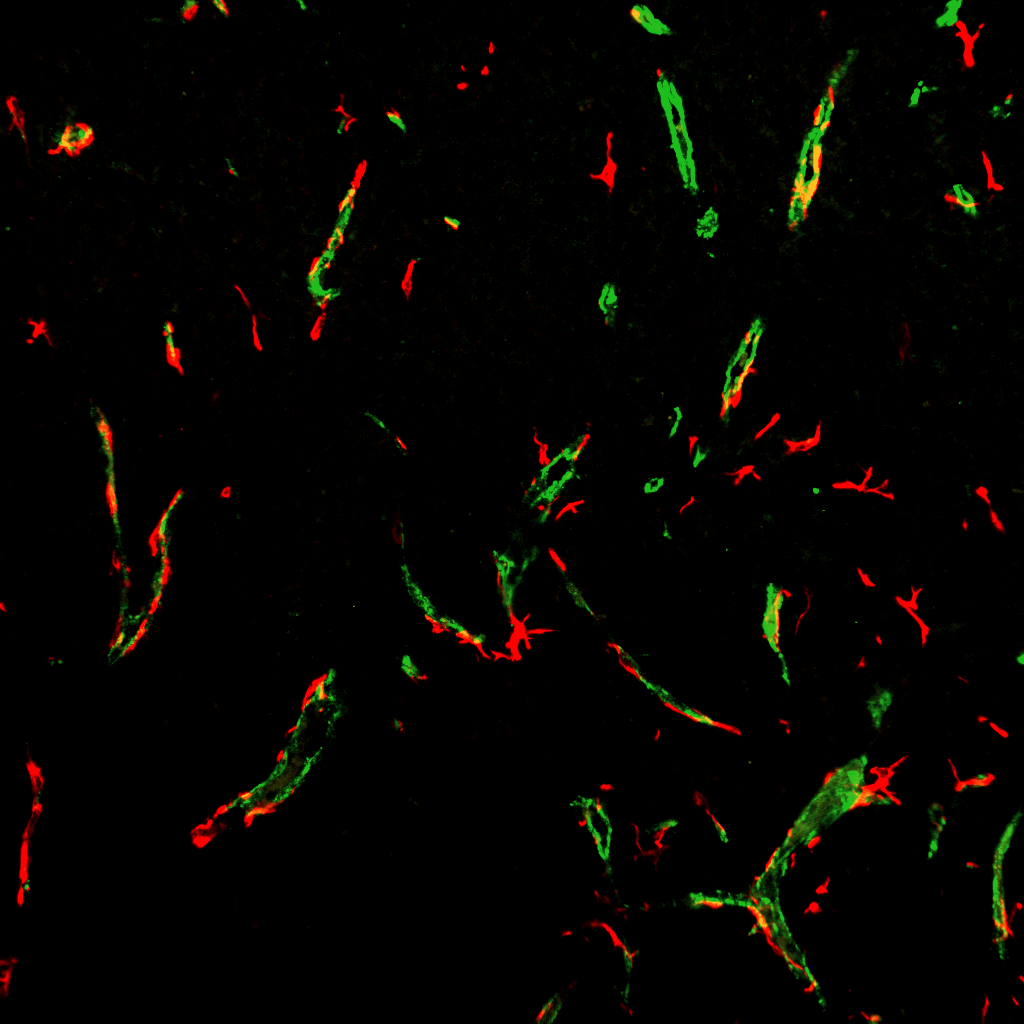

Supplement: Supplementary file 6 — Source data Fig. 6 [file 44318_2024_78_MOESM6_ESM.zip › Figure 6/6E/sGC╬öpc+Vehicle-Desmin.tif]

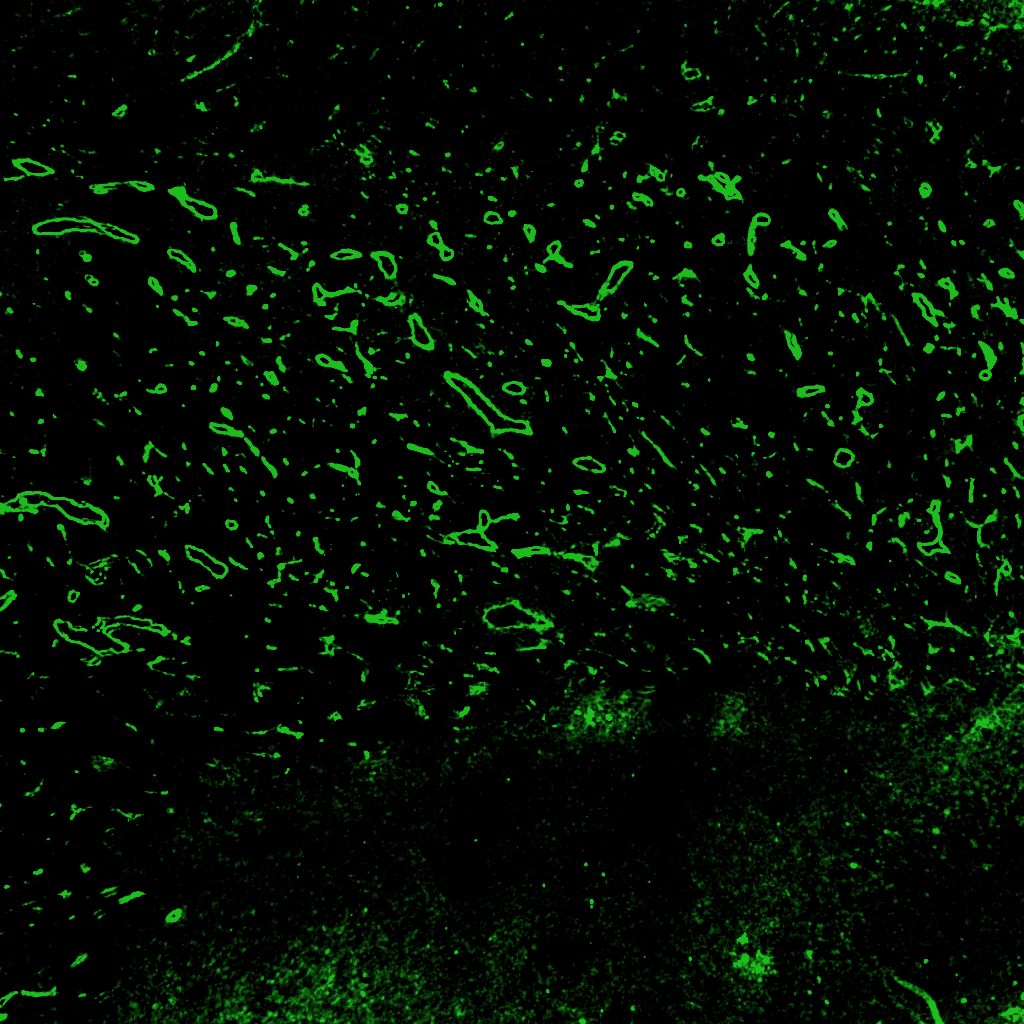

Supplement: Supplementary file 6 — Source data Fig. 6 [file 44318_2024_78_MOESM6_ESM.zip › Figure 6/6K/Vehicle+Vehicle-CD31-Zoom in.tif]

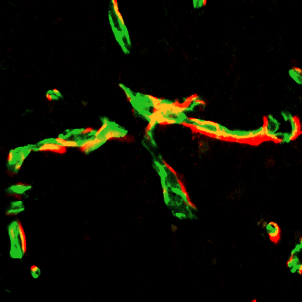

Supplement: Supplementary file 6 — Source data Fig. 6 [file 44318_2024_78_MOESM6_ESM.zip › Figure 6/6K/Vehicle+Fruquintinib-Desmin-Zoom in.tif]

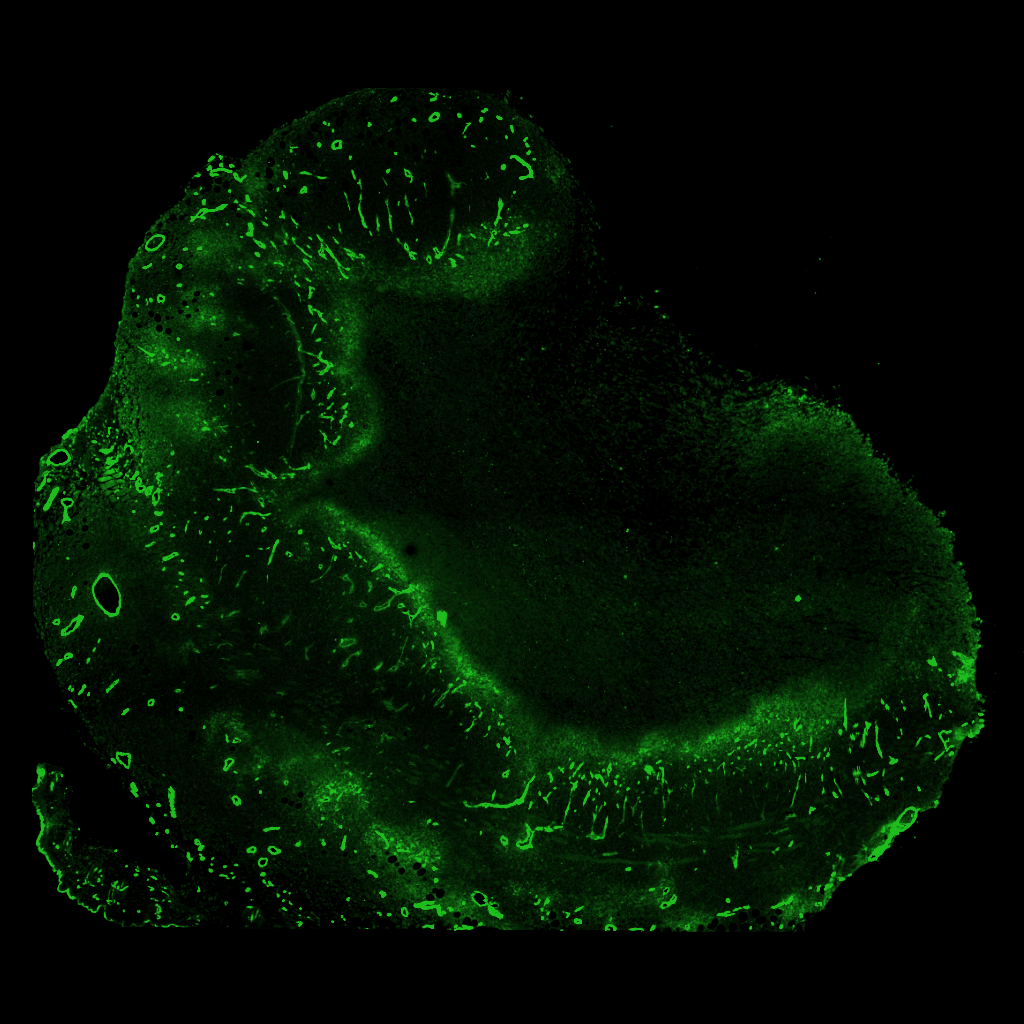

Supplement: Supplementary file 6 — Source data Fig. 6 [file 44318_2024_78_MOESM6_ESM.zip › Figure 6/6K/ODQ+Fruquintinib-CD31.tif]

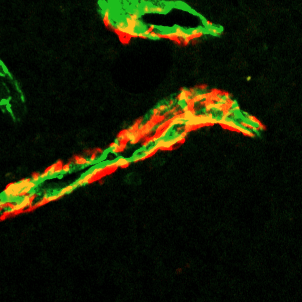

Supplement: Supplementary file 6 — Source data Fig. 6 [file 44318_2024_78_MOESM6_ESM.zip › Figure 6/6K/ODQ+Fruquintinib-Desmin-Zoom in.tif]

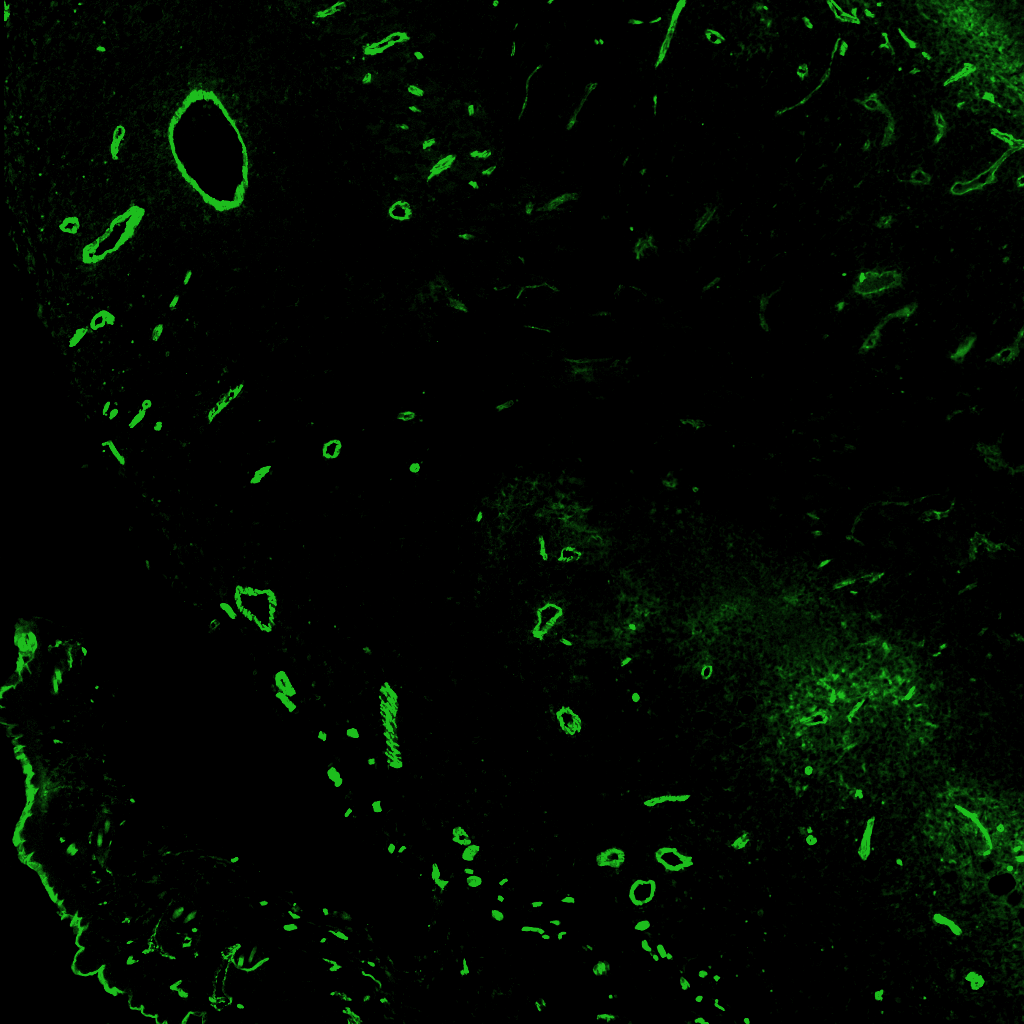

Supplement: Supplementary file 6 — Source data Fig. 6 [file 44318_2024_78_MOESM6_ESM.zip › Figure 6/6K/ODQ+Fruquintinib-CD31-Zoom in.tif]

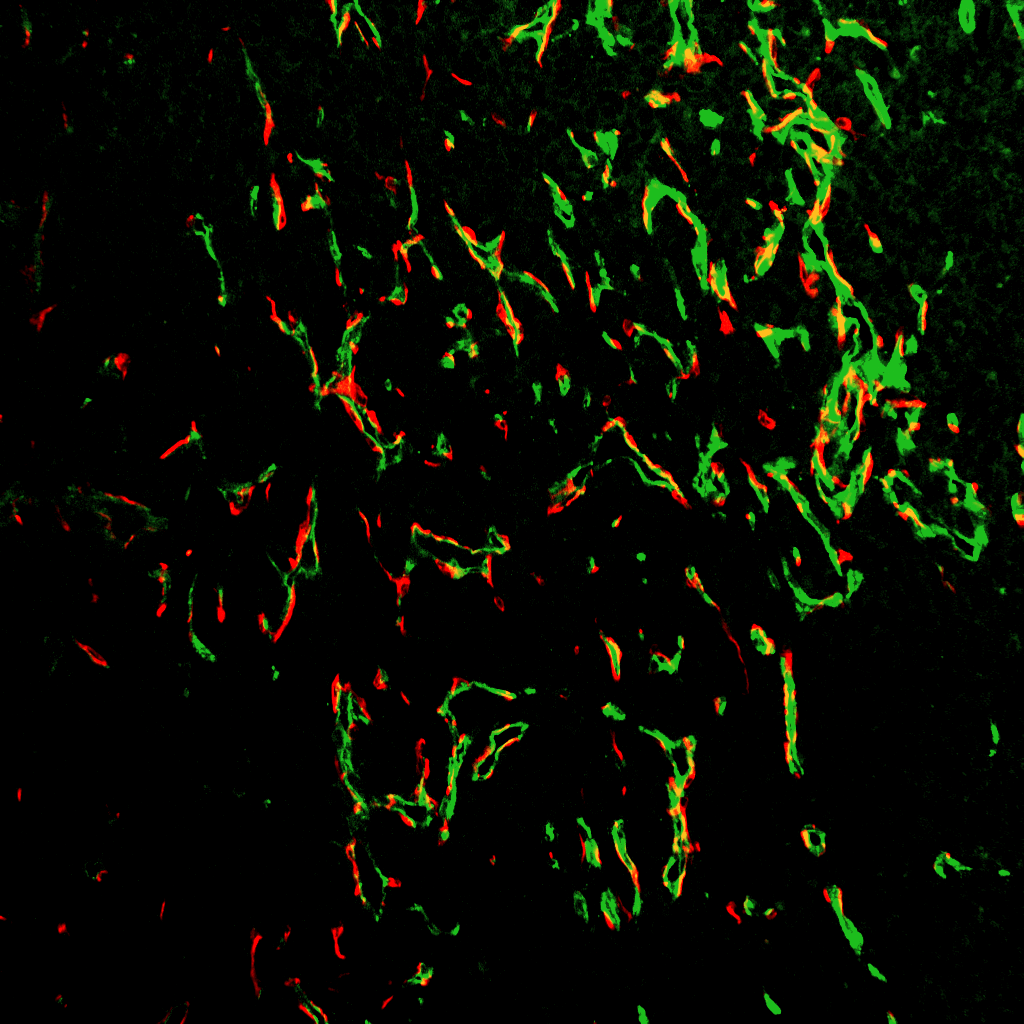

Supplement: Supplementary file 6 — Source data Fig. 6 [file 44318_2024_78_MOESM6_ESM.zip › Figure 6/6K/Vehicle+Vehicle-Desmin.tif]

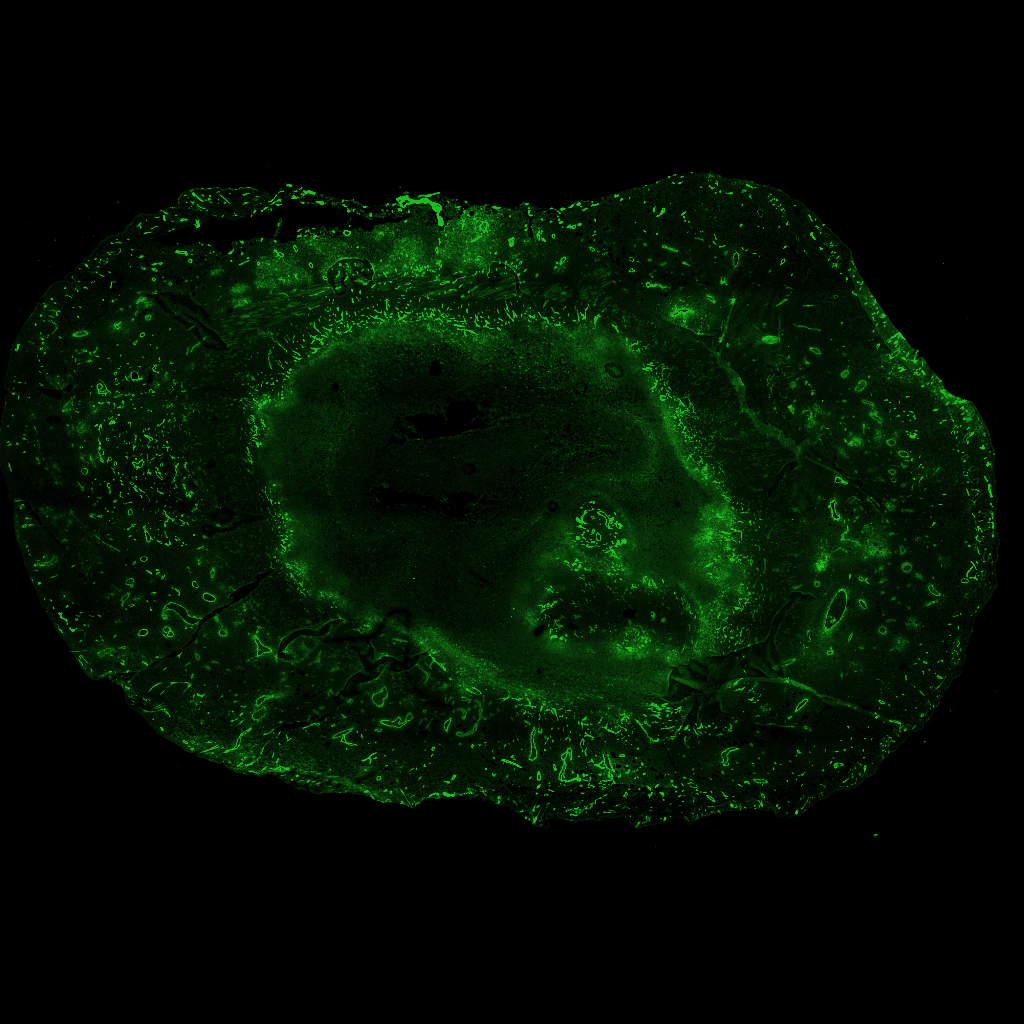

Supplement: Supplementary file 6 — Source data Fig. 6 [file 44318_2024_78_MOESM6_ESM.zip › Figure 6/6K/Vehicle+Fruquintinib-CD31.tif]

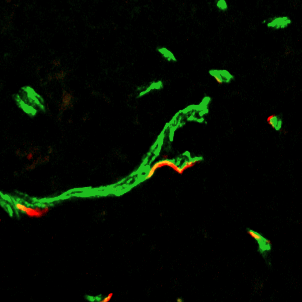

Supplement: Supplementary file 6 — Source data Fig. 6 [file 44318_2024_78_MOESM6_ESM.zip › Figure 6/6K/ODQ+Vehicle-desmin-Zoom in.tif]

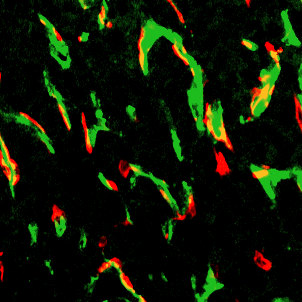

Supplement: Supplementary file 6 — Source data Fig. 6 [file 44318_2024_78_MOESM6_ESM.zip › Figure 6/6K/Vehicle+Vehicle-Desmin-Zoom in.tif]

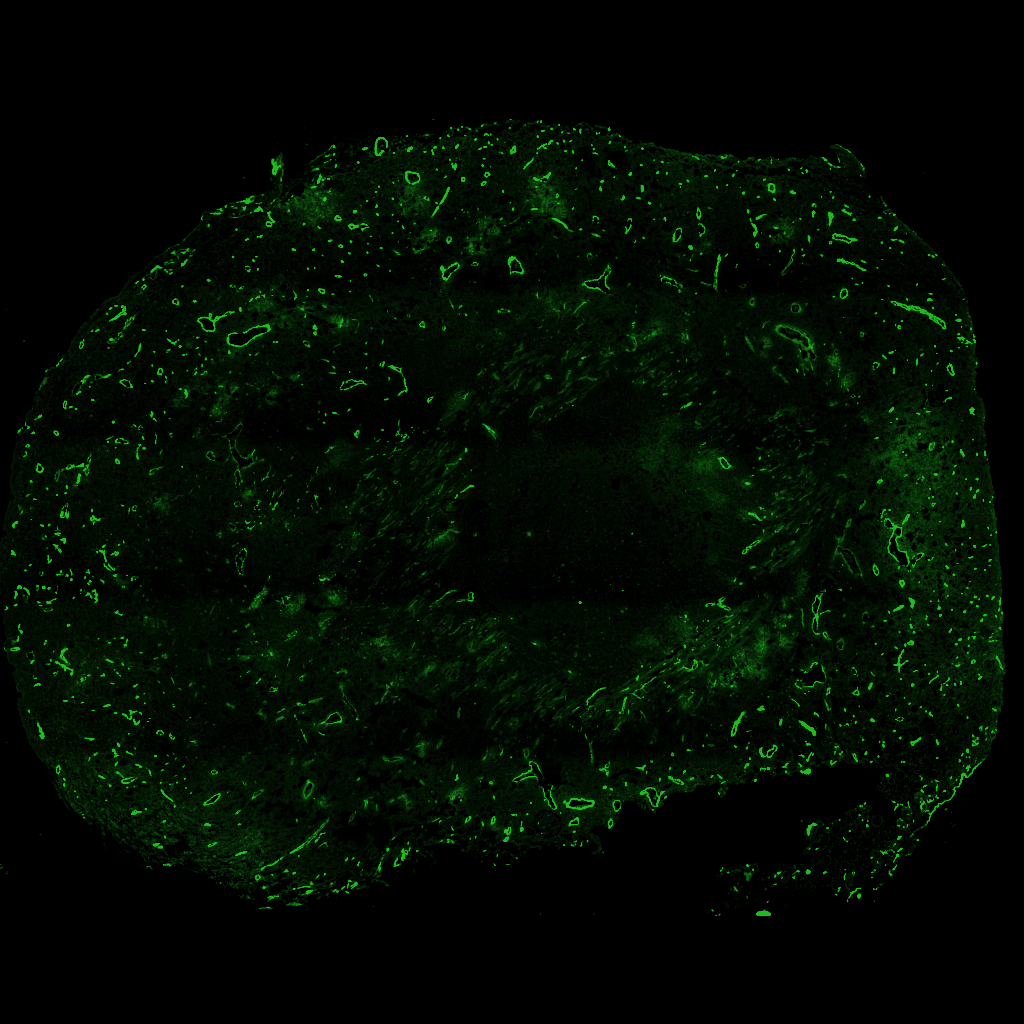

Supplement: Supplementary file 6 — Source data Fig. 6 [file 44318_2024_78_MOESM6_ESM.zip › Figure 6/6K/ODQ+Vehicle-CD31.tif]

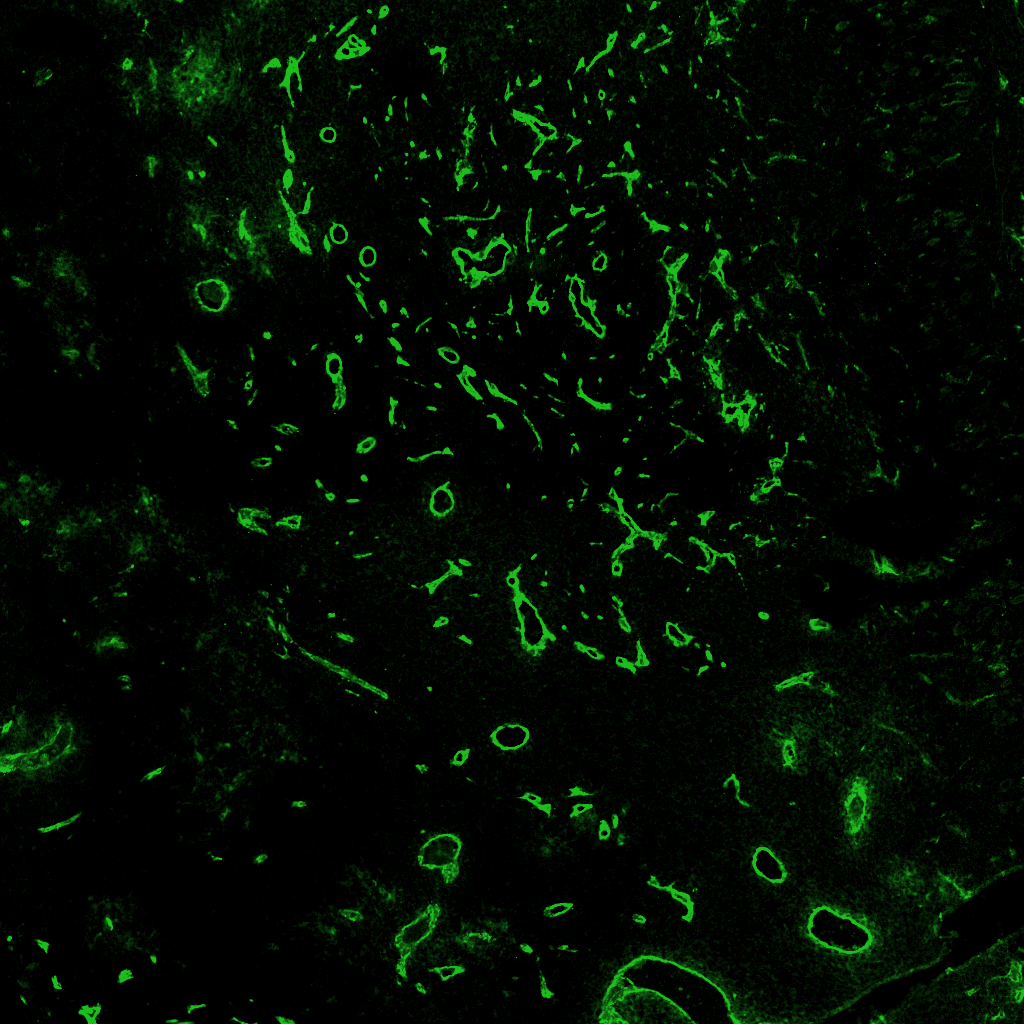

Supplement: Supplementary file 6 — Source data Fig. 6 [file 44318_2024_78_MOESM6_ESM.zip › Figure 6/6K/Vehicle+Fruquintinib-CD31-Zoom in.tif]

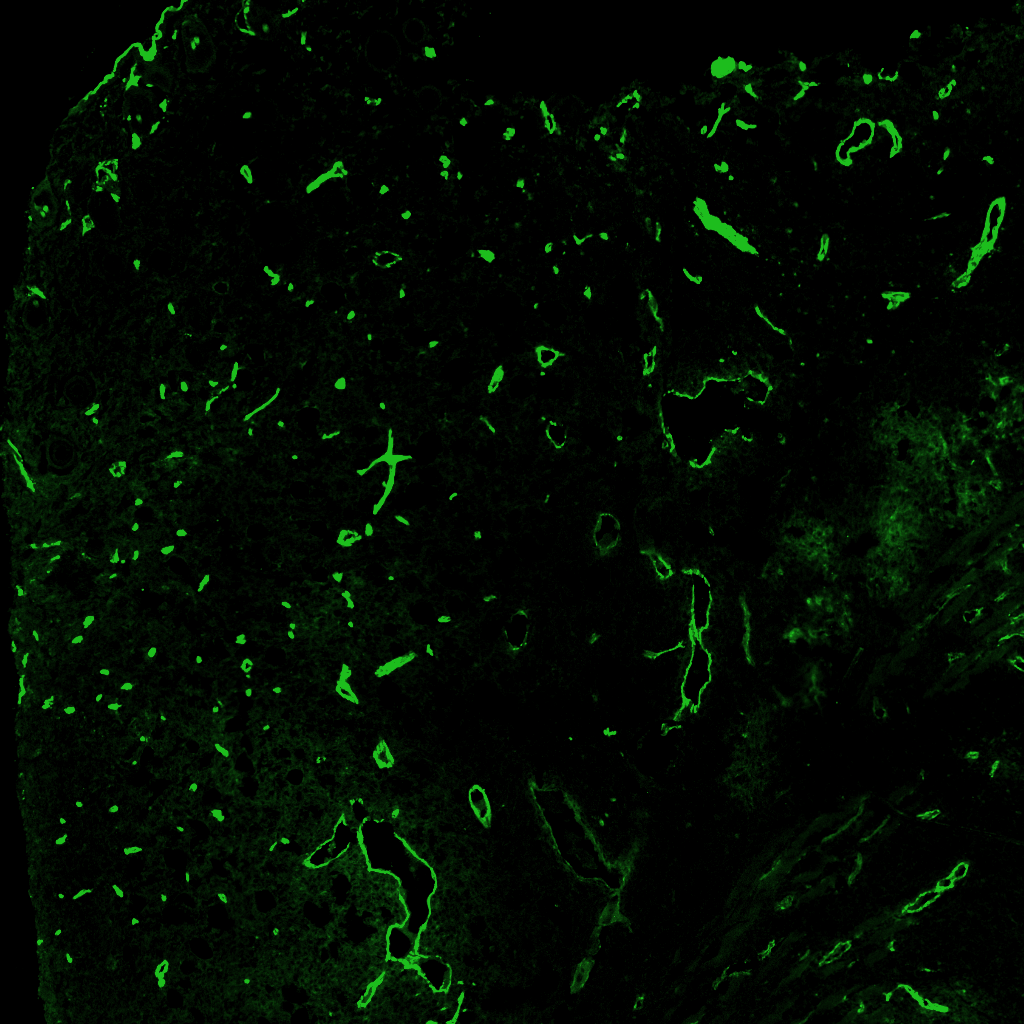

Supplement: Supplementary file 6 — Source data Fig. 6 [file 44318_2024_78_MOESM6_ESM.zip › Figure 6/6K/ODQ+Vehicle-CD31-Zoom in.tif]

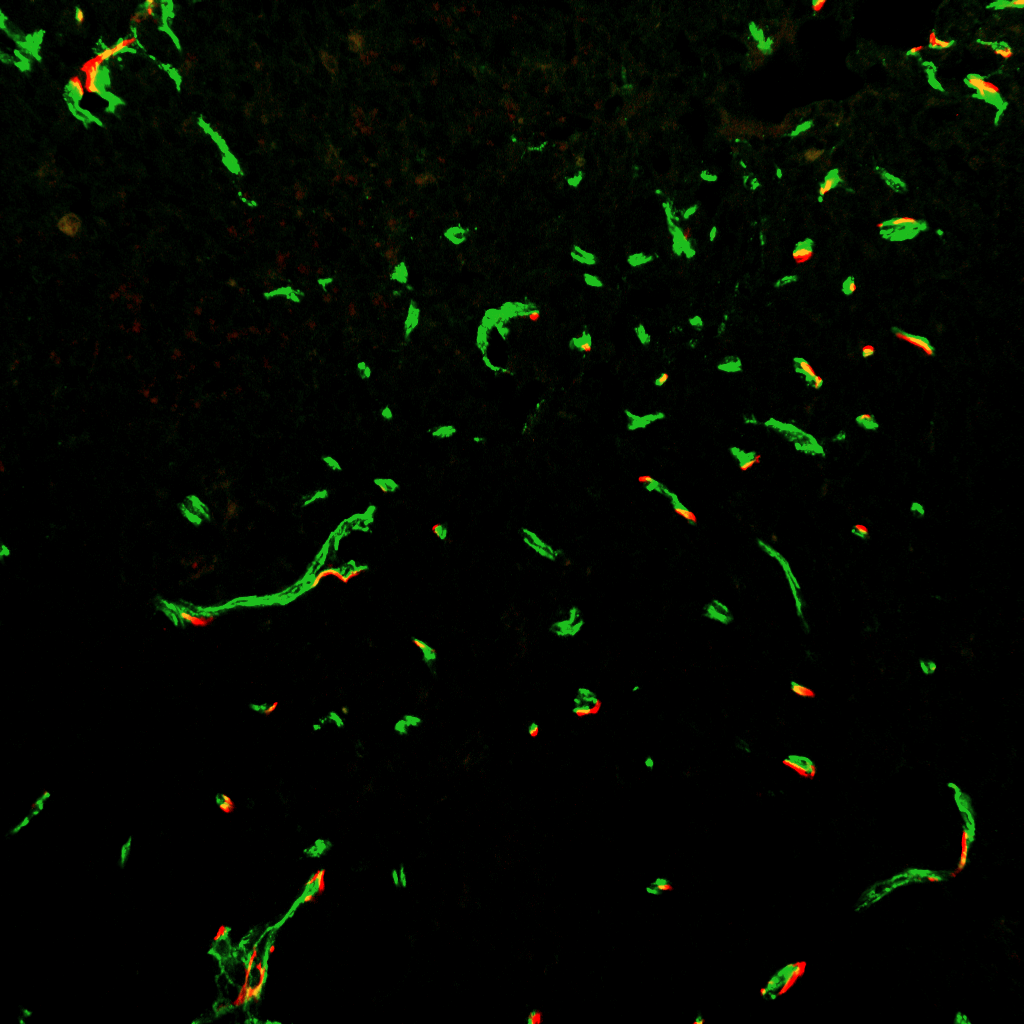

Supplement: Supplementary file 6 — Source data Fig. 6 [file 44318_2024_78_MOESM6_ESM.zip › Figure 6/6K/ODQ+Vehicle-desmin.tif]

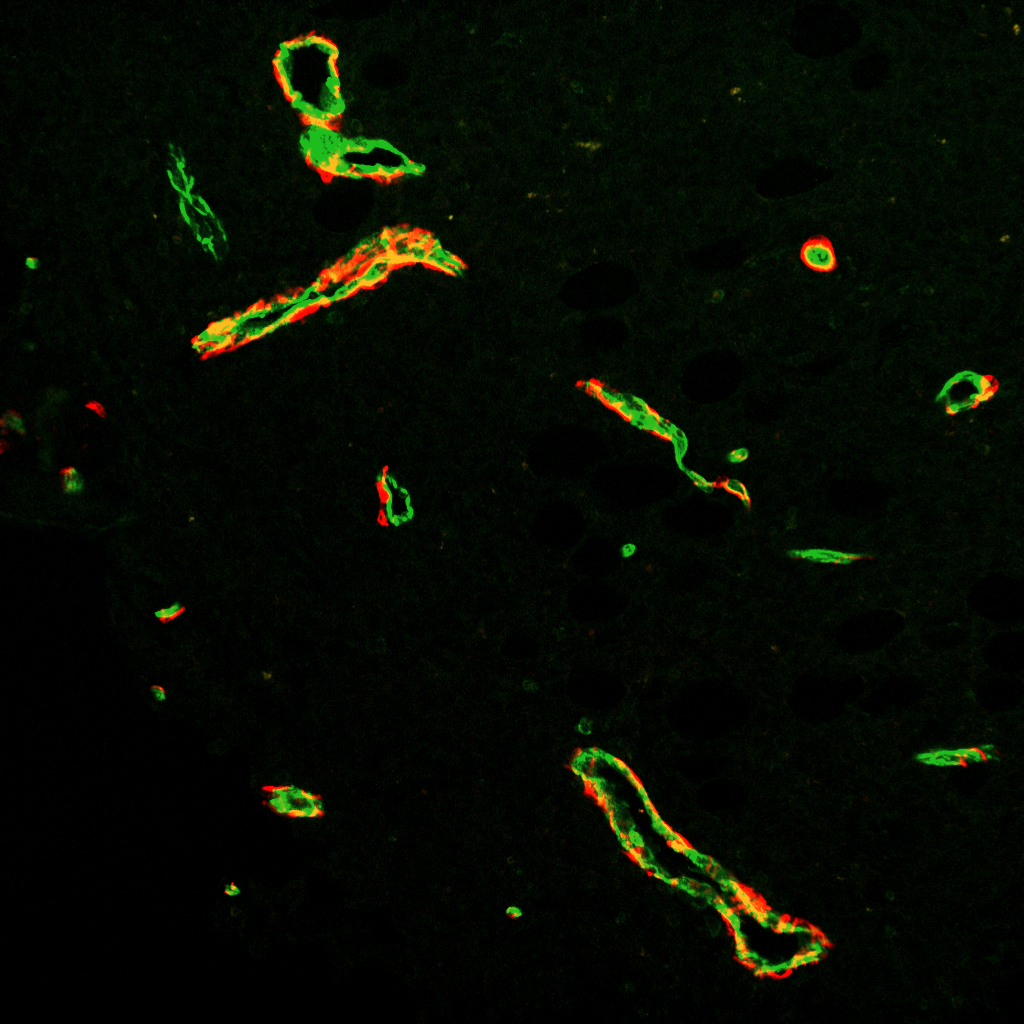

Supplement: Supplementary file 6 — Source data Fig. 6 [file 44318_2024_78_MOESM6_ESM.zip › Figure 6/6K/ODQ+Fruquintinib-Desmin.tif]

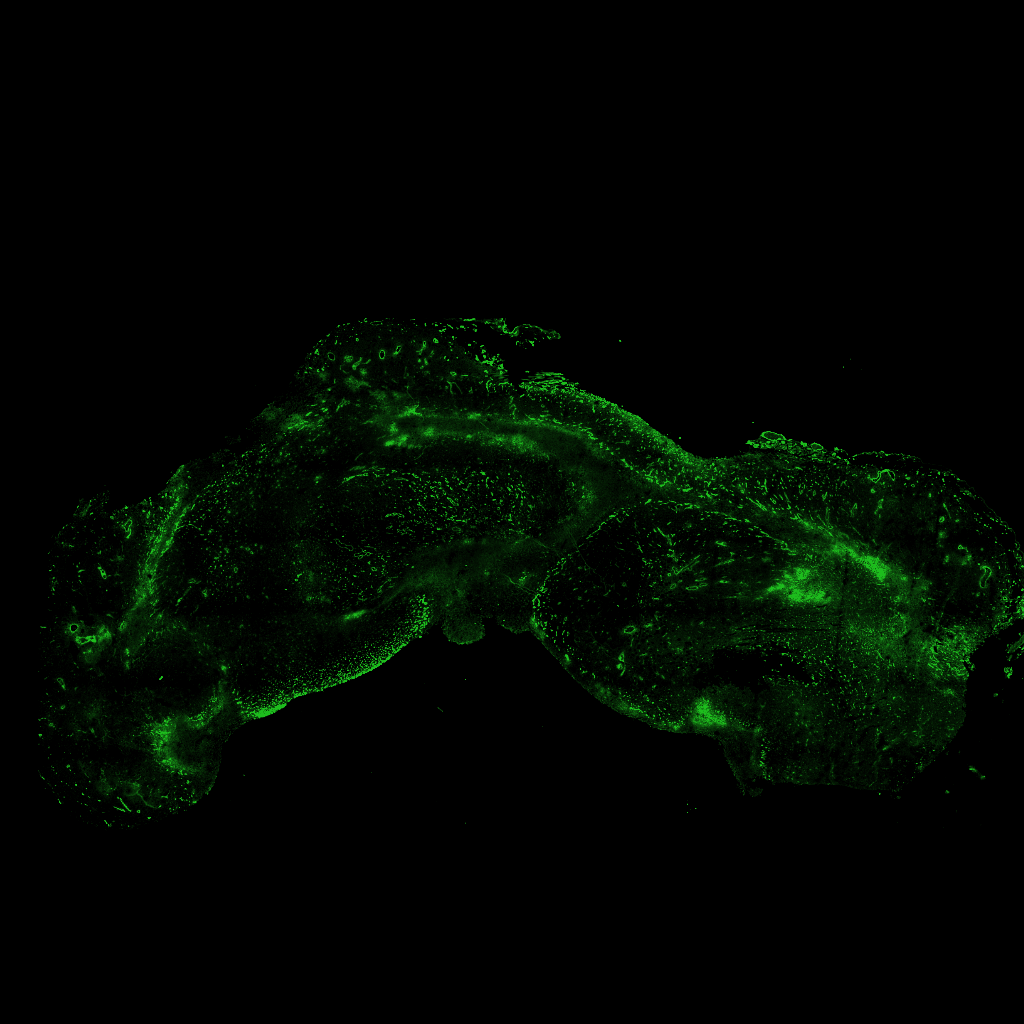

Supplement: Supplementary file 6 — Source data Fig. 6 [file 44318_2024_78_MOESM6_ESM.zip › Figure 6/6K/Vehicle+Vehicle-CD31.tif]

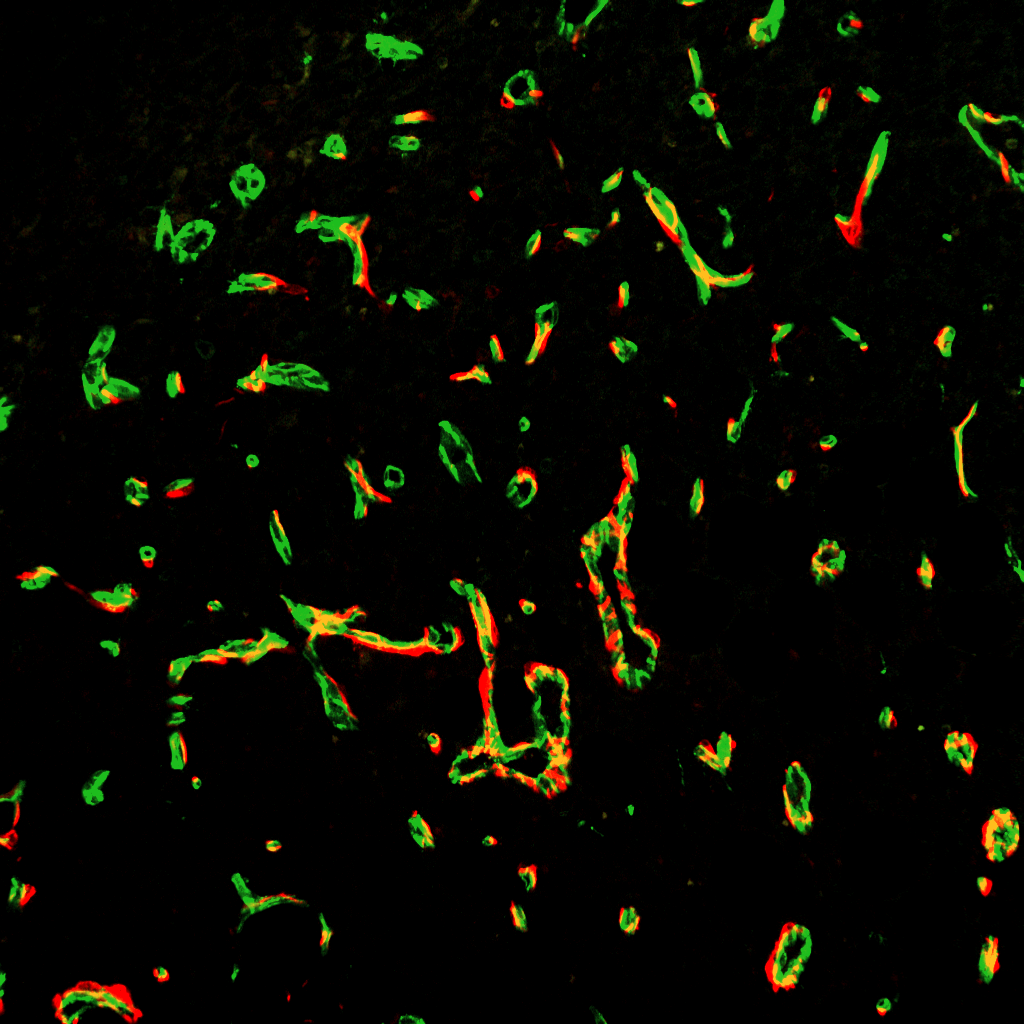

Supplement: Supplementary file 6 — Source data Fig. 6 [file 44318_2024_78_MOESM6_ESM.zip › Figure 6/6K/Vehicle+Fruquintinib-Desmin.tif]

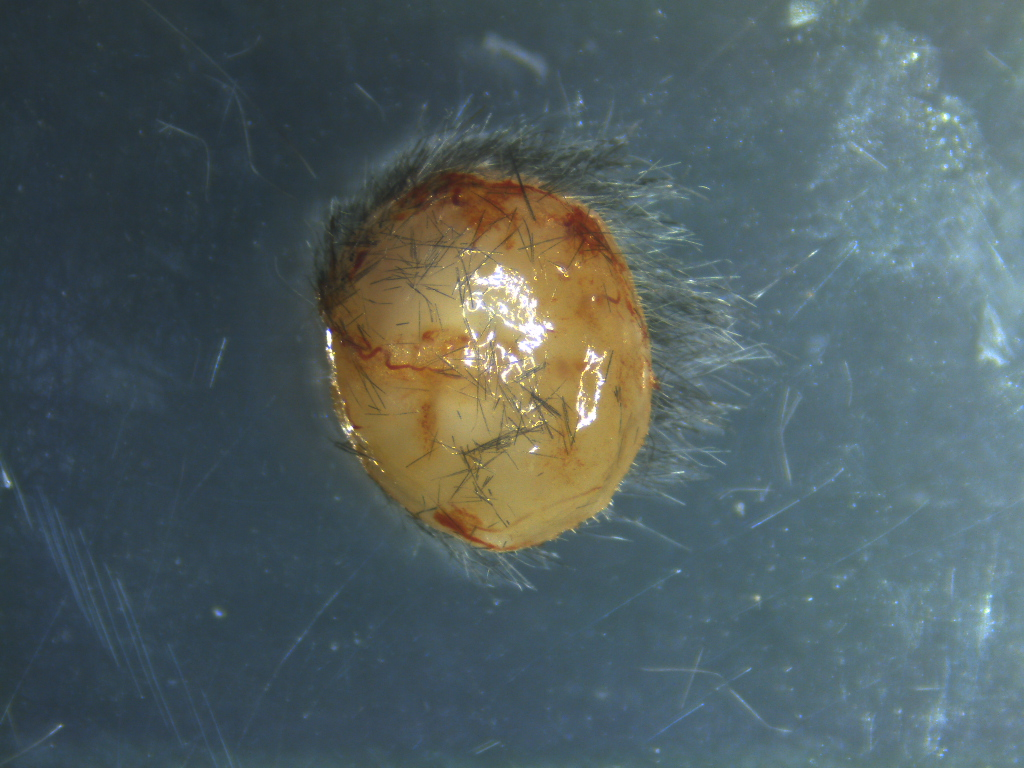

Supplement: Supplementary file 6 — Source data Fig. 6 [file 44318_2024_78_MOESM6_ESM.zip › Figure 6/6C/sGC╬öpc+Fruquintinib-6.tif]

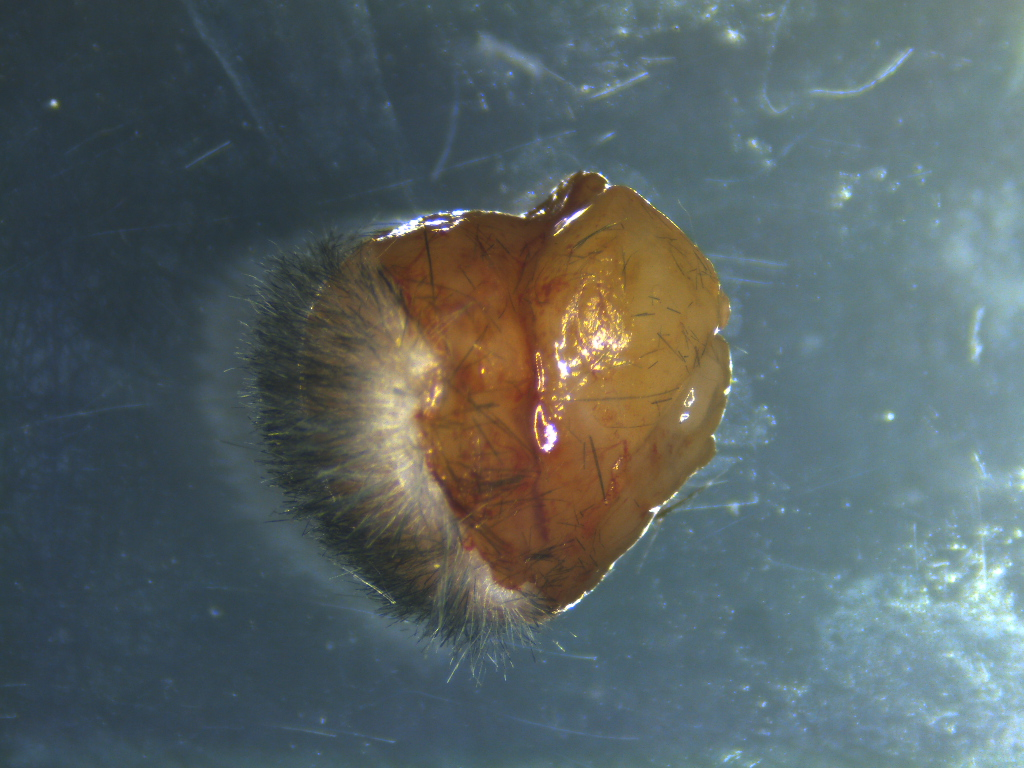

Supplement: Supplementary file 6 — Source data Fig. 6 [file 44318_2024_78_MOESM6_ESM.zip › Figure 6/6C/sGC╬öpc+Vehicle-7.tif]

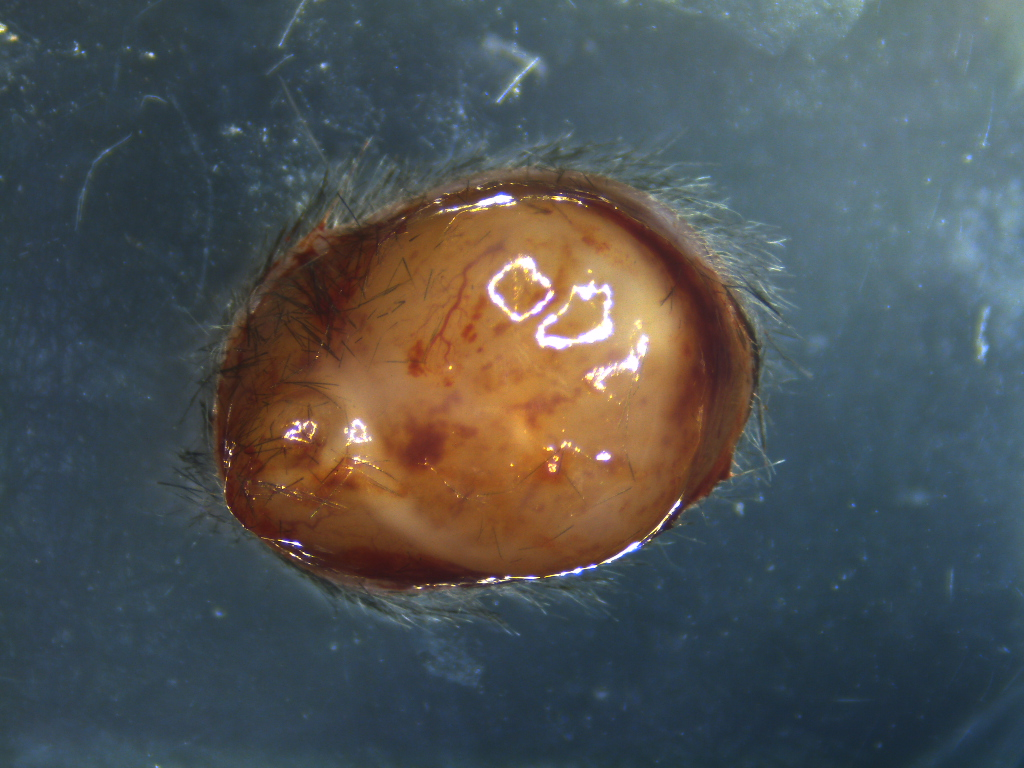

Supplement: Supplementary file 6 — Source data Fig. 6 [file 44318_2024_78_MOESM6_ESM.zip › Figure 6/6C/sGC╬öpc+Vehicle-6.tif]

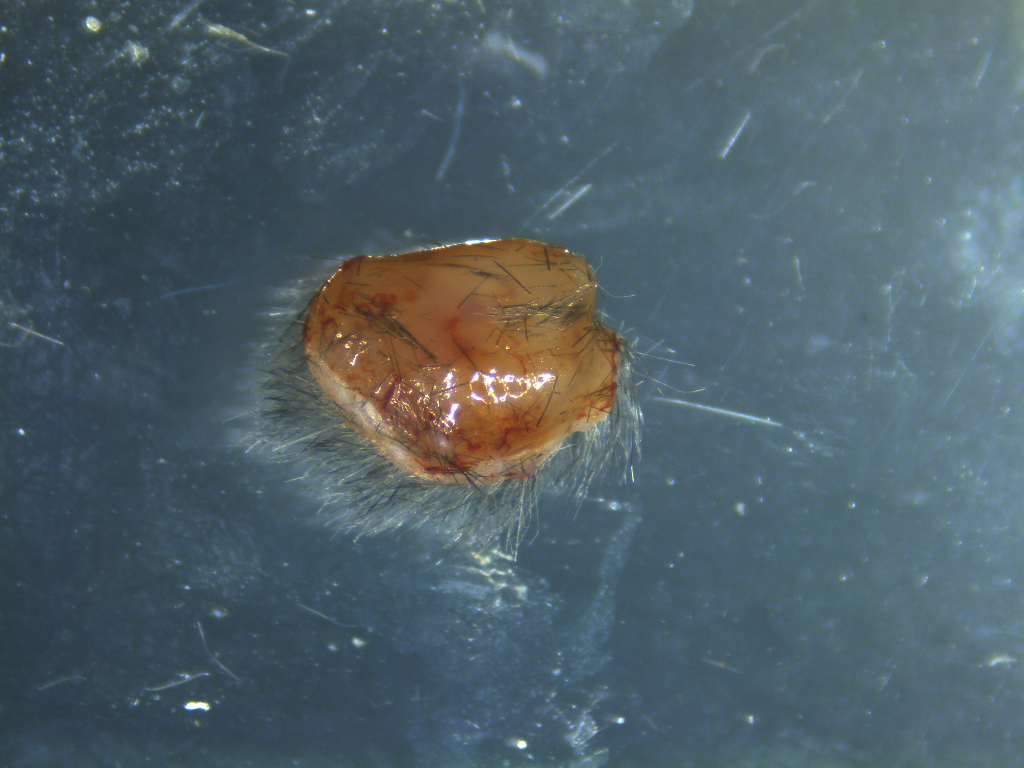

Supplement: Supplementary file 6 — Source data Fig. 6 [file 44318_2024_78_MOESM6_ESM.zip › Figure 6/6C/sGC╬öpc+Fruquintinib-7.tif]

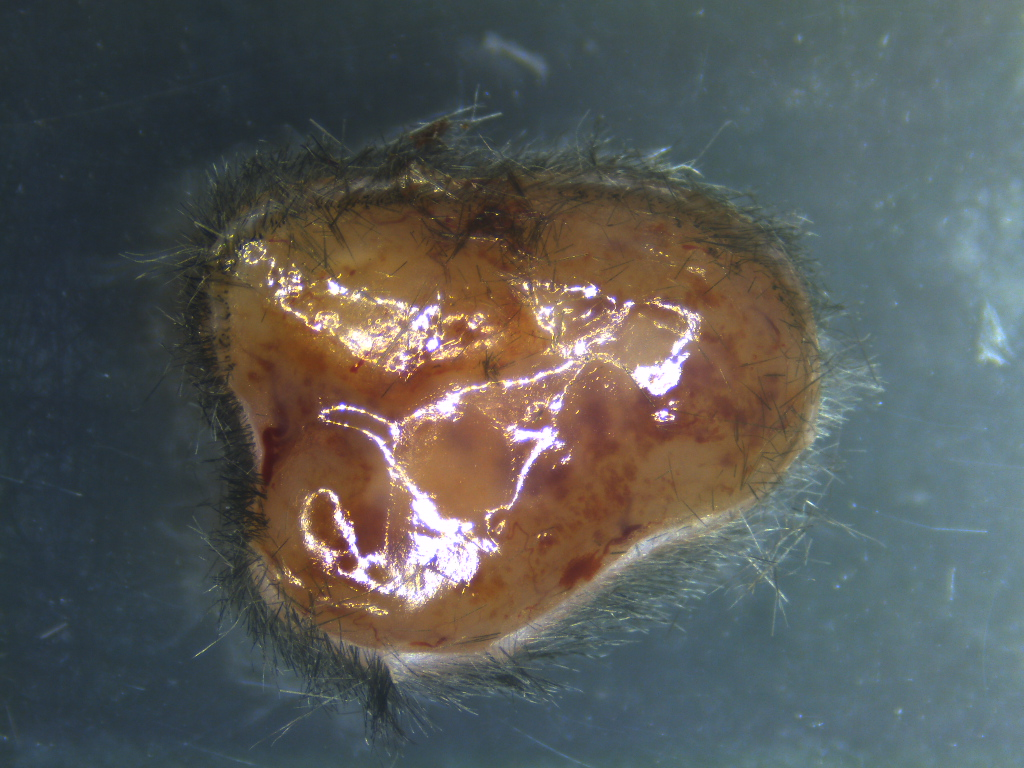

Supplement: Supplementary file 6 — Source data Fig. 6 [file 44318_2024_78_MOESM6_ESM.zip › Figure 6/6C/sGC╬öpc+Vehicle-4.tif]

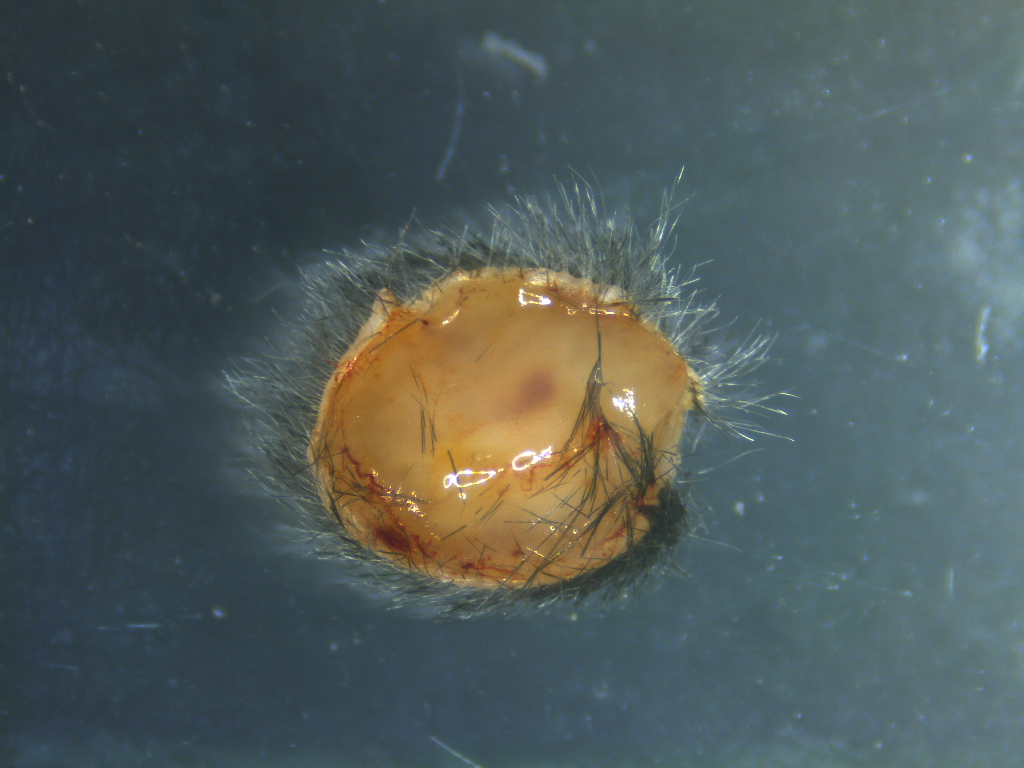

Supplement: Supplementary file 6 — Source data Fig. 6 [file 44318_2024_78_MOESM6_ESM.zip › Figure 6/6C/sGC╬öpc+Fruquintinib-5.tif]

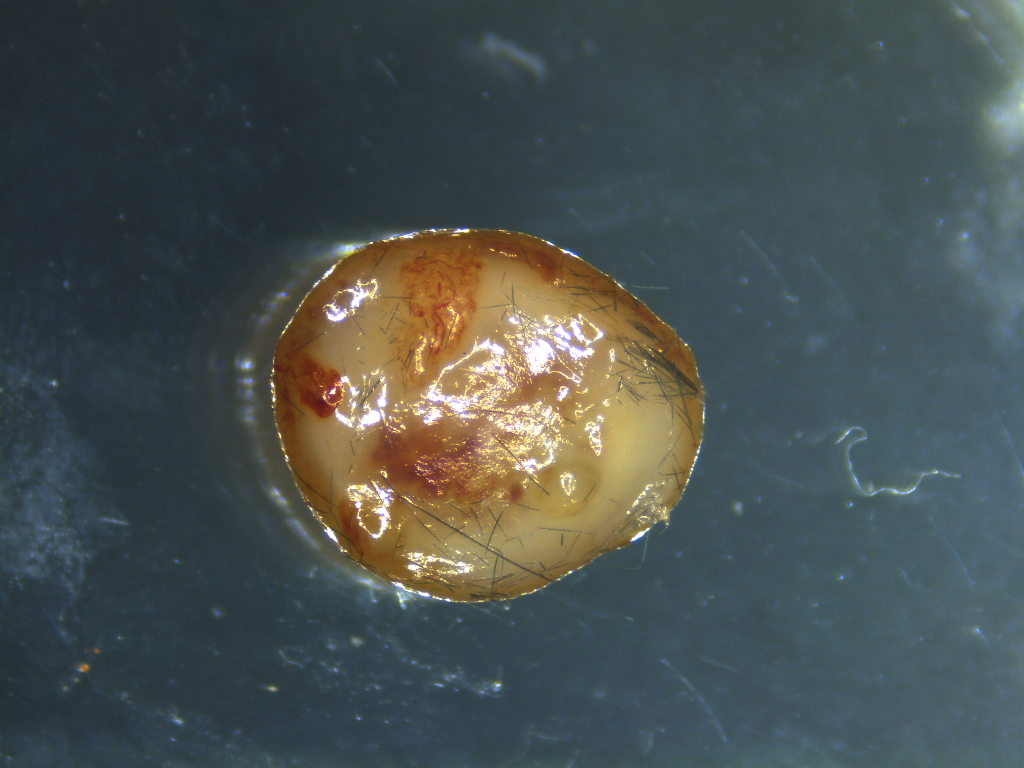

Supplement: Supplementary file 6 — Source data Fig. 6 [file 44318_2024_78_MOESM6_ESM.zip › Figure 6/6C/sGC╬öpc+Fruquintinib-4.tif]

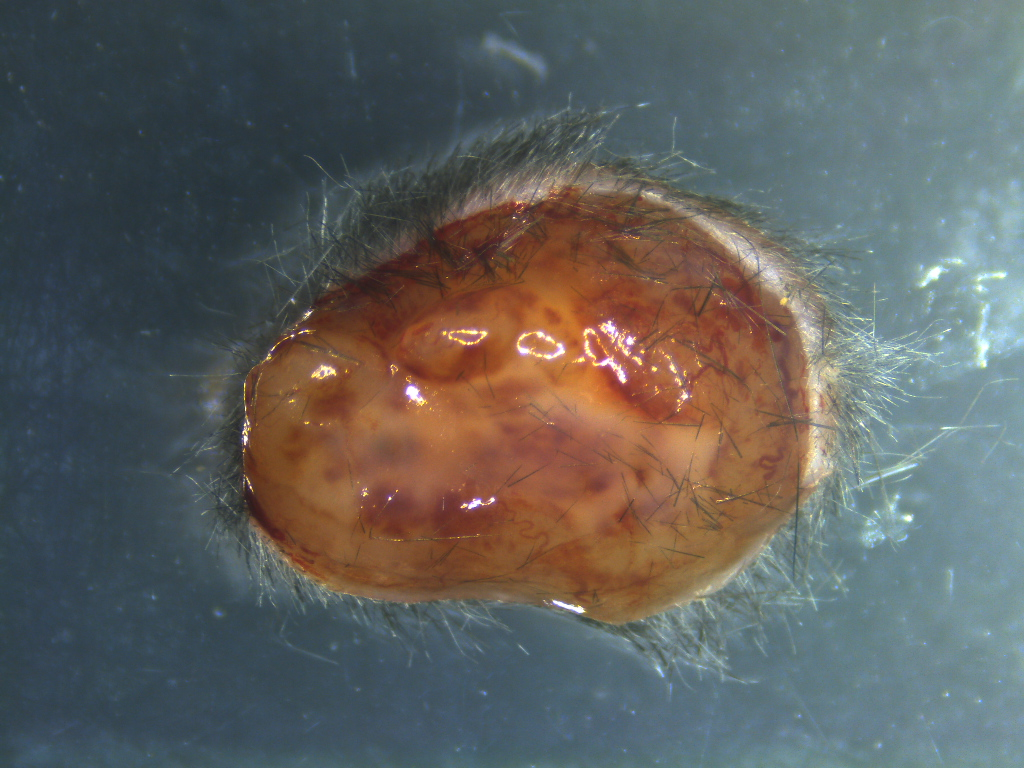

Supplement: Supplementary file 6 — Source data Fig. 6 [file 44318_2024_78_MOESM6_ESM.zip › Figure 6/6C/sGC╬öpc+Vehicle-5.tif]

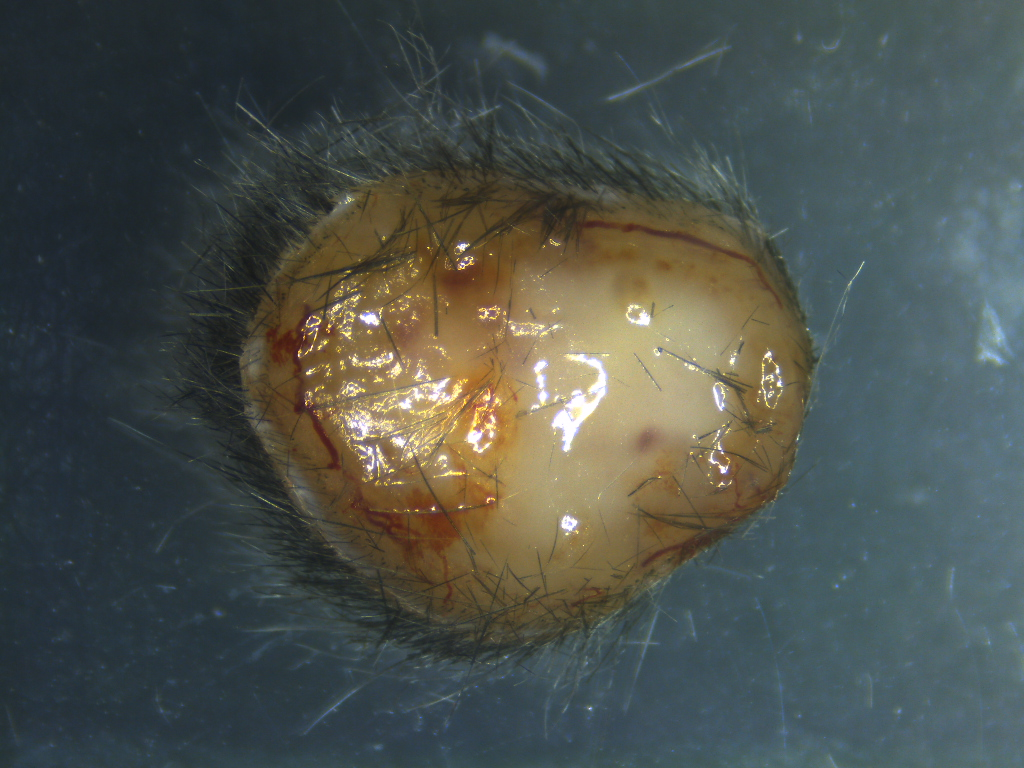

Supplement: Supplementary file 6 — Source data Fig. 6 [file 44318_2024_78_MOESM6_ESM.zip › Figure 6/6C/sGCCtr+Fruquintinib-8.tif]

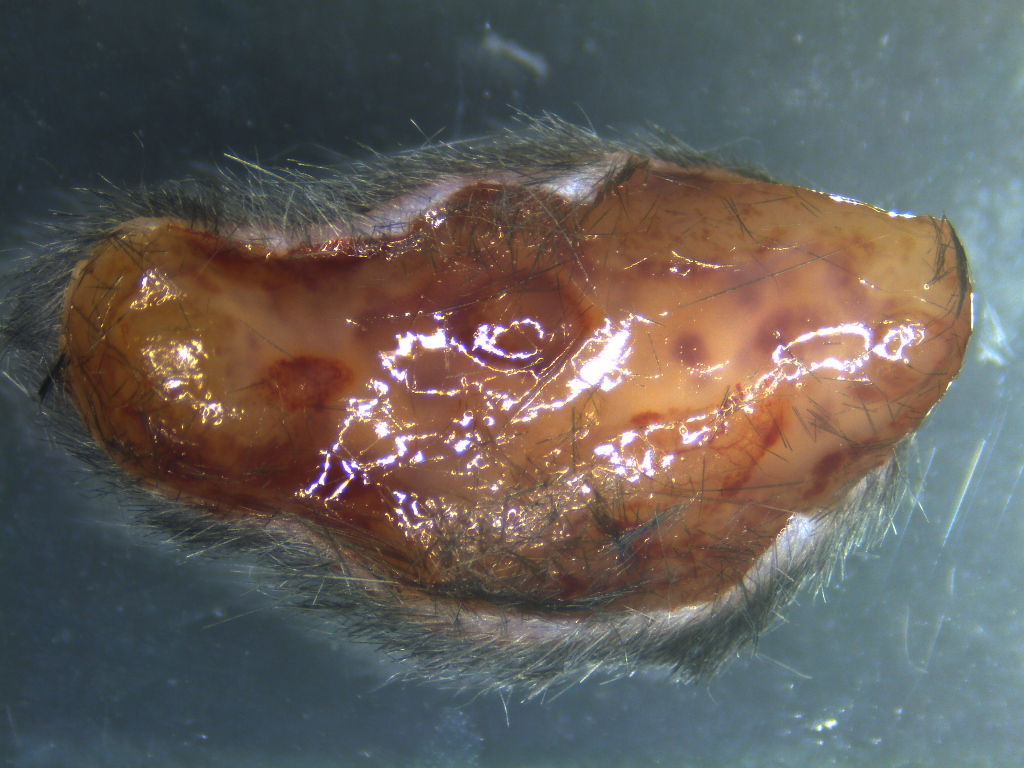

Supplement: Supplementary file 6 — Source data Fig. 6 [file 44318_2024_78_MOESM6_ESM.zip › Figure 6/6C/sGC╬öpc+Vehicle-1.tif]

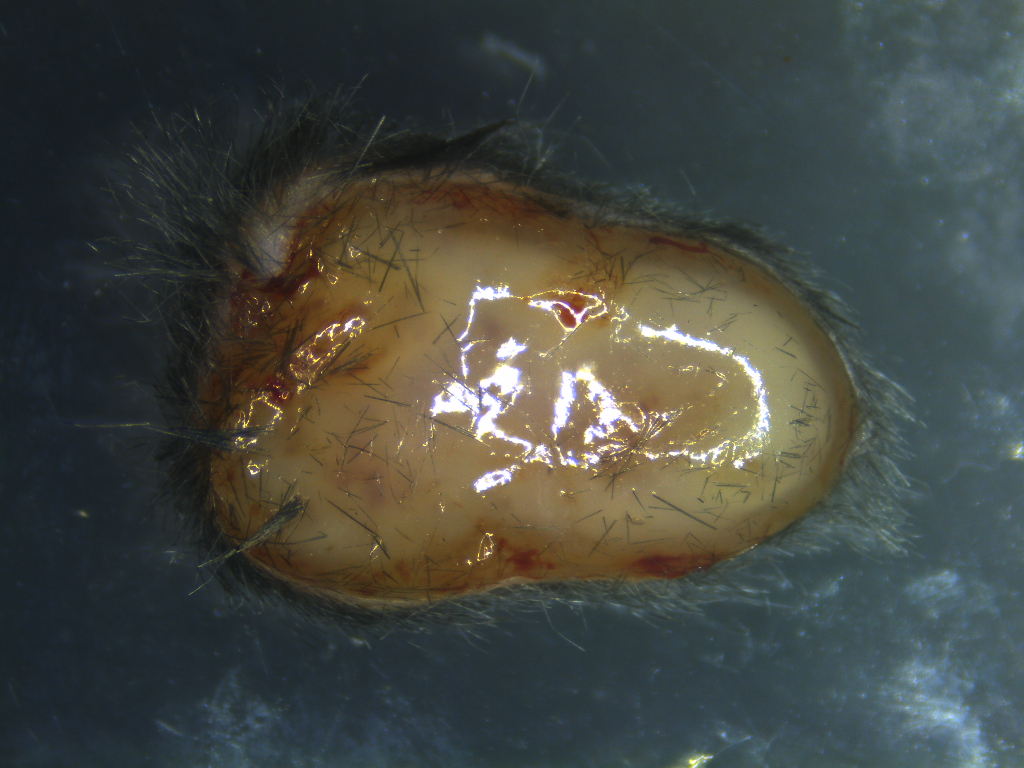

Supplement: Supplementary file 6 — Source data Fig. 6 [file 44318_2024_78_MOESM6_ESM.zip › Figure 6/6C/sGC╬öpc+Fruquintinib-1.tif]

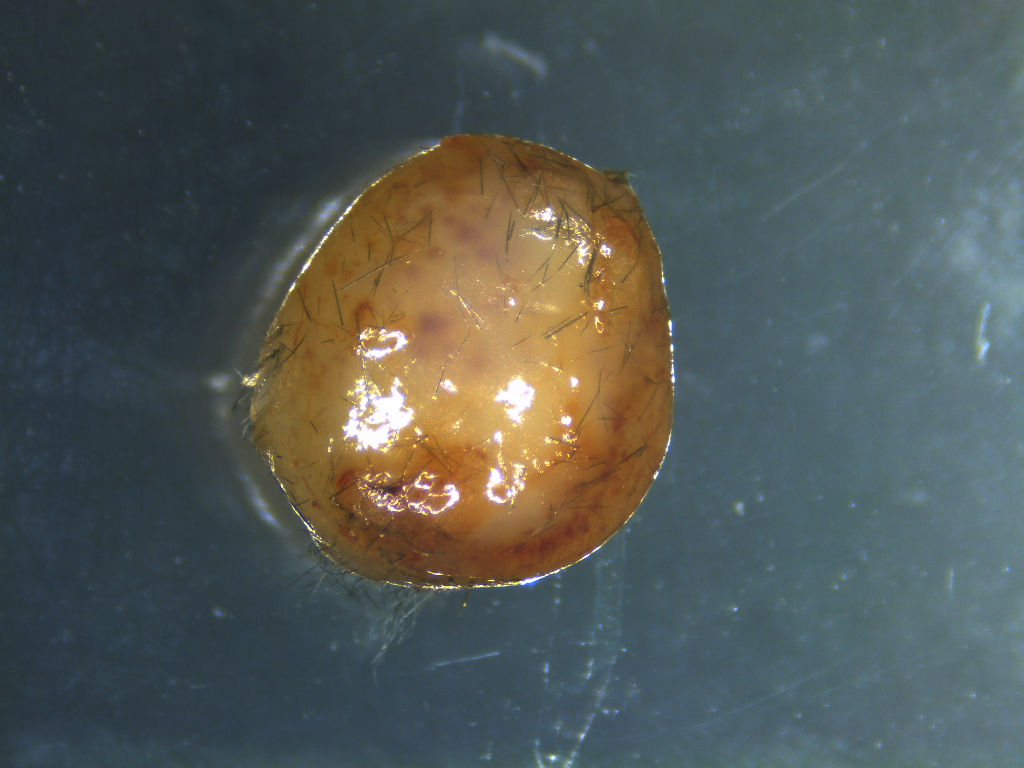

Supplement: Supplementary file 6 — Source data Fig. 6 [file 44318_2024_78_MOESM6_ESM.zip › Figure 6/6C/sGC╬öpc+Fruquintinib-3.tif]

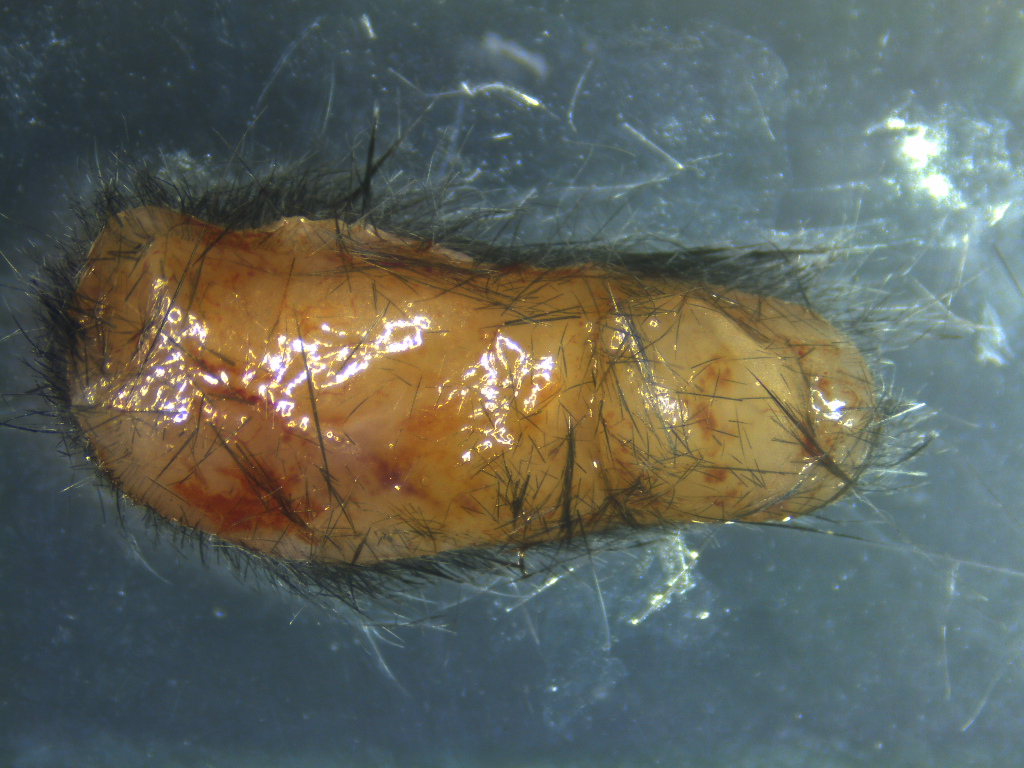

Supplement: Supplementary file 6 — Source data Fig. 6 [file 44318_2024_78_MOESM6_ESM.zip › Figure 6/6C/sGC╬öpc+Vehicle-2.tif]

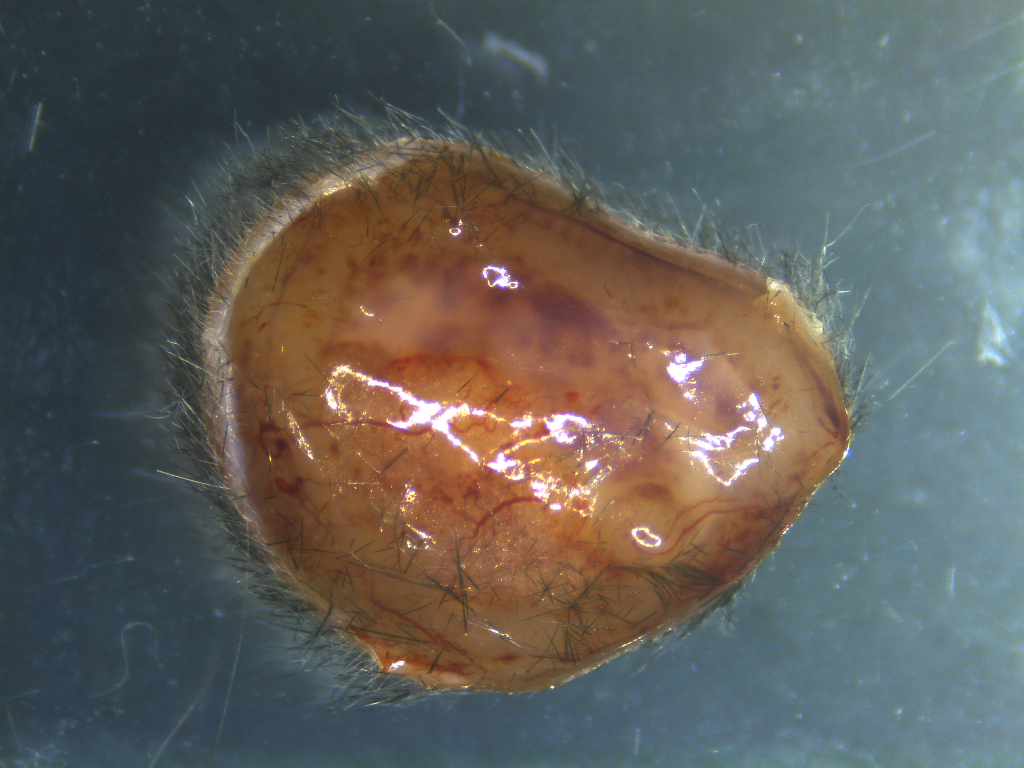

Supplement: Supplementary file 6 — Source data Fig. 6 [file 44318_2024_78_MOESM6_ESM.zip › Figure 6/6C/sGC╬öpc+Vehicle-3.tif]

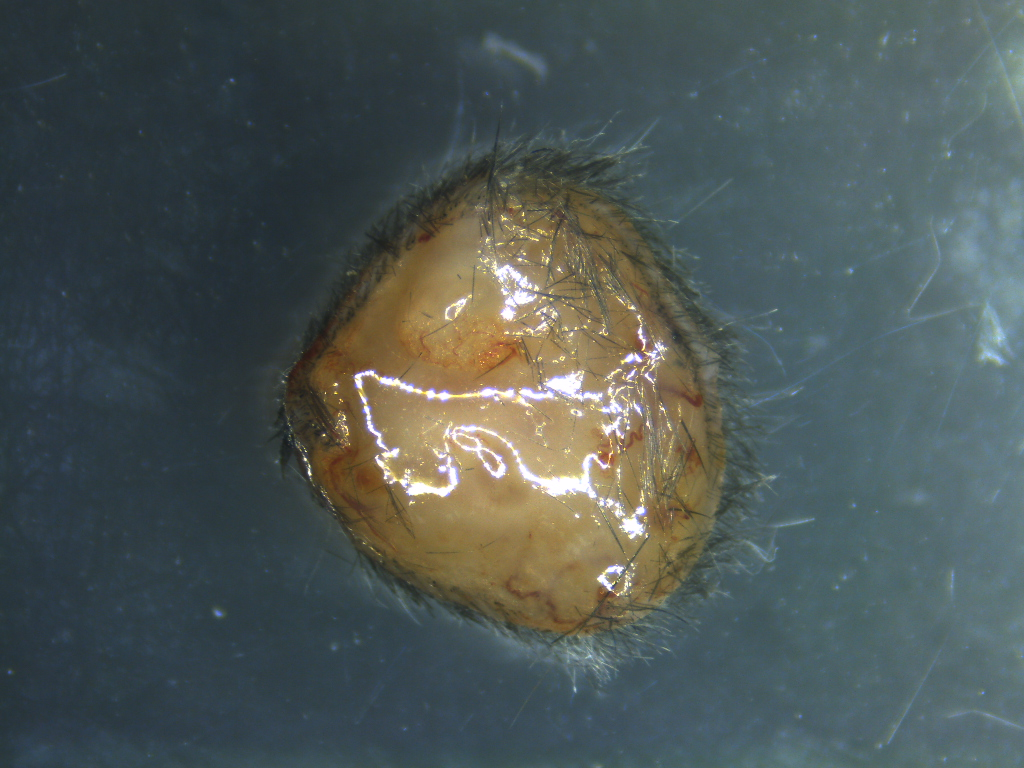

Supplement: Supplementary file 6 — Source data Fig. 6 [file 44318_2024_78_MOESM6_ESM.zip › Figure 6/6C/sGC╬öpc+Fruquintinib-2.tif]

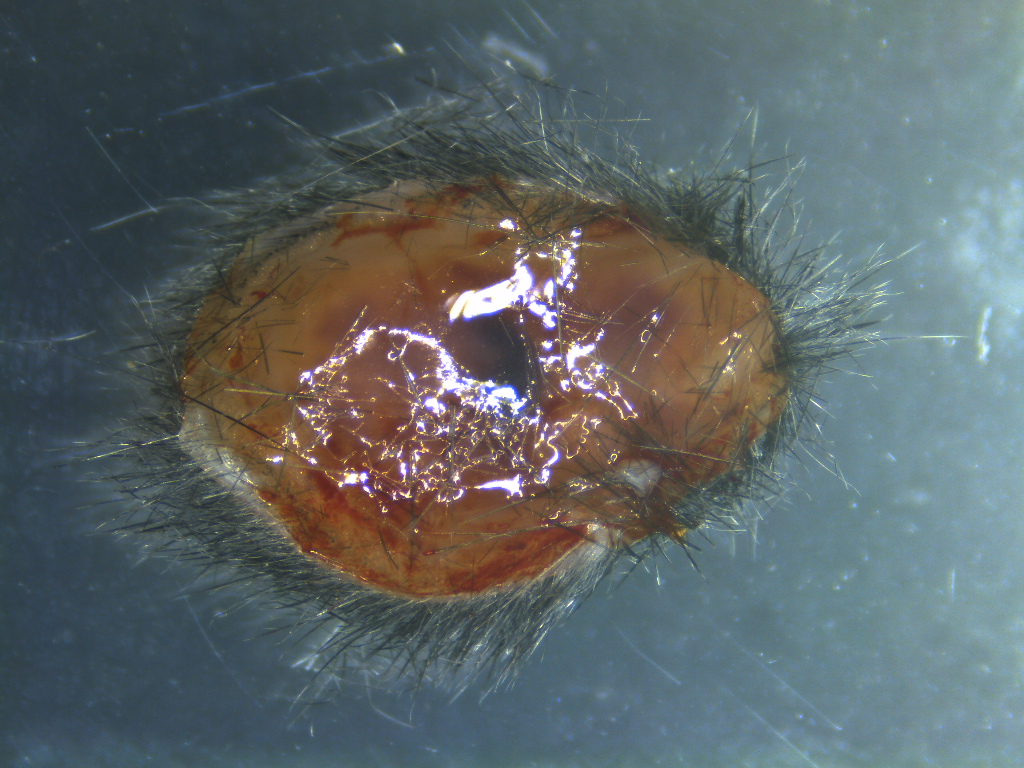

Supplement: Supplementary file 6 — Source data Fig. 6 [file 44318_2024_78_MOESM6_ESM.zip › Figure 6/6C/sGCCtr+Vehicle-8.tif]

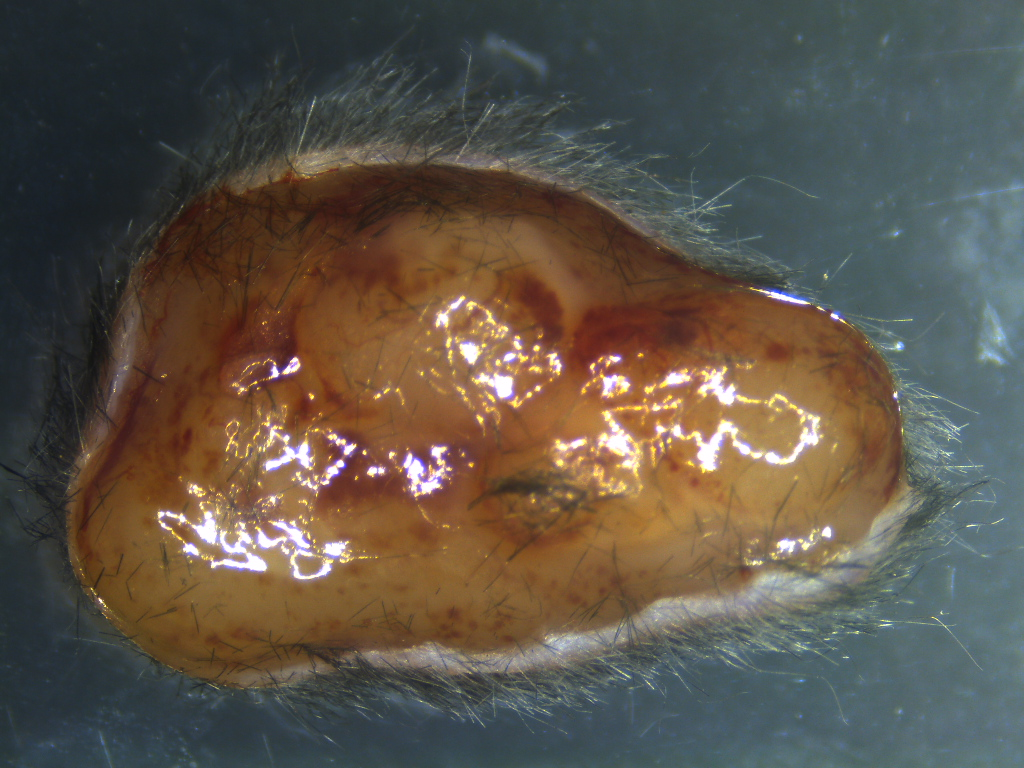

Supplement: Supplementary file 6 — Source data Fig. 6 [file 44318_2024_78_MOESM6_ESM.zip › Figure 6/6C/sGCCtr+Vehicle-4.tif]

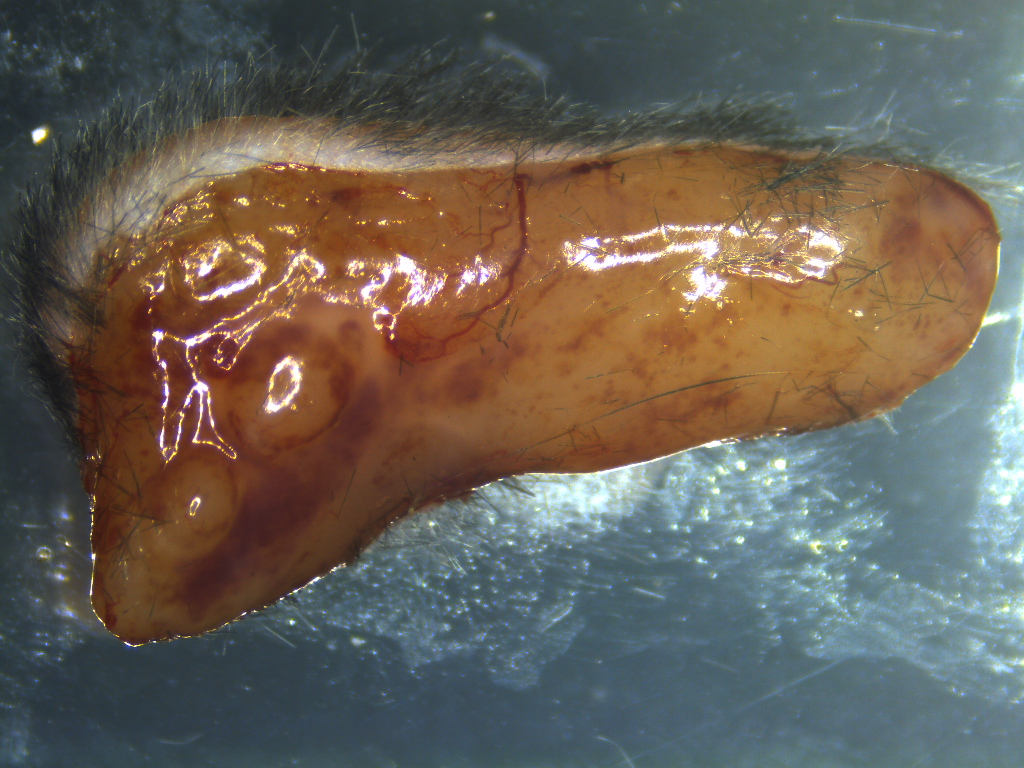

Supplement: Supplementary file 6 — Source data Fig. 6 [file 44318_2024_78_MOESM6_ESM.zip › Figure 6/6C/sGCCtr+Vehicle-5.tif]

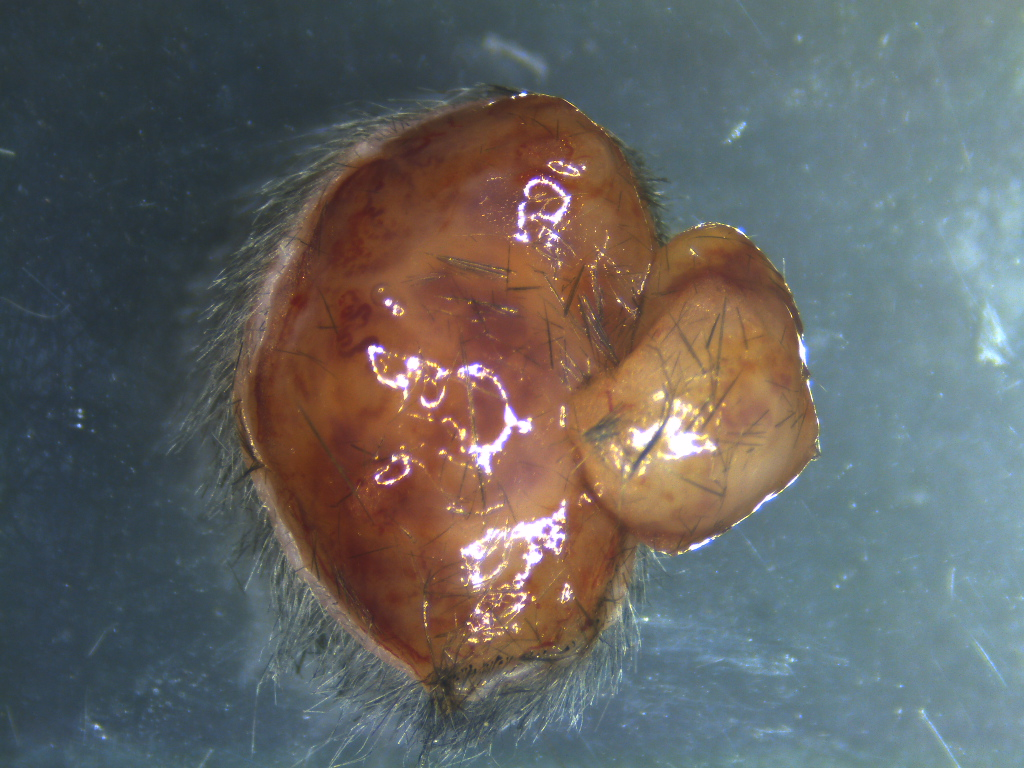

Supplement: Supplementary file 6 — Source data Fig. 6 [file 44318_2024_78_MOESM6_ESM.zip › Figure 6/6C/sGCCtr+Vehicle-7.tif]

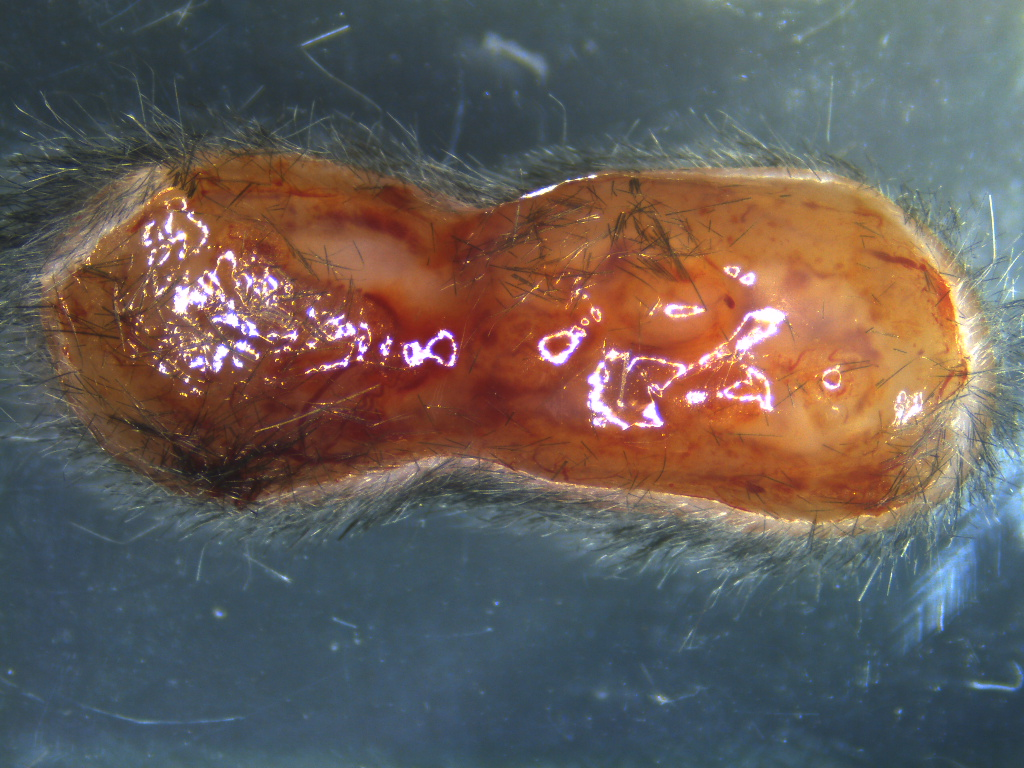

Supplement: Supplementary file 6 — Source data Fig. 6 [file 44318_2024_78_MOESM6_ESM.zip › Figure 6/6C/sGCCtr+Vehicle-6.tif]

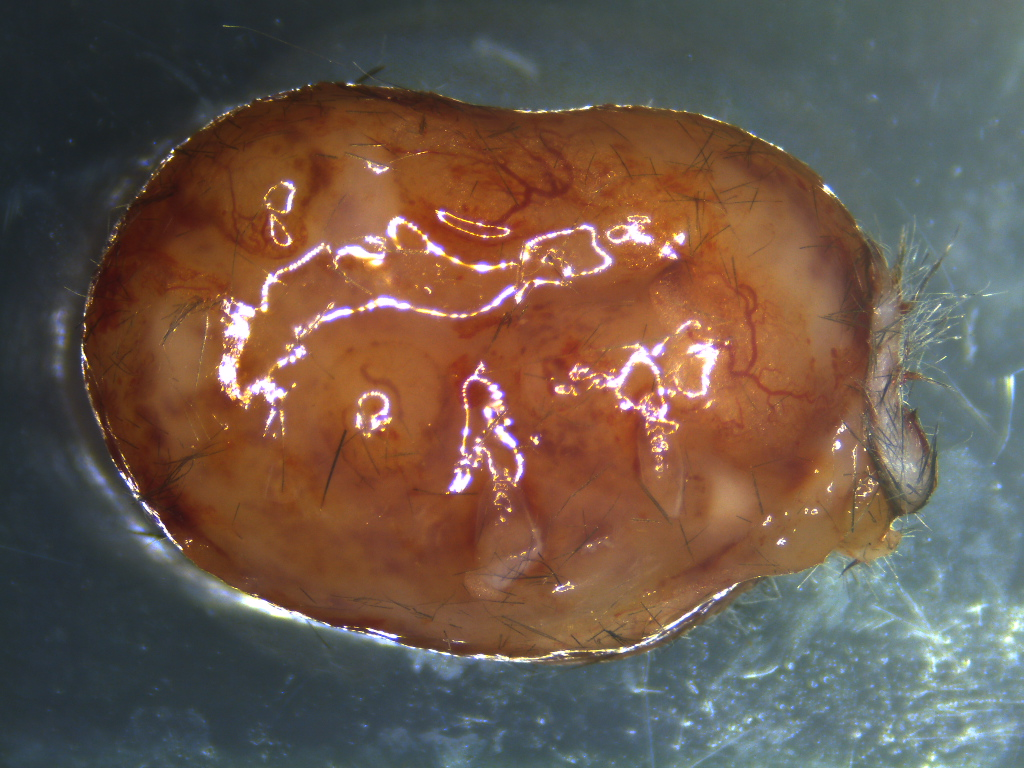

Supplement: Supplementary file 6 — Source data Fig. 6 [file 44318_2024_78_MOESM6_ESM.zip › Figure 6/6C/sGCCtr+Vehicle-2.tif]

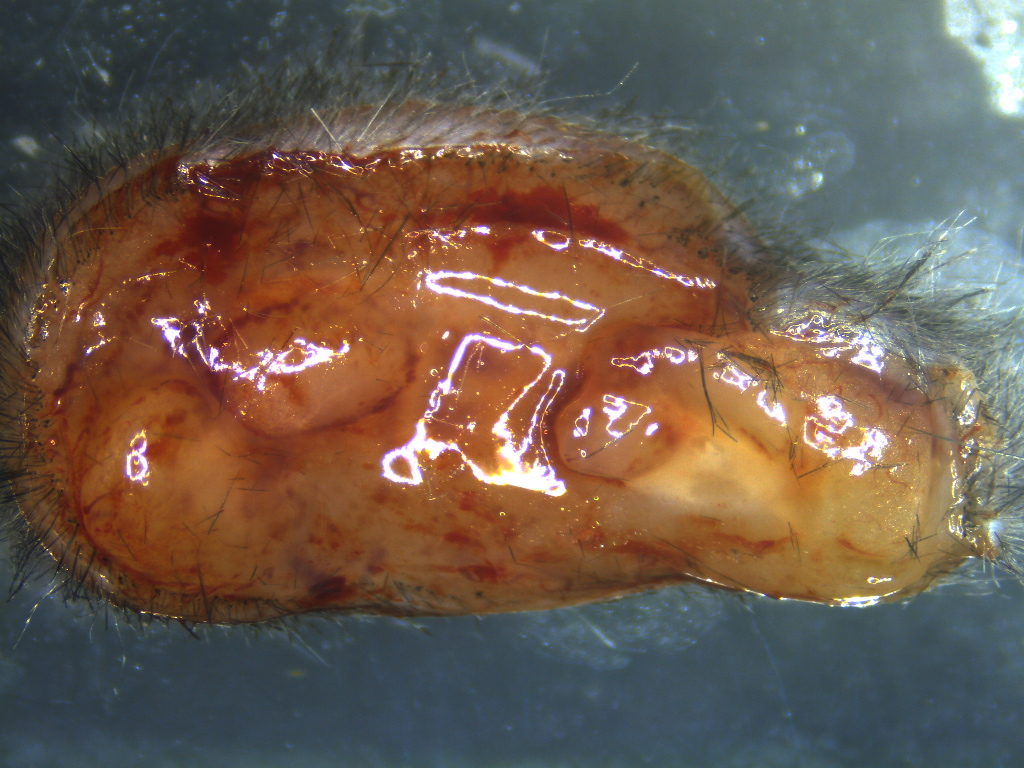

Supplement: Supplementary file 6 — Source data Fig. 6 [file 44318_2024_78_MOESM6_ESM.zip › Figure 6/6C/sGCCtr+Vehicle-3.tif]

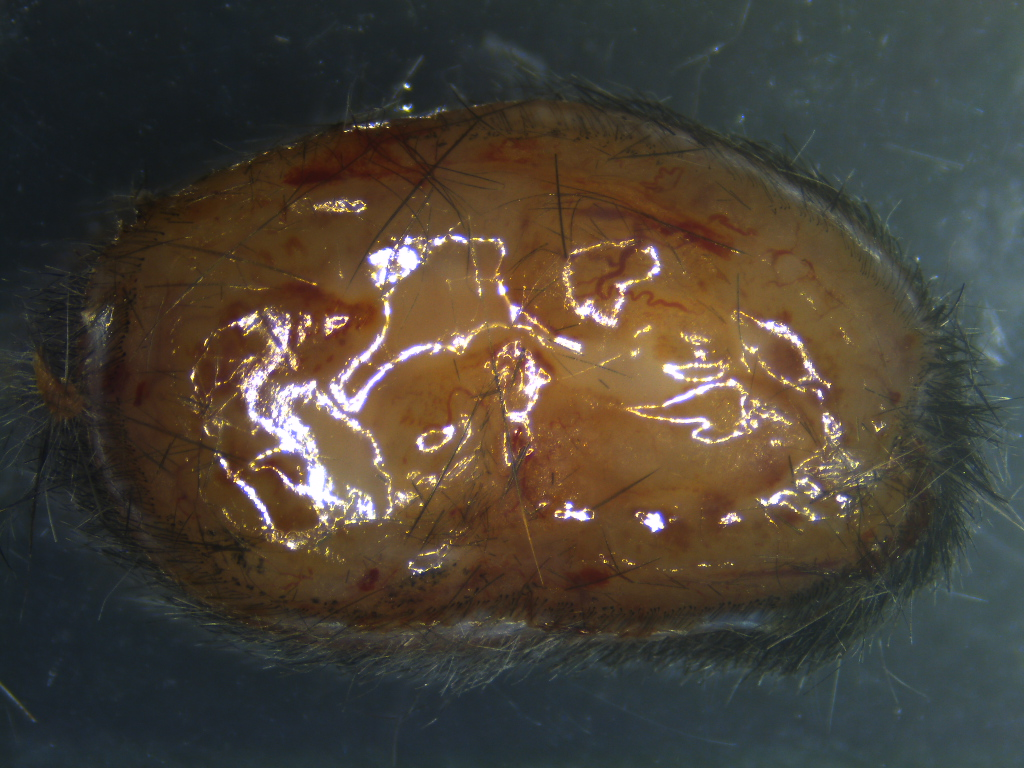

Supplement: Supplementary file 6 — Source data Fig. 6 [file 44318_2024_78_MOESM6_ESM.zip › Figure 6/6C/sGCCtr+Vehicle-1.tif]

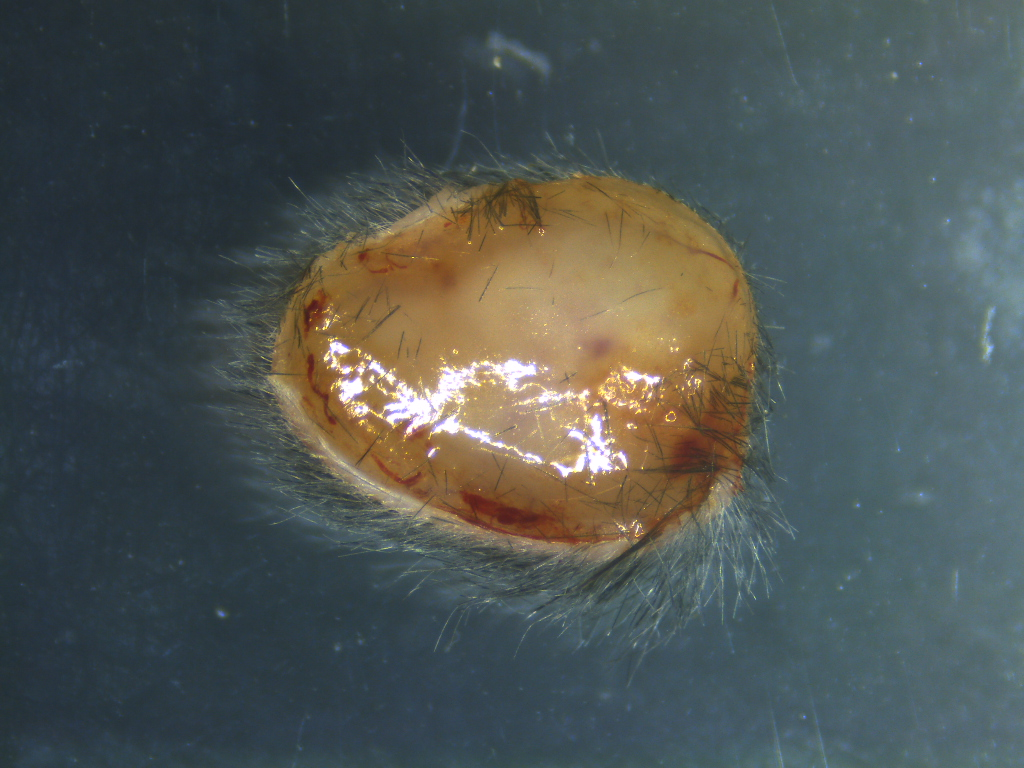

Supplement: Supplementary file 6 — Source data Fig. 6 [file 44318_2024_78_MOESM6_ESM.zip › Figure 6/6C/sGCCtr+Fruquintinib-7.tif]

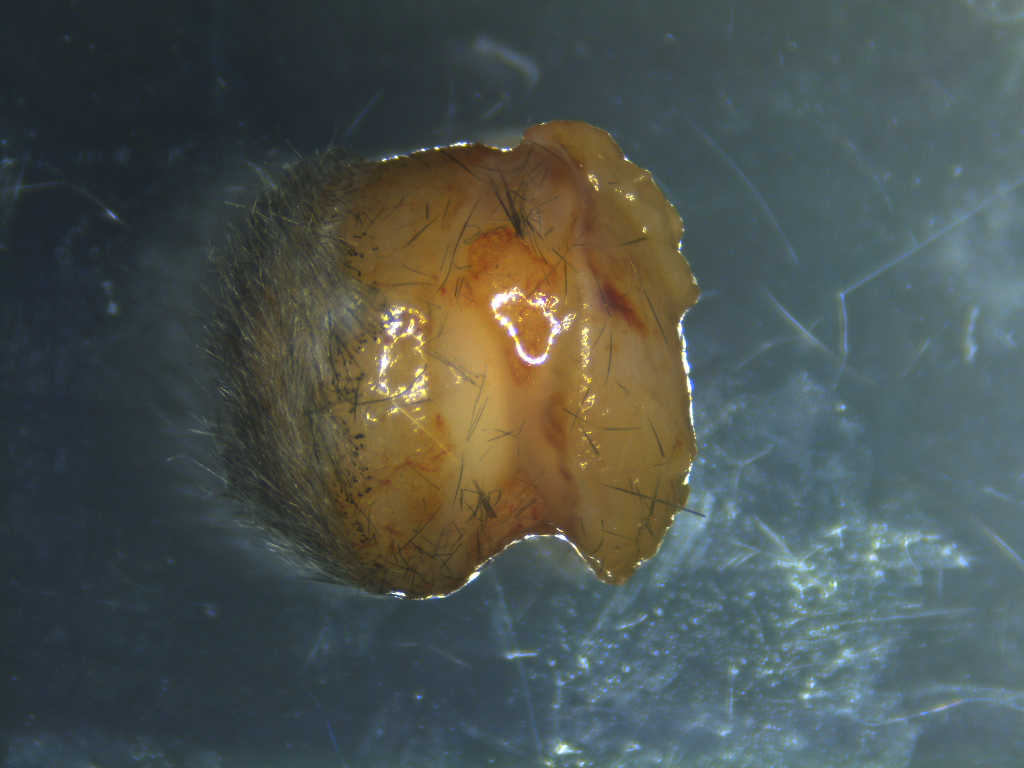

Supplement: Supplementary file 6 — Source data Fig. 6 [file 44318_2024_78_MOESM6_ESM.zip › Figure 6/6C/sGCCtr+Fruquintinib-6.tif]

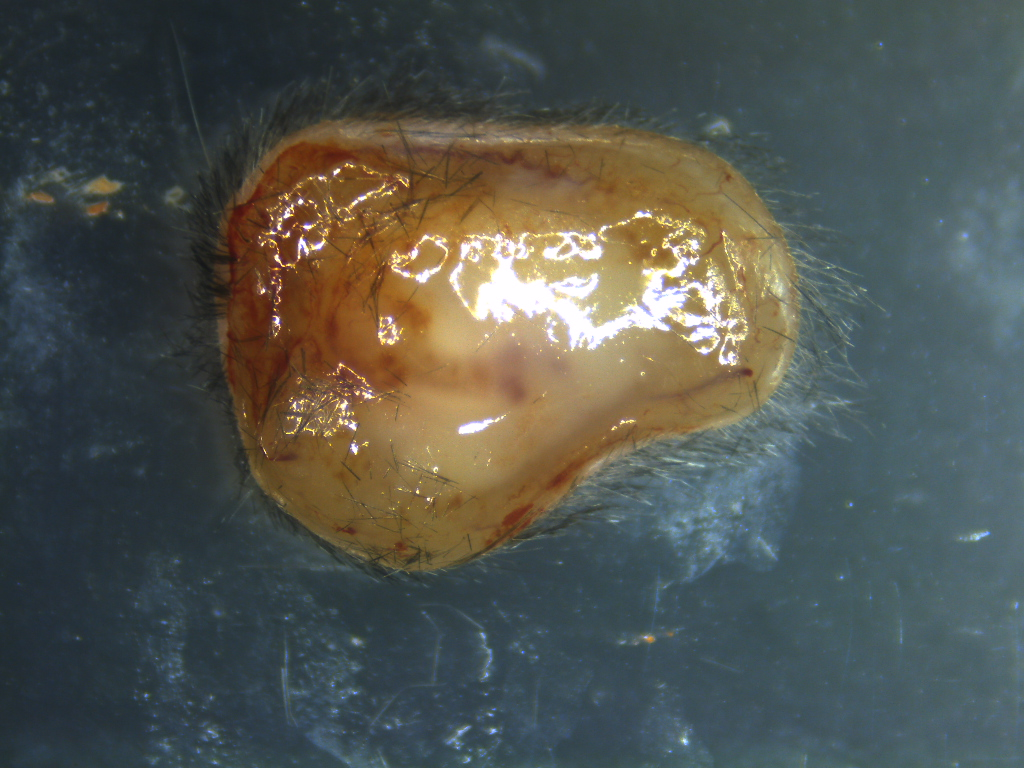

Supplement: Supplementary file 6 — Source data Fig. 6 [file 44318_2024_78_MOESM6_ESM.zip › Figure 6/6C/sGCCtr+Fruquintinib-4.tif]

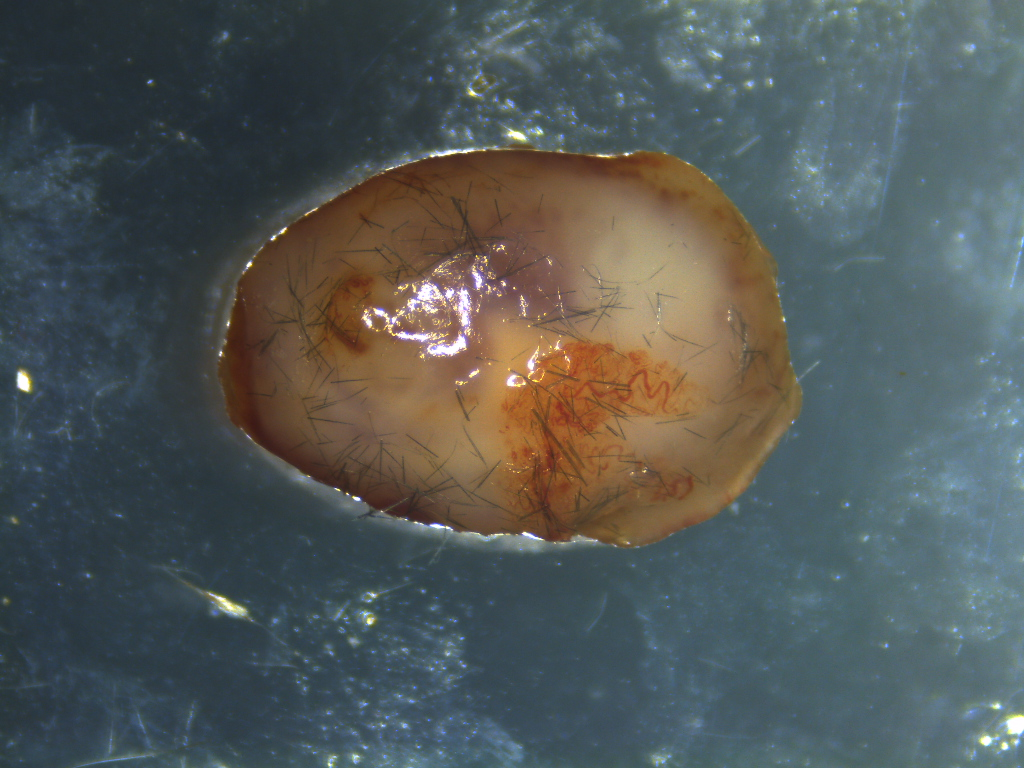

Supplement: Supplementary file 6 — Source data Fig. 6 [file 44318_2024_78_MOESM6_ESM.zip › Figure 6/6C/sGCCtr+Fruquintinib-5.tif]

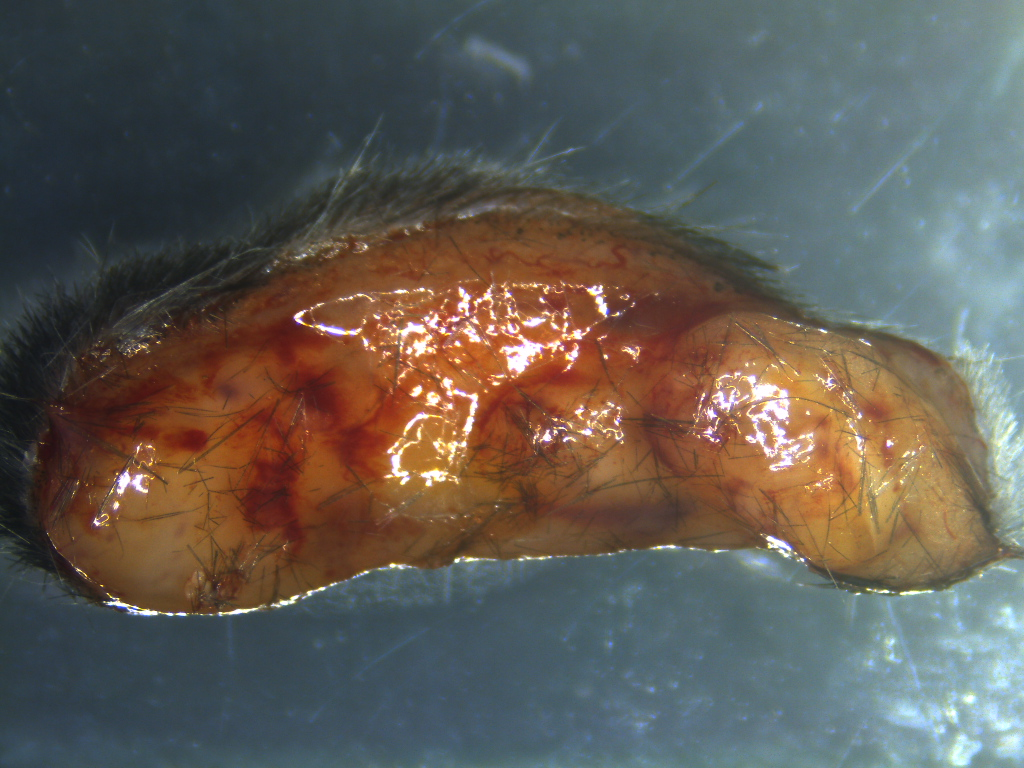

Supplement: Supplementary file 6 — Source data Fig. 6 [file 44318_2024_78_MOESM6_ESM.zip › Figure 6/6C/sGCCtr+Fruquintinib-1.tif]

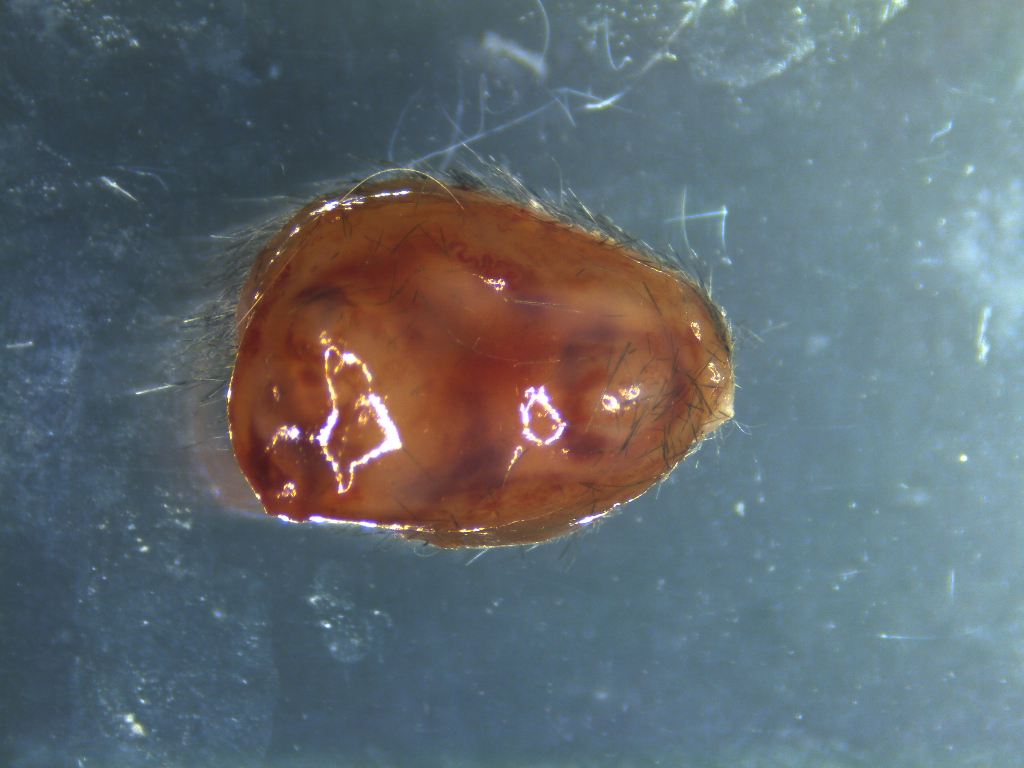

Supplement: Supplementary file 6 — Source data Fig. 6 [file 44318_2024_78_MOESM6_ESM.zip › Figure 6/6C/sGC╬öpc+Vehicle-8.tif]

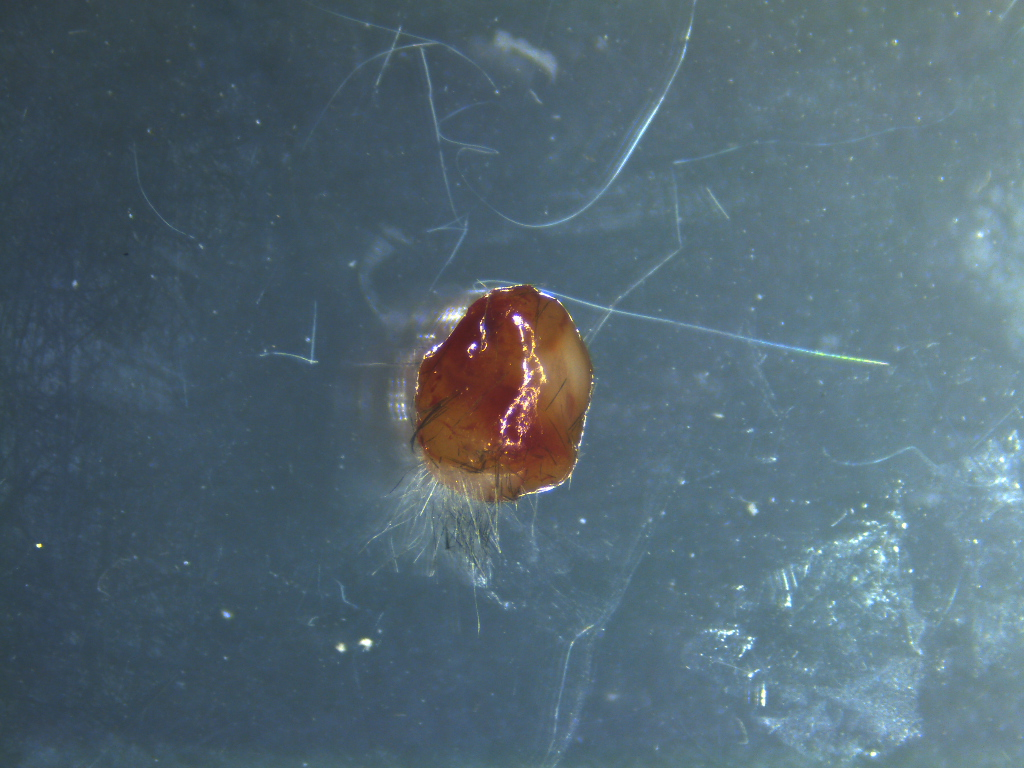

Supplement: Supplementary file 6 — Source data Fig. 6 [file 44318_2024_78_MOESM6_ESM.zip › Figure 6/6C/sGC╬öpc+Fruquintinib-8.tif]

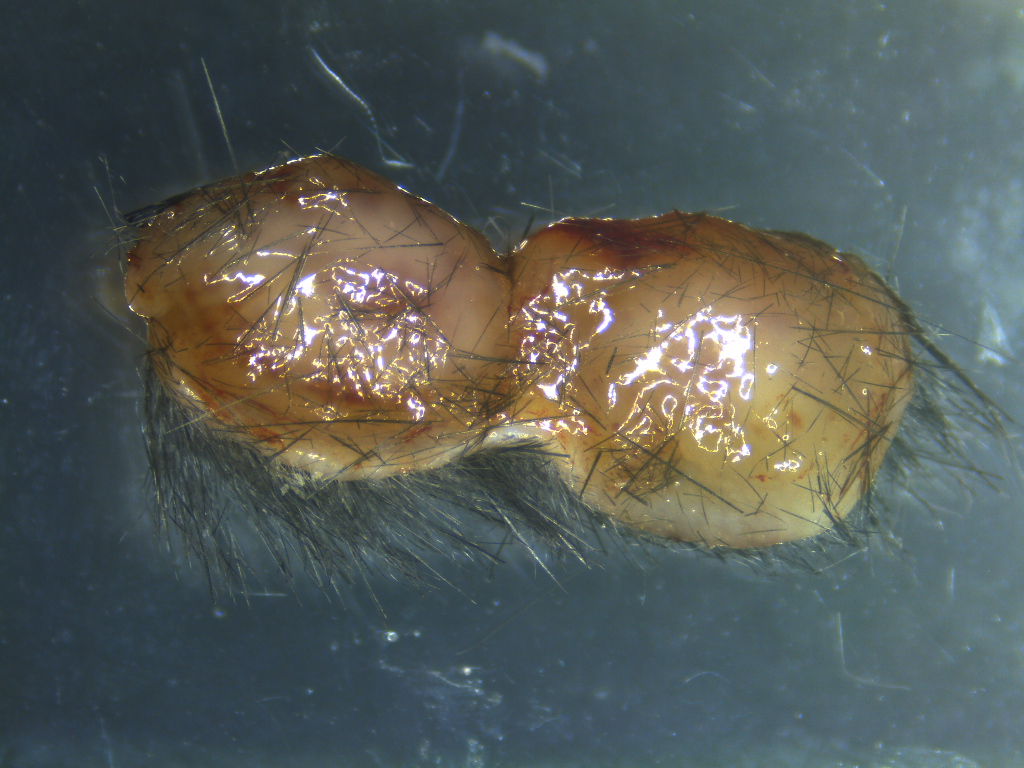

Supplement: Supplementary file 6 — Source data Fig. 6 [file 44318_2024_78_MOESM6_ESM.zip › Figure 6/6C/sGCCtr+Fruquintinib-2.tif]

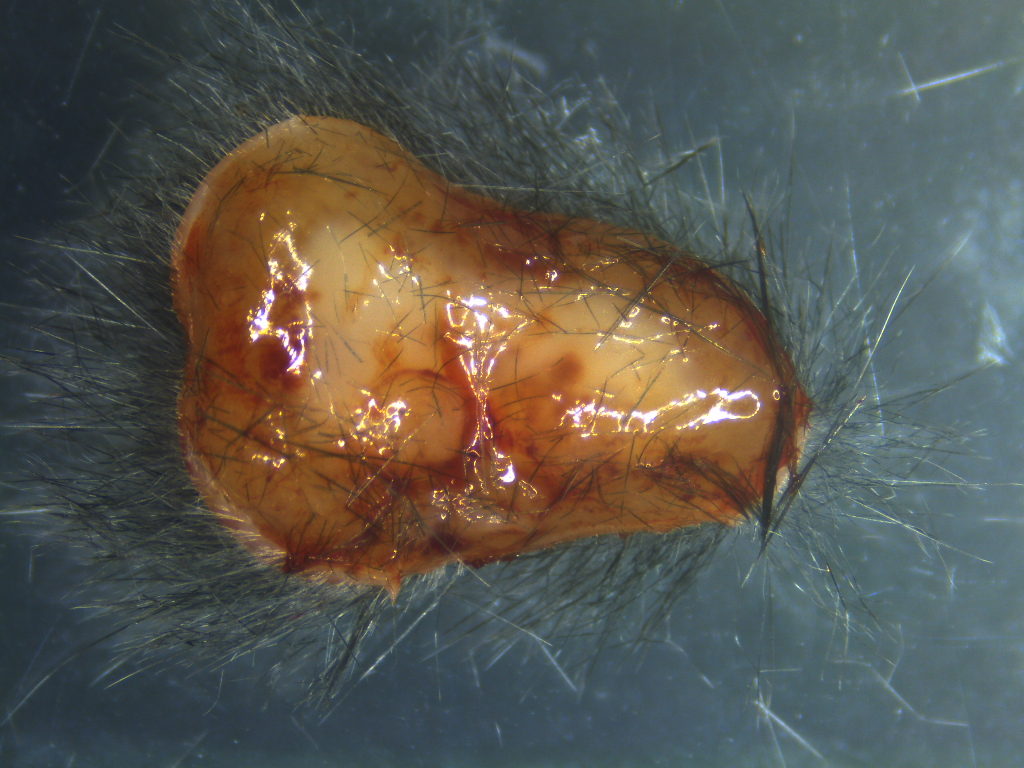

Supplement: Supplementary file 6 — Source data Fig. 6 [file 44318_2024_78_MOESM6_ESM.zip › Figure 6/6C/sGCCtr+Fruquintinib-3.tif]

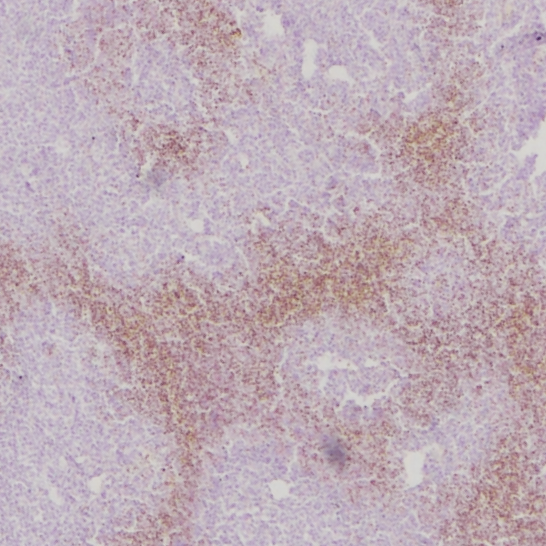

Supplement: Supplementary file 7 — EV Figure Source Data [file 44318_2024_78_MOESM7_ESM.zip › Expanded View/Expanded View 4/EV4F/sGCCtr-2.tif]

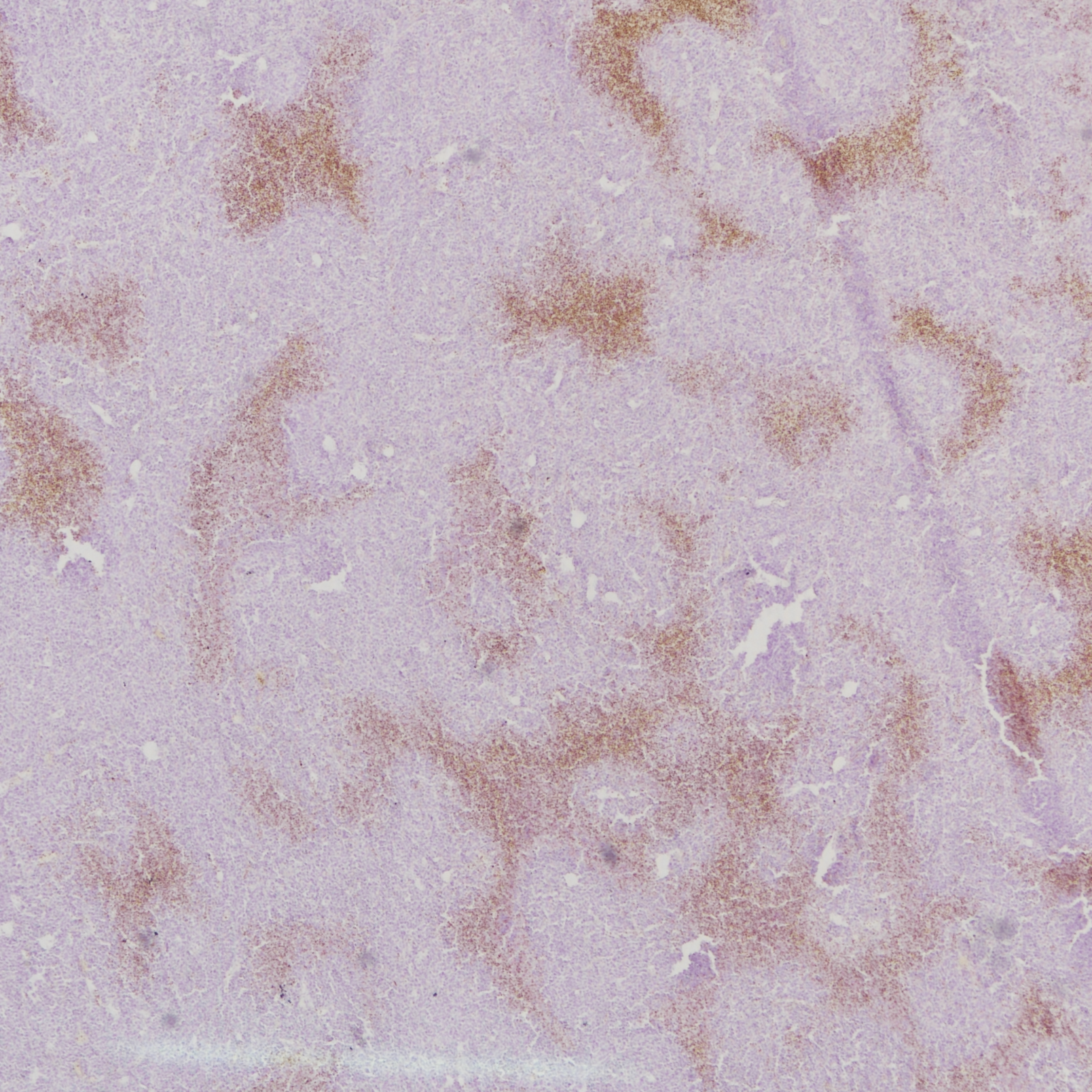

Supplement: Supplementary file 7 — EV Figure Source Data [file 44318_2024_78_MOESM7_ESM.zip › Expanded View/Expanded View 4/EV4F/sGCCtr-1.tif]

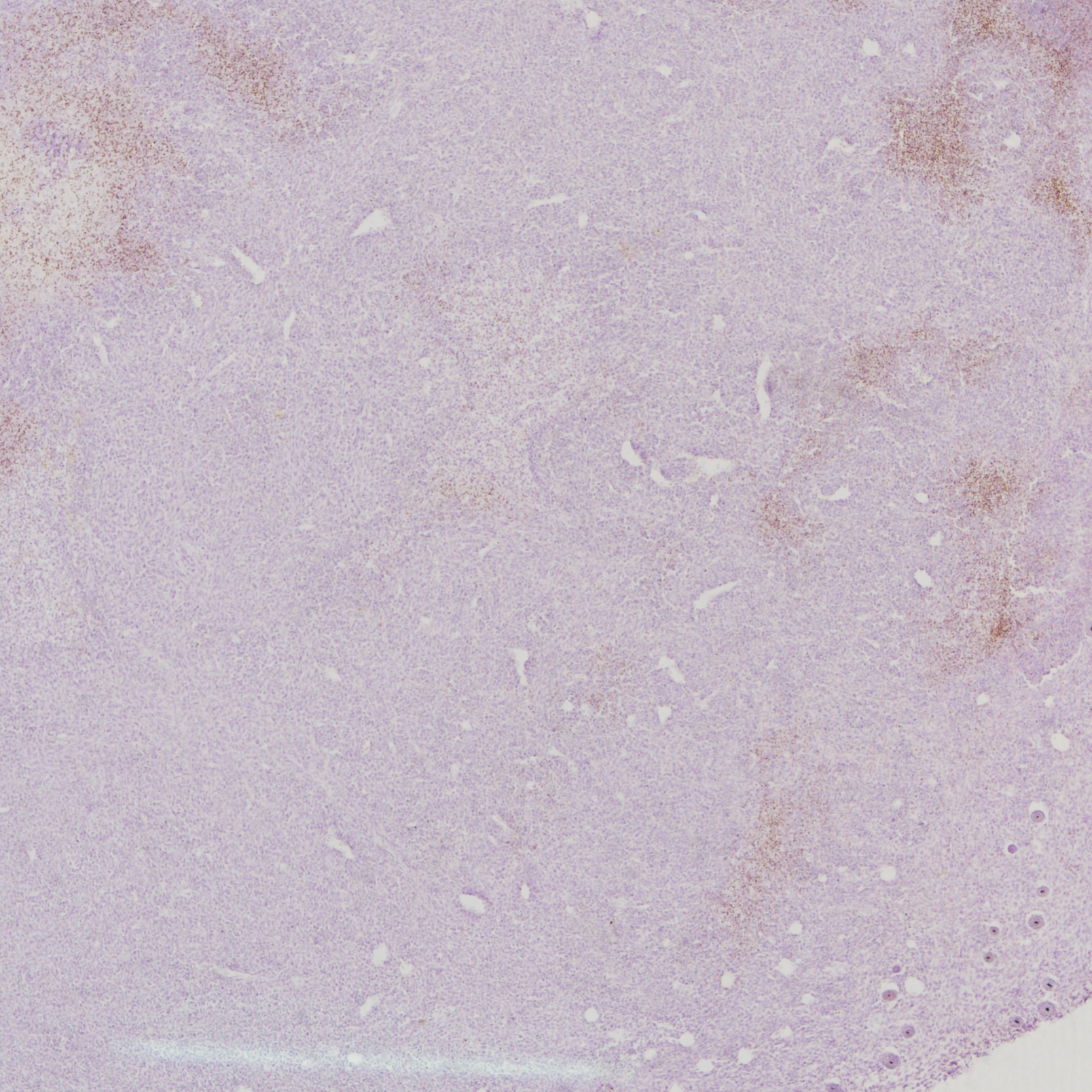

Supplement: Supplementary file 7 — EV Figure Source Data [file 44318_2024_78_MOESM7_ESM.zip › Expanded View/Expanded View 4/EV4F/sGC╬öpc-1.tif]

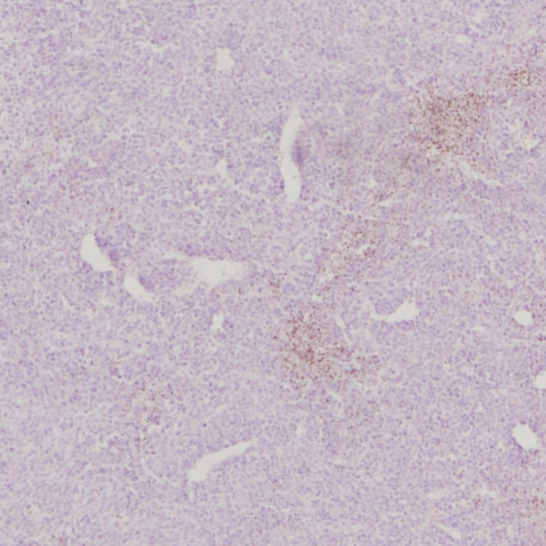

Supplement: Supplementary file 7 — EV Figure Source Data [file 44318_2024_78_MOESM7_ESM.zip › Expanded View/Expanded View 4/EV4F/sGC╬öpc-2.tif]

Original blot of EV3 E

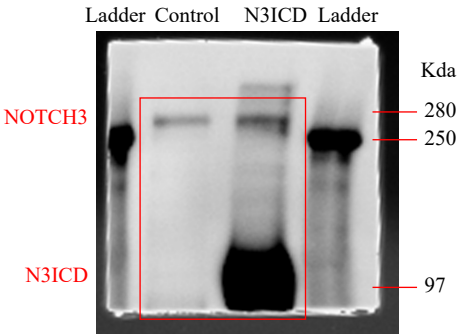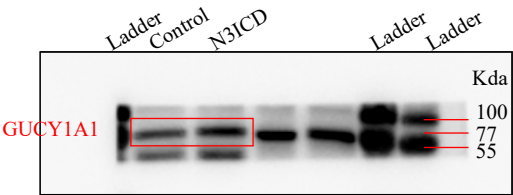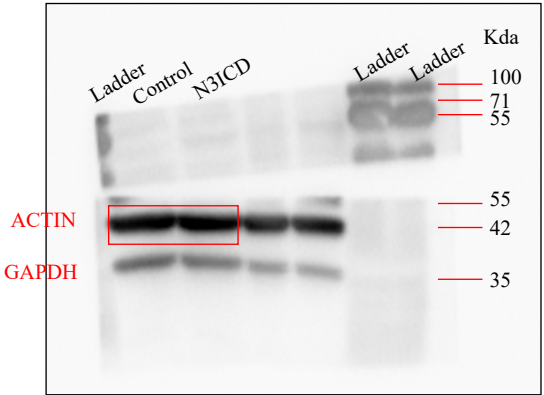

Long time exposure

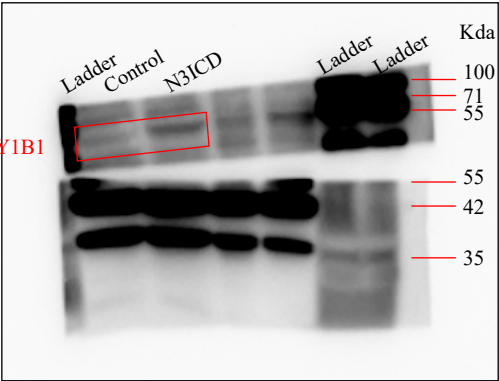

Supplement: Supplementary file 7 — EV Figure Source Data [file 44318_2024_78_MOESM7_ESM.zip › Expanded View/Expanded View 3/EV3E/WB.pdf]

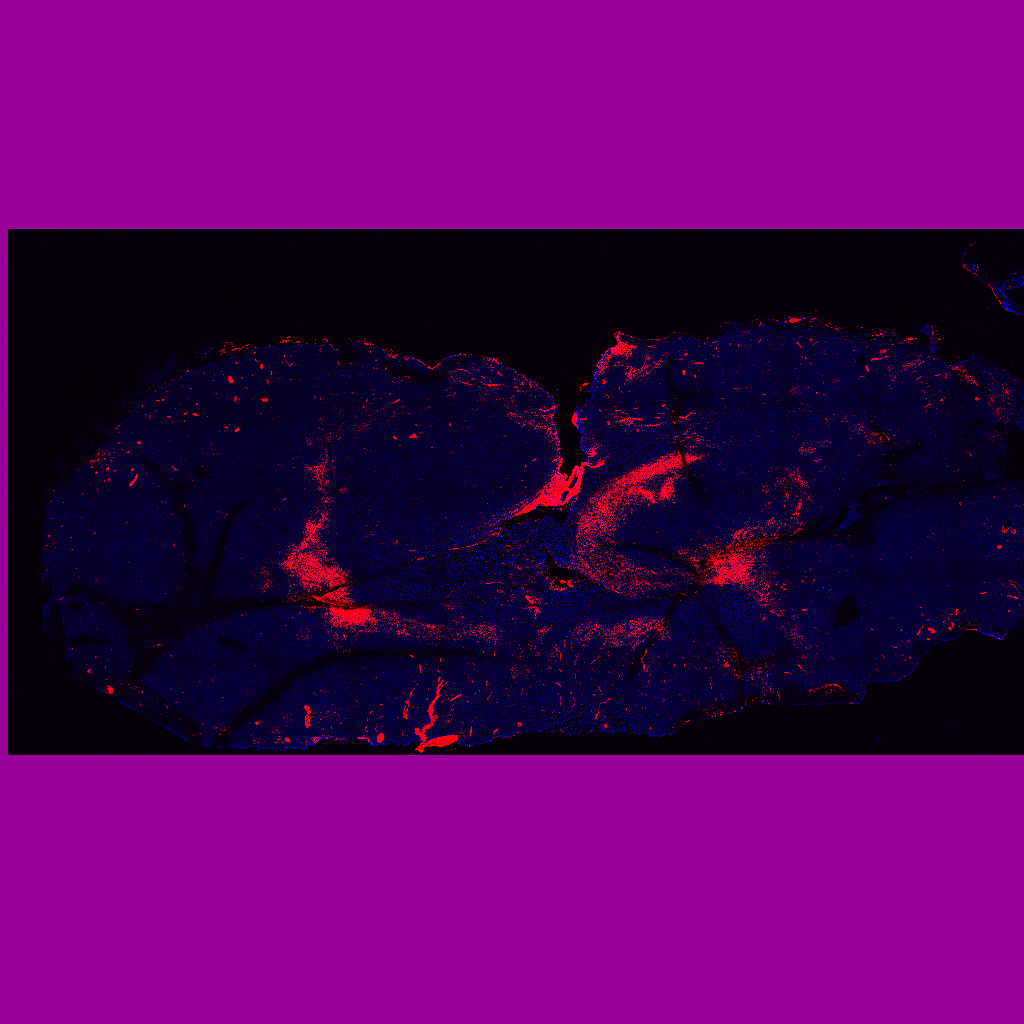

Supplement: Supplementary file 7 — EV Figure Source Data [file 44318_2024_78_MOESM7_ESM.zip › Expanded View/Expanded View 2/EV2B/sGCCtr-2.tif]

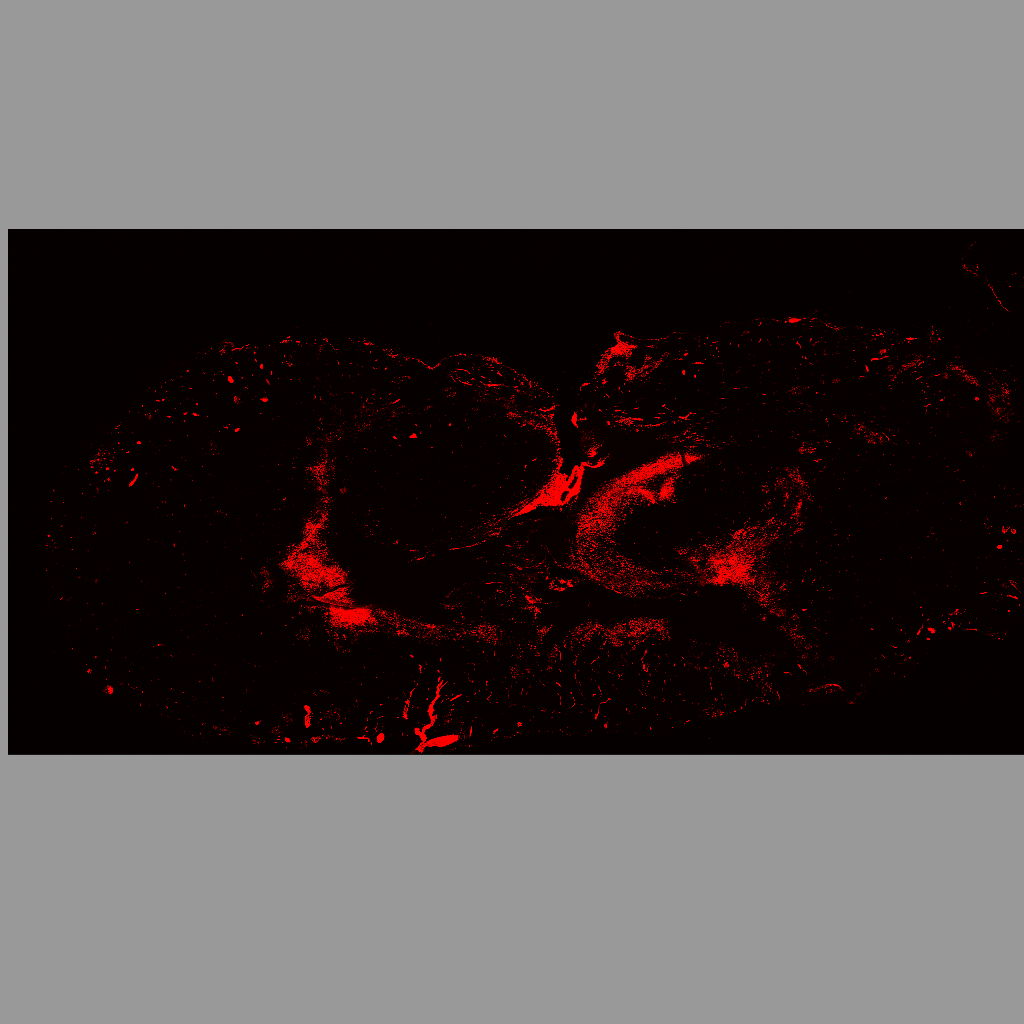

Supplement: Supplementary file 7 — EV Figure Source Data [file 44318_2024_78_MOESM7_ESM.zip › Expanded View/Expanded View 2/EV2B/sGCCtr-1.tif]

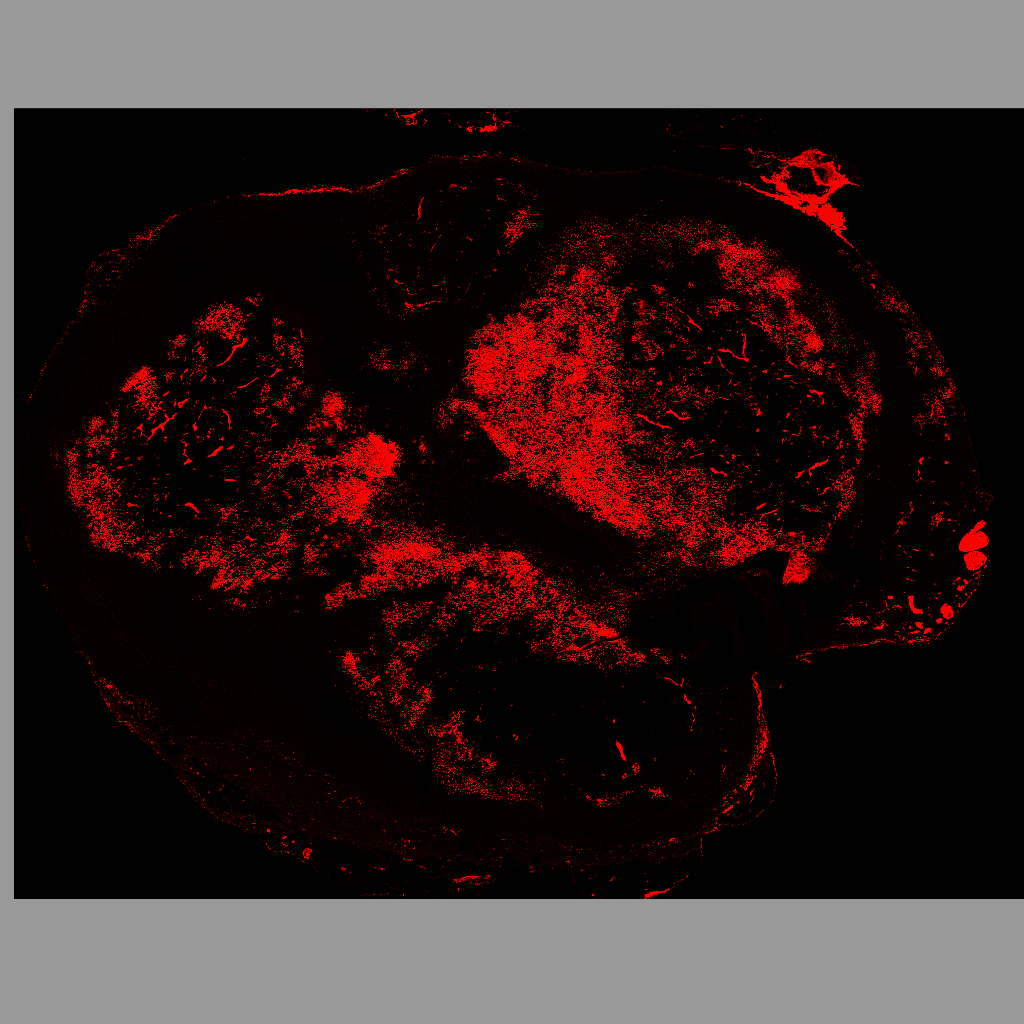

Supplement: Supplementary file 7 — EV Figure Source Data [file 44318_2024_78_MOESM7_ESM.zip › Expanded View/Expanded View 2/EV2B/sGC╬öpc-1.tif]

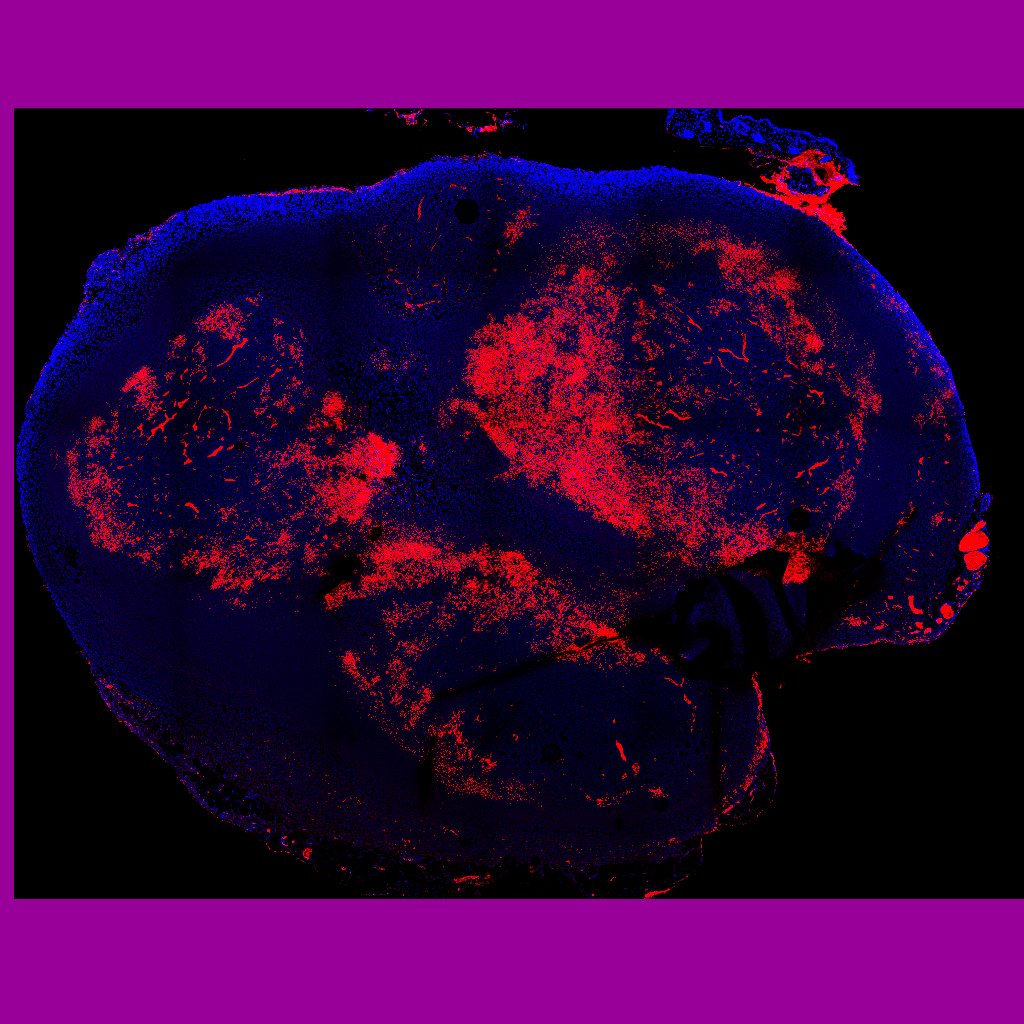

Supplement: Supplementary file 7 — EV Figure Source Data [file 44318_2024_78_MOESM7_ESM.zip › Expanded View/Expanded View 2/EV2B/sGC╬öpc-2.tif]

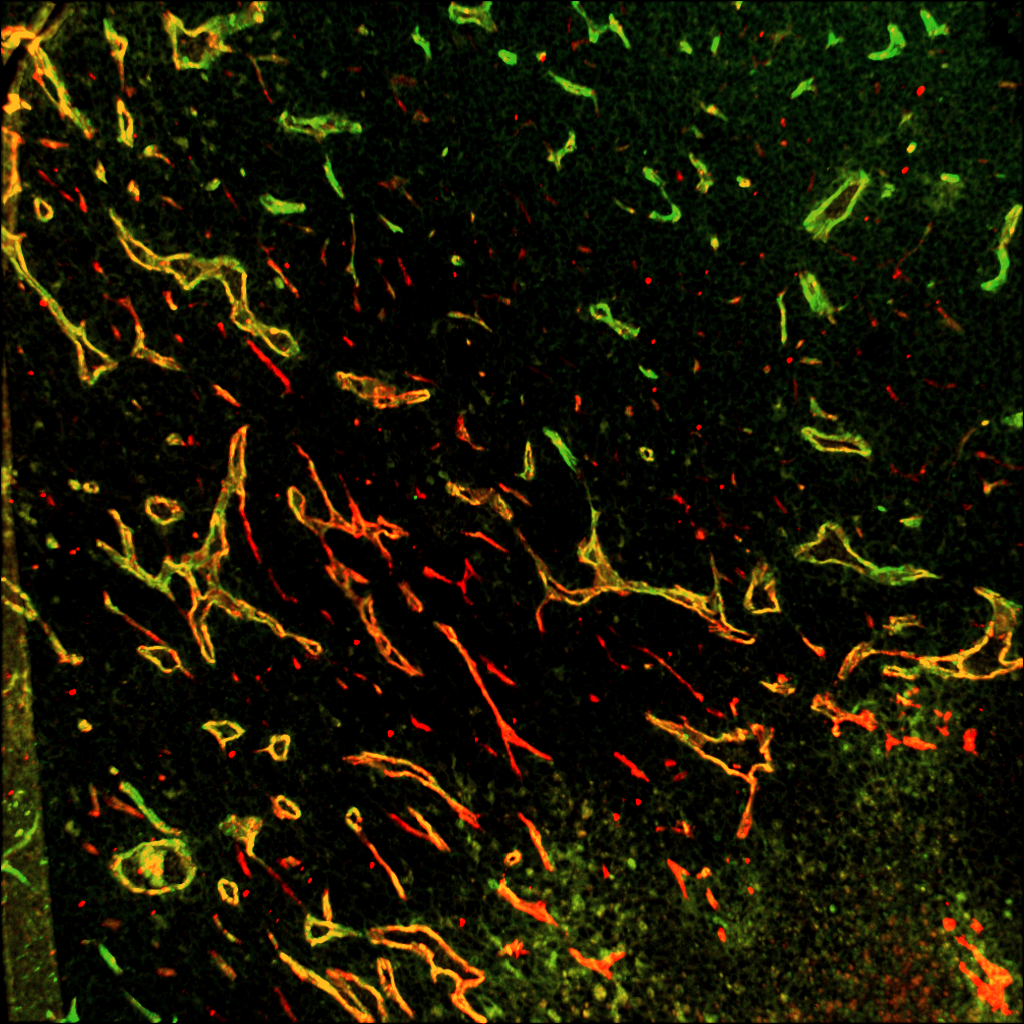

Supplement: Supplementary file 7 — EV Figure Source Data [file 44318_2024_78_MOESM7_ESM.zip › Expanded View/Expanded View 2/EV2C/sGCCtr-4.tif]

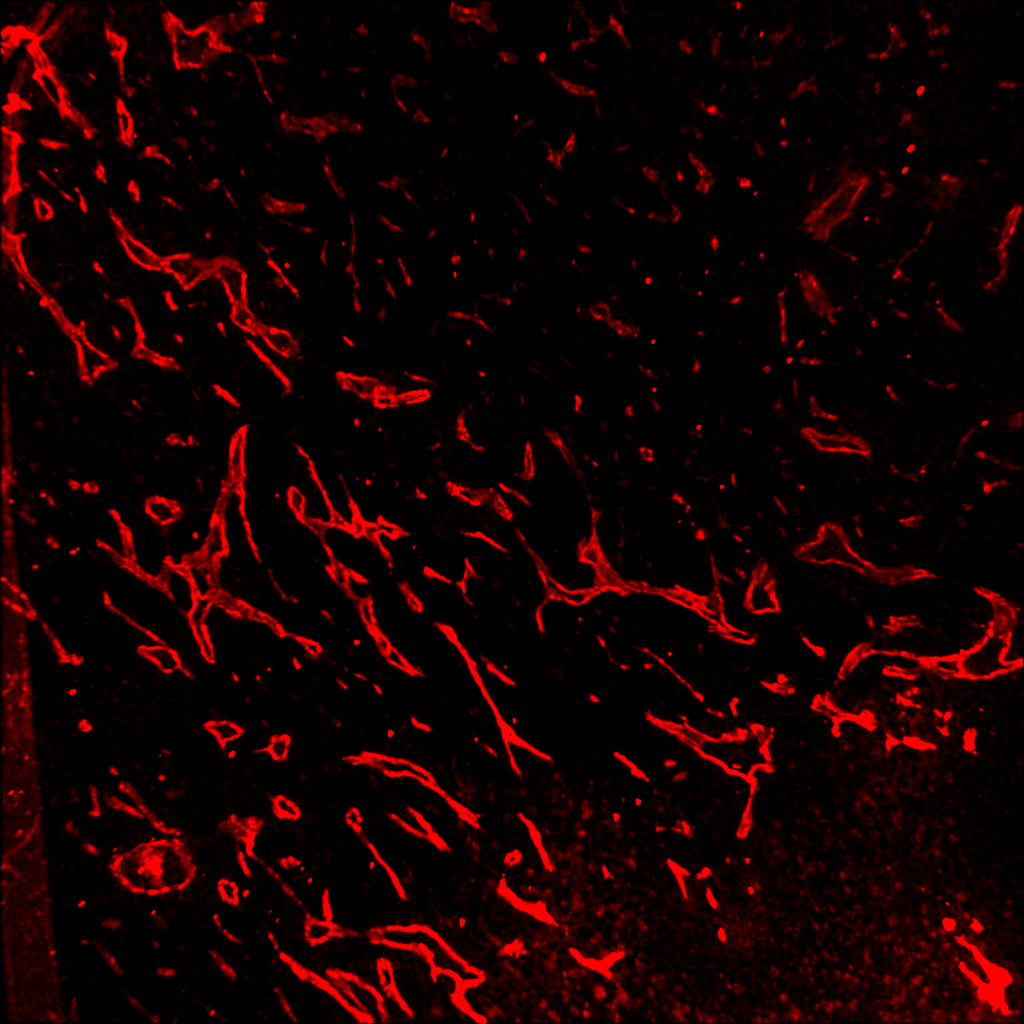

Supplement: Supplementary file 7 — EV Figure Source Data [file 44318_2024_78_MOESM7_ESM.zip › Expanded View/Expanded View 2/EV2C/sGCCtr-3.tif]

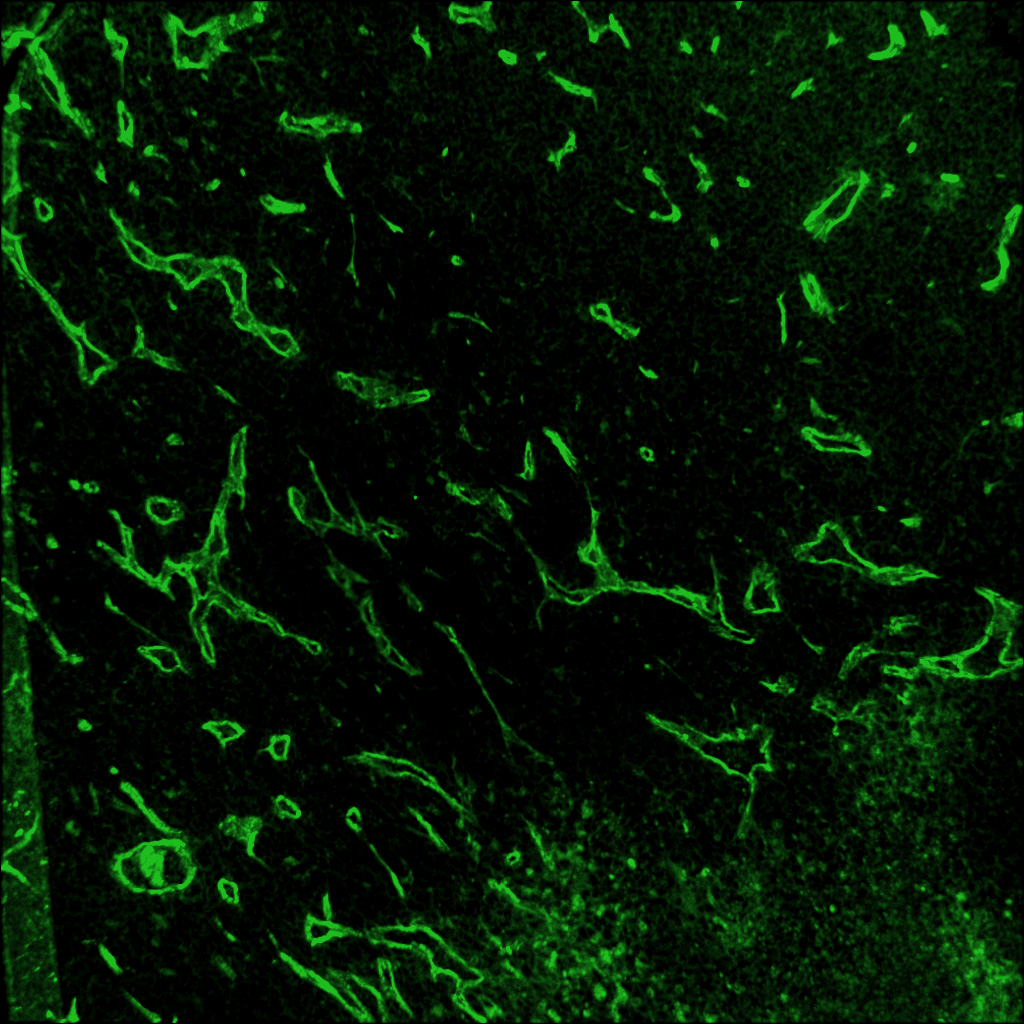

Supplement: Supplementary file 7 — EV Figure Source Data [file 44318_2024_78_MOESM7_ESM.zip › Expanded View/Expanded View 2/EV2C/sGCCtr-2.tif]

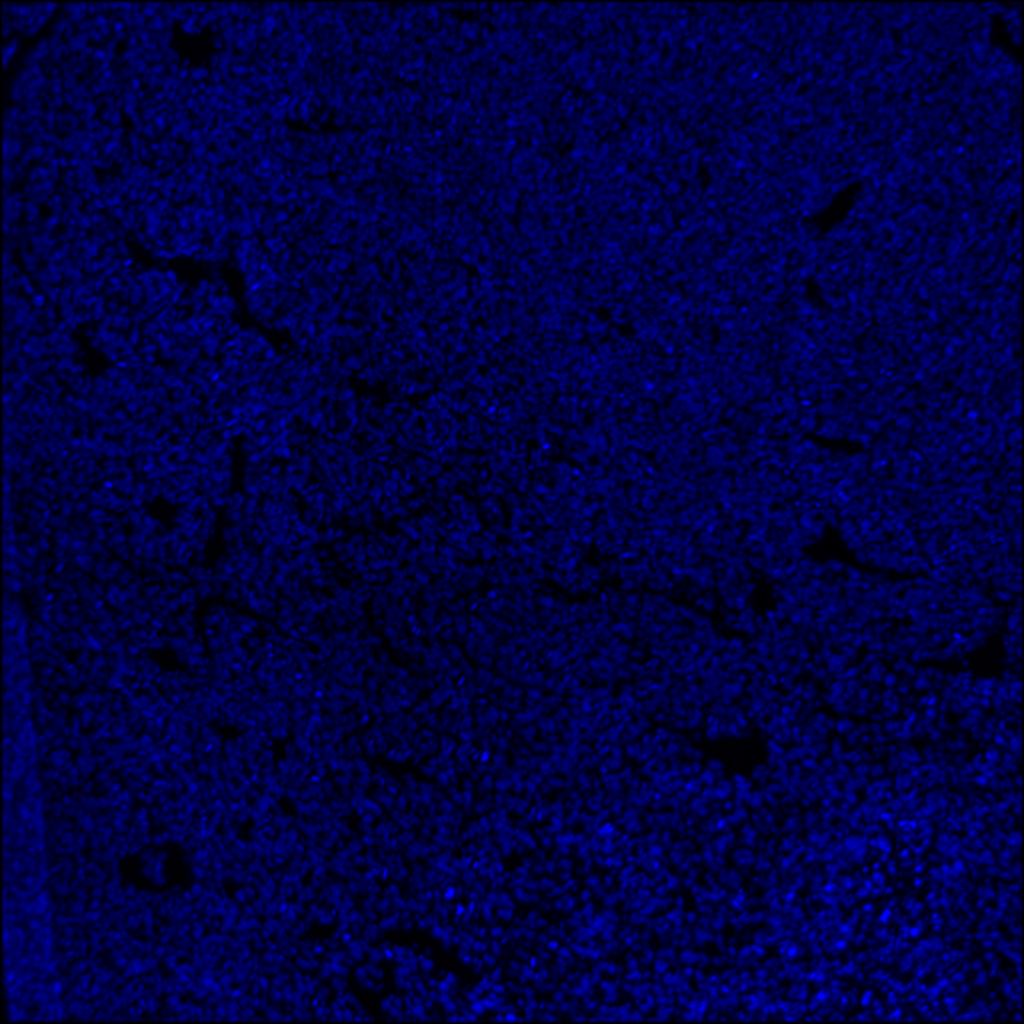

Supplement: Supplementary file 7 — EV Figure Source Data [file 44318_2024_78_MOESM7_ESM.zip › Expanded View/Expanded View 2/EV2C/sGCCtr-1.tif]

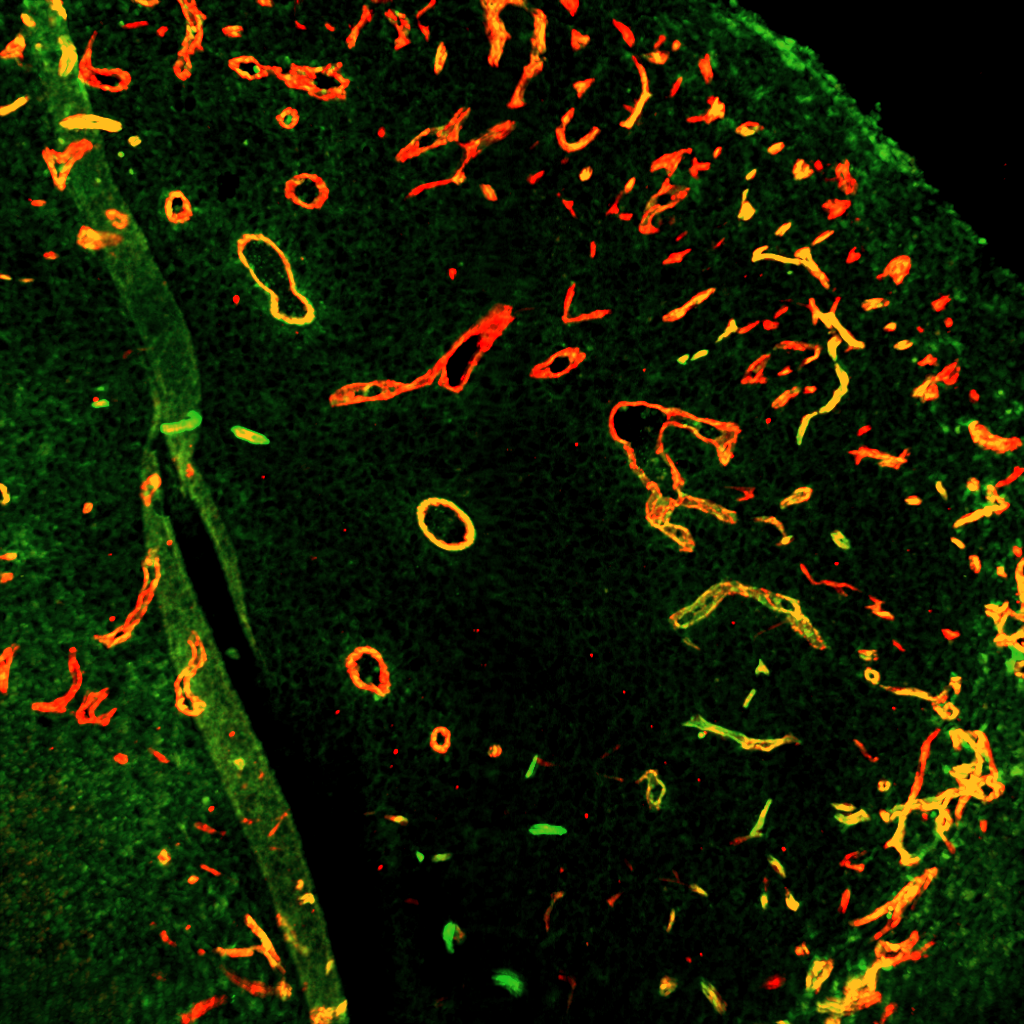

Supplement: Supplementary file 7 — EV Figure Source Data [file 44318_2024_78_MOESM7_ESM.zip › Expanded View/Expanded View 2/EV2C/sGC╬öpc-4.tif]

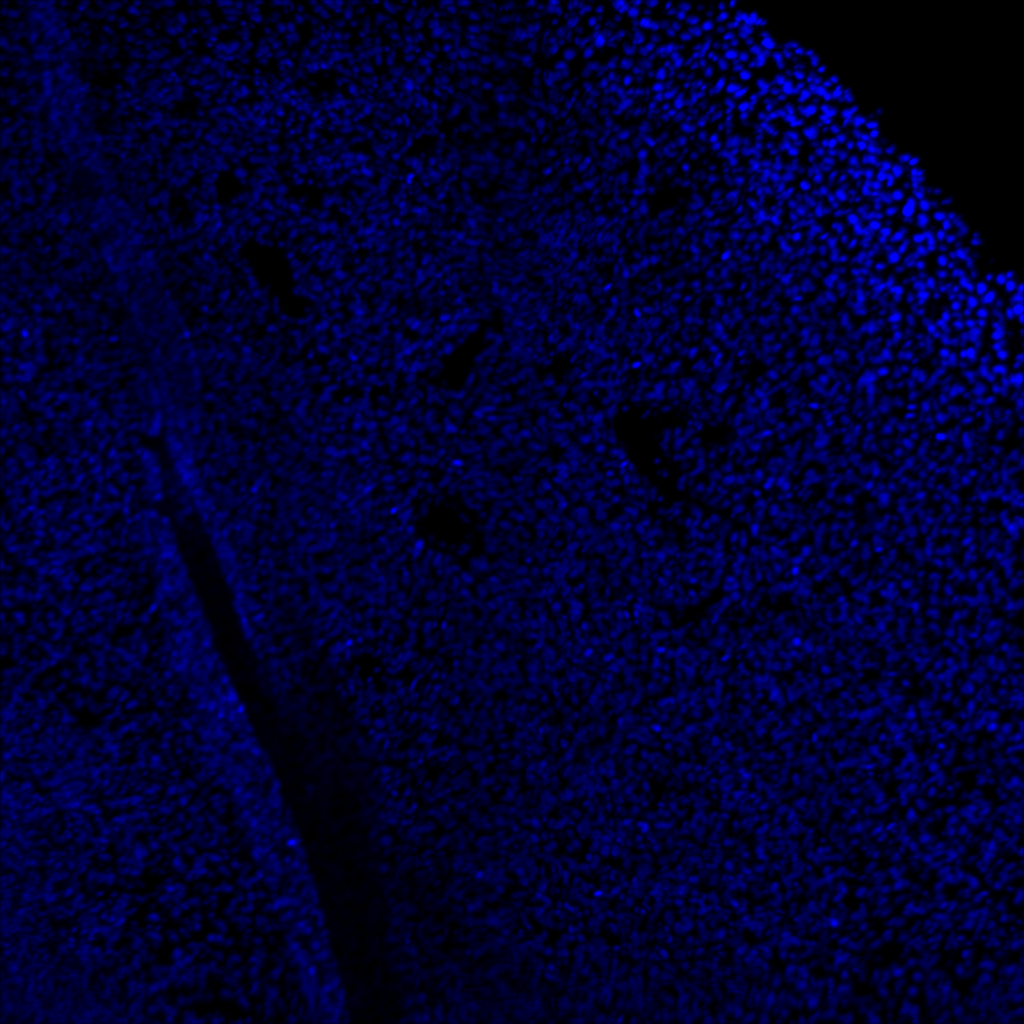

Supplement: Supplementary file 7 — EV Figure Source Data [file 44318_2024_78_MOESM7_ESM.zip › Expanded View/Expanded View 2/EV2C/sGC╬öpc-1.tif]

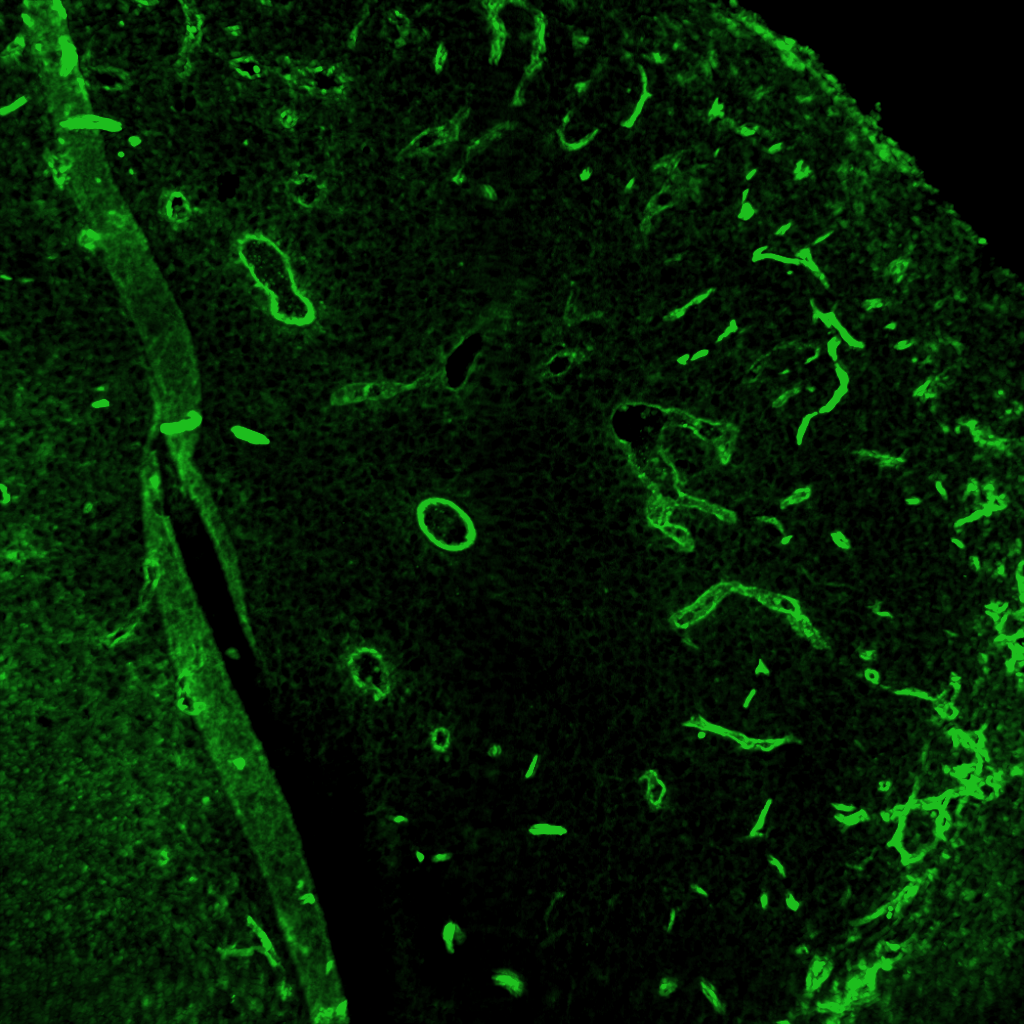

Supplement: Supplementary file 7 — EV Figure Source Data [file 44318_2024_78_MOESM7_ESM.zip › Expanded View/Expanded View 2/EV2C/sGC╬öpc-2.tif]

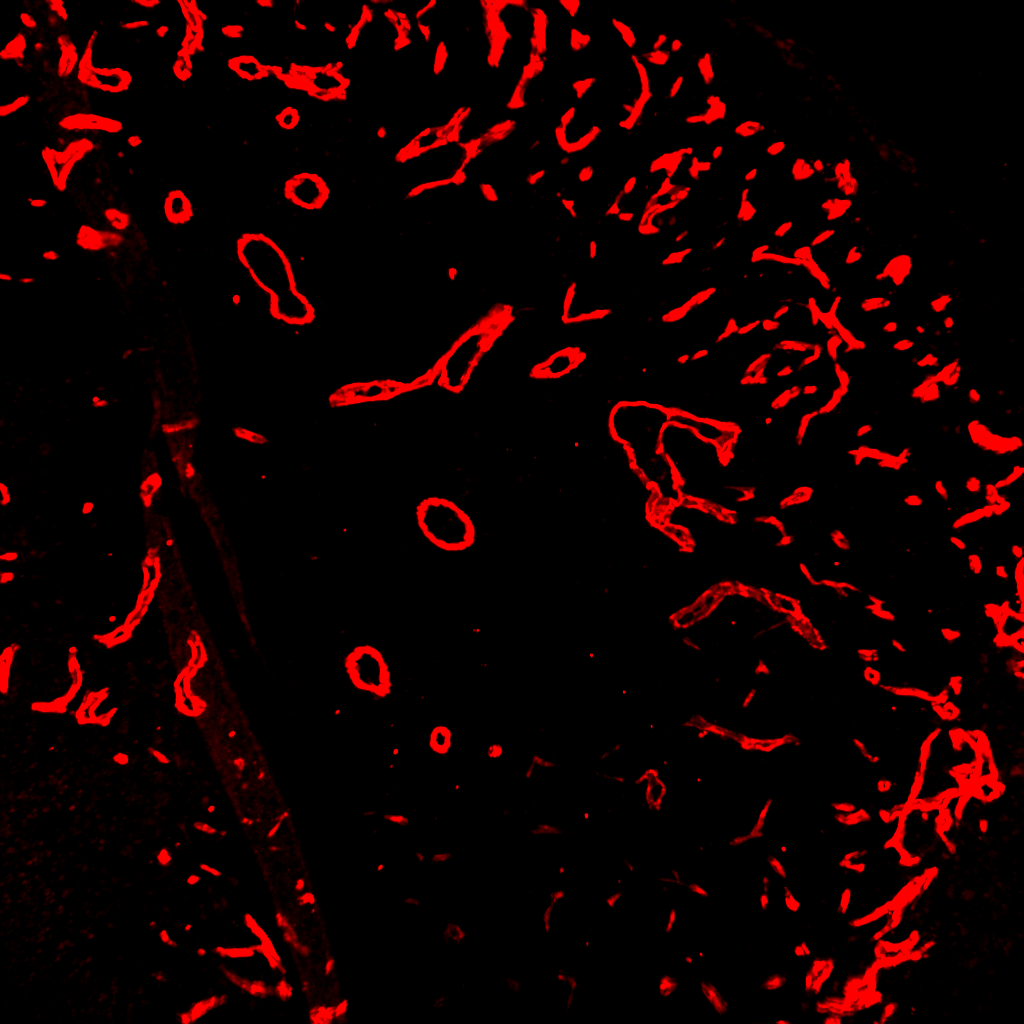

Supplement: Supplementary file 7 — EV Figure Source Data [file 44318_2024_78_MOESM7_ESM.zip › Expanded View/Expanded View 2/EV2C/sGC╬öpc-3.tif]

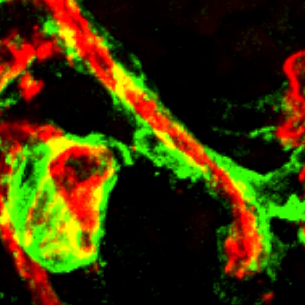

Supplement: Supplementary file 7 — EV Figure Source Data [file 44318_2024_78_MOESM7_ESM.zip › Expanded View/Expanded View 2/EV2A/sGCCtr-4.tif]

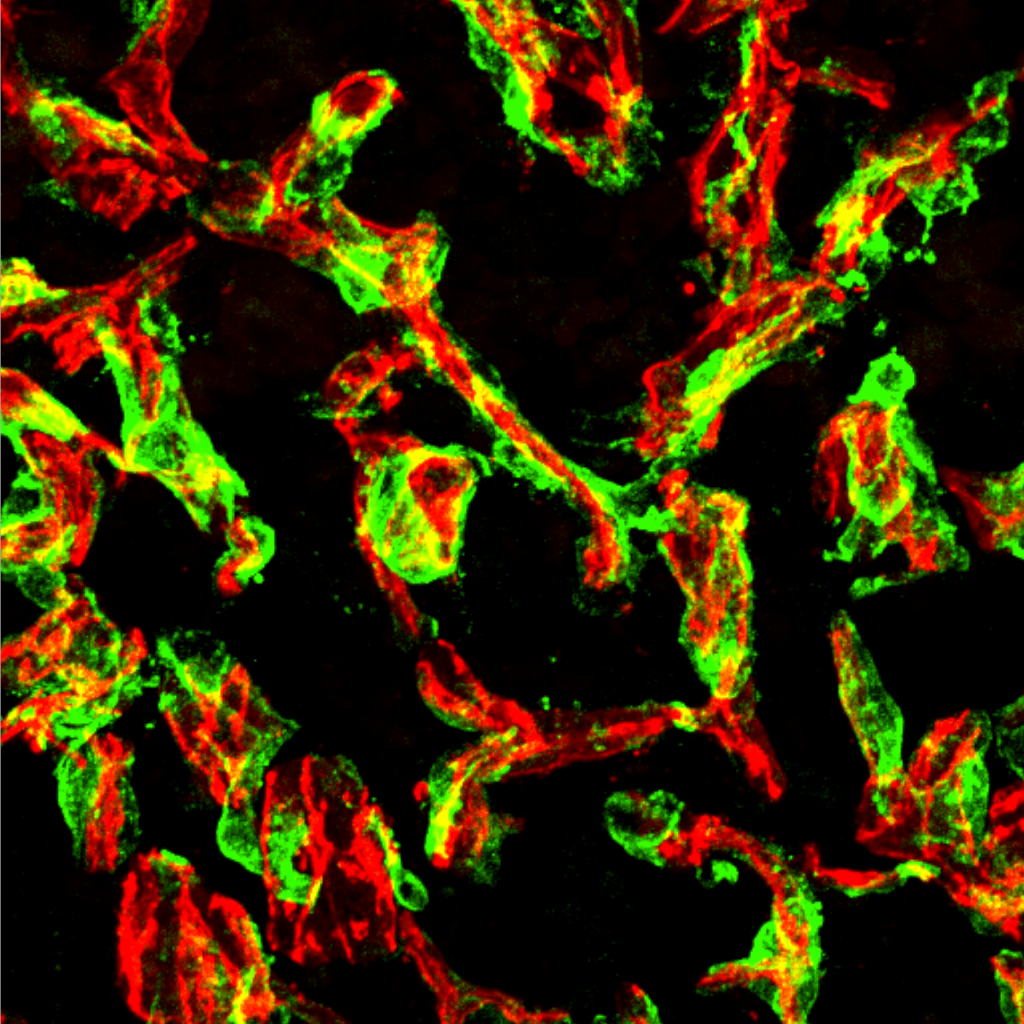

Supplement: Supplementary file 7 — EV Figure Source Data [file 44318_2024_78_MOESM7_ESM.zip › Expanded View/Expanded View 2/EV2A/sGCCtr-3.tif]

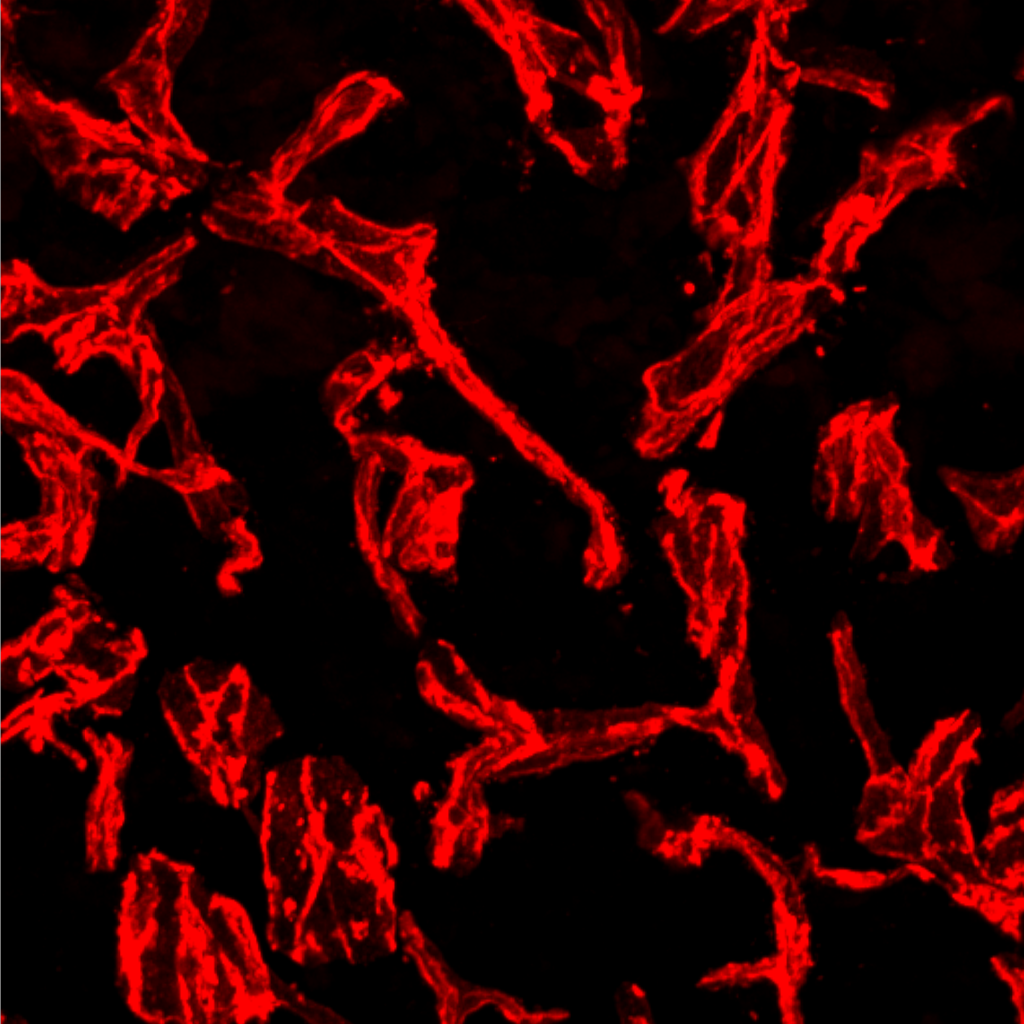

Supplement: Supplementary file 7 — EV Figure Source Data [file 44318_2024_78_MOESM7_ESM.zip › Expanded View/Expanded View 2/EV2A/sGCCtr-2.tif]

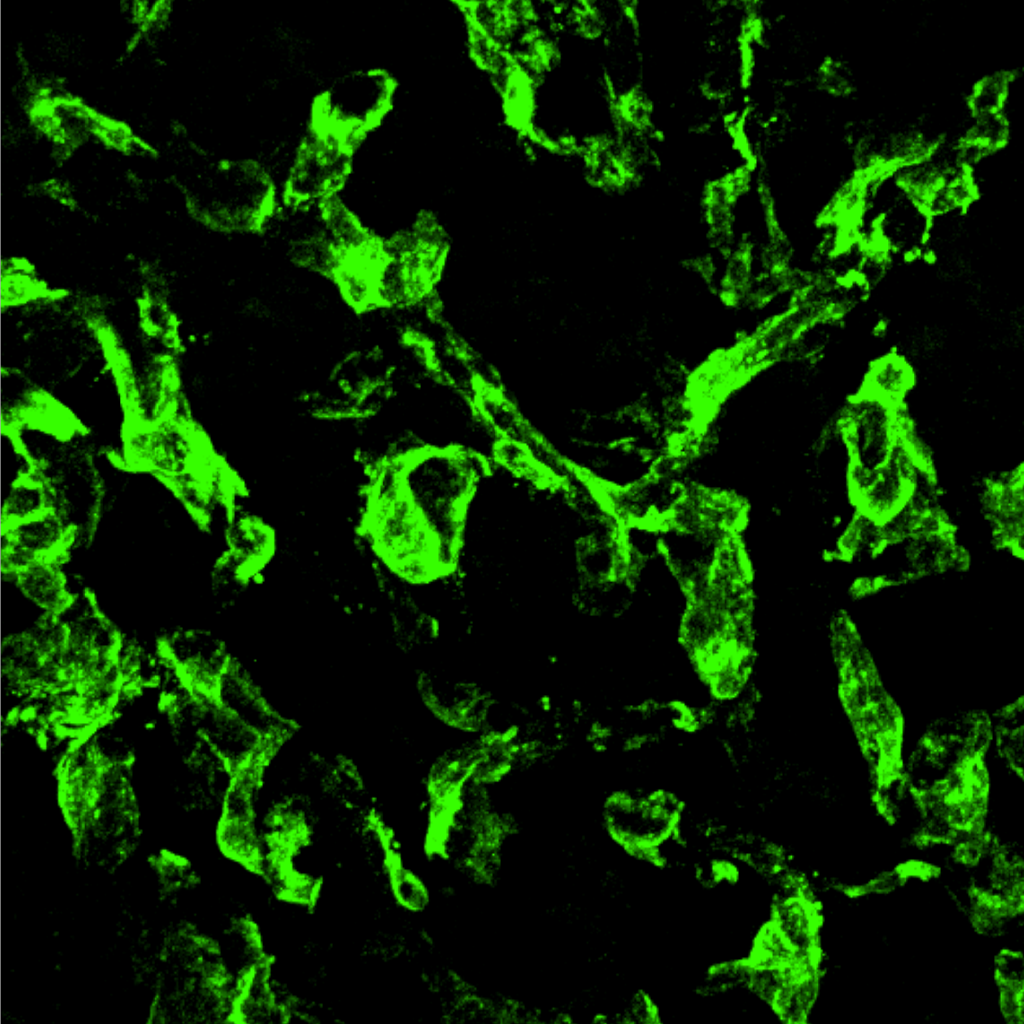

Supplement: Supplementary file 7 — EV Figure Source Data [file 44318_2024_78_MOESM7_ESM.zip › Expanded View/Expanded View 2/EV2A/sGCCtr-1.tif]

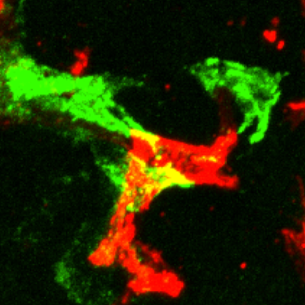

Supplement: Supplementary file 7 — EV Figure Source Data [file 44318_2024_78_MOESM7_ESM.zip › Expanded View/Expanded View 2/EV2A/sGC╬öpc-4.tif]

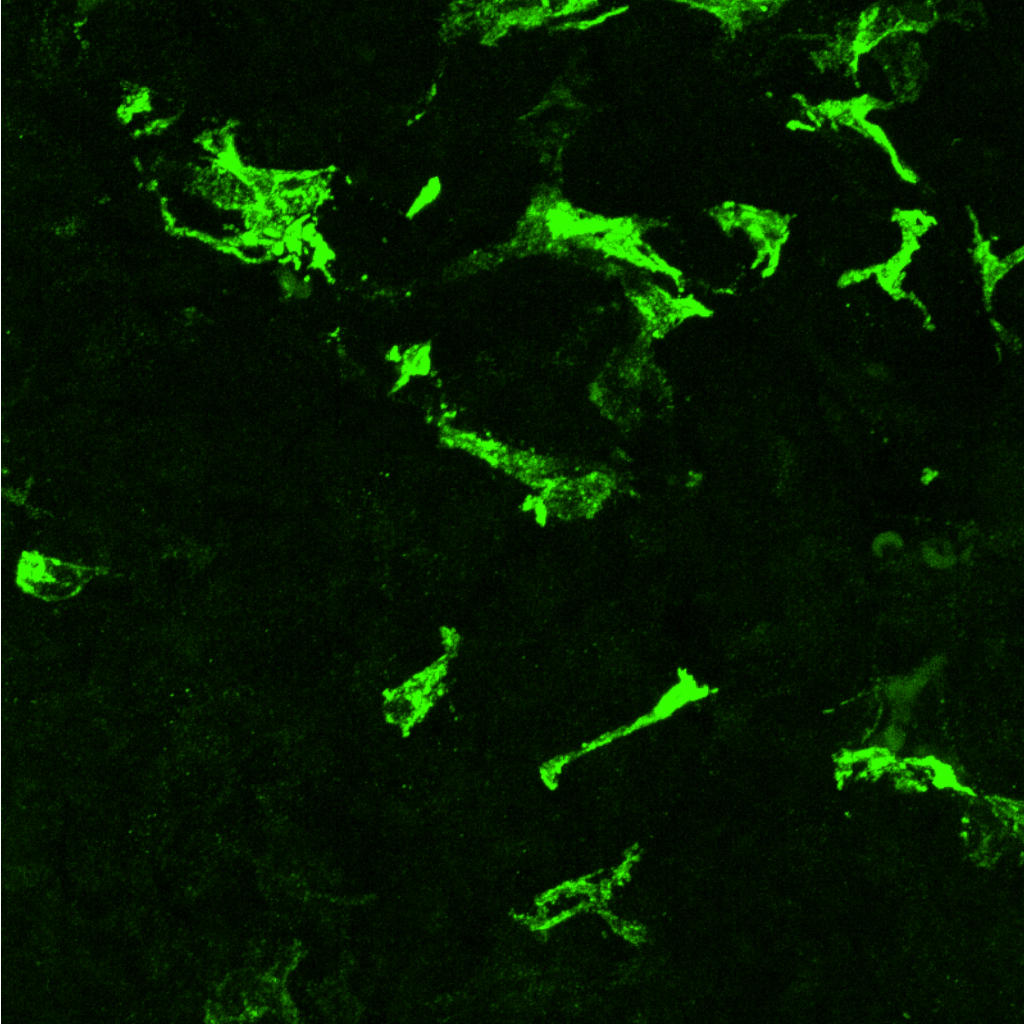

Supplement: Supplementary file 7 — EV Figure Source Data [file 44318_2024_78_MOESM7_ESM.zip › Expanded View/Expanded View 2/EV2A/sGC╬öpc-1.tif]

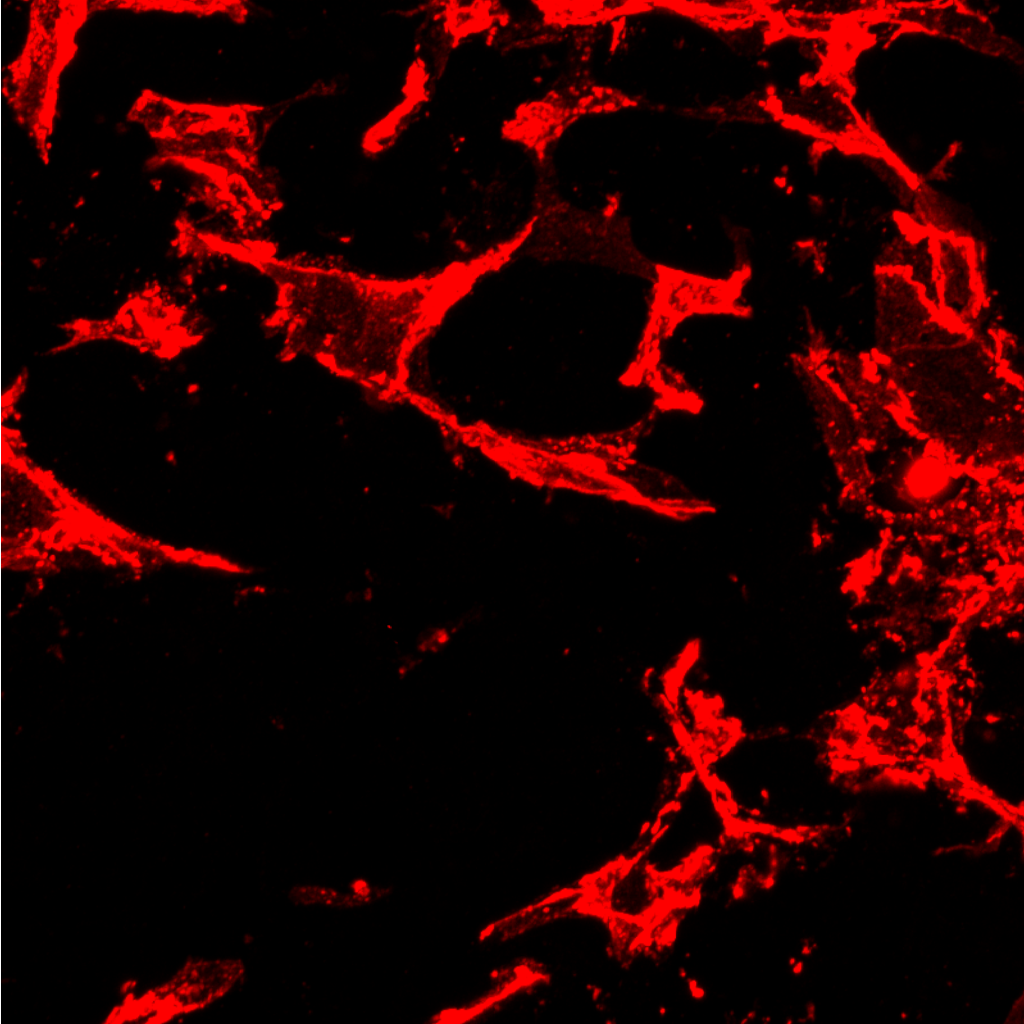

Supplement: Supplementary file 7 — EV Figure Source Data [file 44318_2024_78_MOESM7_ESM.zip › Expanded View/Expanded View 2/EV2A/sGC╬öpc-2.tif]

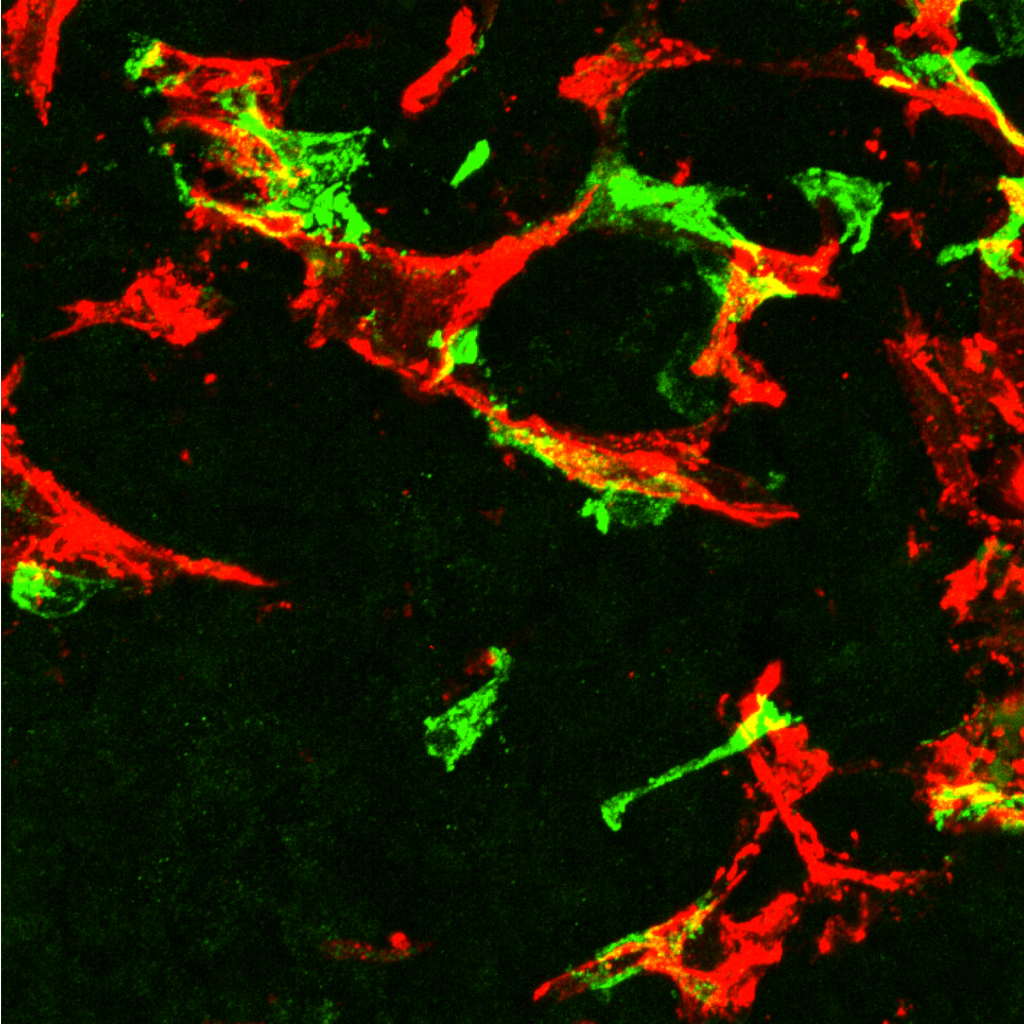

Supplement: Supplementary file 7 — EV Figure Source Data [file 44318_2024_78_MOESM7_ESM.zip › Expanded View/Expanded View 2/EV2A/sGC╬öpc-3.tif]

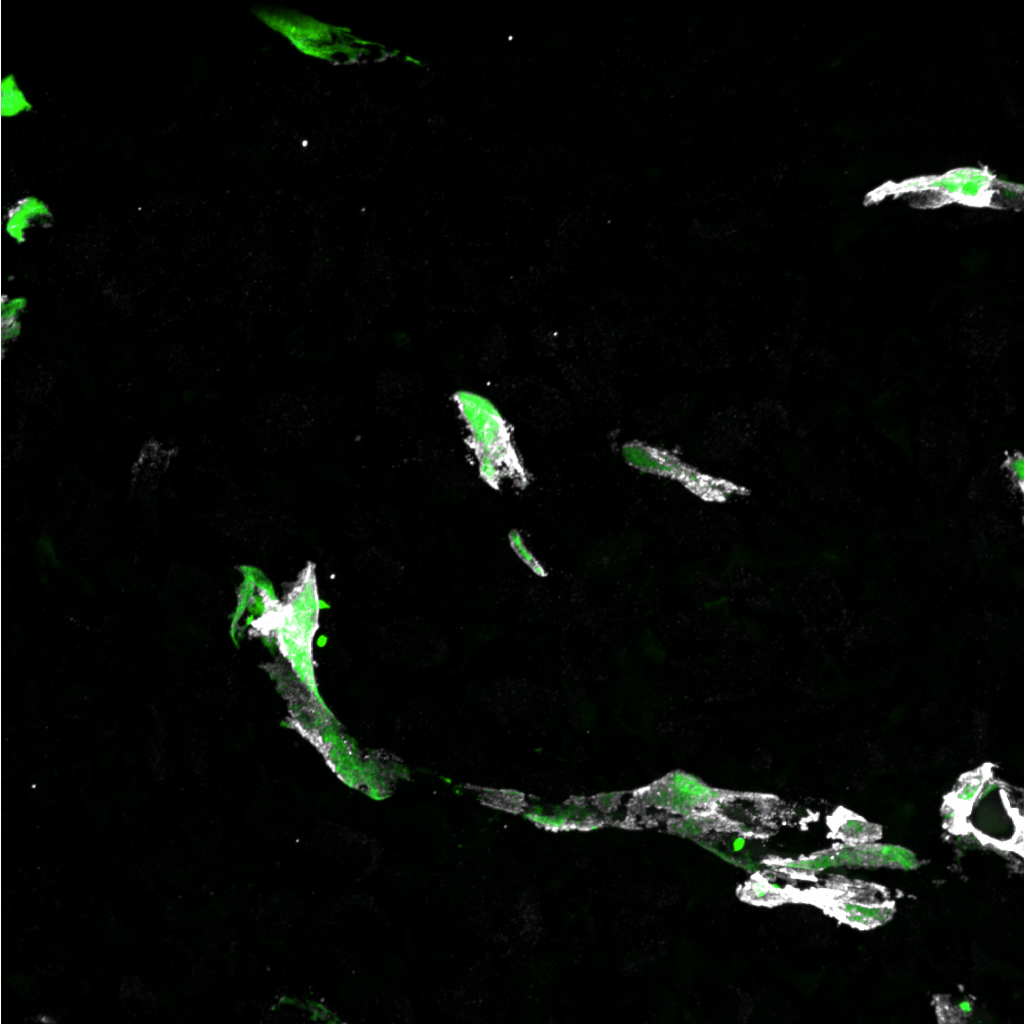

Supplement: Supplementary file 7 — EV Figure Source Data [file 44318_2024_78_MOESM7_ESM.zip › Expanded View/Expanded View 1/1B/NG2-6.tif]

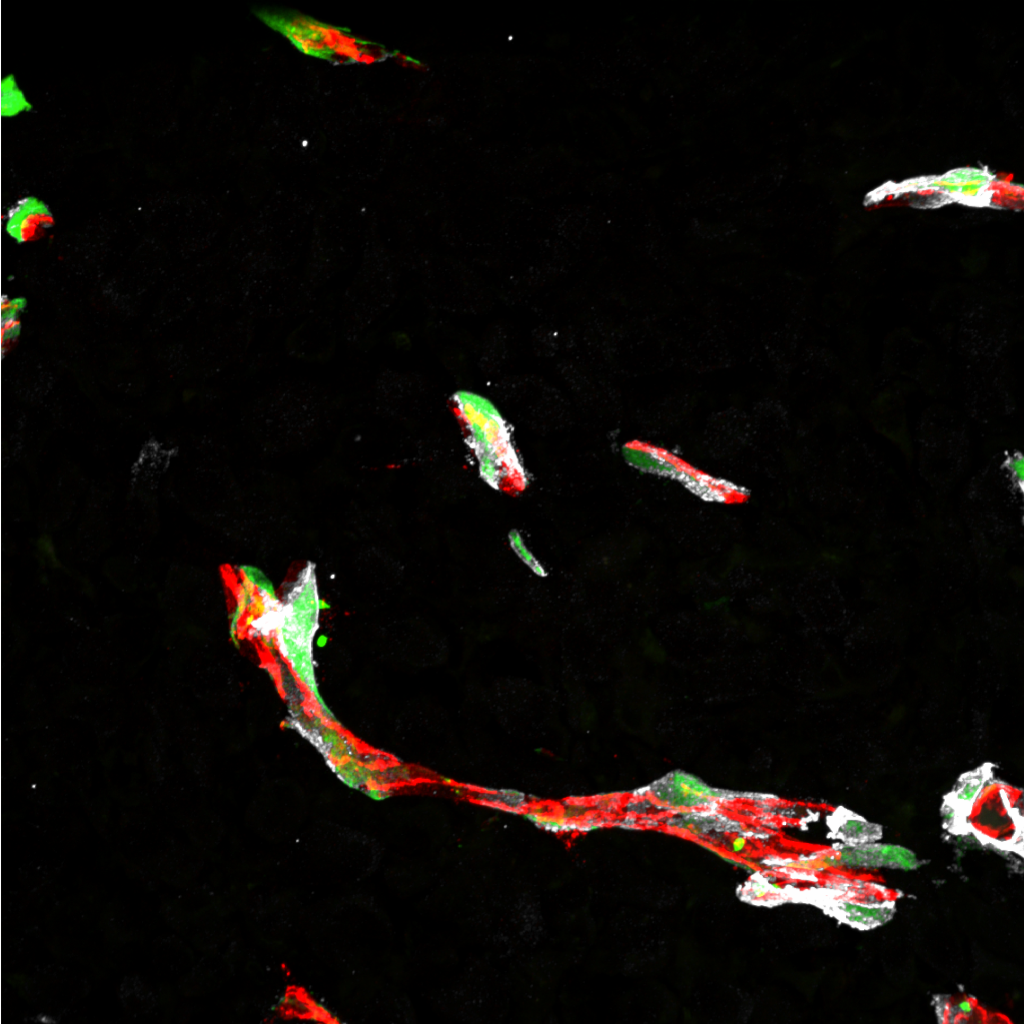

Supplement: Supplementary file 7 — EV Figure Source Data [file 44318_2024_78_MOESM7_ESM.zip › Expanded View/Expanded View 1/1B/NG2-7.tif]

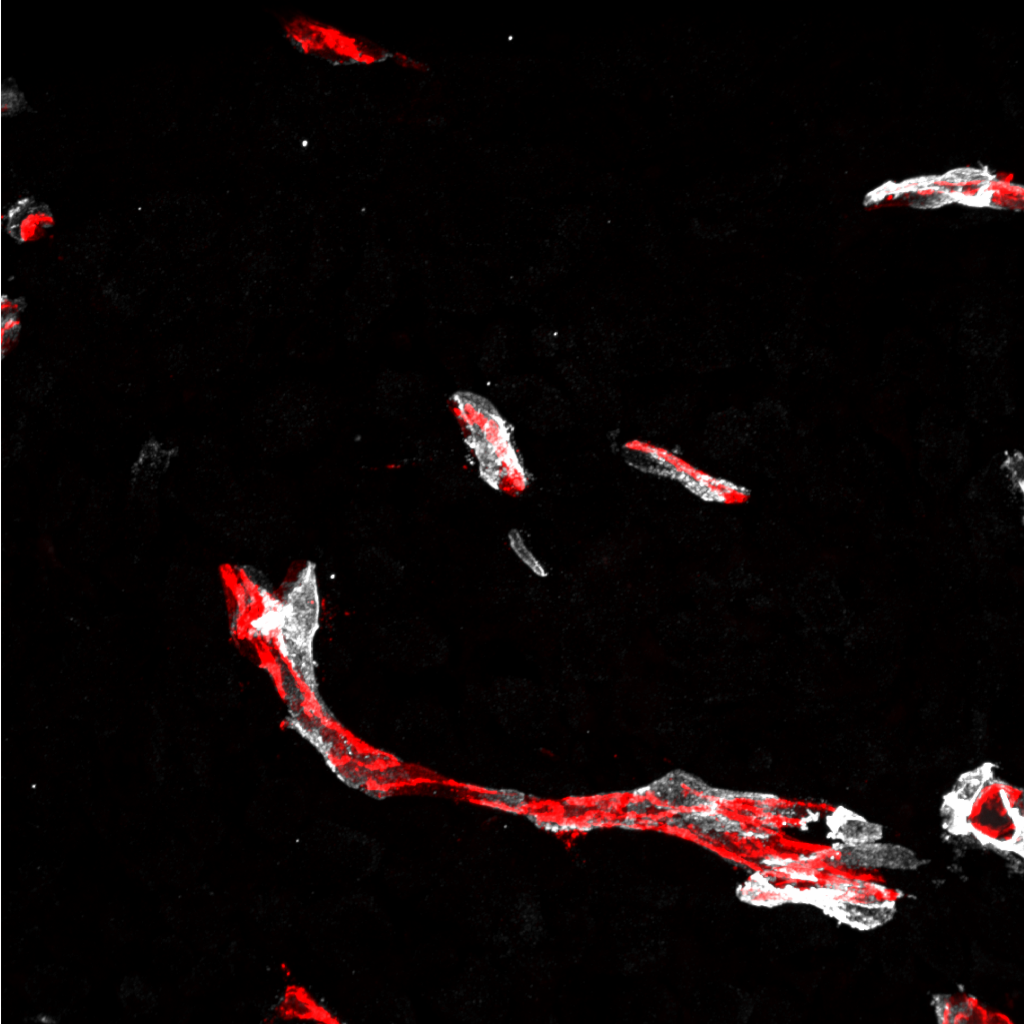

Supplement: Supplementary file 7 — EV Figure Source Data [file 44318_2024_78_MOESM7_ESM.zip › Expanded View/Expanded View 1/1B/NG2-5.tif]

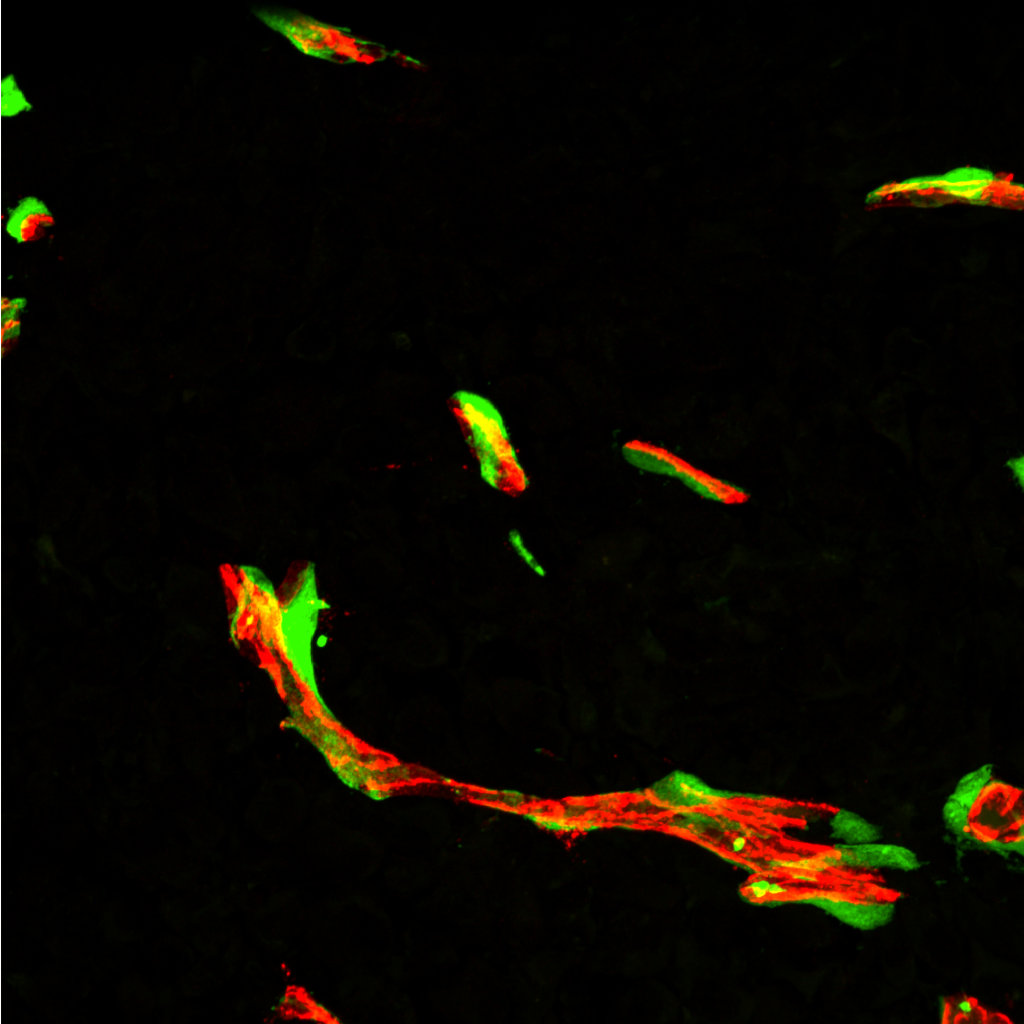

Supplement: Supplementary file 7 — EV Figure Source Data [file 44318_2024_78_MOESM7_ESM.zip › Expanded View/Expanded View 1/1B/NG2-4.tif]

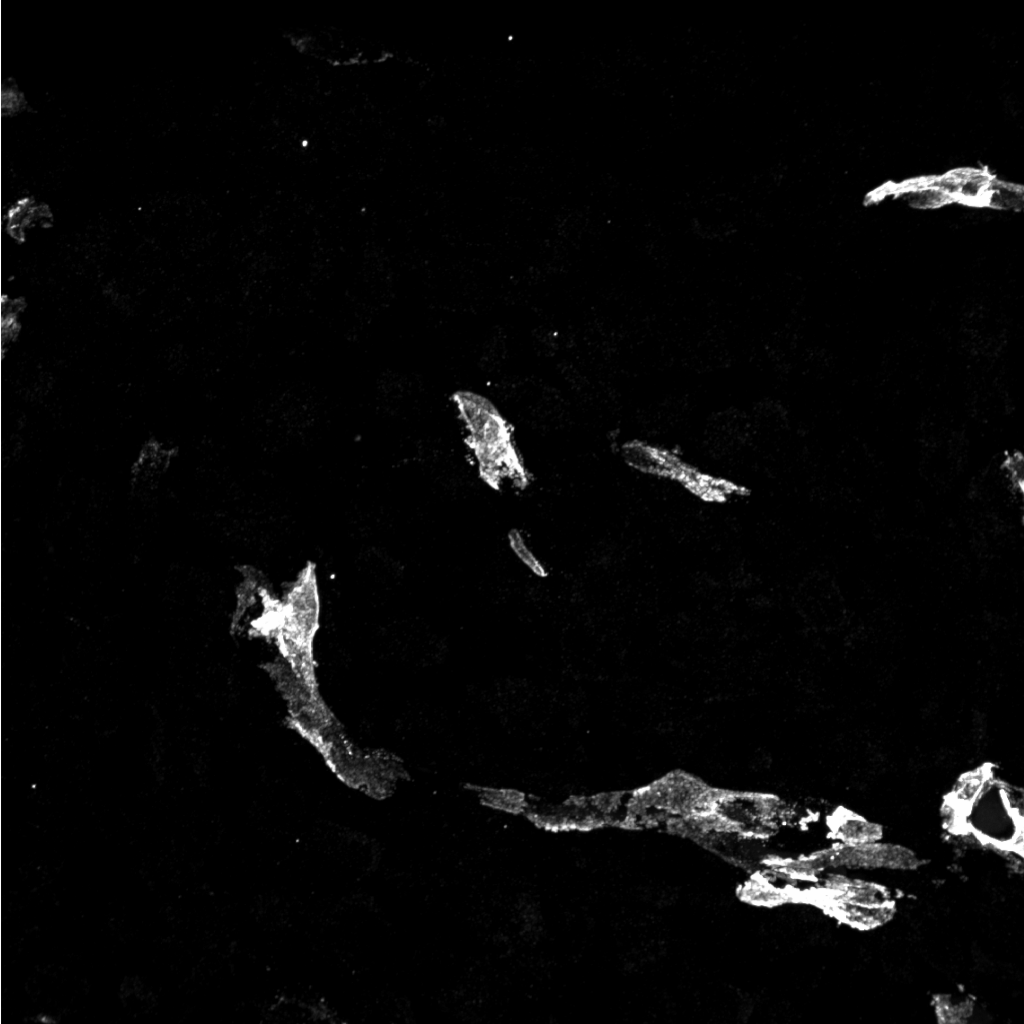

Supplement: Supplementary file 7 — EV Figure Source Data [file 44318_2024_78_MOESM7_ESM.zip › Expanded View/Expanded View 1/1B/NG2-1.tif]

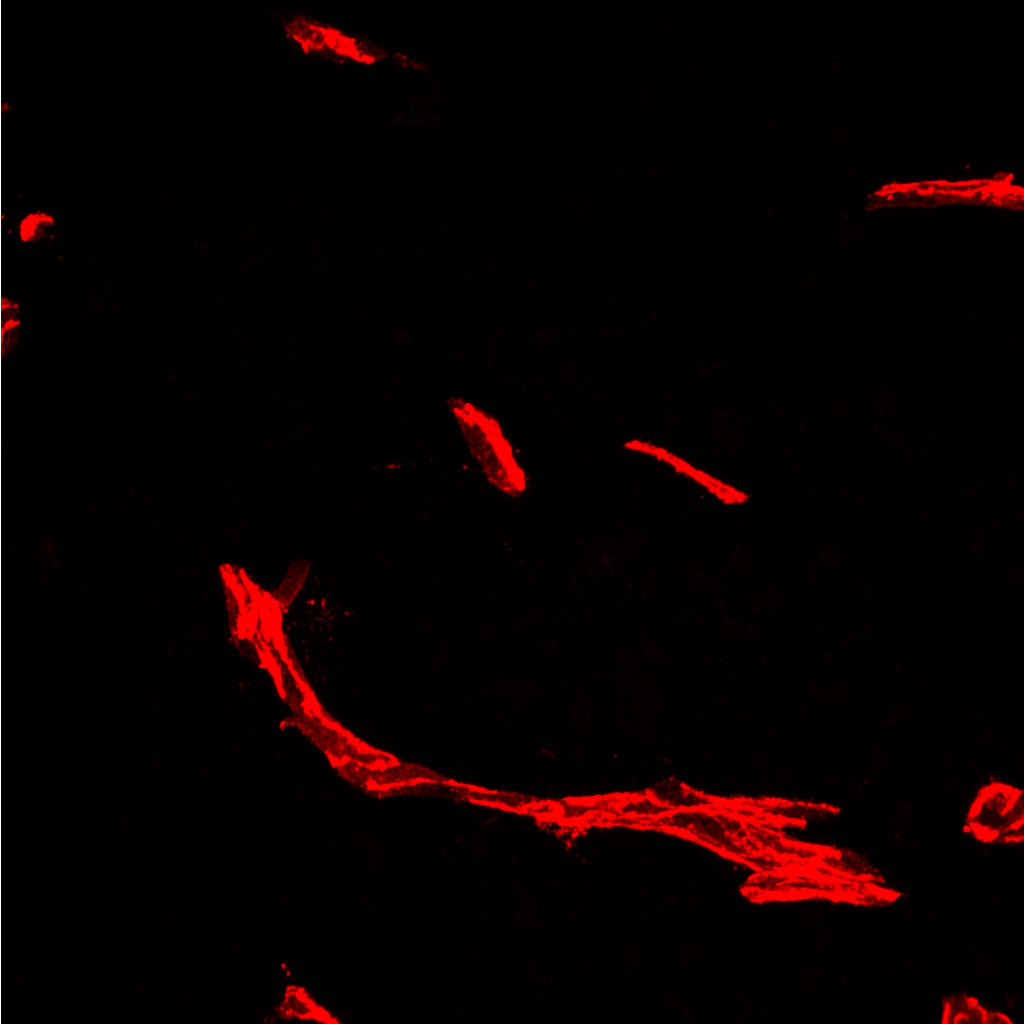

Supplement: Supplementary file 7 — EV Figure Source Data [file 44318_2024_78_MOESM7_ESM.zip › Expanded View/Expanded View 1/1B/NG2-3.tif]

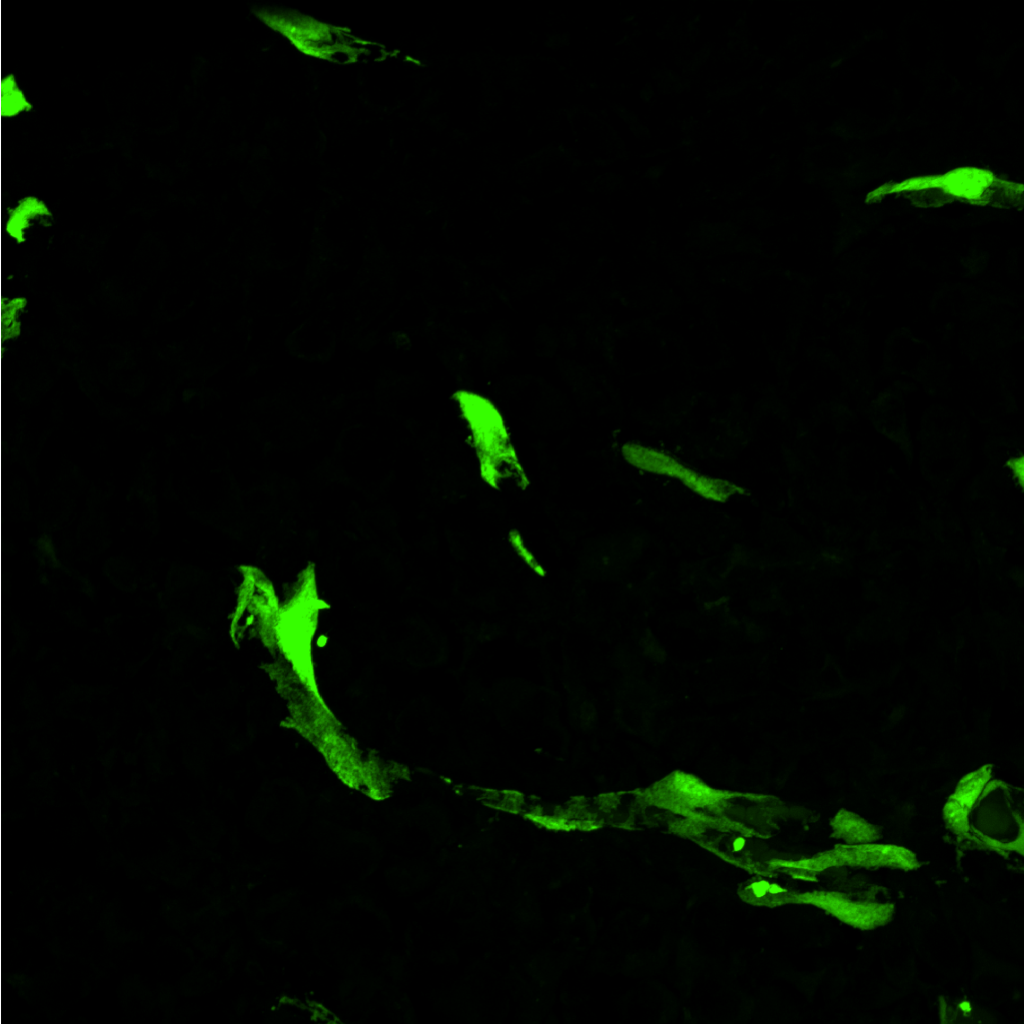

Supplement: Supplementary file 7 — EV Figure Source Data [file 44318_2024_78_MOESM7_ESM.zip › Expanded View/Expanded View 1/1B/NG2-2.tif]

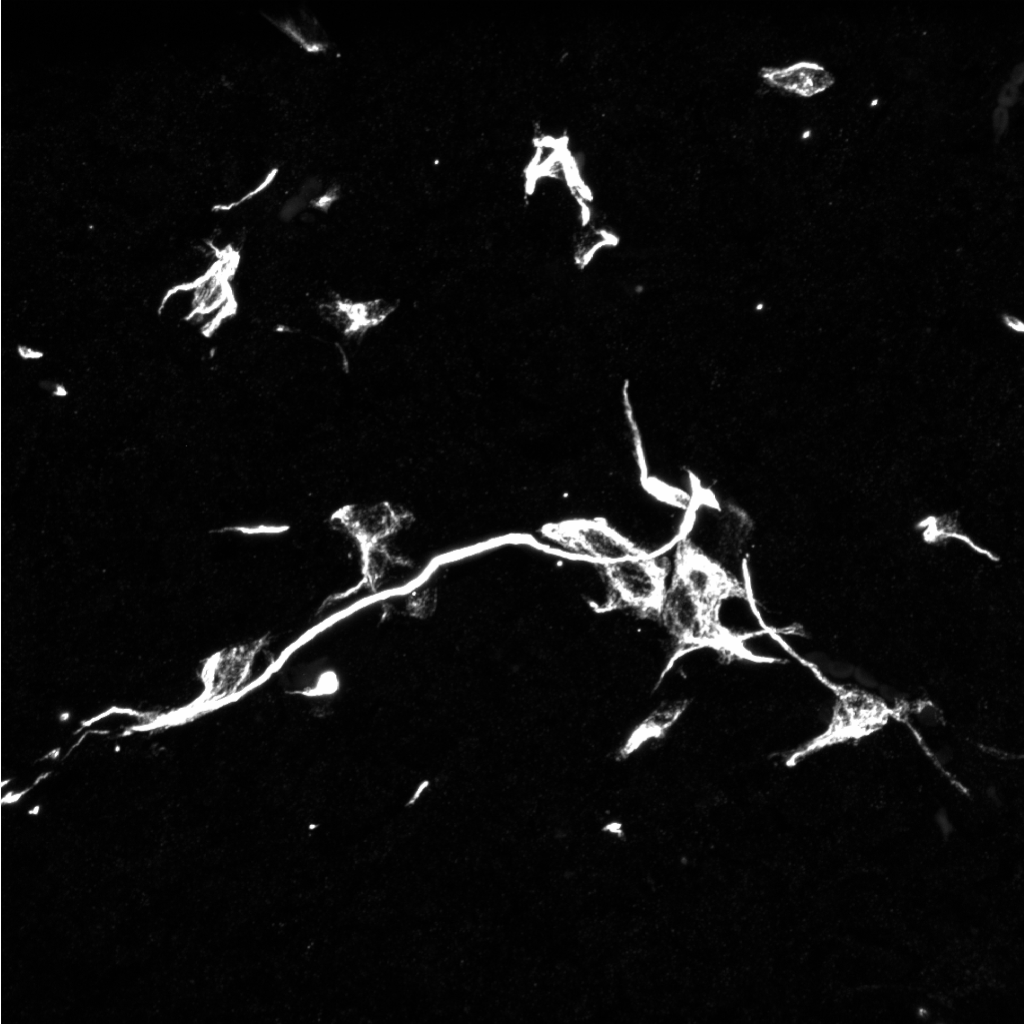

Supplement: Supplementary file 7 — EV Figure Source Data [file 44318_2024_78_MOESM7_ESM.zip › Expanded View/Expanded View 1/1B/Desmin-1.tif]

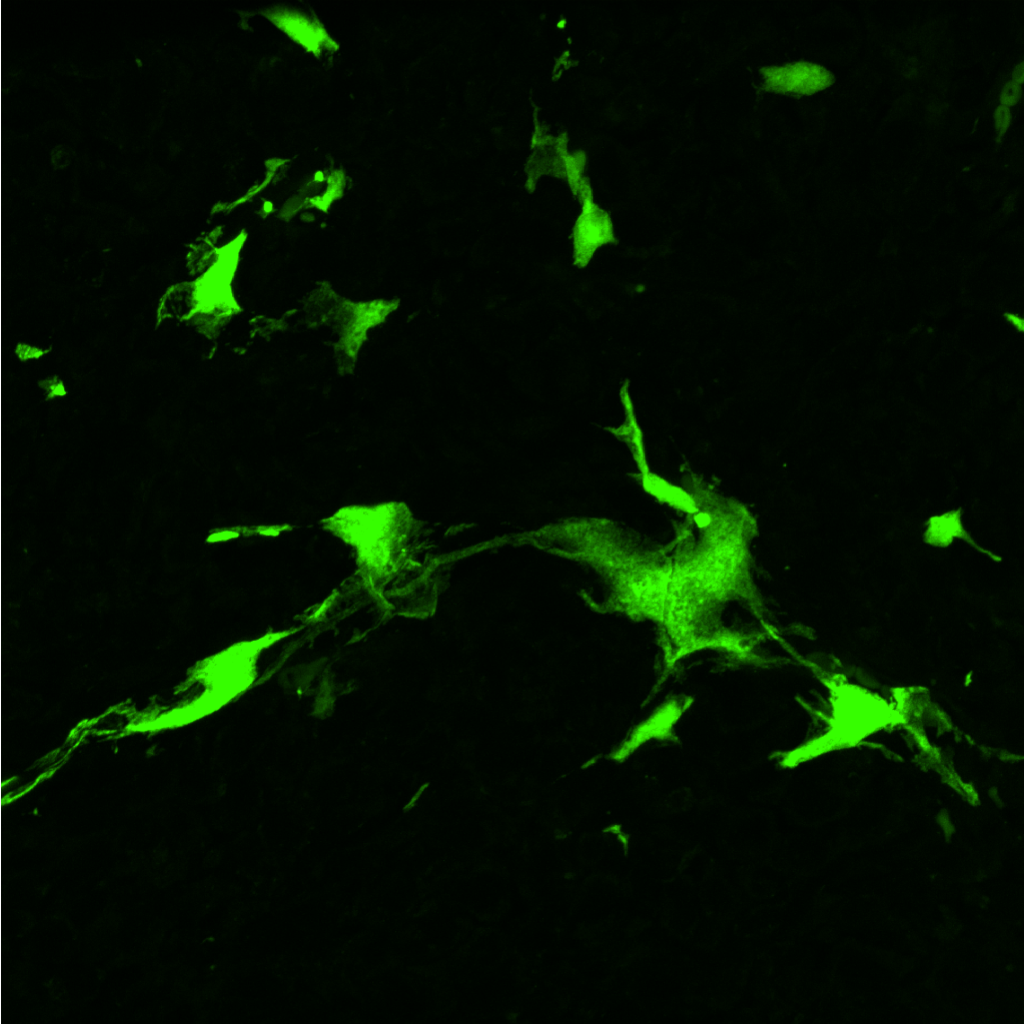

Supplement: Supplementary file 7 — EV Figure Source Data [file 44318_2024_78_MOESM7_ESM.zip › Expanded View/Expanded View 1/1B/Desmin-2.tif]
