# Supplementary figures and images for: Pericyte signaling via soluble guanylate cyclase shapes the vascular niche and microenvironment of tumors (part 3 of 4)
Source: EMBO J. 2024 Mar 25;43(8):7. doi: 10.1038/s44318-024-00078-5 (PMC11021551; doi:10.1038/s44318-024-00078-5)

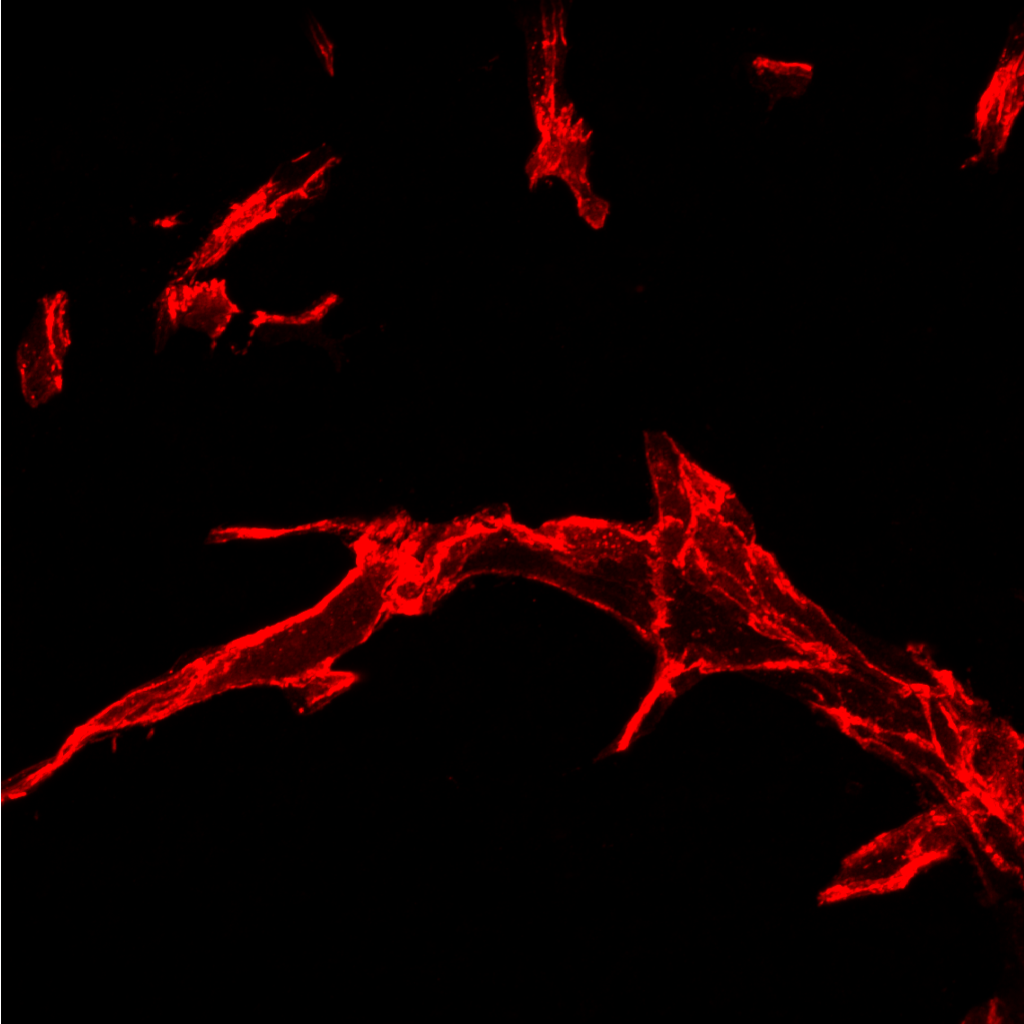

Supplement: Supplementary file 7 — EV Figure Source Data [file 44318_2024_78_MOESM7_ESM.zip › Expanded View/Expanded View 1/1B/Desmin-3.tif]

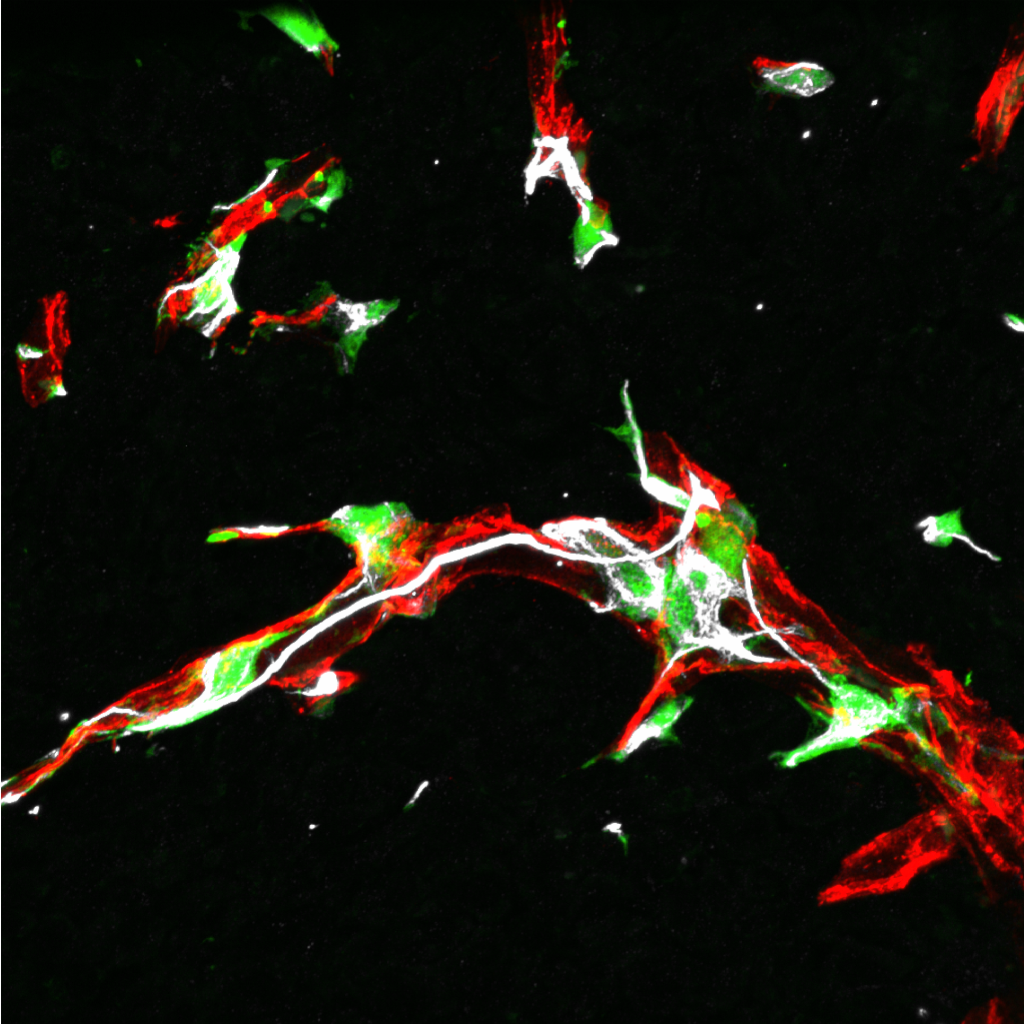

Supplement: Supplementary file 7 — EV Figure Source Data [file 44318_2024_78_MOESM7_ESM.zip › Expanded View/Expanded View 1/1B/Desmin-7.tif]

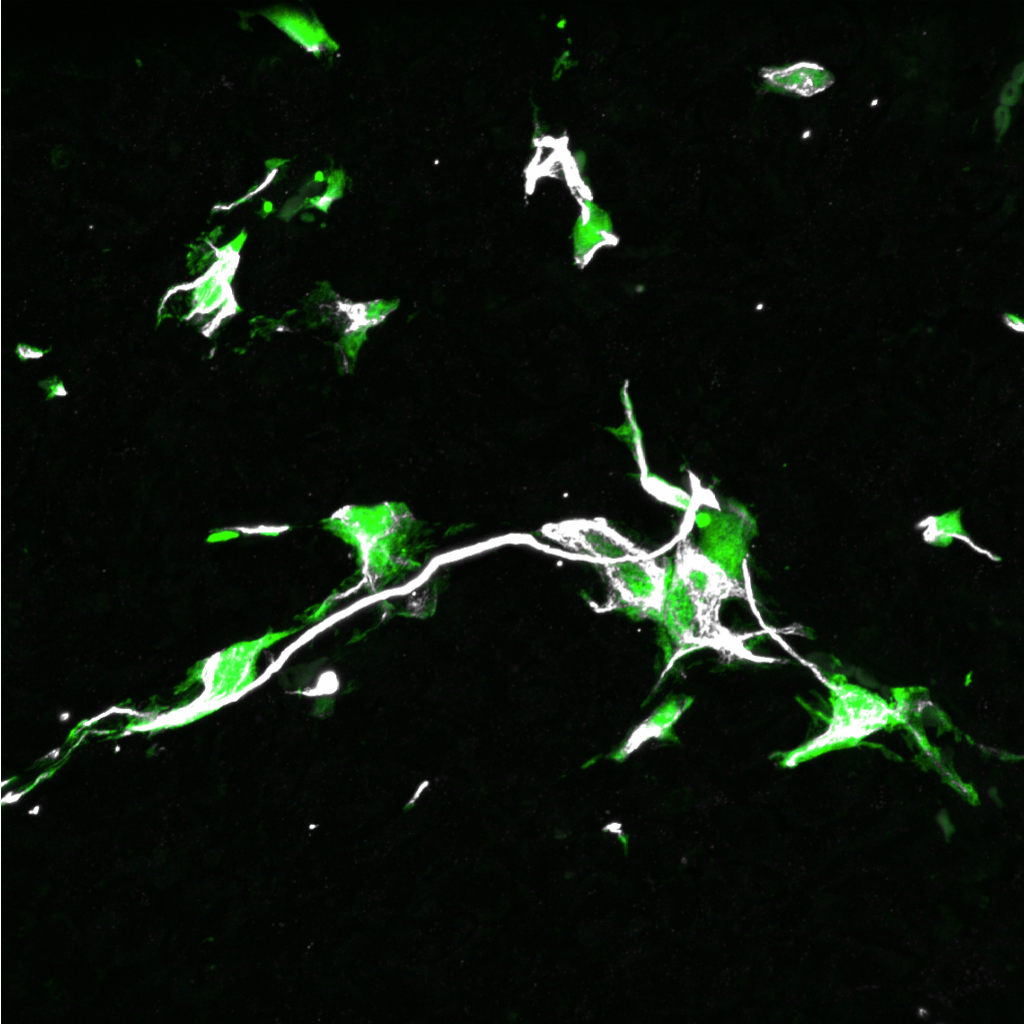

Supplement: Supplementary file 7 — EV Figure Source Data [file 44318_2024_78_MOESM7_ESM.zip › Expanded View/Expanded View 1/1B/Desmin-6.tif]

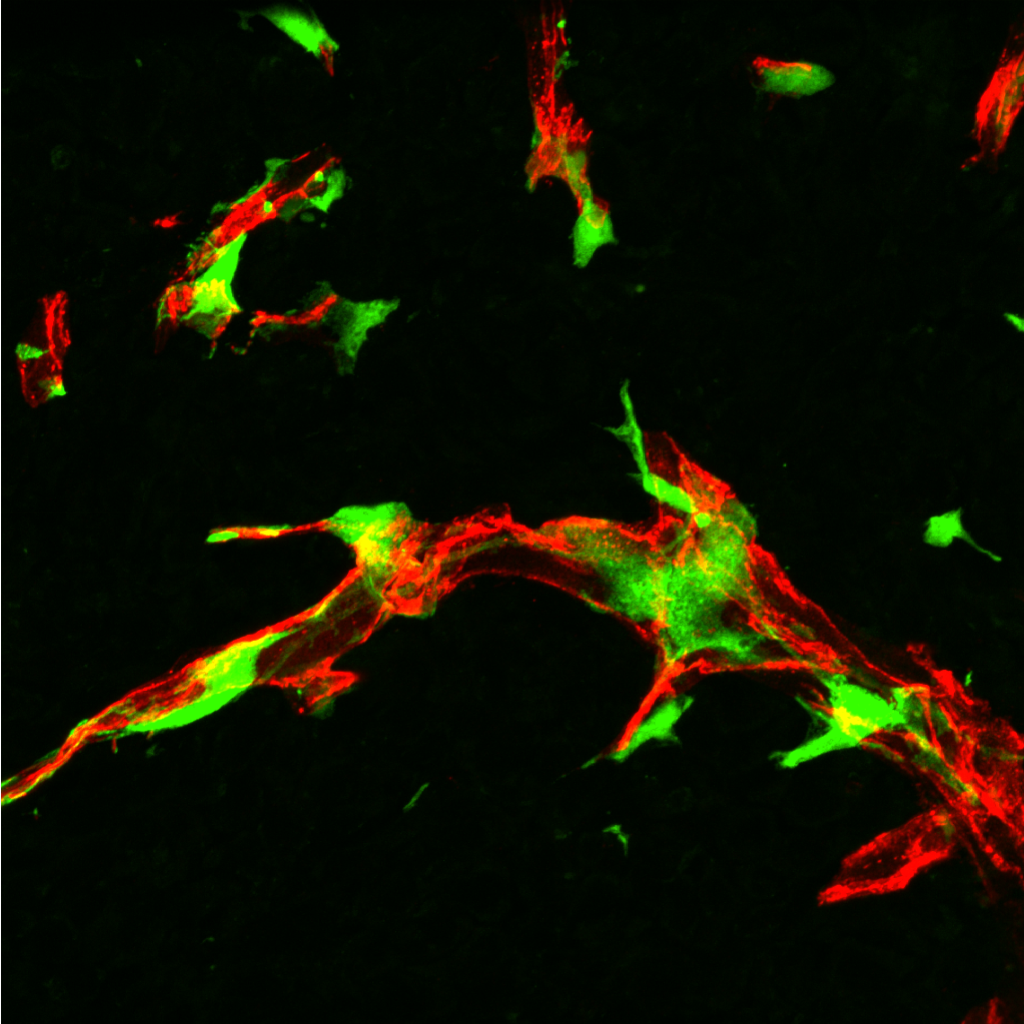

Supplement: Supplementary file 7 — EV Figure Source Data [file 44318_2024_78_MOESM7_ESM.zip › Expanded View/Expanded View 1/1B/Desmin-4.tif]

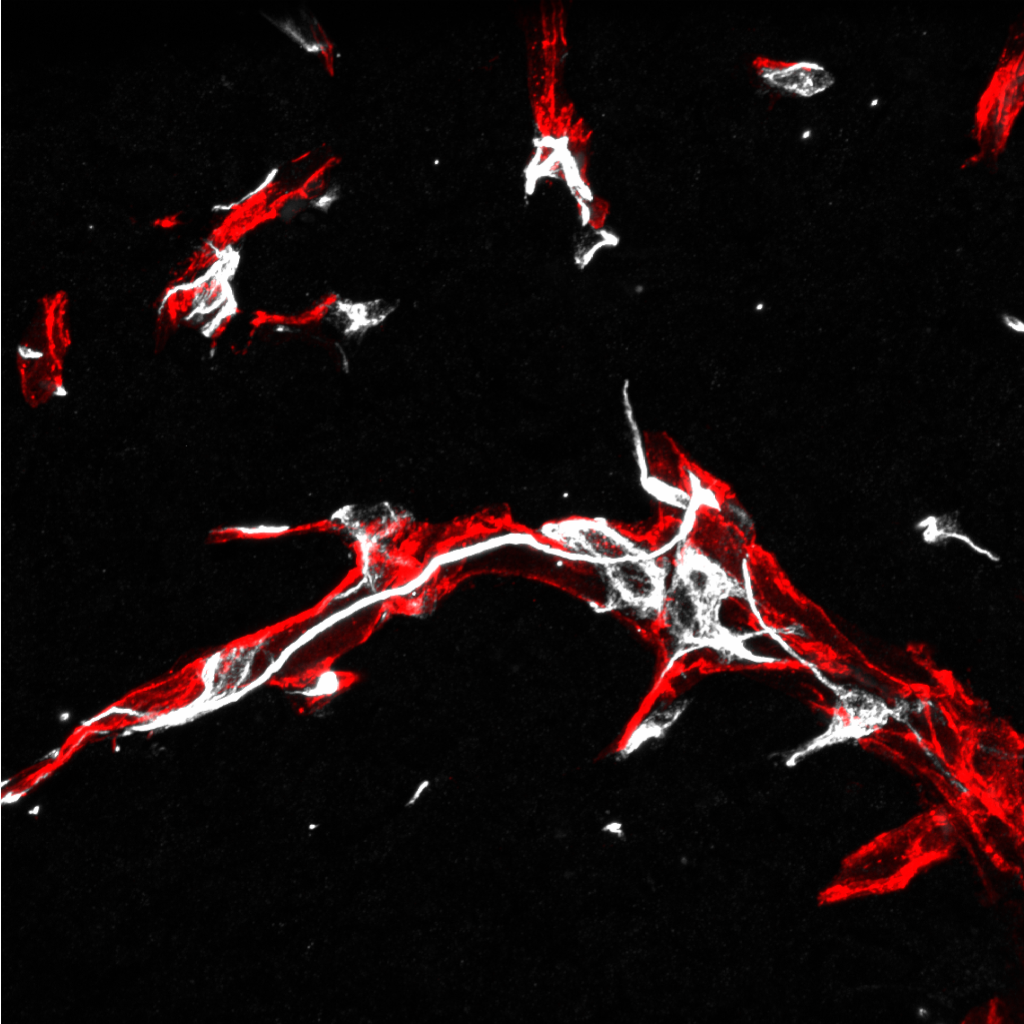

Supplement: Supplementary file 7 — EV Figure Source Data [file 44318_2024_78_MOESM7_ESM.zip › Expanded View/Expanded View 1/1B/Desmin-5.tif]

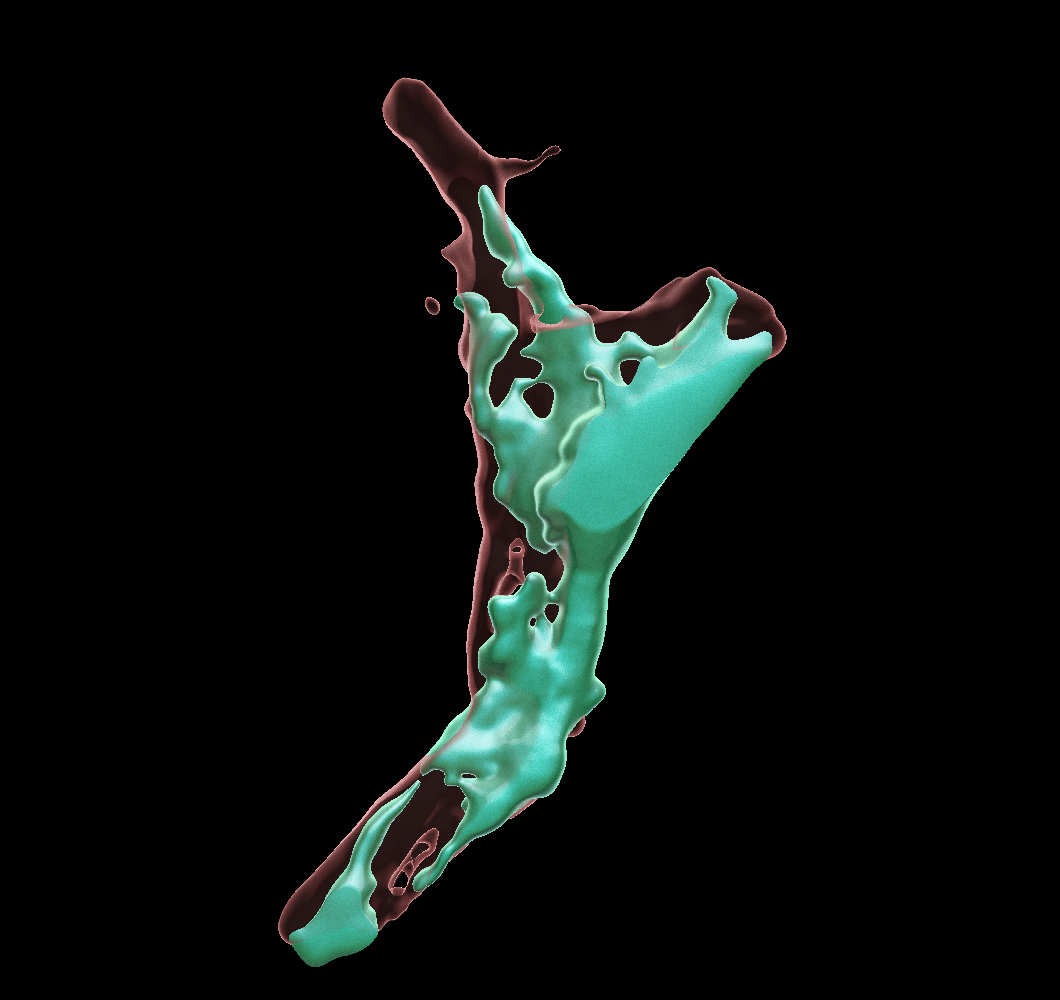

Supplement: Supplementary file 7 — EV Figure Source Data [file 44318_2024_78_MOESM7_ESM.zip › Expanded View/Expanded View 1/1D/3.tif]

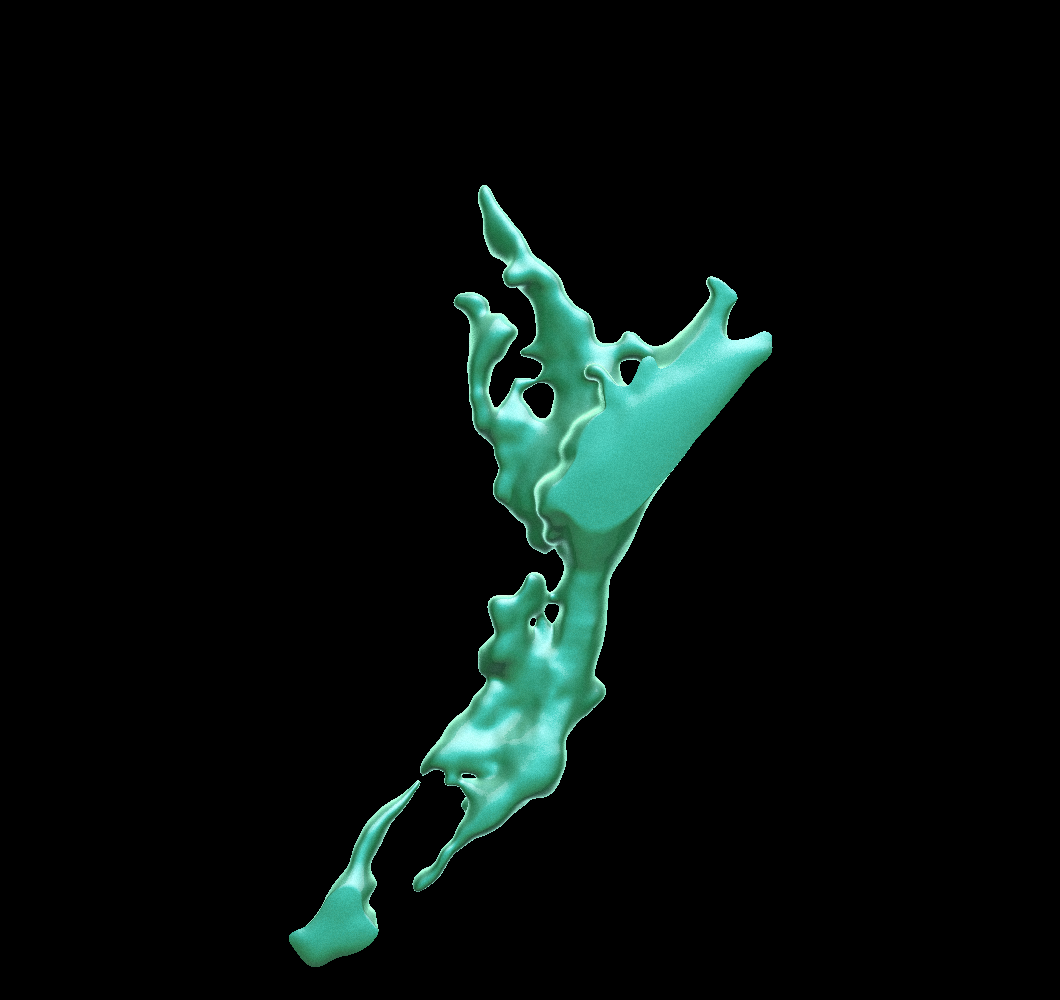

Supplement: Supplementary file 7 — EV Figure Source Data [file 44318_2024_78_MOESM7_ESM.zip › Expanded View/Expanded View 1/1D/2.tif]

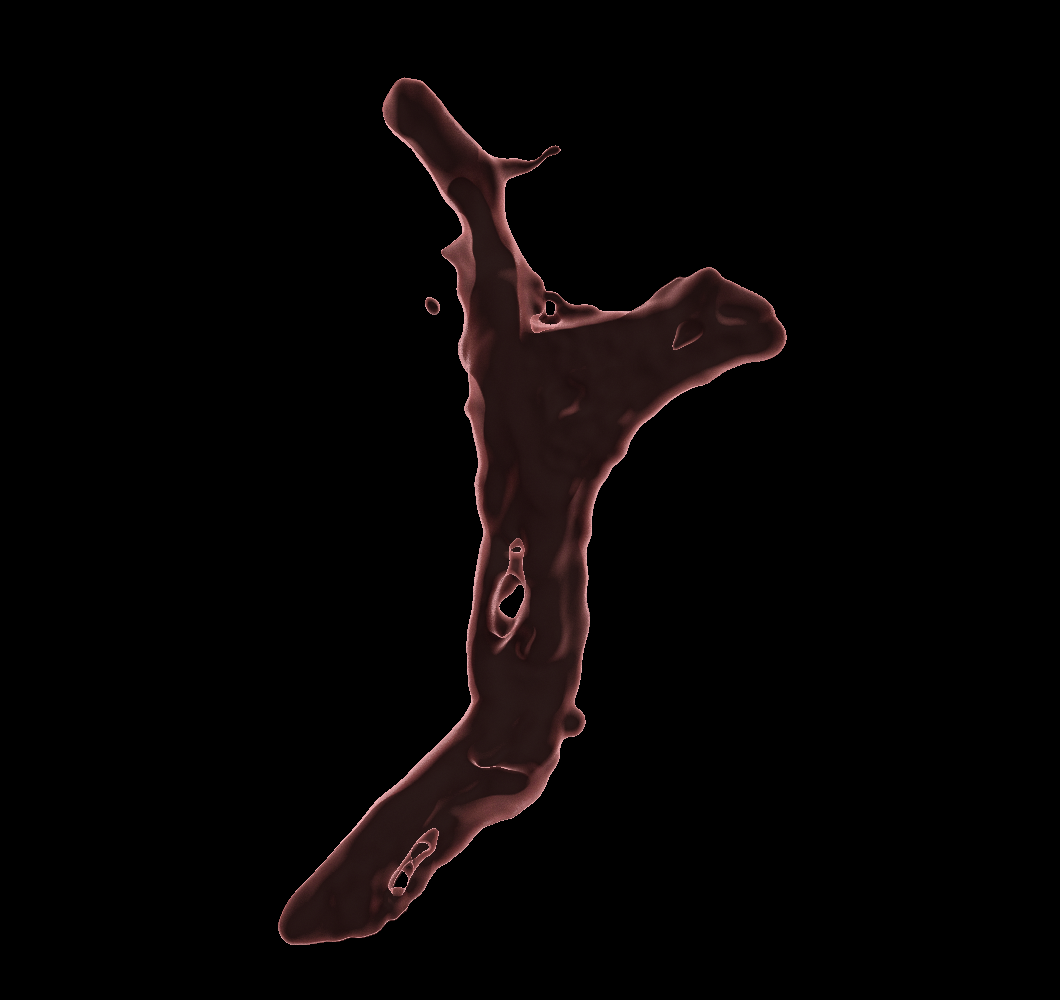

Supplement: Supplementary file 7 — EV Figure Source Data [file 44318_2024_78_MOESM7_ESM.zip › Expanded View/Expanded View 1/1D/1.tif]

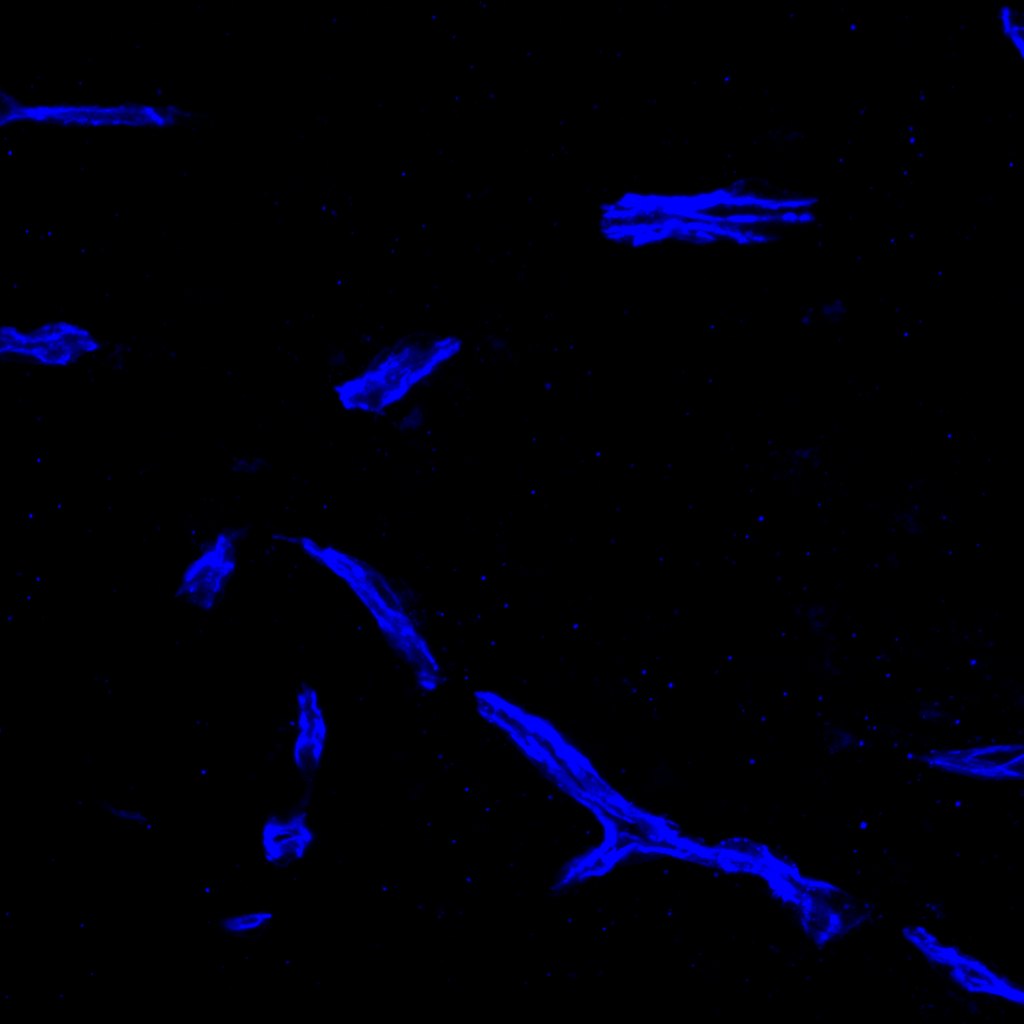

Supplement: Supplementary file 7 — EV Figure Source Data [file 44318_2024_78_MOESM7_ESM.zip › Expanded View/Expanded View 1/1C/3.tif]

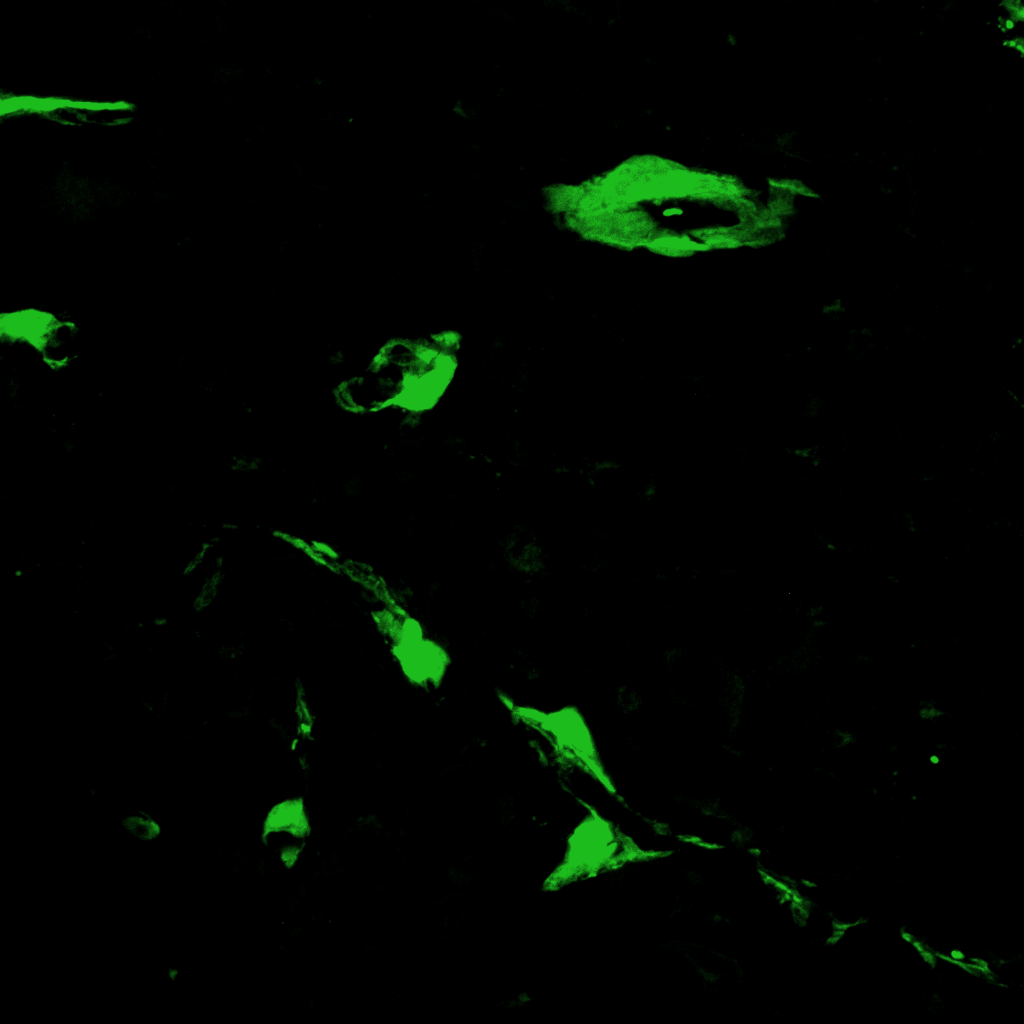

Supplement: Supplementary file 7 — EV Figure Source Data [file 44318_2024_78_MOESM7_ESM.zip › Expanded View/Expanded View 1/1C/2.tif]

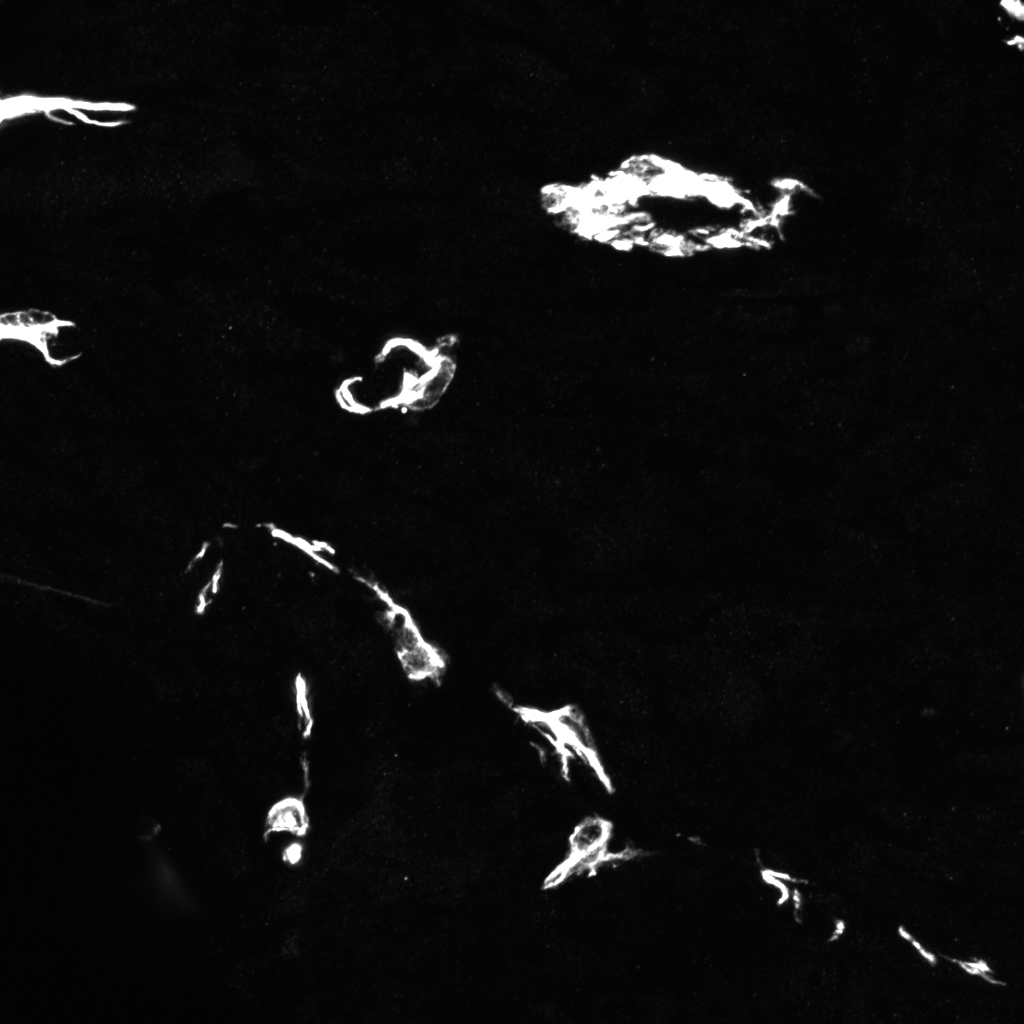

Supplement: Supplementary file 7 — EV Figure Source Data [file 44318_2024_78_MOESM7_ESM.zip › Expanded View/Expanded View 1/1C/1.tif]

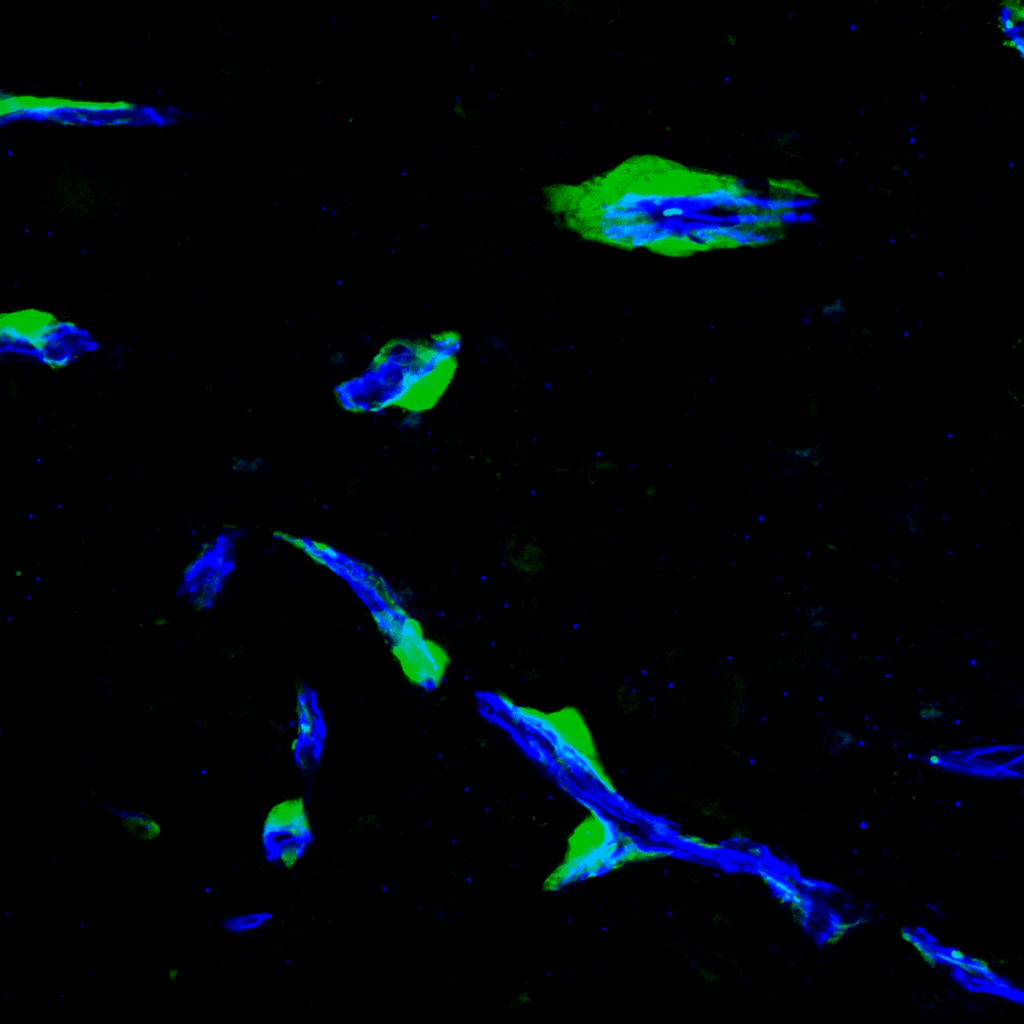

Supplement: Supplementary file 7 — EV Figure Source Data [file 44318_2024_78_MOESM7_ESM.zip › Expanded View/Expanded View 1/1C/5.tif]

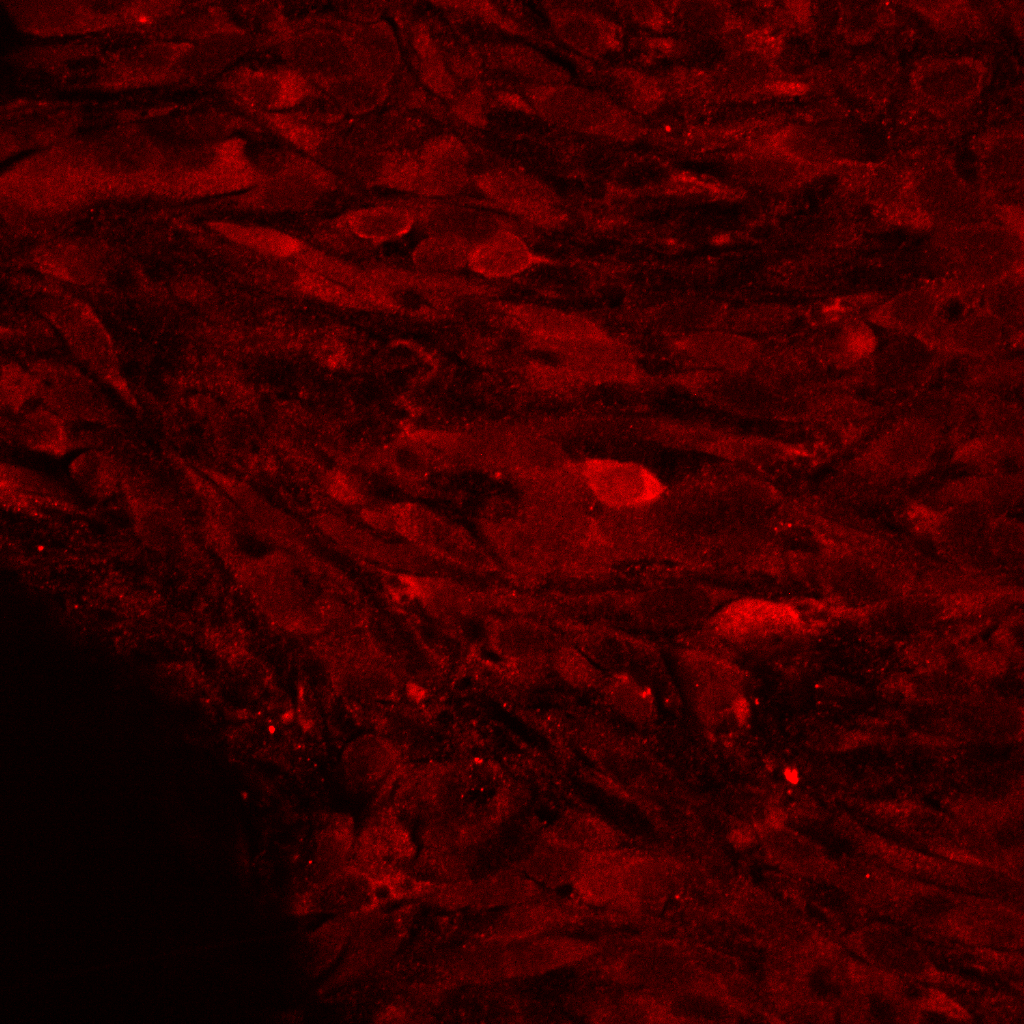

Supplement: Supplementary file 7 — EV Figure Source Data [file 44318_2024_78_MOESM7_ESM.zip › Expanded View/Expanded View 1/1C/4.tif]

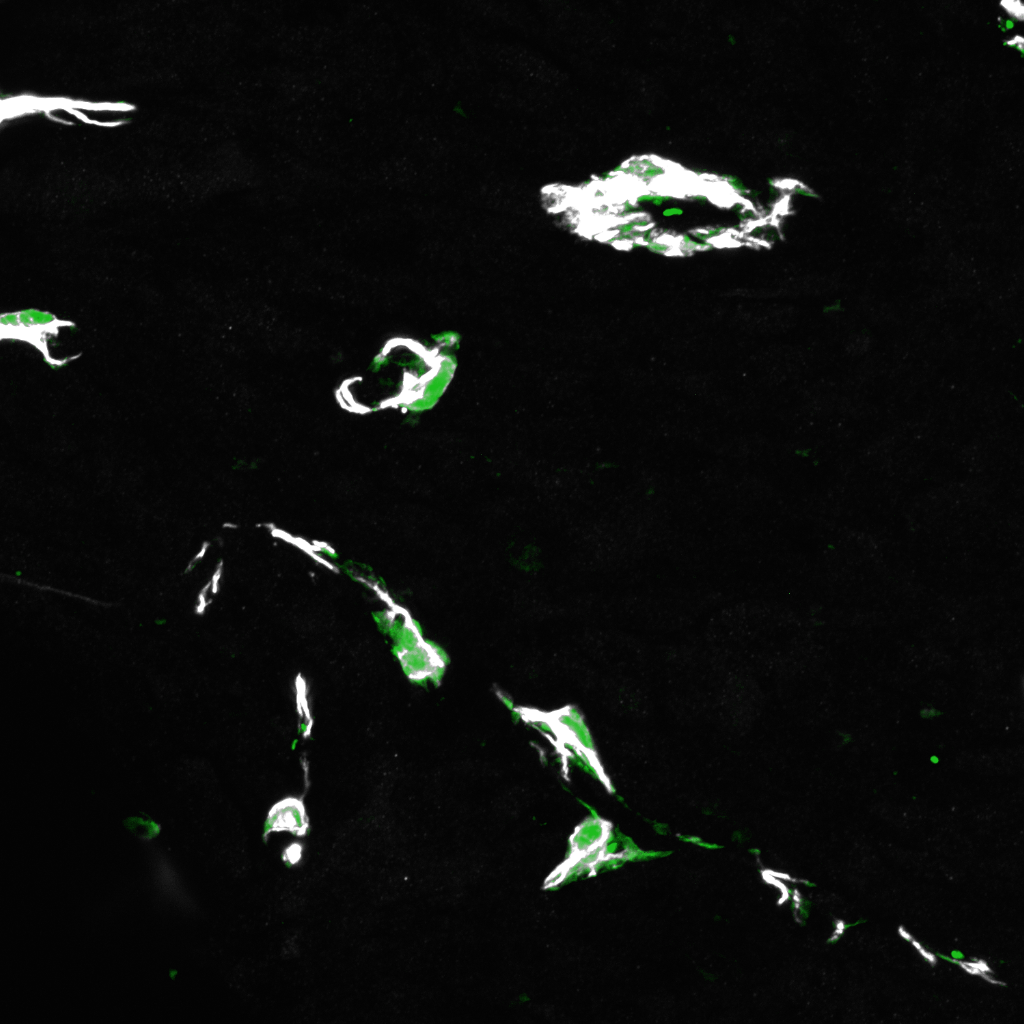

Supplement: Supplementary file 7 — EV Figure Source Data [file 44318_2024_78_MOESM7_ESM.zip › Expanded View/Expanded View 1/1C/6.tif]

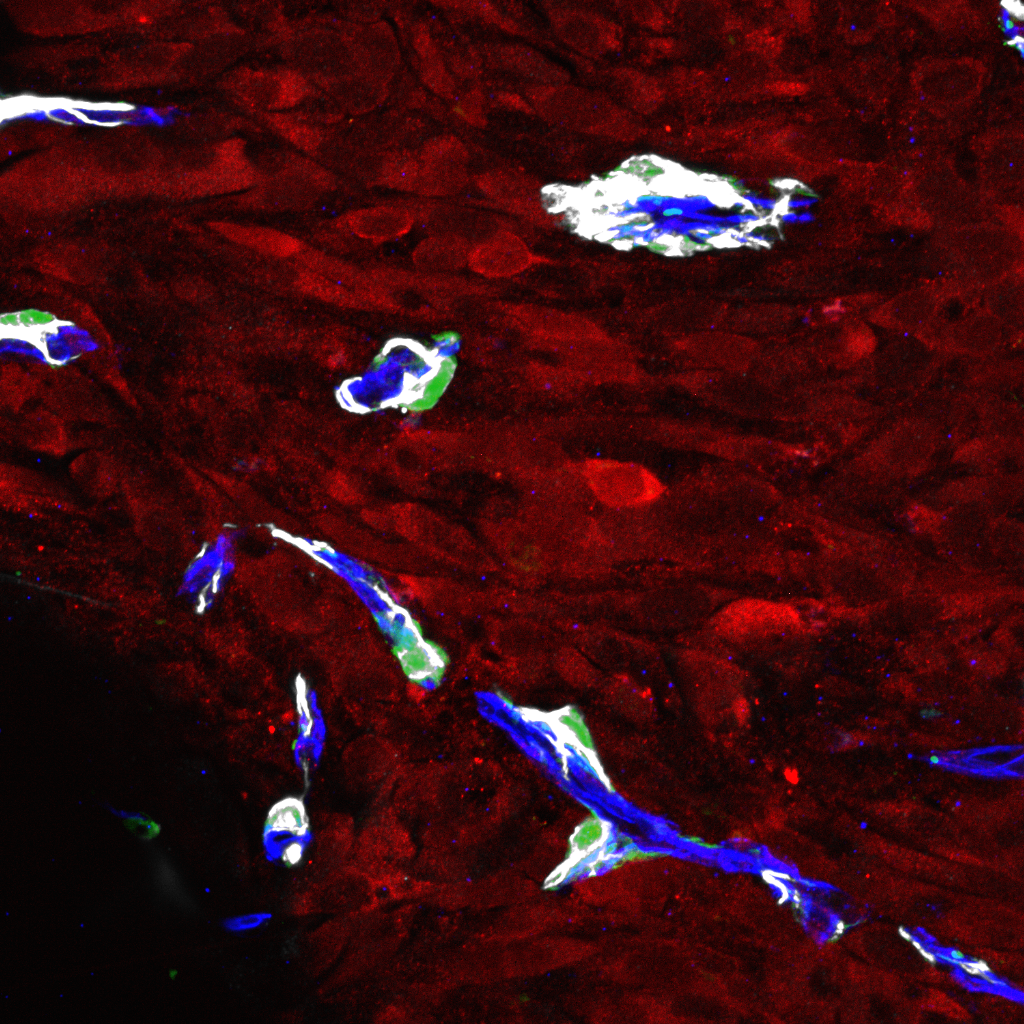

Supplement: Supplementary file 7 — EV Figure Source Data [file 44318_2024_78_MOESM7_ESM.zip › Expanded View/Expanded View 1/1C/7.tif]

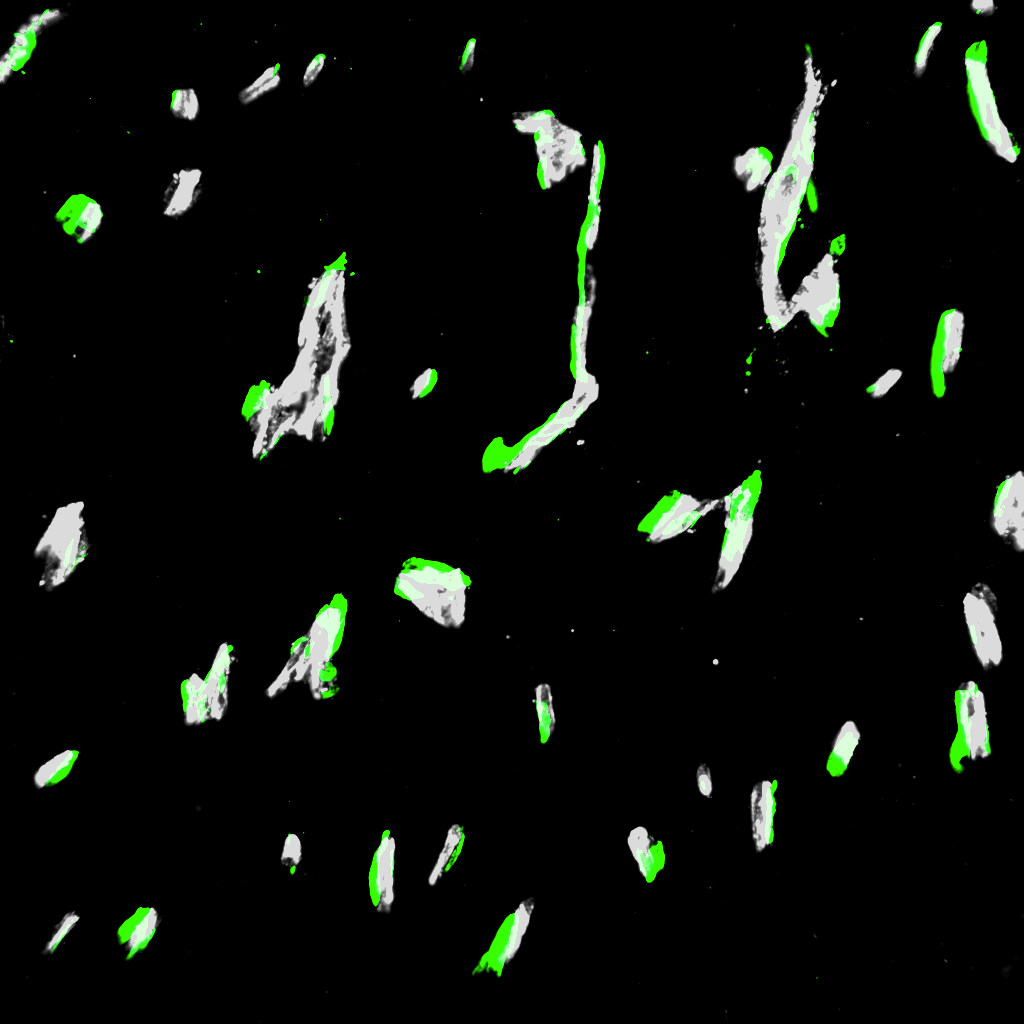

Supplement: Supplementary file 8 — Appendix Figure Source Data [file 44318_2024_78_MOESM8_ESM.zip › Appendix Figure/Appendix Figure S7/S7B/sGCCtr tdTomato-5.tif]

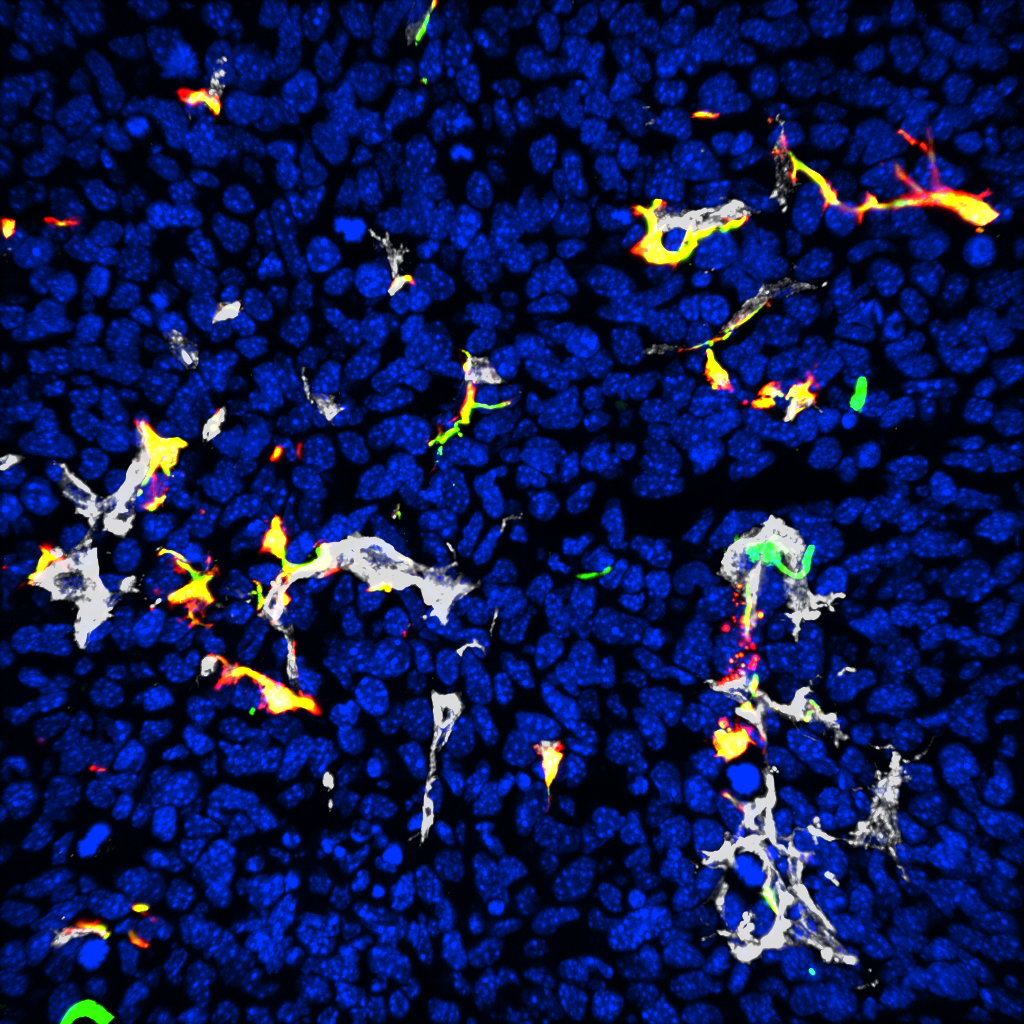

Supplement: Supplementary file 8 — Appendix Figure Source Data [file 44318_2024_78_MOESM8_ESM.zip › Appendix Figure/Appendix Figure S7/S7B/sGC╬öpc tdTomato-4.tif]

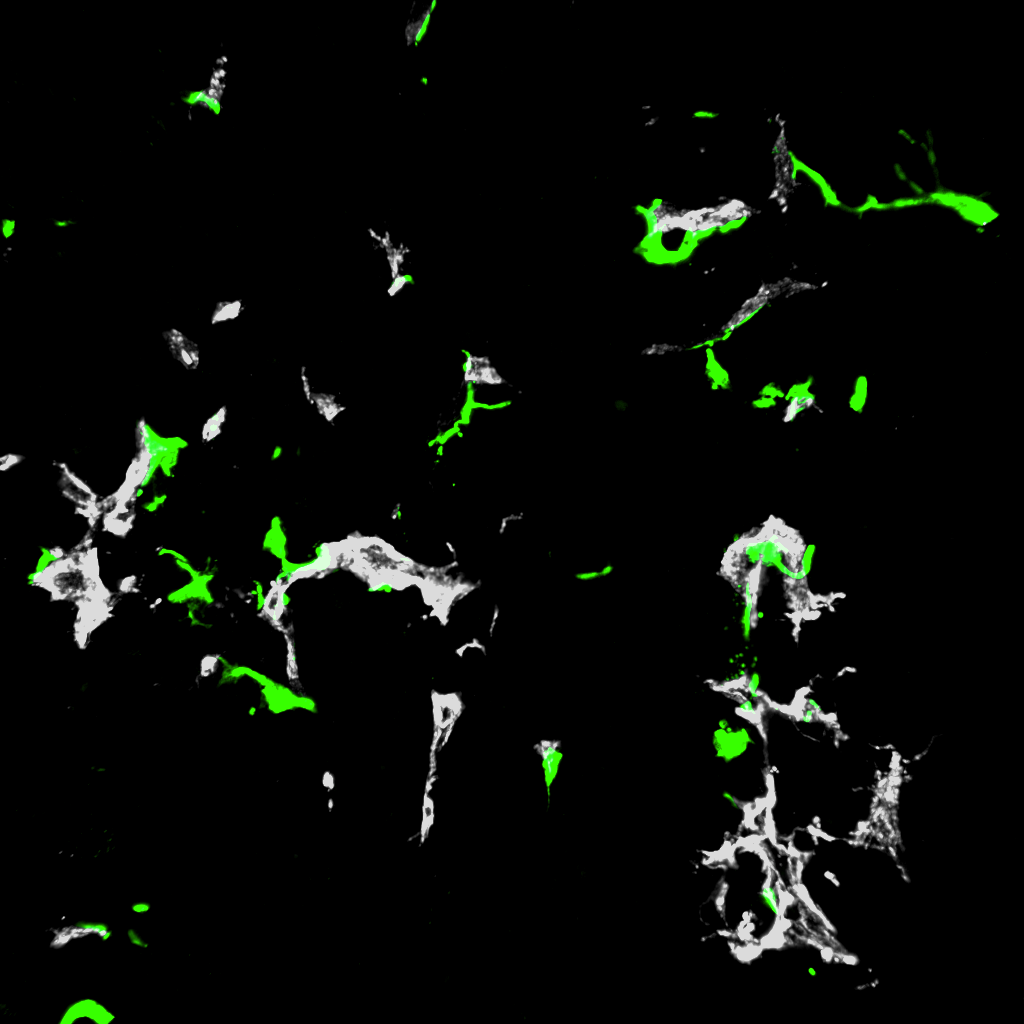

Supplement: Supplementary file 8 — Appendix Figure Source Data [file 44318_2024_78_MOESM8_ESM.zip › Appendix Figure/Appendix Figure S7/S7B/sGC╬öpc tdTomato-5.tif]

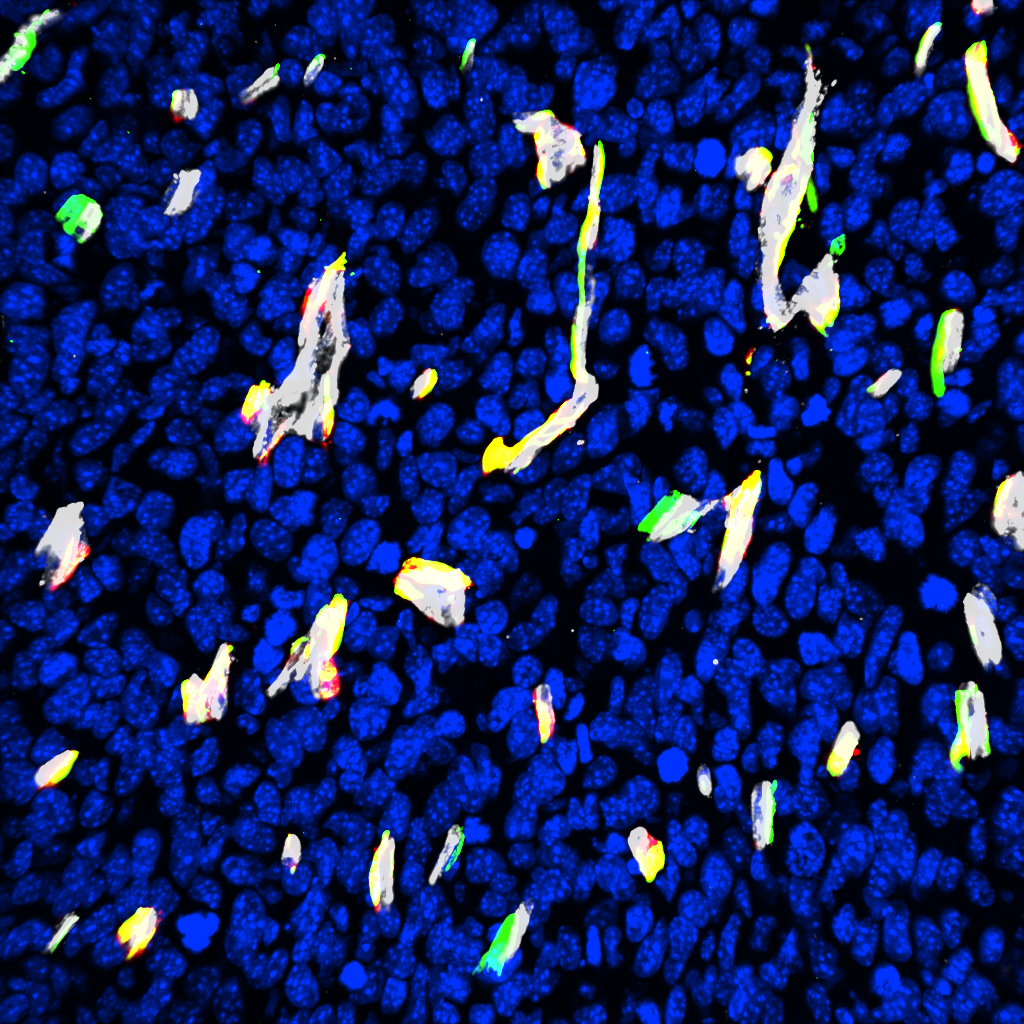

Supplement: Supplementary file 8 — Appendix Figure Source Data [file 44318_2024_78_MOESM8_ESM.zip › Appendix Figure/Appendix Figure S7/S7B/sGCCtr tdTomato-4.tif]

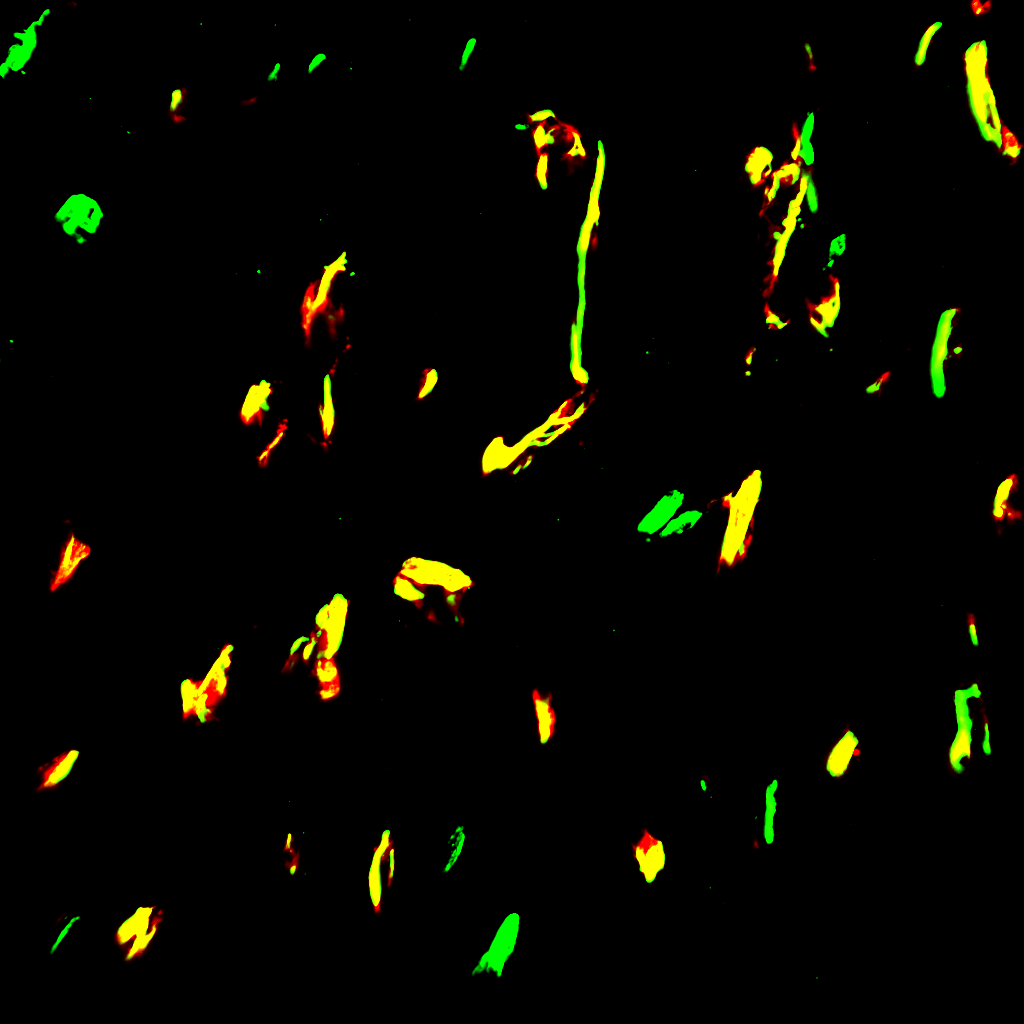

Supplement: Supplementary file 8 — Appendix Figure Source Data [file 44318_2024_78_MOESM8_ESM.zip › Appendix Figure/Appendix Figure S7/S7B/sGCCtr tdTomato-6.tif]

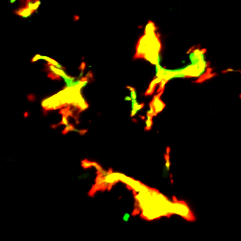

Supplement: Supplementary file 8 — Appendix Figure Source Data [file 44318_2024_78_MOESM8_ESM.zip › Appendix Figure/Appendix Figure S7/S7B/sGC╬öpc tdTomato-7.tif]

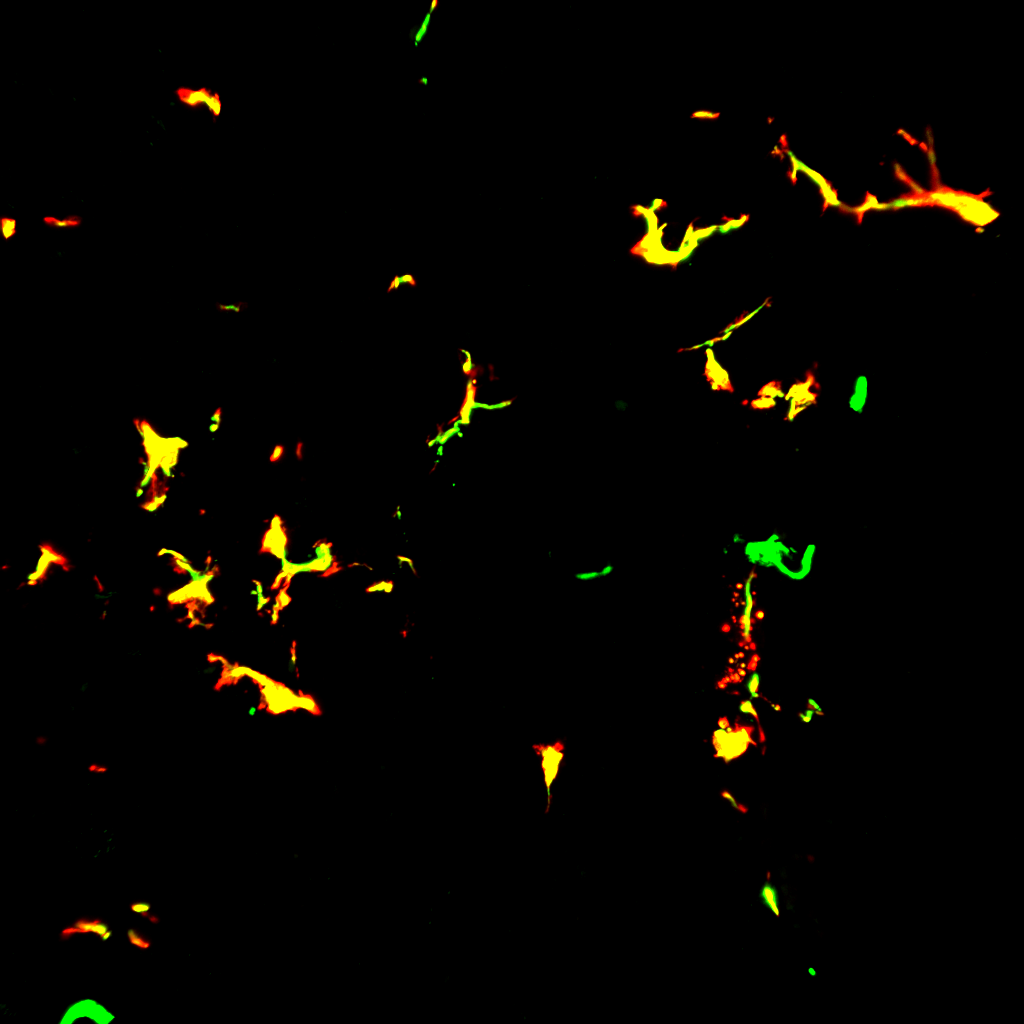

Supplement: Supplementary file 8 — Appendix Figure Source Data [file 44318_2024_78_MOESM8_ESM.zip › Appendix Figure/Appendix Figure S7/S7B/sGC╬öpc tdTomato-6.tif]

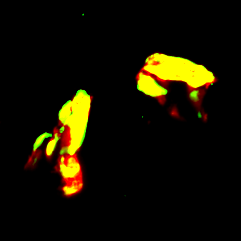

Supplement: Supplementary file 8 — Appendix Figure Source Data [file 44318_2024_78_MOESM8_ESM.zip › Appendix Figure/Appendix Figure S7/S7B/sGCCtr tdTomato-7.tif]

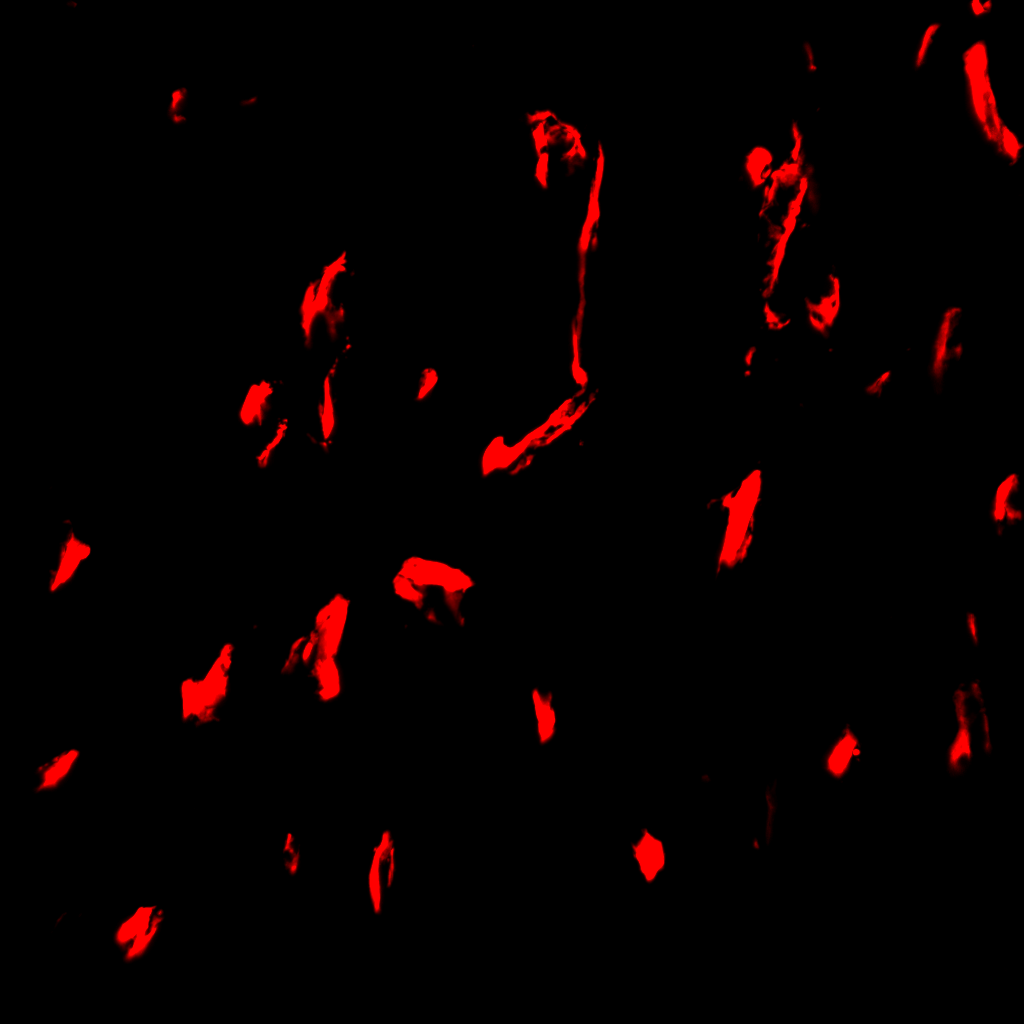

Supplement: Supplementary file 8 — Appendix Figure Source Data [file 44318_2024_78_MOESM8_ESM.zip › Appendix Figure/Appendix Figure S7/S7B/sGCCtr tdTomato-3.tif]

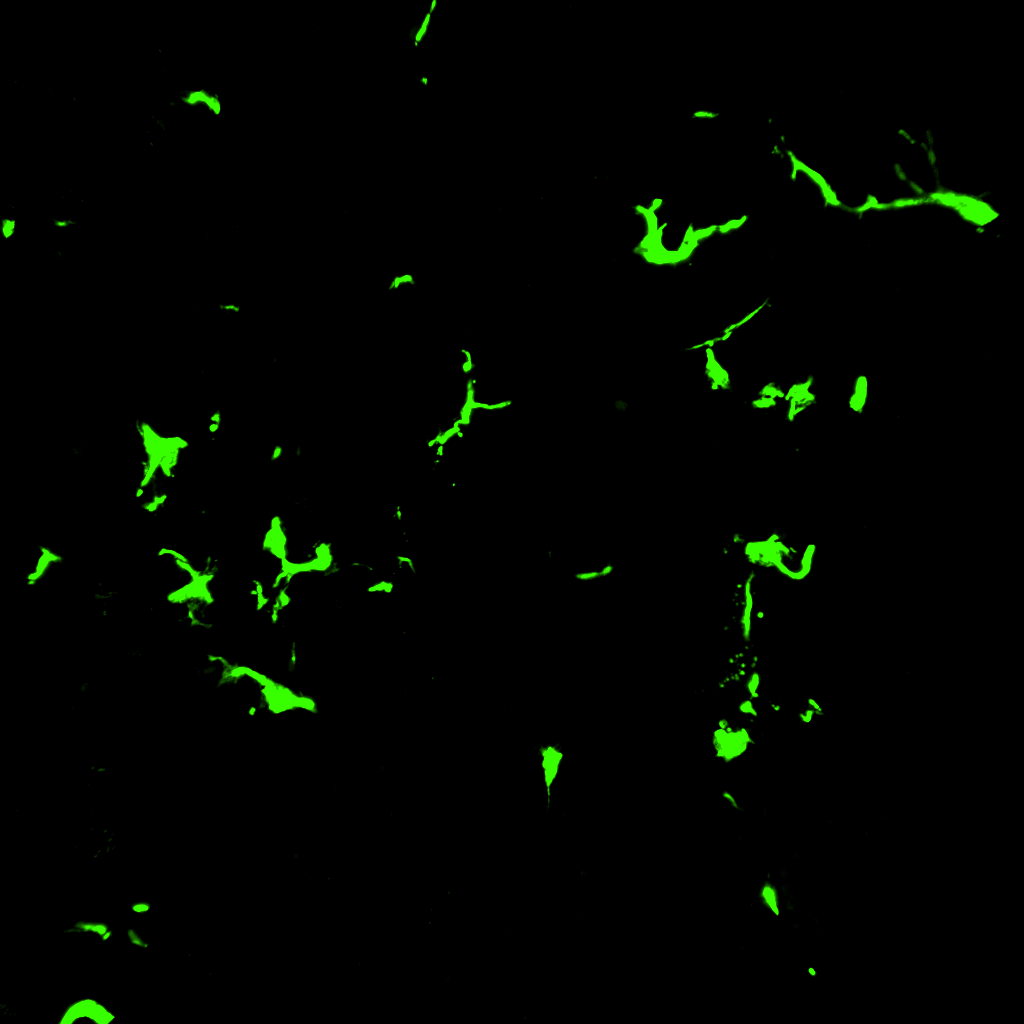

Supplement: Supplementary file 8 — Appendix Figure Source Data [file 44318_2024_78_MOESM8_ESM.zip › Appendix Figure/Appendix Figure S7/S7B/sGC╬öpc tdTomato-2.tif]

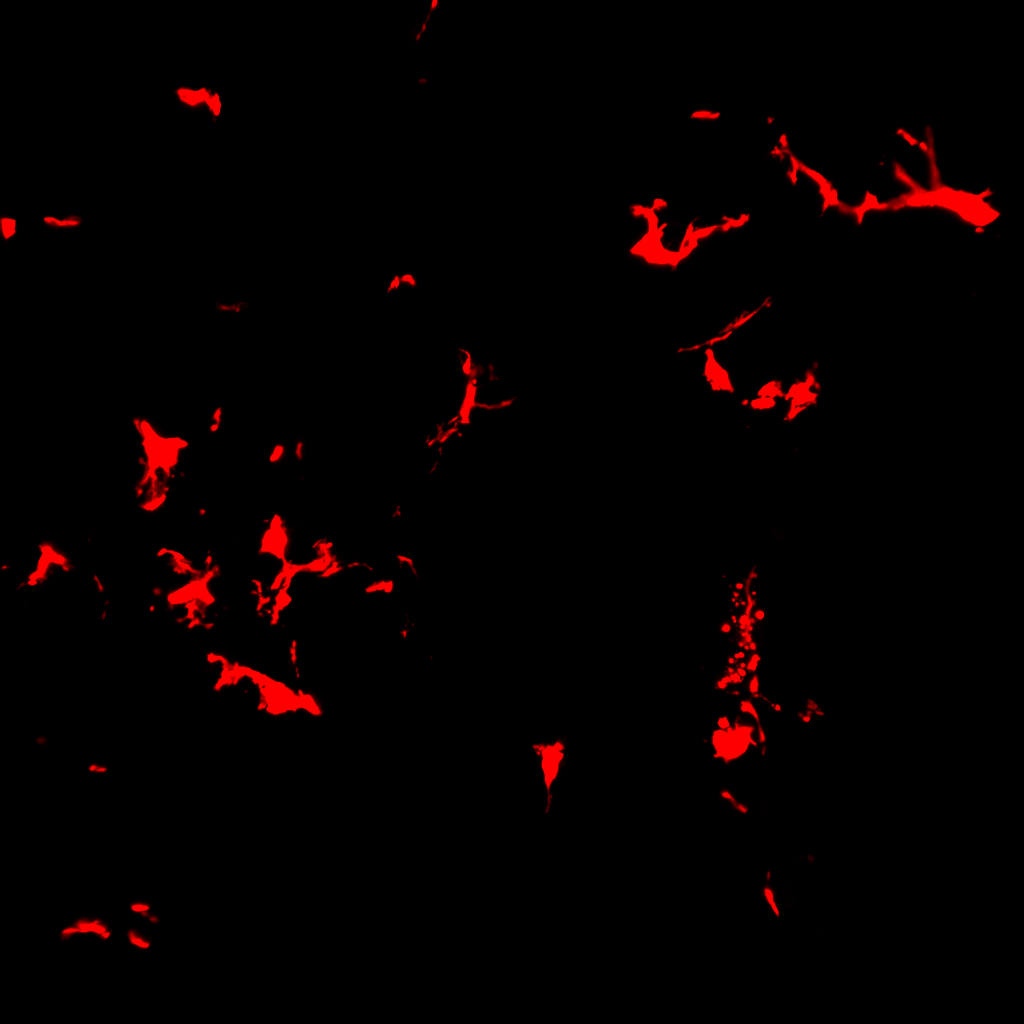

Supplement: Supplementary file 8 — Appendix Figure Source Data [file 44318_2024_78_MOESM8_ESM.zip › Appendix Figure/Appendix Figure S7/S7B/sGC╬öpc tdTomato-3.tif]

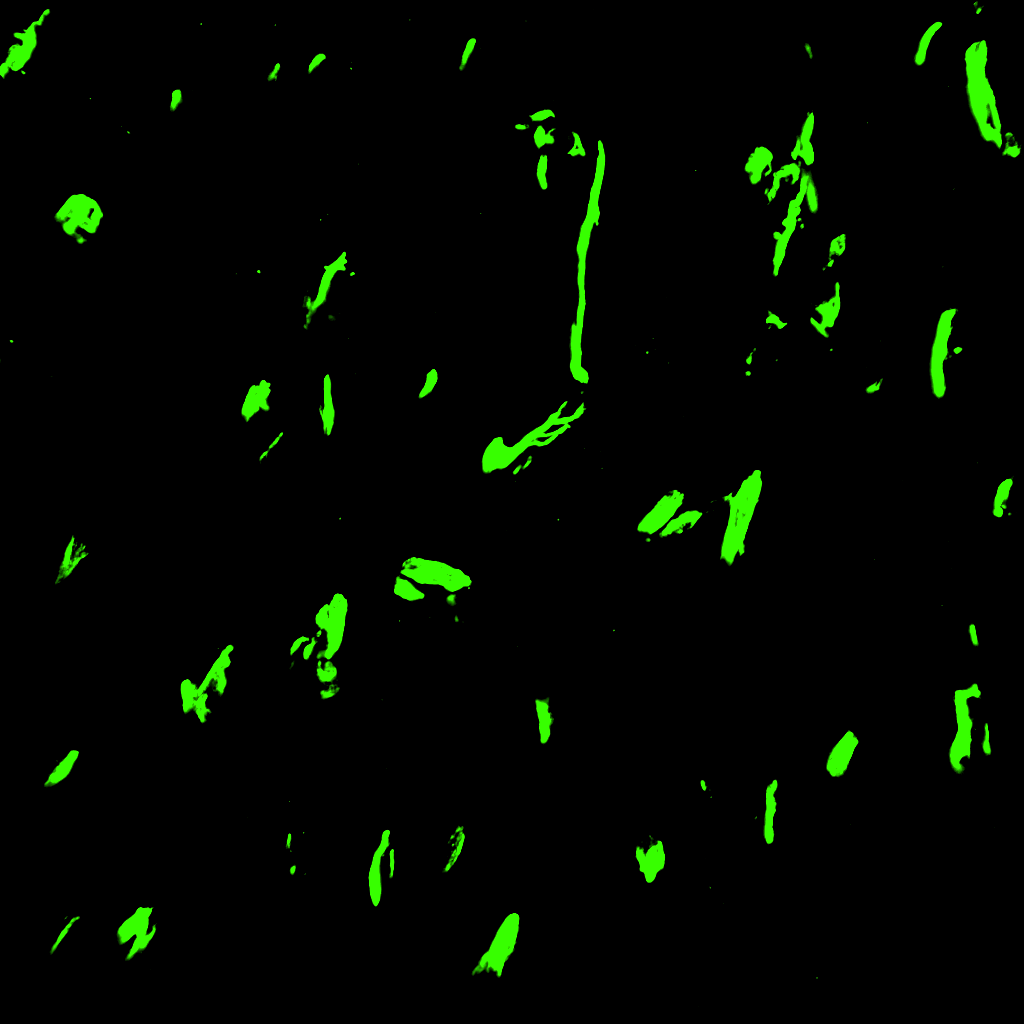

Supplement: Supplementary file 8 — Appendix Figure Source Data [file 44318_2024_78_MOESM8_ESM.zip › Appendix Figure/Appendix Figure S7/S7B/sGCCtr tdTomato-2.tif]

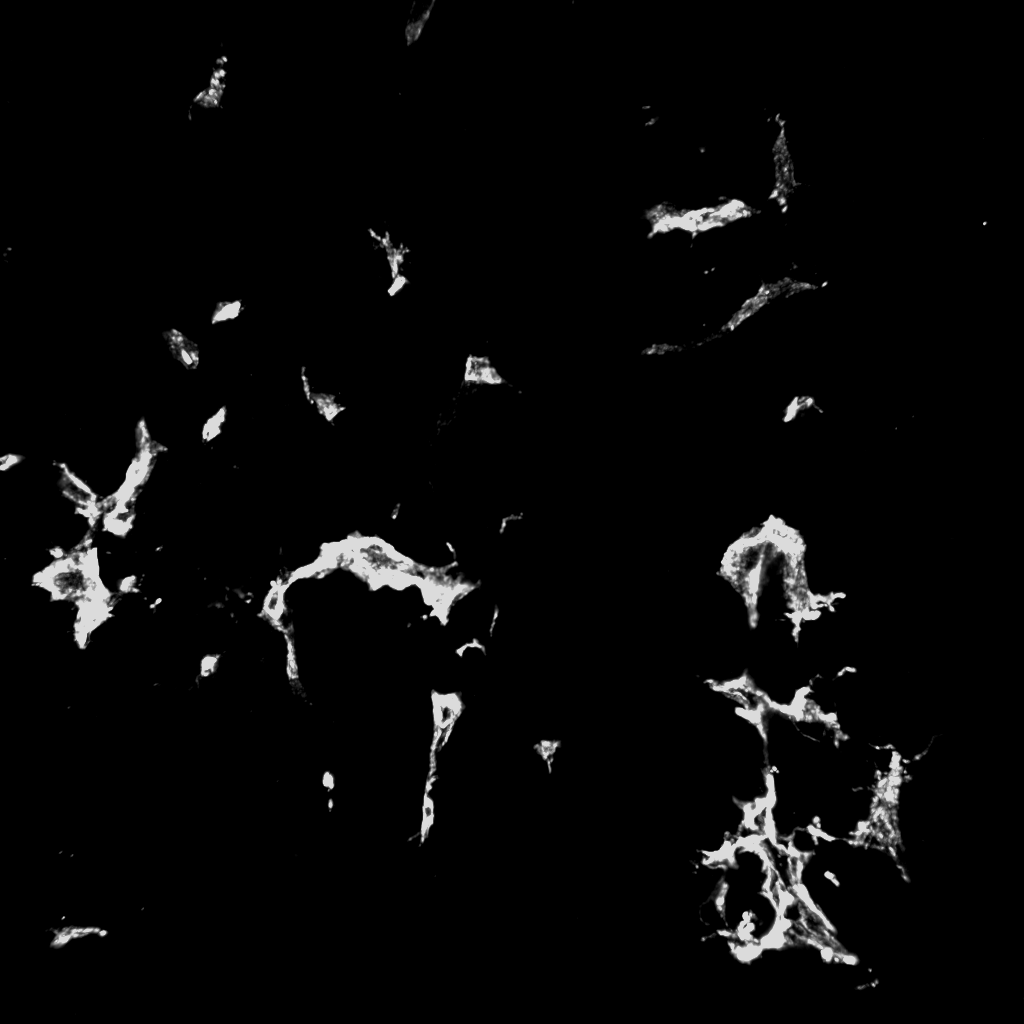

Supplement: Supplementary file 8 — Appendix Figure Source Data [file 44318_2024_78_MOESM8_ESM.zip › Appendix Figure/Appendix Figure S7/S7B/sGC╬öpc tdTomato-1.tif]

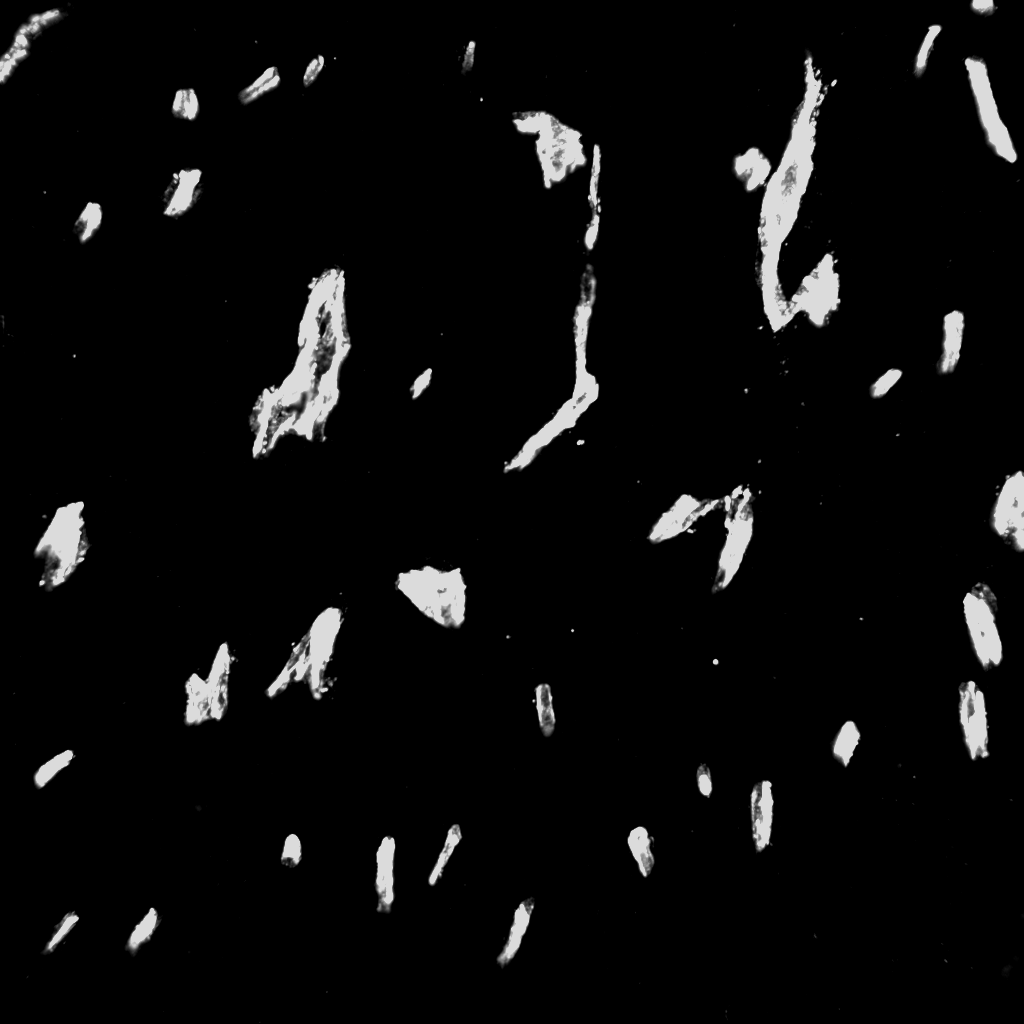

Supplement: Supplementary file 8 — Appendix Figure Source Data [file 44318_2024_78_MOESM8_ESM.zip › Appendix Figure/Appendix Figure S7/S7B/sGCCtr tdTomato-1.tif]

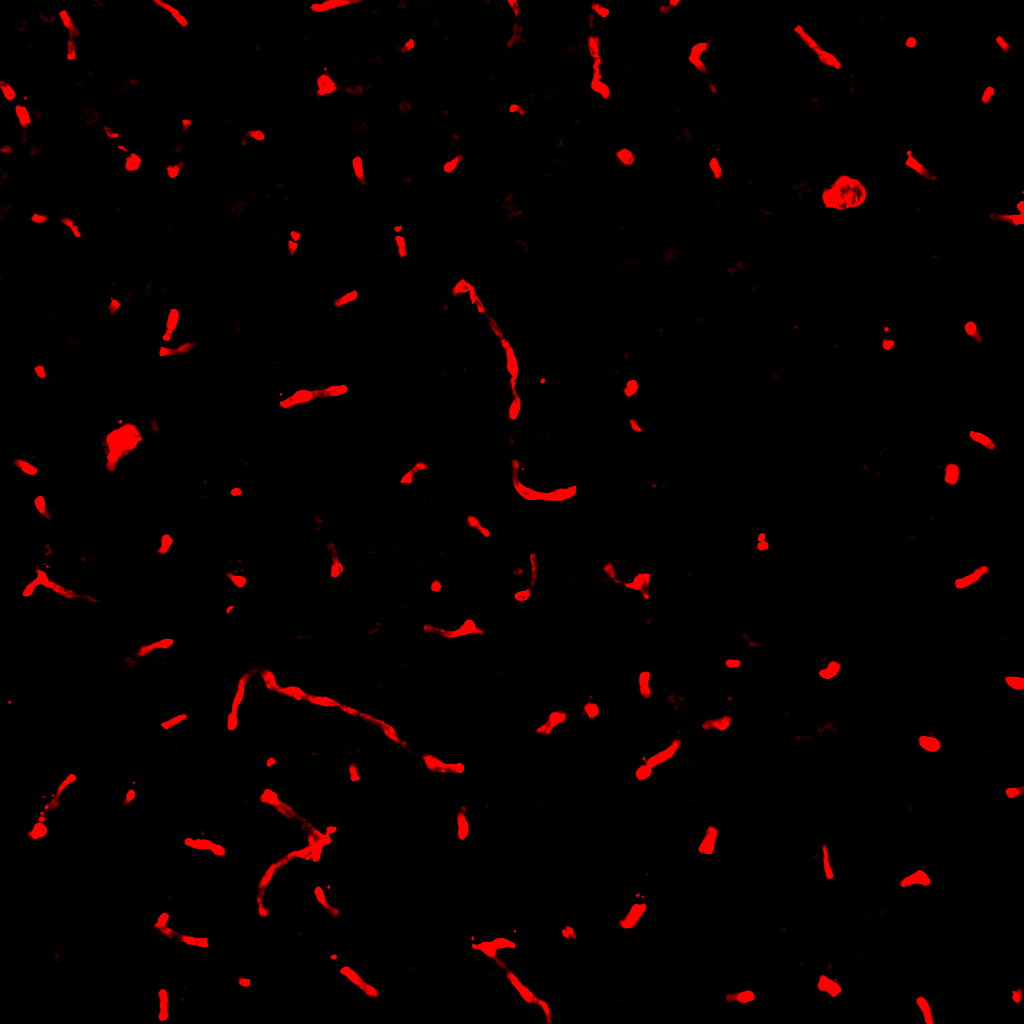

Supplement: Supplementary file 8 — Appendix Figure Source Data [file 44318_2024_78_MOESM8_ESM.zip › Appendix Figure/Appendix Figure S9/S9C/Control-2.tif]

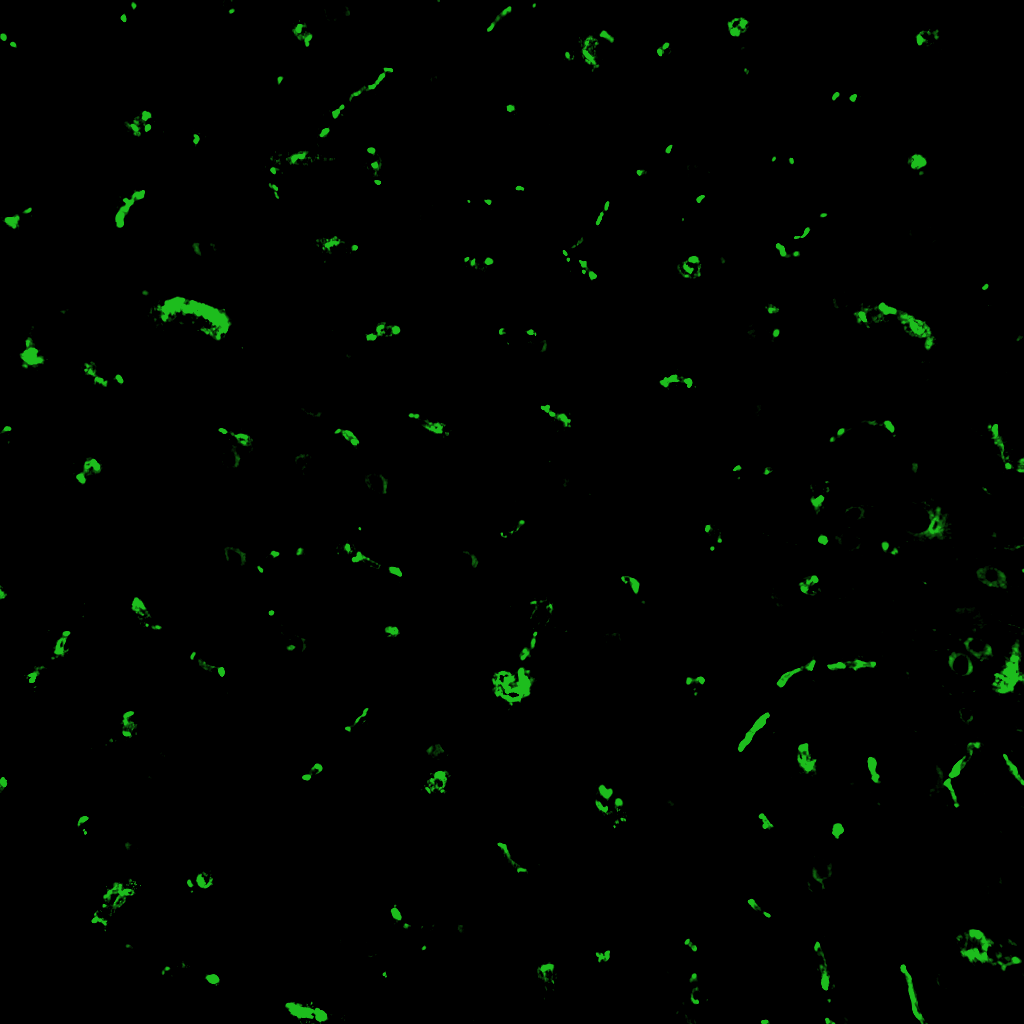

Supplement: Supplementary file 8 — Appendix Figure Source Data [file 44318_2024_78_MOESM8_ESM.zip › Appendix Figure/Appendix Figure S9/S9C/ODQ-1.tif]

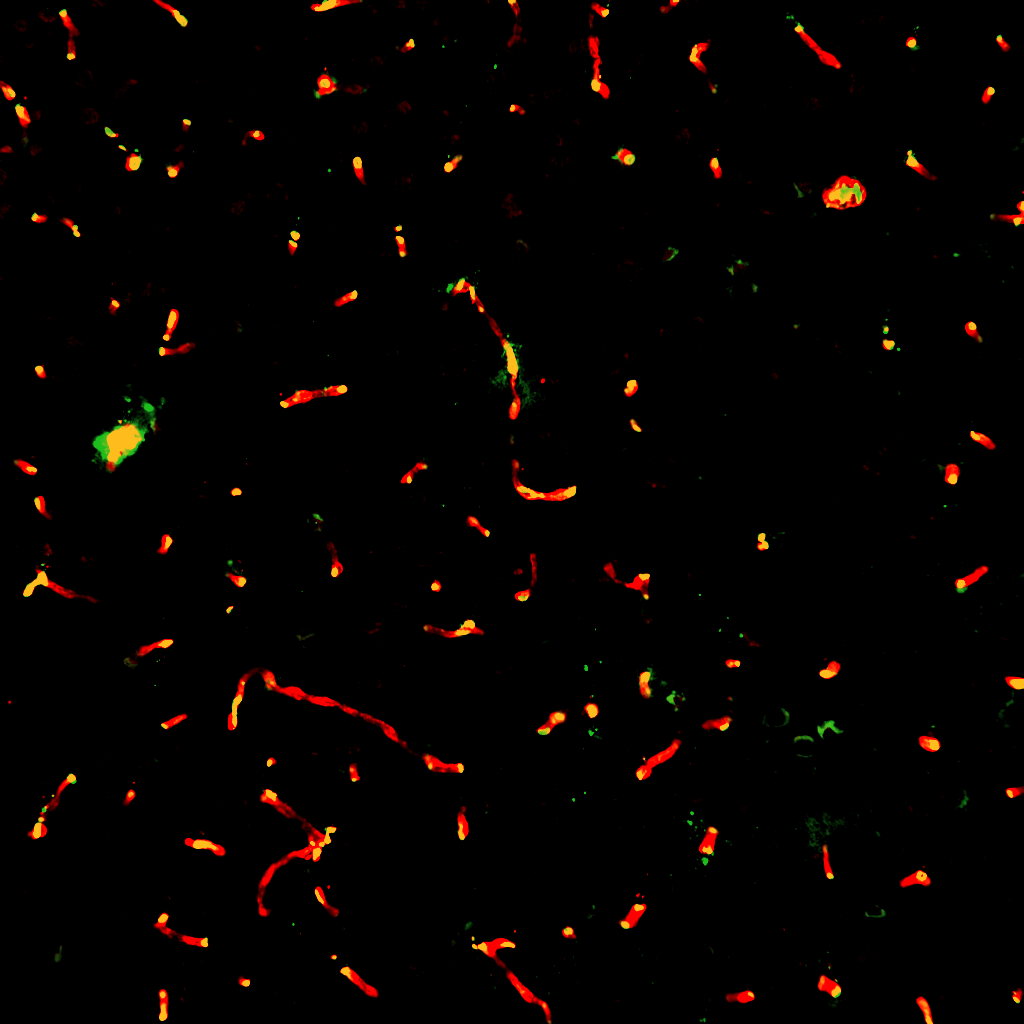

Supplement: Supplementary file 8 — Appendix Figure Source Data [file 44318_2024_78_MOESM8_ESM.zip › Appendix Figure/Appendix Figure S9/S9C/Control-3.tif]

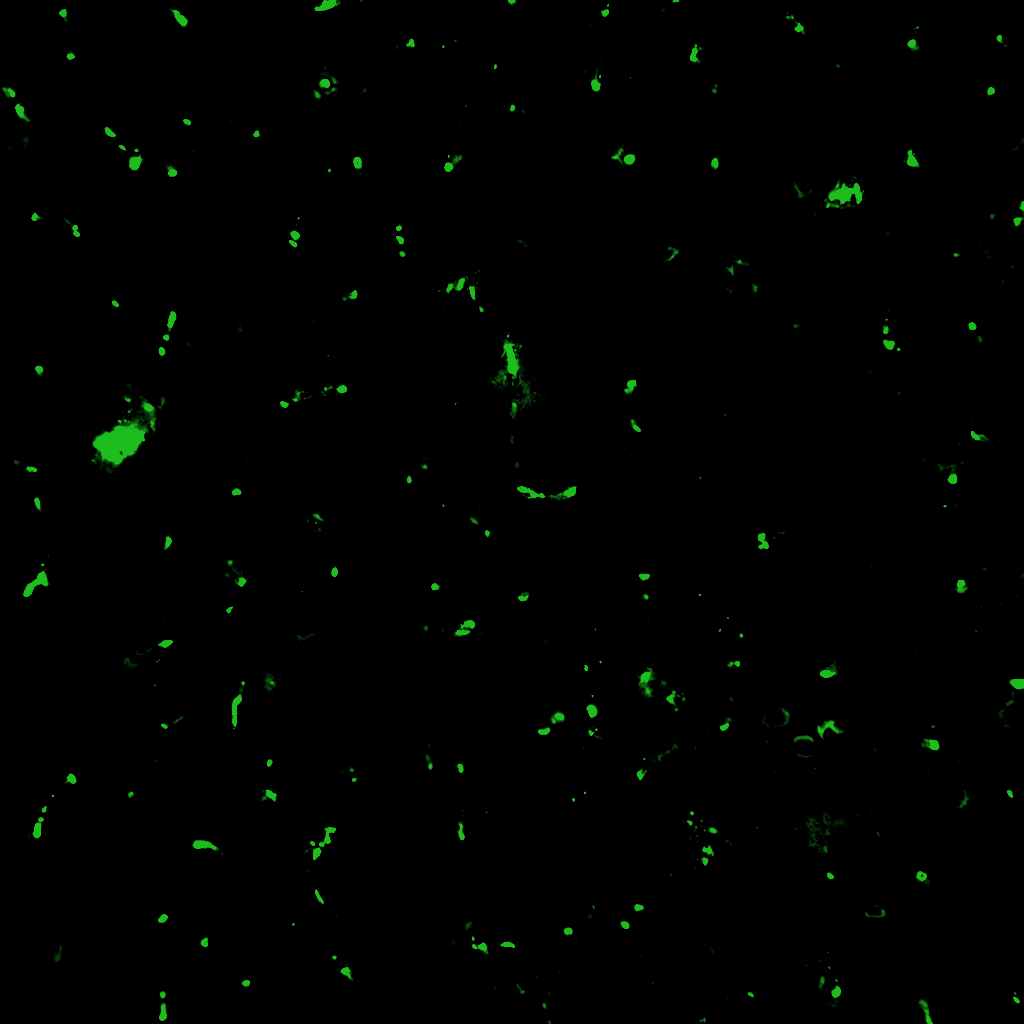

Supplement: Supplementary file 8 — Appendix Figure Source Data [file 44318_2024_78_MOESM8_ESM.zip › Appendix Figure/Appendix Figure S9/S9C/Control-1.tif]

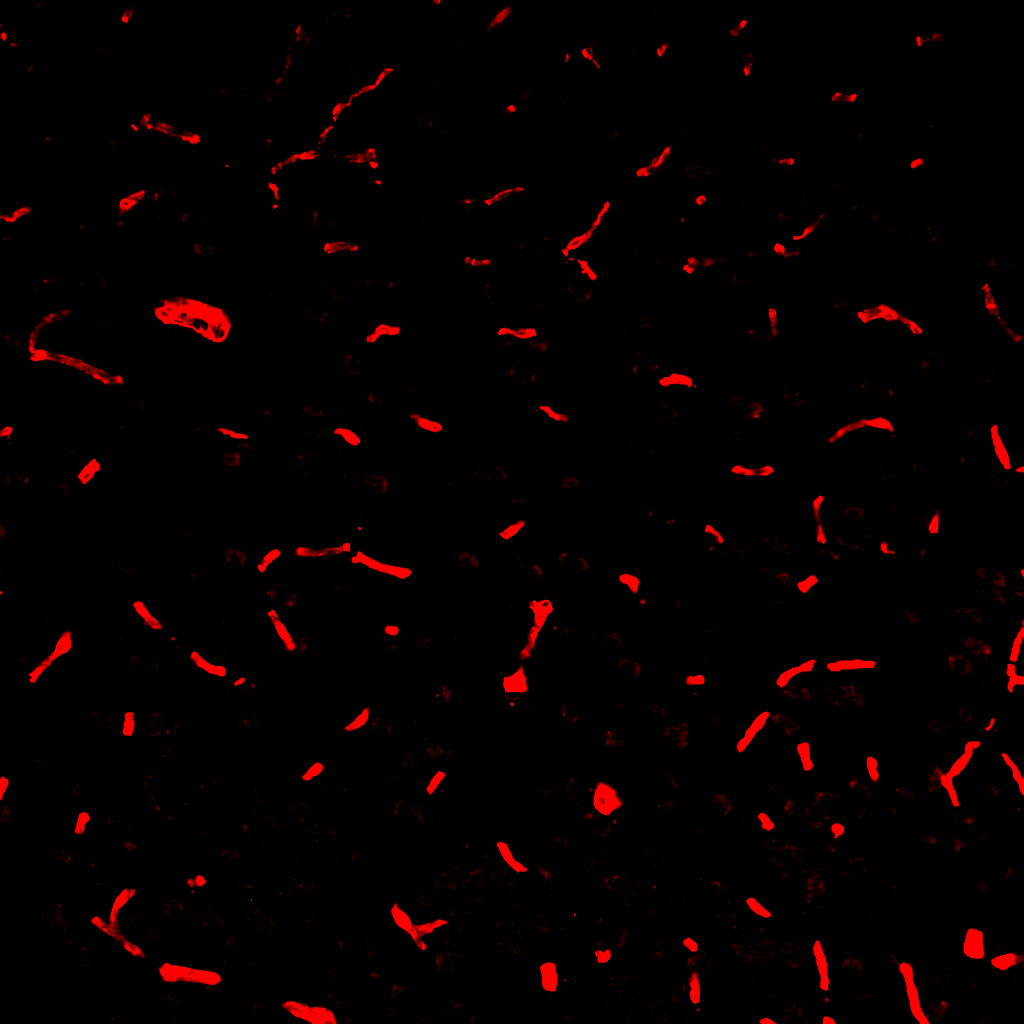

Supplement: Supplementary file 8 — Appendix Figure Source Data [file 44318_2024_78_MOESM8_ESM.zip › Appendix Figure/Appendix Figure S9/S9C/ODQ-2.tif]

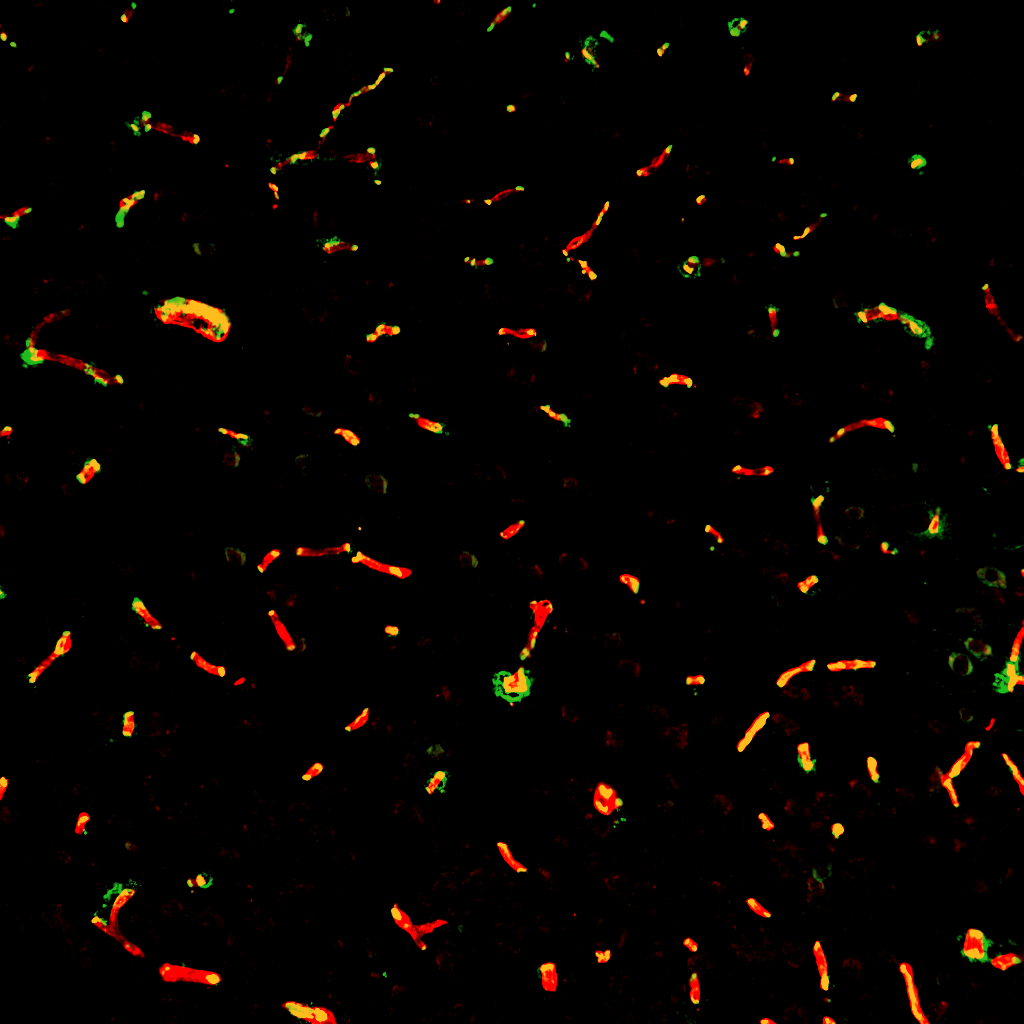

Supplement: Supplementary file 8 — Appendix Figure Source Data [file 44318_2024_78_MOESM8_ESM.zip › Appendix Figure/Appendix Figure S9/S9C/ODQ-3.tif]

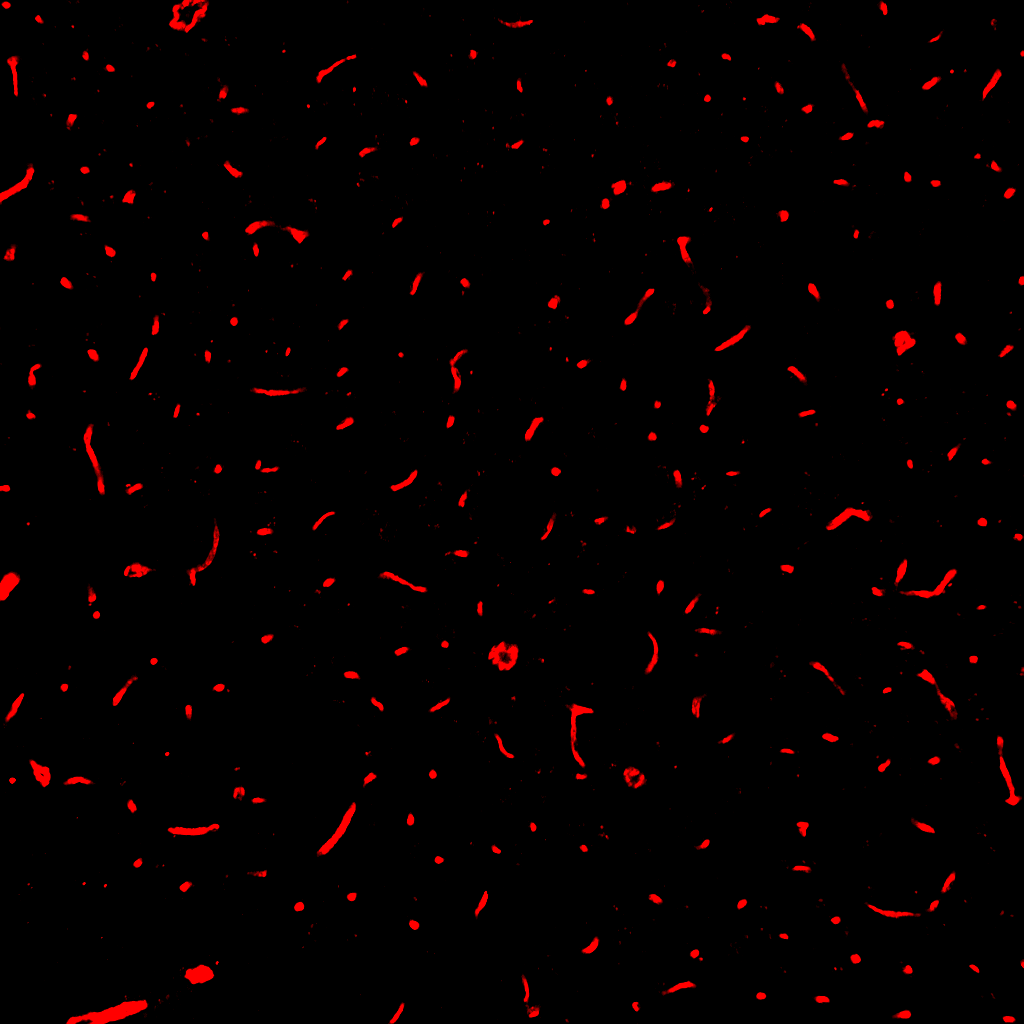

Supplement: Supplementary file 8 — Appendix Figure Source Data [file 44318_2024_78_MOESM8_ESM.zip › Appendix Figure/Appendix Figure S9/S9B/Control-2.tif]

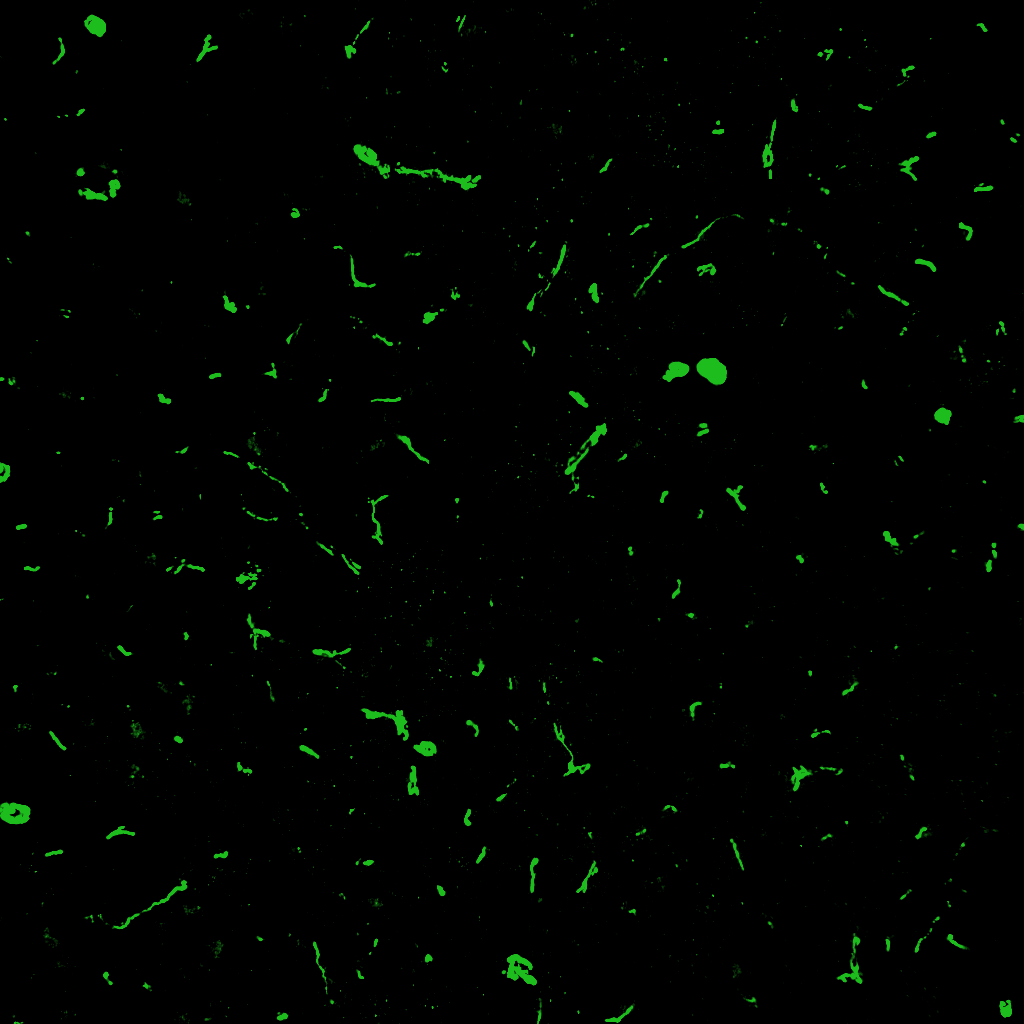

Supplement: Supplementary file 8 — Appendix Figure Source Data [file 44318_2024_78_MOESM8_ESM.zip › Appendix Figure/Appendix Figure S9/S9B/ODQ-1.tif]

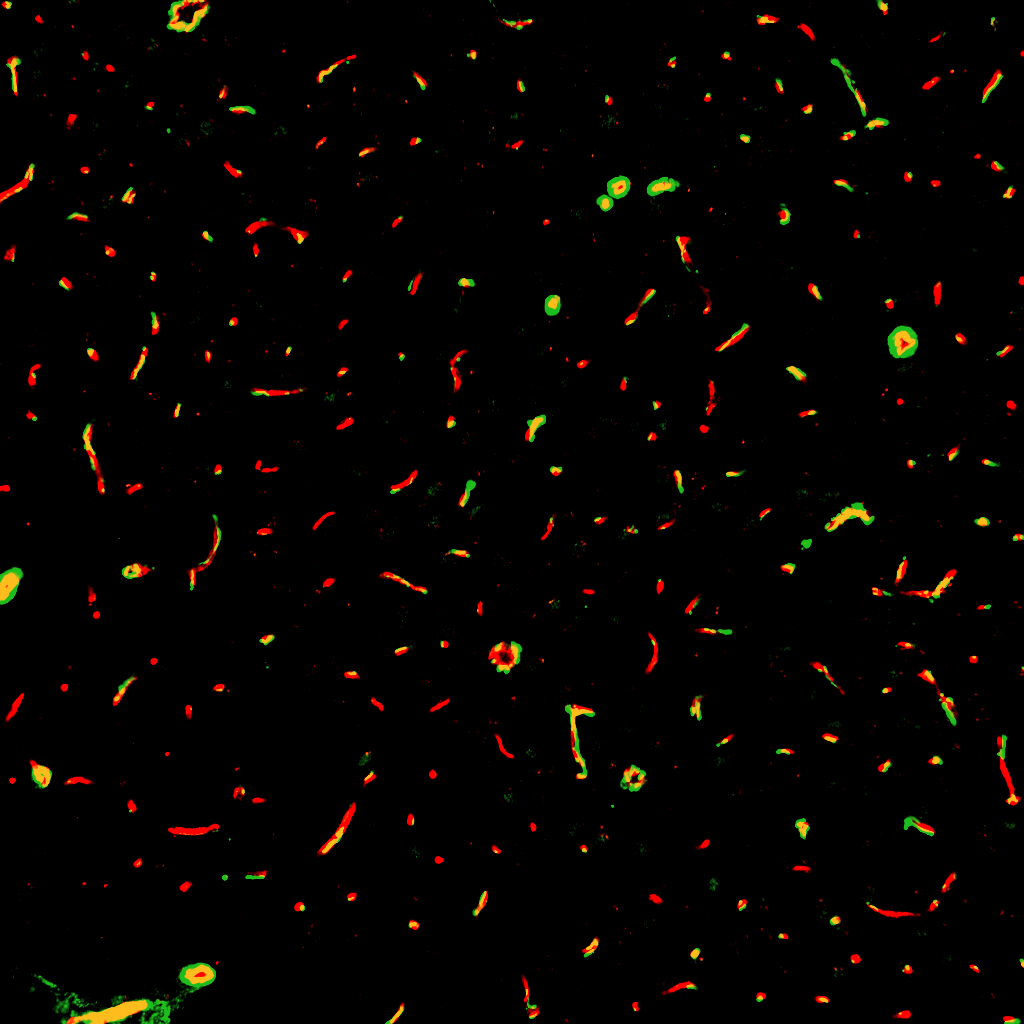

Supplement: Supplementary file 8 — Appendix Figure Source Data [file 44318_2024_78_MOESM8_ESM.zip › Appendix Figure/Appendix Figure S9/S9B/Control-3.tif]

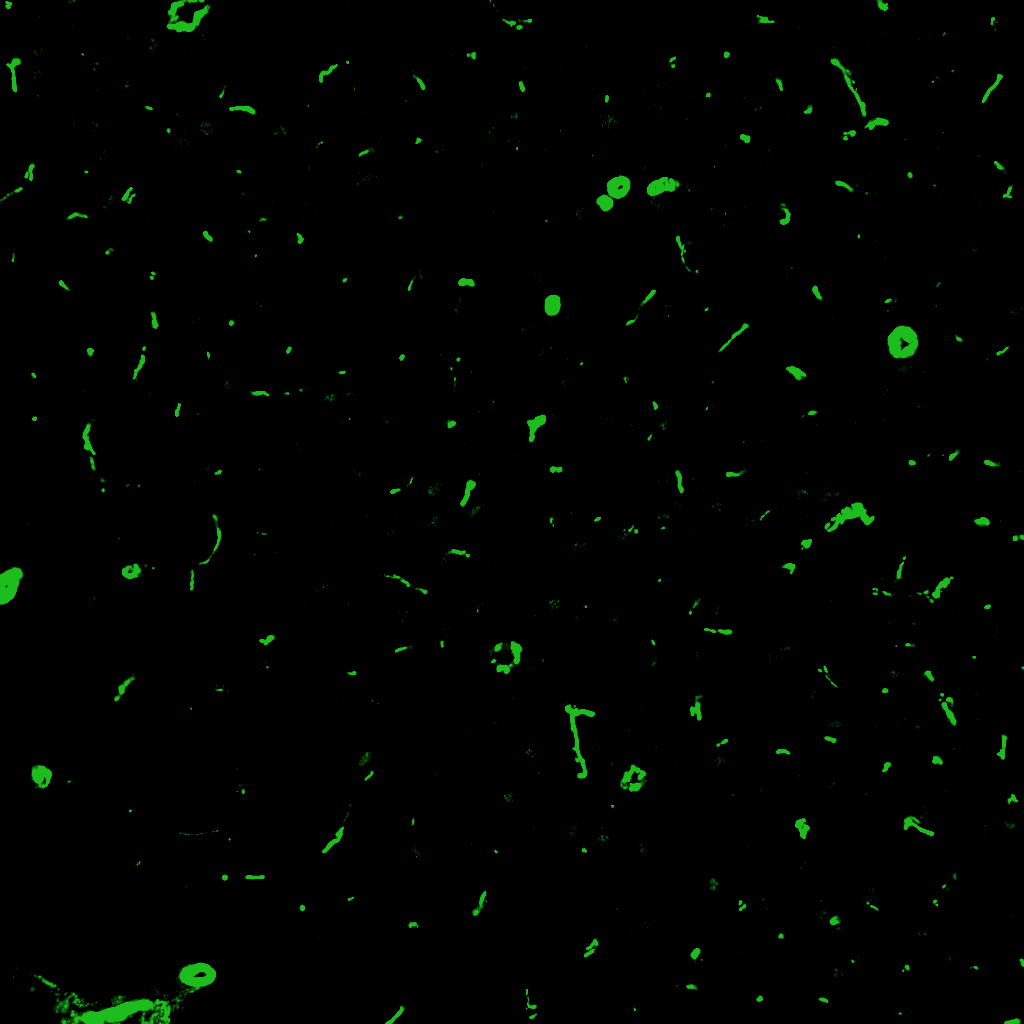

Supplement: Supplementary file 8 — Appendix Figure Source Data [file 44318_2024_78_MOESM8_ESM.zip › Appendix Figure/Appendix Figure S9/S9B/Control-1.tif]

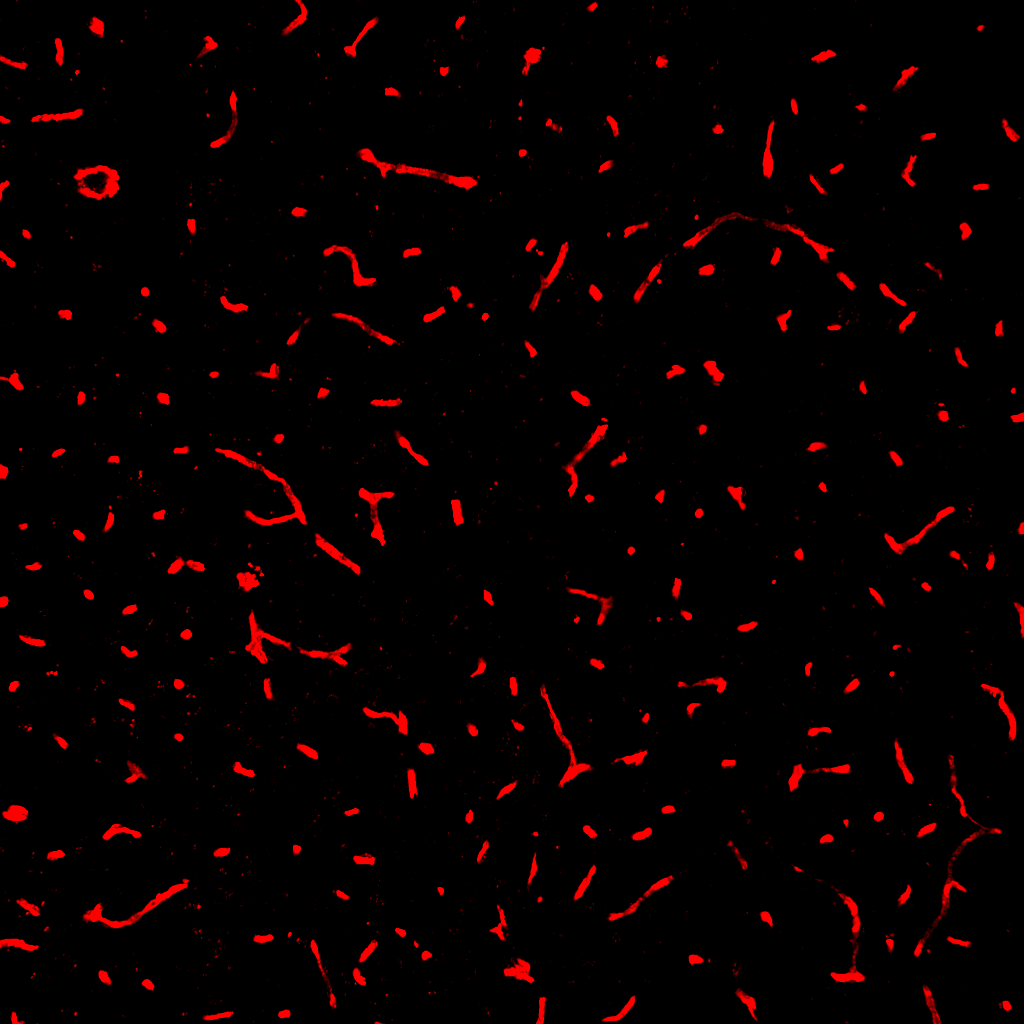

Supplement: Supplementary file 8 — Appendix Figure Source Data [file 44318_2024_78_MOESM8_ESM.zip › Appendix Figure/Appendix Figure S9/S9B/ODQ-2.tif]

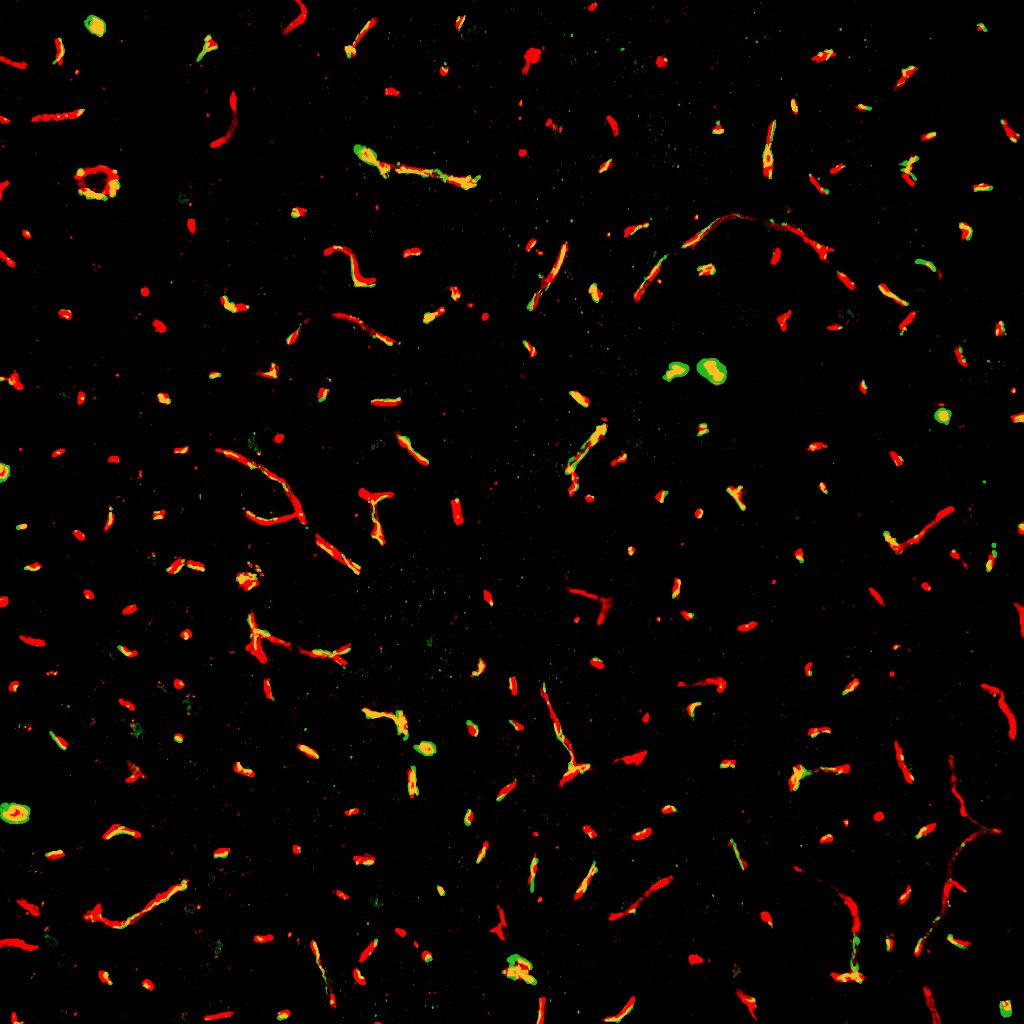

Supplement: Supplementary file 8 — Appendix Figure Source Data [file 44318_2024_78_MOESM8_ESM.zip › Appendix Figure/Appendix Figure S9/S9B/ODQ-3.tif]

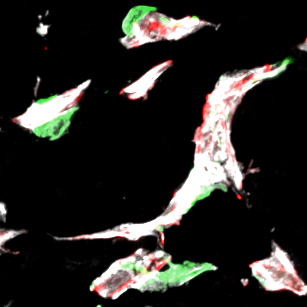

Supplement: Supplementary file 8 — Appendix Figure Source Data [file 44318_2024_78_MOESM8_ESM.zip › Appendix Figure/Appendix Figure S8/S8B/8.tif]

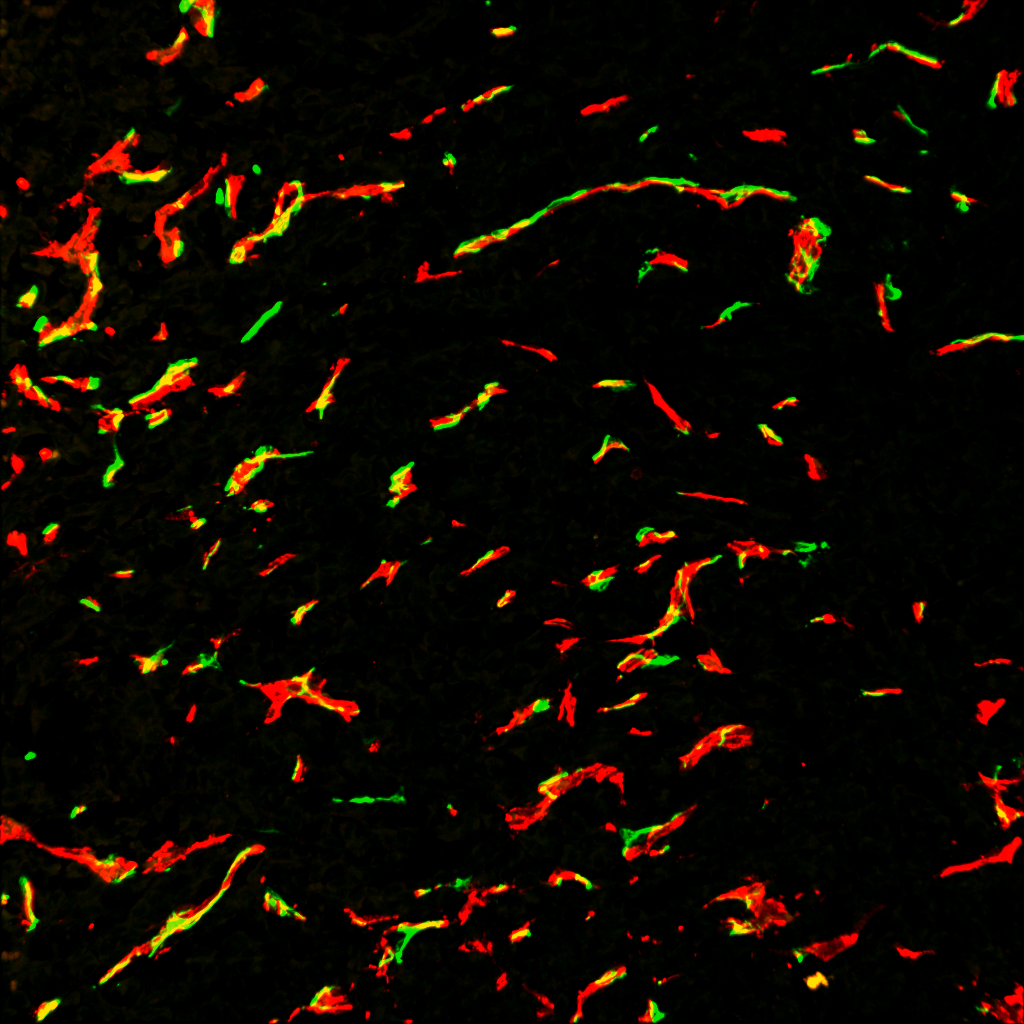

Supplement: Supplementary file 8 — Appendix Figure Source Data [file 44318_2024_78_MOESM8_ESM.zip › Appendix Figure/Appendix Figure S8/S8B/3.tif]

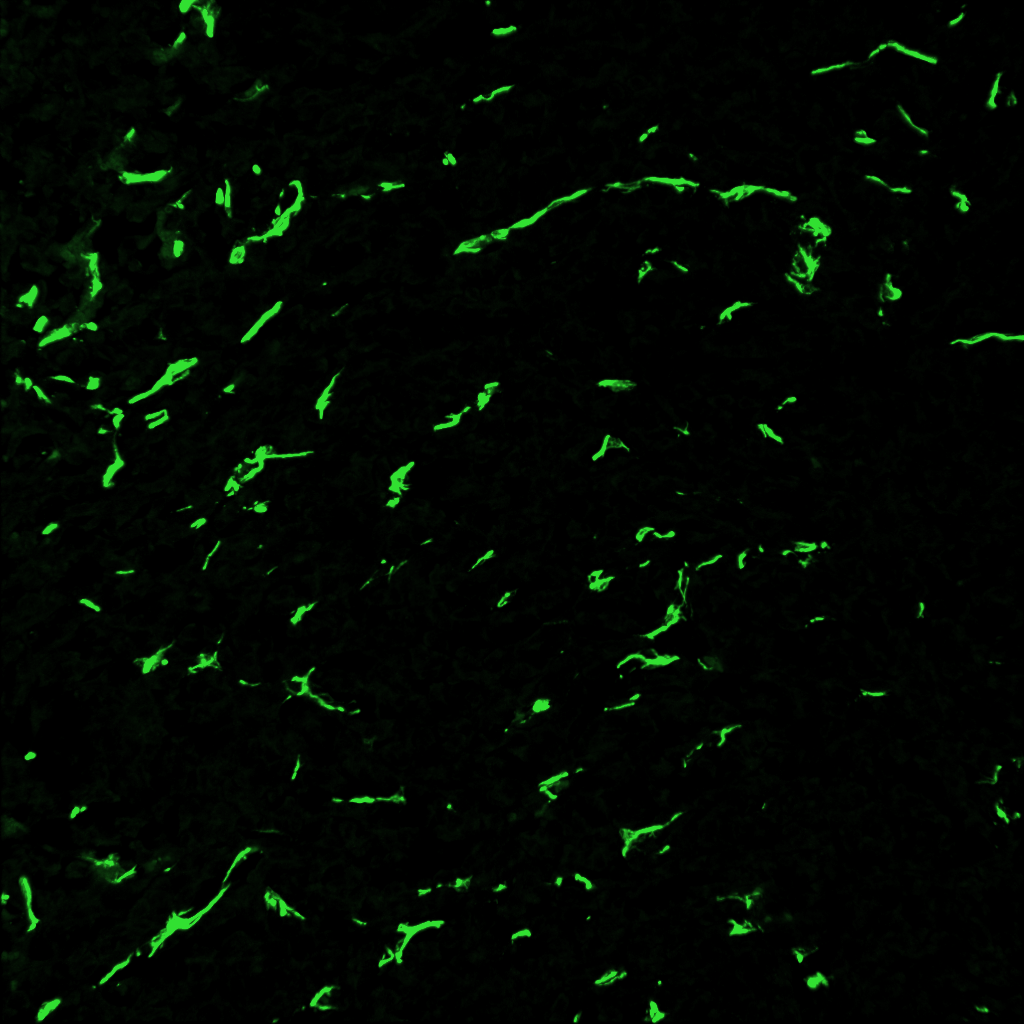

Supplement: Supplementary file 8 — Appendix Figure Source Data [file 44318_2024_78_MOESM8_ESM.zip › Appendix Figure/Appendix Figure S8/S8B/2.tif]

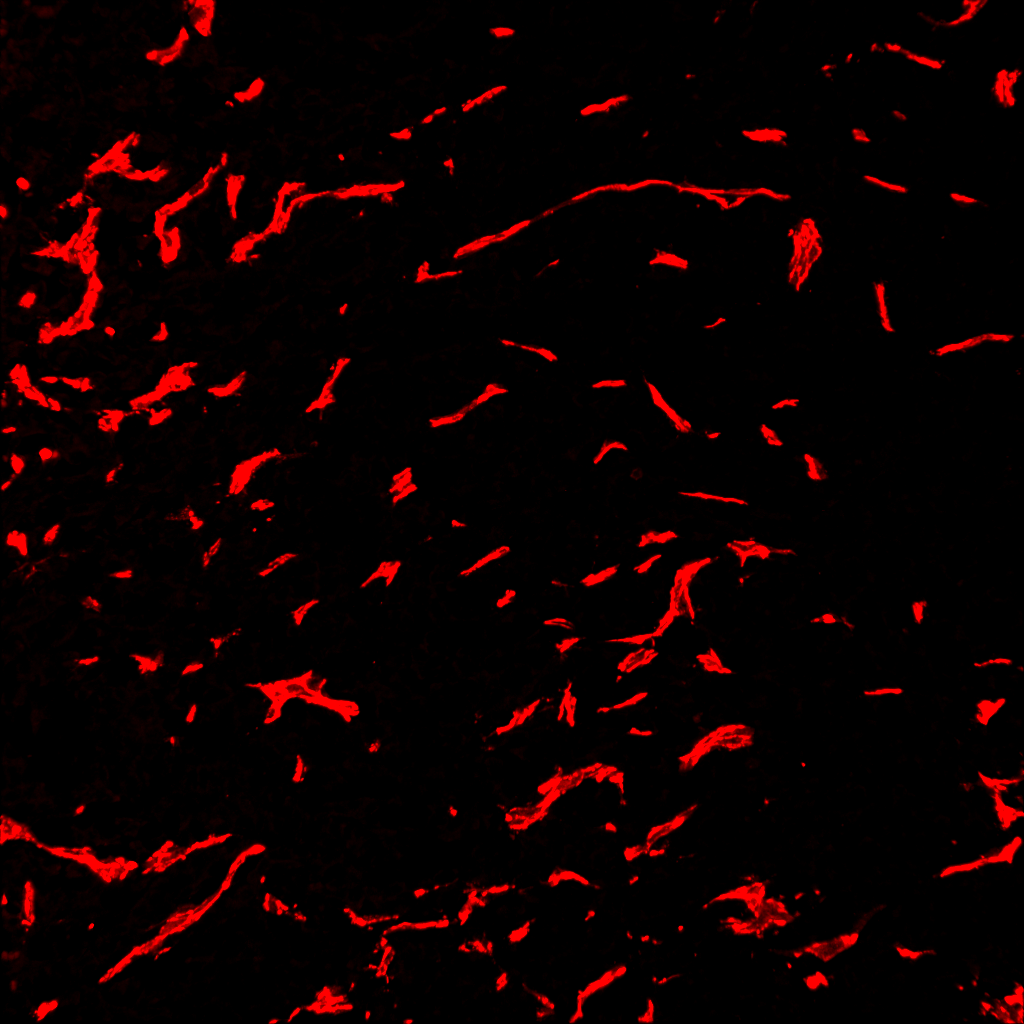

Supplement: Supplementary file 8 — Appendix Figure Source Data [file 44318_2024_78_MOESM8_ESM.zip › Appendix Figure/Appendix Figure S8/S8B/1.tif]

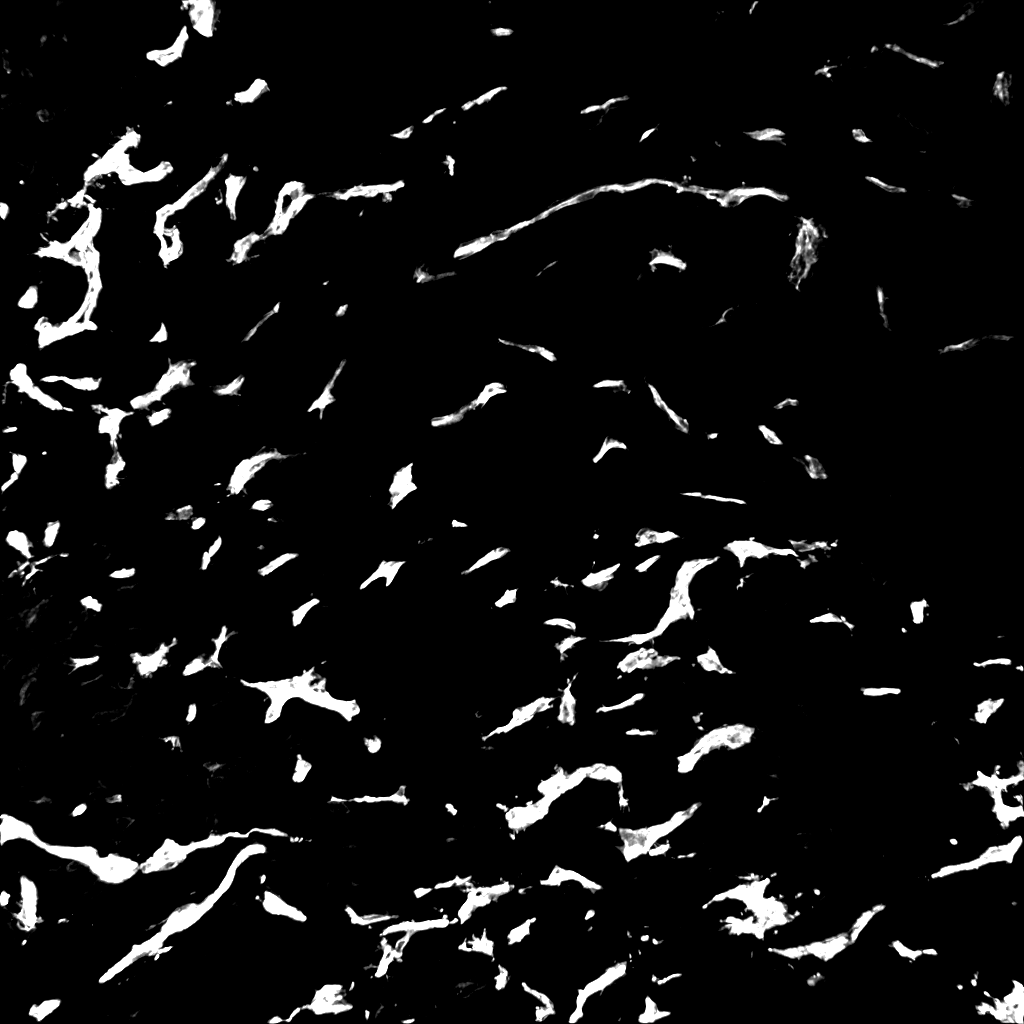

Supplement: Supplementary file 8 — Appendix Figure Source Data [file 44318_2024_78_MOESM8_ESM.zip › Appendix Figure/Appendix Figure S8/S8B/5.tif]

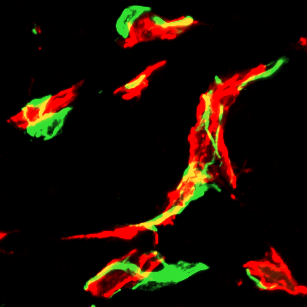

Supplement: Supplementary file 8 — Appendix Figure Source Data [file 44318_2024_78_MOESM8_ESM.zip › Appendix Figure/Appendix Figure S8/S8B/4.tif]

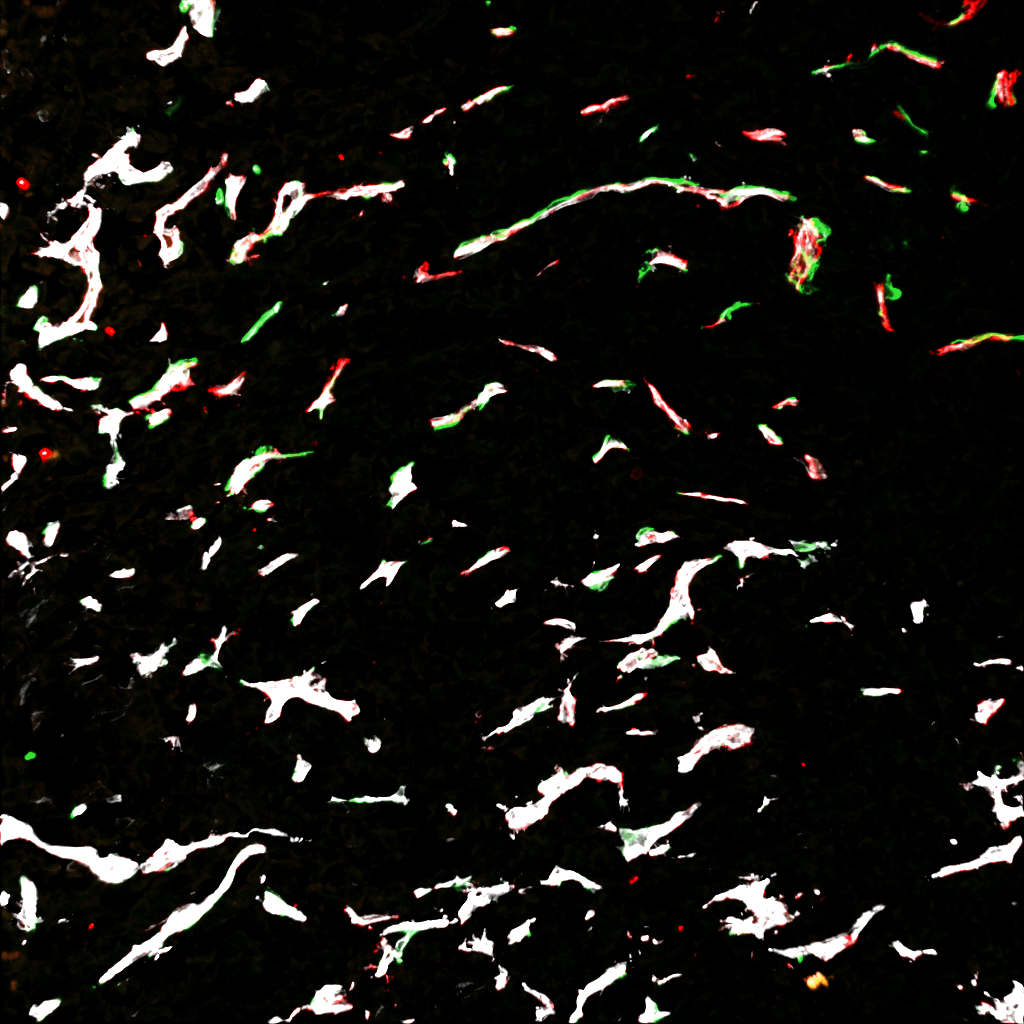

Supplement: Supplementary file 8 — Appendix Figure Source Data [file 44318_2024_78_MOESM8_ESM.zip › Appendix Figure/Appendix Figure S8/S8B/6.tif]

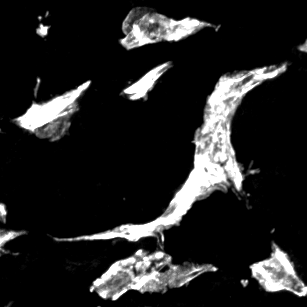

Supplement: Supplementary file 8 — Appendix Figure Source Data [file 44318_2024_78_MOESM8_ESM.zip › Appendix Figure/Appendix Figure S8/S8B/7.tif]

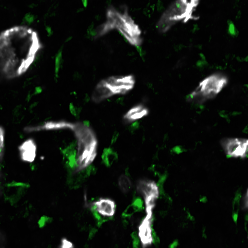

Supplement: Supplementary file 8 — Appendix Figure Source Data [file 44318_2024_78_MOESM8_ESM.zip › Appendix Figure/Appendix Figure S6/S6B/sGCCtr-4.tif]

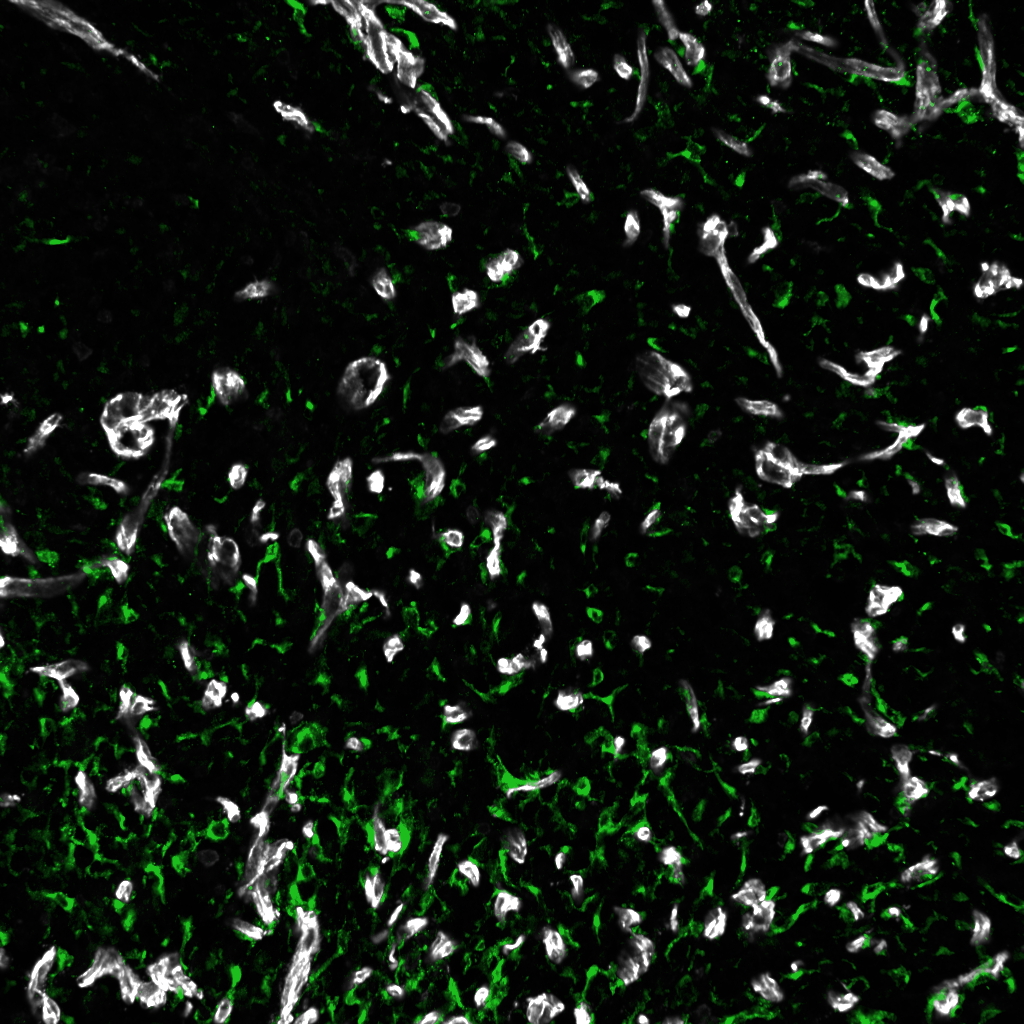

Supplement: Supplementary file 8 — Appendix Figure Source Data [file 44318_2024_78_MOESM8_ESM.zip › Appendix Figure/Appendix Figure S6/S6B/sGCCtr-3.tif]

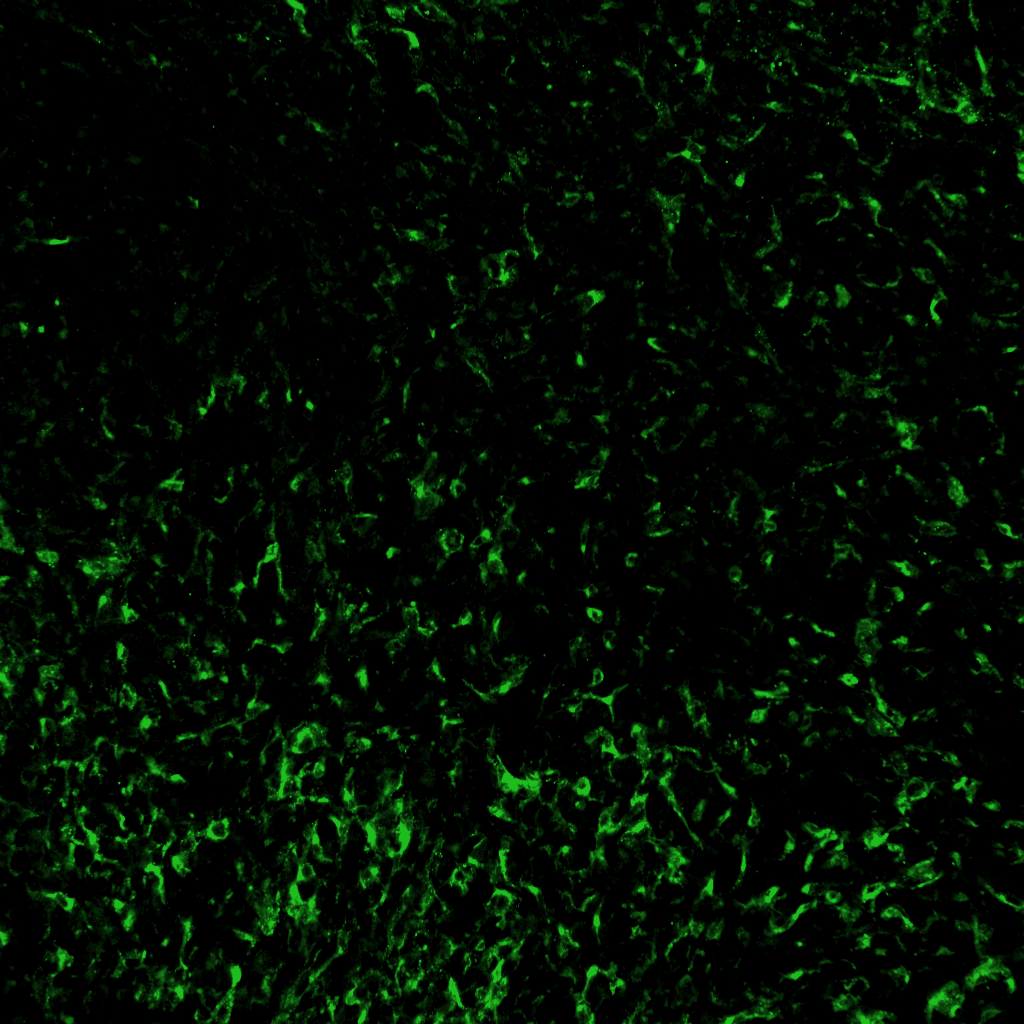

Supplement: Supplementary file 8 — Appendix Figure Source Data [file 44318_2024_78_MOESM8_ESM.zip › Appendix Figure/Appendix Figure S6/S6B/sGCCtr-2.tif]

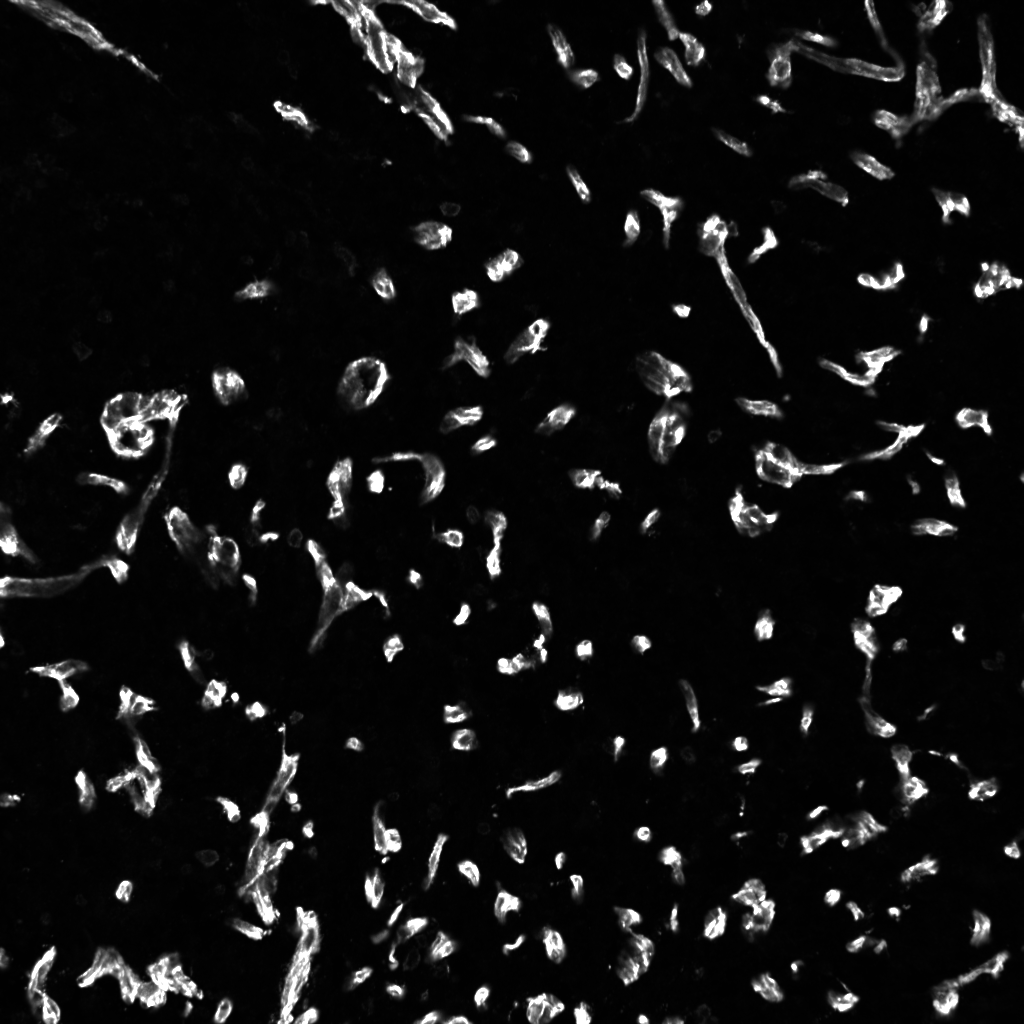

Supplement: Supplementary file 8 — Appendix Figure Source Data [file 44318_2024_78_MOESM8_ESM.zip › Appendix Figure/Appendix Figure S6/S6B/sGCCtr-1.tif]

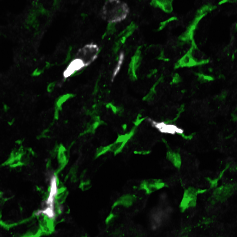

Supplement: Supplementary file 8 — Appendix Figure Source Data [file 44318_2024_78_MOESM8_ESM.zip › Appendix Figure/Appendix Figure S6/S6B/sGC╬öpc-4.tif]

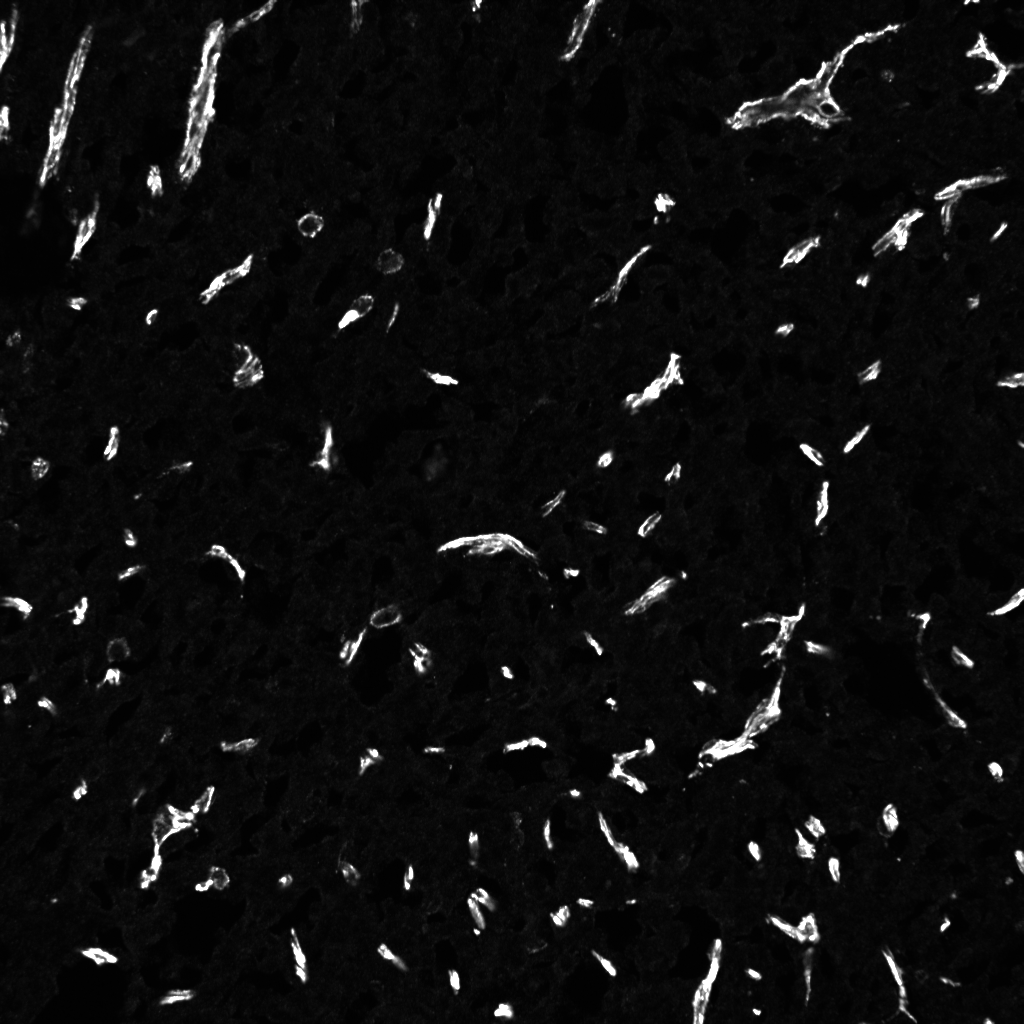

Supplement: Supplementary file 8 — Appendix Figure Source Data [file 44318_2024_78_MOESM8_ESM.zip › Appendix Figure/Appendix Figure S6/S6B/sGC╬öpc-1.tif]

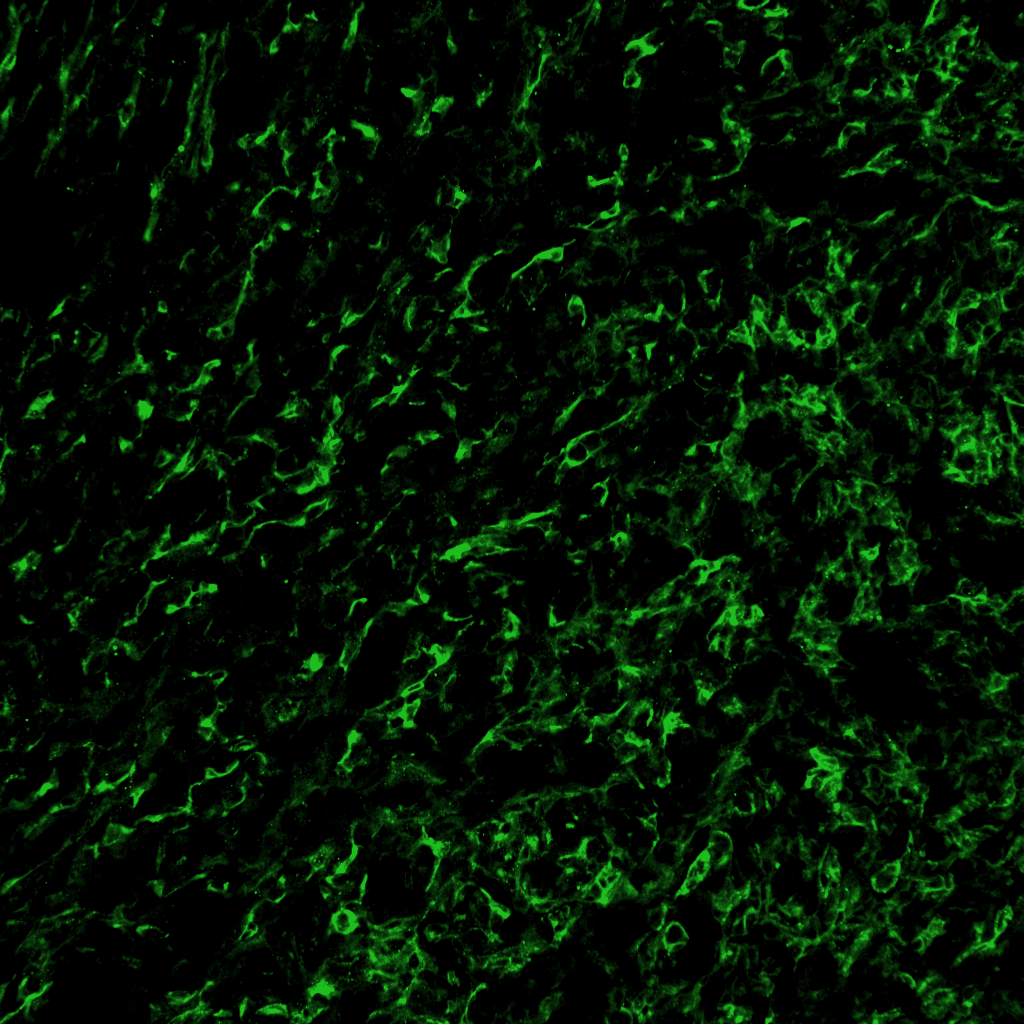

Supplement: Supplementary file 8 — Appendix Figure Source Data [file 44318_2024_78_MOESM8_ESM.zip › Appendix Figure/Appendix Figure S6/S6B/sGC╬öpc-2.tif]

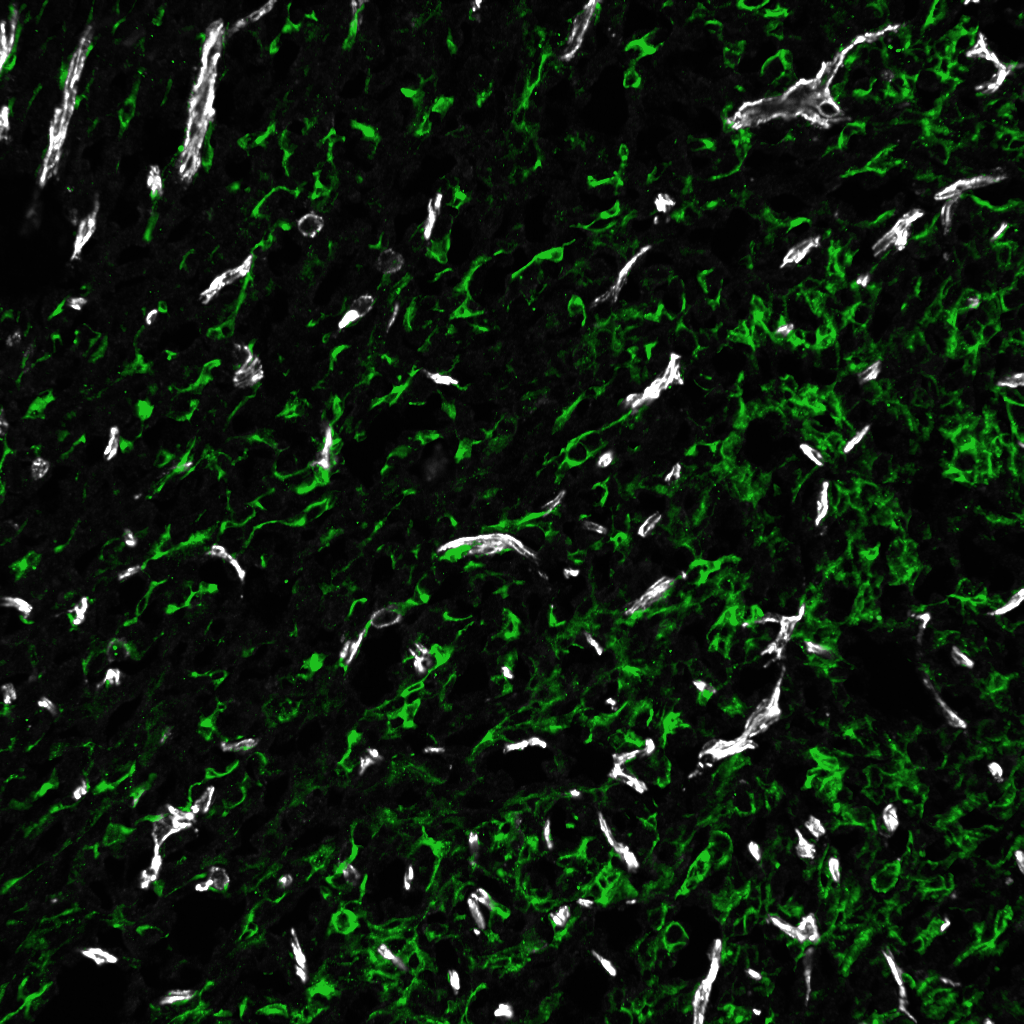

Supplement: Supplementary file 8 — Appendix Figure Source Data [file 44318_2024_78_MOESM8_ESM.zip › Appendix Figure/Appendix Figure S6/S6B/sGC╬öpc-3.tif]

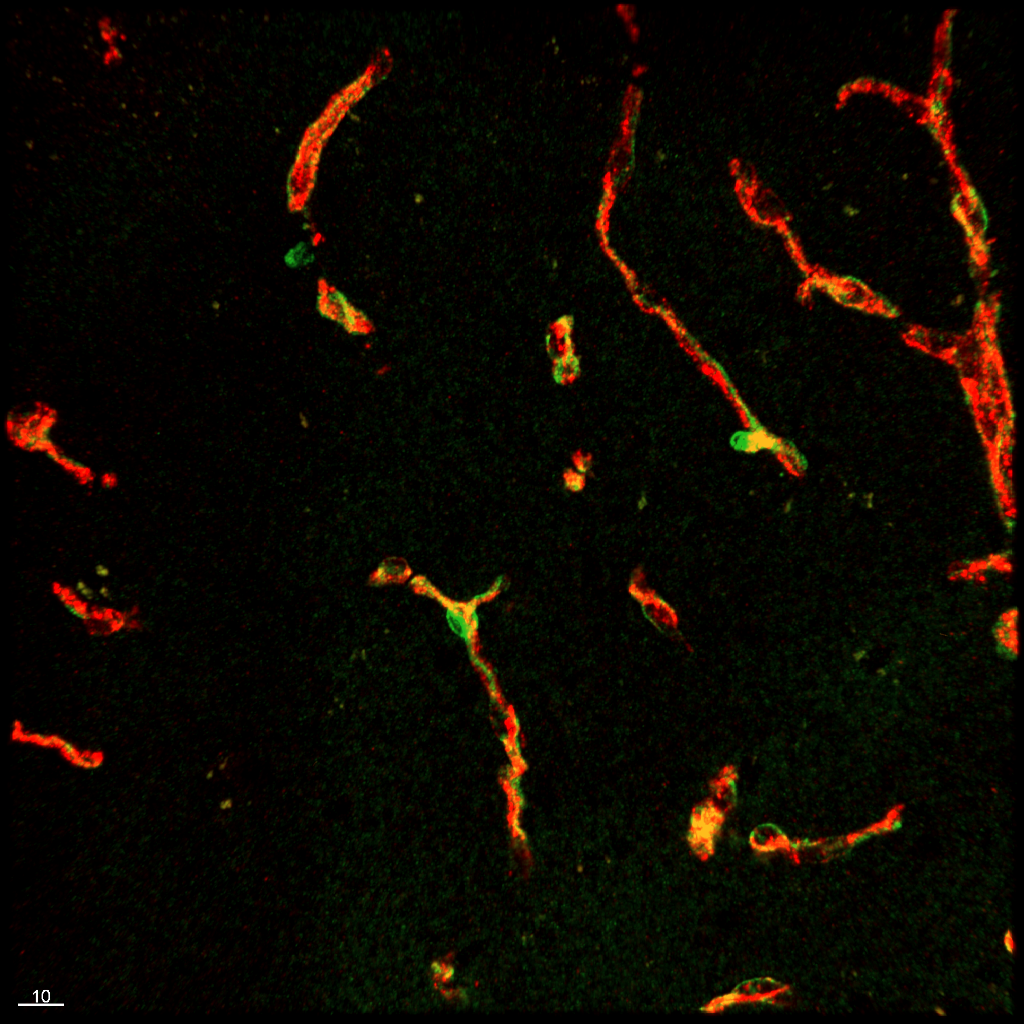

Supplement: Supplementary file 8 — Appendix Figure Source Data [file 44318_2024_78_MOESM8_ESM.zip › Appendix Figure/Appendix Figure S1/S1D/sGCCtr-4.tif]

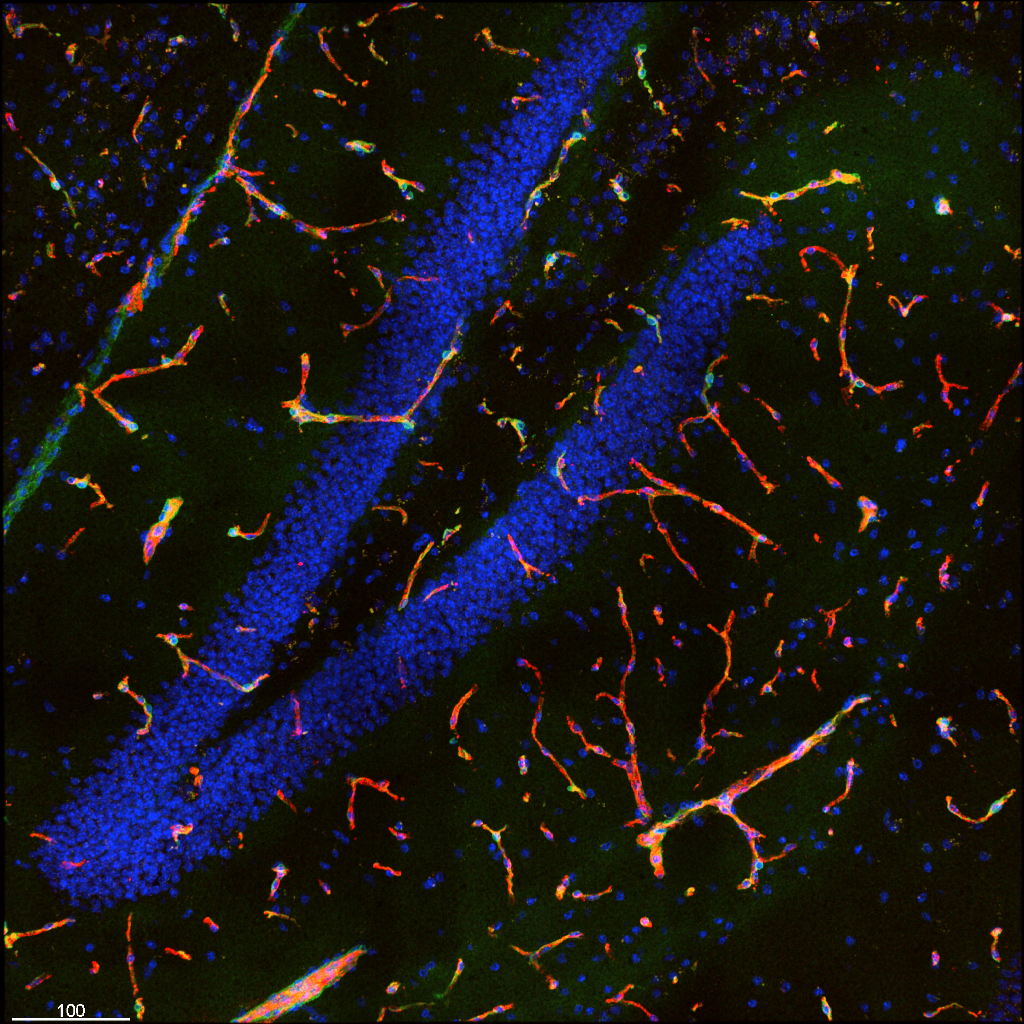

Supplement: Supplementary file 8 — Appendix Figure Source Data [file 44318_2024_78_MOESM8_ESM.zip › Appendix Figure/Appendix Figure S1/S1D/sGCCtr-3.tif]

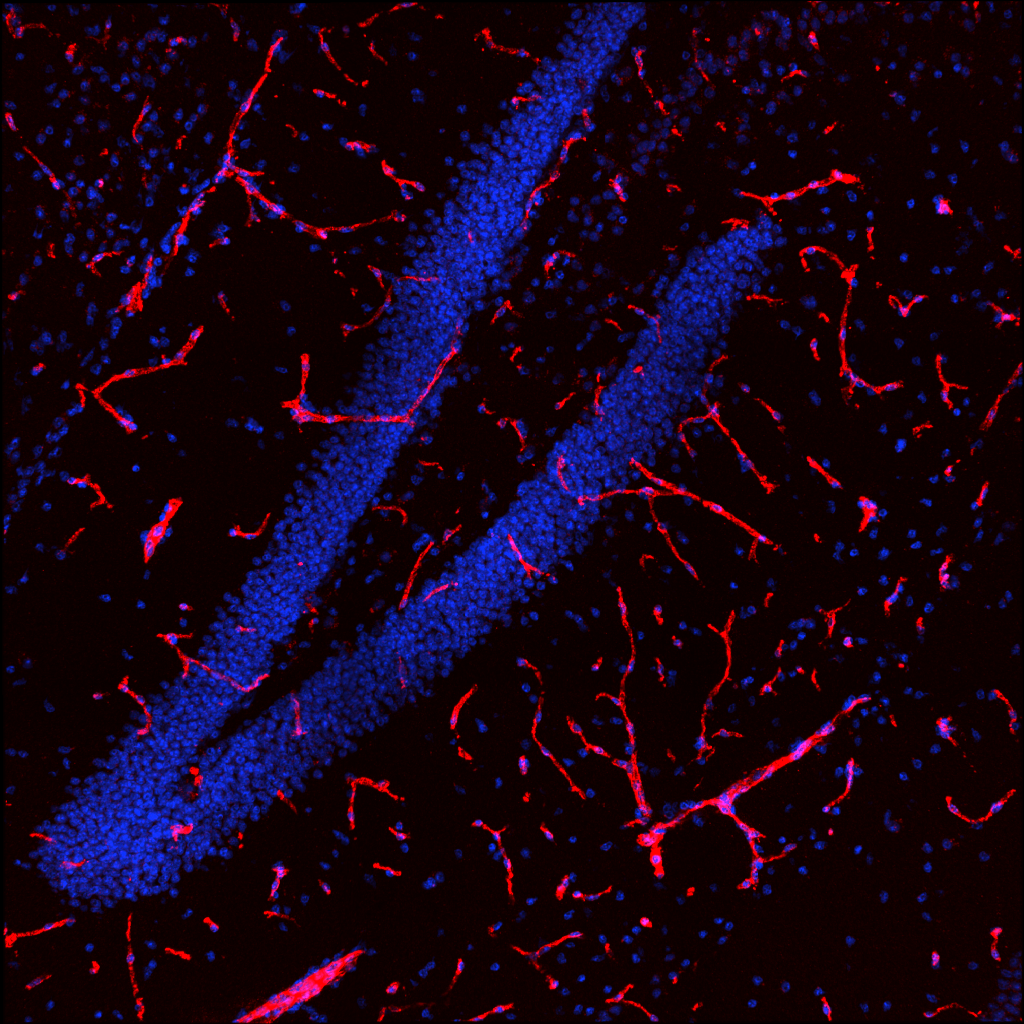

Supplement: Supplementary file 8 — Appendix Figure Source Data [file 44318_2024_78_MOESM8_ESM.zip › Appendix Figure/Appendix Figure S1/S1D/sGCCtr-2.tif]

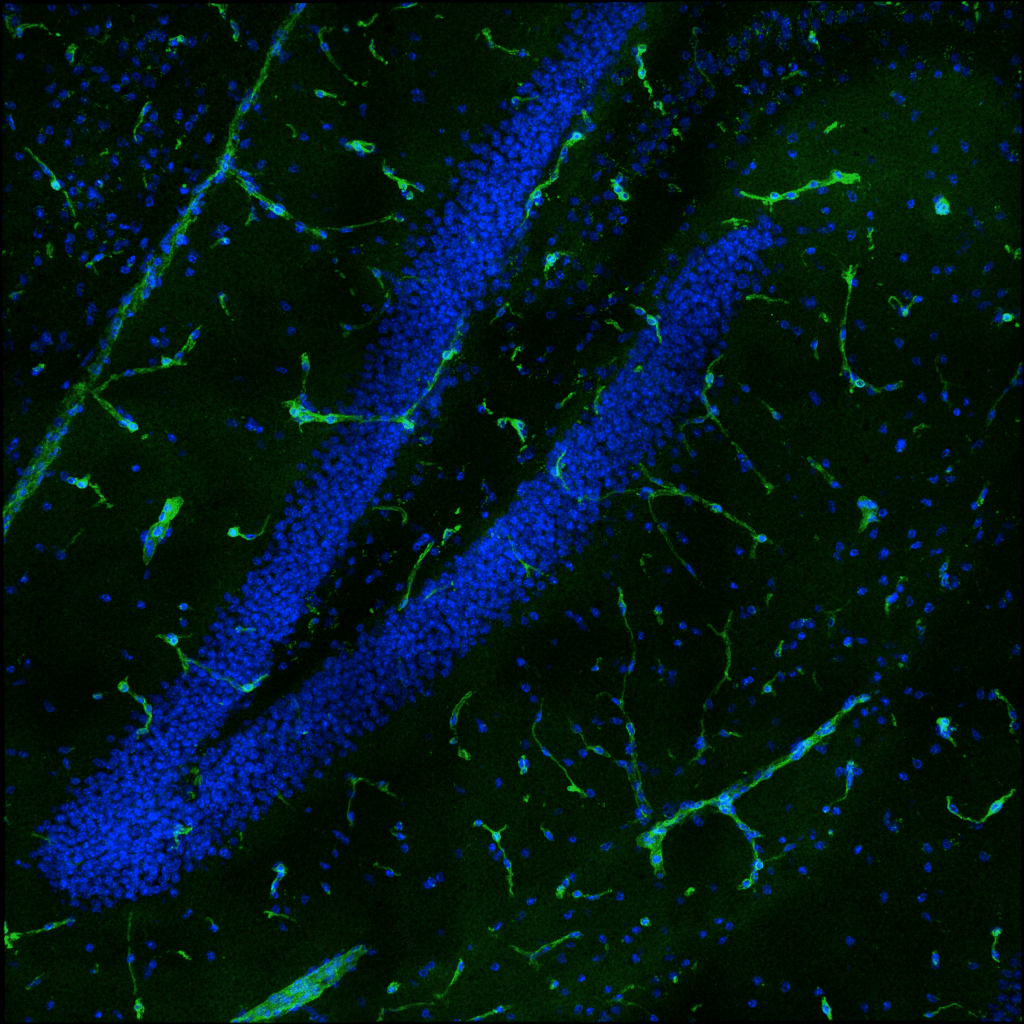

Supplement: Supplementary file 8 — Appendix Figure Source Data [file 44318_2024_78_MOESM8_ESM.zip › Appendix Figure/Appendix Figure S1/S1D/sGCCtr-1.tif]

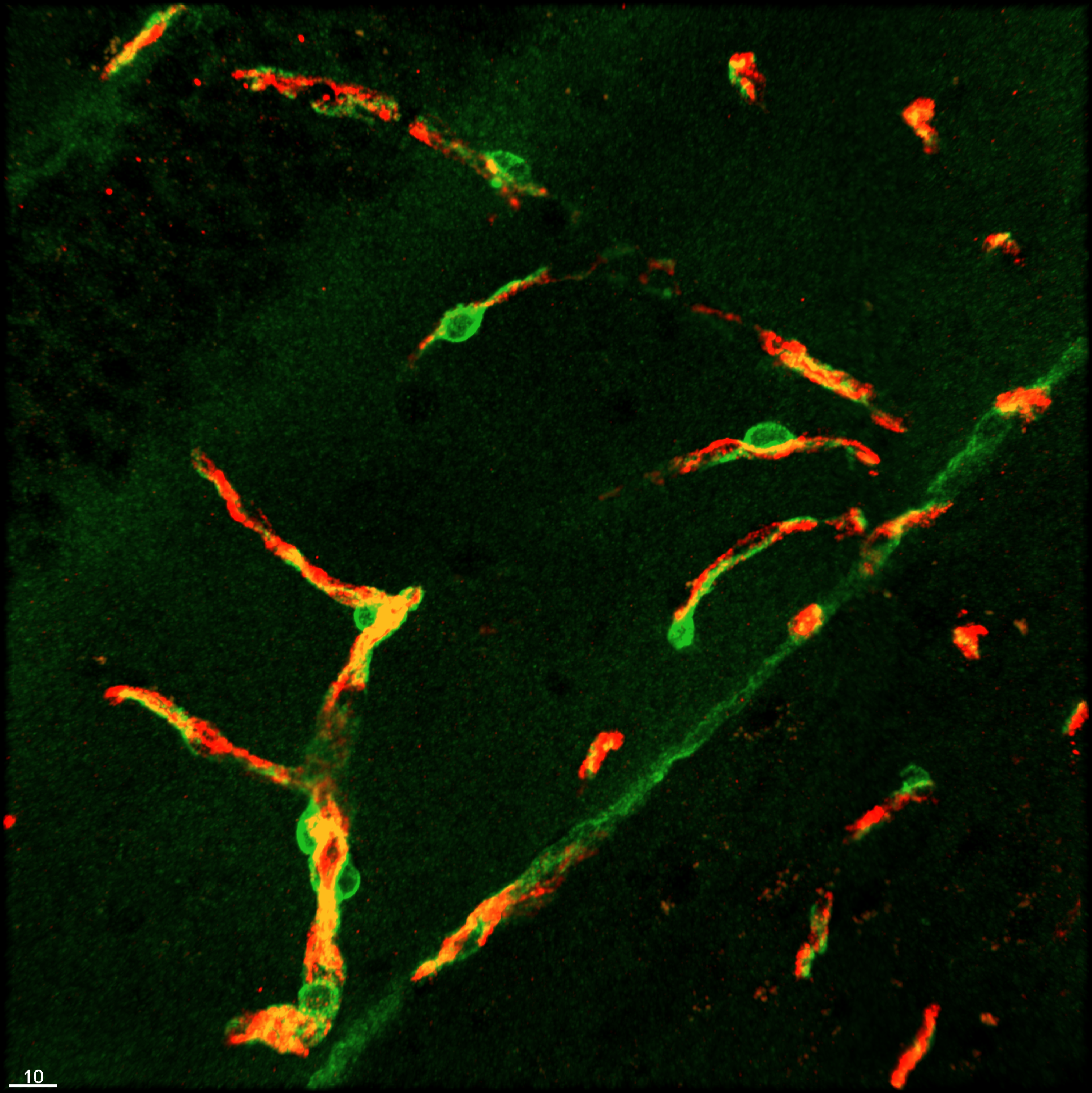

Supplement: Supplementary file 8 — Appendix Figure Source Data [file 44318_2024_78_MOESM8_ESM.zip › Appendix Figure/Appendix Figure S1/S1D/sGC╬öpc-4.tif]

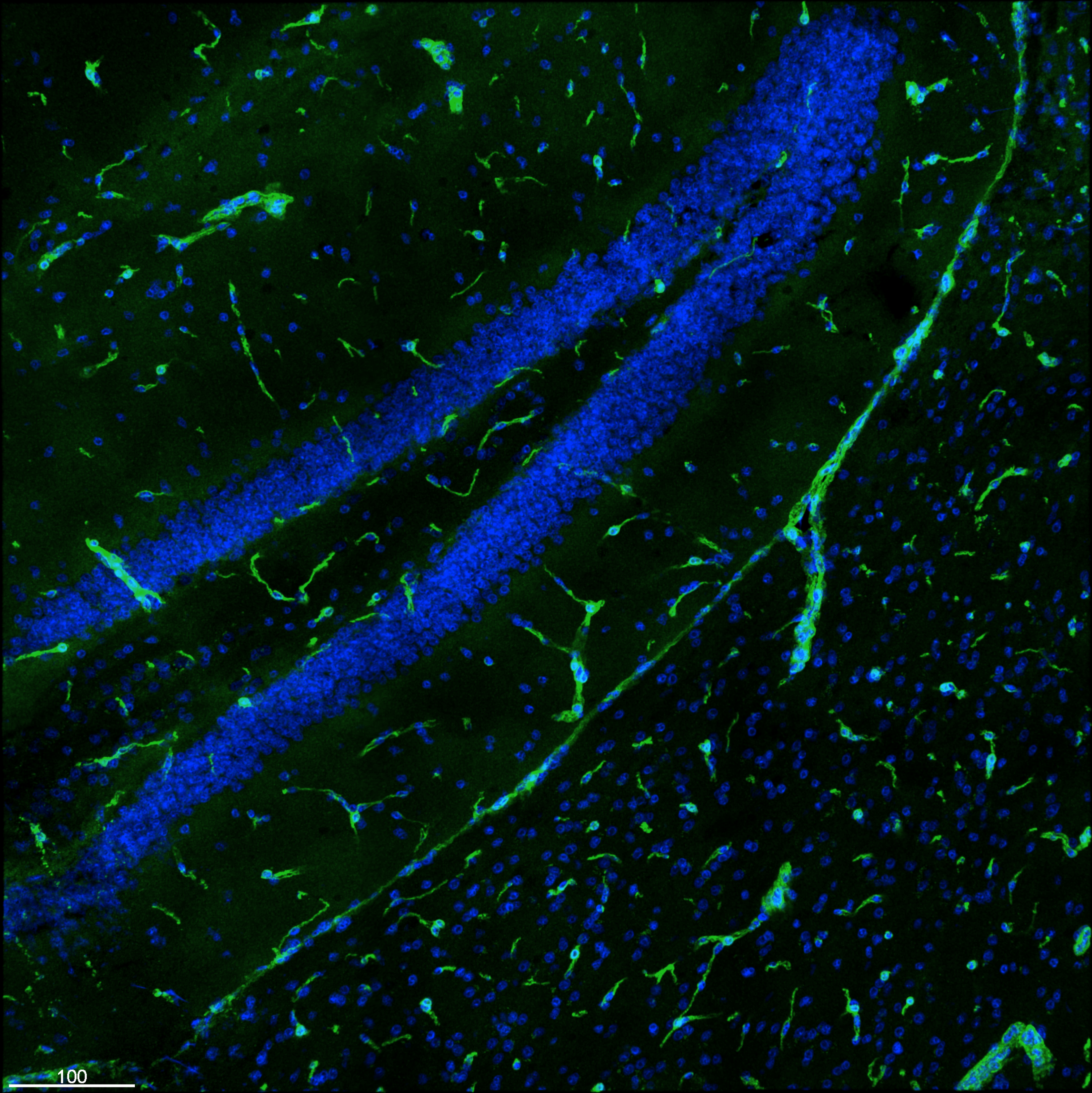

Supplement: Supplementary file 8 — Appendix Figure Source Data [file 44318_2024_78_MOESM8_ESM.zip › Appendix Figure/Appendix Figure S1/S1D/sGC╬öpc-1.tif]

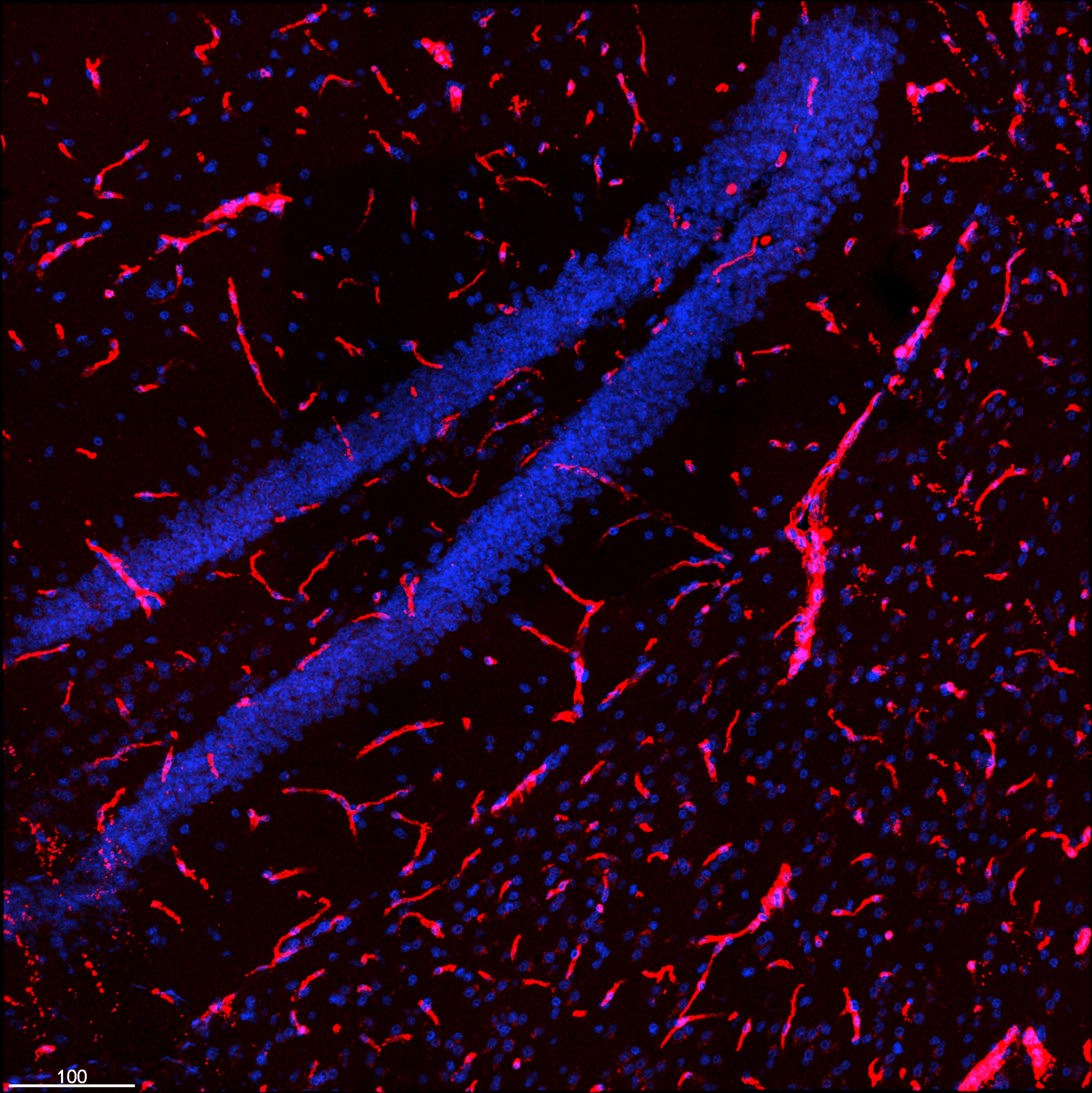

Supplement: Supplementary file 8 — Appendix Figure Source Data [file 44318_2024_78_MOESM8_ESM.zip › Appendix Figure/Appendix Figure S1/S1D/sGC╬öpc-2.tif]

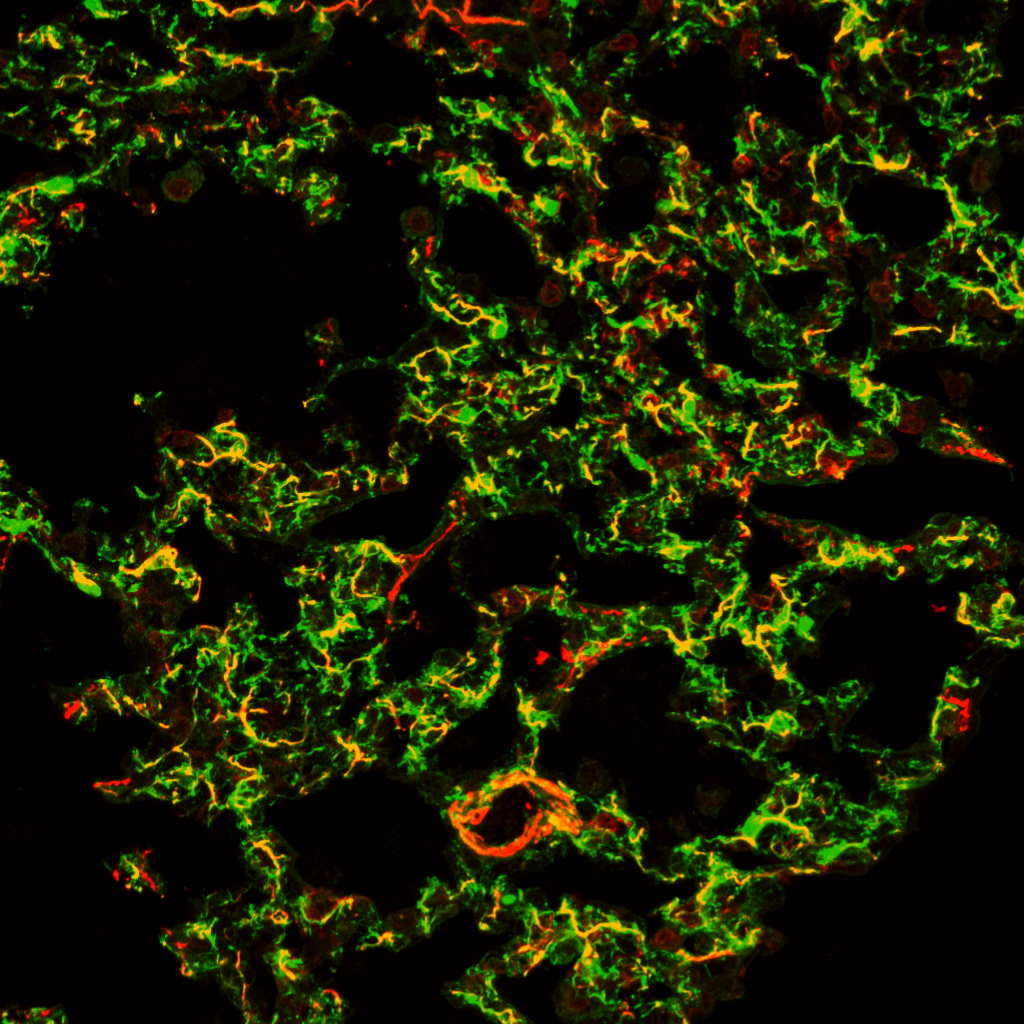

Supplement: Supplementary file 8 — Appendix Figure Source Data [file 44318_2024_78_MOESM8_ESM.zip › Appendix Figure/Appendix Figure S1/S1C/sGCCtr-4.tif]

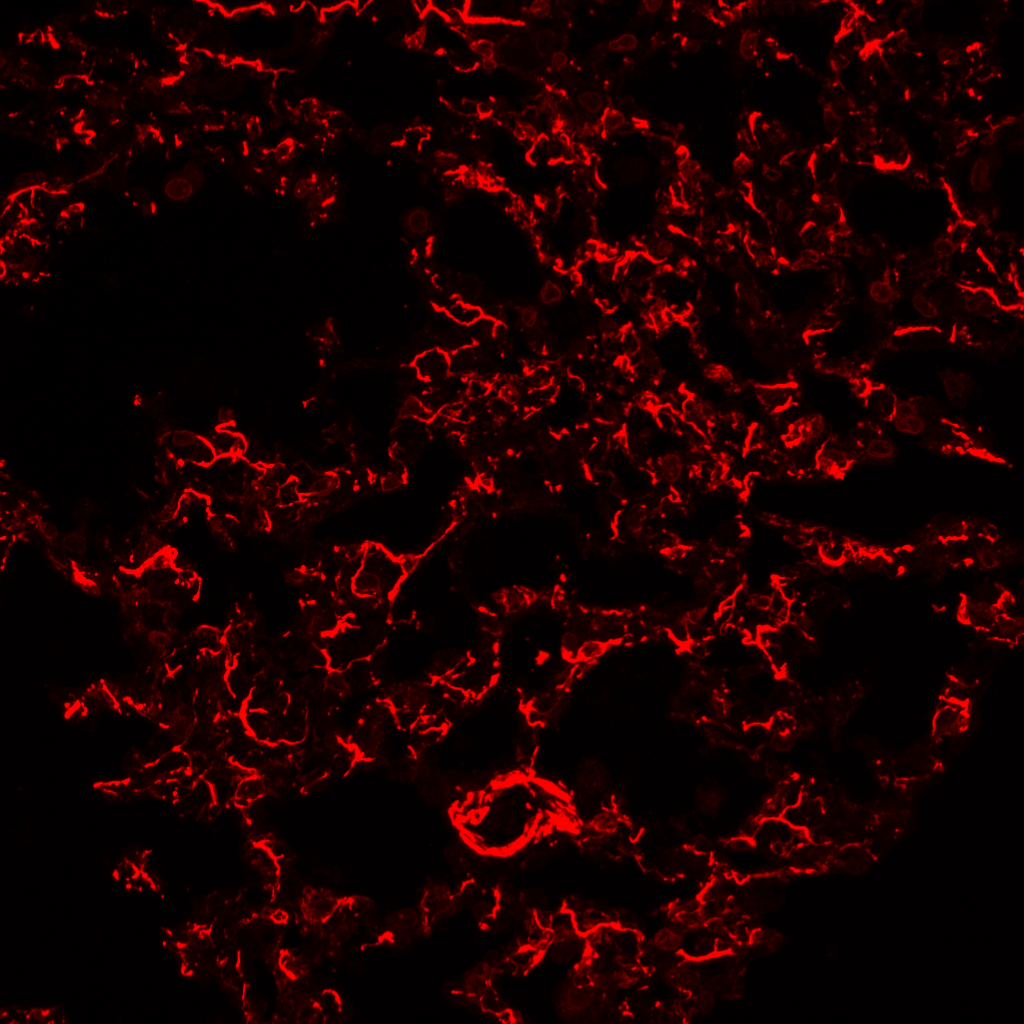

Supplement: Supplementary file 8 — Appendix Figure Source Data [file 44318_2024_78_MOESM8_ESM.zip › Appendix Figure/Appendix Figure S1/S1C/sGCCtr-3.tif]

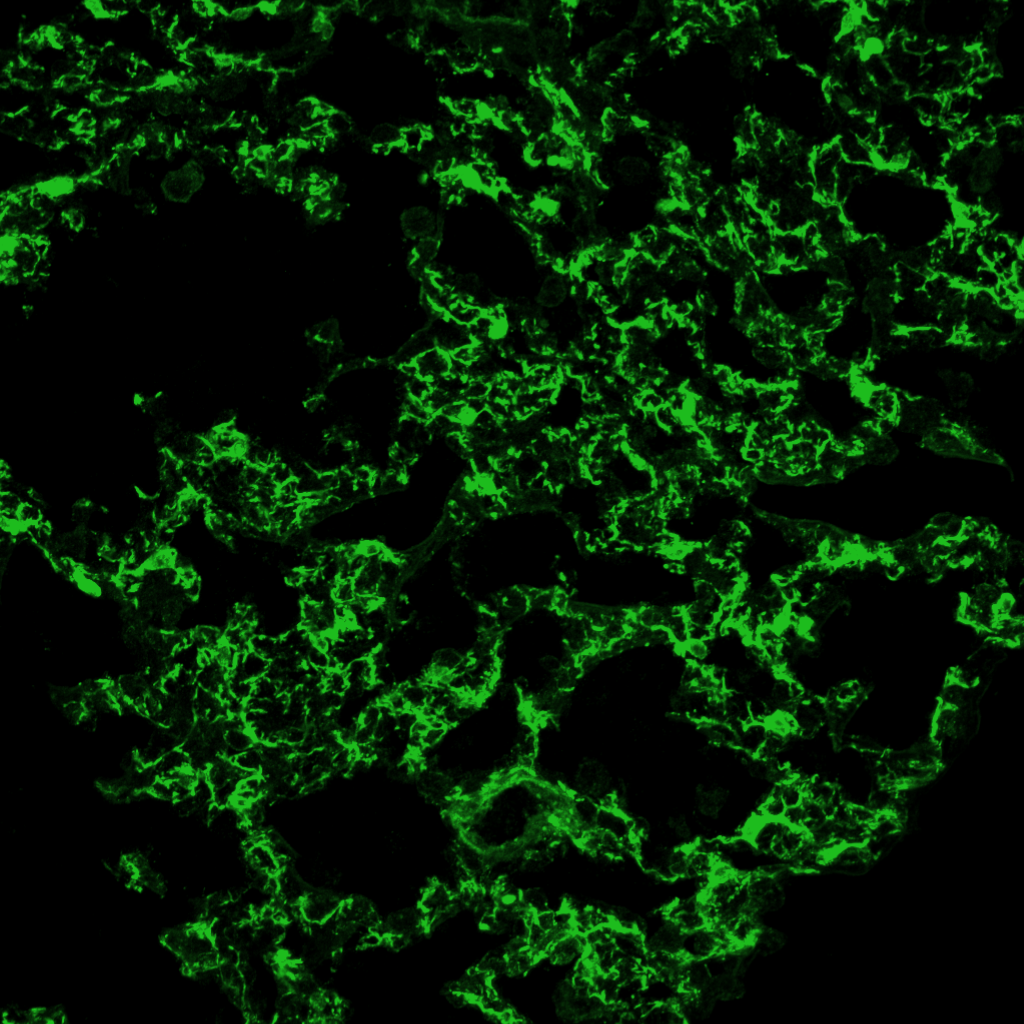

Supplement: Supplementary file 8 — Appendix Figure Source Data [file 44318_2024_78_MOESM8_ESM.zip › Appendix Figure/Appendix Figure S1/S1C/sGCCtr-2.tif]

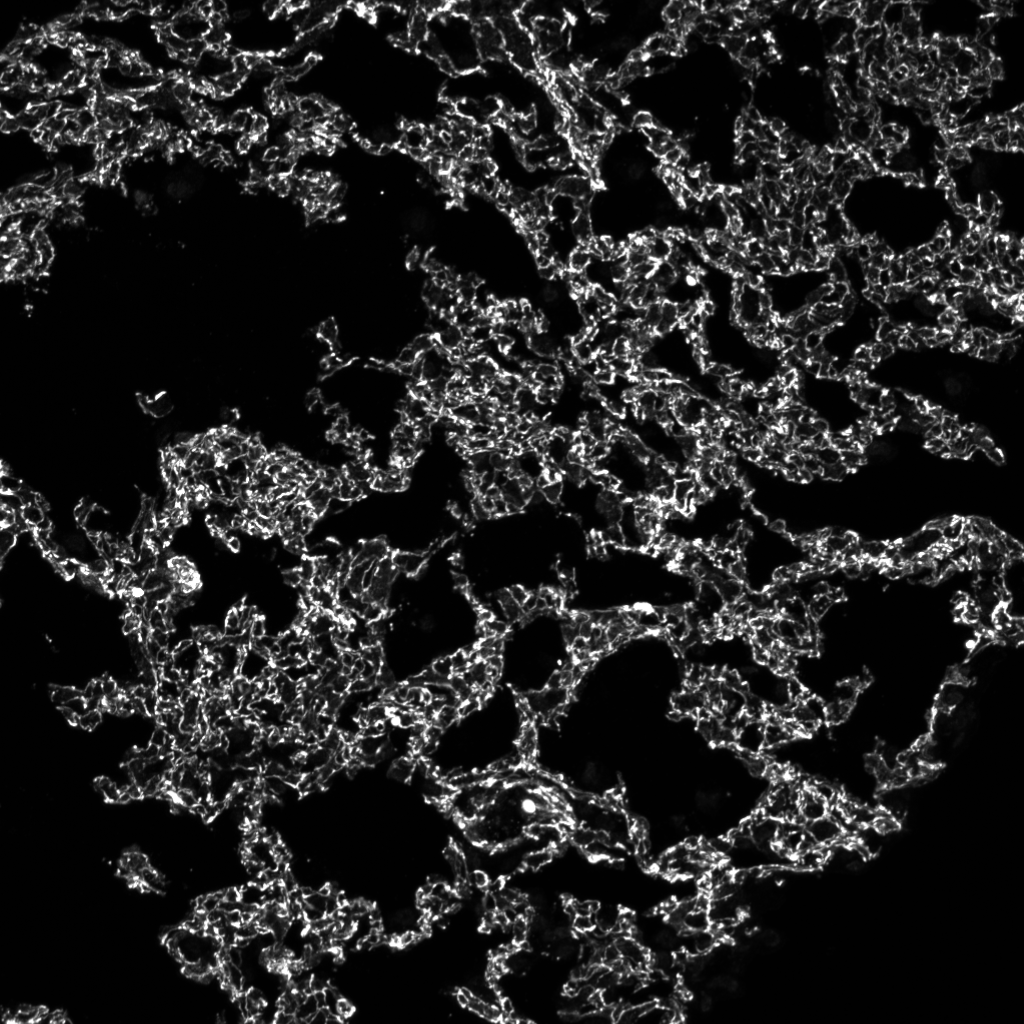

Supplement: Supplementary file 8 — Appendix Figure Source Data [file 44318_2024_78_MOESM8_ESM.zip › Appendix Figure/Appendix Figure S1/S1C/sGCCtr-1.tif]

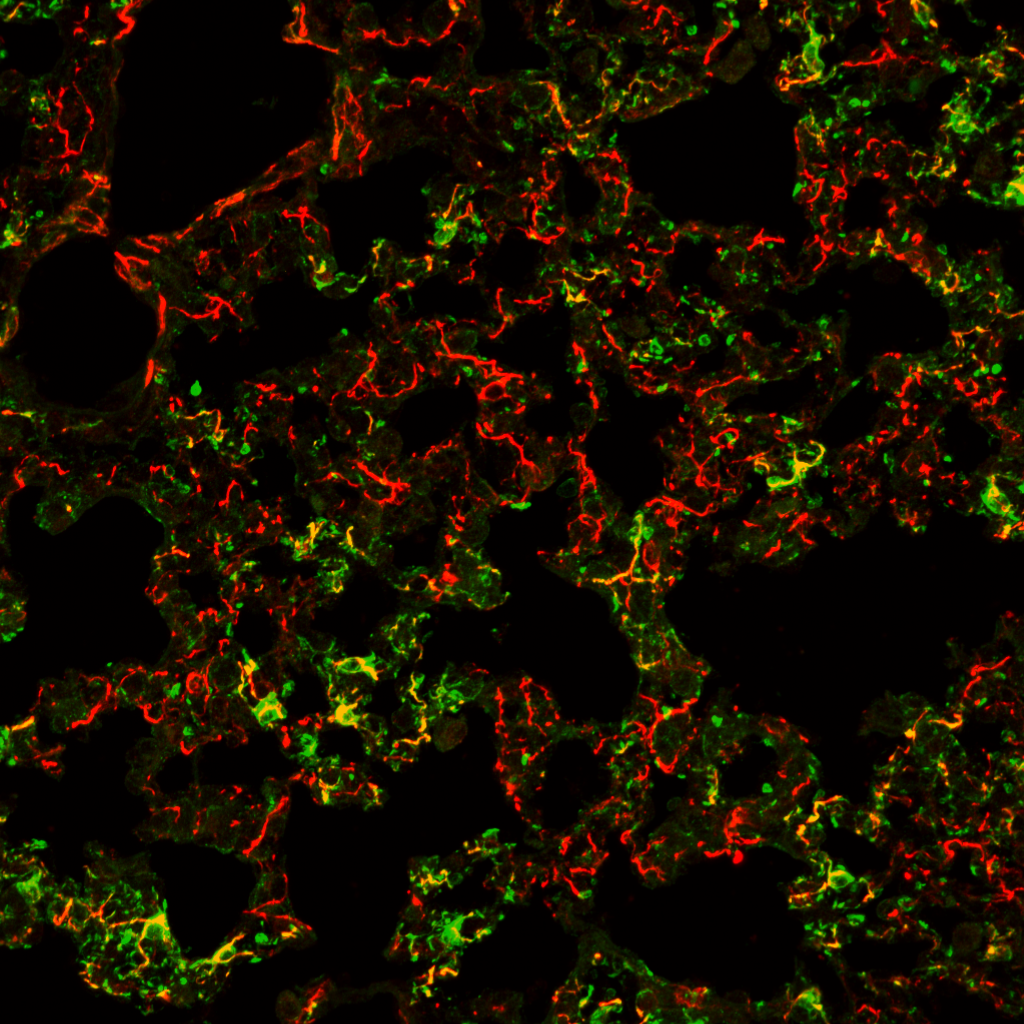

Supplement: Supplementary file 8 — Appendix Figure Source Data [file 44318_2024_78_MOESM8_ESM.zip › Appendix Figure/Appendix Figure S1/S1C/sGC╬öpc-4.tif]

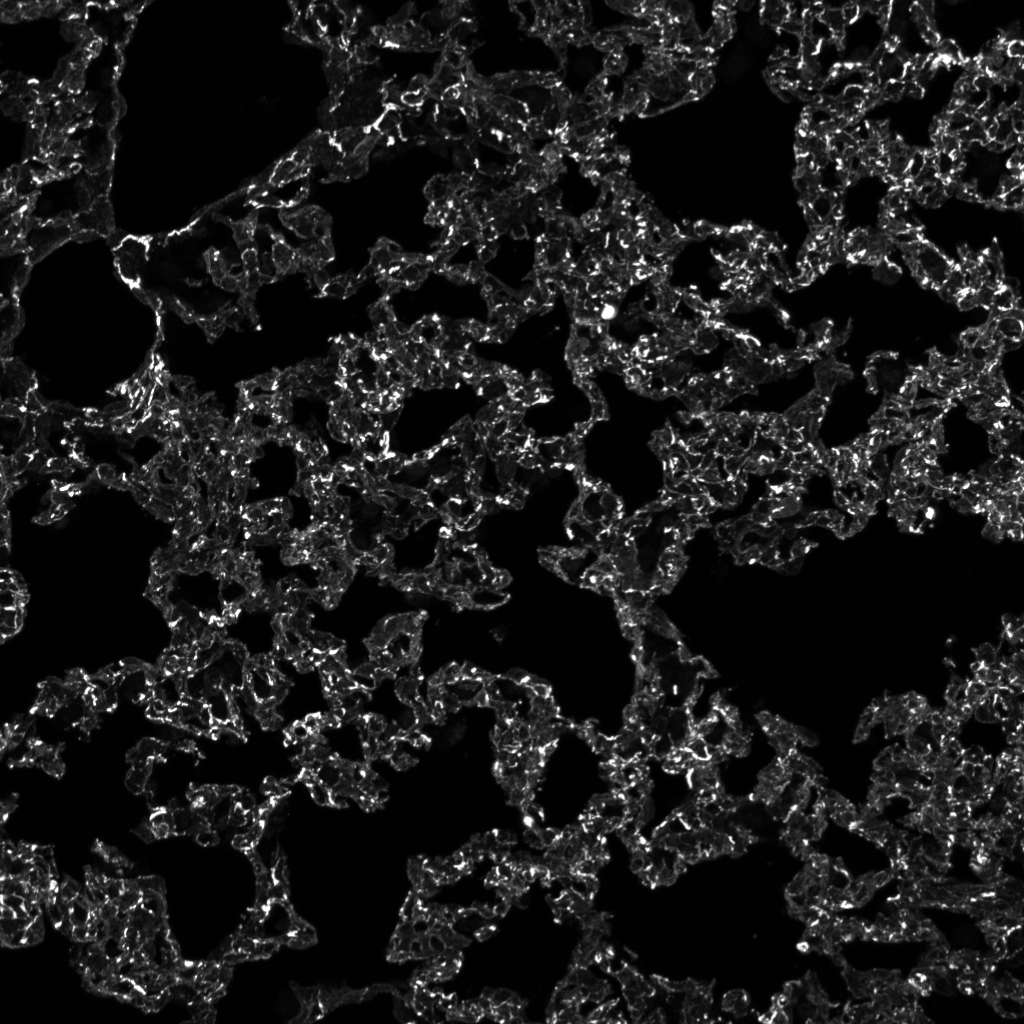

Supplement: Supplementary file 8 — Appendix Figure Source Data [file 44318_2024_78_MOESM8_ESM.zip › Appendix Figure/Appendix Figure S1/S1C/sGC╬öpc-1.tif]

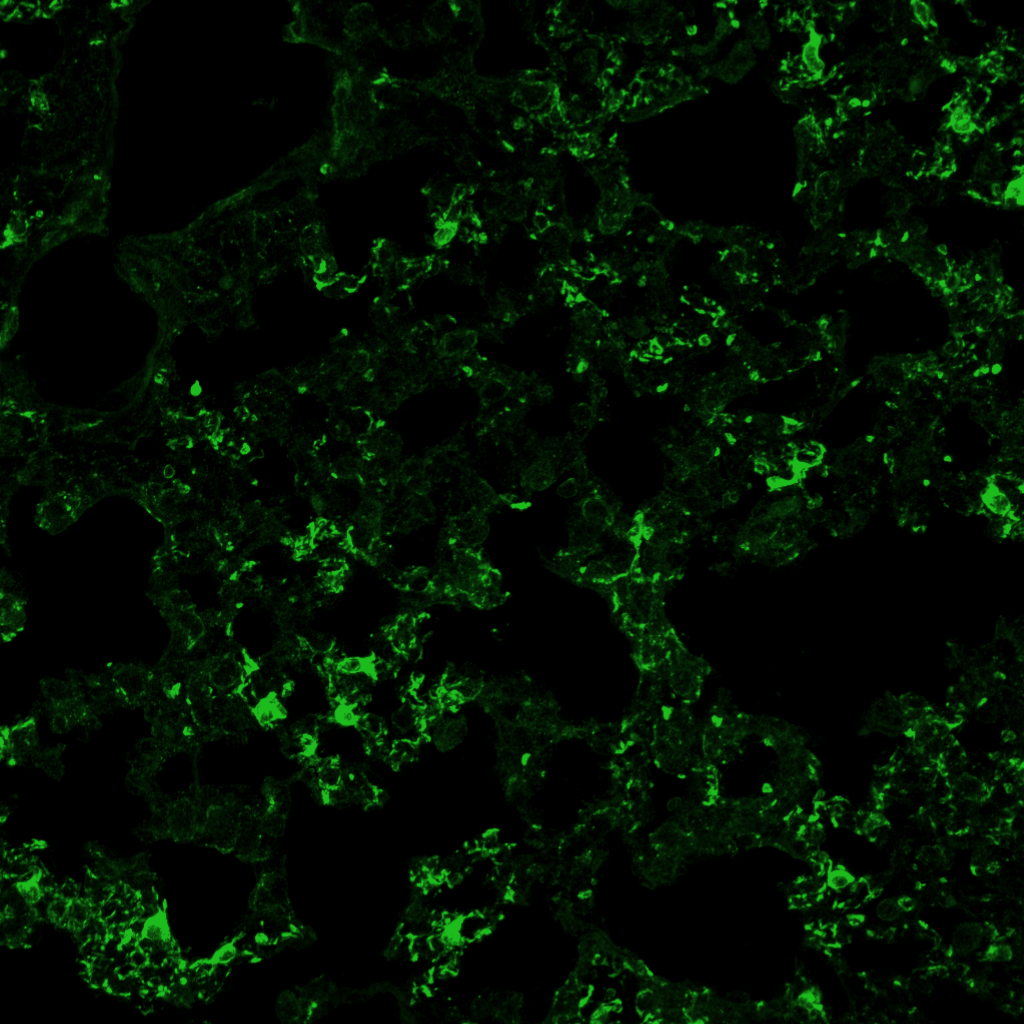

Supplement: Supplementary file 8 — Appendix Figure Source Data [file 44318_2024_78_MOESM8_ESM.zip › Appendix Figure/Appendix Figure S1/S1C/sGC╬öpc-2.tif]

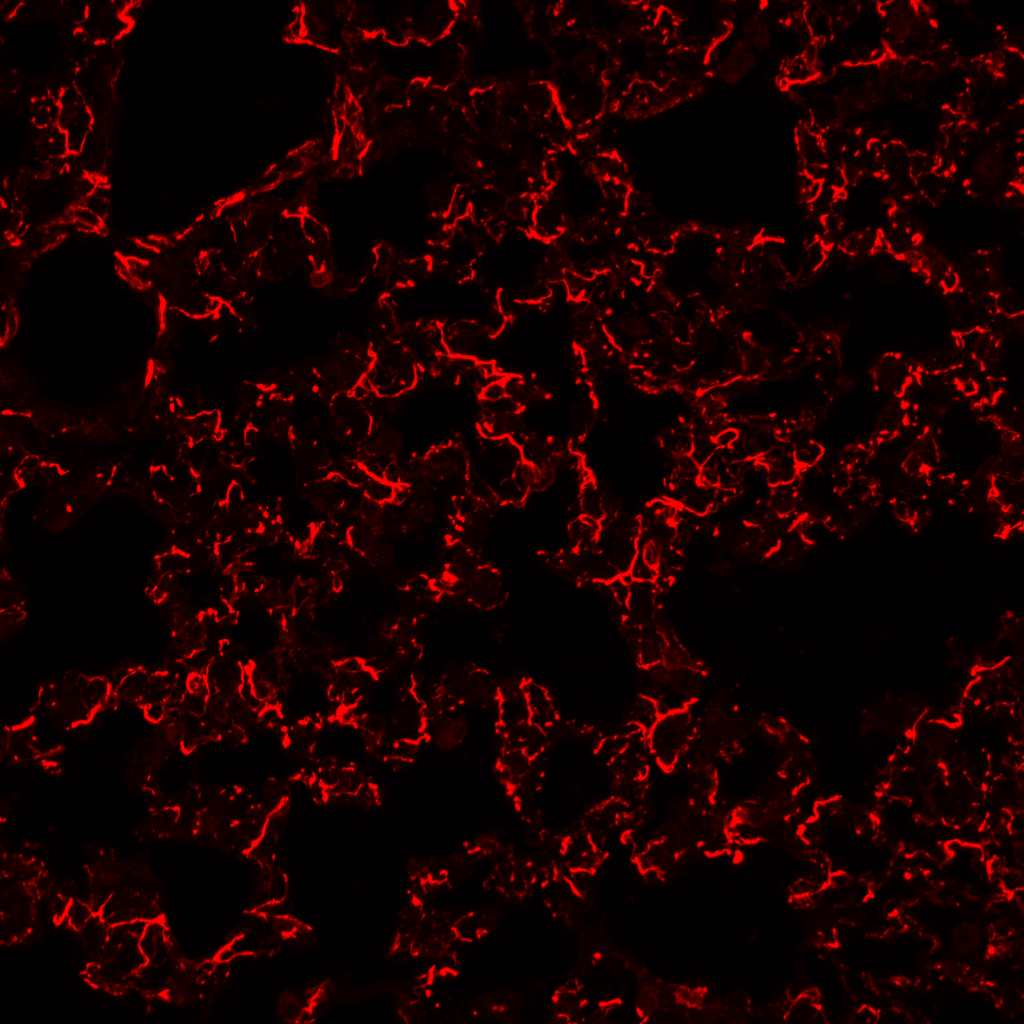

Supplement: Supplementary file 8 — Appendix Figure Source Data [file 44318_2024_78_MOESM8_ESM.zip › Appendix Figure/Appendix Figure S1/S1C/sGC╬öpc-3.tif]

Original blots of Appendix Figure S1 B

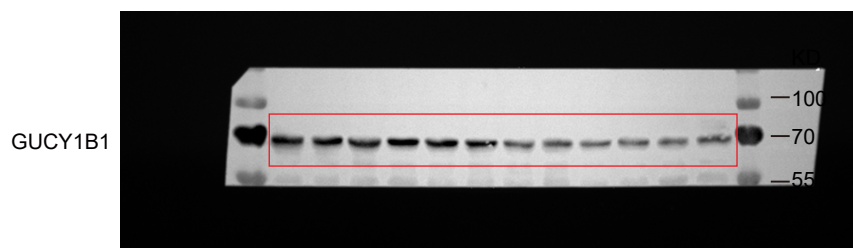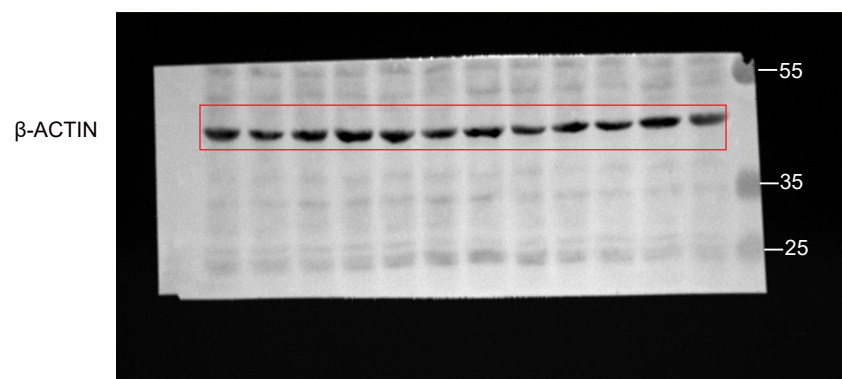

Supplement: Supplementary file 8 — Appendix Figure Source Data [file 44318_2024_78_MOESM8_ESM.zip › Appendix Figure/Appendix Figure S1/S1B/S1 B.pdf]

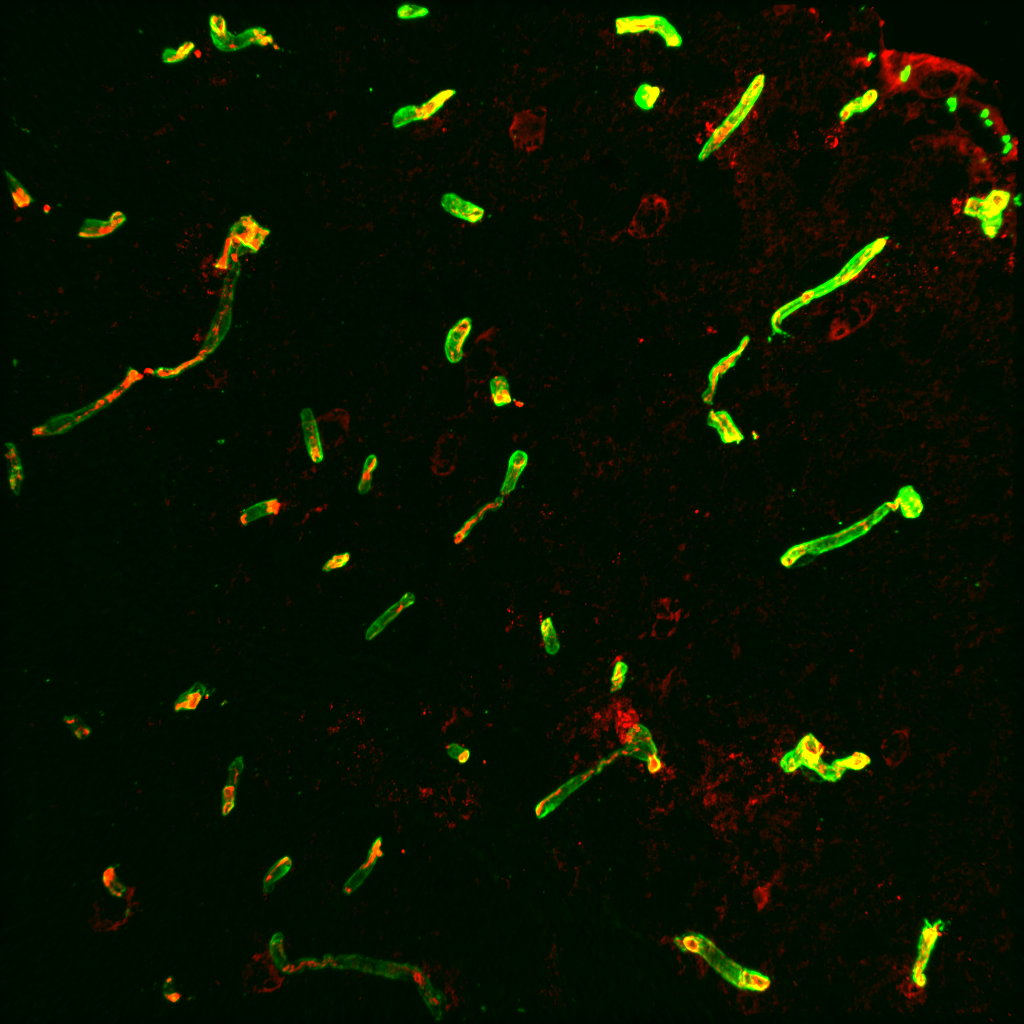

Supplement: Supplementary file 8 — Appendix Figure Source Data [file 44318_2024_78_MOESM8_ESM.zip › Appendix Figure/Appendix Figure S1/S1E/sGCCtr-3.tif]

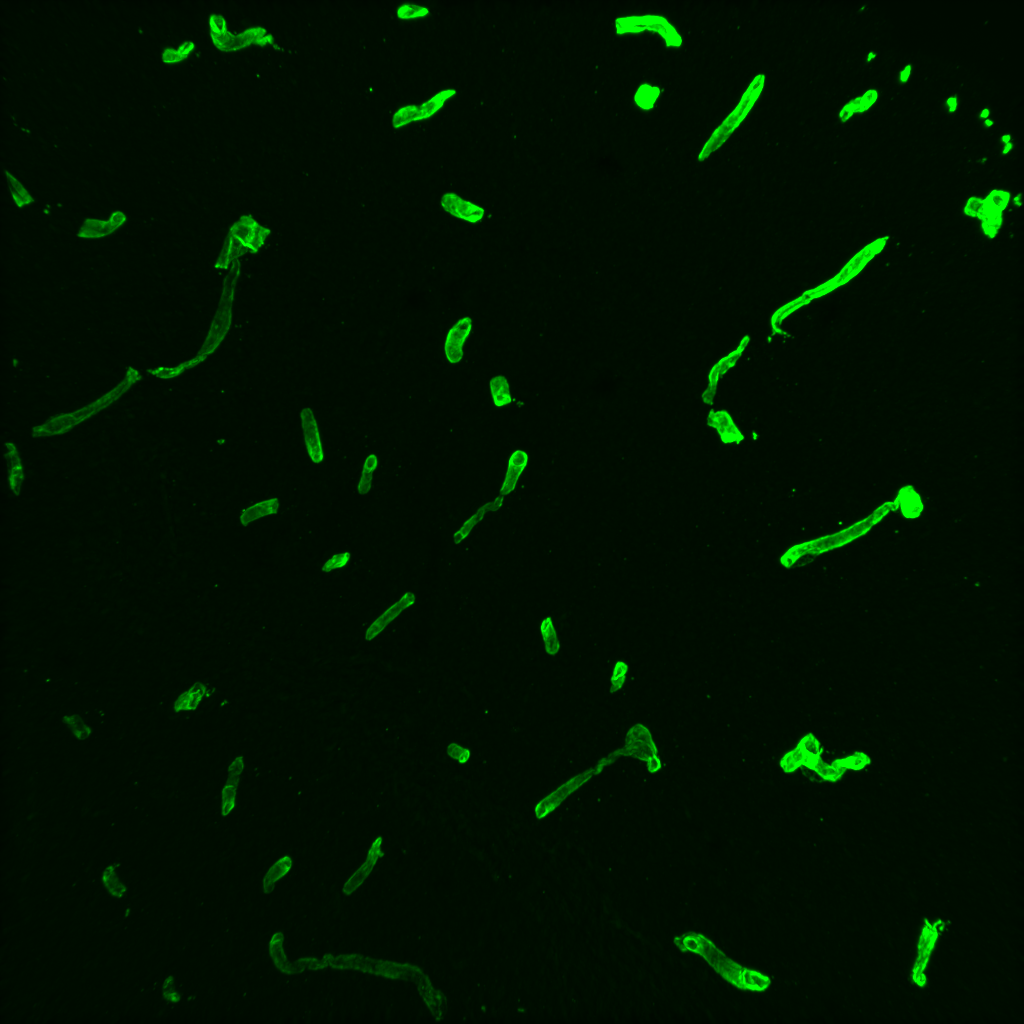

Supplement: Supplementary file 8 — Appendix Figure Source Data [file 44318_2024_78_MOESM8_ESM.zip › Appendix Figure/Appendix Figure S1/S1E/sGCCtr-2.tif]

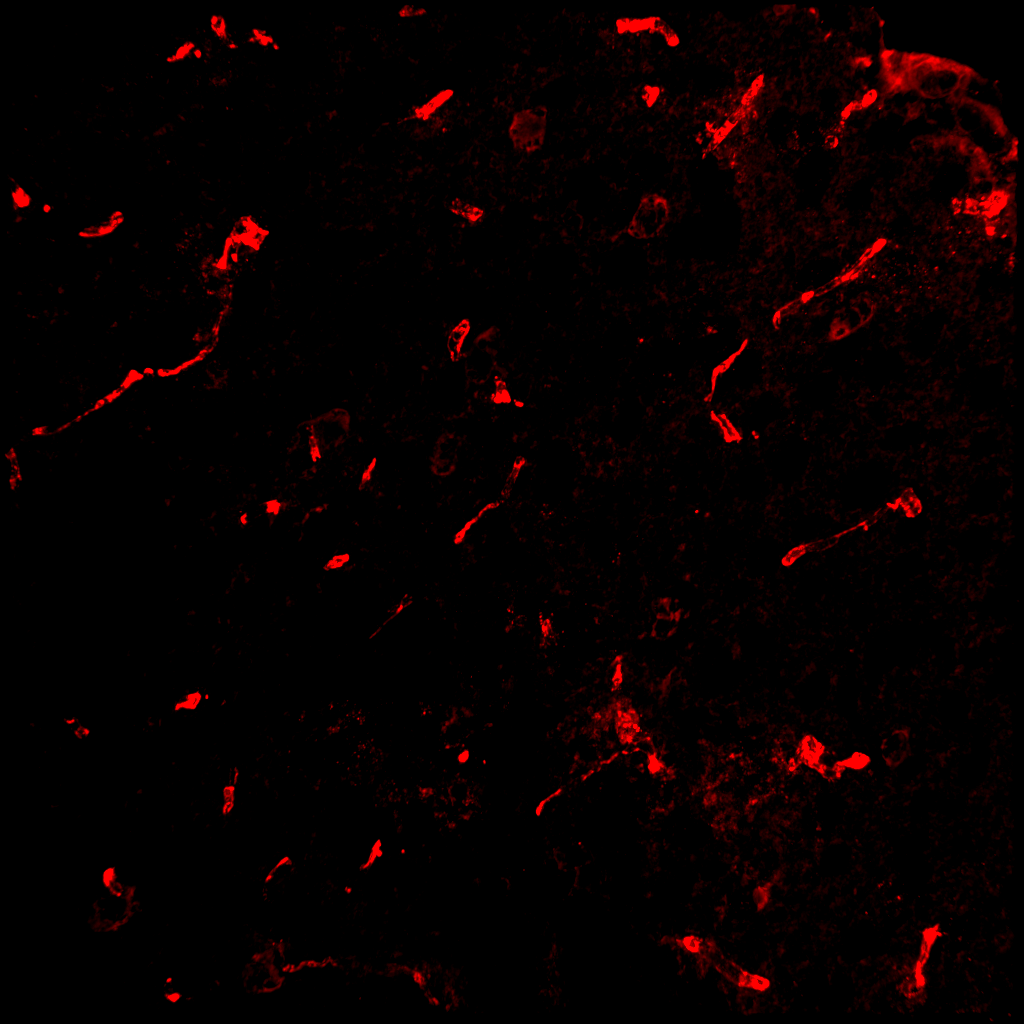

Supplement: Supplementary file 8 — Appendix Figure Source Data [file 44318_2024_78_MOESM8_ESM.zip › Appendix Figure/Appendix Figure S1/S1E/sGCCtr-1.tif]

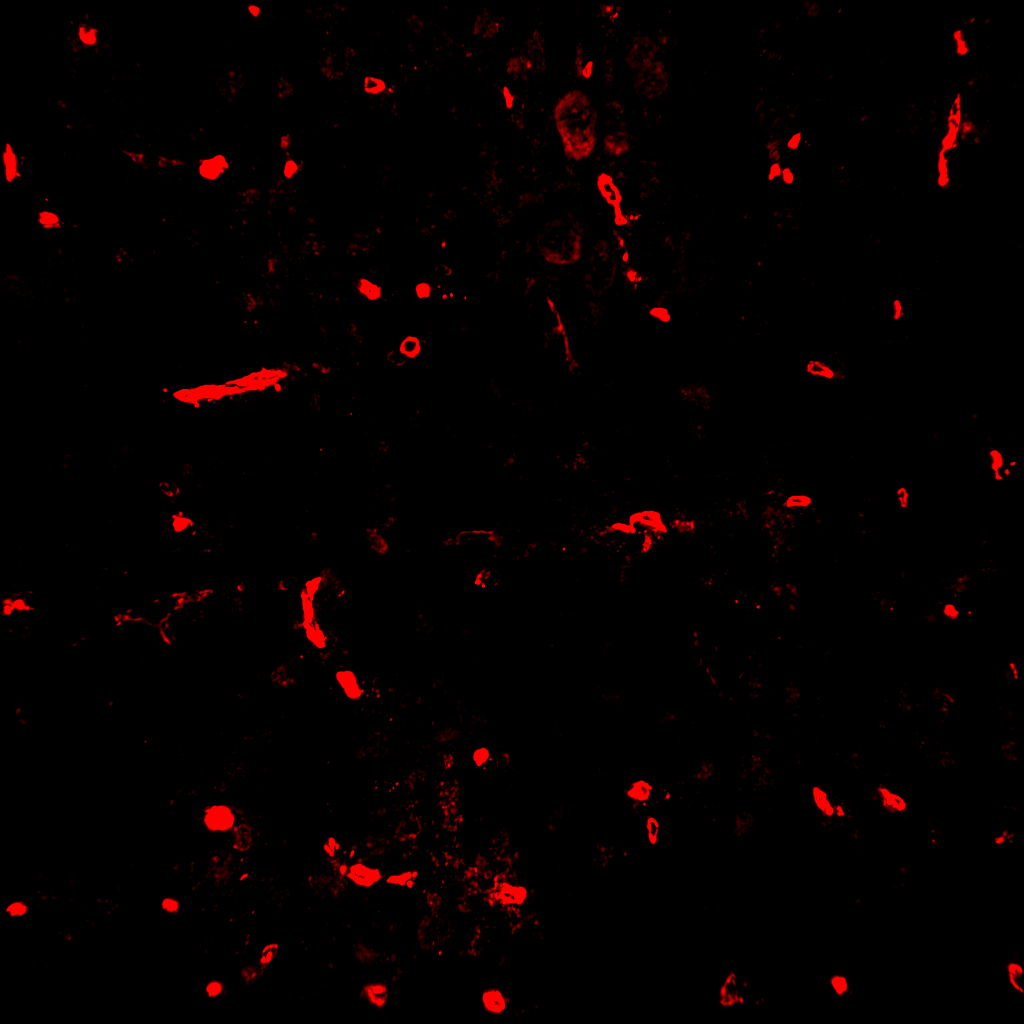

Supplement: Supplementary file 8 — Appendix Figure Source Data [file 44318_2024_78_MOESM8_ESM.zip › Appendix Figure/Appendix Figure S1/S1E/sGC╬öpc-1.tif]

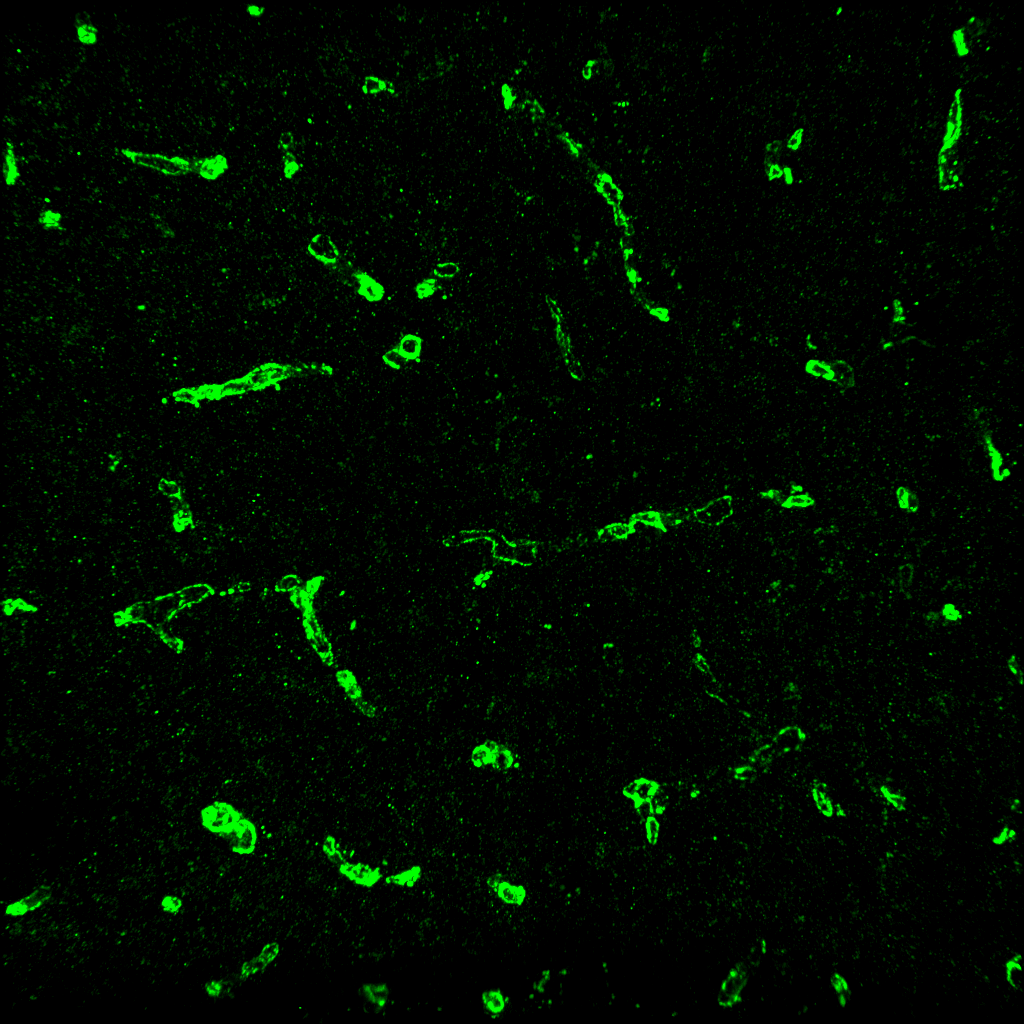

Supplement: Supplementary file 8 — Appendix Figure Source Data [file 44318_2024_78_MOESM8_ESM.zip › Appendix Figure/Appendix Figure S1/S1E/sGC╬öpc-2.tif]

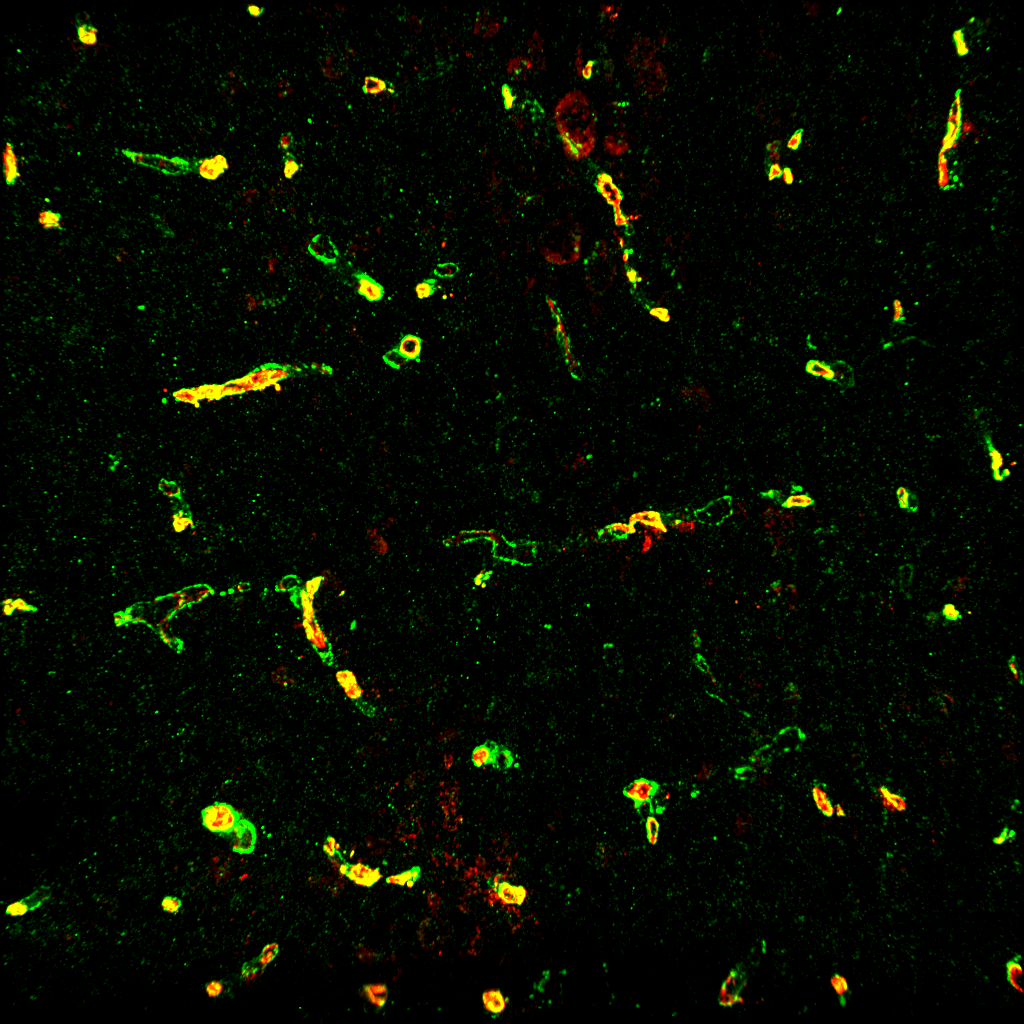

Supplement: Supplementary file 8 — Appendix Figure Source Data [file 44318_2024_78_MOESM8_ESM.zip › Appendix Figure/Appendix Figure S1/S1E/sGC╬öpc-3.tif]

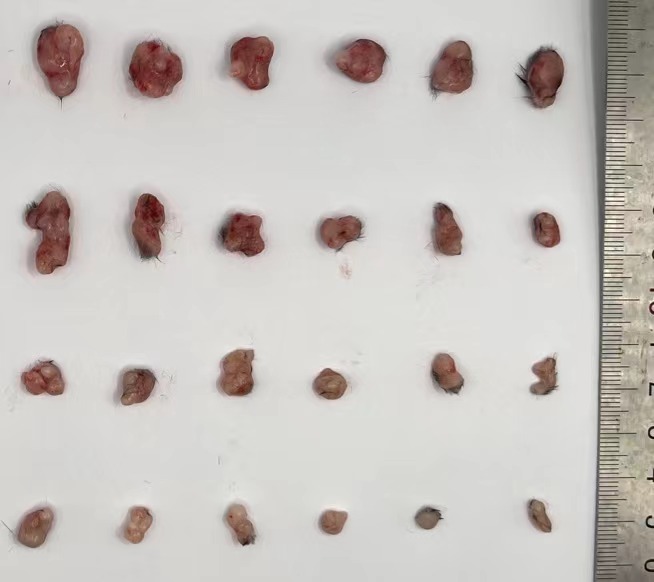

Supplement: Supplementary file 8 — Appendix Figure Source Data [file 44318_2024_78_MOESM8_ESM.zip › Appendix Figure/Appendix Figure S10/S10B/1.jpg]

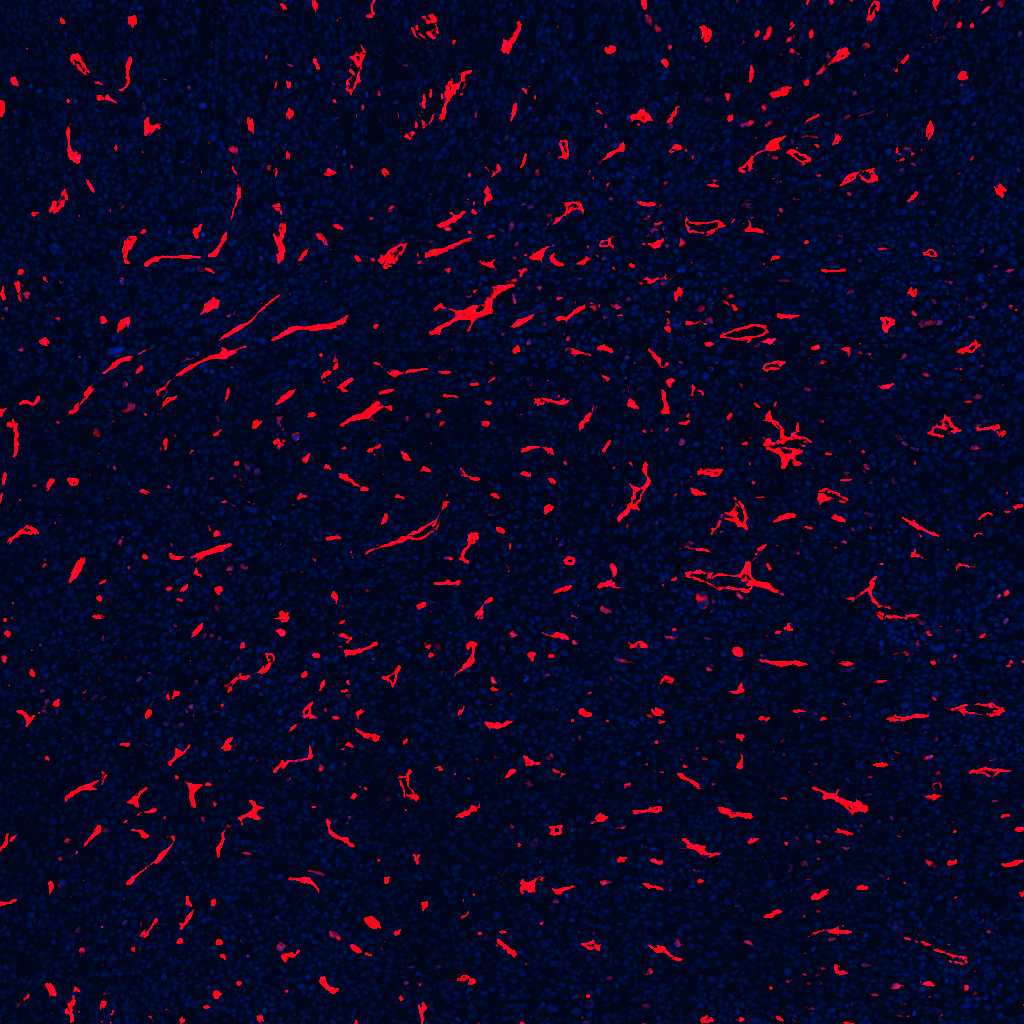

Supplement: Supplementary file 8 — Appendix Figure Source Data [file 44318_2024_78_MOESM8_ESM.zip › Appendix Figure/Appendix Figure S3/S3C/sGCCtr-3.tif]

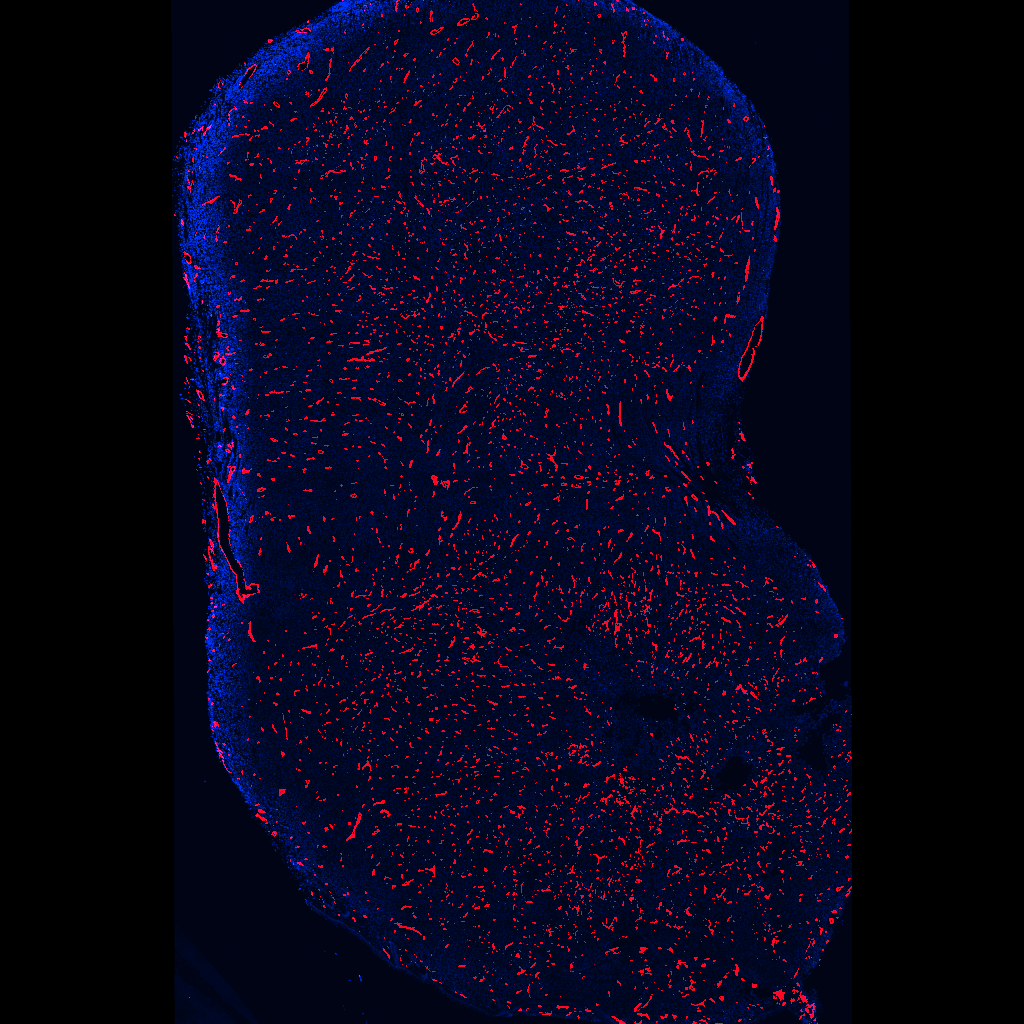

Supplement: Supplementary file 8 — Appendix Figure Source Data [file 44318_2024_78_MOESM8_ESM.zip › Appendix Figure/Appendix Figure S3/S3C/sGCCtr-2.tif]

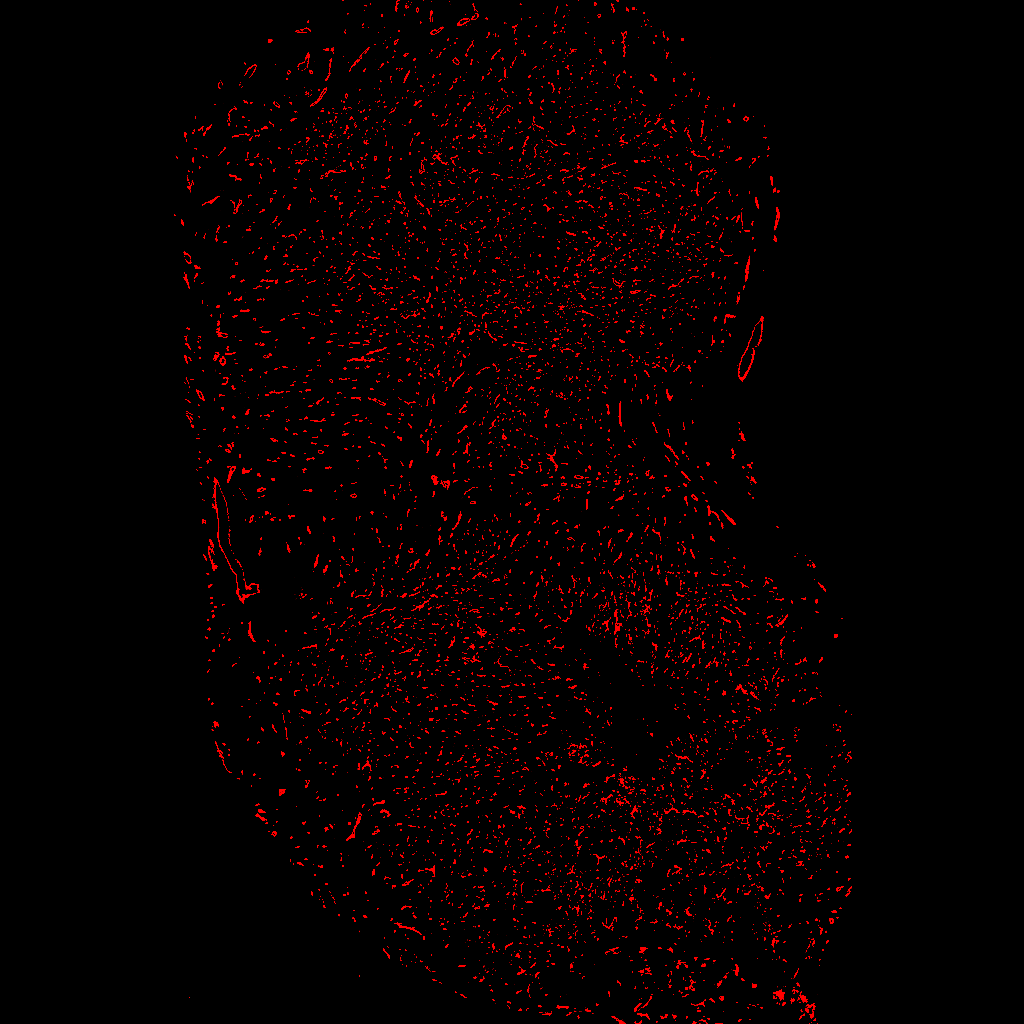

Supplement: Supplementary file 8 — Appendix Figure Source Data [file 44318_2024_78_MOESM8_ESM.zip › Appendix Figure/Appendix Figure S3/S3C/sGCCtr-1.tif]

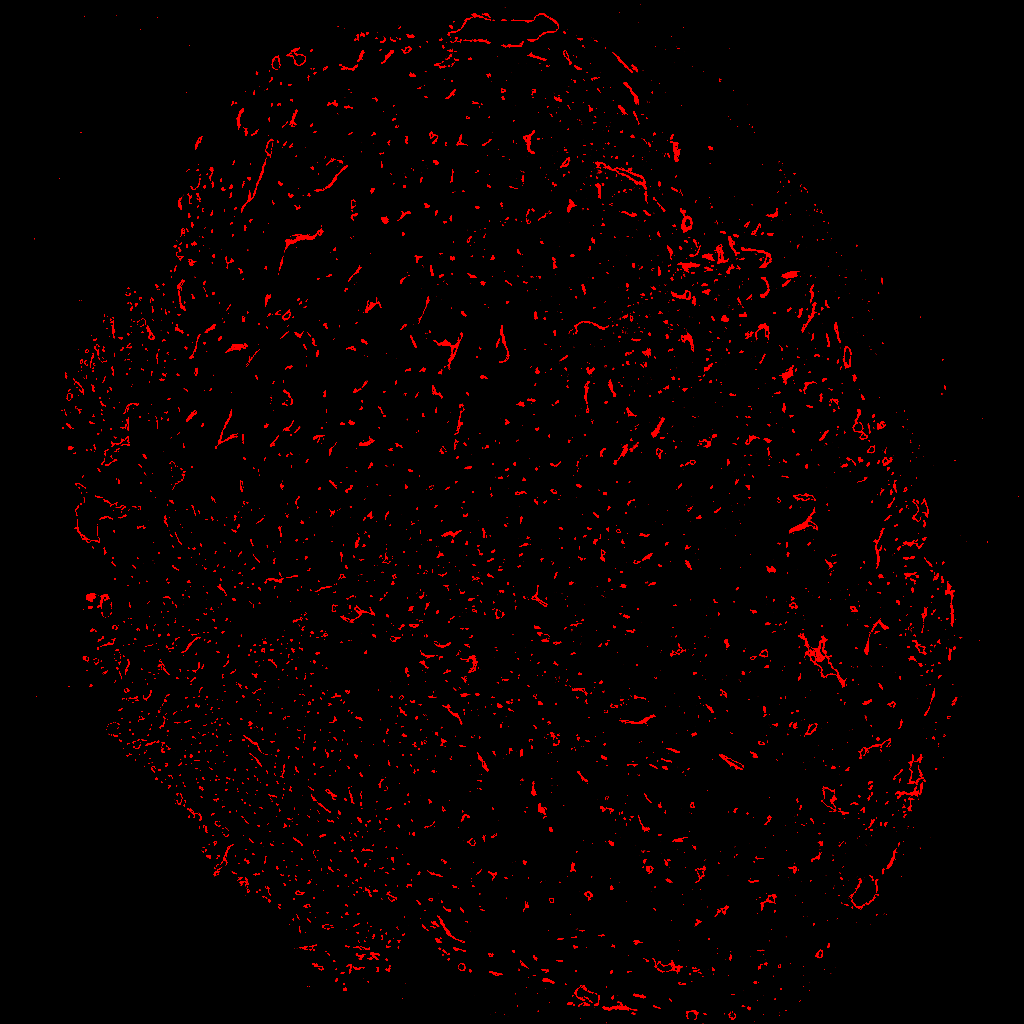

Supplement: Supplementary file 8 — Appendix Figure Source Data [file 44318_2024_78_MOESM8_ESM.zip › Appendix Figure/Appendix Figure S3/S3C/sGC╬öpc-1.tif]

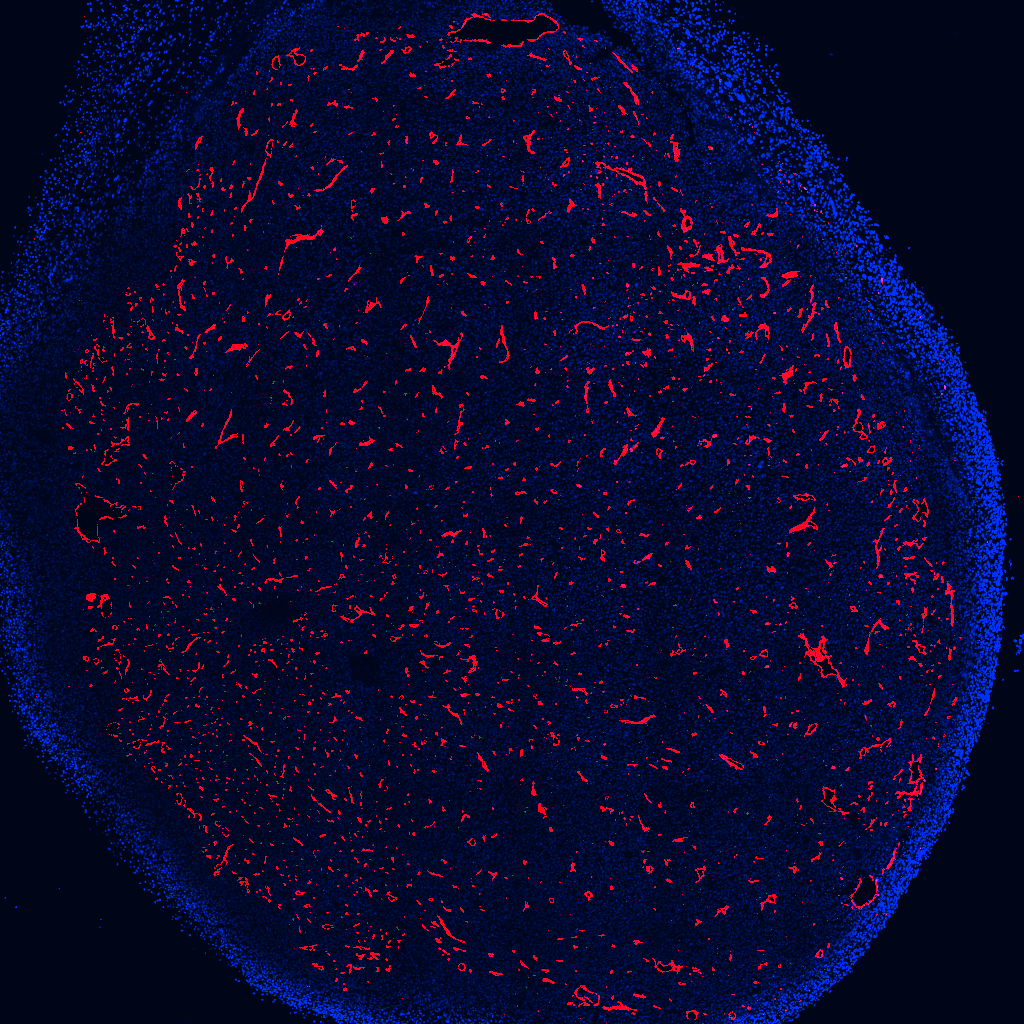

Supplement: Supplementary file 8 — Appendix Figure Source Data [file 44318_2024_78_MOESM8_ESM.zip › Appendix Figure/Appendix Figure S3/S3C/sGC╬öpc-2.tif]

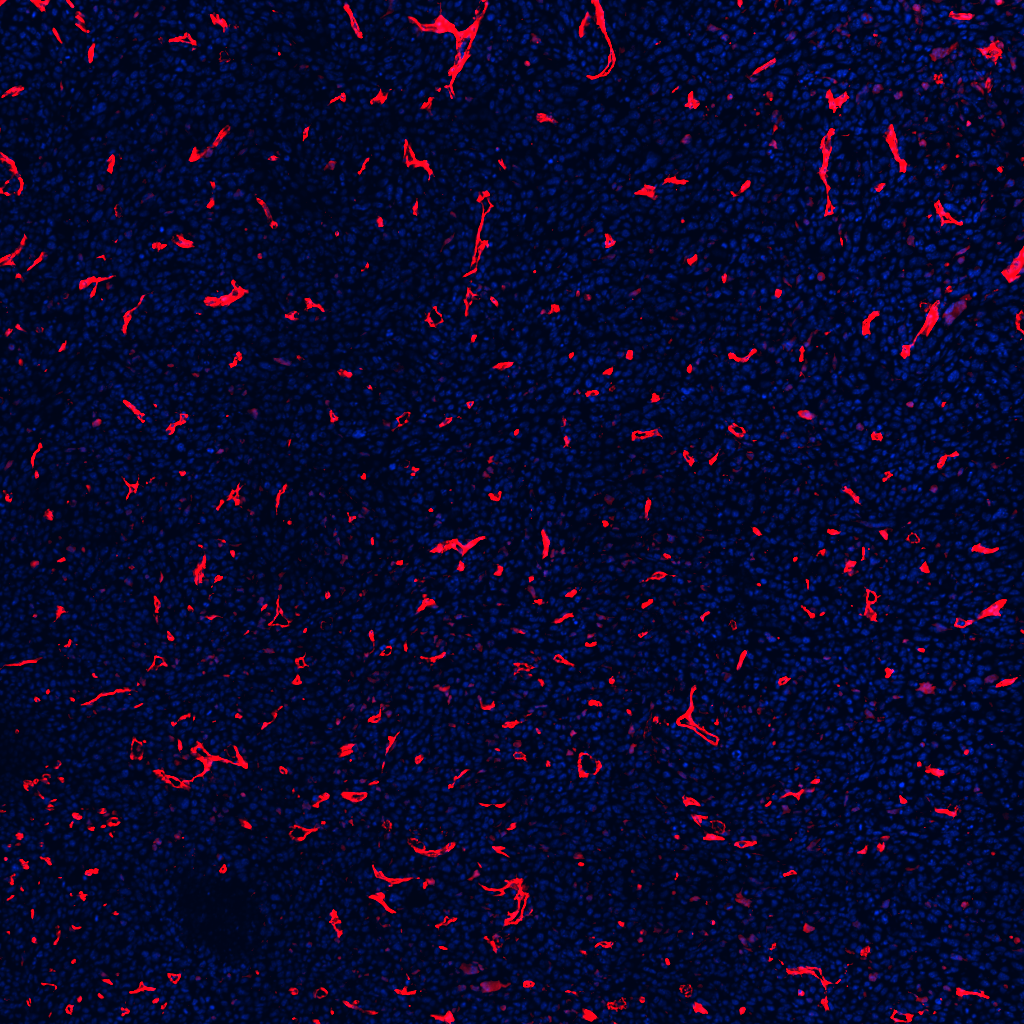

Supplement: Supplementary file 8 — Appendix Figure Source Data [file 44318_2024_78_MOESM8_ESM.zip › Appendix Figure/Appendix Figure S3/S3C/sGC╬öpc-3.tif]

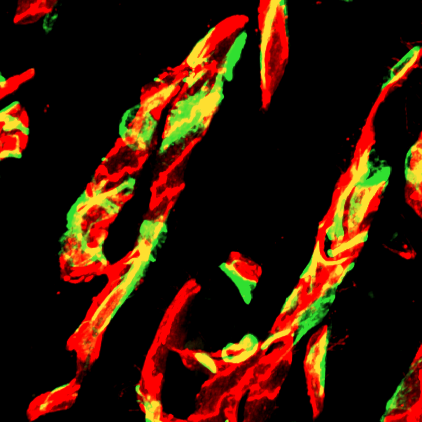

Supplement: Supplementary file 8 — Appendix Figure Source Data [file 44318_2024_78_MOESM8_ESM.zip › Appendix Figure/Appendix Figure S3/S3D/sGCCtr-4.tif]

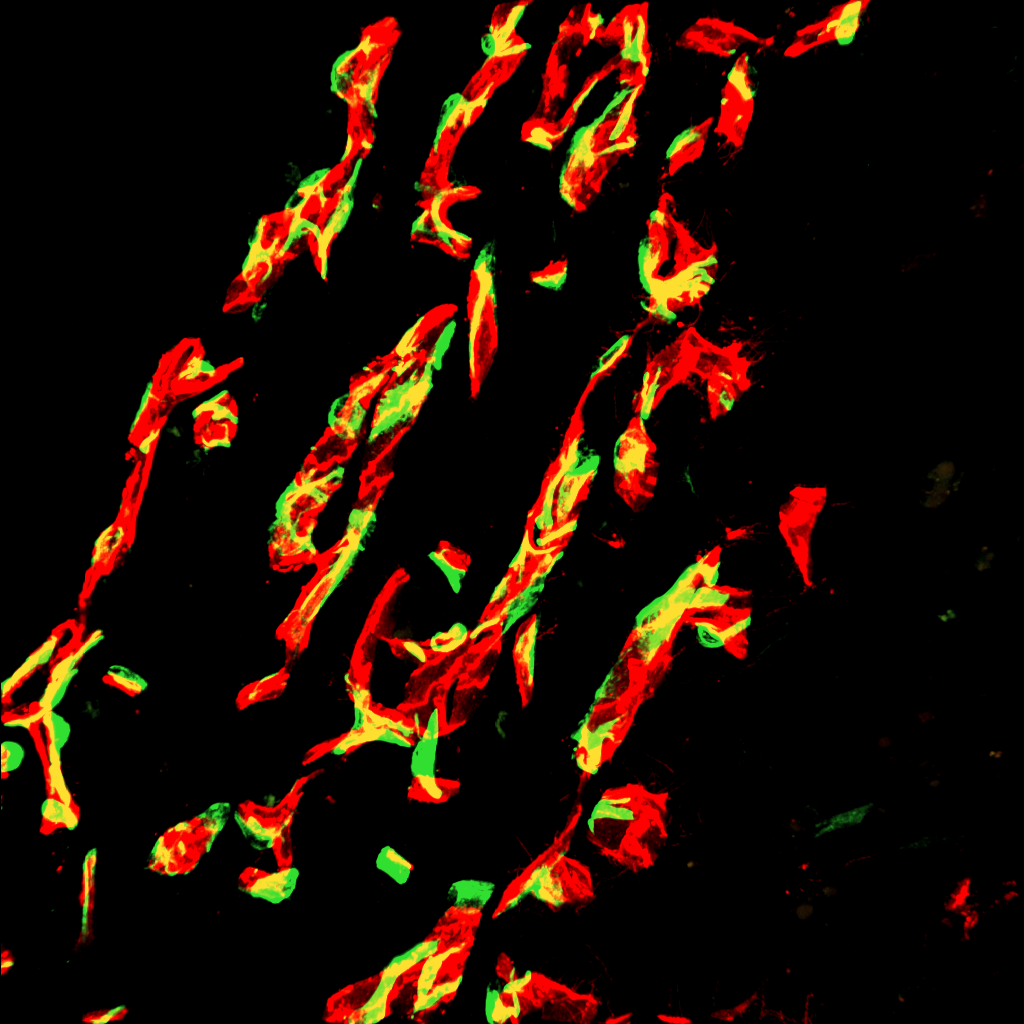

Supplement: Supplementary file 8 — Appendix Figure Source Data [file 44318_2024_78_MOESM8_ESM.zip › Appendix Figure/Appendix Figure S3/S3D/sGCCtr-3.tif]

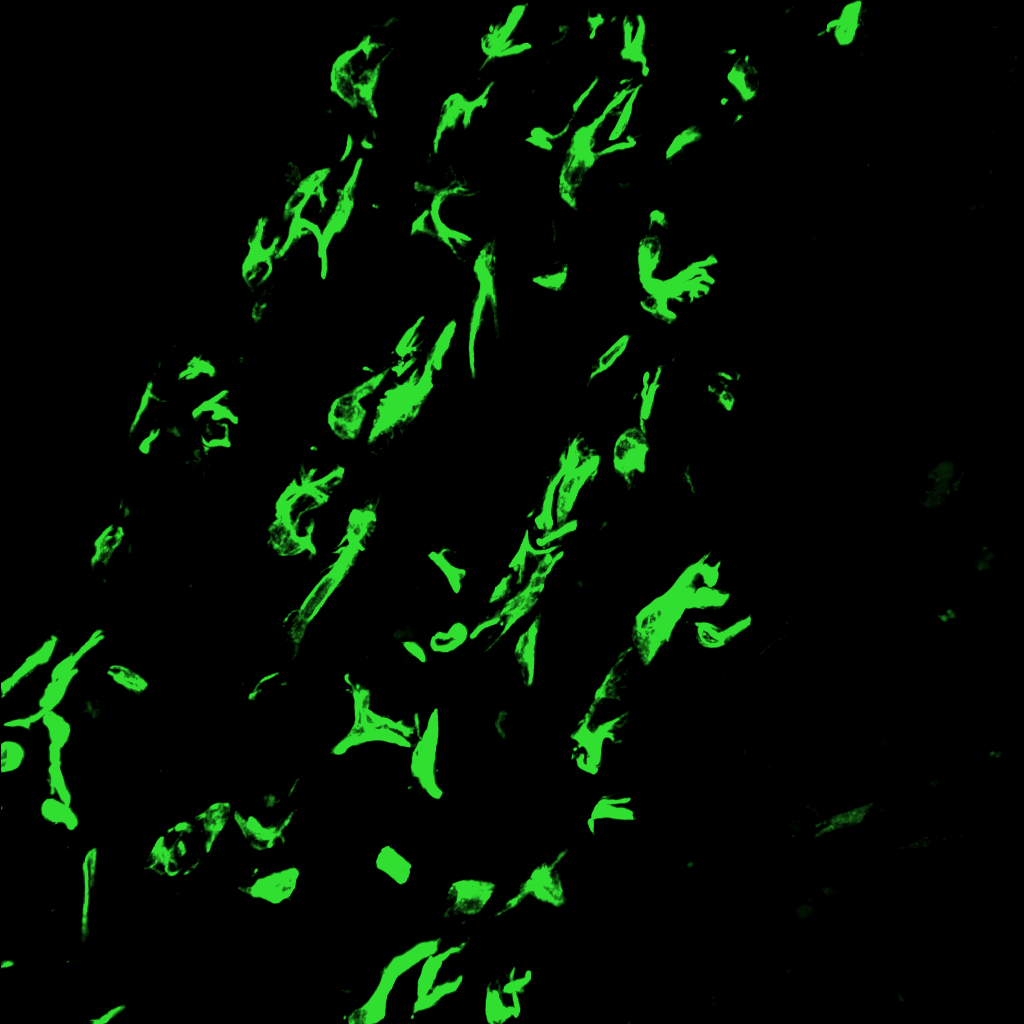

Supplement: Supplementary file 8 — Appendix Figure Source Data [file 44318_2024_78_MOESM8_ESM.zip › Appendix Figure/Appendix Figure S3/S3D/sGCCtr-2.tif]

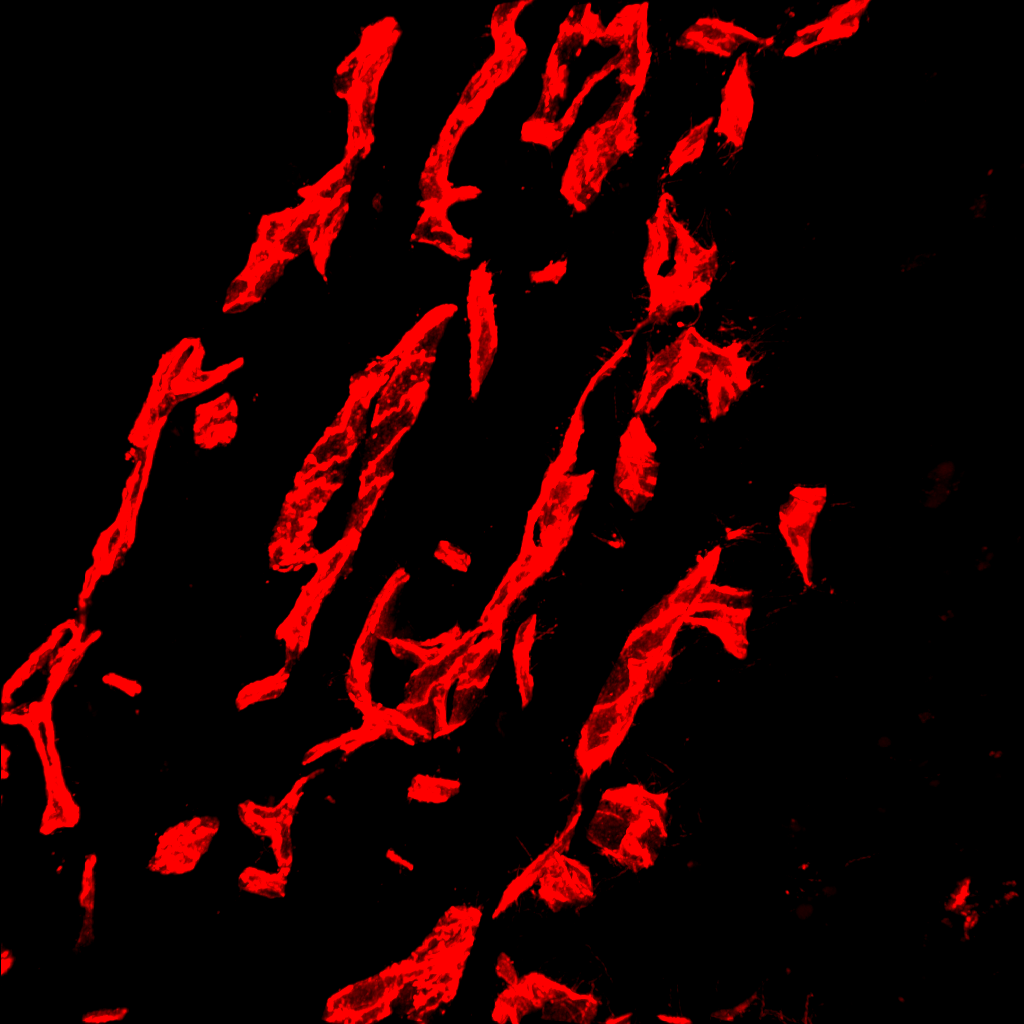

Supplement: Supplementary file 8 — Appendix Figure Source Data [file 44318_2024_78_MOESM8_ESM.zip › Appendix Figure/Appendix Figure S3/S3D/sGCCtr-1.tif]

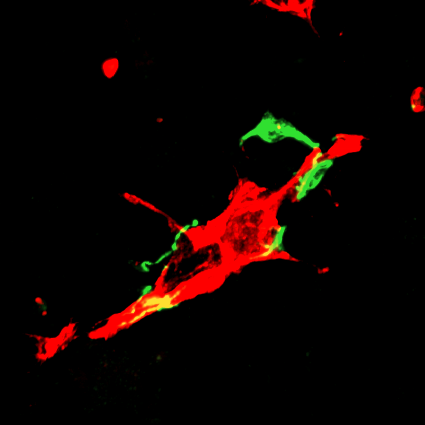

Supplement: Supplementary file 8 — Appendix Figure Source Data [file 44318_2024_78_MOESM8_ESM.zip › Appendix Figure/Appendix Figure S3/S3D/sGC╬öpc-4.tif]

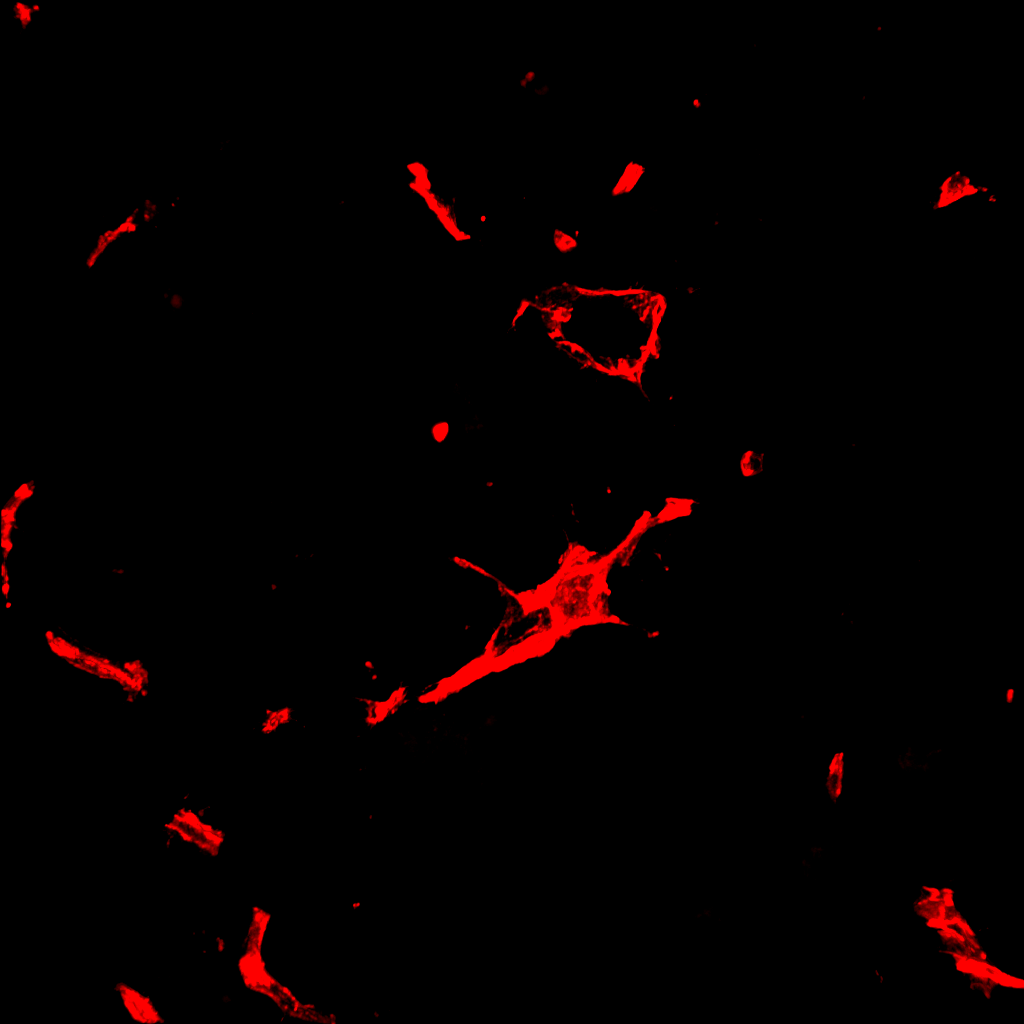

Supplement: Supplementary file 8 — Appendix Figure Source Data [file 44318_2024_78_MOESM8_ESM.zip › Appendix Figure/Appendix Figure S3/S3D/sGC╬öpc-1.tif]

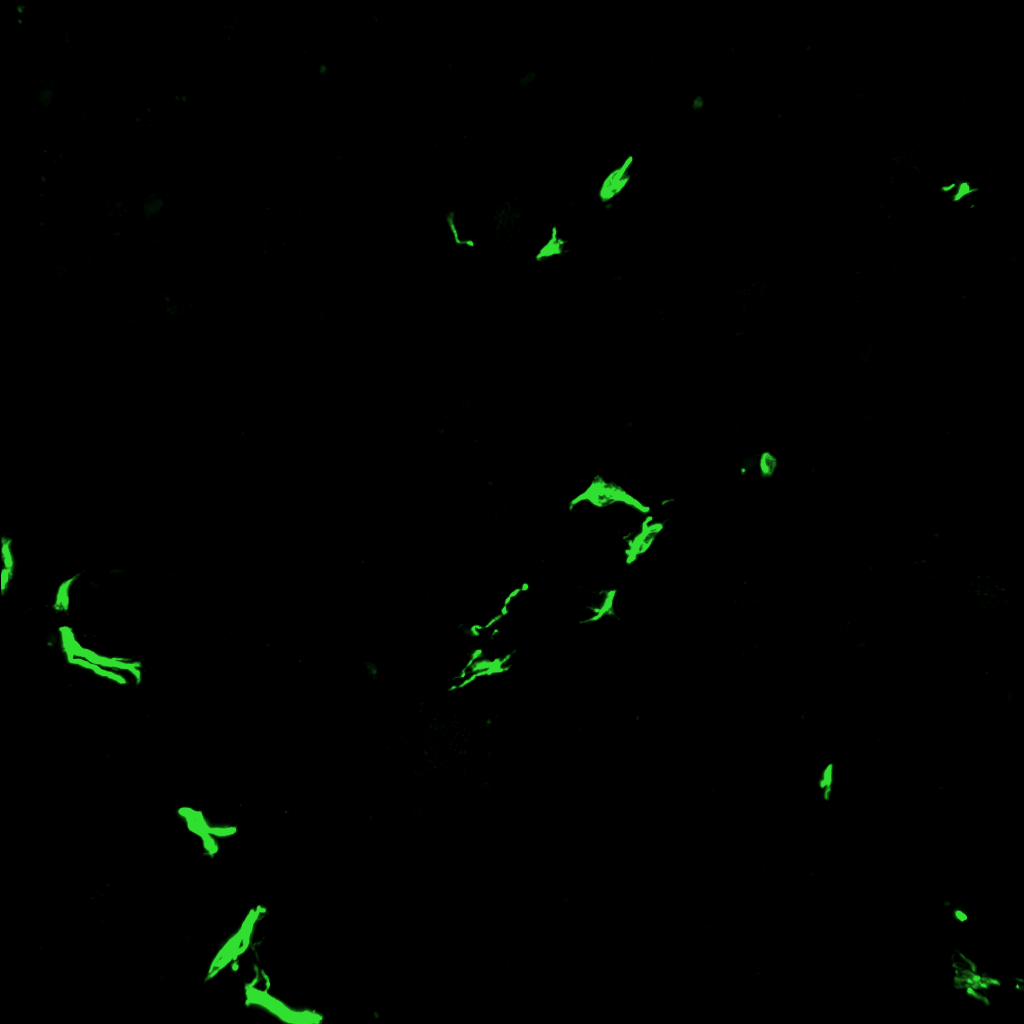

Supplement: Supplementary file 8 — Appendix Figure Source Data [file 44318_2024_78_MOESM8_ESM.zip › Appendix Figure/Appendix Figure S3/S3D/sGC╬öpc-2.tif]

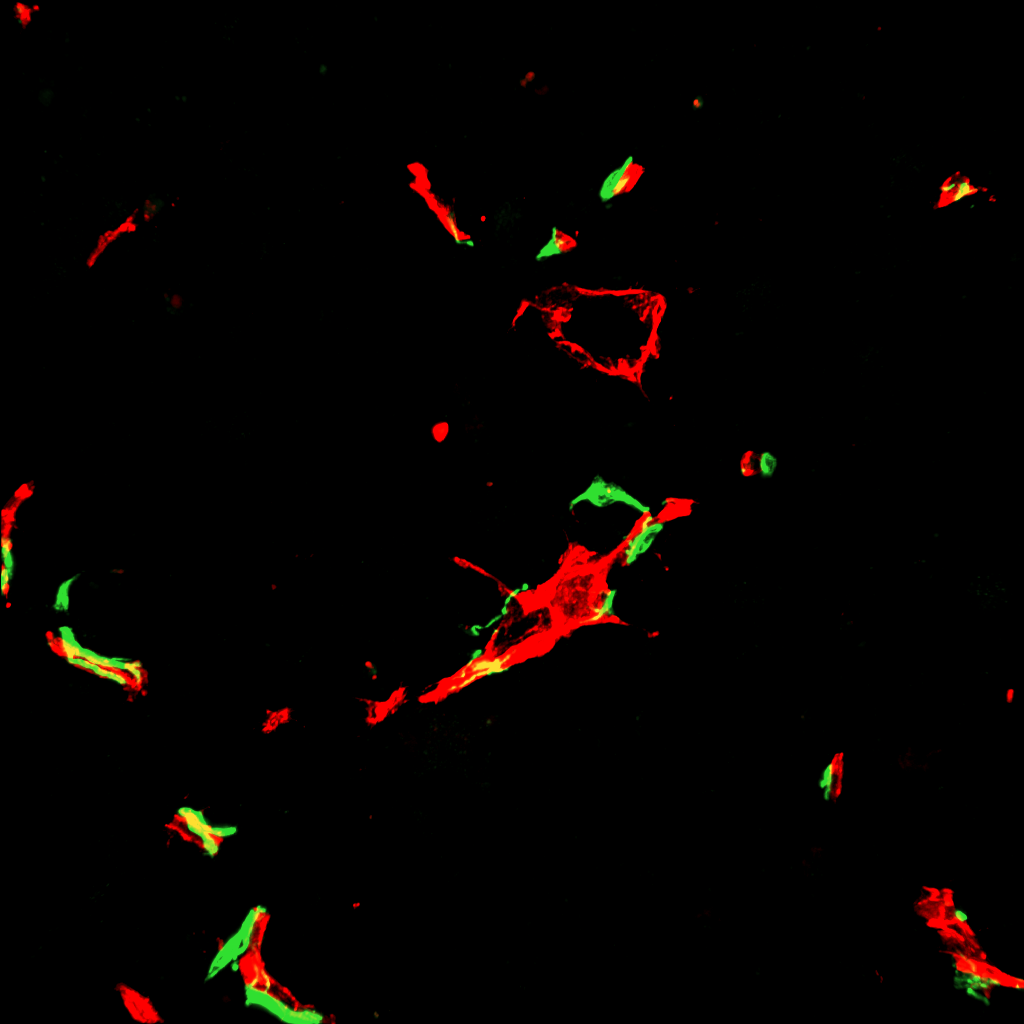

Supplement: Supplementary file 8 — Appendix Figure Source Data [file 44318_2024_78_MOESM8_ESM.zip › Appendix Figure/Appendix Figure S3/S3D/sGC╬öpc-3.tif]

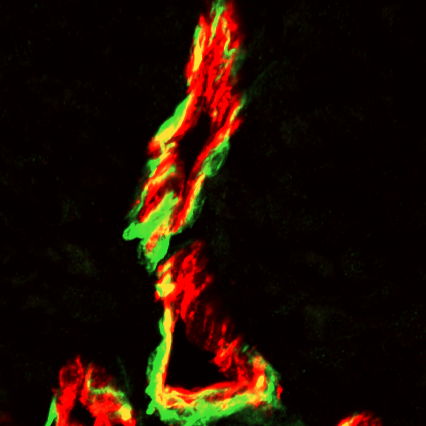

Supplement: Supplementary file 8 — Appendix Figure Source Data [file 44318_2024_78_MOESM8_ESM.zip › Appendix Figure/Appendix Figure S3/S3B/sGCCtr-4.tif]

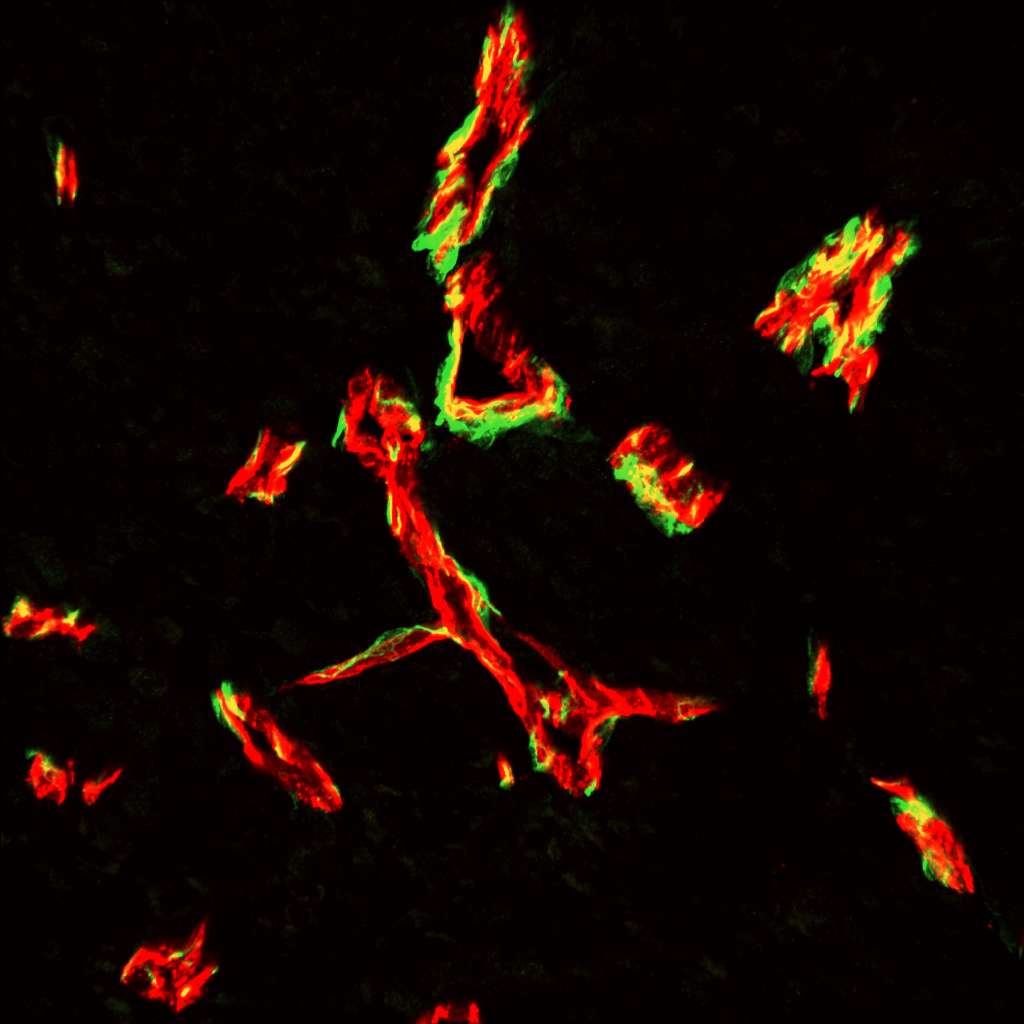

Supplement: Supplementary file 8 — Appendix Figure Source Data [file 44318_2024_78_MOESM8_ESM.zip › Appendix Figure/Appendix Figure S3/S3B/sGCCtr-3.tif]

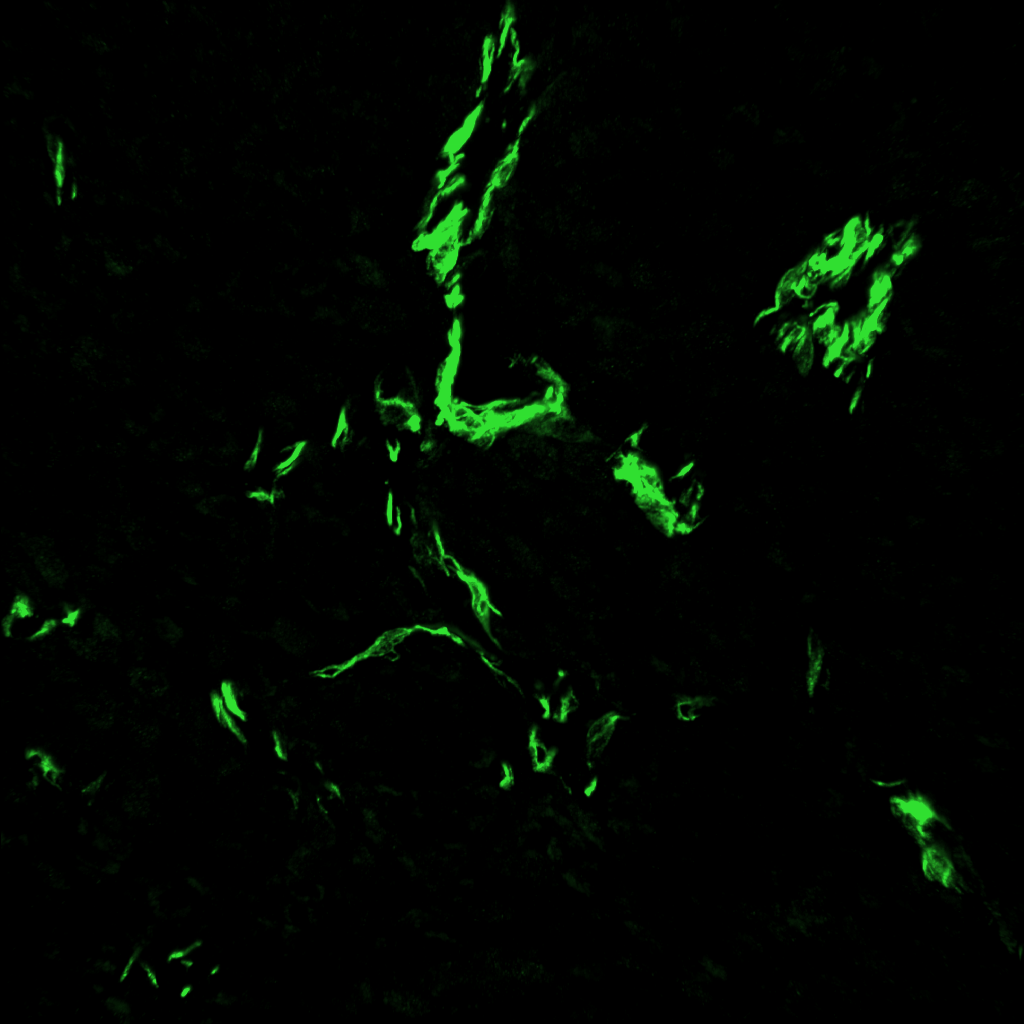

Supplement: Supplementary file 8 — Appendix Figure Source Data [file 44318_2024_78_MOESM8_ESM.zip › Appendix Figure/Appendix Figure S3/S3B/sGCCtr-2.tif]

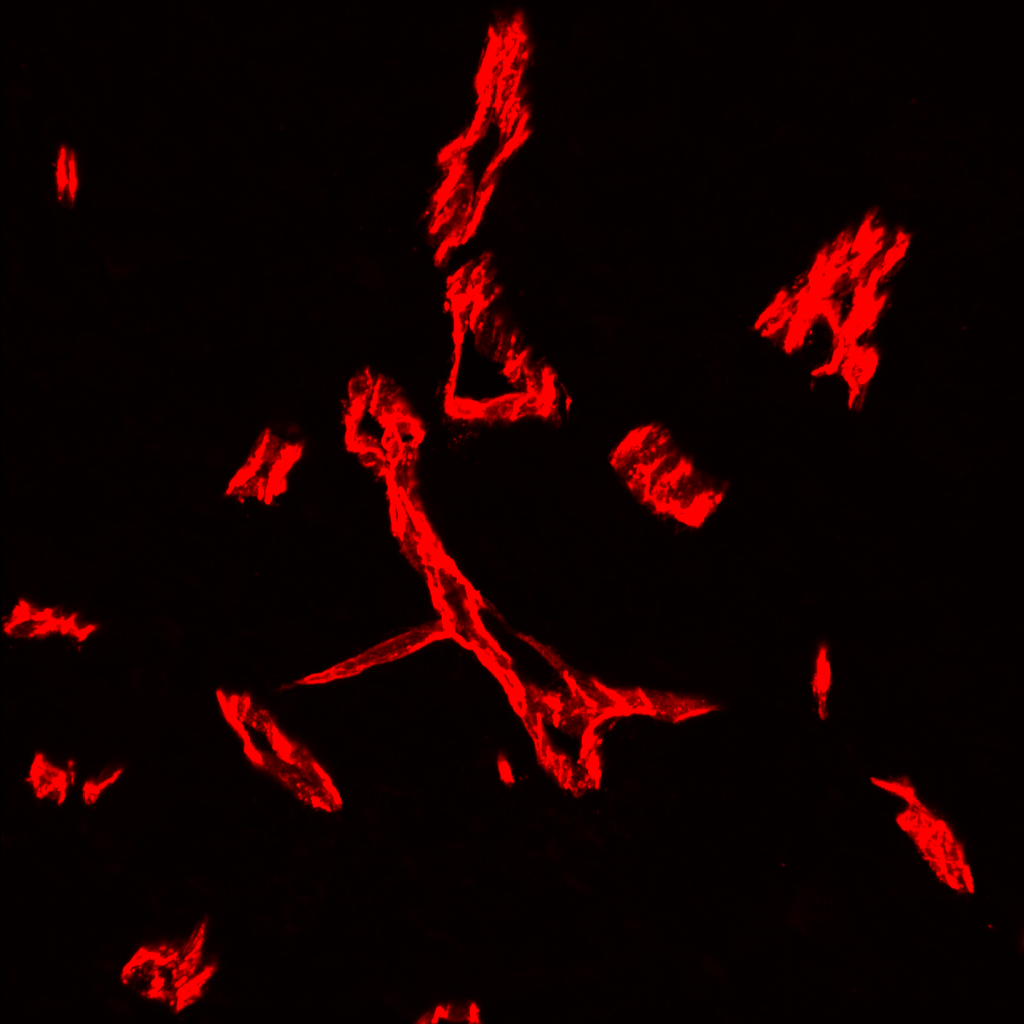

Supplement: Supplementary file 8 — Appendix Figure Source Data [file 44318_2024_78_MOESM8_ESM.zip › Appendix Figure/Appendix Figure S3/S3B/sGCCtr-1.tif]

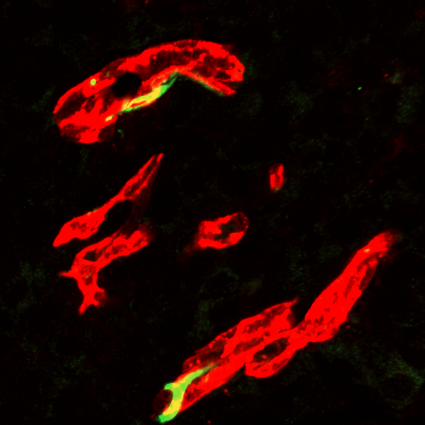

Supplement: Supplementary file 8 — Appendix Figure Source Data [file 44318_2024_78_MOESM8_ESM.zip › Appendix Figure/Appendix Figure S3/S3B/sGC╬öpc-4.tif]

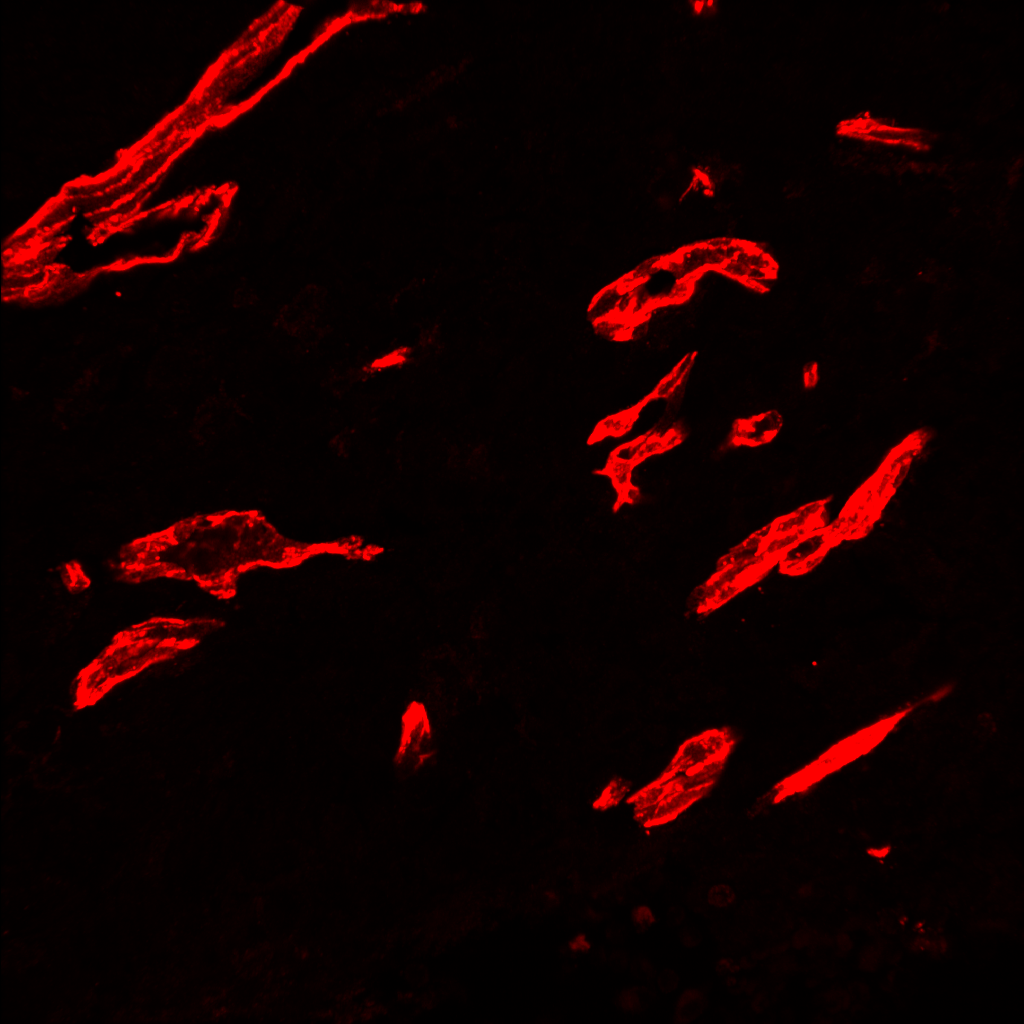

Supplement: Supplementary file 8 — Appendix Figure Source Data [file 44318_2024_78_MOESM8_ESM.zip › Appendix Figure/Appendix Figure S3/S3B/sGC╬öpc-1.tif]
