# Supplementary figures and images for: Pericyte signaling via soluble guanylate cyclase shapes the vascular niche and microenvironment of tumors (part 4 of 4)
Source: EMBO J. 2024 Mar 25;43(8):7. doi: 10.1038/s44318-024-00078-5 (PMC11021551; doi:10.1038/s44318-024-00078-5)

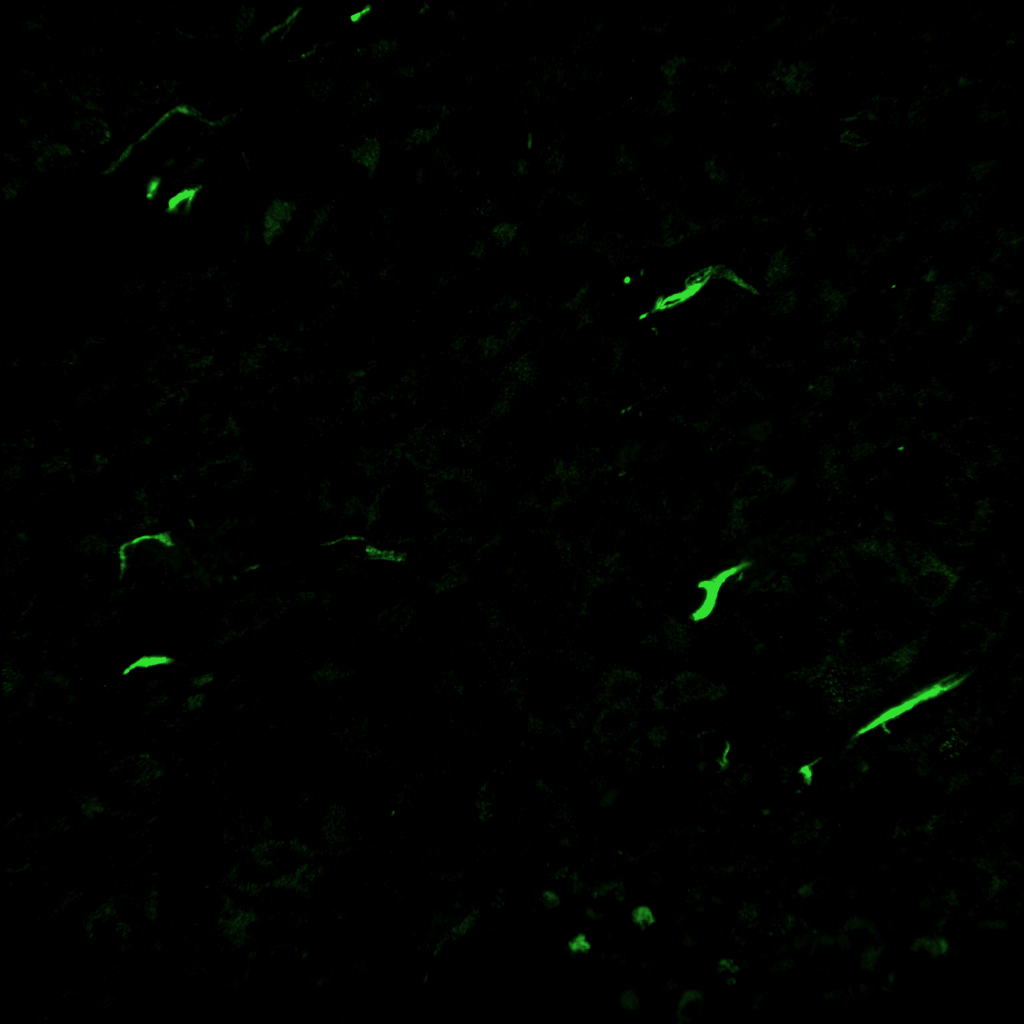

Supplement: Supplementary file 8 — Appendix Figure Source Data [file 44318_2024_78_MOESM8_ESM.zip › Appendix Figure/Appendix Figure S3/S3B/sGC╬öpc-2.tif]

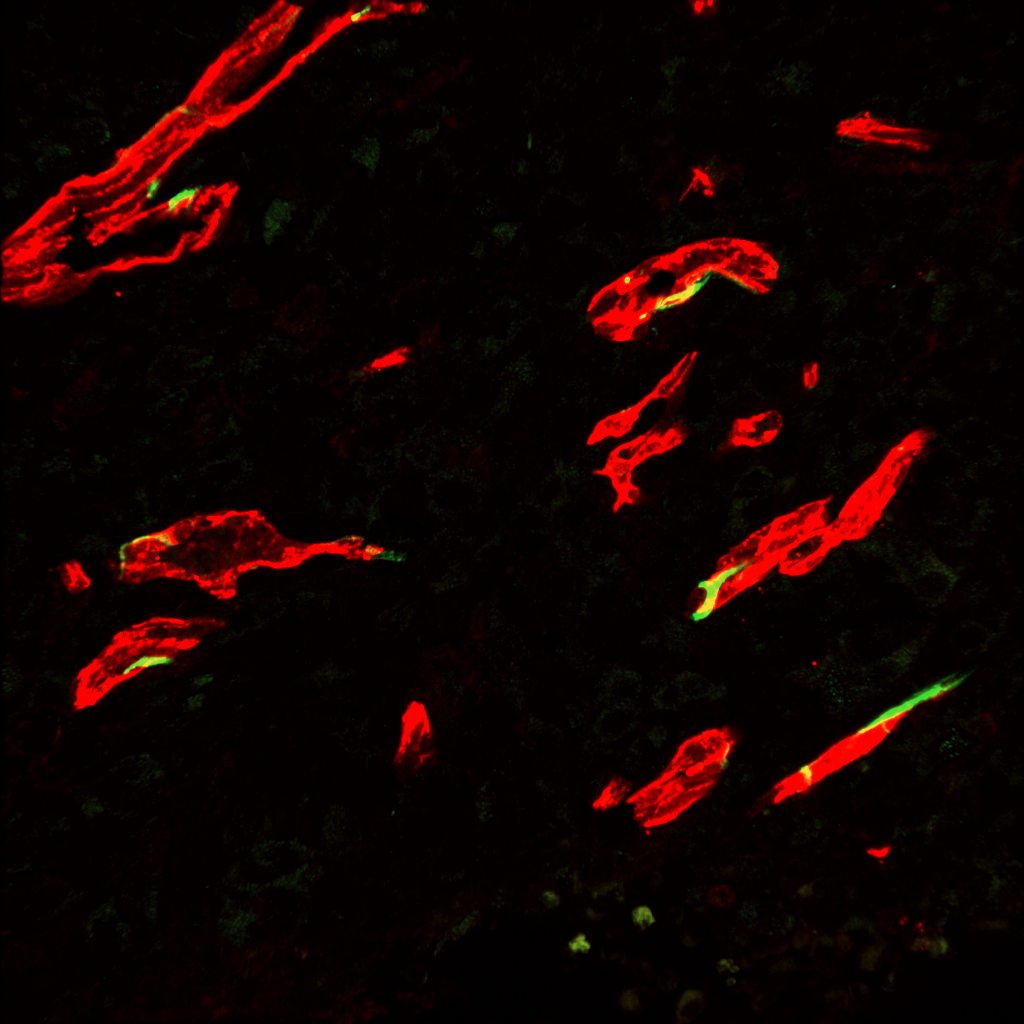

Supplement: Supplementary file 8 — Appendix Figure Source Data [file 44318_2024_78_MOESM8_ESM.zip › Appendix Figure/Appendix Figure S3/S3B/sGC╬öpc-3.tif]

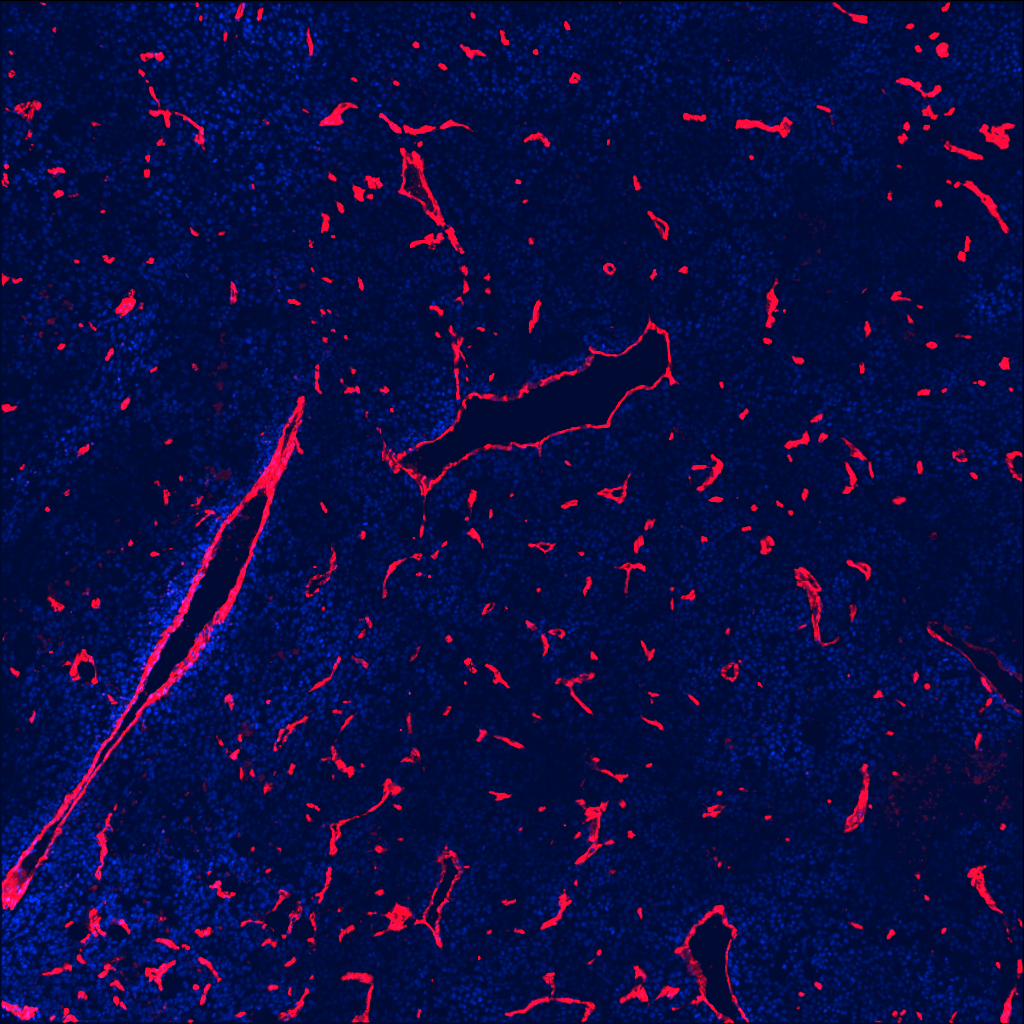

Supplement: Supplementary file 8 — Appendix Figure Source Data [file 44318_2024_78_MOESM8_ESM.zip › Appendix Figure/Appendix Figure S3/S3A/sGCCtr-3.tif]

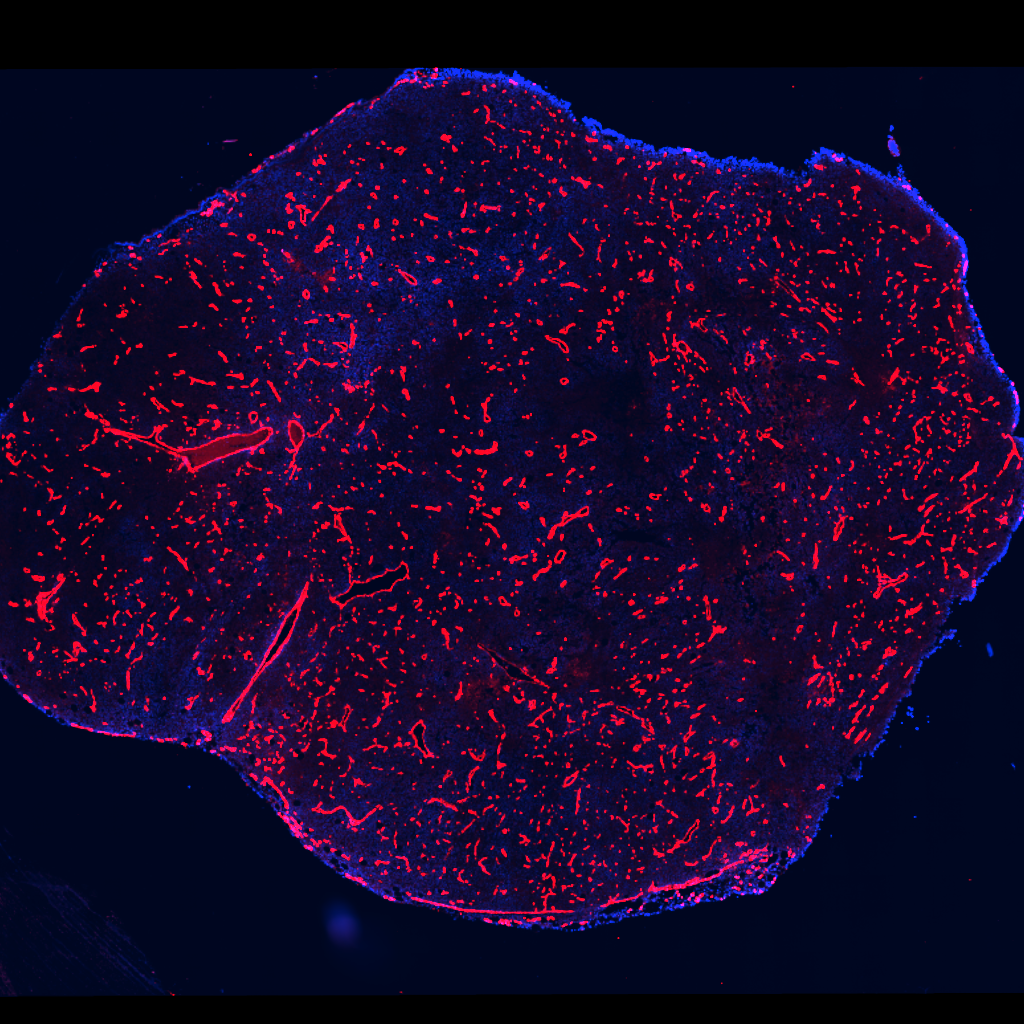

Supplement: Supplementary file 8 — Appendix Figure Source Data [file 44318_2024_78_MOESM8_ESM.zip › Appendix Figure/Appendix Figure S3/S3A/sGCCtr-2.tif]

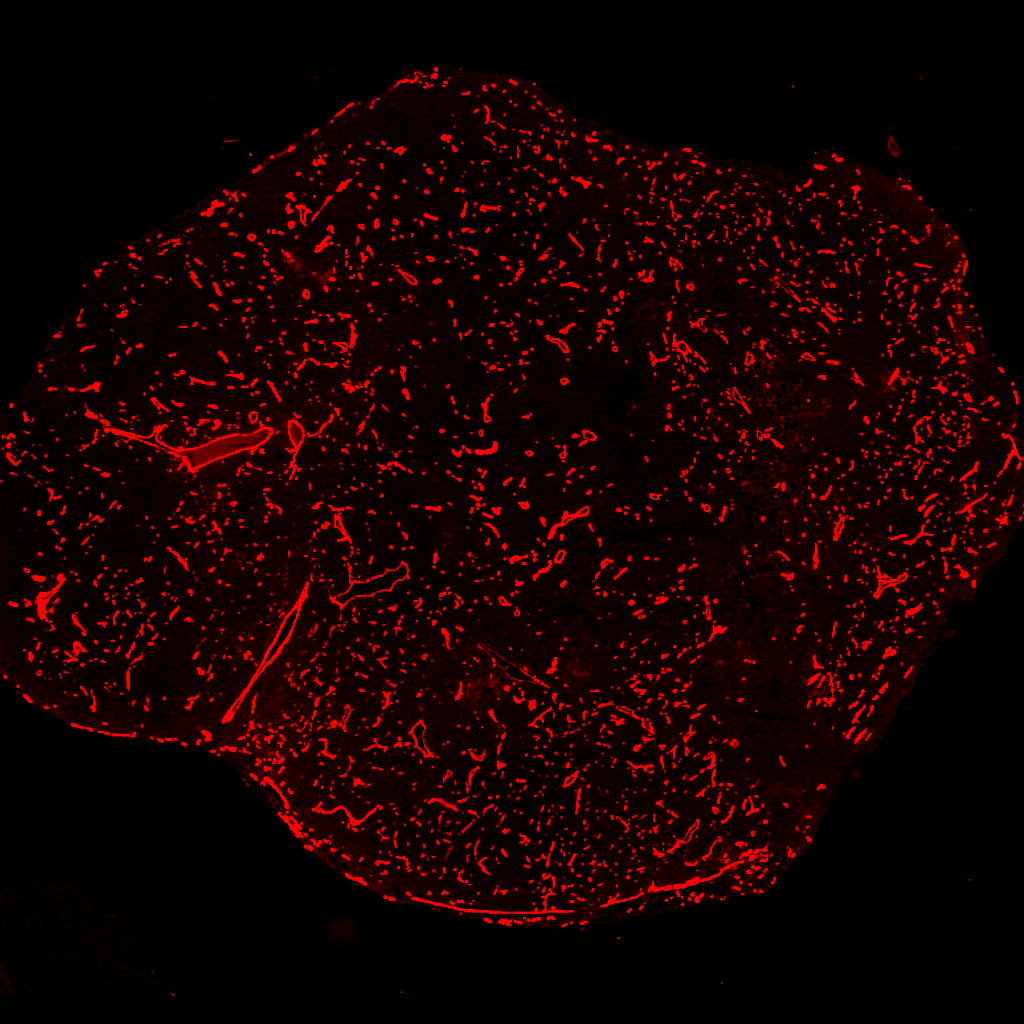

Supplement: Supplementary file 8 — Appendix Figure Source Data [file 44318_2024_78_MOESM8_ESM.zip › Appendix Figure/Appendix Figure S3/S3A/sGCCtr-1.tif]

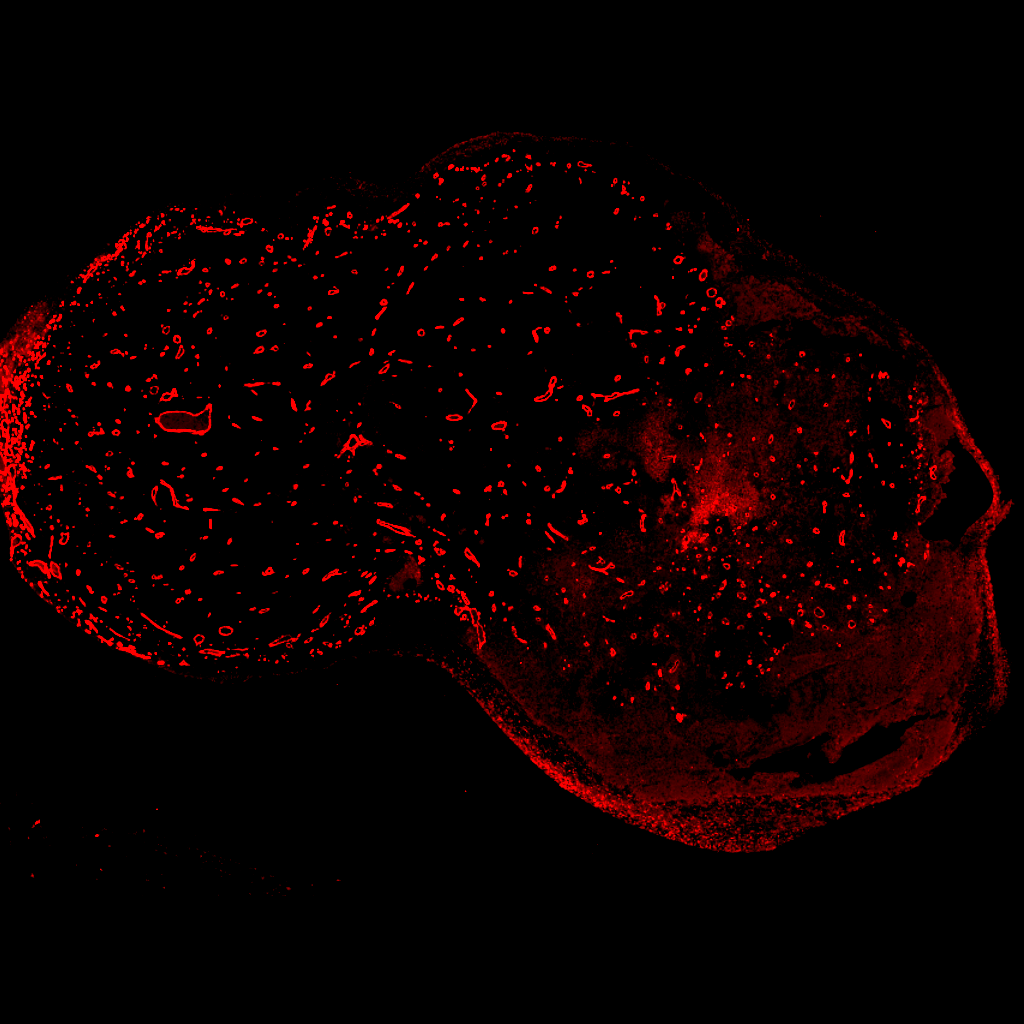

Supplement: Supplementary file 8 — Appendix Figure Source Data [file 44318_2024_78_MOESM8_ESM.zip › Appendix Figure/Appendix Figure S3/S3A/sGC╬öpc-1.tif]

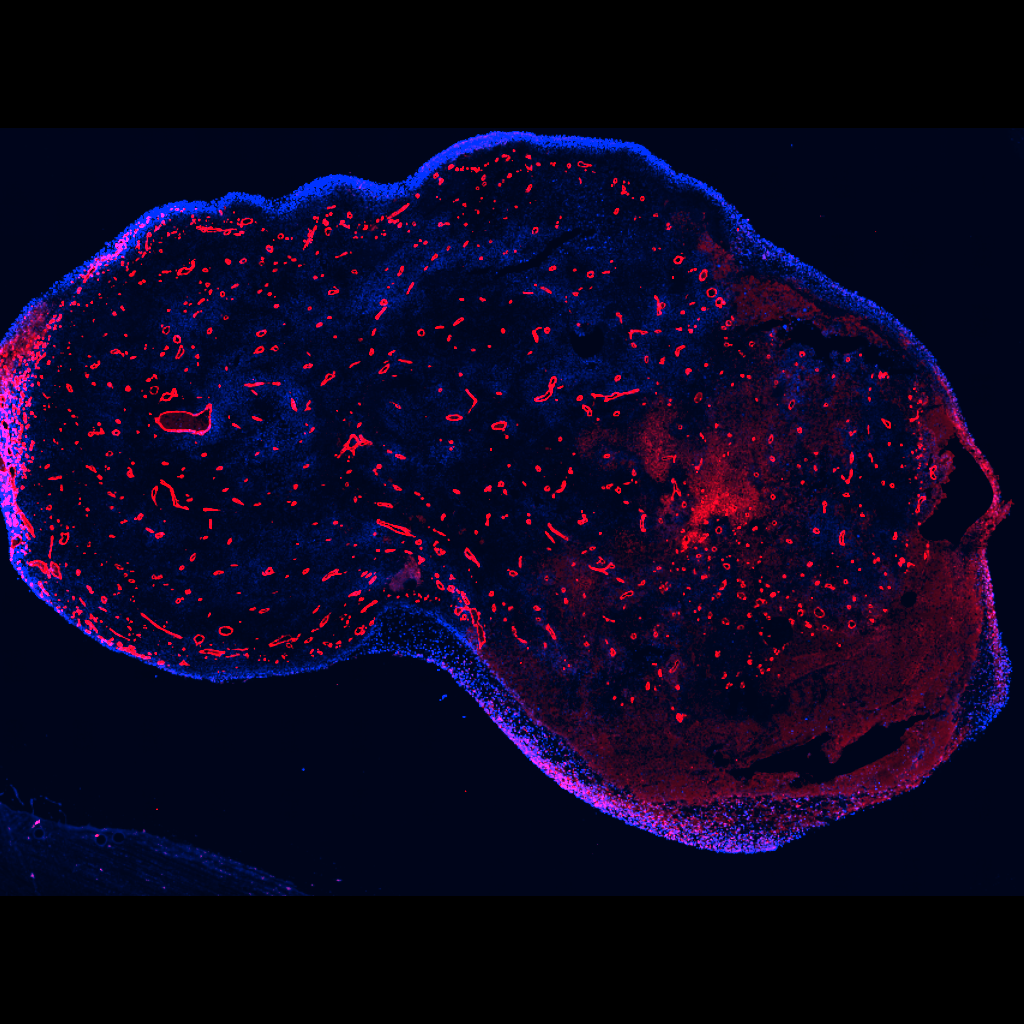

Supplement: Supplementary file 8 — Appendix Figure Source Data [file 44318_2024_78_MOESM8_ESM.zip › Appendix Figure/Appendix Figure S3/S3A/sGC╬öpc-2.tif]

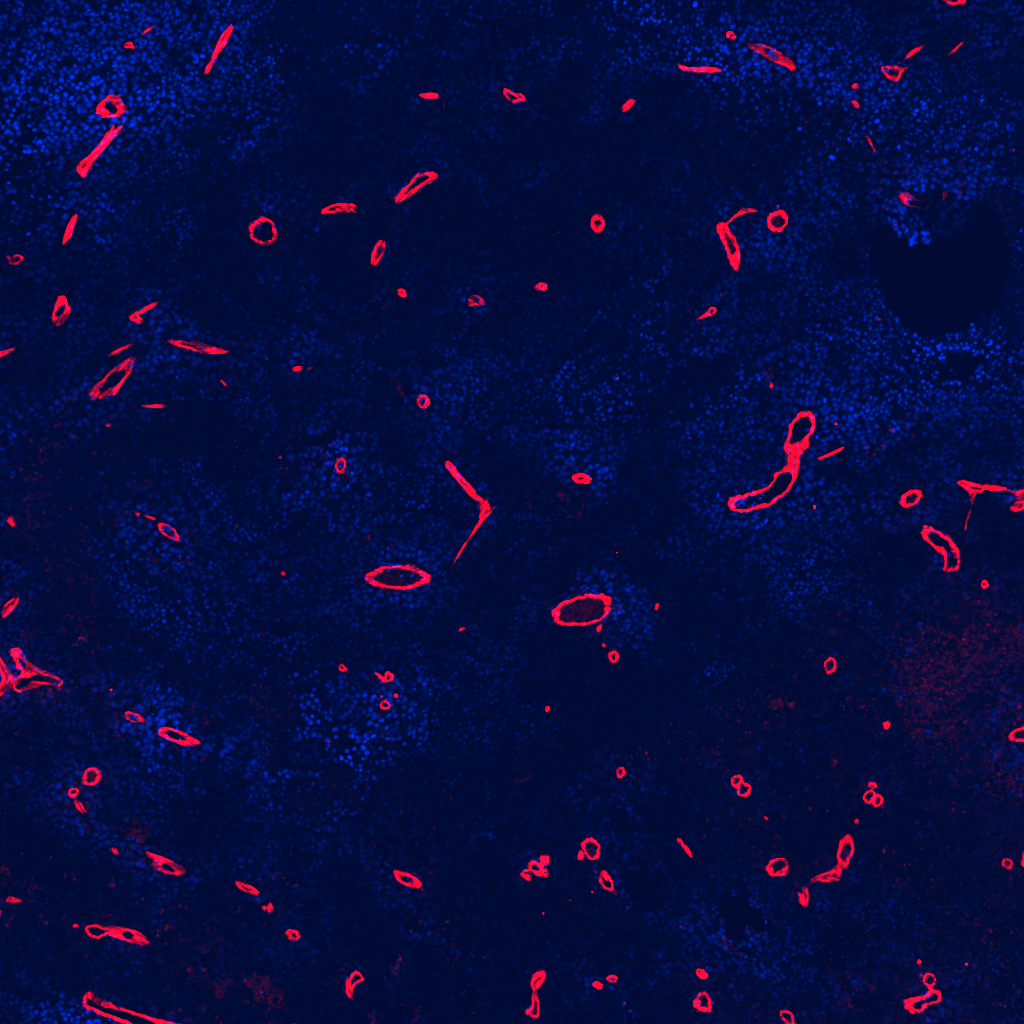

Supplement: Supplementary file 8 — Appendix Figure Source Data [file 44318_2024_78_MOESM8_ESM.zip › Appendix Figure/Appendix Figure S3/S3A/sGC╬öpc-3.tif]

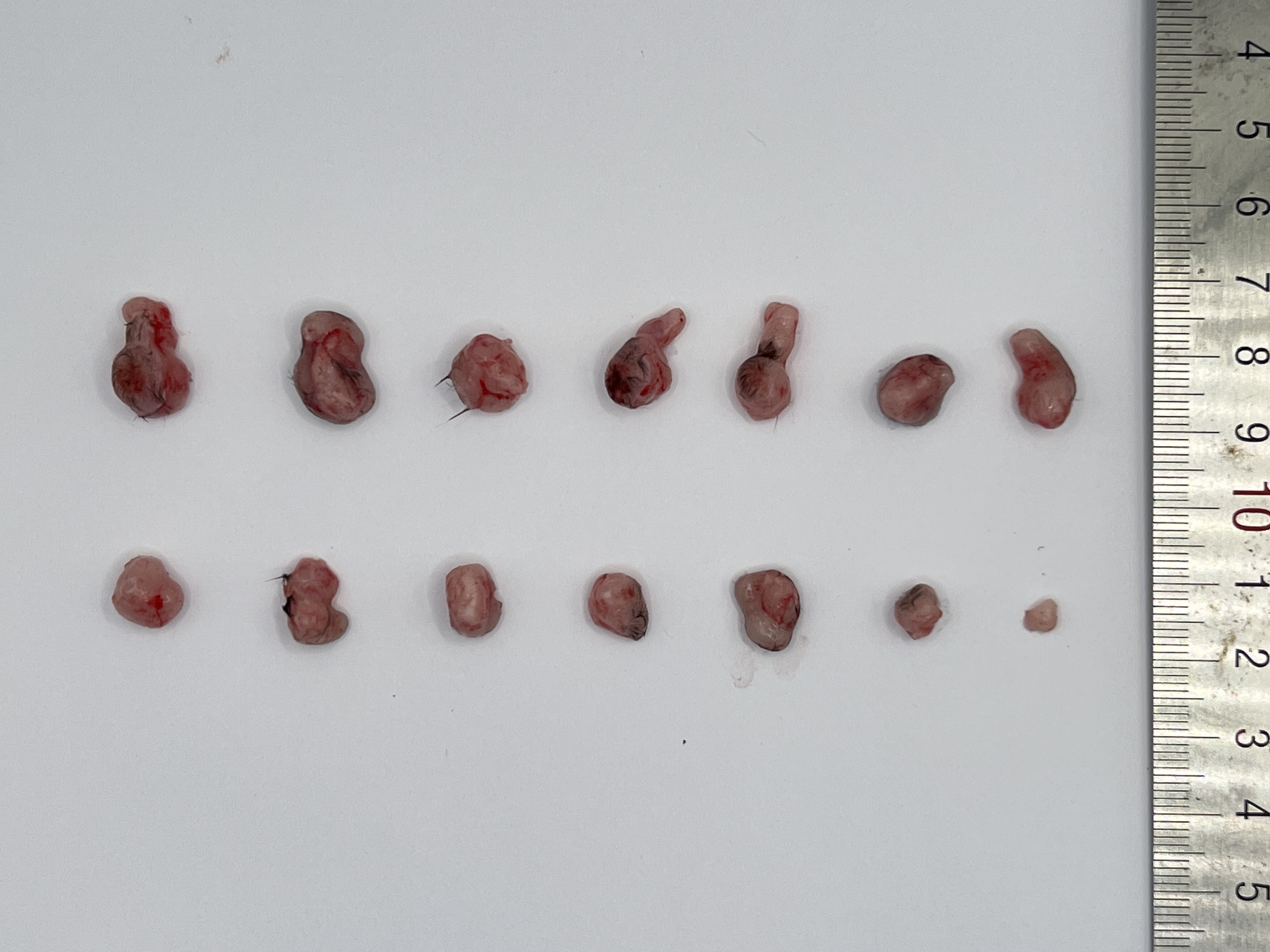

Supplement: Supplementary file 8 — Appendix Figure Source Data [file 44318_2024_78_MOESM8_ESM.zip › Appendix Figure/Appendix Figure S2/S2E/EO771 TUMOR.jpg]

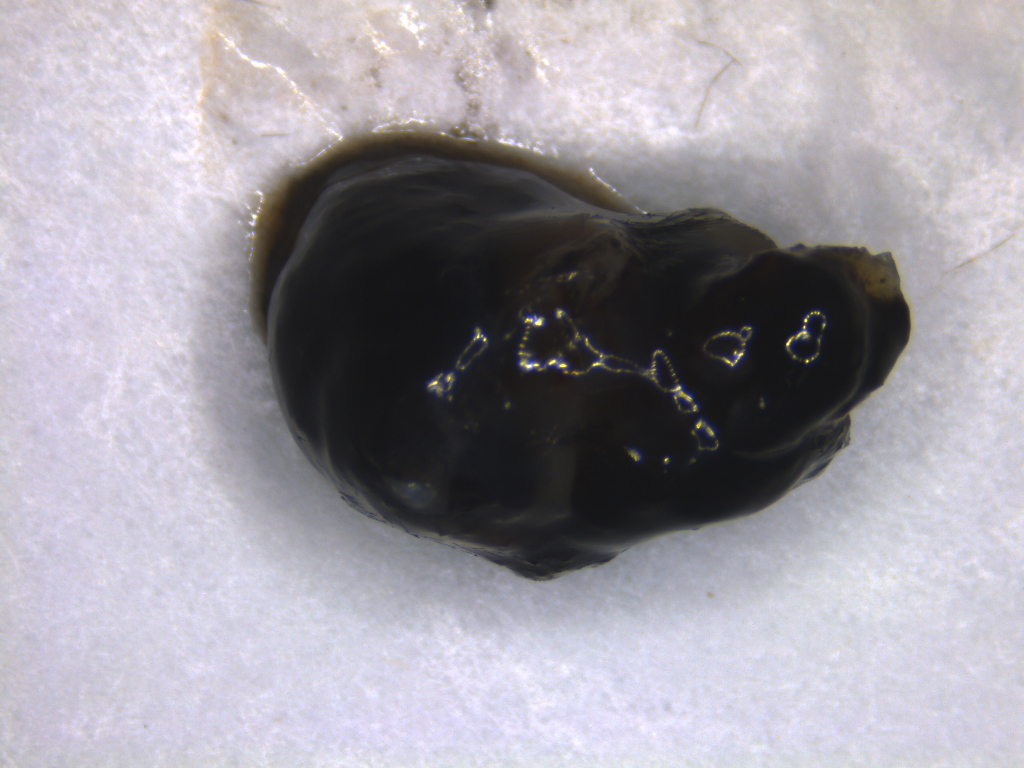

Supplement: Supplementary file 8 — Appendix Figure Source Data [file 44318_2024_78_MOESM8_ESM.zip › Appendix Figure/Appendix Figure S2/S2B/sGCCtr-5.tif]

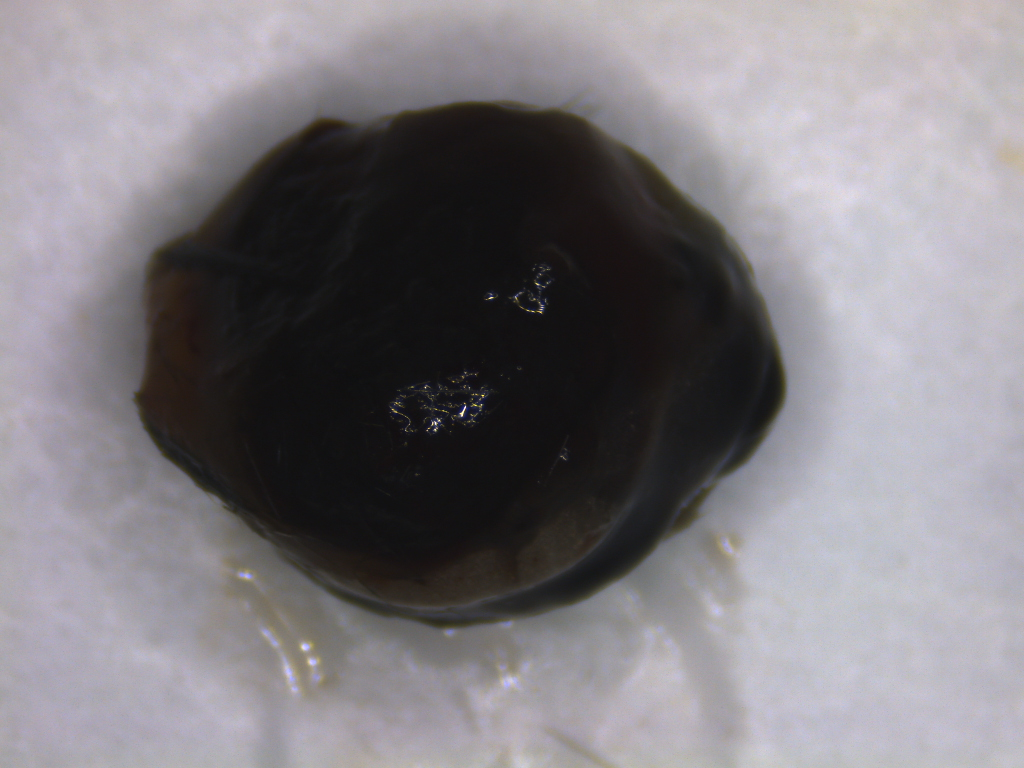

Supplement: Supplementary file 8 — Appendix Figure Source Data [file 44318_2024_78_MOESM8_ESM.zip › Appendix Figure/Appendix Figure S2/S2B/sGCCtr-4.tif]

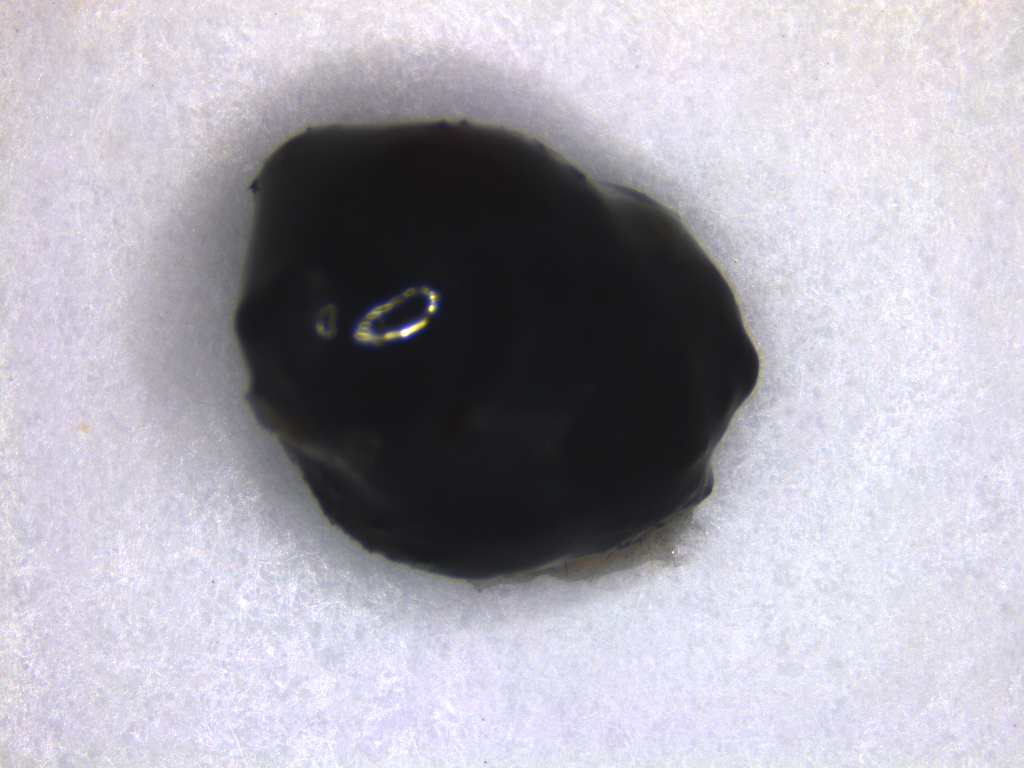

Supplement: Supplementary file 8 — Appendix Figure Source Data [file 44318_2024_78_MOESM8_ESM.zip › Appendix Figure/Appendix Figure S2/S2B/sGCCtr-6.tif]

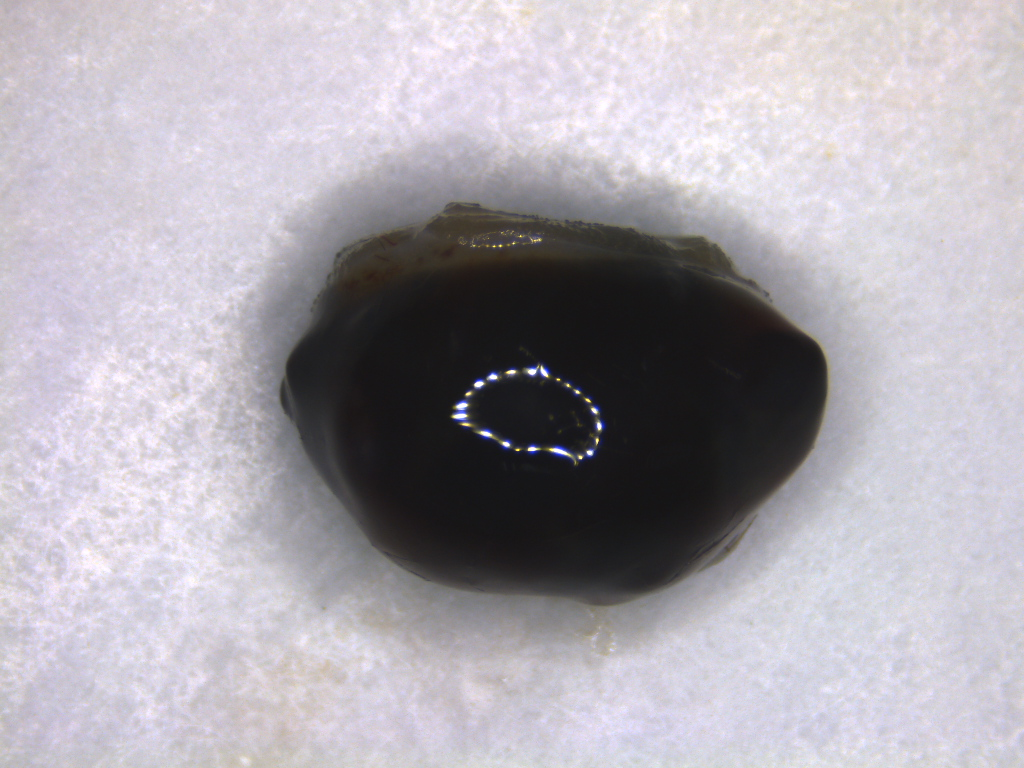

Supplement: Supplementary file 8 — Appendix Figure Source Data [file 44318_2024_78_MOESM8_ESM.zip › Appendix Figure/Appendix Figure S2/S2B/sGCCtr-7.tif]

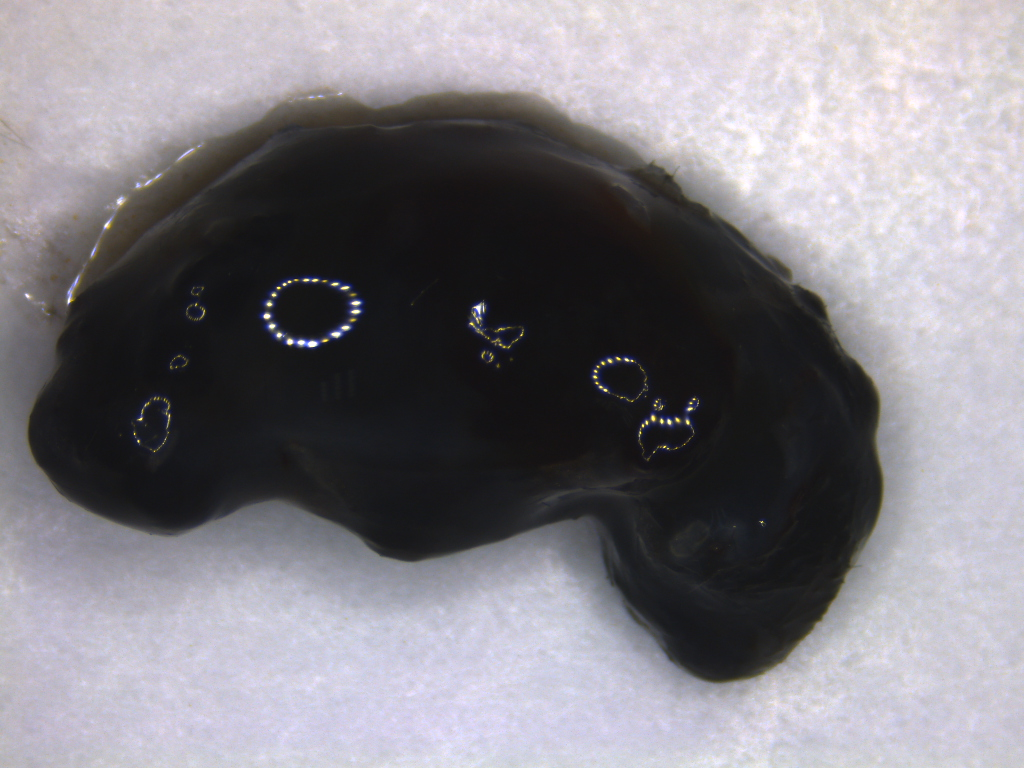

Supplement: Supplementary file 8 — Appendix Figure Source Data [file 44318_2024_78_MOESM8_ESM.zip › Appendix Figure/Appendix Figure S2/S2B/sGCCtr-3.tif]

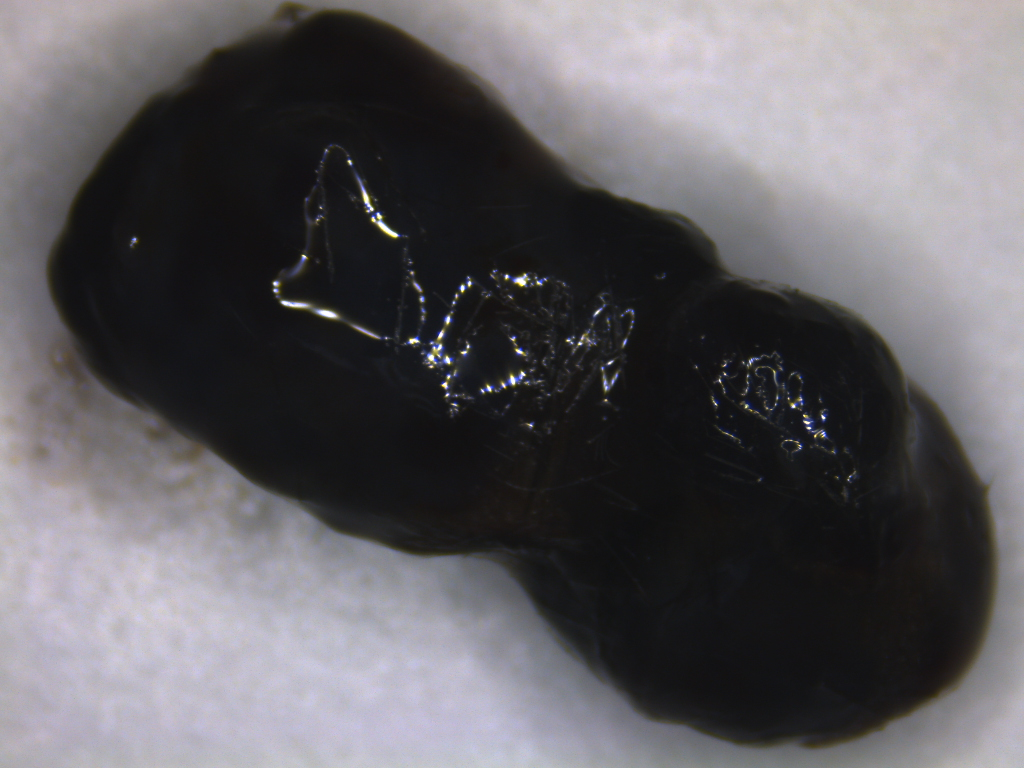

Supplement: Supplementary file 8 — Appendix Figure Source Data [file 44318_2024_78_MOESM8_ESM.zip › Appendix Figure/Appendix Figure S2/S2B/sGCCtr-2.tif]

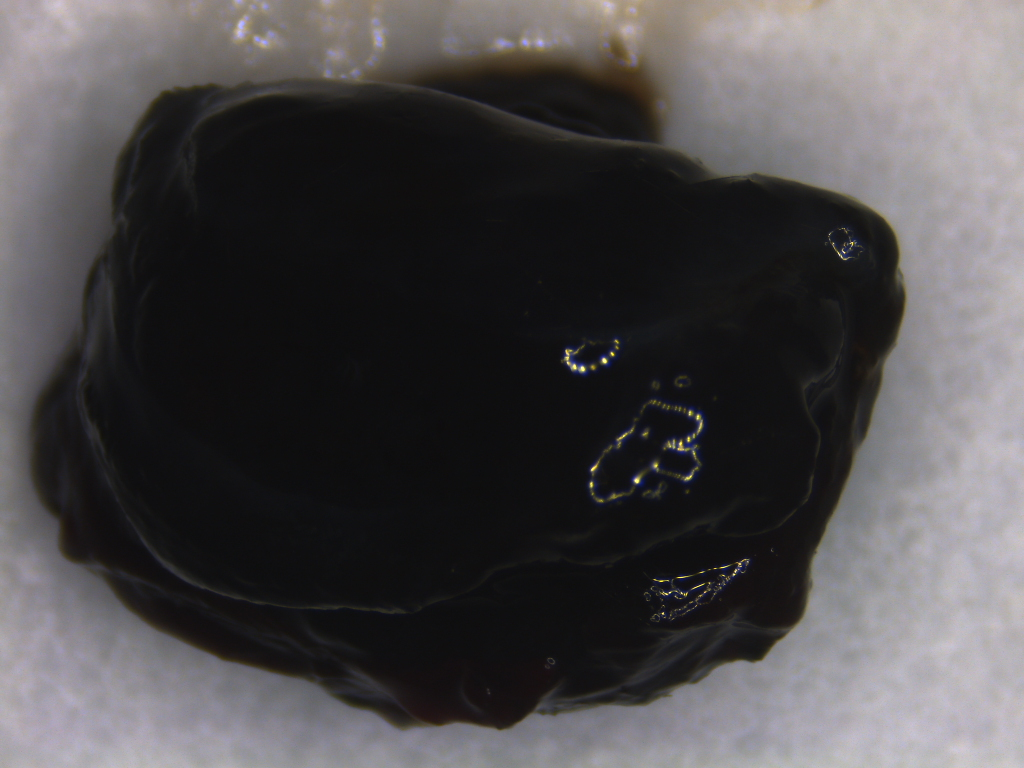

Supplement: Supplementary file 8 — Appendix Figure Source Data [file 44318_2024_78_MOESM8_ESM.zip › Appendix Figure/Appendix Figure S2/S2B/sGCCtr-1.tif]

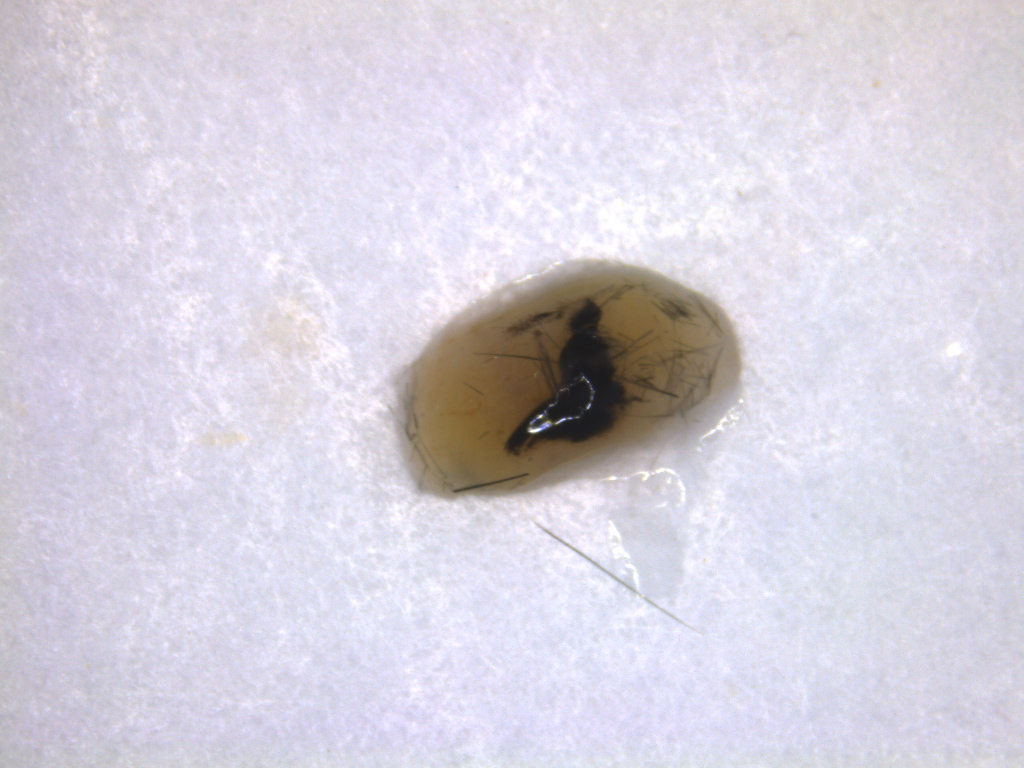

Supplement: Supplementary file 8 — Appendix Figure Source Data [file 44318_2024_78_MOESM8_ESM.zip › Appendix Figure/Appendix Figure S2/S2B/sGC╬öpc-7.tif]

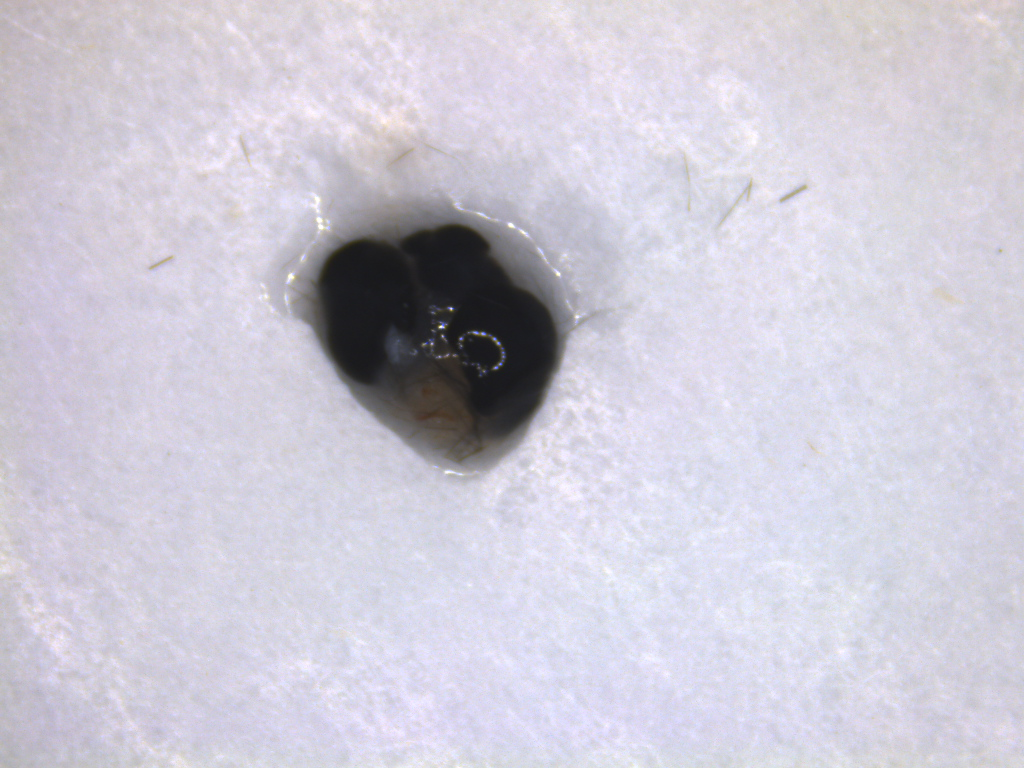

Supplement: Supplementary file 8 — Appendix Figure Source Data [file 44318_2024_78_MOESM8_ESM.zip › Appendix Figure/Appendix Figure S2/S2B/sGC╬öpc-6.tif]

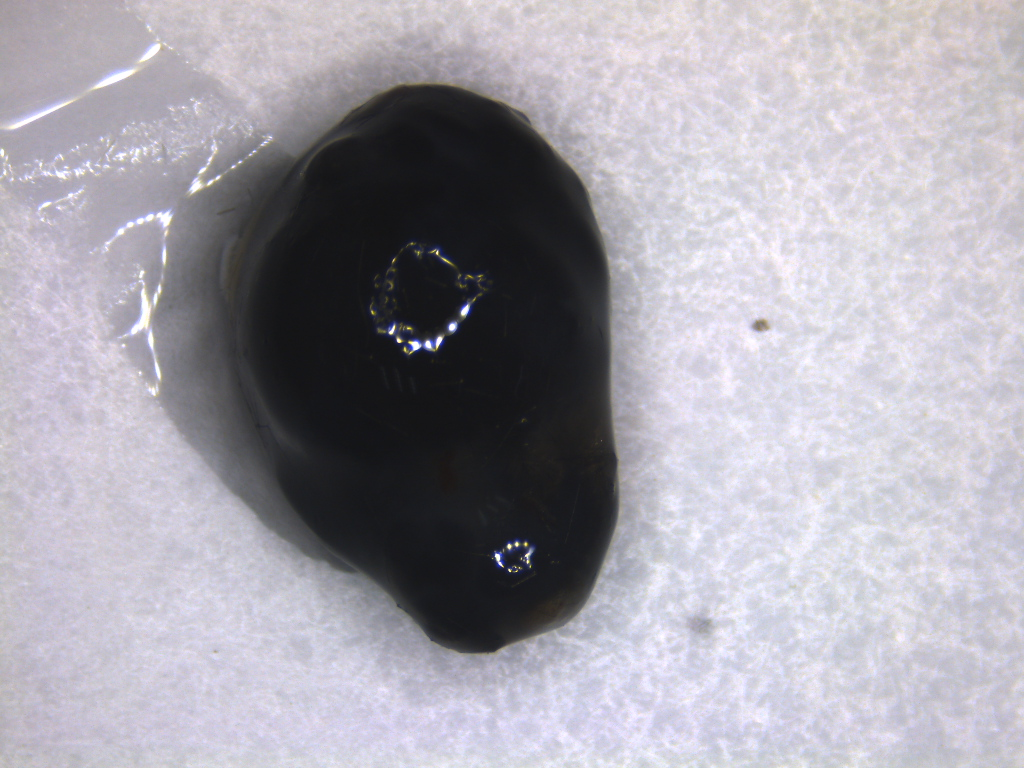

Supplement: Supplementary file 8 — Appendix Figure Source Data [file 44318_2024_78_MOESM8_ESM.zip › Appendix Figure/Appendix Figure S2/S2B/sGC╬öpc-4.tif]

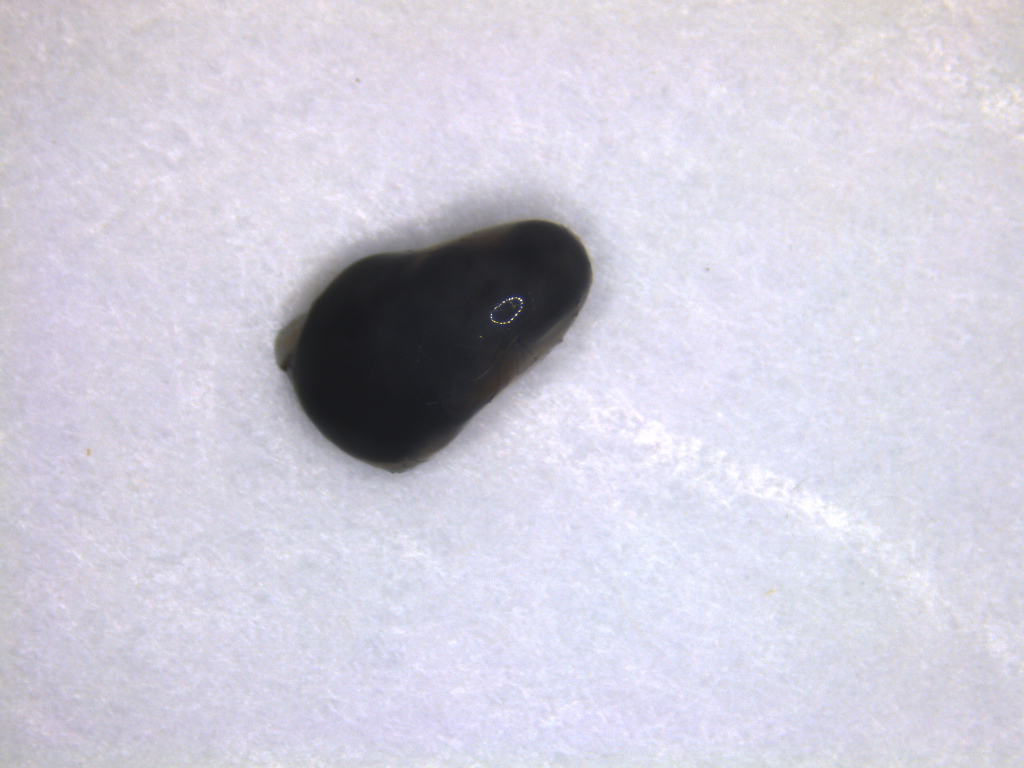

Supplement: Supplementary file 8 — Appendix Figure Source Data [file 44318_2024_78_MOESM8_ESM.zip › Appendix Figure/Appendix Figure S2/S2B/sGC╬öpc-5.tif]

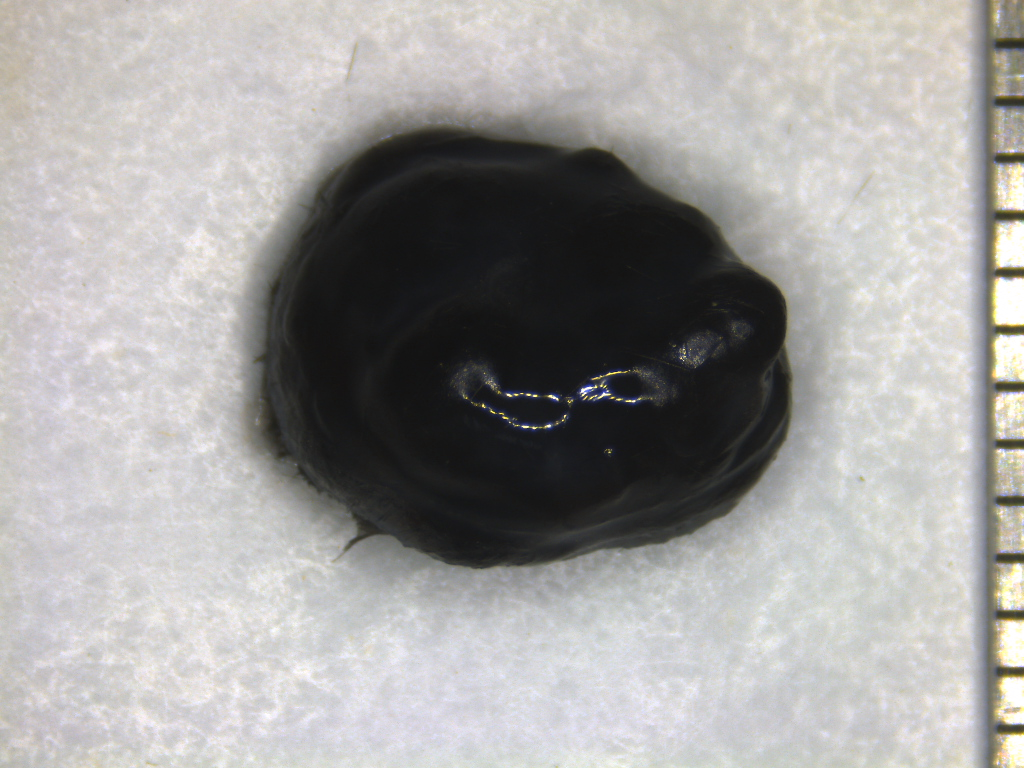

Supplement: Supplementary file 8 — Appendix Figure Source Data [file 44318_2024_78_MOESM8_ESM.zip › Appendix Figure/Appendix Figure S2/S2B/sGC╬öpc-1.tif]

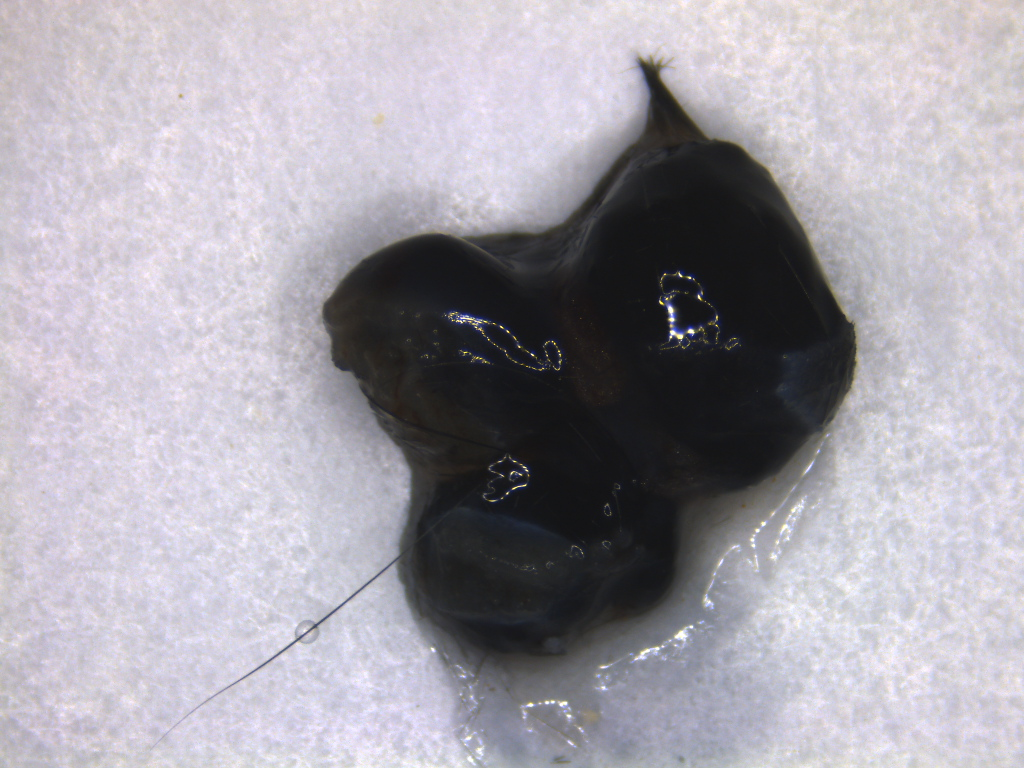

Supplement: Supplementary file 8 — Appendix Figure Source Data [file 44318_2024_78_MOESM8_ESM.zip › Appendix Figure/Appendix Figure S2/S2B/sGC╬öpc-2.tif]

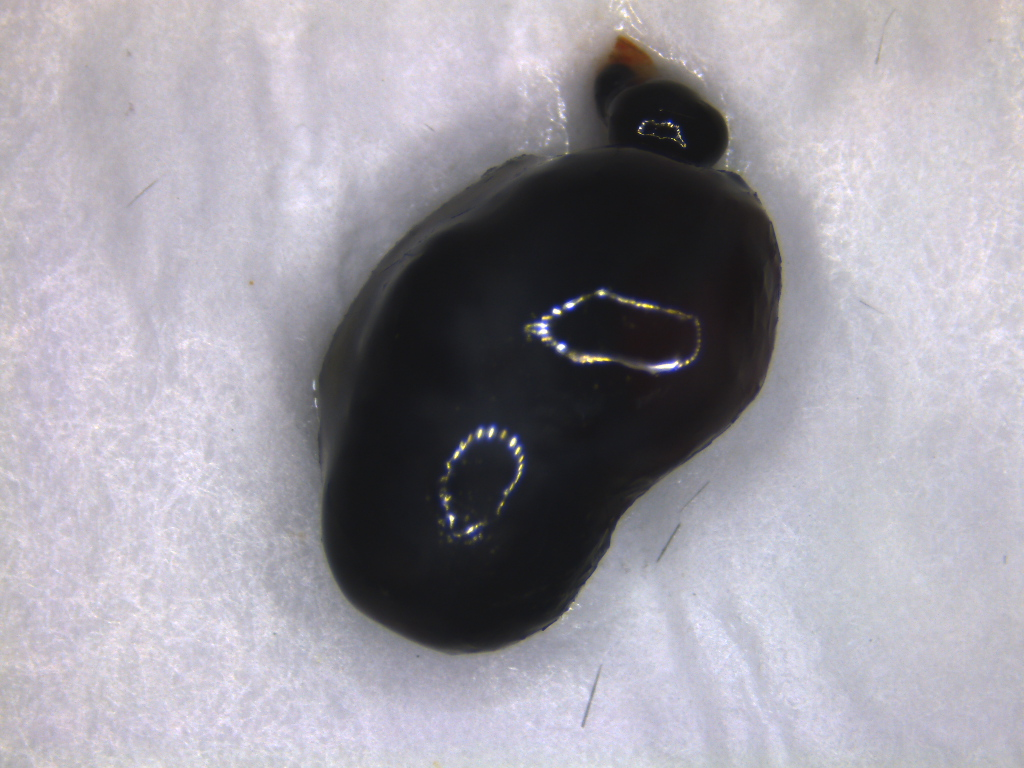

Supplement: Supplementary file 8 — Appendix Figure Source Data [file 44318_2024_78_MOESM8_ESM.zip › Appendix Figure/Appendix Figure S2/S2B/sGC╬öpc-3.tif]

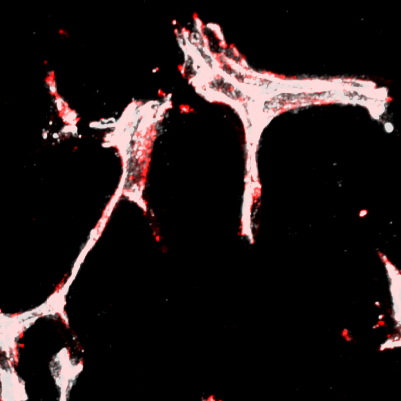

Supplement: Supplementary file 8 — Appendix Figure Source Data [file 44318_2024_78_MOESM8_ESM.zip › Appendix Figure/Appendix Figure S5/S5C/sGCCtr-3.tif]

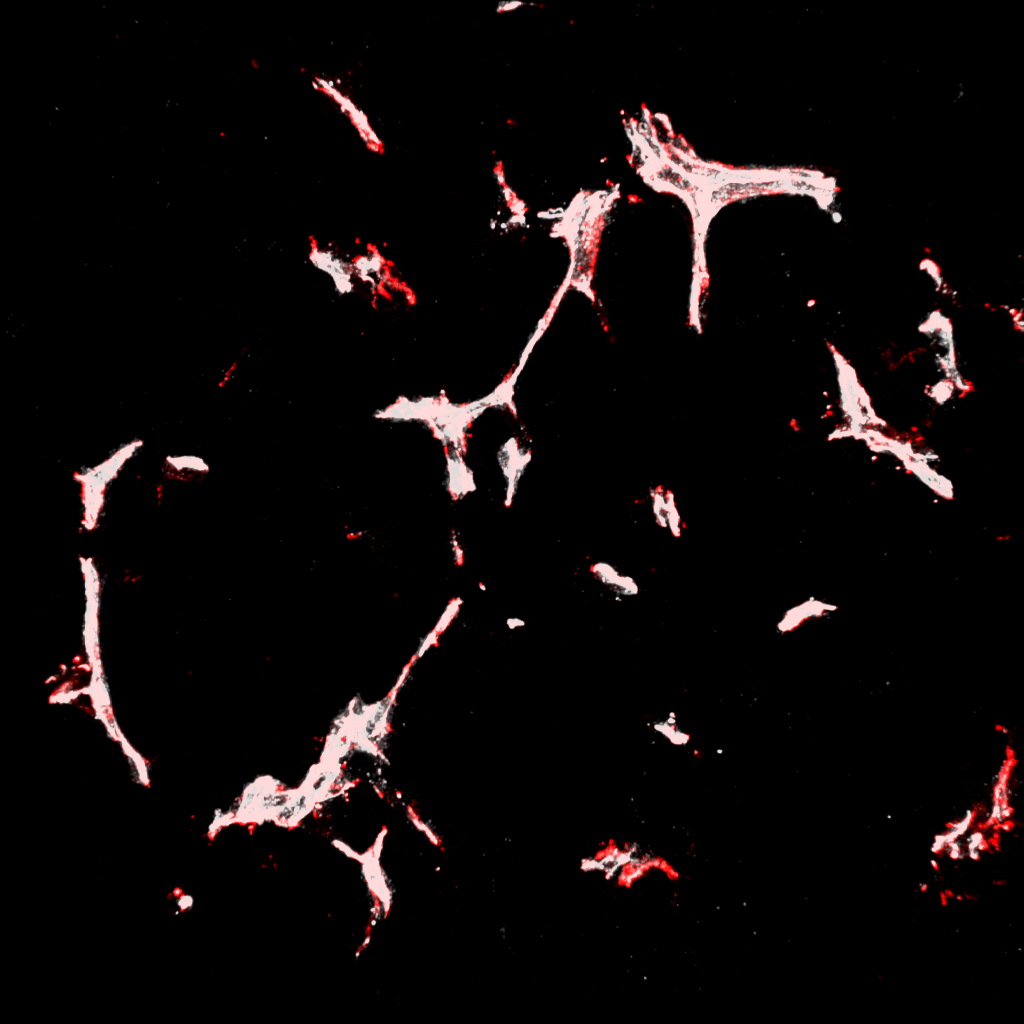

Supplement: Supplementary file 8 — Appendix Figure Source Data [file 44318_2024_78_MOESM8_ESM.zip › Appendix Figure/Appendix Figure S5/S5C/sGCCtr-2.tif]

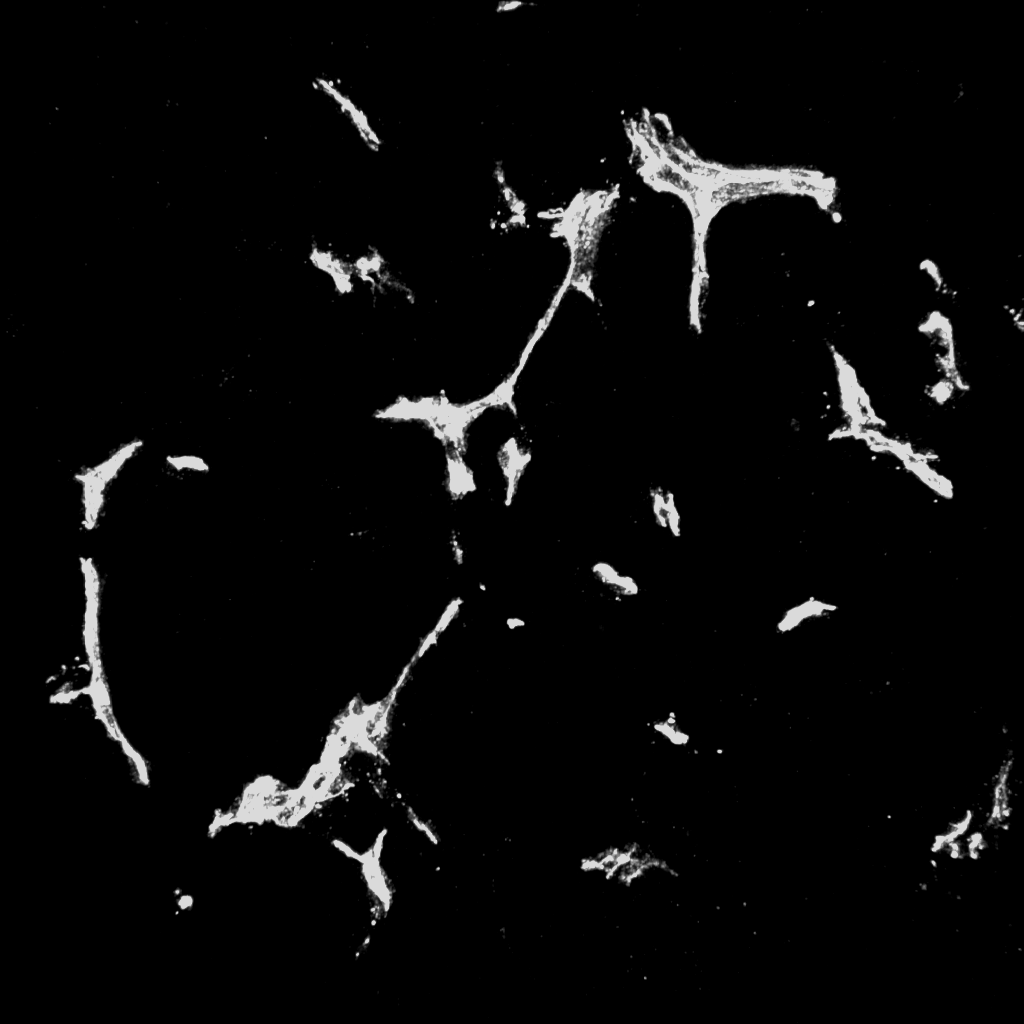

Supplement: Supplementary file 8 — Appendix Figure Source Data [file 44318_2024_78_MOESM8_ESM.zip › Appendix Figure/Appendix Figure S5/S5C/sGCCtr-1.tif]

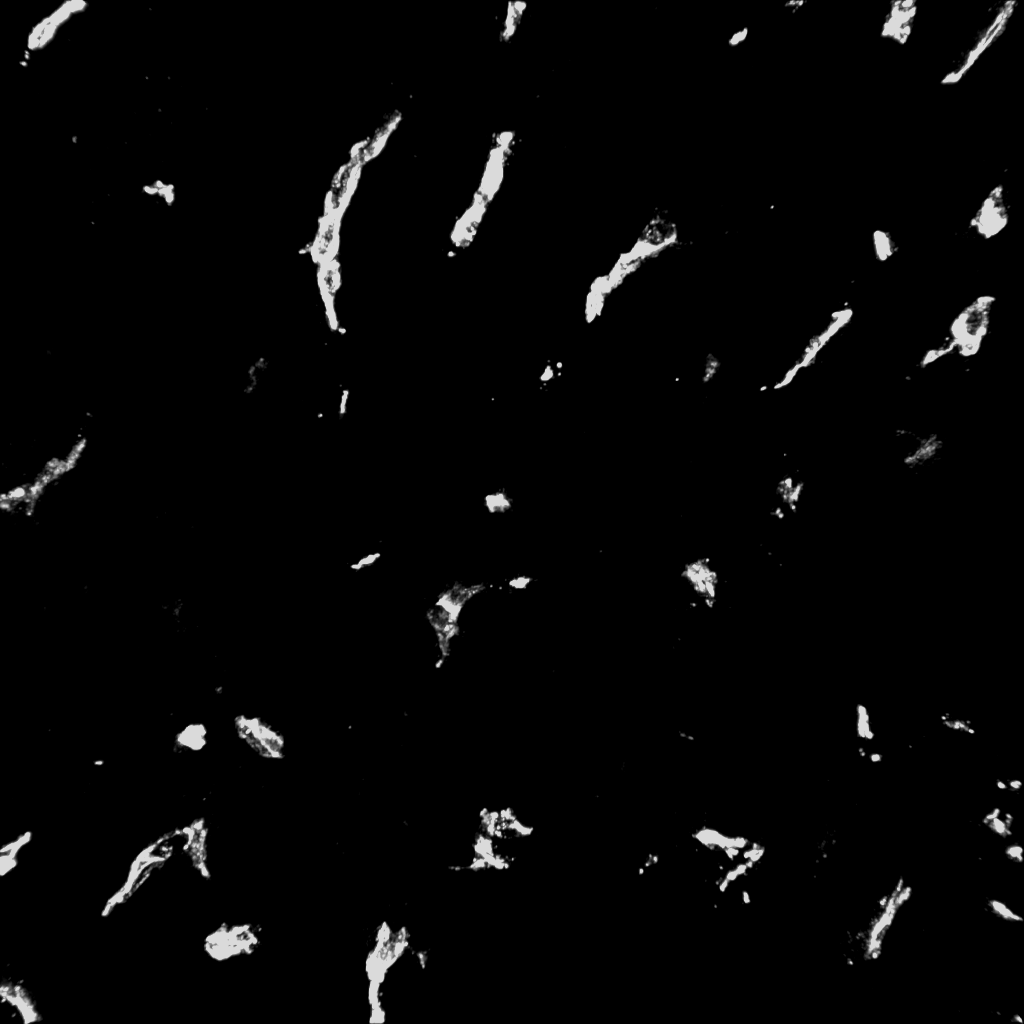

Supplement: Supplementary file 8 — Appendix Figure Source Data [file 44318_2024_78_MOESM8_ESM.zip › Appendix Figure/Appendix Figure S5/S5C/sGC╬öpc-1.tif]

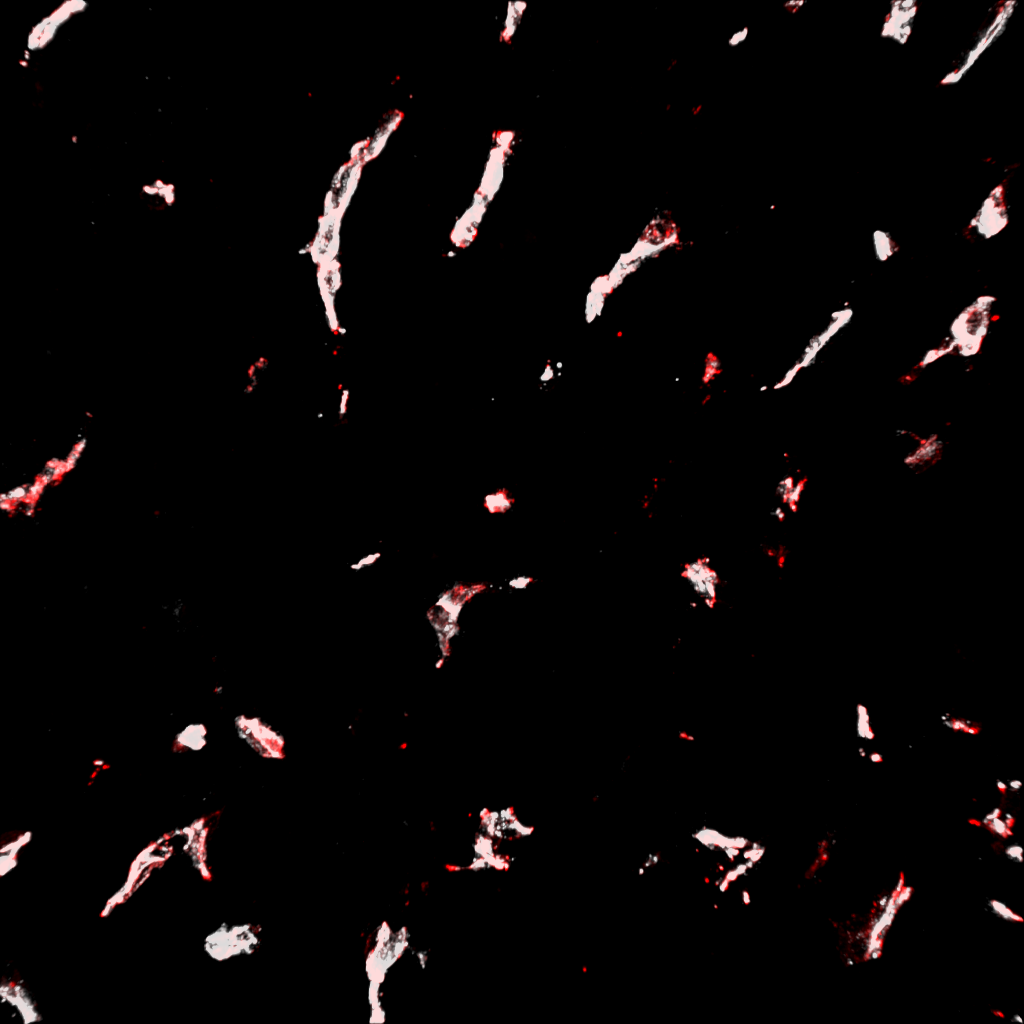

Supplement: Supplementary file 8 — Appendix Figure Source Data [file 44318_2024_78_MOESM8_ESM.zip › Appendix Figure/Appendix Figure S5/S5C/sGC╬öpc-2.tif]

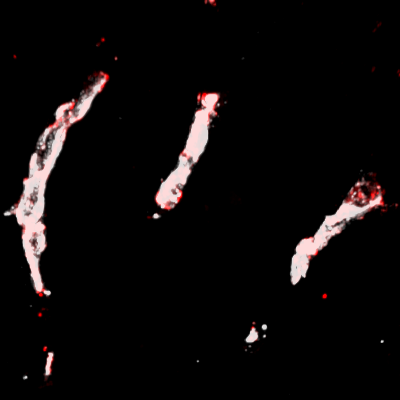

Supplement: Supplementary file 8 — Appendix Figure Source Data [file 44318_2024_78_MOESM8_ESM.zip › Appendix Figure/Appendix Figure S5/S5C/sGC╬öpc-3.tif]

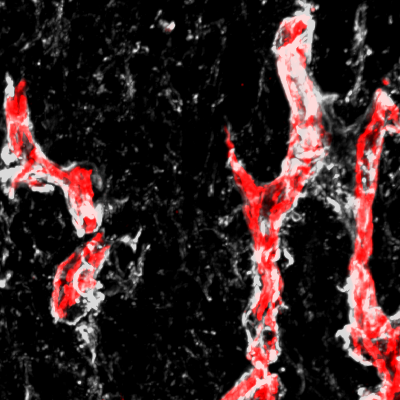

Supplement: Supplementary file 8 — Appendix Figure Source Data [file 44318_2024_78_MOESM8_ESM.zip › Appendix Figure/Appendix Figure S5/S5D/sGCCtr-3.tif]

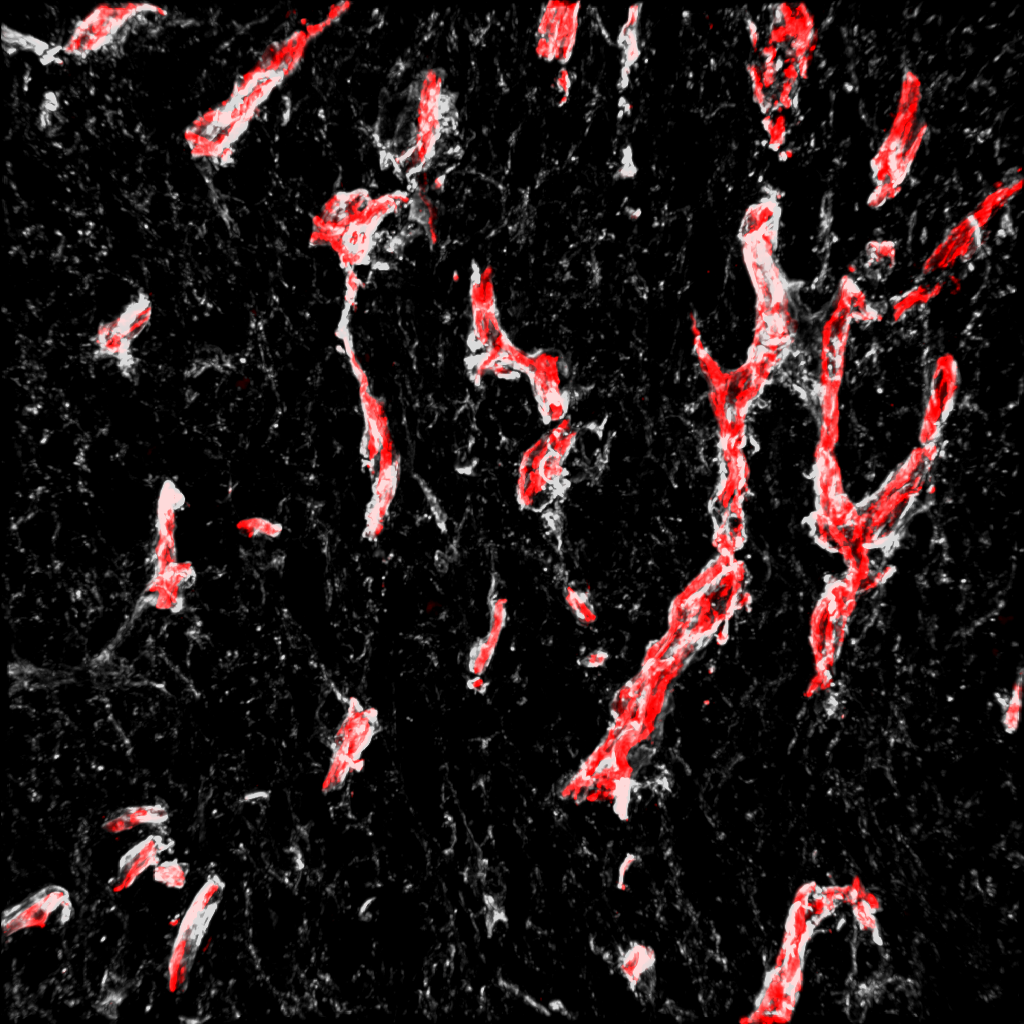

Supplement: Supplementary file 8 — Appendix Figure Source Data [file 44318_2024_78_MOESM8_ESM.zip › Appendix Figure/Appendix Figure S5/S5D/sGCCtr-2.tif]

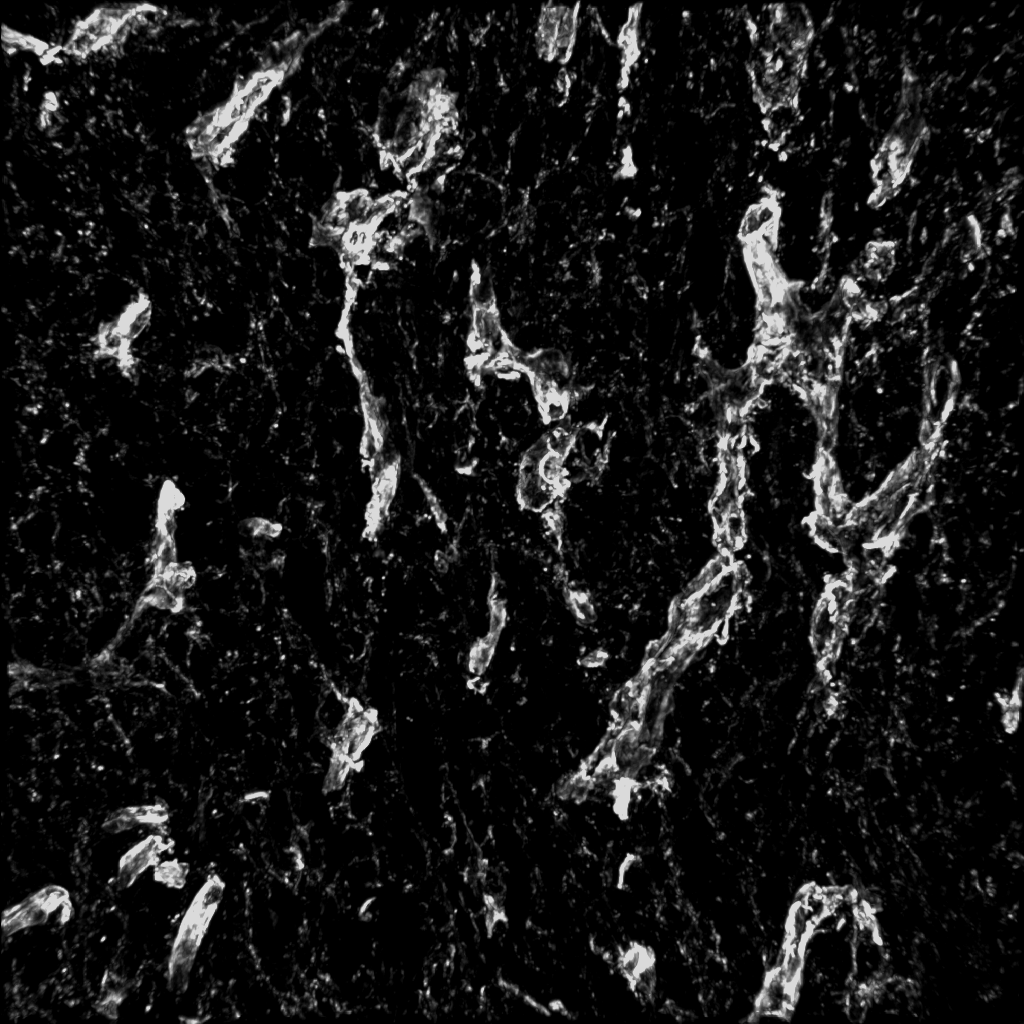

Supplement: Supplementary file 8 — Appendix Figure Source Data [file 44318_2024_78_MOESM8_ESM.zip › Appendix Figure/Appendix Figure S5/S5D/sGCCtr-1.tif]

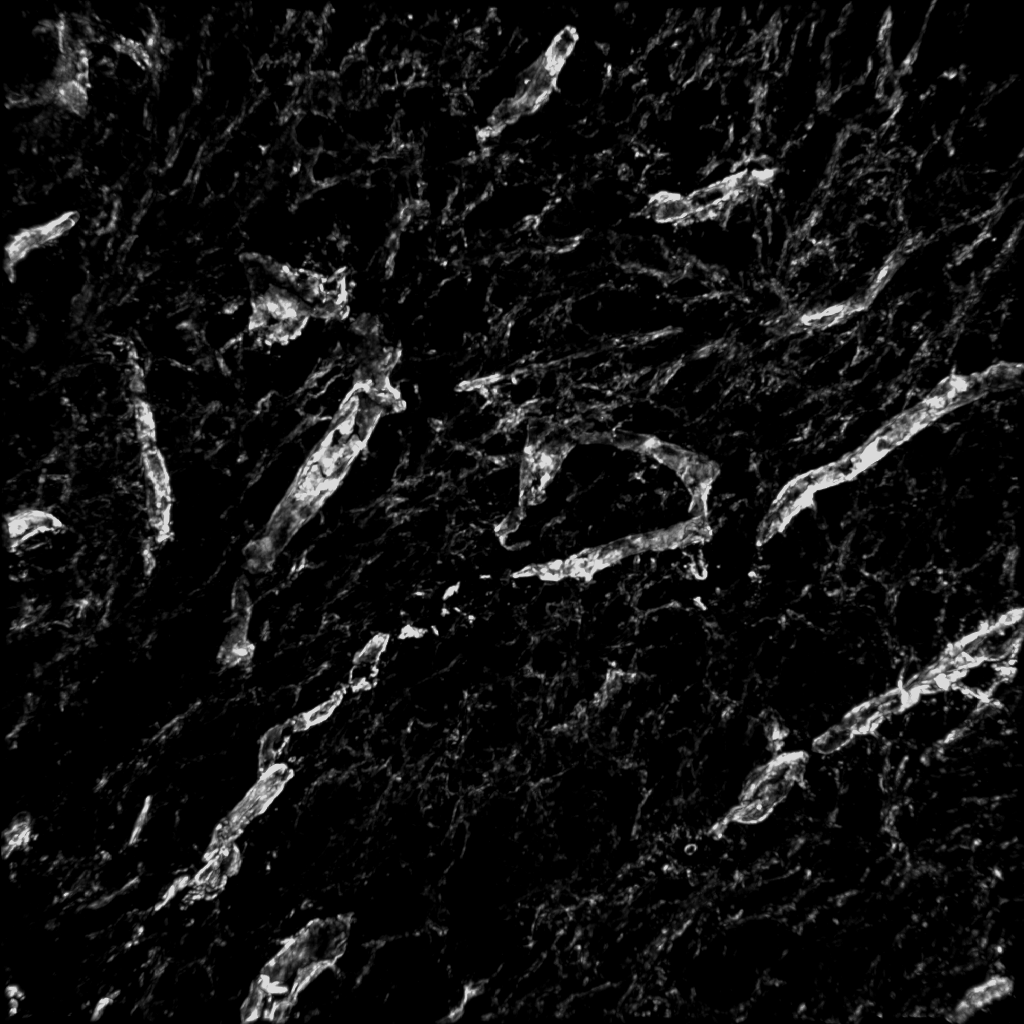

Supplement: Supplementary file 8 — Appendix Figure Source Data [file 44318_2024_78_MOESM8_ESM.zip › Appendix Figure/Appendix Figure S5/S5D/sGC╬öpc-1.tif]

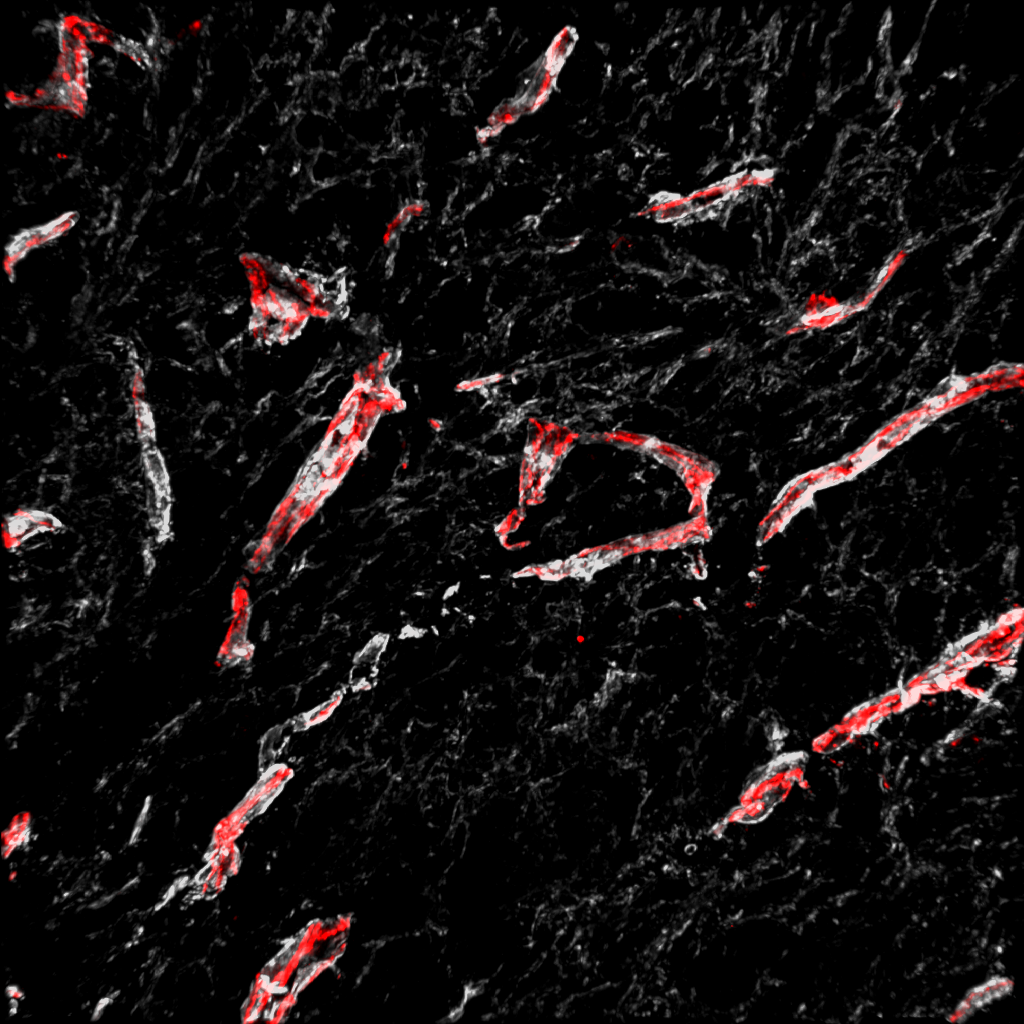

Supplement: Supplementary file 8 — Appendix Figure Source Data [file 44318_2024_78_MOESM8_ESM.zip › Appendix Figure/Appendix Figure S5/S5D/sGC╬öpc-2.tif]

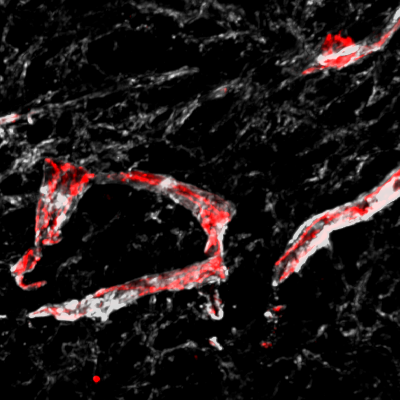

Supplement: Supplementary file 8 — Appendix Figure Source Data [file 44318_2024_78_MOESM8_ESM.zip › Appendix Figure/Appendix Figure S5/S5D/sGC╬öpc-3.tif]
