# Supplementary material for: Pericyte signaling via soluble guanylate cyclase shapes the vascular niche and microenvironment of tumors
Source: EMBO J. 2024 Mar 25;43(8):7. doi: 10.1038/s44318-024-00078-5 (PMC11021551; doi:10.1038/s44318-024-00078-5)
Supplement: Supplementary file 10 — Expanded View Figures [file 44318_2024_78_MOESM10_ESM.pdf]

Expanded View Figures

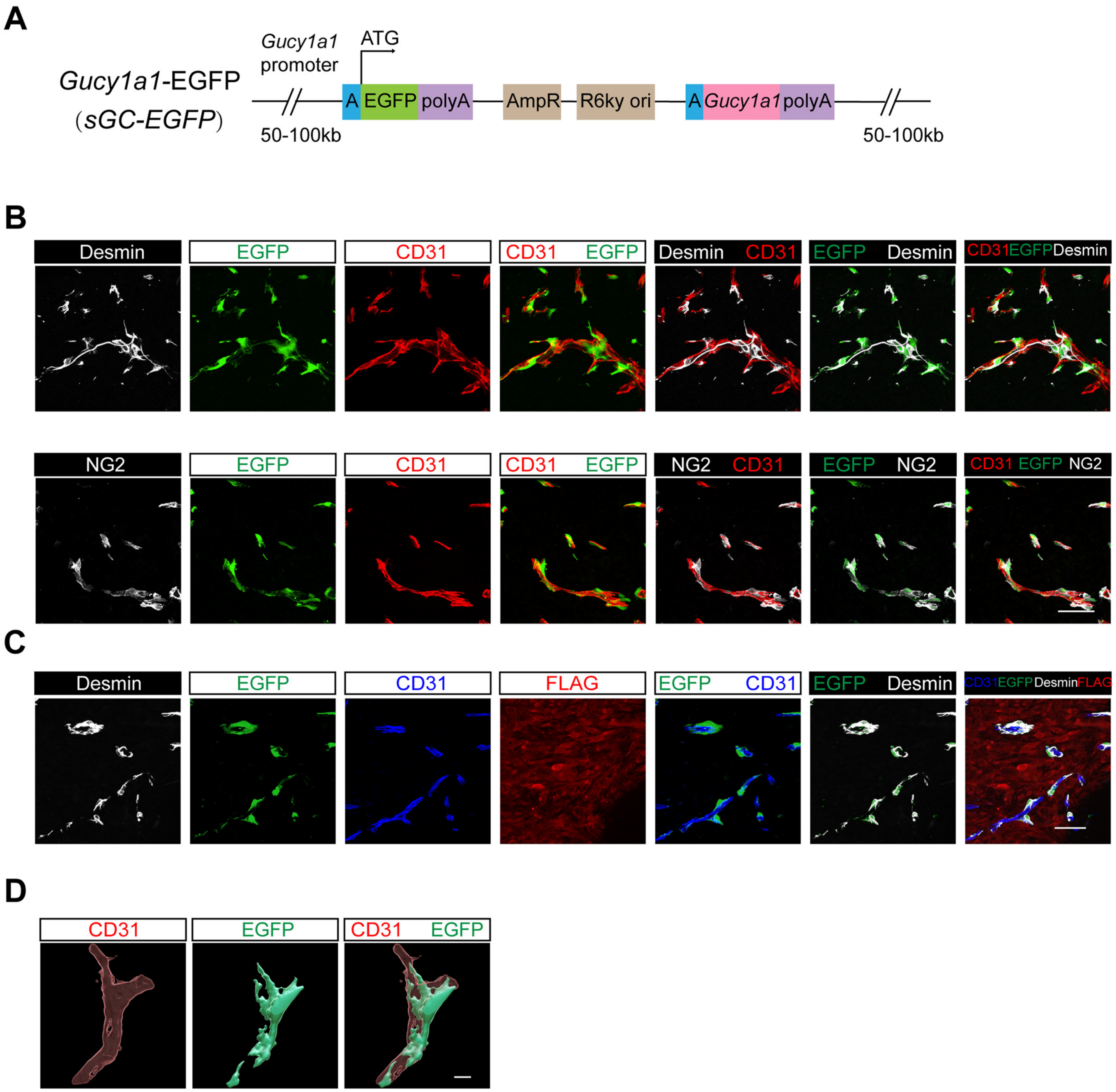

Figure EV1. **sGC is specifically expressed in tumor vascular pericytes.**

(**A**) Schematic depiction of the construction strategy of *Gucy1a1-EGFP* (*sGC-EGFP*) transgenic mice. (**B**) Representative fluorescent images showing CD31, NG2, and Desmin-stained LLC tumor sections from *sGC-EGFP* mice. Scale bar, 50 μm. (**C**) Representative fluorescent images showing CD31, Desmin, and Flag-stained tumor sections from sGC-EGFP mice, demonstrating that tumor cells transduced with Flag-luciferase did not express sGC, as evidenced by the lack of co-localization with the sGC-EGFP signal. Scale bar, 50 μm. (**D**) Three-dimensional reconstruction depicting EGFP-expressing pericyte wrapping around a capillary within LLC tumors. Scale bar, 8 μm. Source data are available online for this figure.

© The Author(s)

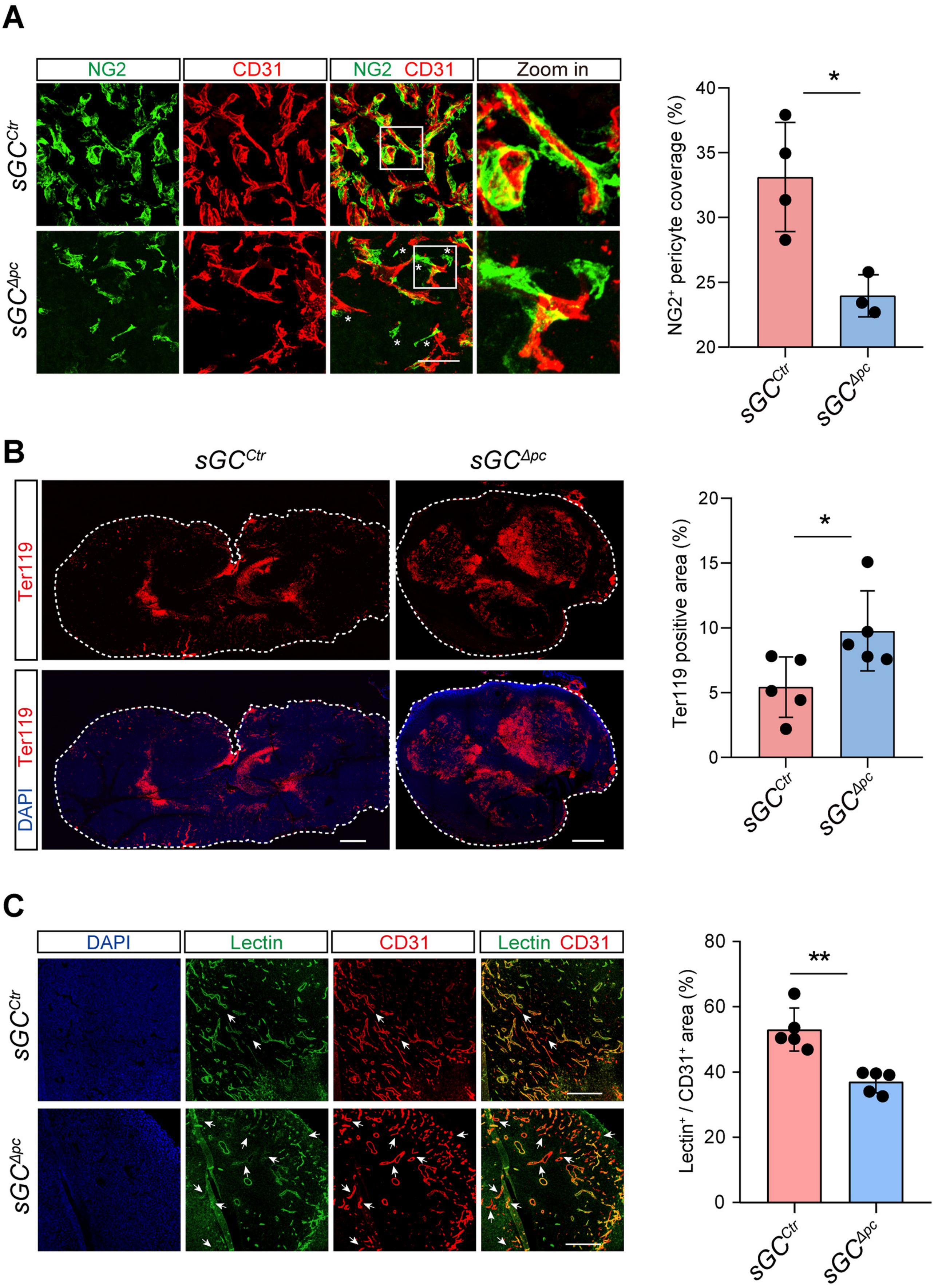
A
NG2
CD31
NG2 CD31
Zoom in
sGC^Ctr
sGC^Δpc
NG2+ pericyte coverage (%)
40
35
30
25
20
*
B
sGC^Ctr
sGC^Δpc
Ter119
DAPI Ter119
Ter119 positive area (%)
20
15
10
5
0
C
DAPI
Lectin
CD31
Lectin CD31
Lectin+ / CD31+ area (%)
80
60
40
20
0
**

© The Author(s)

◀ **Figure EV2. Pericyte-specific sGC inactivation impairs blood vessel stability in LLC tumors.**

(**A**) Representative fluorescent images depicting CD31 and NG2-stained tumor sections from sGC^{ctr} and $sGC^{\Delta PC}$ mice. Plot showing the percentage of vessels covered by NG2-positive pericytes. Data presented as mean ± SD, with $n = 3$–4 mice per group. Scale bar, 100 μm. (**B**) Representative fluorescent images displaying DAPI and Ter119-stained LLC tumor sections from sGC^{ctr} and $sGC^{\Delta PC}$ mice. Plot showing the Ter119-positive area in tumors. Data presented as mean ± SD, with $n = 5$ mice per group. Scale bar, 1 mm. (**C**) Representative fluorescent images displaying DAPI, Lectin and CD31-stained LLC tumor sections obtained from sGC^{ctr} and $sGC^{\Delta PC}$ mice. Arrows indicate non-perfused vessels. Plot showing the functional vessel area in tumors. Data presented as mean ± SD, with $n = 5$ mice per group. Scale bar, 300 μm. Statistical significance assessed using two-tailed Student's t test (**A**–**C**). $^*P < 0.05$; $^{**}P < 0.01$. Source data are available online for this figure.

© The Author(s)

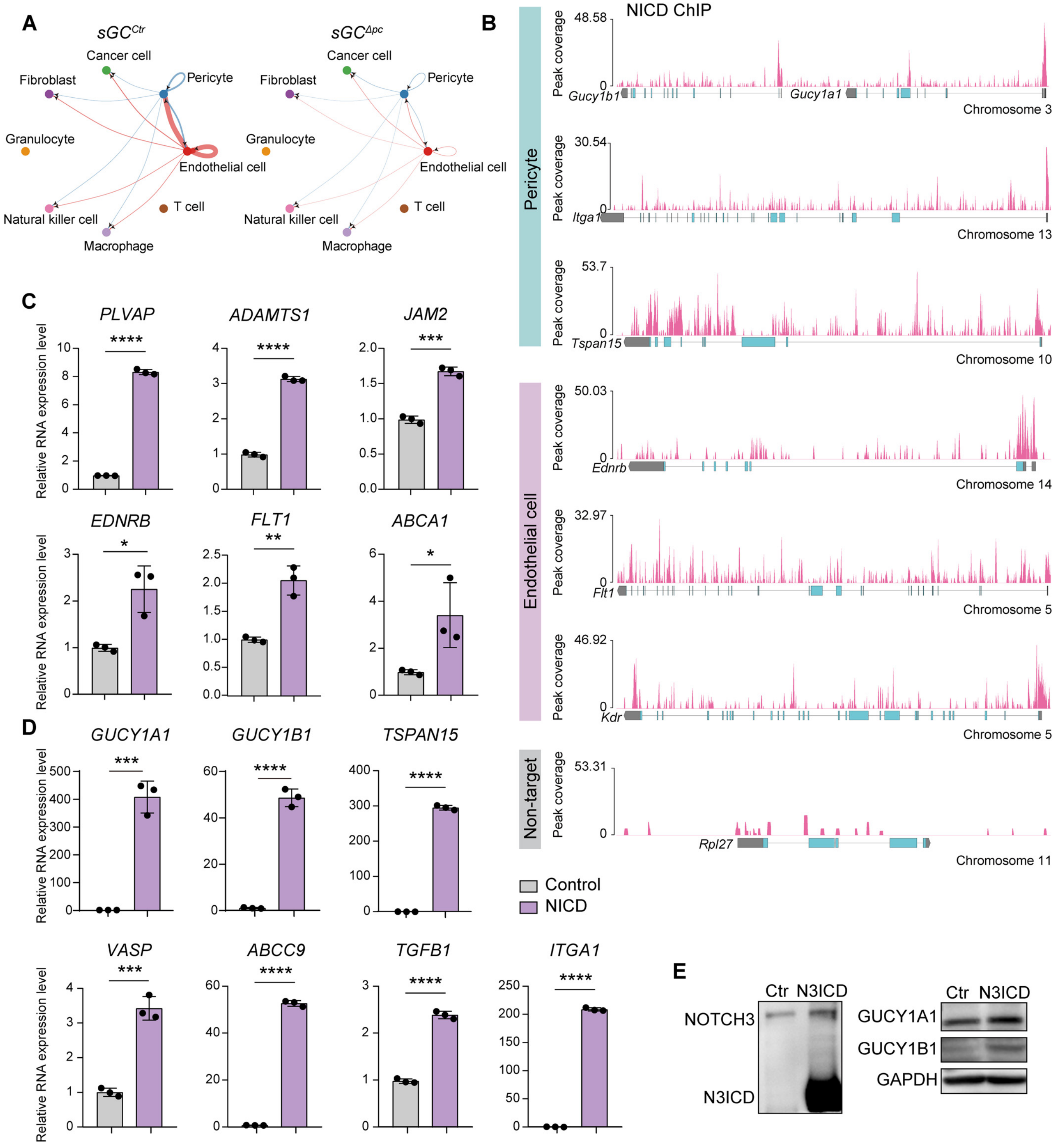

Figure EV3. Pericyte-specific sGC inactivation alters Notch signaling pathway.

(**A**) Circle plot showing the Notch signaling interactions among different cell populations within the tumors. Arrows indicate the direction of ligand-to-receptor interaction, while edge thickness reflects the cumulative weighted pathways between cell populations. (**B**) Analysis of NICD ChIP-seq dataset (GSE34954, comparing NICD-overexpressing cells to cells lacking Notch expression) reveals the enrichment of NICD at the promoter regions of *Gucy1a1, Gucy1b1, Itga1, Tspan15, Ednrb, Flt1, Kdr*, and *Rpl27*. The non-targeting gene *Rpl27* was included as a reference to confirm the specificity of NICD binding. (**C, D**) qPCR analysis of gene expression in control and NICD-overexpressing HUVECs (**C**) and HBVPs (**D**). Data presented as mean ± SD, with $n = 3$ replicates. (**E**) Western blot analysis of GUCY1A1 and GUCY1B1 protein levels in control and NICD-overexpressing HBVPs. Data presented as mean ± SD, with $n = 3$ replicates. Statistical significance assessed using two-tailed Student's *t* test (**C, D**). $^*P < 0.05$; $^{**}P < 0.01$; $^{***}P < 0.001$; $^{****}P < 0.0001$. Source data are available online for this figure.

© The Author(s)

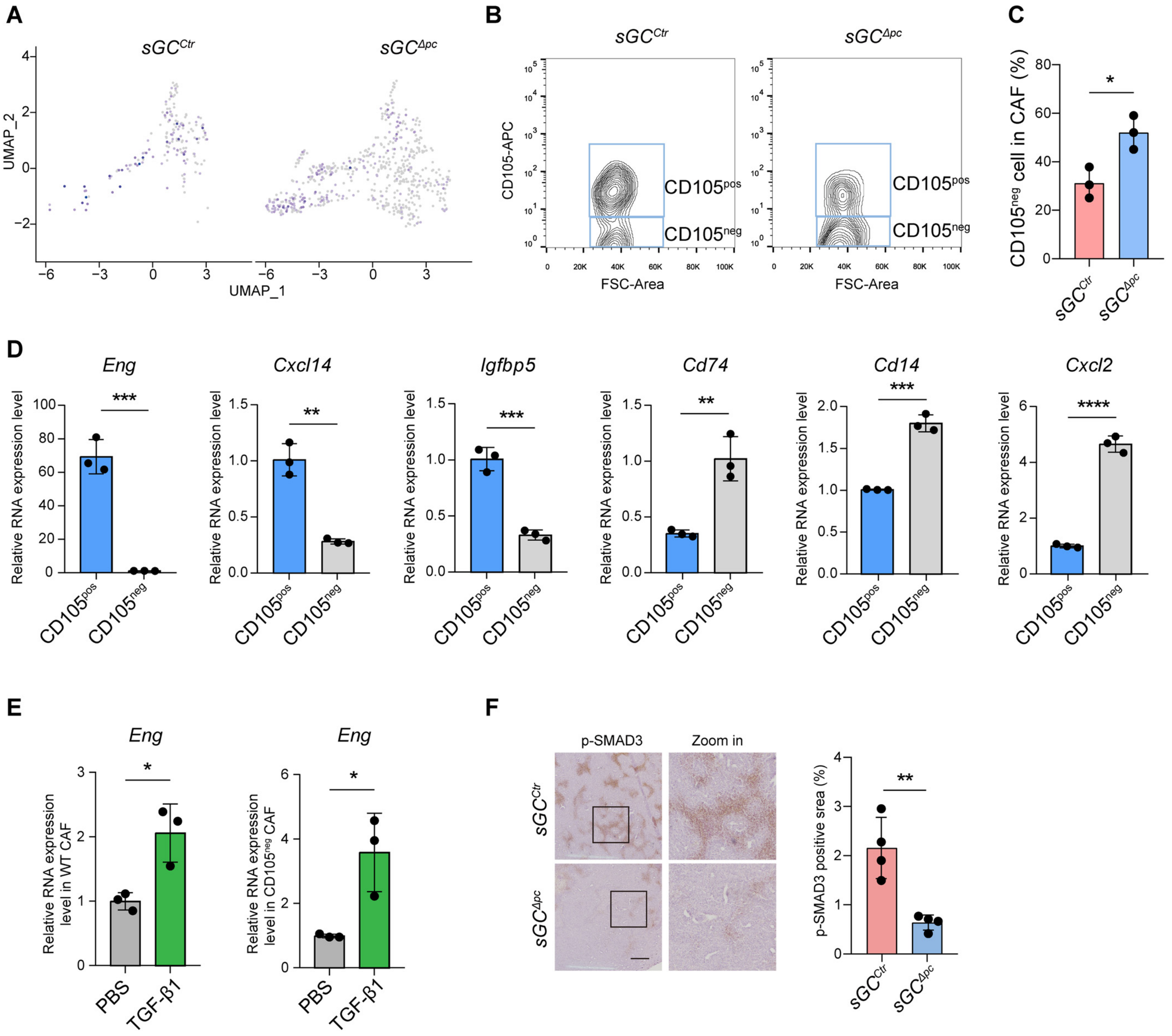

Figure EV4. CD105^{pos} and CD105^{neg} CAFs identification.

(**A**) UMAP plots showing *Eng* expression levels in CAFs. (**B**) FACS gating strategy employed for CD105^{pos} and CD105^{neg} CAFs isolation. (**C**) Plots showing the proportion of CD105^{neg} CAFs in *sGC^{ctr}* and *sGC$^{\Delta PC}$* mice, Data presented as mean ± SD, with $n = 3$ mice per group. (**D**) qPCR analysis of the expression levels of *Eng, Cd74, Igfbp5, Cxcl14, Cd14*, and *Cxcl2* in CD105^{pos} and CD105^{neg} CAFs. CD105^{pos} and CD105^{neg} CAFs were isolated from five LLC tumors and pooled to ensure an adequate cell number for qPCR analysis. The plot depicts one representative dataset from two independent experimental repeats. Data presented as mean ± SD, with $n = 3$ technical replicates. (**E**) qPCR analysis of *Eng* expression in total CAFs and CD105^{neg} CAFs after TGF-β1 treatment. Data presented as mean ± SD. with n = 3 replicates. (**F**) Representative immunohistochemistry staining of pSMAD3 in *sGC^{ctr}* and *sGC$^{\Delta PC}$* LLC tumors. The plot illustrates the quantification of the pSMAD3 positive area. Data presented as mean ± SD, with $n = 4$ mice per group. Scale bar, 500 μm. Statistical significance assessed using two-tailed Student's *t* test (**C–F**). *$P < 0.05$; **$P < 0.01$; ***$P < 0.001$; ****$P < 0.0001$. Source data are available online for this figure.

© The Author(s)

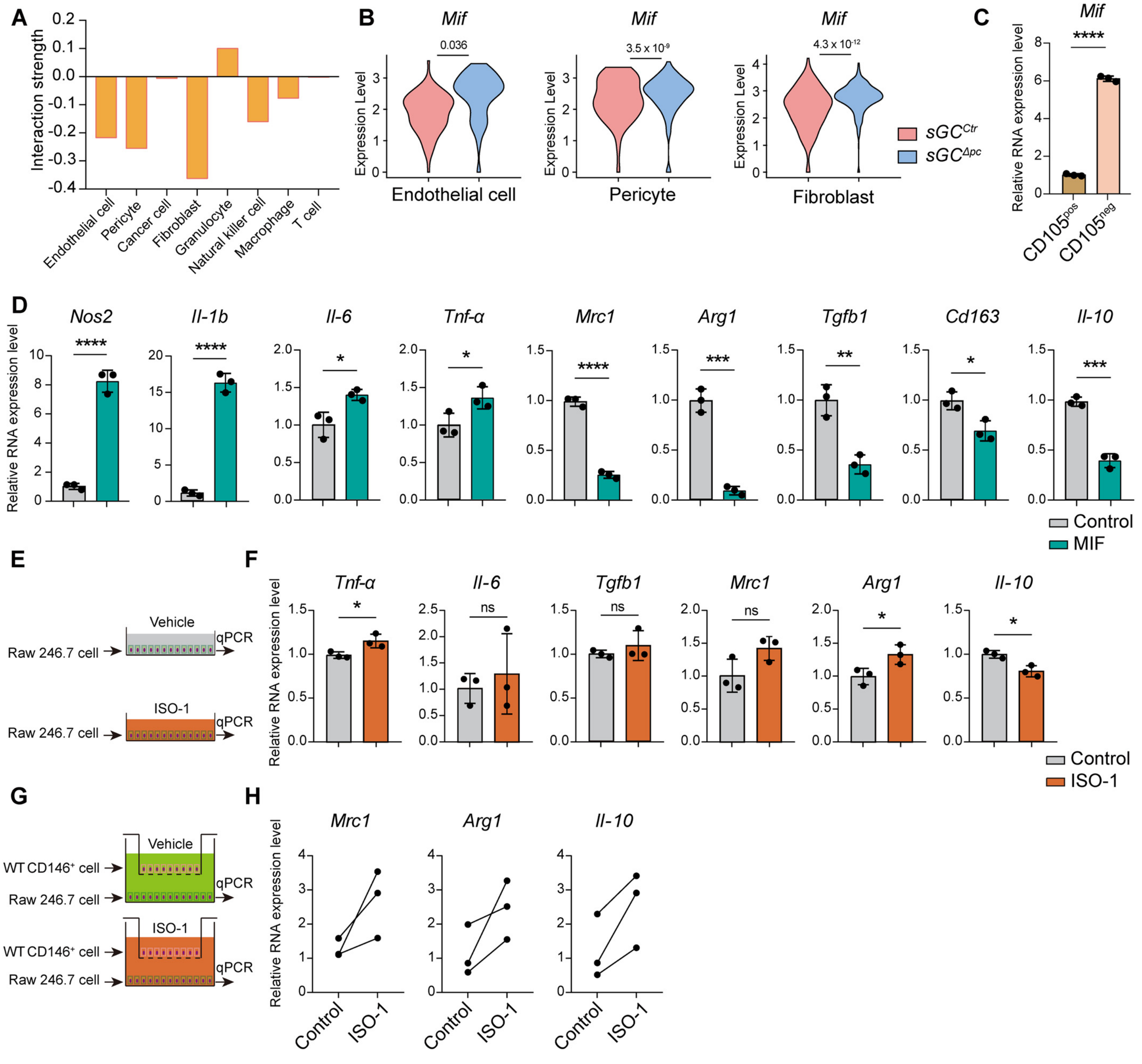

Figure EV5. MIF promotes macrophage M1-like polarization.

(**A**) The alteration in cell–cell interaction strength between TAMs and other cellular components in the tumors following pericyte-specific sGC inactivation. (**B**) Violin plots showing the expression levels of *Mif* in ECs, pericytes, and CAFs within *sGC^ctr* and *sGC^ΔPC* tumors. (**C**) Plot showing *Mif* expression levels in CD105^pos and CD105^neg CAFs. To obtain a sufficient number of cells, CD105^pos and CD105^neg CAFs were isolated from five LLC tumors and subsequently pooled for the experiment. Data presented as mean ± SD. (**D**) qPCR analysis of gene expression levels of *Nos2, Il-1b, Il-6, Tnf-a, Mrc1, Arg1, Tgfb1, Cd163*, and *Il-10* in Raw246.7 cells treated with either MIF or vehicle. Data presented as mean ± SD, with n = 3 replicates. (**E**) Schematic depiction of the experimental design. Raw246.7 cells were treated with either vehicle or ISO-1. (**F**) qPCR analysis of gene expression levels of *Il-6, Tnf-a, Mrc1, Arg1, Tgfb1*, and *Il-10* in Raw246.7 cells treated with ISO-1 or vehicle. Data presented as mean ± SD, with *n* = 3 replicates. (**G**) Schematic depiction of the experimental design. WT CD146$^+$ cells were seeded into the transwell upper chamber, Raw246.7 cells in the bottom chamber. The culture medium was supplemented with either vehicle or ISO-1. (**H**) qPCR analysis of gene expression levels of *Mrc1, Arg1*, and *Il-10* in Raw246.7 cells treated with ISO-1 or vehicle. Data presented as mean ± SD, with *n* = 3 mice per group. Statistical significance assessed using unpaired two-samples Wilcoxon test (**B**), two-tailed Student's *t* test (**C**, **D**, **F**). *$P < 0.05$; **$P < 0.01$; ***$P < 0.001$; ****$P < 0.0001$. Source data are available online for this figure.

© The Author(s)
